# Supplementary material for: Alternative splicing regulation in plants by SP7-like effectors from symbiotic arbuscular mycorrhizal fungi
Source: Nat Commun. 2024 Aug 19;15:7107. doi: 10.1038/s41467-024-51512-5 (PMC11333574; doi:10.1038/s41467-024-51512-5)
Supplement: Supplementary file 1 — Supplementary Information [file 41467_2024_51512_MOESM1_ESM.pdf]

Supplementary Information

Alternative splicing regulation in plants by SP7-like effectors from symbiotic arbuscular mycorrhizal fungi

Ruben Betz[1*], Sven Heidt[1*], David Figueira-Galán[1], Meike Hartmann[1], Thorsten Langner[2] and Natalia Requena[1,a]

[1] Joseph Kölreuter Institute for Plant Sciences. Molecular Phytopathology Department, Karlsruhe Institute of Technology (KIT) - South Campus, Fritz-Haber-Weg 4, Karlsruhe 76131, Germany
[2] Max Planck Institute for Biology Tübingen - Max-Planck-Ring 5, Tübingen 72076, Germany

* These authors contributed equally
[a] Corresponding author: natalia.requena@kit.edu

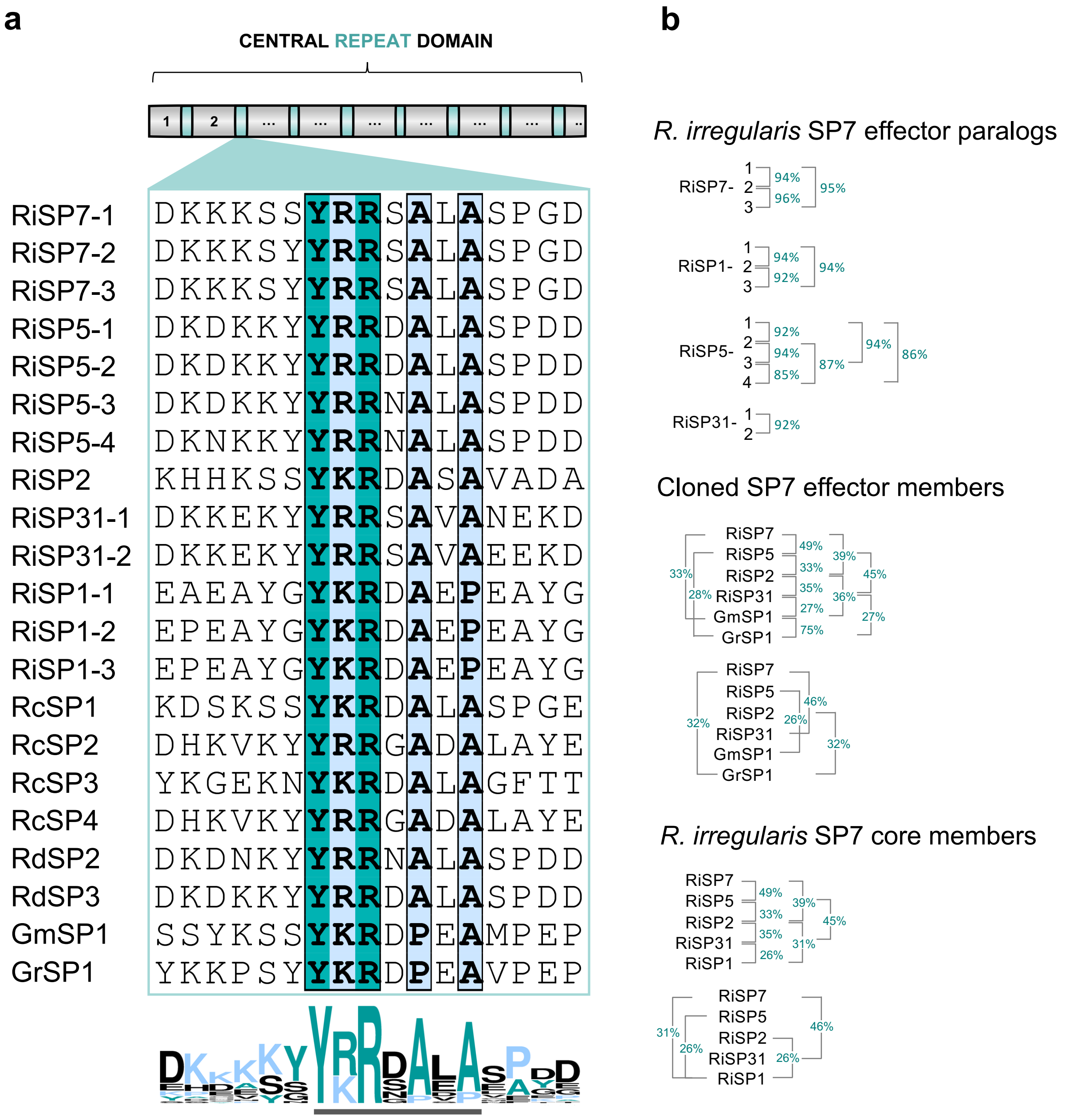
a
b
CENTRAL REPEAT DOMAIN
1
2
RiSP7-1 DKKKSSYRRSALASPGD
RiSP7-2 DKKKSYYRRSALASPGD
RiSP7-3 DKKKSYYRRSALASPGD
RiSP5-1 DKDKKYYRRDALASPDD
RiSP5-2 DKDKKYYRRDALASPDD
RiSP5-3 DKDKKYYRRNALASPDD
RiSP5-4 DKNKKYYRRNALASPDD
RiSP2 KHHKSSYKRDASAVADA
RiSP31-1 DKKEKYYRRSAVANEKD
RiSP31-2 DKKEKYYRRSAVAEEKD
RiSP1-1 EAEAYGYKRDAEPEAYG
RiSP1-2 EPEAYGYKRDAEPEAYG
RiSP1-3 EPEAYGYKRDAEPEAYG
RcSP1 KDSKSSYKRDALASPGE
RcSP2 DHKVKYYRRGADALAYE
RcSP3 YKGEKNYKRDALAGFTT
RcSP4 DHKVKYYRRGADALAYE
RdSP2 DKDNKYYRRNALASPDD
RdSP3 DKDKKYYRRDALASPDD
GmSP1 SSYKSSYKRDPEAMPEP
GrSP1 YKKPSYYKRDPEAVPEP
R. irregularis SP7 effector paralogs
RiSP7- 1 2 3 94% 96% 95%
RiSP1- 1 2 3 94% 92% 94%
RiSP5- 1 2 3 4 92% 94% 85% 87% 94% 86%
RiSP31- 1 2 92%
Cloned SP7 effector members
RiSP7
RiSP5
RiSP2
RiSP31
GmSP1
GrSP1
49% 33% 35% 27% 75% 39% 36% 45% 27% 33% 28%
46% 26% 32% 32%
R. irregularis SP7 core members
RiSP7
RiSP5
RiSP2
RiSP31
RiSP1
49% 33% 35% 26% 39% 31% 45%
31% 26% 46% 26%

Supplementary Figure 1. Identification of SP7 family characteristic conserved islands and effector motif.

a All identified full length SP7-like effectors (Supplementary Table 2) contain family conserved amino acid stretches (islands) within each repeat subunits. Shown is an alignment of the conserved islands from the 2nd repeat of each effector member. Ri (*Rhizophagus irregularis*), Rc (*Rhizophagus clarus*), Rd (*Rhizophagus diaphanus*), Gm (*Gigaspora margarita*), Gr (*Gigaspora rosea*). Bottom Logo represents consensus sequence (Weblogo) of the conserved islands, revealing the newly identified SP7 effector family motif Y-[KR]-R-X-[AP]-X-[AP] (underlined). Aligned sequences were visualized using ESpript3.0 (Blosom62 score). Identical amino acids are highlighted with bold letters, green background and conserved amino acids with bold letters, blue background. **b** Comparison of percentage protein identities between *R. irregularis* SP7-like paralogs, cloned and functionally analyzed SP7-like members and SP7-like core effectors present in *R. irregularis* after multiple ClustalO alignments. For protein identities between all identified members see Supplementary Table 2.

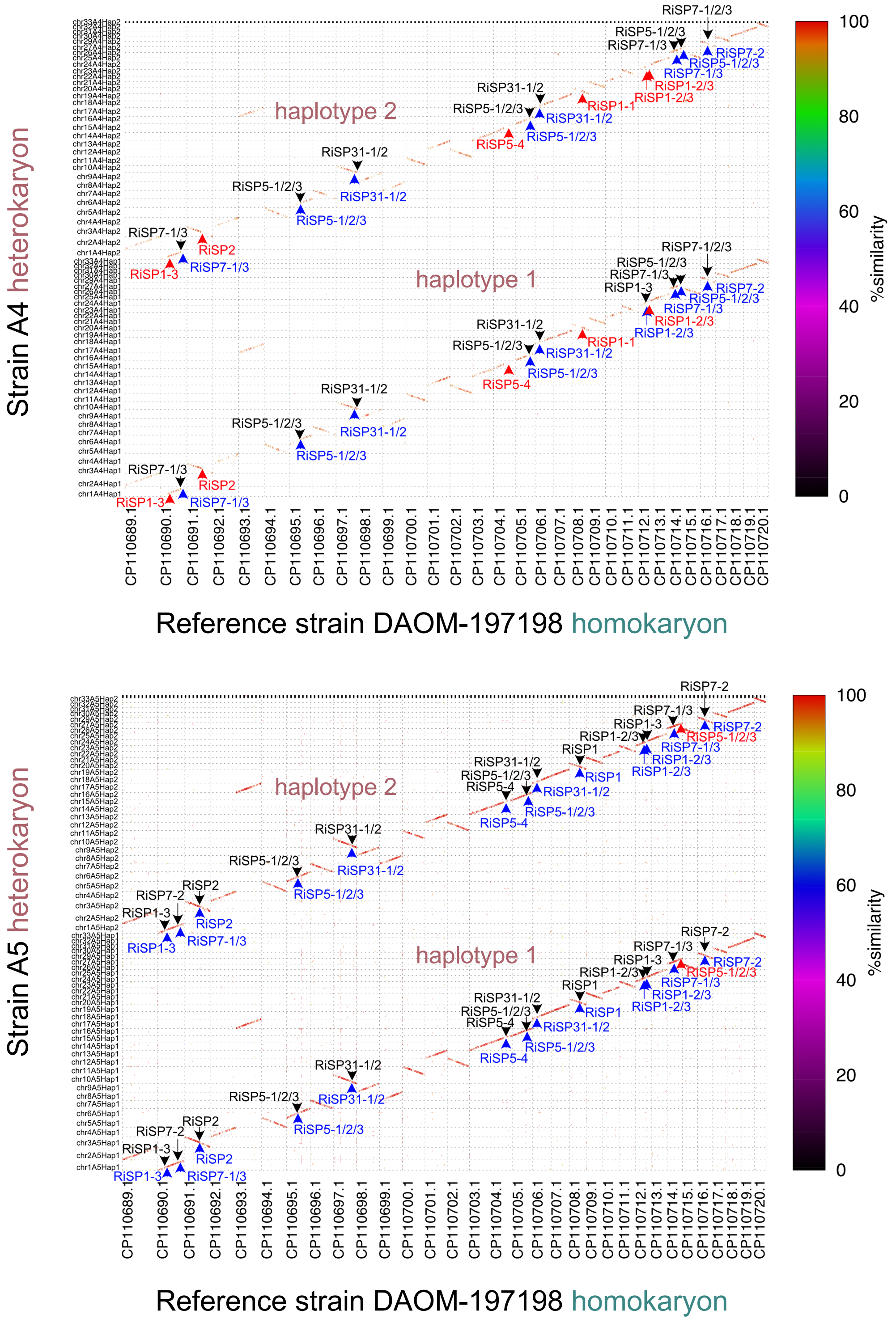

Supplementary Figure 2. SP-like effectors show extensive gene loss in heterokaryotic strains.

Whole genome alignments comparing nine publicly available genomes to the reference strain DAOM-197198 assembly[1]. Illustrated are two example pairwise dotplot comparisons between A4 or A5 heterokaryon and the homokaryotic refrence strain DAOM-197198. Alignments were generated using nucmer and viualized using mummerplot and gnuplot. Arrows indicate the coordinates of SP7-like effector genes. Blue color indicates effector genes in the reference assembly, black indicates effectors in query assemblies. Red color indicates presence/absence variation between the reference and query assemblies. Dotplot alignments and karyoplots of the remaining genomes are available in the source data.

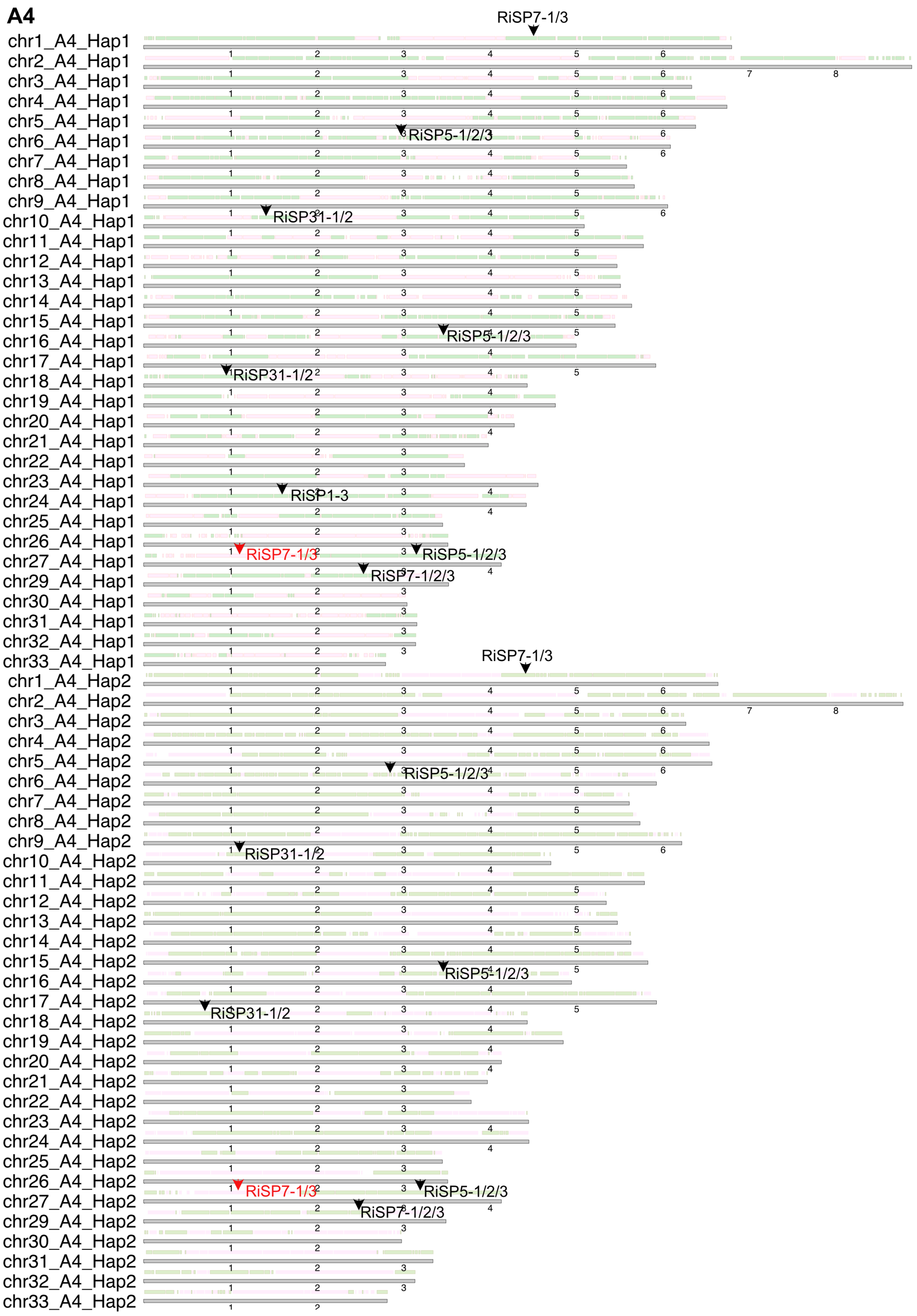

Supplementary Fig. 3. Genomic location of SP-like genes in the heterokaryotic strain A4 of *R. irregularis*.

The karyotype and location of SP7-like effector genes was plotted using the R package KaryoploteR. Effector coordinates were extracted from BLASTN-based sequence homology searches. Arrows indicate the location of effector genes. Red color indicates SP7-like effctors not locating in the B compartment. In the plot green denotes the B and pink the A compartment regions according to previous classifications[2]. Most effectors are in the B compartment in this strain. The exception is SP7 (AXY96529.1) on Chr 27 which is located in the A compartment.

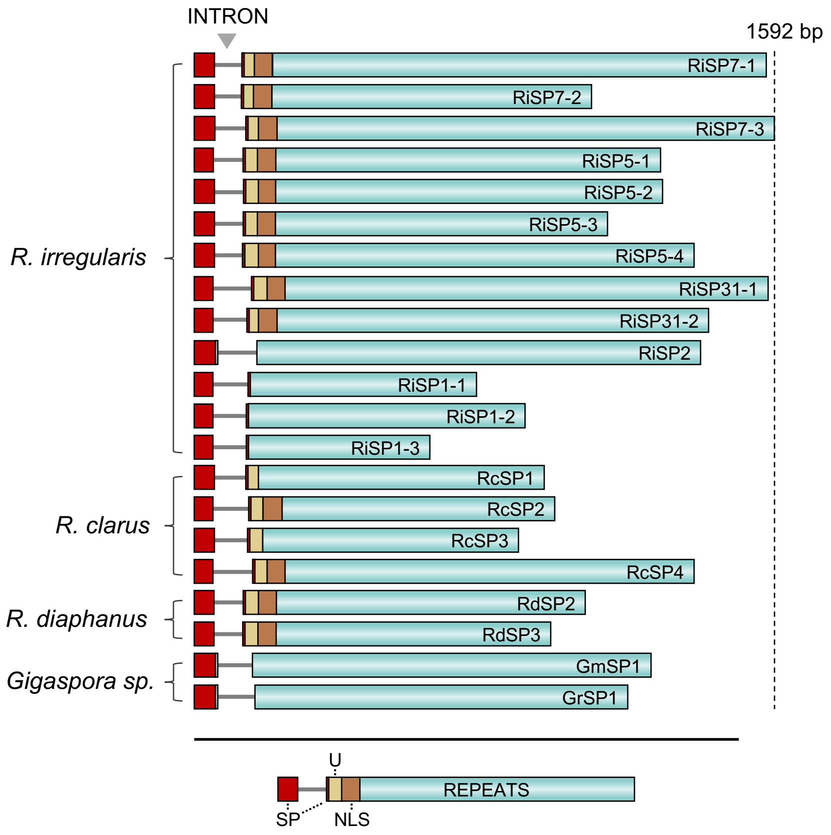

Supplementary Figure 4. Analysis of the SP7-like effector family gene organization reveals position conserved introns.

Illustrated are the coding sequences of all identified full lenght SP7 family effectors (Supplementary Table 2) from four different Glomeromycotina fungal species. All family members contain a single intron at the N-terminal region prior to the central repeat regions. For most effectors, the intron disrupts the SP coding sequences. Signal peptide (SP) highlighted in red, conserved domain with unknown function (U) in yellow, NLS in brown and repeat regions in green. Gene illustrations are drawn to scale.

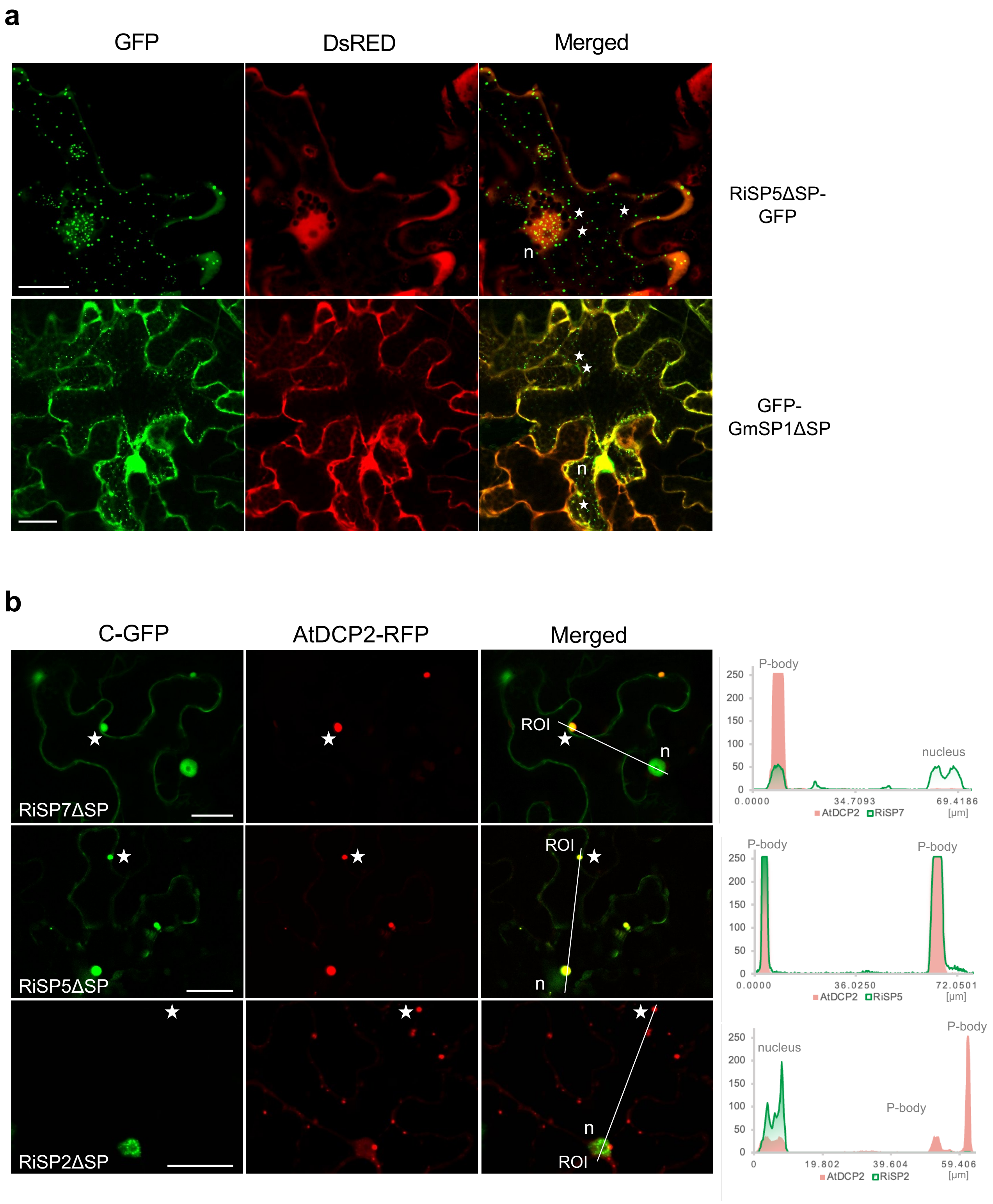
a
GFP
DsRED
Merged
RiSP5ΔSP-GFP
GFP-GmSP1ΔSP
n
b
C-GFP
AtDCP2-RFP
Merged
RiSP7ΔSP
ROI
n
P-body
nucleus
34.7093
69.4186
AtDCP2
RiSP7
RiSP5ΔSP
P-body
36.0250
72.0501
RiSP5
RiSP2ΔSP
nucleus
19.802
39.604
59.406
RiSP2
[μm]

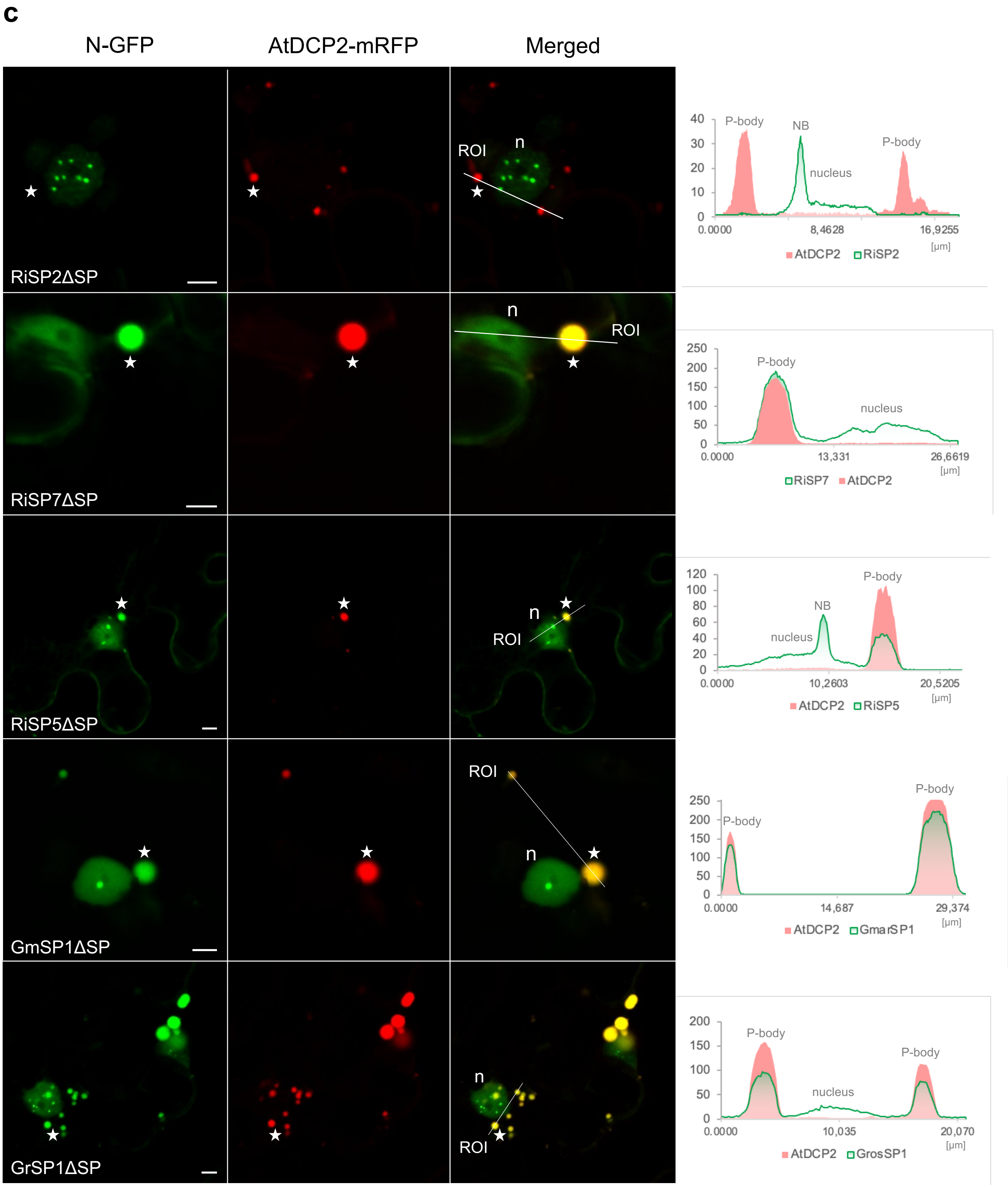
c
N-GFP
AtDCP2-mRFP
Merged
RiSP2ΔSP
ROI
n
P-body
NB
nucleus
P-body
0.0000
8,4628
16,9255
[μm]
AtDCP2
RiSP2
RiSP7ΔSP
n
ROI
P-body
nucleus
0.0000
13,331
26,6619
[μm]
RiSP7
AtDCP2
RiSP5ΔSP
n
ROI
P-body
NB
nucleus
0.0000
10,2603
20,5205
[μm]
AtDCP2
RiSP5
GmSP1ΔSP
ROI
n
P-body
P-body
0.0000
14,687
29,374
[μm]
AtDCP2
GmarSP1
GrSP1ΔSP
n
ROI
P-body
P-body
nucleus
0.0000
10,035
20,070
[μm]
AtDCP2
GrosSP1

Supplementary Figure 5. Co-localization study of SP7 family members with AtDCP2 P-body marker in *N. benthamiana*.

a Example pictures of cytoplasmic condensates occasionally found in a few cells for some SP7-like effectors. Shown are RiSP5ΔSP with C-terminal GFP fusion and GmSP1ΔSP with N-terminal GFP fusion expressed together with free DsRed as control to visualize nucleus and cytoplasm in *N. benthamiana* epidermal cells. Cytoplasmic condensates were absent from the DsRed channel. Exemplary cytoplasmic condensates for RiSP5ΔSP and GmSP1ΔSP are marked with stars. Scale bars represent 25 µm; n = nucleus. **b-c** Co-localization of SP7-like effectors without SP and fused to either C-terminal GFP (b, C-GFP) or N-terminal GFP (c, N-GFP) together with P-body marker AtDCP2 C-terminally fused to mRFP in *N. benthamiana* epidermal cells. With exception of nuclear localized RiSP2ΔSP, all tested effectors localized to the same cytoplasmic P-bodies marked by AtDCP2. Exemplary P-bodies are marked with stars. Fluorescence intensity blots generated from individual GFP and mRFP channels along transection lines are shown next to each panel. At positions of mRFP marked P-bodies, increase of GFP fluorescence can be observed for all SP7-like proteins but RiSP2. Scale bars represent 25 µm (b) and 5 µm (c). ROI = start of intensity measurement. NB = nuclear body. n = nucleus. All expression construct (a-c) under control of the 35S promoter.

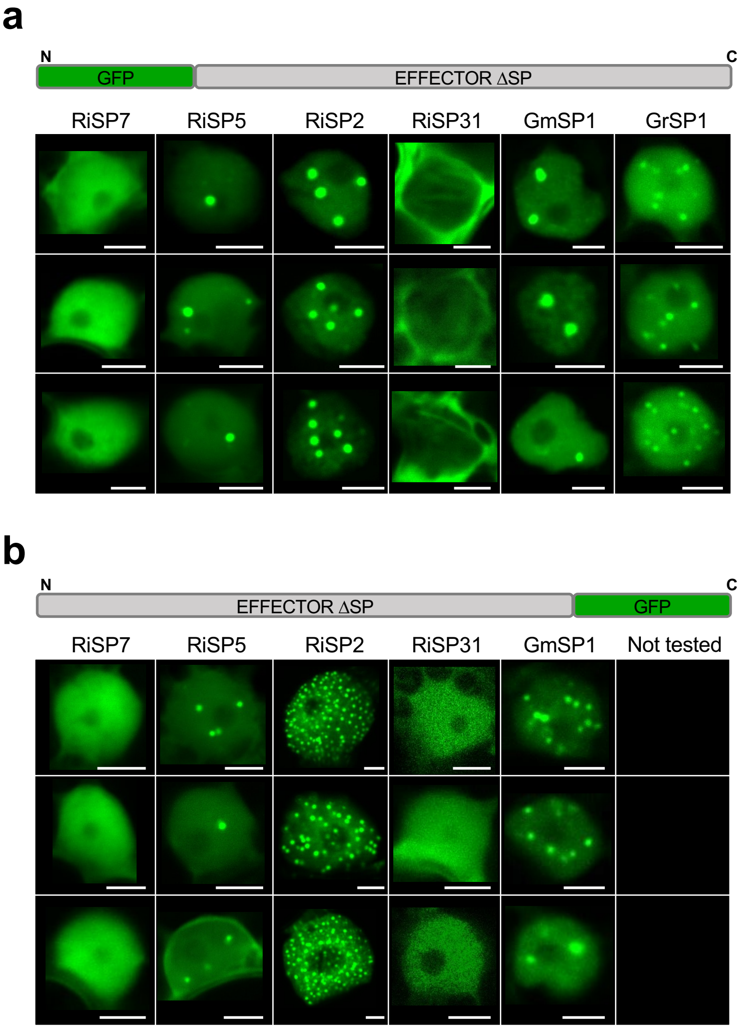

Supplementary Figure 6. Nuclear localization patterns of SP7-like effectors fused N- or C- terminally to eGFP in *Nicotiana benthamiana*.

a-b Shown are always three representative plant nuclei with the localization pattern of SP7-like effector proteins lacking their signal peptide (ΔSP) fused either N- (a) or C- terminally (b) to eGFP under control of the P35S promoter after transient expression in *N. benthamiana* epidermal cells. RiSP2, RiSP5, GmSP1 and GrSP1 localize to the plant nucleus and accumulate in nuclear bodies. Number and sizes of nuclear bodies differ, ranging from one to many bodies. RiSP7 N- and C- terminal GFP constructs lack the formation of nuclear bodies and show evenly distributed fluorescent signals throughout the nucleoplasm instead. GFP-RiSP31 is strongly located in the nuclear surroundings often with visible transversally stripes spanning the nucleus, while RiSP31-GFP can only be weakly detected in diffuse signals within nuclei as indicated by its grain-like signals. Scale bars represent 5 µm.

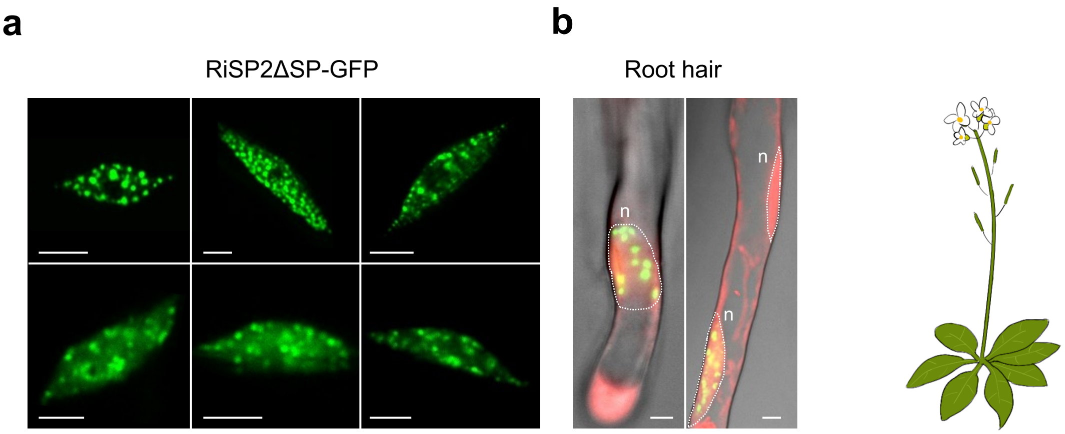

Supplementary Figure 7. RiSP2 localizes to nuclear bodies in *A. thaliana* roots and root hair.

a-b Shown are magnified pictures of nuclei from transgenic *A. thaliana* plants expressing RiSP2 fused to eGFP lacking its signal peptide (ΔSP) under control of the P35S promoter. **a** RiSP2 localizes at nuclear bodies in *A. thaliana* nuclei from root cells. **b** RiSP2 also localizes at nuclear bodies in root hair cells. Scale bars represent 5 μm.

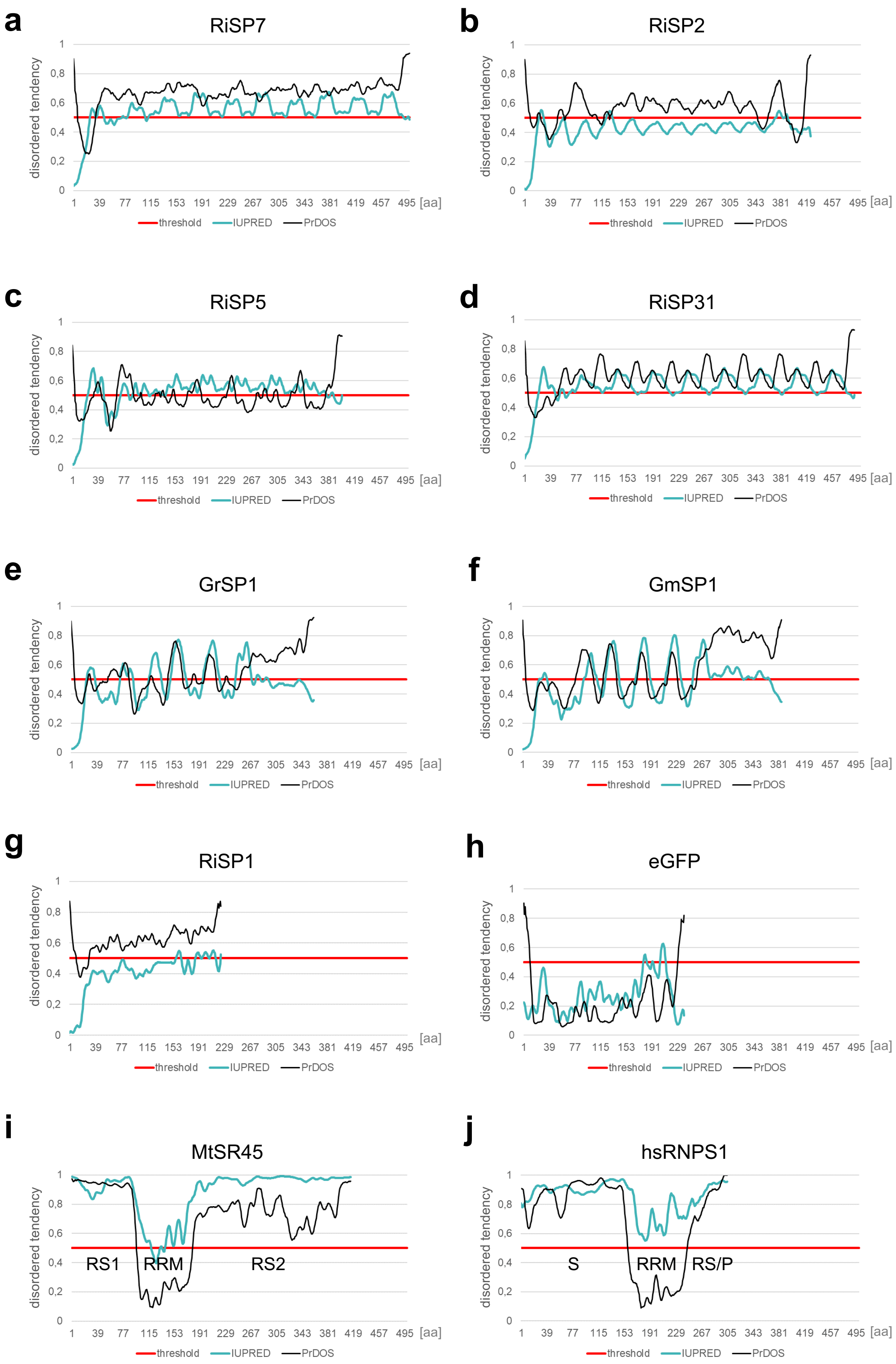

a
RiSP7
b
RiSP2
c
RiSP5
d
RiSP31
e
GrSP1
f
GmSP1
g
RiSP1
h
eGFP
i
MtSR45
RS1
RRM
RS2
j
hsRNPS1
S
RRM
RS/P
disordered tendency
0
0,2
0,4
0,6
0,8
1
1 39 77 115 153 191 229 267 305 343 381 419 457 495 [aa]
threshold
IUPRED
PrDOS

Supplementary Figure 8. Disorder probabilities of SP7-like effectors analyzed by PrDOS and IUPred prediction softwares.

a-g All SP7-like proteins showed an overall disordered tendency. **h** eGFP is, in contrast, structurally ordered. **i-j** MtSR45 and hsRNPS1 also show a disordered tendency in agreement with the predicted disordered RS domains of SR proteins[3], while the RRM domains are less disordered. Threshold for disorder probability was 0.5 marked with a red line. False positive rate (PrDOS) 5%.

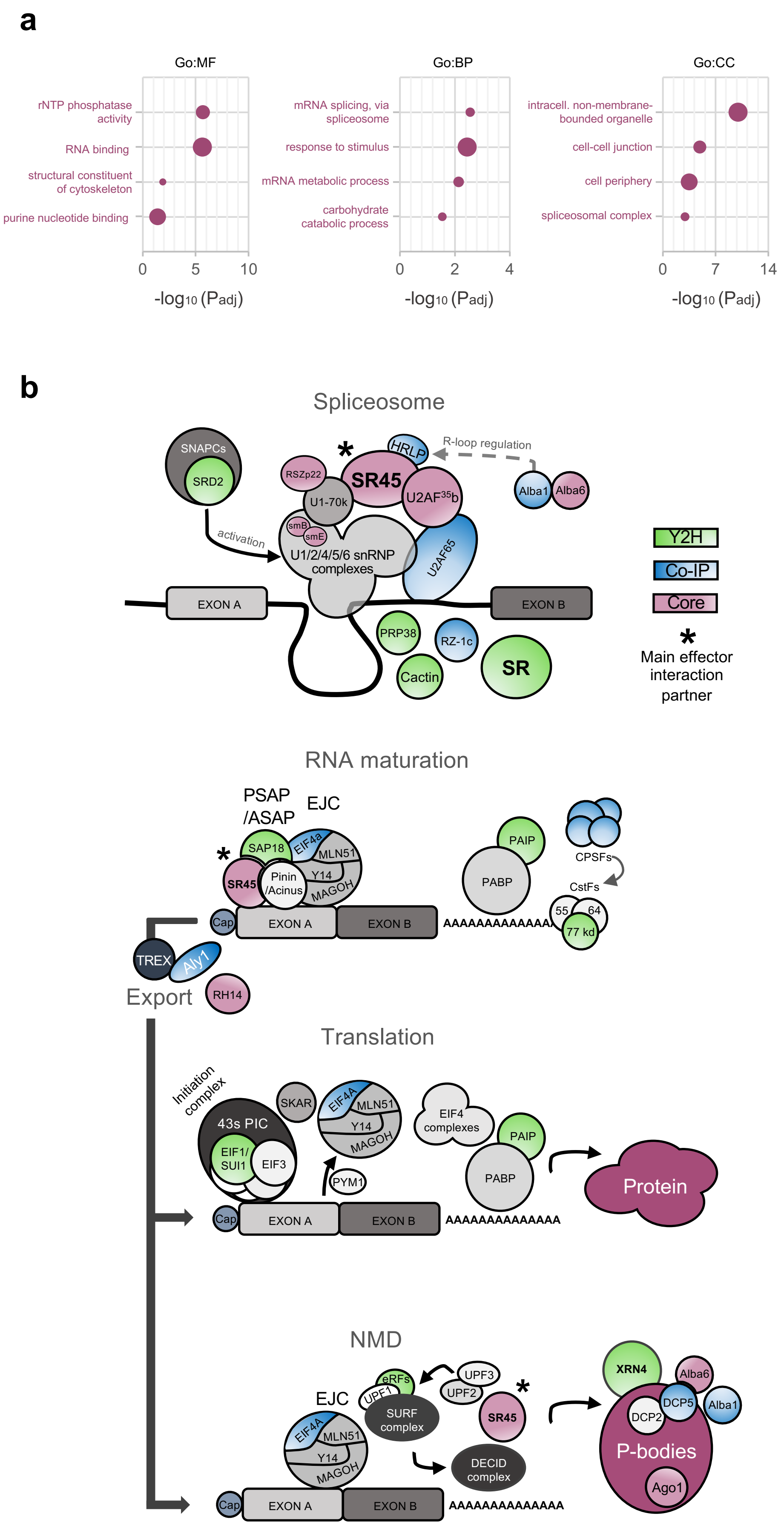
a
Go:MF
rNTP phosphatase activity
RNA binding
structural constituent of cytoskeleton
purine nucleotide binding
0 5 10
-log10 (Padj)
Go:BP
mRNA splicing, via spliceosome
response to stimulus
mRNA metabolic process
carbohydrate catabolic process
0 2 4
-log10 (Padj)
Go:CC
intracell. non-membrane-bounded organelle
cell-cell junction
cell periphery
spliceosomal complex
0 7 14
-log10 (Padj)
b
Spliceosome
SNAPCs
SRD2
activation
RSZp22
SR45
HRLP
R-loop regulation
Alba1
Alba6
U1-70k
U2AF35b
smB
smE
U1/2/4/5/6 snRNP complexes
U2AF65
EXON A
EXON B
PRP38
RZ-1c
Cactin
SR
Y2H
Co-IP
Core
Main effector interaction partner
RNA maturation
PSAP /ASAP
EJC
SAP18
EIF4a
MLN51
SR45
Pinin /Acinus
Y14
MAGOH
Cap
EXON A
EXON B
PAIP
PABP
CPSFs
CstFs
55
64
77 kd
AAAAAAAAAAAAA
TREX
Aly1
RH14
Export
Translation
Initiation complex
43s PIC
EIF1/ SUI1
EIF3
SKAR
EIF4A
MLN51
Y14
MAGOH
PYM1
EIF4 complexes
PAIP
PABP
Protein
Cap
EXON A
EXON B
AAAAAAAAAAAAAA
NMD
EJC
eRFs
UPF1
UPF3
UPF2
SURF complex
SR45
DECID complex
EIF4A
MLN51
Y14
MAGOH
XRN4
Alba6
DCP5
Alba1
DCP2
P-bodies
Ago1
Cap
EXON A
EXON B
AAAAAAAAAAAAAA

Supplementary Figure 9. The interactome of SP7-like effectors is enriched for plant proteins involved in RNA metabolism.

a-b By employing Y2H screens with SP7-like effectors as baits against *M. truncatula* and *A. thaliana* cDNA libraries as well as Co-IPs of RiSP7-GFP fusions (with and without SP) in *N. benthamiana*, a large set of putative plant interactors were identified. From those, 53 (core) effector target proteins either interacted with more than one effector or were repeatedly retrieved for RiSP7 in independent Co-IPs (detailed in Supplementary Table 3). **a** Go term enrichment analysis of the 53 core interactors using g:Profiler. Shown are the four most significant enriched functional gene groups in each category based on leading term filtering and p-value analysis (Supplementary Table 3). Significant enrichments of terms involved in RNA metabolism were found for each category. Circle sizes indicate number of interactors in each term. MF = Molecular function; BP = Biological process; CC = Cellular component. **b** Schematic illustration of a subset of identified interactors within the context of mRNA processing based on Arabidposis TAIR descriptions (Supplementary Table 3) and proposed functions[4,5]. With appearance in six different screens, SR45 (asterisk) was revealed as common effector interaction partner. Spliceosome: SnRNP complexes (activated by SNAPCs), SR proteins (including SR45/RNPS1) and other splicing factors assemble at splice sites. RNA maturation: After splicing, additional complexes are recruited at the exon junction including tetrameric EJC (exon junction complex), trimeric RNPS1/SR45 containing ASAP/PSAP complexes and polyadenylation factors (PABP, PAIP, CPSFs). After nuclear export (TREX, Aly1), mRNAs are either translated or send for nonsense-mediated decay (NMD). Translation: EJC and peripheral factors dissociate (PYM1). Translation initiation complexes (43s PIC, SKAR) and ribosome association enable protein synthesis. NMD: RNPS1/SR45 and different NMD complexes (SURF, DECID) activate degradation of aberrant mRNAs in P-bodies. Green coloured = Y2H identified interactors. Blue coloured = Co-IP identified interactors (RiSP7). Red coloured = Core Interactors.

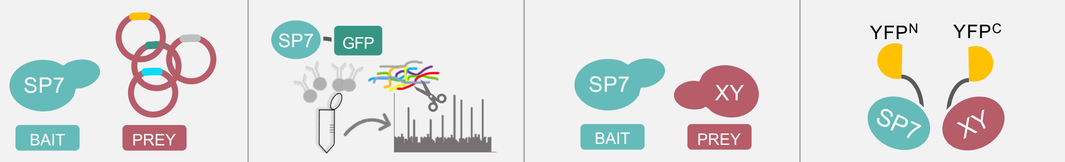

	Untargeted Approaches		Targeted Approaches	
	Y2H SCREEN	Co-IP SCREEN	Direct Y2H	BiFC
**SR45**	a,d,f,g	h,i	1,2,3,4,5,6	1,2,3,4,5,6
U2AF35b	a,c	/	1,2,3,4,5,6	1,2,3,4,5,6
U1-70K	/	/	1,2,3,4,5,6	1,2,3,4,5,6
SR45A	c	/	2,3,5,6	2,3,5,6
RsZp22	e	i	not tested	not tested
SC35	c	/	2,3,5,6	not tested
SCL30	c	/	3,5,6	not tested
TRA2	c	/	1,2,3,5,6	not tested
SAP18	b	/	2,3,5,6	not tested
Alba6	e	h,i	not tested	not tested
RH14	b	h,i	2,3,5,6	not tested
SUI1	g	/	1,2,3,4,5,6	not tested
Aly1	/	h,i	not tested	1,2,3

Screens:

[a] Y2H SP7 vs. cDNA library myc. *M. trunactula* roots
[b] Y2H SP5 vs. cDNA library myc. *M. trunactula* roots
[c] Y2H SP2 vs. cDNA library myc. *M. trunactula* roots
[d] Y2H SP31 vs. cDNA library myc. *M. trunactula* roots
[e] Y2H GmSP1 vs. cDNA library myc. *M. trunactula* roots
[f] Y2H SP7 vs. cDNA library *A. thaliana*
[g] Y2H SP5 vs. cDNA library *A. thaliana*
[h] Co-IP SP7ΔSP-GFP in N. benthamiana
[i] Co-IP SP7+SP-GFP in N. benthamiana

Bait Targeted approaches:

[1] RiSP7 [2] RiSP5 [3] RiSP2 [4] RiSP31 [5] GmSP1 [6] GrSP1

Supplemenary Figure 10. Overview of plant RNA processing proteins that were identified or tested in at least two different effector interaction assays. Untargeted screens = a-i; Targeted approaches: Numbers 1-6 indicated which effectors showed interaction in either Y2H or BiFC experiments. No values indicate not identified (untargeted approaches) or not tested (targeted approaches). SR45 was identified as interaction partner in most screens and for each SP7-like effector member.

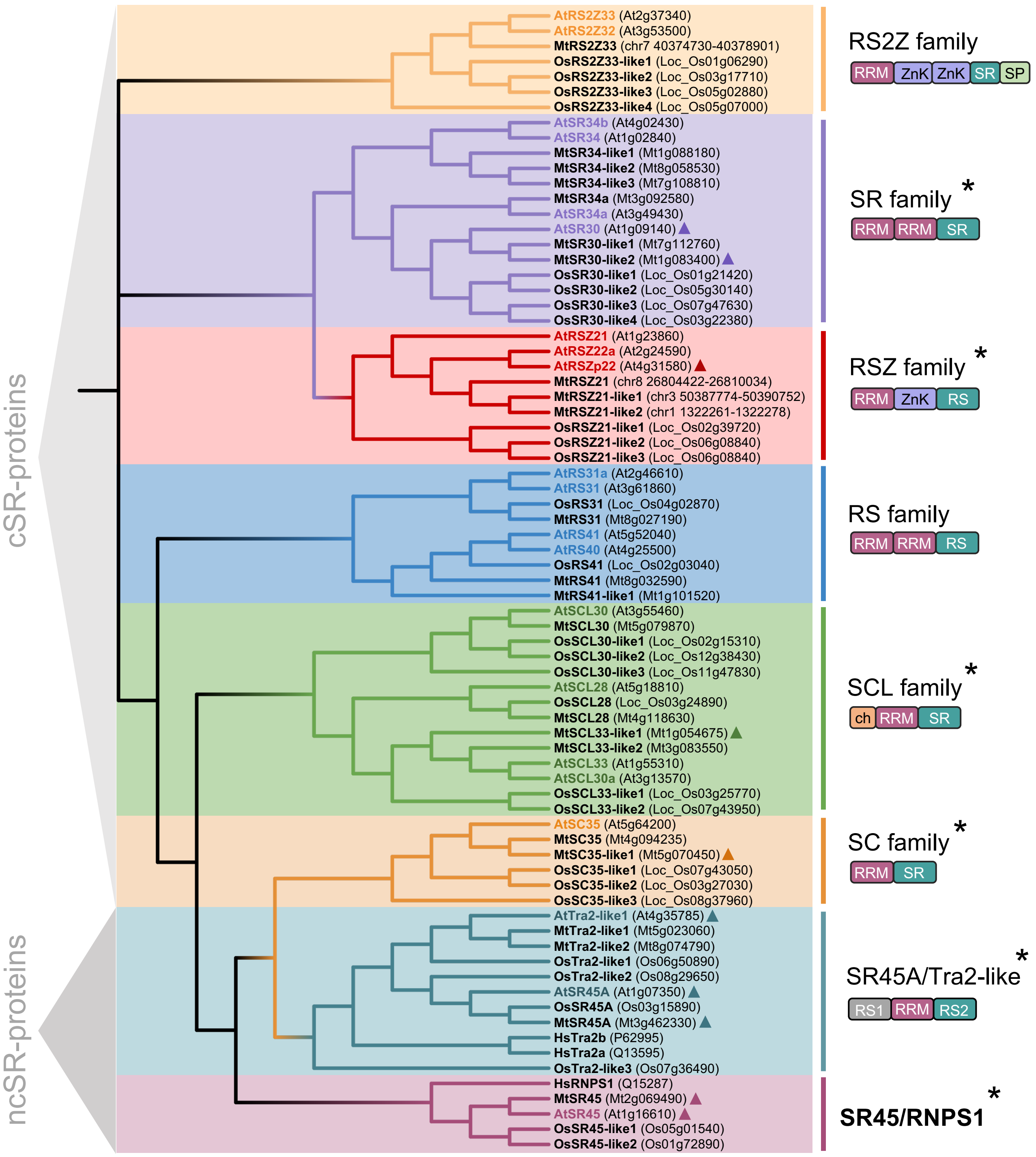

Supplemenary Figure 11. Phylogenetic relationship of *A. thaliana* (At), *M. truncatula* (Mt) and *Oryza sativa* (Os) SR protein members. Shown is a rooted Neighbor-Joining method tree (MEGA7, JTT modelling, Bootstrap 1000 replicates, evolutionary distances computed using the p-distance method) of SR protein family members after ClustalO alignment. Tree was visualized using iTOL. In accordance with identified members and nomenclature proposed previously[6], *A. thaliana* encodes 18 classical (cSR-proteins) and 3 non-classical (ncSR-proteins; containing two RS domains) SR proteins (colored IDs), while *O. sativa* contains 22 cSR-proteins. In addition, *O. sativa* also possess 6 ncSR members including two SR45 orthologs. Moreover, the human proteins RNPS1, Tra2a and Tra2b were used as representatives for metazoan orthologs of SR45 and SR45A. All members group into 8 subfamily clades with distinct domain architectures. Using BLAST searches with *A. thaliana* members as query in *M. truncatula* (JCVI MT4.0 genome assembly), we identified 19 *Medicago* cSR- and 4 ncSR-protein orthologs. SR proteins and families identified as interaction partners of SP7-like effectors (Fig. 3; Supplementary Fig. 10 and Supplementary Table 3) are marked by triangles and asterisks, respectively.

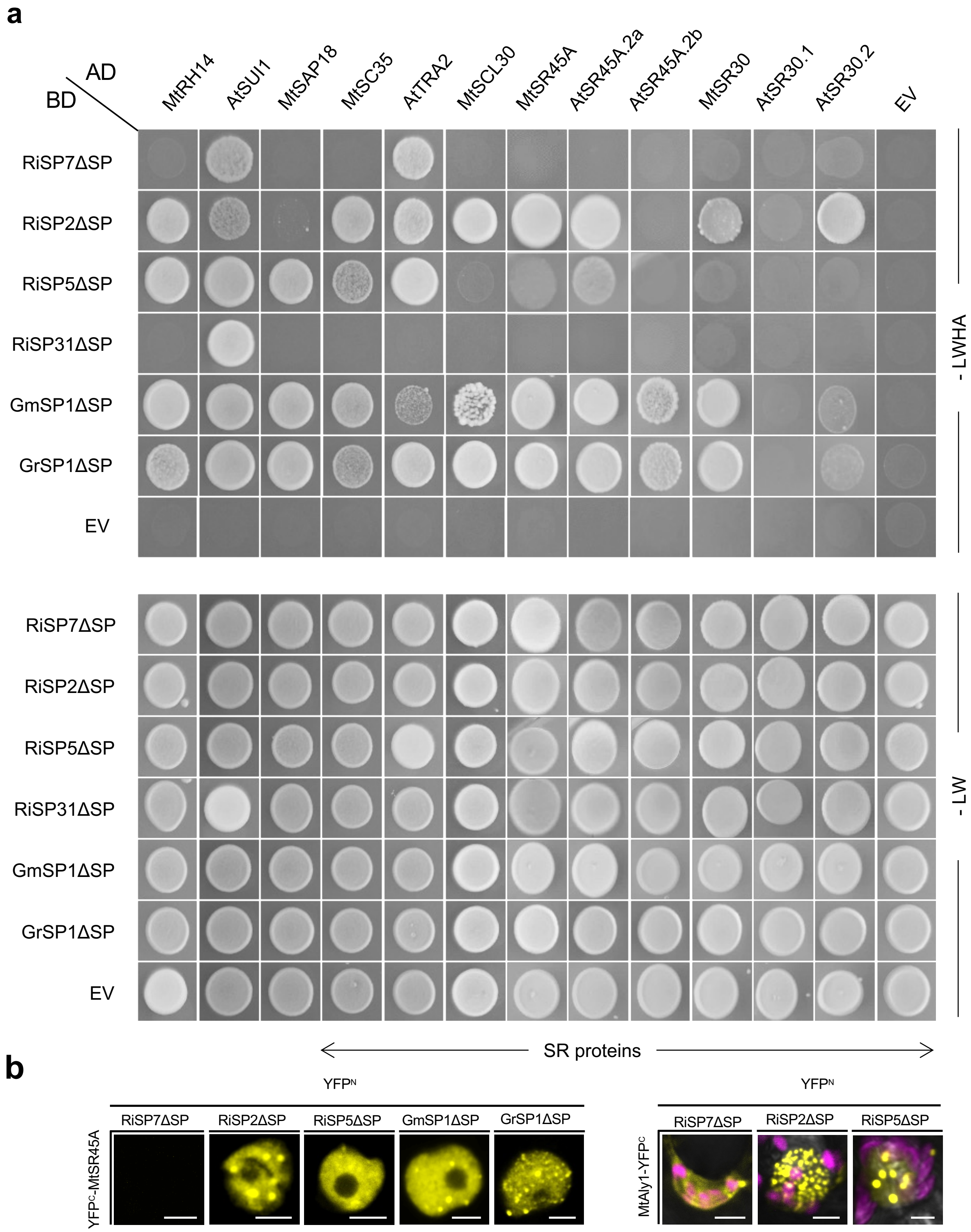
a
AD
BD
MtRH14
AtSUI1
MtSAP18
MtSC35
AtTRA2
MtSCL30
MtSR45A
AtSR45A.2a
AtSR45A.2b
MtSR30
AtSR30.1
AtSR30.2
EV
RiSP7ΔSP
RiSP2ΔSP
RiSP5ΔSP
RiSP31ΔSP
GmSP1ΔSP
GrSP1ΔSP
EV
- LWHA
- LW
SR proteins
b
YFP^N
RiSP7ΔSP
RiSP2ΔSP
RiSP5ΔSP
GmSP1ΔSP
GrSP1ΔSP
YFP^C-MtSR45A
MtAly1-YFP^C

c

d

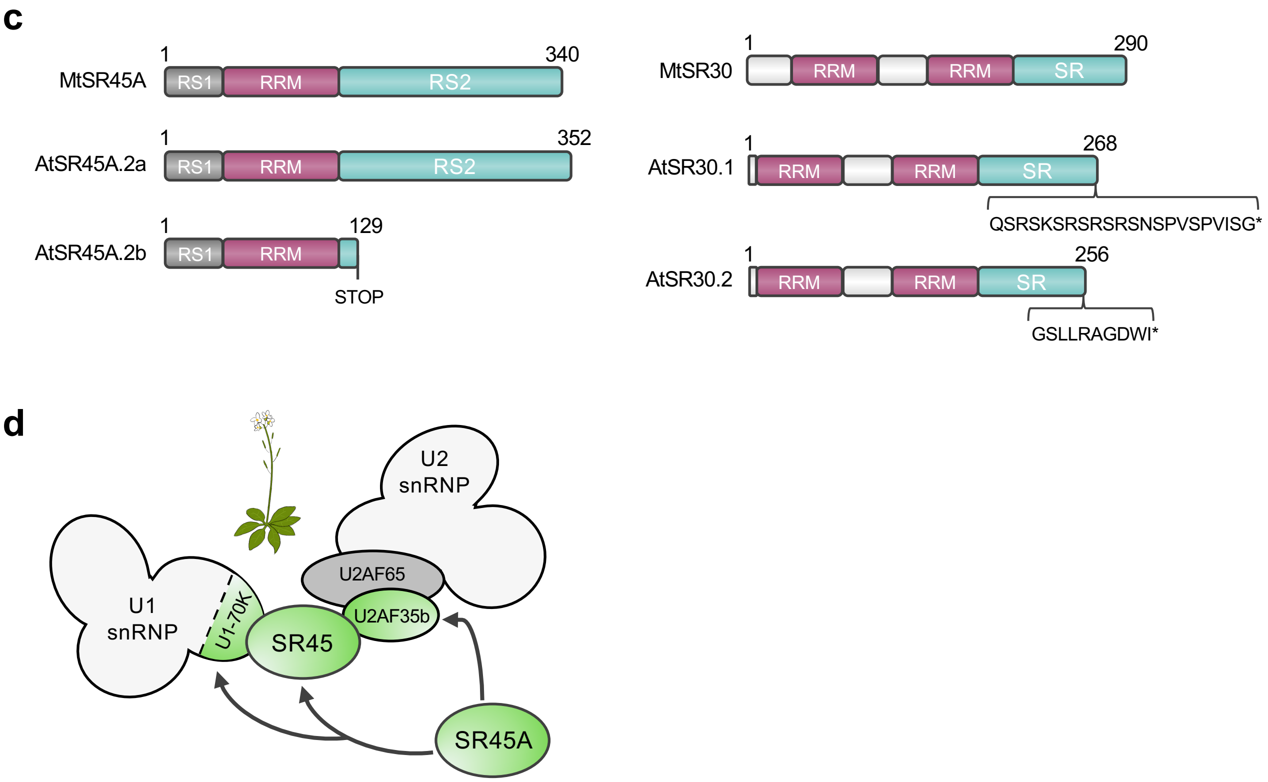

Supplementary Figure 12. SP7-like effectors show differences in interaction specificities with SR and mRNA processing proteins identified in interatomic screens

a Direct yeast two hybrid interaction assays between SP7-like effectors (as Bait fused to the Gal4-BD) and multiple plant SR proteins and mRNA processing proteins (as Prey fused to the Gal4-AD) identified in different screens. In addition, MtSR30 and its *Arabidopsis* ortholog AtSR30 (isoforms AtSR30.1 and AtSR30.2) were tested. While RiSP2 and the *Gigaspora* effectors interacted with most of the plant interaction partners, RiSP7, RiSP5 and RiSP31 showed less promiscuity. MtRH14 (Medtr6g005550), AtSUI1 (AT5G54760), MtSAP18 (Medtr8g033340). SR proteins: AtTRA2 (AT4G35785), MtSCL30 (Medtr1g054675), MtSC35 (Medtr5g070450), MtSR45A (Medtr3g462330), isoforms (AtSR45A.2 and AtSR45A.2b) of AtSR45A (At1g07350), MtSR30 (Medtr1g083400), isoforms (AtSR30.1 and AtSR30.2) of AtSR30 (At1g09140). Positive interactions are indicated by yeast colony growth on media lacking leucine, tryptophan, histidine and adenine (-LWHA). BD = Binding domain, AD = Activation Domain, EV = Empty vector control. **b** Bimolecular fluorescence complementation assays in *N. benthamiana* leaf epidermal cells of SP7-like effectors and MtSR45A (left panel) or MtAly1 (Medtr4g063557, right panel). Shown are magnified pictures of single plant nuclei. As observed in (a) interactions of RiSP2, RiSP5, GmSP1 and GrSP1 (fused to N-terminal half of YFP, YFPN) with MtSR45A (fused to C-terminal half of YFP, YFPC) can be detected at the nucleus and nuclear bodies. For RiSP7, no YFP signals could be found within the transformed leaf tissue. Similarly, YFP signals were present in nuclei and nuclear bodies for RiSP7, RiSP2 and RiSP5 when co-expressed with MtAly1, initially identified in Co-IPs with RiSP7. Chloroplast autofluorescence is pseudocoloured in magenta (merged pictures). All constructs under control of the 35S promoter. Scale bar = 5 µm. **c** Protein structures of MtSR45A, AtSR45A, MtSR30, AtSR30 and existing isoforms. The AtSR45A and AtSR30 pre-mRNAs are alternatively spliced into several isoforms[7,8,9]. Isoforms AtSR45A.2, AtSR45A.2b as well as AtSR30.1 and AtSR30.2 were chosen for interaction studies in this work. **d** Model for published interaction networks[10] of *Arabidopsis* SR45A, SR45, U1-70k and U2AF35b during spliceosome complex formation.

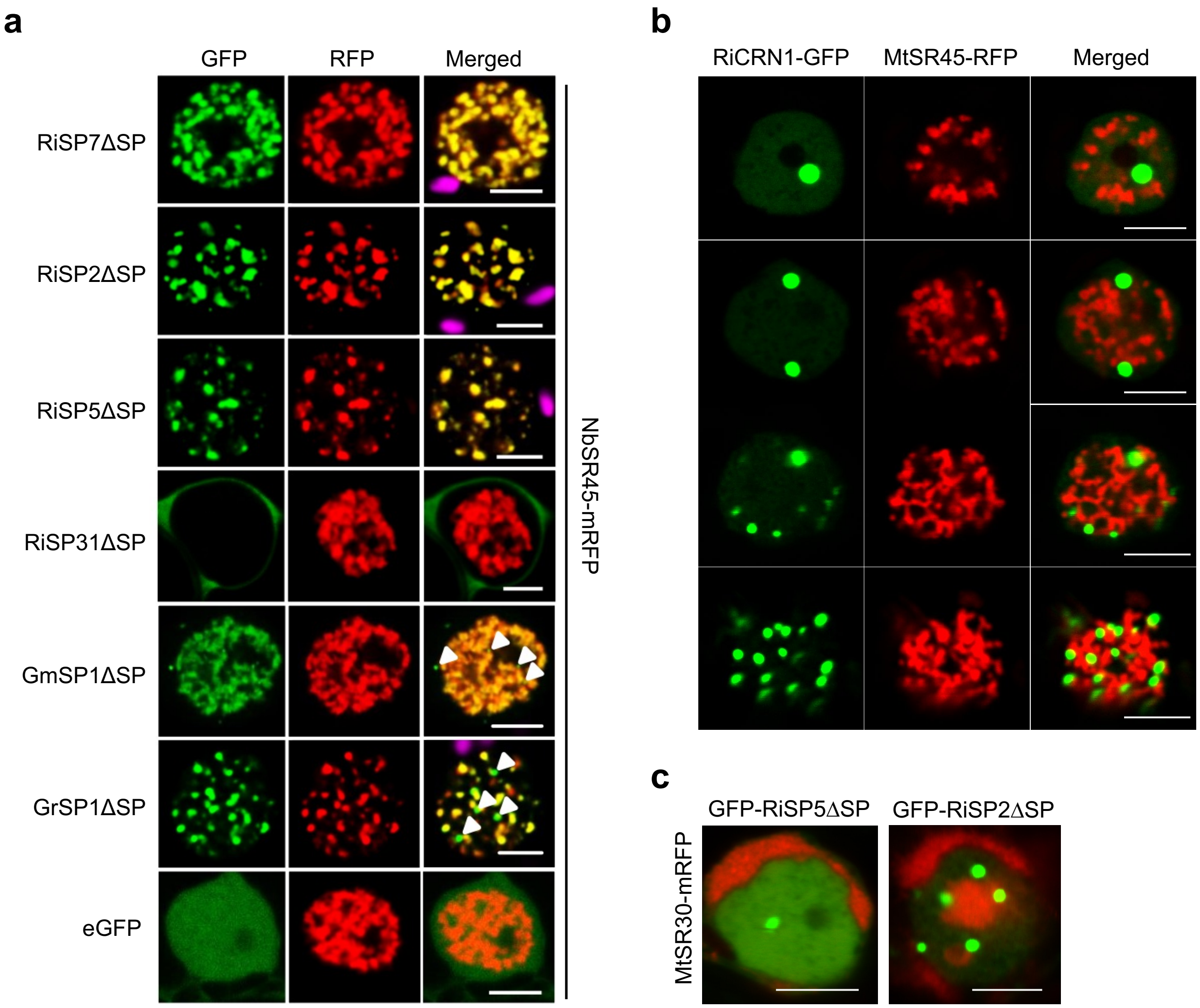

Supplementary Figure 13. Co-localization of NbSR45 or additional nuclear body Co-localization controls and SP7-like effectors.

a-c All experiments were carried out with SP7-like effectors lacking their signal peptides (ΔSP). Shown are localization patterns in single plant nuclei after transient expression of fluorescent tagged proteins under control of the P35S promoter in *N. benthamiana* epidermal cells. Scale bars represent 5 µm. **a** Co-localization of SP7-like effectors N-terminally fused to eGFP and NbSR45 fused C-terminally to mRFP. In contrast to the eGFP control, all tested SP7-like effectors re-localized to nuclear condensates occupied by NbSR45. For *Gigaspora* effectors, exclusive nuclear bodies were observed in GFP channels (white arrowheads). **b** Co-localization of MtSR45-mRFP together with the C-terminal part of the mycorrhizal effector RiCRN1 fused to GFP[11]. RiCRN1 localizes in nuclear bodies in different shapes and sizes, but it does not co-localize with MtSR45. **c** Co-localization of GFP-RiSP2 and GFP-RiSP5 with MtSR30 fused to mRFP (merged pictures). Both RiSP7-like effectors show the same localization pattern observed for single localizations in *N. benthaminana* (see Fig. 2 and Supplementary Fig. 6) No re-localization of RiSP2 and RiSP5 to MtSR30 localizations were observed.

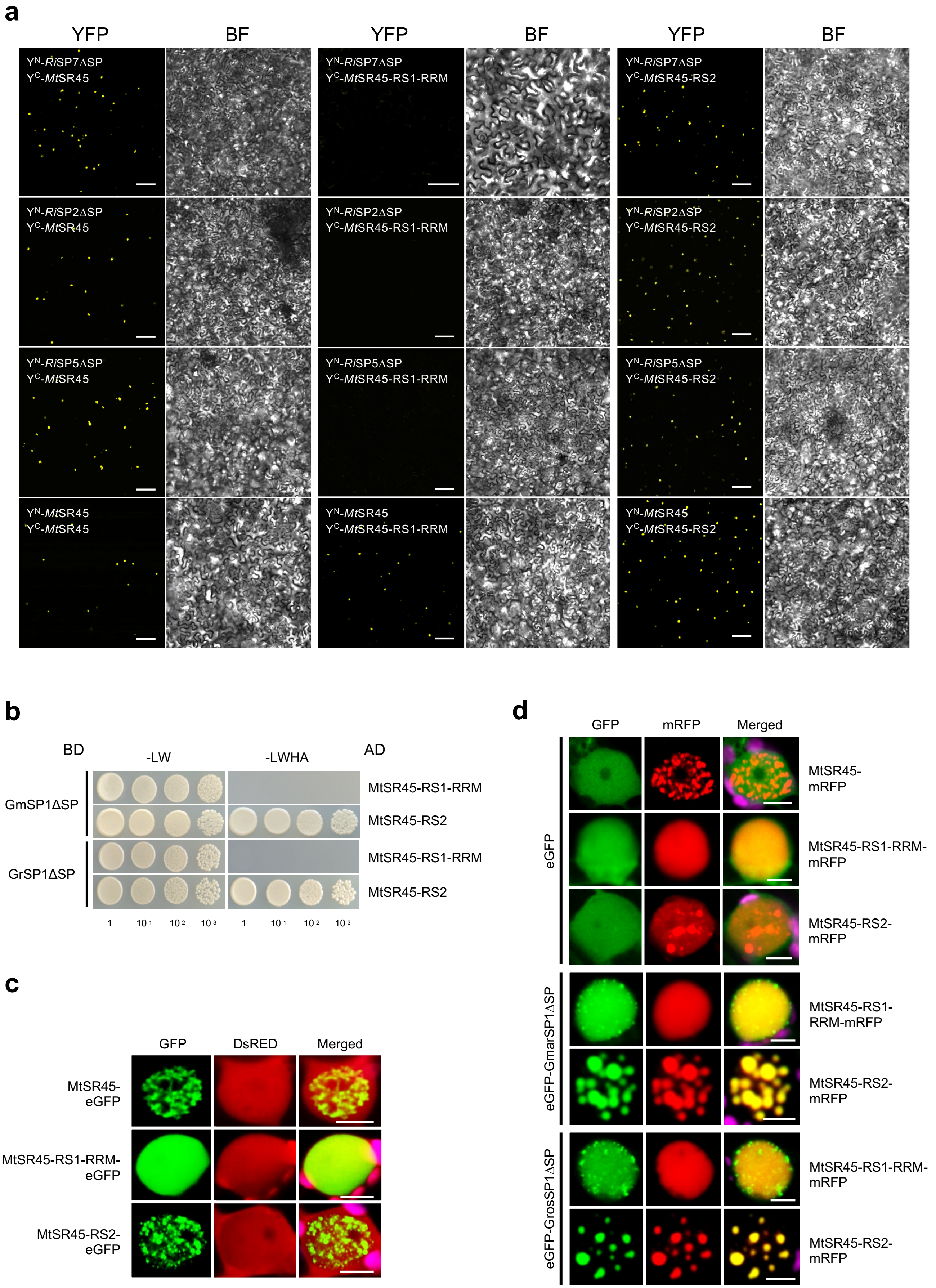
a
YFP
BF
YN-RiSP7ΔSP
YC-MtSR45
YN-RiSP7ΔSP
YC-MtSR45-RS1-RRM
YN-RiSP7ΔSP
YC-MtSR45-RS2
YN-RiSP2ΔSP
YC-MtSR45
YN-RiSP2ΔSP
YC-MtSR45-RS1-RRM
YN-RiSP2ΔSP
YC-MtSR45-RS2
YN-RiSP5ΔSP
YC-MtSR45
YN-RiSP5ΔSP
YC-MtSR45-RS1-RRM
YN-RiSP5ΔSP
YC-MtSR45-RS2
YN-MtSR45
YC-MtSR45
YN-MtSR45
YC-MtSR45-RS1-RRM
YN-MtSR45
YC-MtSR45-RS2
b
BD
-LW
-LWHA
AD
GmSP1ΔSP
GrSP1ΔSP
MtSR45-RS1-RRM
MtSR45-RS2
1
10-1
10-2
10-3
c
GFP
DsRED
Merged
MtSR45-eGFP
MtSR45-RS1-RRM-eGFP
MtSR45-RS2-eGFP
d
GFP
mRFP
Merged
eGFP
eGFP-GmarSP1ΔSP
eGFP-GrosSP1ΔSP
MtSR45-mRFP
MtSR45-RS1-RRM-mRFP
MtSR45-RS2-mRFP

Supplementary Figure 14. Additional interaction and localization assays of MtSR45 truncations and SP7-like effectors.

a Overview pictures for BiFC assays between RiSP7ΔSP, RiSP2ΔSP or RiSP5ΔSP (fused to N-terminal half of YFP, YFP^{N}). and MtSR45 or the truncation domains RS1-RRM (MtSR45-RS1-RRM) and RS2 (MtSR45-RS2) fused to C-terminal half of YFP, YFP^{C}. Shown are pictures of infiltrated *N. benthamiana* whole cell tissue areas. All consructs under control of the P35S promoter. Reconstituted YFP signals (left panels) in nuclei of several cells demonstrated interactions between SP7-like effectors and full length MtSR45 as well as the RS2 domain alone. No YFP signals were detected for RS1-RRM truncations. In addition, interaction of MtSR45 with itself as well as with all MtSR45 truncations was observed. Right panels: Brightfield pictures (BF) of respective cell areas seen in YFP channels. Scale bars represent 100 µm. **b** The RS2 domain of MtSR45 is sufficient and required for interaction with *Gigaspora* SP7-like effectors. Shown are Y2H interaction spotting assays (using yeast dilution series $1\text{-}10^{-3}$) between GmSP1ΔSP or GrSP1ΔSP as bait (BD) and the RS1-RRM or RS2 truncations as prey (AD). Positive interactions are indicated by colony growth on media lacking leucine, tryptophan, histidine, adenine (-LWHA). EV = Empty vector. **c-d** single plant nuclei are shown after transient expression of fluorescent tagged proteins in *N. benthamiana* epidermal cells. Chloroplast autofluorescences are pseudo-coloured in magenta (merged pictures). All constructs under control of the P35S promoter. Scale bars represent 5 µm. **c** Subcellular localization of MtSR45 and truncations (with C-terminal eGFP tag). Only full length MtSR45 and the RS2 domain localized to nuclear speckles. As control, free DsRed was co-expressed. **d** Co-localization assay of GmSP1ΔSP and GrSP1ΔSP (N-terminal eGFP tag) with MtSR45, RS1-RRM or RS2 truncation domains (C-terminal mRFP tag). Both *Gigaspora* effectors only co-localized with full length MtSR45 and the RS2 domain in nuclear bodies. All experiments were performed with SP7-like effectors lacking their signal peptides (ΔSP).

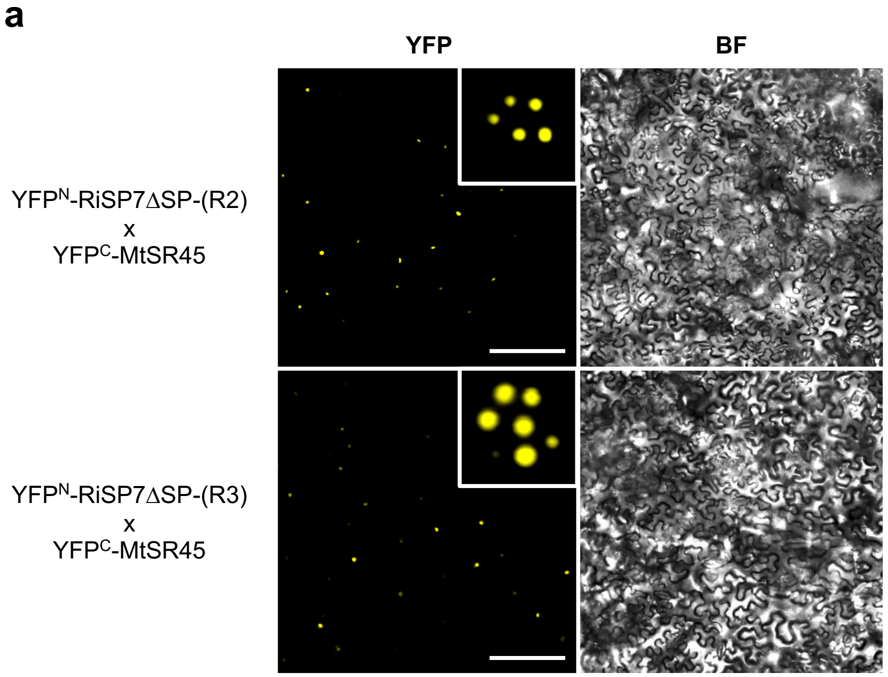

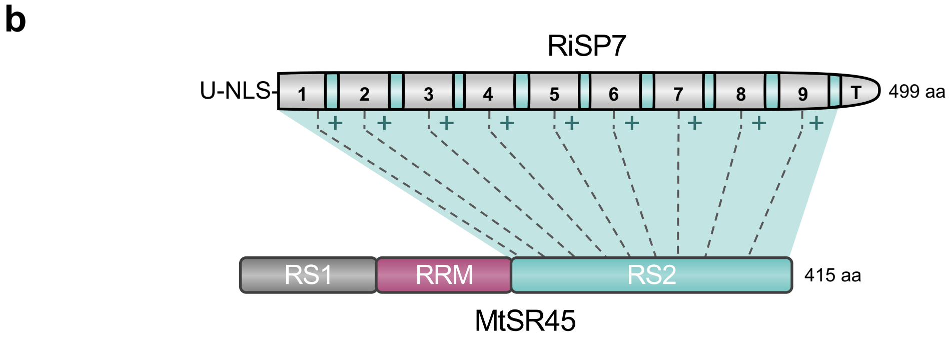

Supplementary Figure 15. Number of RiSP7 repeats determines its interaction strength with MtSR45.

a Overview pictures of BiFC assays using RiSP7ΔSP truncations (without SP, fused to N-terminal half of YFP, YFP^{N}) together with MtSR45 (fused to C-terminal half of YFP, YFP^{C}) under control of the P35S promoter after transient expression in *N. benthamiana* epidermal cells. Shown are pictures of infiltrated *N. benthamiana* whole cell tissue areas. Reconstituted YFP signals in nuclear condensates could be observed for truncations with two and three repeat units in several cell nuclei (inlet pictures show exemplary magnified single nuclei with nuclear bodies). BF = Brightfield pictures of respective cell areas seen in YFP channels. Scale bars represent 200 µm. **b** Model of proposed mode of interaction between RiSP7 and the RS2 domain of SR45. Each single repeat unit enhances the affinity and interaction strength (+) with the RS2 domain.

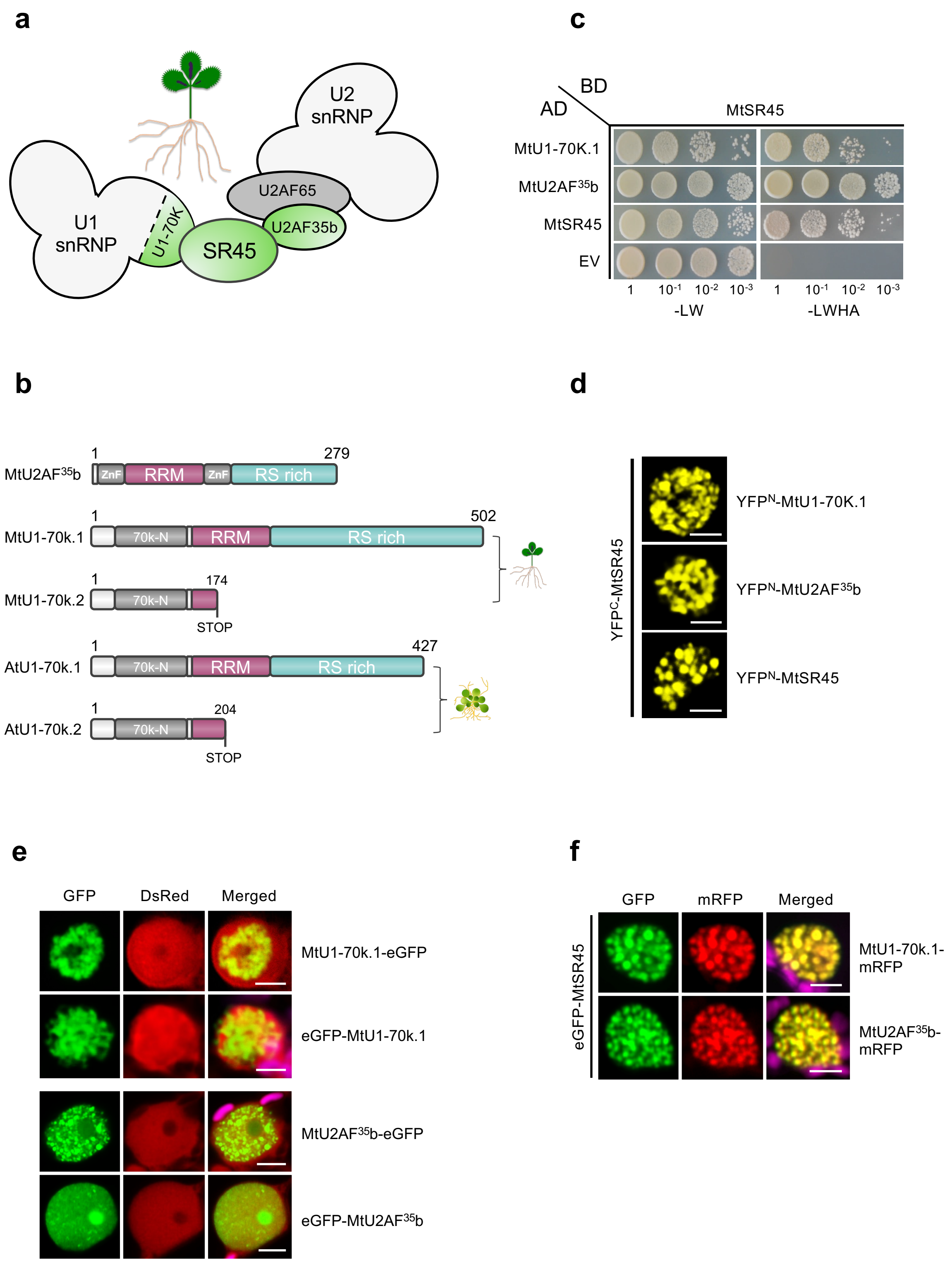

a
U2
snRNP
U2AF65
U1
snRNP
U1-70K
SR45
U2AF35b
c
BD
AD
MtSR45
MtU1-70K.1
MtU2AF35b
MtSR45
EV
1
10-1
10-2
10-3
-LW
-LWHA
b
MtU2AF35b
ZnF
RRM
RS rich
1
279
MtU1-70k.1
70k-N
502
MtU1-70k.2
174
STOP
AtU1-70k.1
427
AtU1-70k.2
204
d
YFPC-MtSR45
YFPN-MtU1-70K.1
YFPN-MtU2AF35b
YFPN-MtSR45
e
GFP
DsRed
Merged
MtU1-70k.1-eGFP
eGFP-MtU1-70k.1
MtU2AF35b-eGFP
eGFP-MtU2AF35b
f
GFP
mRFP
Merged
eGFP-MtSR45
MtU1-70k.1-mRFP
MtU2AF35b-mRFP

Supplementary Figure 16. SR45 interactions with spliceosomal components U2AF^{35}b and U1-70k are conserved in *Medicago*.

a Model for possible interaction network of *Medicago* SR45 with MtU2AF^{35}b and MtU1-70k based on published interactions of *Arabidopsis* SR45 (see Fig. 6). **b** Protein structures of MtU2AF^{35}b and MtU1-70k. MtU2AF^{35}b comprises a RRM motif flanked by two CCCH-type zinc fingers (ZnFs) and a RS-rich domain similar to its *Arabidopsis* orthologue[12]. MtU1-70K exists as two isoforms (MtU1-70k.1 and MtU1-70k.2) also described for the AtU1-70k ortholog[13] with a conserved N-terminal domain (70K-N). Isoform MtU1-70k.2 lacks RRM and RS-rich domains. **c** Y2H assays revealed the interaction between MtSR45 (Bait, BD) with *Medicago* U2AF^{35}b and U1-70k.1 (Preys, AD). MtSR45 was also tested for homo-dimerization as previously reported for plant SR proteins[10,14]. Positive interactions are indicated by yeast colony growth on media lacking leucine, tryptophan, histidine, adenine (-LWHA) using dilution series (1-10^{-3}). EV = Empty vector. **d-f** Shown are localization patterns in single plant nuclei after transient expression of fluorescent tagged proteins under control of the P35S promoter in *N. benthamiana* epidermal cells. Scale bars represent 5 µm. **e-f** Chloroplast autofluorescences are pseudo-coloured in magenta (merged pictures). **d** Confirmation of interactions seen in (c) using BiFC. Interaction was detected in nuclear speckles for MtSR45 (fused to C-terminal half of YFP, YFP^{C}) co-expressed with either MtU2AF^{35}b, MtU1-70k.1 or with itself (fused to N-terminal half of YFP, YFP^{N}). **e** Subcelluar localization of MtU1-70k.1 or MtU2AF^{35}b (N- or C-terminal eGFP fusions) showed localizations in differently shaped nuclear speckles. Additionally, eGFP-MtU2AF^{35}b localized in the nucleolus. As control, free DsRed was co-expressed. **f** Co-localization of eGFP-MtSR45 with MtU1-70k.1 or MtU2AF^{35}b fused C-terminally to mRFP. Both proteins co-localized with MtSR45 in nuclear speckles.

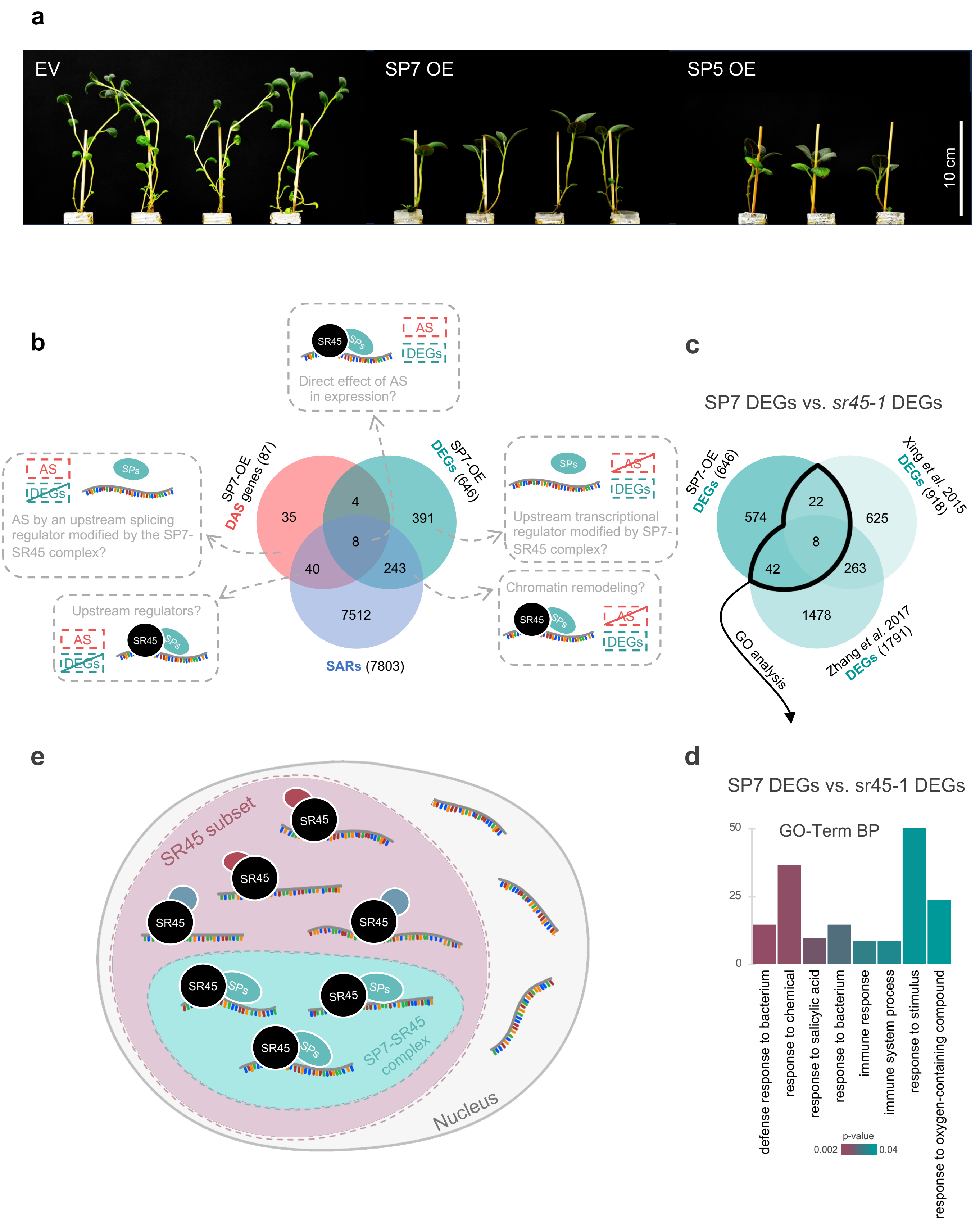
a
EV
SP7 OE
SP5 OE
10 cm
b
SR45
SPs
AS
DEGs
Direct effect of AS in expression?
AS by an upstream splicing regulator modified by the SP7-SR45 complex?
SP7-OE DAS genes (87)
SP7-OE DEGs (646)
35
4
391
8
40
243
7512
SARs (7803)
Upstream transcriptional regulator modified by SP7-SR45 complex?
Upstream regulators?
Chromatin remodeling?
c
SP7 DEGs vs. sr45-1 DEGs
SP7-OE DEGs (646)
Xing et al. 2015 DEGs (918)
Zhang et al. 2017 DEGs (1791)
574
22
625
8
42
263
1478
GO analysis
d
SP7 DEGs vs. sr45-1 DEGs
GO-Term BP
50
25
0
defense response to bacterium
response to chemical
response to salicylic acid
response to bacterium
immune response
immune system process
response to stimulus
response to oxygen-containing compound
p-value
0.002
0.04
e
SR45 subset
SR45
SPs
SP7-SR45 complex
Nucleus

Supplementary Figure 17. Ectopic expression of *RiSP7* and *RiSP5* in *Solanum tuberosum* modifies plant development and impacts on SR45 associated gene regulatory pathways.

a Representative pictures of transgenic potato plants that are either transformed with an empty vector (EV), with a vector for RiSP7-expression (SP7-OE) or a vector for RiSP5-expression (SP5 OE). All constructs under control of the 2XP35S promoter. Effector expression leads to a stunted growth phenotype compared to EV control plants. **b** Venn diagram showing the overlap between DEGs (green) as well as DAS genes (red) in SP7-OE plants (*Arabidopsis* orthologs of SP7-OE potato) compared to known *Arabidopsis* SR45 RNA targets (SARs, blue) identified by RNA immunoprecipitation[15,16]. Possible effector-mediated regulatory mechanisms explaining the different subgroups are depicted in boxes. **c** Venn diagram showing the overlap of DEGs between SP7-OE (*Arabidopsis* orthologs of SP7-OE potato DEGs) and *sr54-1 Arabidopsis* mutant plants[15,16]. A total of 72 SP7 DEGs are present in at least one SAR dataset. **d** GO-Term enrichment analysis (ThaleMine v5.1.0) of the common DEGs identified in (c), reveals enrichment for genes involved in defense and immunity reactions. Most significant enrichment Term: Defense response to bacterium. **e** Model for the envisioned SR45-effector mRNA target selection. Within the total pool of SARs (SR45 subset, pink area), binding to and modification of specific mRNA targets depend on the precise combination of SR45 co-interactors. Thus, the SR45-SP7 effector complex binds to a specific effector dependent target subset (green area).

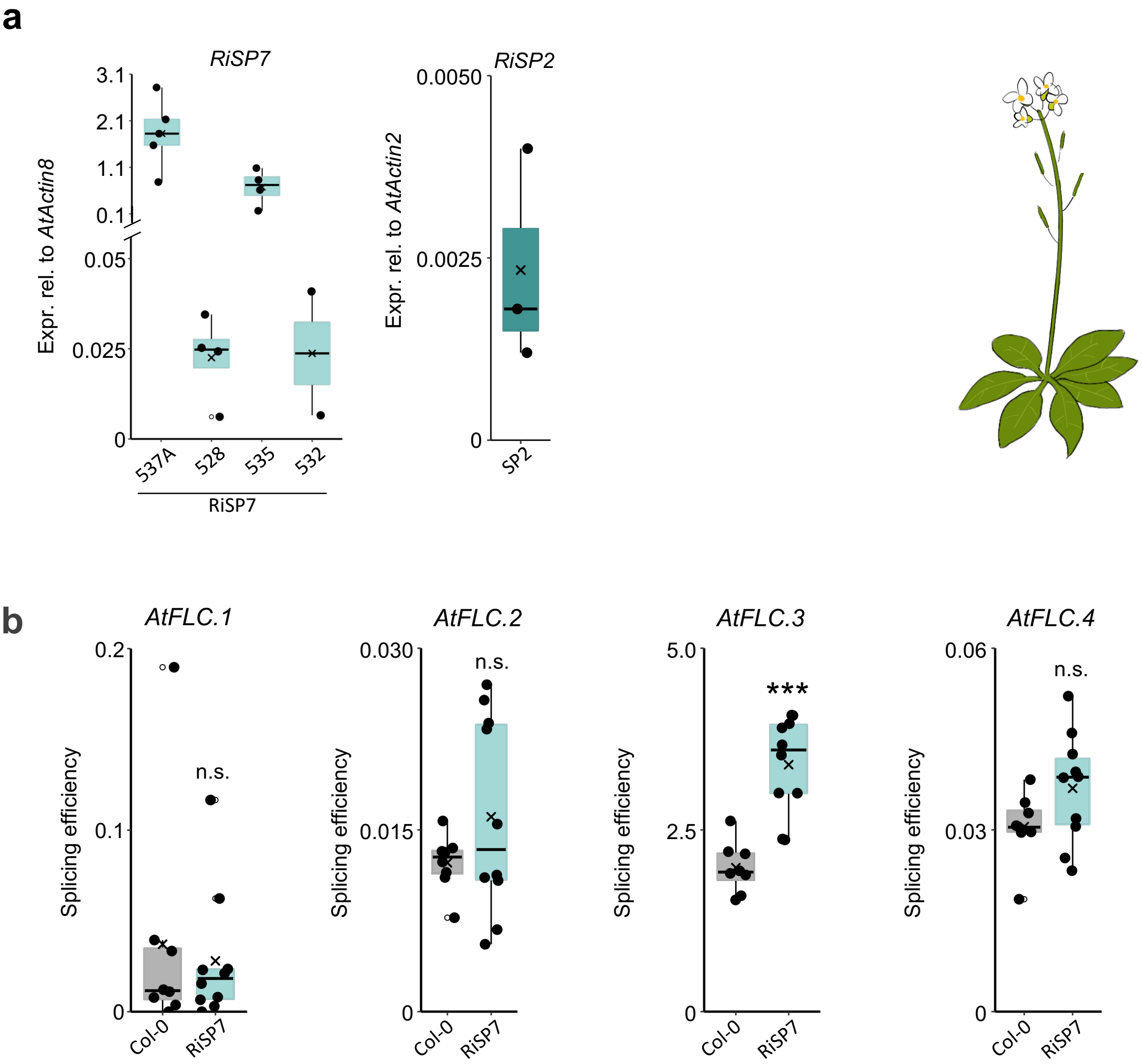

Supplementary figure 18. Transgenic *A. thaliana* lines overexpress mycorrhizal SP7-like effectors and this leads to an alteration of the splicing efficiency of the *AtFLC* splicing isoform 3.

a Quantification using qRT-PCR of the expression levels of *RiSP7* and *RiSP2* of *A. thaliana* transgenic lines relative to *AtActin8* and *AtActin2,* respectively. **b** Splicing efficiency in WT (Col-0) and *RiSP7*-expressing *Arabidopsis* plants of the different *AtFLC* splicing isoforms expressed as the ratio of the relative expression levels of the spliced version and the unspliced version of each isoform. The *AtFLC* isoform 3 is significantly more efficiently spliced in the *RiSP7*-expressing plants compared to the Col-0 plants.
Sample sizes: (a) n = 5 (RiSP7 line 537A), n = 4 (RiSP7 lines 528 and 535), n = 2 (RiSP7 line 532) and n = 3 (RiSP2); (b) n = 8 (Col-0) and n = 10 (RiSP7 lines: 528, 532 and 535). In all boxplots, the boxes show the quartiles, the whiskers mark the maximum and minimum values, except outliers that are shown as extra dots. Statistical significance (b) was calculated either with a T-test or a Mann-Whitney U test as explained in Materials and Methods. Significance levels: n.s. (non-significant) p>0.05, * p<0.05, ** p<0.01, *** p<0.001. Exact p-values are shown in Source Data File.

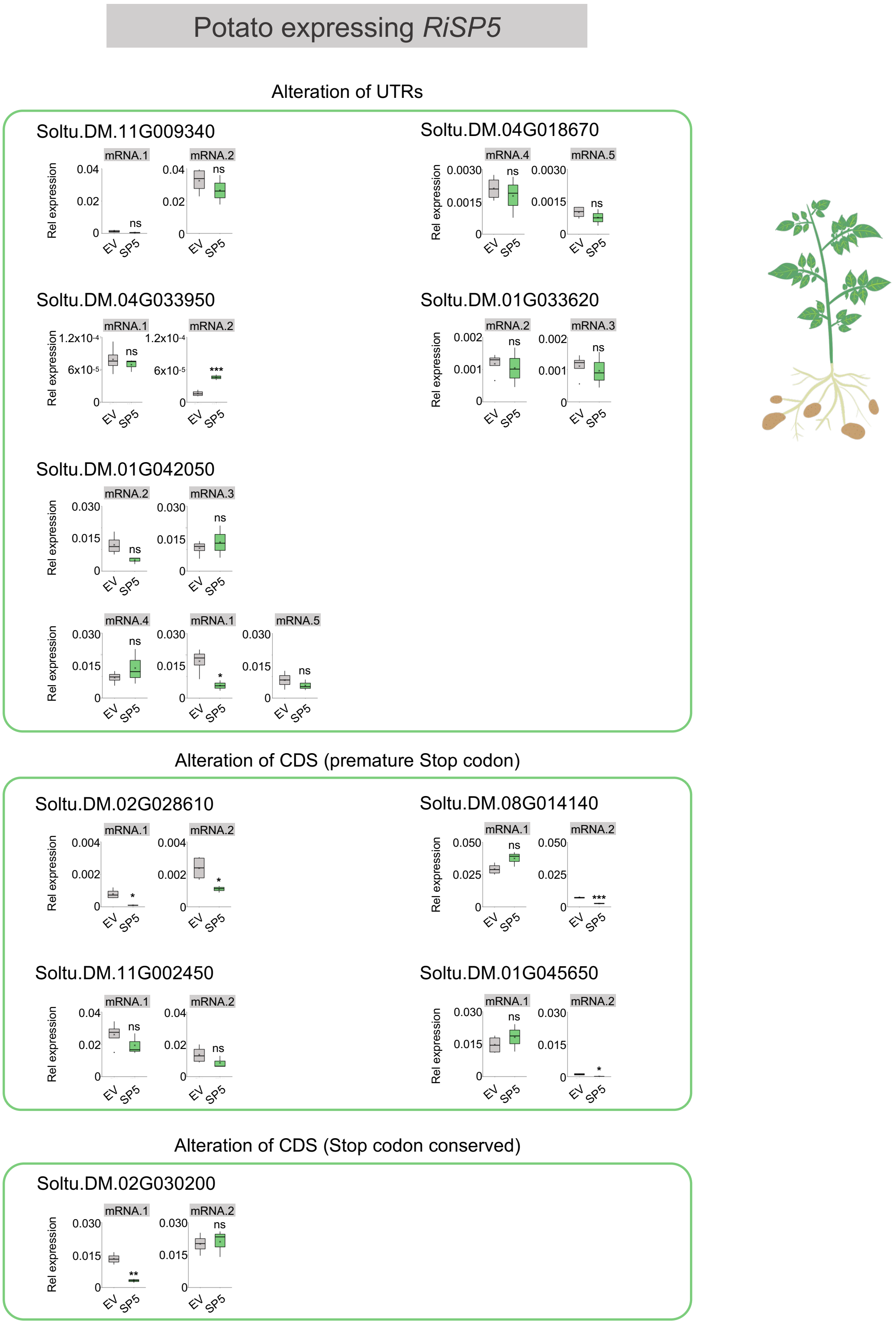

Supplementary Figure 19. qRT-PCR analysis of the splicing in response to ectopic expression of RiSP5 in potato.

qRT-PCR quantification of the relative expression of each splicing isoform of the selected DAS potato genes in roots of control plants (EV) and plants ectopically expressing *RiSP5* under control of the 2XP35S promoter. In all boxplots, the boxes show the quartiles, the whiskers mark the maximum and minimum values, except outliers that are shown as extra dots.
Statistical significance was assessed by performing a Student T test or a Mann Whitney U test after checking the normal distribution of the data and homocedasticity as explained in Materials and Methods. Sample size was n = 4 for EV (from two independent lines) and n = 3 for SP5 (from one line). Significance levels: n.s. (non-significant) p>0.05, * p<0.05, ** p<0.01, *** p<0.001. Exact p-values are shown in Source Data File.

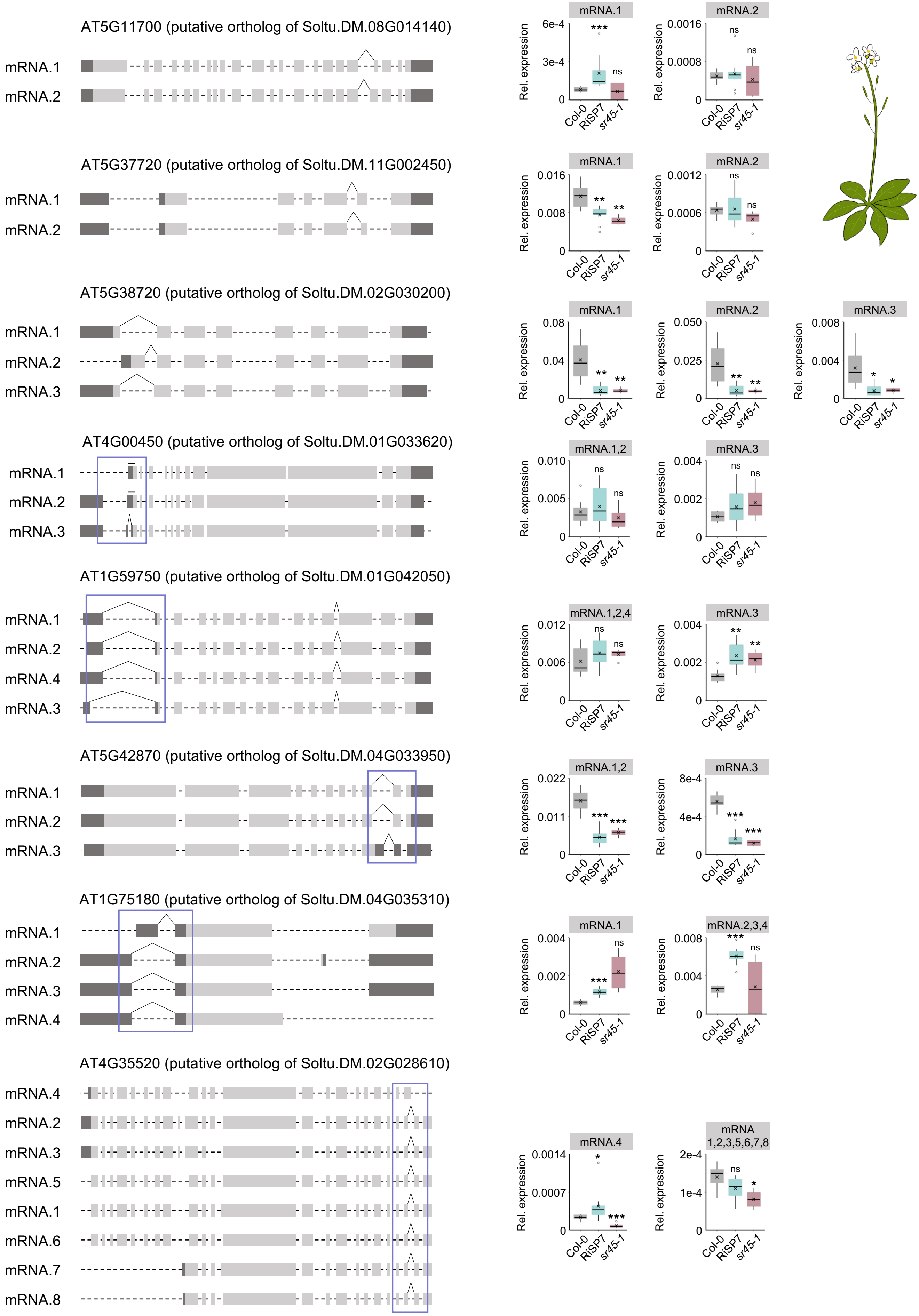

AT5G11700 (putative ortholog of Soltu.DM.08G014140)
mRNA.1
mRNA.2
AT5G37720 (putative ortholog of Soltu.DM.11G002450)
mRNA.1
mRNA.2
AT5G38720 (putative ortholog of Soltu.DM.02G030200)
mRNA.1
mRNA.2
mRNA.3
AT4G00450 (putative ortholog of Soltu.DM.01G033620)
mRNA.1
mRNA.2
mRNA.3
AT1G59750 (putative ortholog of Soltu.DM.01G042050)
mRNA.1
mRNA.2
mRNA.4
mRNA.3
AT5G42870 (putative ortholog of Soltu.DM.04G033950)
mRNA.1
mRNA.2
mRNA.3
AT1G75180 (putative ortholog of Soltu.DM.04G035310)
mRNA.1
mRNA.2
mRNA.3
mRNA.4
AT4G35520 (putative ortholog of Soltu.DM.02G028610)
mRNA.4
mRNA.2
mRNA.3
mRNA.5
mRNA.1
mRNA.6
mRNA.7
mRNA.8
mRNA.1
mRNA.2
mRNA.3
mRNA.1,2
mRNA.1,2,4
mRNA.2,3,4
mRNA 1,2,3,5,6,7,8
Rel. expression
Col-0
RiSP7
sr45-1
ns
*
**
***
6e-4
3e-4
0
0.0016
0.0008
0.016
0.008
0.0012
0.0006
0.08
0.04
0.050
0.025
0.004
0.010
0.005
0.002
0.012
0.006
0.022
0.011
8e-4
4e-4
0.0014
0.0007
2e-4
1e-4

Supplementary figure 20. Ectopic expression of *RiSP7* in *Arabidopsis thaliana* has an impact on the alternative splicing of the plant.

Splicing models and quantification of the relative expression of different splicing isoforms through qRT-PCR of some *Arabidopsis* SARs, whose putative orthologous genes in potato have been identified as RiSP7-regulated DAS genes. In the splicing models the dashed lines represent introns and the boxes exons. Boxes in dark grey correspond to untranslated regions (UTRs) and those in light grey to protein coding sequence (CDS). Expression values relative to *AtActin2* are shown as boxplots. In all boxplots, the boxes show the quartiles, the whiskers mark the maximum and minimum values, except outliers that are shown as extra dots.

Each group (RiSP7 or *sr45-1*) was compared to Col-0 and statistical significance was assessed by performing a T-test or a Mann-Whitney U test after checking the normal distribution of the data and homocedasticity as explained in Materials and Methods. Sample size was n = 8 for WT, n = 10 for RiSP7-expressing plants under control of the 2XP35S promoter (from three independent lines) and n = 4 for *sr45-1*. Significance levels: n.s. (non-significant) $p>0.05$, * $p<0.05$, ** $p<0.01$, *** $p<0.001$. Exact p-values are shown in Source Data File.

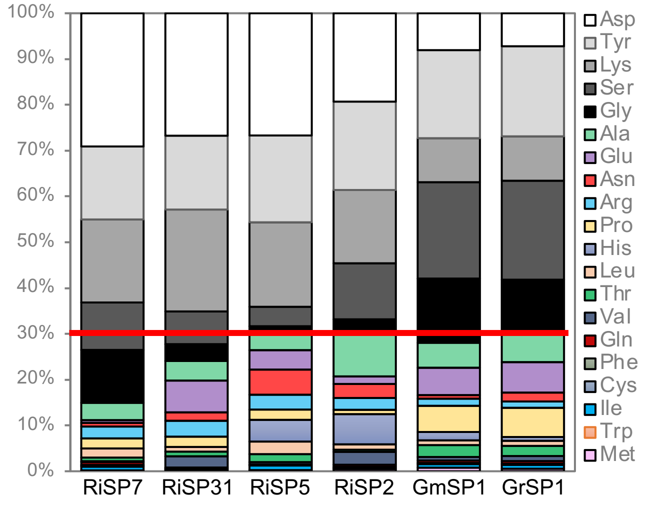

Supplementary Figure 21. Relative aa composition for each SP7 family member.

All effectors are composed of only 5 different amino acids (Asp, Tyr, Lys, Ser, Gly; white-black colors) in 70-85 % of their sequence as known for other proteins with intrinsically disordered domains. Red line indicates 70% proportion.

Supplementary references:

1 Manley, B. F. et al. A highly contiguous genome assembly reveals sources of genomic novelty in the symbiotic fungus Rhizophagus irregularis. *G3 (Bethesda)* **13** (2023). https://doi.org:10.1093/g3journal/jkad077

2 Sperschneider, J. et al. Arbuscular mycorrhizal fungi heterokaryons have two nuclear populations with distinct roles in host-plant interactions. *Nat Microbiol* **8**, 2142-2153 (2023). https://doi.org:10.1038/s41564-023-01495-8

3 Haynes, C. & Iakoucheva, L. M. Serine/arginine-rich splicing factors belong to a class of intrinsically disordered proteins. *Nucleic Acids Res* **34**, 305-312 (2006). https://doi.org:10.1093/nar/gkj424

4 Woodward, L. A., Mabin, J. W., Gangras, P. & Singh, G. The exon junction complex: a lifelong guardian of mRNA fate. *Wiley Interdiscip Rev RNA* **8** (2017). https://doi.org:10.1002/wrna.1411

5 Schlautmann, L.P. & Gehring, N.H. A Day in the Life of the Exon Junction Complex. *Biomolecules* **10**, 866 (2020). https://doi.org/10.3390/biom10060866

6 Barta, A., Kalyna, M., Reddy, A. S. Implementing a rational and consistent nomenclature for serine/arginine-rich protein splicing factors (SR proteins) in plants. *Plant Cell* **22**, 2926-9 (2010). https://doi.org/10.1105/tpc.110.078352

7 Lopato, S., Kalyna, M., Dorner, S., Kobayashi, R., Krainer, A. R., Barta, A. atSRp30, one of two SF2/ASF-like proteins from Arabidopsis thaliana, regulates splicing of specific plant genes. *Genes Dev* **15**, 987-1001 (1999). https://doi.org/10.1101/gad.13.8.987

8 Tanabe, N., Yoshimura, K., Kimura, A., Yabuta, Y., Shigeoka, S. Differential expression of alternatively spliced mRNAs of Arabidopsis SR protein homologs, atSR30 and atSR45a, in response to environmental stress. *Plant Cell Physiol* **48**,1036-49 (2007). https://doi.org/10.1093/pcp/pcm069

9 Palusa, S. G., Ali, G. S, Reddy, A. S. Alternative splicing of pre-mRNAs of Arabidopsis serine/arginine-rich proteins: regulation by hormones and stresses. *Plant J* **49**, 1091-107 (2007). https://doi.org/10.1111/j.1365-313X.2006.03020.x

10 Tanabe, N., Kimura, A., Yoshimura, K., Shigeoka, S. Plant-specific SR-related protein atSR45a interacts with spliceosomal proteins in plant nucleus. *Plant Mol Biol* **70**, 241-252 (2009). https://doi.org/10.1007/s11103-009-9469-y

11 Voß, S., Betz, R., Heidt, S., Corradi, N. & Requena, N. RiCRN1, a Crinkler Effector From the Arbuscular Mycorrhizal Fungus Rhizophagus irregularis, Functions in Arbuscule Development. *Frontiers in Microbiology* **9** (2018). https://doi.org:10.3389/fmicb.2018.02068

12 Wang, B. B. & Brendel, V. Molecular characterization and phylogeny of U2AF35 homologs in plants. *Plant Physiol* **140**, 624-36 (2006). https://doi.org/10.1104/pp.105.073858

13 Golovkin M., Reddy A. S. Structure and expression of a plant U1 snRNP 70K gene: alternative splicing of U1 snRNP 70K pre-mRNAs produces two different transcripts. *Plant Cell* **8**, 1421-35 (1996). https://doi.org/10.1105/tpc.8.8.1421

14 Golovkin, M. & Reddy, A. S. An SC35-like protein and a novel serine/arginine-rich protein interact with Arabidopsis U1-70K protein. *J Biol Chem* **274**, 36428-36438 (1999). https://doi.org:10.1074/jbc.274.51.36428

15 Xing, D., Wang, Y., Hamilton, M., Ben-Hur, A. & Reddy, A. S. Transcriptome-Wide Identification of RNA Targets of Arabidopsis SERINE/ARGININE-RICH45 Uncovers the Unexpected Roles of This RNA Binding Protein in RNA Processing. *Plant Cell* **27**, 3294-3308 (2015). https://doi.org:10.1105/tpc.15.00641

16 Zhang, X. N. et al. Transcriptome analyses reveal SR45 to be a neutral splicing regulator and a suppressor of innate immunity in Arabidopsis thaliana. *BMC Genomics* **18**, 772 (2017). https://doi.org:10.1186/s12864-017-4183-7
