# Supplementary material for: Alternative splicing regulation in plants by SP7-like effectors from symbiotic arbuscular mycorrhizal fungi
Source: Nat Commun. 2024 Aug 19;15:7107. doi: 10.1038/s41467-024-51512-5 (PMC11333574; doi:10.1038/s41467-024-51512-5)
Supplement: Supplementary file 3 — Description of additional supplementary files [file 41467_2024_51512_MOESM3_ESM.pdf]

Description of Additional Supplementary files

Supplementary Data 1: SRA BLAST search results for identification of SP7-like homologous sequences within the fungal kingdom.

BLASTn search in public available SRA gene expression datasets of various fungal species with SP7-like effector coding sequences as query were employed to find putatively expressed SP7-like effectors.

Supplementary Data 2: Identified SP7-like sequences in Mucoromycota genomes.

Available genome data from Glomeromycotina species and 19 different Mucoromycotina and Mortierellamycotina species were used to search for SP7 type effectors.

Supplementary Data 3: List of Identified plant interaction partners for SP7-like effectors using Y2H and Co-IP screens.

Different interaction approaches were used in a survey to find plant effector interaction partners. Identified plant interactors were further analyzed for functional Go Term enrichments.

Supplementary Data 4: Comparison lists of DEGs and DAS genes identified in RiSP7 expressing potato plants.

Identified DEGs and DAS genes in RiSP7 transgenic potato plants were compared to the regulation of SR45 associated RNAs from different published datasets.

Supplementary Data 5: List of Primers used in this study.
