# Supplementary material for: Alternative splicing regulation in plants by SP7-like effectors from symbiotic arbuscular mycorrhizal fungi
Source: Nat Commun. 2024 Aug 19;15:7107. doi: 10.1038/s41467-024-51512-5 (PMC11333574; doi:10.1038/s41467-024-51512-5)
Supplement: Supplementary file 9 — Reporting Summary [file 41467_2024_51512_MOESM9_ESM.pdf]

nature portfolio

Corresponding author(s): Natalia Requena

Last updated by author(s): Jul 23, 2024

Reporting Summary

Nature Portfolio wishes to improve the reproducibility of the work that we publish. This form provides structure for consistency and transparency in reporting. For further information on Nature Portfolio policies, see our Editorial Policies and the Editorial Policy Checklist.

Statistics

For all statistical analyses, confirm that the following items are present in the figure legend, table legend, main text, or Methods section.

n/a	Confirmed	
☐	☒	The exact sample size (*n*) for each experimental group/condition, given as a discrete number and unit of measurement
☐	☒	A statement on whether measurements were taken from distinct samples or whether the same sample was measured repeatedly
☐	☒	The statistical test(s) used AND whether they are one- or two-sided<br>*Only common tests should be described solely by name; describe more complex techniques in the Methods section.*
☒	☐	A description of all covariates tested
☐	☒	A description of any assumptions or corrections, such as tests of normality and adjustment for multiple comparisons
☐	☒	A full description of the statistical parameters including central tendency (e.g. means) or other basic estimates (e.g. regression coefficient) AND variation (e.g. standard deviation) or associated estimates of uncertainty (e.g. confidence intervals)
☐	☒	For null hypothesis testing, the test statistic (e.g. *F*, *t*, *r*) with confidence intervals, effect sizes, degrees of freedom and *P* value noted<br>*Give P values as exact values whenever suitable.*
☒	☐	For Bayesian analysis, information on the choice of priors and Markov chain Monte Carlo settings
☒	☐	For hierarchical and complex designs, identification of the appropriate level for tests and full reporting of outcomes
☒	☐	Estimates of effect sizes (e.g. Cohen's *d*, Pearson's *r*), indicating how they were calculated

Our web collection on statistics for biologists contains articles on many of the points above.

Software and code

Policy information about availability of computer code

Data collection	Leica, TCS-SP5P (Confocal imaging), LASAF v2.6<br>Software, BioRad iCycler MyIQ, single color
Data analysis	Excel, Statistics Kingdom and R Studio for Statistical Analyses, Fiji for image processing. For the visualization of translocation of genes in the genome, Affinity designer 2 was used. Other DNA Softwares are indicated in M & M.

For manuscripts utilizing custom algorithms or software that are central to the research but not yet described in published literature, software must be made available to editors and reviewers. We strongly encourage code deposition in a community repository (e.g. GitHub). See the Nature Portfolio guidelines for submitting code & software for further information.

Data

Policy information about availability of data

All manuscripts must include a data availability statement. This statement should provide the following information, where applicable:

- Accession codes, unique identifiers, or web links for publicly available datasets
- A description of any restrictions on data availability
- For clinical datasets or third party data, please ensure that the statement adheres to our policy

The sequences of the SP7-like effectors are available in GenBank with the following accessions: MF521604 (RiSP7), MF521605 (RiSP2), MF521606 (RiSP5), MF521607 (RiSP31), MF521608 (GmarSP1), MF521609 (GrosSP1). M. truncatula gene names are from the genome release (Mt4.0v2, http://

www.medicagogenome.org/about/project-overview). The RNAseq data generated in this project has been deposited at NCBI with the following accession number BioProject PRJNA1027625
[https://www.ncbi.nlm.nih.gov/bioproject/PRJNA1027625].

Source data are provided with this paper.

Research involving human participants, their data, or biological material

Policy information about studies with human participants or human data. See also policy information about sex, gender (identity/presentation), and sexual orientation and race, ethnicity and racism.

Reporting on sex and gender	n/a
Reporting on race, ethnicity, or other socially relevant groupings	n/a
Population characteristics	n/a
Recruitment	n/a
Ethics oversight	n/a

Note that full information on the approval of the study protocol must also be provided in the manuscript.

Field-specific reporting

Please select the one below that is the best fit for your research. If you are not sure, read the appropriate sections before making your selection.

☒ Life sciences ☐ Behavioural & social sciences ☐ Ecological, evolutionary & environmental sciences

For a reference copy of the document with all sections, see nature.com/documents/nr-reporting-summary-flat.pdf

Life sciences study design

All studies must disclose on these points even when the disclosure is negative.

Sample size	No sample size was performed, and sample size was estimated based on our and other previous established protocols.
Data exclusions	No data was excluded. Outliers in Boxplot graphics are still included in the statistical analysis.
Replication	The number of replicates is indicated in the manuscript. Main conclusions were confirmed in different assays. All experiments were reproducible
Randomization	Sample are allocated into experimental groups depending on treatments, therefore allocation was not random.
Blinding	Blinding was not used in this study.

Reporting for specific materials, systems and methods

We require information from authors about some types of materials, experimental systems and methods used in many studies. Here, indicate whether each material, system or method listed is relevant to your study. If you are not sure if a list item applies to your research, read the appropriate section before selecting a response.

Materials & experimental systems

n/a	Involved in the study	
☐	☒	Antibodies
☒	☐	Eukaryotic cell lines
☒	☐	Palaeontology and archaeology
☒	☐	Animals and other organisms
☒	☐	Clinical data
☒	☐	Dual use research of concern
☐	☒	Plants

Methods

n/a	Involved in the study	
☒	☐	ChIP-seq
☒	☐	Flow cytometry
☒	☐	MRI-based neuroimaging

Antibodies

Antibodies used	GFP-trap magnetic beads (Chromotek, Planegg-Martinsried, Germany)
Validation	GFP trap magnetics were validated by recovery of GFP peptides in the pull down experiments in the GFP control samples, included in the source data.

Plants

Seed stocks	Described in M&M
Novel plant genotypes	Described in M & M. In brief transgenic potato plants were produced through Agrobacterium tumefaciens transformation and a callus regeneration protocol. A. thaliana transformation was made using the floral dip method.
Authentication	We tested by qRT-PCR and by fluorescence if transgenic potato plants contained the transgenes. Arabidopsis transgenic plants were selected using Km selection.
