# Supplementary material for: Alternative splicing regulation in plants by SP7-like effectors from symbiotic arbuscular mycorrhizal fungi
Source: Nat Commun. 2024 Aug 19;15:7107. doi: 10.1038/s41467-024-51512-5 (PMC11333574; doi:10.1038/s41467-024-51512-5)
Supplement: Supplementary file 10 — Source Data [file 41467_2024_51512_MOESM10_ESM.zip › Requena_8071-1_ProteinSummary (RiSP7+SP).pdf]

Mascot Search Results

User :
Email :
Search title : 8071-1
MS data file : 8071_1.mgf
Database : TopLab_8071 8071_20160923 (76598 sequences; 22305840 residues)
Timestamp : 1 Dec 2016 at 12:41:47 GMT
Enzyme : Trypsin
Fixed modifications : Carbamidomethyl (C)
Variable modifications : Oxidation (M)
Mass values : Monoisotopic
Protein Mass : Unrestricted
Peptide Mass Tolerance : ± 50 ppm
Fragment Mass Tolerance: ± 0.6 Da
Max Missed Cleavages : 1
Instrument type : Default
Number of queries : 21635
Protein hits :

NbS00023525g0004.1	NbS00023525g0004.1 protein AED:0.27 eAED:0.28 QI:0|0|0|0.5|1|1|2|0|137; (*GB) gi|356576357|ref|XP_003556299.1| (e_value=4e-60) PREDICTED: probabl
NbS00031648g0011.1	NbS00031648g0011.1 protein AED:0.23 eAED:0.23 QI:280|1|1|1|0.95|0.91|23|300|916; (*GB) gi|225465253|ref|XP_002268311.1| (e_value=0.0) PREDICTED:
gi|297262447|ref|XP_001098182.2|	gi|297262447|ref|XP_001098182.2| PREDICTED: keratin, type II cytoskeletal 1-like isoform 6 [Macaca mulatta]
NbS00021492g0001.1	NbS00021492g0001.1 protein AED:0.22 eAED:0.25 QI:0|0.70|0.72|0.94|0.94|0.88|18|255|639; (*SWP) sp|Q39828|SDL5A_SOYBN (e_value=0.0) Dynamin-relate
gi|28317|emb|CAA32649.1|	gi|28317|emb|CAA32649.1| unnamed protein product [Homo sapiens]
NbS00056353g0008.1	NbS00056353g0008.1 protein AED:0.25 eAED:0.25 QI:0|0.92|0.85|1|0.92|0.92|14|0|554; (*SWP) sp|Q9FNX5|DRP1E_ARATH (e_value=0.0) Dynamin-related prc
NbS00008178g0018.1	NbS00008178g0018.1 protein AED:0.03 eAED:0.03 QI:116|1|1|1|1|1|2|157|148; (*GB) gi|27529852|dbj|BAC53941.1| (e_value=4e-56) H2A histone [Nicotian
gi|148727309|ref|NP_001092039.1|	gi|148727309|ref|NP_001092039.1| keratin, type II cytoskeletal 2 epidermal [Pan troglodytes]
gi|291410763|ref|XP_002721657.1|	gi|291410763|ref|XP_002721657.1| PREDICTED: histone cluster 1, H2ag-like [Oryctolagus cuniculus]
NbS00054358g0003.1	NbS00054358g0003.1 protein AED:0.12 eAED:0.14 QI:177|0.91|0.84|1|0.91|0.84|13|0|563; (*GB) gi|359489241|ref|XP_003633899.1| (e_value=0.0) PREDICT
NbS00000471g0009.1	NbS00000471g0009.1 protein AED:0.15 eAED:0.15 QI:0|0|0.33|1|1|1|3|0|301; (*GB) gi|115802|sp|P27494.1|CB23_TOBAC (e_value=0.0) RecName: Full=Chlor
NbS00001169g0153.1	NbS00001169g0153.1 protein AED:0.25 eAED:0.25 QI:0|0.5|0.4|1|1|1|5|0|341
NbS00027807g0002.1	NbS00027807g0002.1 protein AED:0.05 eAED:0.05 QI:0|1|0.5|1|0|0|2|953|413; (*GB) gi|68566313|sp|Q40450.2|EFTUA_NICSY (e_value=0.0) RecName: Full=E
NbS00004898g0002.1	NbS00004898g0002.1 protein AED:0.00 eAED:0.00 QI:0|-1|0|1|-1|1|1|0|103; (*GB) gi|195617694|gb|ACG30677.1| (e_value=1e-51) histone H4 [Zea mays];;
NbS00001942g0006.1	NbS00001942g0006.1 protein AED:0.00 eAED:0.00 QI:75|1|1|1|1|1|2|198|151; (*GB) gi|351725389|ref|NP_001235555.1| (e_value=1e-63) uncharacterized p
NbS00010297g0001.1	NbS00010297g0001.1 protein AED:0.04 eAED:0.04 QI:122|1|1|1|1|1|2|350|144; (*GB) gi|225439749|ref|XP_002274570.1| (e_value=6e-44) PREDICTED: proba
NbS00017066g0003.1	NbS00017066g0003.1 protein AED:0.01 eAED:0.01 QI:0|-1|0|1|-1|1|1|0|208; (*GB) gi|222051768|dbj|BAH15357.1| (e_value=3e-127) germin like protein [
NbS00033686g0006.1	NbS00033686g0006.1 protein AED:0.16 eAED:0.16 QI:197|1|1|1|1|1|15|214|623; (*GB) gi|225453246|ref|XP_002265511.1| (e_value=0.0) PREDICTED: dynami
NbS00000987g0004.1	NbS00000987g0004.1 protein AED:0.21 eAED:0.21 QI:0|-1|0|1|-1|1|1|0|266; (*GB) gi|3036948|dbj|BAA25392.1| (e_value=0.0) light harvesting chlorophy
NbS00011945g0012.1	NbS00011945g0012.1 protein AED:0.16 eAED:0.16 QI:0|0|0|1|0|0.5|2|147|203; (*GB) gi|195617694|gb|ACG30677.1| (e_value=3e-50) histone H4 [Zea mays]
NbS00010009g0002.1	NbS00010009g0002.1 protein AED:0.20 eAED:0.20 QI:0|1|1|1|1|1|21|408|1058; (*SWP) sp|O04379|AGO1_ARATH (e_value=0.0) Protein argonaute 1 OS=Arabid
NbS00014580g0002.1	NbS00014580g0002.1 protein AED:0.38 eAED:0.38 QI:0|-1|0|1|-1|1|1|0|265; (*GB) gi|3036944|dbj|BAA25389.1| (e_value=0.0) light harvesting chlorophy
NbS00000867g0011.1	NbS00000867g0011.1 protein AED:0.34 eAED:0.34 QI:171|0.9|0.90|1|0.7|0.63|11|450|270; (*GB) gi|359492084|ref|XP_002284740.2| (e_value=1e-70) PREDI
NbS00010583g0001.1	NbS00010583g0001.1 protein AED:0.25 eAED:0.25 QI:0|-1|0|1|-1|1|1|0|210; (*GB) gi|31711507|dbj|BAC77634.1| (e_value=2e-132) 24K germin like protei
NbS00007511g0005.1	NbS00007511g0005.1 protein AED:0.26 eAED:0.26 QI:0|0.62|0.55|0.88|1|1|9|0|1084; (*GB) gi|359477631|ref|XP_002274485.2| (e_value=0.0) PREDICTED: u
NbS00004085g0015.1	NbS00004085g0015.1 protein AED:0.29 eAED:0.29 QI:92|0.25|0.2|1|1|1|5|0|225; (*GB) gi|110377793|gb|ABG73417.1| (e_value=7e-87) chloroplast pigment
NbS00014977g0008.1	NbS00014977g0008.1 protein AED:0.16 eAED:0.16 QI:16|0.95|0.90|1|0.95|0.90|22|432|1125; (*GB) gi|84688908|gb|ABC61503.1| (e_value=0.0) AGO1-2, par
NbS00035687g0007.1	NbS00035687g0007.1 protein AED:0.23 eAED:0.23 QI:300|1|0.75|1|1|1|4|0|274; (*GB) gi|84620802|gb|ABC59515.1| (e_value=9e-120) chloroplast photosys
NbS00000671g0042.1	NbS00000671g0042.1 protein AED:0.13 eAED:0.13 QI:0|0.83|0.57|1|1|0.85|7|0|571; (*GB) gi|729623|sp|Q03685.1|BIP5_TOBAC (e_value=0.0) RecName: Full
NbS00007993g0305.1	NbS00007993g0305.1 protein AED:0.00 eAED:0.00 QI:0|-1|0|1|-1|1|1|0|261
NbS00019305g0027.1	NbS00019305g0027.1 protein AED:0.37 eAED:0.37 QI:0|1|0.88|1|1|1|9|0|556; (*GB) gi|3676296|gb|AAD03392.1| (e_value=0.0) mitochondrial ATPase beta
NbS00043180g0003.1	NbS00043180g0003.1 protein AED:0.10 eAED:0.10 QI:0|1|0.5|1|1|1|4|944|486; (*GB) gi|113461|sp|P27081.1|ADT2_SOLTU (e_value=0.0) RecName: Full=ADP,
NbS00009714g0011.1	NbS00009714g0011.1 protein AED:0.20 eAED:0.20 QI:131|0.83|0.71|1|0.83|0.57|7|463|410; (*SWP) sp|Q40565|RCA2_TOBAC (e_value=0.0) Ribulose bisphosp
NbS00019623g0001.1	NbS00019623g0001.1 protein AED:0.21 eAED:0.21 QI:0|1|0.5|1|1|1|2|0|447; (*GB) gi|3869088|dbj|BAA34348.1| (e_value=0.0) elongation factor-1 alpha
NbS00019862g0010.1	NbS00019862g0010.1 protein AED:0.10 eAED:0.10 QI:0|0.92|1|1|0.92|0.93|15|328|640; (*GB) gi|5931765|emb|CAB56619.1| (e_value=0.0) phragmoplastin [
NbS00042109g0018.1	NbS00042109g0018.1 protein AED:0.00 eAED:0.00 QI:0|1|0.5|1|1|1|2|0|256; (*SWP) sp|P27524|CB4A_SOLLC (e_value=3e-163) Chlorophyll a-b binding prot
NbS00010295g0007.1	NbS00010295g0007.1 protein AED:0.12 eAED:0.12 QI:0|0|0|0.5|1|1|2|0|119; (*SWP) sp|P69569|RBL_EUPES (e_value=5e-22) Ribulose bisphosphate carboxyl
NbS00029393g0012.1	NbS00029393g0012.1 protein AED:0.08 eAED:0.08 QI:319|0.42|0.37|1|1|1|8|307|444; (*GB) gi|12643757|sp|Q40460.1|RCA1_TOBAC (e_value=0.0) RecName: F
NbS00033593g0008.1	NbS00033593g0008.1 protein AED:0.04 eAED:0.05 QI:310|0|0.5|1|0|0|2|0|280; (*GB) gi|388491254|gb|AFK33693.1| (e_value=3e-98) unknown [Lotus japoni
NbS00008911g0002.1	NbS00008911g0002.1 protein AED:0.35 eAED:0.35 QI:460|0.77|0.7|1|1|1|10|229|443; (*GB) gi|327198779|emb|CBL43264.1| (e_value=0.0) glyceraldehyde-3
NbS00002894g0003.1	NbS00002894g0003.1 protein AED:0.02 eAED:0.02 QI:0|1|0.5|1|1|1|2|0|386; (*GB) gi|78191448|gb|ABB29945.1| (e_value=0.0) ADP/ATP translocator-like
NbS00010663g0016.1	NbS00010663g0016.1 protein AED:0.20 eAED:0.20 QI:7|1|0.88|1|1|1|9|298|622; (*GB) gi|224135925|ref|XP_002322195.1| (e_value=0.0) predicted protein
NbS00004793g0016.1	NbS00004793g0016.1 protein AED:0.15 eAED:0.27 QI:0|0|0|1|1|1|2|0|286; (*SWP) sp|P27492|CB21_TOBAC (e_value=9e-155) Chlorophyll a-b binding protei

NbS00000634g0101.1 NbS00000634g0101.1 protein AED:0.21 eAED:0.21 QI:0|-1|0|1|-1|1|1|0|136
NbS00003380g0115.1 NbS00003380g0115.1 protein AED:0.12 eAED:0.12 QI:355|0.66|0.85|1|0.33|0.42|7|134|238
NbS00010743g0014.1 NbS00010743g0014.1 protein AED:0.24 eAED:0.24 QI:0|1|1|1|1|1|8|290|288; (*GB) gi|77416949|gb|ABA81870.1| (e_value=2e-155) unknown [Solanum tuberc
NbS00011757g0115.1 NbS00011757g0115.1 protein AED:0.33 eAED:0.33 QI:0|0.57|0.37|1|0.85|0.75|8|0|590
NbS00045545g0004.1 NbS00045545g0004.1 protein AED:0.13 eAED:0.13 QI:0|0.78|0.73|0.86|0.92|0.86|15|481|616; (*SWP) sp|Q7XTT4|NUCL2_ORYSJ (e_value=6e-73) Nucleolin 2
NbS00006116g0019.1 NbS00006116g0019.1 protein AED:0.27 eAED:0.27 QI:568|1|1|1|1|1|7|0|473; (*GB) gi|219560127|gb|ACL27272.1| (e_value=0.0) catalase [Nicotiana benth
sp|TRYP_PIG| sp|TRYP_PIG|
NbS00002621g0212.1 NbS00002621g0212.1 protein AED:0.19 eAED:0.19 QI:122|0.8|1|1|1|1|6|306|255
NbS00006644g0116.1 NbS00006644g0116.1 protein AED:0.00 eAED:0.00 QI:364|1|1|1|1|1|3|541|1093
NbS00020769g0006.1 NbS00020769g0006.1 protein AED:0.25 eAED:0.25 QI:307|0.66|1|1|0.66|0.75|4|448|250; (*GB) gi|84620804|gb|ABC59516.1| (e_value=5e-102) chloroplast
NbS00000681g0006.1 NbS00000681g0006.1 protein AED:0.29 eAED:0.30 QI:89|0.75|0.88|1|0.75|0.77|9|282|301; (*GB) gi|358248282|ref|NP_001239854.1| (e_value=7e-62) uncha
NbS00021398g0012.1 NbS00021398g0012.1 protein AED:0.33 eAED:0.35 QI:0|0.81|0.75|1|0.81|0.75|12|0|838; (*GB) gi|225450401|ref|XP_002278318.1| (e_value=0.0) PREDICTED
NbS00001988g0005.1 NbS00001988g0005.1 protein AED:0.30 eAED:0.30 QI:0|1|0.83|1|1|1|6|0|610; (*GB) gi|225441549|ref|XP_002281113.1| (e_value=0.0) PREDICTED: DEAD-box
NbS00001538g0002.1 NbS00001538g0002.1 protein AED:0.19 eAED:0.19 QI:248|1|1|1|1|1|11|261|788; (*SWP) sp|Q7TP47|HNRPQ_RAT (e_value=2e-31) Heterogeneous nuclear ribon
NbS00001559g0021.1 NbS00001559g0021.1 protein AED:0.25 eAED:0.31 QI:138|0.5|0.53|1|0.85|0.93|15|300|837; (*GB) gi|356513635|ref|XP_003525517.1| (e_value=0.0) PREDIC
NbS00021832g0023.1 NbS00021832g0023.1 protein AED:0.35 eAED:0.35 QI:0|0|0|1|0|0|2|0|80; (*GB) gi|305671961|gb|ADM63350.1| (e_value=2e-44) photosystem II cp47 protei
NbS00001259g0009.1 NbS00001259g0009.1 protein AED:0.04 eAED:0.04 QI:128|0.75|0.77|1|0.75|0.88|9|0|318; (*GB) gi|357505379|ref|XP_003622978.1| (e_value=2e-55) Ribonu
NbS00017935g0003.1 NbS00017935g0003.1 protein AED:0.00 eAED:0.00 QI:240|1|0.5|1|1|1|2|0|284; (*SWP) sp|Q9XF88|CB4B_ARATH (e_value=4e-162) Chlorophyll a-b binding pr
NbS00022486g0001.1 NbS00022486g0001.1 protein AED:0.21 eAED:0.21 QI:0|1|0.66|1|1|1|3|0|181; (*GB) gi|132118|sp|P26573.1|RBS8_NICPL (e_value=4e-116) RecName: Full=Ri
NbS00004956g0015.1 NbS00004956g0015.1 protein AED:0.25 eAED:0.30 QI:0|0|0|1|0|0|2|0|84; (*GB) gi|42718201|gb|AAS38532.1| (e_value=7e-41) ribulose-1,5-bisphosphate c
NbS00016159g0006.1 NbS00016159g0006.1 protein AED:0.34 eAED:0.36 QI:356|0.57|0.5|1|0.71|0.62|8|0|302; (*GB) gi|115473|sp|P27141.1|CAHC_TOBAC (e_value=3e-179) RecNam
NbS00014345g0007.1 NbS00014345g0007.1 protein AED:0.10 eAED:0.10 QI:698|0.5|0.61|0.76|0.75|0.76|13|0|1156; (*GB) gi|359472595|ref|XP_002281257.2| (e_value=0.0) PRED
NbS00036430g0009.1 NbS00036430g0009.1 protein AED:0.35 eAED:0.35 QI:0|0|0|1|0|0|4|0|448; (*GB) gi|113170490|ref|YP_717281.1| (e_value=2e-170) Atp1 [Ostreococcus tau
NbS00004717g0103.1 NbS00004717g0103.1 protein AED:0.03 eAED:0.03 QI:0|-1|0|1|-1|1|1|0|128
NbS00028679g0001.1 NbS00028679g0001.1 protein AED:0.08 eAED:0.08 QI:281|0.88|0.9|1|0.88|0.8|10|299|305; (*GB) gi|255542956|ref|XP_002512541.1| (e_value=0.0) NAD dep
NbS00011040g0001.1 NbS00011040g0001.1 protein AED:0.00 eAED:0.03 QI:0|1|0.66|1|0|0|3|164|270; (*GB) gi|255578051|ref|XP_002529896.1| (e_value=1e-58) small nuclear r
NbS00047378g0007.1 NbS00047378g0007.1 protein AED:0.08 eAED:0.08 QI:0|0.9|0.90|1|0.9|0.90|11|3|723; (*GB) gi|255561268|ref|XP_002521645.1| (e_value=0.0) arsenite-re
NbS00010277g0007.1 NbS00010277g0007.1 protein AED:0.15 eAED:0.15 QI:45|1|1|1|0.81|0.75|12|537|515; (*GB) gi|350538893|ref|NP_001234620.1| (e_value=0.0) beta-mannosi
NbS00006224g0007.1 NbS00006224g0007.1 protein AED:0.32 eAED:0.43 QI:327|0.6|0.66|0.83|0.6|0.66|6|0|143; (*GB) gi|319903976|gb|ADV77190.1| (e_value=2e-51) histone H3
NbS00004238g0018.1 NbS00004238g0018.1 protein AED:0.16 eAED:0.16 QI:156|0.87|0.88|1|0.87|0.77|9|398|304; (*GB) gi|77416949|gb|ABA81870.1| (e_value=2e-140) unknown [
NbS00000485g0008.1 NbS00000485g0008.1 protein AED:0.27 eAED:0.27 QI:81|1|1|1|0.8|0.83|6|329|238; (*GB) gi|225442156|ref|XP_002275541.1| (e_value=6e-155) PREDICTED:
NbS00027201g0002.1 NbS00027201g0002.1 protein AED:0.33 eAED:0.33 QI:0|1|0.75|1|1|1|4|0|180; (*GB) gi|59800169|sp|P69249.1|RBS_TOBAC (e_value=9e-123) RecName: Full=R
NbS00002044g0005.1 NbS00002044g0005.1 protein AED:0.28 eAED:0.28 QI:0|1|0.66|1|1|1|3|0|273; (*GB) gi|226872|prf||1609235A (e_value=3e-155) chlorophyll a/b binding p
NbS00005969g0002.1 NbS00005969g0002.1 protein AED:0.07 eAED:0.07 QI:0|0.5|0.55|0.88|1|1|9|177|561; (*GB) gi|225456270|ref|XP_002283518.1| (e_value=0.0) PREDICTED: n
NbS00010087g0103.1 NbS00010087g0103.1 protein AED:0.35 eAED:0.35 QI:0|0|0|0.66|1|1|3|0|235
NbC24305910g0003.1 NbC24305910g0003.1 protein AED:0.03 eAED:0.03 QI:0|1|0.5|1|1|1|2|0|101; (*GB) gi|12643758|sp|Q40565.1|RCA2_TOBAC (e_value=6e-65) RecName: Full=Ri
NbS00012584g0001.1 NbS00012584g0001.1 protein AED:0.12 eAED:0.12 QI:0|-1|0|1|-1|1|1|0|370; (*GB) gi|231610|sp|P29790.1|ATPG_TOBAC (e_value=0.0) RecName: Full=ATP sy
NbS00036785g0006.1 NbS00036785g0006.1 protein AED:0.27 eAED:0.32 QI:267|0.88|0.8|1|0.77|0.6|10|263|336; (*GB) gi|356572914|ref|XP_003554610.1| (e_value=0.0) PREDICT
NbS00027670g0006.1 NbS00027670g0006.1 protein AED:0.24 eAED:0.24 QI:614|0.93|0.93|1|1|1|16|163|536; (*SWP) sp|P50433|GLYM_SOLTU (e_value=0.0) Serine hydroxymethyltr
NbS00042812g0008.1 NbS00042812g0008.1 protein AED:0.19 eAED:0.19 QI:0|1|1|1|1|1|4|386|180; (*GB) gi|132118|sp|P26573.1|RBS8_NICPL (e_value=3e-119) RecName: Full=Rib
NbS00009983g0008.1 NbS00009983g0008.1 protein AED:0.11 eAED:0.11 QI:0|0|0|1|0|0.5|2|0|384; (*GB) gi|108864705|gb|ABG22608.1| (e_value=0.0) Heat shock cognate 70 kDa
NbS00059497g0003.1 NbS00059497g0003.1 protein AED:0.17 eAED:0.17 QI:0|1|1|1|1|1|3|550|697; (*SWP) sp|Q653H7|ARFR_ORYSJ (e_value=0.0) Auxin response factor 18 OS=Ory
NbS00051197g0005.1 NbS00051197g0005.1 protein AED:0.14 eAED:0.15 QI:0|0.88|0.8|1|1|1|10|0|891; (*SWP) sp|Q9FFQ1|RH31_ARATH (e_value=0.0) DEAD-box ATP-dependent RNA
NbS00036674g0013.1 NbS00036674g0013.1 protein AED:0.14 eAED:0.17 QI:0|0.5|0.4|1|0.5|0.4|5|0|1730; (*GB) gi|225433894|ref|XP_002266580.1| (e_value=0.0) PREDICTED: U5
NbS00011860g0002.1 NbS00011860g0002.1 protein AED:0.14 eAED:0.14 QI:0|0.83|0.76|0.84|1|1|13|0|788; (*GB) gi|255561268|ref|XP_002521645.1| (e_value=0.0) arsenite-res
gi|119581148|gb|EAW60744.1| gi|119581148|gb|EAW60744.1| keratin 9 (epidermolytic palmoplantar keratoderma) [Homo sapiens]
NbS00007742g0006.1 NbS00007742g0006.1 protein AED:0.40 eAED:0.42 QI:0|0.5|0.42|1|1|1|7|0|319; (*GB) gi|94466659|emb|CAJ44458.1| (e_value=2e-110) ALY protein [Nicoti
NbS00008510g0008.1 NbS00008510g0008.1 protein AED:0.13 eAED:0.13 QI:0|0.89|0.86|0.98|0.93|0.94|50|0|2033; (*GB) gi|296085156|emb|CBI28651.3| (e_value=0.0) unnamed p
NbS00003662g0021.1 NbS00003662g0021.1 protein AED:0.24 eAED:0.34 QI:0|0|0|1|0|0|3|0|257; (*GB) gi|78102516|ref|YP_358657.1| (e_value=6e-112) ATP synthase CF1 alpha
NbS00001413g0059.1 NbS00001413g0059.1 protein AED:0.42 eAED:0.46 QI:0|0|0|0.8|0.25|0.6|5|0|613; (*GB) gi|68164803|ref|YP_247599.1| (e_value=3e-131) photosystem I P7
gi|297692195|ref|XP_002823453.1| gi|297692195|ref|XP_002823453.1| PREDICTED: LOW QUALITY PROTEIN: ATP synthase subunit beta, mitochondrial-like [Pongo abelii]
NbS00000548g0008.1 NbS00000548g0008.1 protein AED:0.29 eAED:0.30 QI:0|0.8|0.5|1|1|0.66|6|0|363; (*GB) gi|255573386|ref|XP_002527619.1| (e_value=2e-130) Ras-GTPase-a
NbS00015187g0009.1 NbS00015187g0009.1 protein AED:0.20 eAED:0.20 QI:141|0.94|0.94|1|0.72|0.84|19|64|510; (*GB) gi|356504541|ref|XP_003521054.1| (e_value=0.0) PREDIC
NbS00038786g0002.1 NbS00038786g0002.1 protein AED:0.22 eAED:0.22 QI:290|1|1|1|0.89|0.85|20|385|1023; (*GB) gi|359474892|ref|XP_002276432.2| (e_value=0.0) PREDICTED:
NbS00020253g0009.1 NbS00020253g0009.1 protein AED:0.18 eAED:0.18 QI:226|1|1|1|1|1|3|156|265; (*GB) gi|115794|sp|P27489.1|CB23_SOLLC (e_value=2e-176) RecName: Full=C
>gi|74181742|dbj|BAE32582.1| >gi|74181742|dbj|BAE32582.1| unnamed protein product [Mus musculus]
NbS00003163g0002.1 NbS00003163g0002.1 protein ; (*GB) gi|146188483|emb|CAK12837.1| (e_value=1e-52) ribulose 1,5 biphosphate carboxylase/oxygenase [Liparia genistc
NbS00003291g0003.1 NbS00003291g0003.1 protein AED:0.07 eAED:0.07 QI:121|0.83|0.85|1|1|1|7|382|226; (*TAIR) AT3G07030.1 (e_value=4e-47) | Symbols: | Alba DNA/RNA-bi

<table>
<tr><td>NbS00025223g0014.1</td><td>NbS00025223g0014.1 protein AED:0.37 eAED:0.37 QI:0|1|0.33|1|1|0.66|3|0|136; (*GB) gi|51490663|emb|CAG26902.1| (e_value=3e-63) ALY protein [Nicoti</td></tr>
<tr><td>NbS00036843g0004.1</td><td>NbS00036843g0004.1 protein AED:0.04 eAED:0.04 QI:8|0.5|0.66|1|0.5|0.33|3|0|387; (*SWP) sp|P25083|ADT1_SOLTU (e_value=0.0) ADP,ATP carrier protein</td></tr>
<tr><td>NbS00013798g0018.1</td><td>NbS00013798g0018.1 protein AED:0.47 eAED:0.47 QI:0|0|0|1|0|0.5|2|0|68; (*GB) gi|55977763|sp|P00823.2|ATPA_TOBAC (e_value=7e-32) RecName: Full=ATP</td></tr>
<tr><td>NbS00019153g0015.1</td><td>NbS00019153g0015.1 protein AED:0.14 eAED:0.14 QI:159|0.84|0.88|1|0.96|0.92|27|471|1387; (*GB) gi|125535486|gb|EAY81974.1| (e_value=0.0) hypotheti</td></tr>
<tr><td>NbS00044222g0007.1</td><td>NbS00044222g0007.1 protein AED:0.20 eAED:0.20 QI:1972|0.73|0.68|0.87|0.73|0.62|16|0|619; (*GB) gi|225463033|ref|XP_002267199.1| (e_value=0.0) PRE</td></tr>
<tr><td>NbS00002935g0006.1</td><td>NbS00002935g0006.1 protein AED:0.24 eAED:0.28 QI:9|0.53|0.35|1|0.84|0.71|14|0|493; (*GB) gi|6715512|gb|AAF26445.1| (e_value=0.0) vacuolar H+-ATPa</td></tr>
<tr><td>NbS00006964g0008.1</td><td>NbS00006964g0008.1 protein AED:0.23 eAED:0.23 QI:394|0.87|1|1|0.93|0.88|17|339|721; (*GB) gi|255552828|ref|XP_002517457.1| (e_value=0.0) H(\+)-tr</td></tr>
<tr><td>NbS00004901g0005.1</td><td>NbS00004901g0005.1 protein AED:0.05 eAED:0.05 QI:0|1|0.5|1|1|1|4|0|391; (*GB) gi|231503|sp|P30171.1|ACT11_SOLTU (e_value=0.0) RecName: Full=Actin</td></tr>
<tr><td>NbS00003075g0011.1</td><td>NbS00003075g0011.1 protein AED:0.28 eAED:0.28 QI:0|1|0.66|1|1|1|3|0|143; (*GB) gi|2499967|sp|Q41229.1|PSAEB_NICSY (e_value=1e-59) RecName: Full=P</td></tr>
<tr><td>NbS00024580g0005.1</td><td>NbS00024580g0005.1 protein AED:0.21 eAED:0.21 QI:87|0.83|0.85|1|0.83|0.85|7|278|321; (*SWP) sp|Q641W4|RFC2_RAT (e_value=5e-157) Replication facto</td></tr>
<tr><td>NbS00018023g0003.1</td><td>NbS00018023g0003.1 protein AED:0.25 eAED:0.25 QI:0|1|0.75|1|1|1|4|510|435; (*GB) gi|297845476|ref|XP_002890619.1| (e_value=2e-95) hypothetical pr</td></tr>
<tr><td>NbS00009996g0026.1</td><td>NbS00009996g0026.1 protein AED:0.33 eAED:0.33 QI:87|0.83|0.71|1|0.66|0.85|7|314|282; (*GB) gi|224074887|ref|XP_002304476.1| (e_value=7e-172) pred</td></tr>
<tr><td>NbS00010860g0014.1</td><td>NbS00010860g0014.1 protein AED:0.05 eAED:0.05 QI:88|1|1|1|1|1|5|242|88; (*GB) gi|225468340|ref|XP_002272246.1| (e_value=5e-54) PREDICTED: probabl</td></tr>
<tr><td>NbS00001587g0026.1</td><td>NbS00001587g0026.1 protein AED:0.19 eAED:0.20 QI:234|0.91|0.83|1|0.82|0.83|24|284|993; (*SWP) sp|P35601|RFC1_MOUSE (e_value=5e-125) Replication f</td></tr>
<tr><td>NbS00013764g0007.1</td><td>NbS00013764g0007.1 protein AED:0.11 eAED:0.11 QI:116|0.85|0.87|1|0.85|0.75|8|606|455; (*GB) gi|1705613|sp|P49319.2|CATA1_TOBAC (e_value=0.0) RecN</td></tr>
<tr><td>NbS00003763g0016.1</td><td>NbS00003763g0016.1 protein AED:0.10 eAED:0.12 QI:0|0.92|0.8|0.93|1|1|15|0|884; (*GB) gi|225441896|ref|XP_002284404.1| (e_value=0.0) PREDICTED: un</td></tr>
<tr><td>NbS00005390g0012.1</td><td>NbS00005390g0012.1 protein AED:0.37 eAED:0.37 QI:218|1|1|1|1|1|4|363|114; (*GB) gi|296086730|emb|CBI32365.3| (e_value=4e-61) unnamed protein prod</td></tr>
<tr><td>NbS00015339g0013.1</td><td>NbS00015339g0013.1 protein AED:0.20 eAED:0.20 QI:0|0.95|0.80|1|1|0.95|21|0|931; (*GB) gi|584795|sp|Q08436.1|PMA3_NICPL (e_value=0.0) RecName: Ful</td></tr>
<tr><td>NbS00003616g0011.1</td><td>NbS00003616g0011.1 protein AED:0.18 eAED:0.19 QI:0|0.5|0.33|1|1|1|3|0|390; (*SWP) sp|Q9LVA0|BAG7_ARATH (e_value=1e-62) BAG family molecular chape</td></tr>
<tr><td>NbS00004261g0112.1</td><td>NbS00004261g0112.1 protein AED:0.31 eAED:0.34 QI:6|0.92|0.92|1|0.76|0.71|14|341|438</td></tr>
<tr><td>NbS00001859g0006.1</td><td>NbS00001859g0006.1 protein AED:0.10 eAED:0.10 QI:0|0.42|0.5|1|0.85|0.75|8|0|558; (*GB) gi|313585890|gb|ADR71054.1| (e_value=0.0) phosphoglycerate</td></tr>
<tr><td>NbS00028776g0007.1</td><td>NbS00028776g0007.1 protein AED:0.22 eAED:0.37 QI:0|0.6|0.16|1|0.8|0.66|6|0|494; (*GB) gi|262192739|gb|ACY30439.1| (e_value=4e-163) hypothetical p</td></tr>
<tr><td>NbS00013589g0010.1</td><td>NbS00013589g0010.1 protein AED:0.15 eAED:0.18 QI:0|0.33|0|0.75|0.66|0.5|4|0|390; (*SWP) sp|Q9LMR3|TYRA2_ARATH (e_value=4e-132) Arogenate dehydrog</td></tr>
<tr><td>NbS00019903g0005.1</td><td>NbS00019903g0005.1 protein AED:0.20 eAED:0.20 QI:0|1|1|1|1|1|20|174|1434; (*GB) gi|75326590|sp|Q76CU2.1|PDR1_TOBAC (e_value=0.0) RecName: Full=Pl</td></tr>
<tr><td>NbS00056603g0002.1</td><td>NbS00056603g0002.1 protein AED:0.27 eAED:0.27 QI:0|0.33|0.5|1|1|1|4|0|363; (*GB) gi|340501186|gb|EGR27996.1| (e_value=0.0) hypothetical protein I</td></tr>
<tr><td>NbS00004937g0014.1</td><td>NbS00004937g0014.1 protein AED:0.10 eAED:0.10 QI:88|1|1|1|0.57|1|8|322|272; (*GB) gi|356566431|ref|XP_003551435.1| (e_value=3e-54) PREDICTED: unc</td></tr>
<tr><td>gi|3336842|emb|CAA76847.1|</td><td>gi|3336842|emb|CAA76847.1| bovine serum albumin [Bos taurus]</td></tr>
<tr><td>NbS00061449g0009.1</td><td>NbS00061449g0009.1 protein AED:0.21 eAED:0.23 QI:76|1|0.9|1|0.77|0.9|10|0|443; (*SWP) sp|Q8RXH2|NUP85_ARATH (e_value=2e-164) Nuclear pore complex</td></tr>
<tr><td>NbS00047628g0009.1</td><td>NbS00047628g0009.1 protein AED:0.27 eAED:0.27 QI:381|1|1|1|1|1|10|557|477; (*GB) gi|217074984|gb|ACJ85852.1| (e_value=0.0) unknown [Medicago trun</td></tr>
<tr><td>NbS00001849g0017.1</td><td>NbS00001849g0017.1 protein AED:0.76 eAED:1.00 QI:0|0|0|0.66|0.5|0.66|3|0|72; (*GB) gi|94466657|emb|CAJ44457.1| (e_value=3e-26) ALY protein [Nicot</td></tr>
<tr><td>NbS00008453g0109.1</td><td>NbS00008453g0109.1 protein AED:0.28 eAED:0.44 QI:0|0|0|0.66|0.5|0.33|3|0|219</td></tr>
<tr><td>NbS00004515g0209.1</td><td>NbS00004515g0209.1 protein AED:0.13 eAED:0.13 QI:829|0.9|0.90|1|0.6|0.63|11|284|876</td></tr>
<tr><td>NbS00002447g0019.1</td><td>NbS00002447g0019.1 protein AED:0.23 eAED:0.23 QI:0|0.69|0.71|1|0.92|1|14|107|458; (*GB) gi|225451717|ref|XP_002279575.1| (e_value=0.0) PREDICTED:</td></tr>
<tr><td>NbS00030630g0007.1</td><td>NbS00030630g0007.1 protein</td></tr>
<tr><td>NbS00013799g0011.1</td><td>NbS00013799g0011.1 protein AED:0.34 eAED:0.34 QI:205|0.88|0.88|1|0.76|0.77|18|0|685; (*GB) gi|225427716|ref|XP_002265086.1| (e_value=0.0) PREDICT</td></tr>
<tr><td>NbS00001525g0121.1</td><td>NbS00001525g0121.1 protein AED:0.18 eAED:0.18 QI:3|0.62|0.22|1|0.75|0.77|9|0|561</td></tr>
<tr><td>NbS00002899g0003.1</td><td>NbS00002899g0003.1 protein AED:0.26 eAED:0.26 QI:254|1|0.85|1|1|1|7|0|618; (*GB) gi|147770817|emb|CAN63166.1| (e_value=0.0) hypothetical protein</td></tr>
<tr><td>NbS00011076g0013.1</td><td>NbS00011076g0013.1 protein AED:0.25 eAED:0.25 QI:82|1|1|1|1|1|4|551|152; (*GB) gi|40287492|gb|AAR83860.1| (e_value=8e-97) putative ribosomal prot</td></tr>
<tr><td>NbS00002372g0015.1</td><td>NbS00002372g0015.1 protein AED:0.22 eAED:0.23 QI:207|0.66|0.5|1|1|1|4|0|452; (*GB) gi|110083391|dbj|BAE97400.1| (e_value=8e-141) heat shock prote</td></tr>
<tr><td>NbC24809697g0001.1</td><td>NbC24809697g0001.1 protein ; (*GB) gi|324388030|gb|ADY38792.1| (e_value=1e-59) hypothetical protein MA29G21.11 [Coffea arabica];; (*TAIR) AT5G2</td></tr>
<tr><td>NbS00002401g0015.1</td><td>NbS00002401g0015.1 protein AED:0.14 eAED:0.14 QI:244|0.81|0.82|1|0.77|0.78|28|441|989; (*SWP) sp|Q8RWY3|ISW2_ARATH (e_value=0.0) Putative chromat</td></tr>
<tr><td>NbS00045861g0008.1</td><td>NbS00045861g0008.1 protein AED:0.26 eAED:0.26 QI:38|1|0.91|1|0.54|0.75|12|0|334; (*GB) gi|225459625|ref|XP_002285874.1| (e_value=0.0) PREDICTED:</td></tr>
<tr><td>NbS00005183g0002.1</td><td>NbS00005183g0002.1 protein AED:0.18 eAED:0.18 QI:0|0.69|0.57|0.92|1|1|14|0|607; (*GB) gi|1762130|gb|AAB39827.1| (e_value=0.0) chaperonin-60 beta</td></tr>
<tr><td>gi|355708779|gb|AES03376.1|</td><td>gi|355708779|gb|AES03376.1| Group Os04g0486600 [Mustela putorius furo]</td></tr>
<tr><td>NbS00001900g0005.1</td><td>NbS00001900g0005.1 protein AED:0.34 eAED:0.34 QI:0|0|0|0.75|1|1|4|0|334; (*GB) gi|18000042|gb|AAL54878.1|AF004232_1 (e_value=2e-50) hydroxy-methy</td></tr>
<tr><td>NbS00011440g0007.1</td><td>NbS00011440g0007.1 protein AED:0.30 eAED:0.32 QI:0|0.87|0.55|1|0.87|0.88|9|0|608; (*TAIR) AT1G67840.1 (e_value=0.0) | Symbols: CSK | chloroplast</td></tr>
<tr><td>NbS00000172g0008.1</td><td>NbS00000172g0008.1 protein AED:0.09 eAED:0.09 QI:94|0.66|0.75|1|0.33|0|4|260|260; (*SWP) sp|P0C582|M2OM_NEUCR (e_value=7e-53) Putative mitochondr</td></tr>
<tr><td>NbS00016433g0017.1</td><td>NbS00016433g0017.1 protein AED:0.23 eAED:0.24 QI:195|1|0.9|1|1|1|10|0|371; (*GB) gi|255543841|ref|XP_002512983.1| (e_value=0.0) clathrin binding</td></tr>
<tr><td>NbS00007805g0017.1</td><td>NbS00007805g0017.1 protein AED:0.20 eAED:0.20 QI:0|1|0.6|1|1|1|5|0|406; (*GB) gi|255555933|ref|XP_002519002.1| (e_value=0.0) phosphoribulose kina</td></tr>
<tr><td>NbS00033277g0006.1</td><td>NbS00033277g0006.1 protein AED:0.56 eAED:0.56 QI:0|0|0|0.16|1|1|6|0|118</td></tr>
<tr><td>NbS00013334g0009.1</td><td>NbS00013334g0009.1 protein AED:0.05 eAED:0.05 QI:325|0.71|0.75|1|0.57|0.62|8|274|699; (*GB) gi|297742094|emb|CBI33881.3| (e_value=3e-155) unnamed</td></tr>
<tr><td>NbS00030026g0014.1</td><td>NbS00030026g0014.1 protein AED:0.18 eAED:0.23 QI:0|0.66|0.52|1|0.66|0.72|25|0|1122; (*GB) gi|68300799|gb|AAY89342.1| (e_value=0.0) RNA polymerase</td></tr>
<tr><td>NbS00000529g0002.1</td><td>NbS00000529g0002.1 protein AED:0.44 eAED:0.48 QI:0|0.66|0.5|1|1|1|4|0|104; (*GB) gi|224068340|ref|XP_002302713.1| (e_value=7e-69) predicted prote</td></tr>
<tr><td>NbS00001711g0013.1</td><td>NbS00001711g0013.1 protein AED:0.26 eAED:0.26 QI:422|0.85|0.87|1|0.85|0.75|8|401|334; (*TAIR) AT4G17520.1 (e_value=2e-31) | Symbols: | Hyalurona</td></tr>
<tr><td>NbS00009108g0010.1</td><td>NbS00009108g0010.1 protein AED:0.35 eAED:0.35 QI:142|0.5|0.33|1|1|0.66|3|0|414; (*GB) gi|255549002|ref|XP_002515557.1| (e_value=0.0) conserved hy</td></tr>
<tr><td>NbS00001513g0001.1</td><td>NbS00001513g0001.1 protein AED:0.13 eAED:0.14 QI:0|0|0|1|1|1|2|0|575; (*GB) gi|147792200|emb|CAN62034.1| (e_value=8e-171) hypothetical protein VI</td></tr>
<tr><td>NbS00034421g0001.1</td><td>NbS00034421g0001.1 protein AED:0.12 eAED:0.20 QI:0|0|0|1|1|1|3|0|591; (*GB) gi|225448505|ref|XP_002272996.1| (e_value=0.0) PREDICTED: U-box domai</td></tr>
<tr><td>NbS00000131g0001.1</td><td>NbS00000131g0001.1 protein AED:0.33 eAED:0.33 QI:158|1|1|1|1|1|2|235|224; (*GB) gi|3024505|sp|Q40522.1|RB11D_TOBAC (e_value=2e-152) RecName: Full</td></tr>
<tr><td>NbS00010015g0012.1</td><td>NbS00010015g0012.1 protein AED:0.30 eAED:0.33 QI:231|0.66|0.76|0.92|0.75|0.69|13|305|569; (*SWP) sp|Q9ZR40|U2A2B_NICPL (e_value=0.0) Splicing fac</td></tr>
</table>

NbS00053814g0004.1 NbS00053814g0004.1 protein AED:0.08 eAED:0.08 QI:0|0.71|0.75|0.87|0.85|0.75|8|0|460; (*GB) gi|359473220|ref|XP_003631270.1| (e_value=4e-107) PRED

NbS00042606g0008.1 NbS00042606g0008.1 protein AED:0.13 eAED:0.13 QI:139|1|0.75|1|0.66|0.75|4|0|562; (*GB) gi|359484596|ref|XP_002281155.2| (e_value=1e-144) PREDICTE

NbS00009748g0101.1 NbS00009748g0101.1 protein AED:0.12 eAED:0.12 QI:0|-1|0|1|-1|1|1|0|600

NbS00028472g0001.1 NbS00028472g0001.1 protein AED:0.09 eAED:0.09 QI:0|0.5|0|1|1|1|3|0|497; (*GB) gi|147858961|emb|CAN80826.1| (e_value=0.0) hypothetical protein VIT

NbS00001148g0004.1 NbS00001148g0004.1 protein AED:0.25 eAED:0.26 QI:0|0.66|0.5|1|0|0|4|455|319; (*GB) gi|226506550|ref|NP_001141544.1| (e_value=2e-158) uncharacteri

NbS00006130g0035.1 NbS00006130g0035.1 protein AED:0.02 eAED:0.02 QI:1363|1|1|1|0.75|0.4|5|684|545; (*GB) gi|555655|gb|AAA50196.1| (e_value=2e-123) DNA-binding prote

NbS00010382g0016.1 NbS00010382g0016.1 protein AED:0.27 eAED:0.27 QI:0|1|0.5|1|1|1|2|0|280; (*GB) gi|357521321|ref|XP_003630949.1| (e_value=9e-153) 40S ribosomal prc

NbS00010129g0001.1 NbS00010129g0001.1 protein AED:0.16 eAED:0.16 QI:0|0|0.5|1|1|1|2|131|318; (*SWP) sp|P23547|E13G_TOBAC (e_value=0.0) Glucan endo-1,3-beta-glucosid

NbS00007190g0001.1 NbS00007190g0001.1 protein AED:0.08 eAED:0.08 QI:179|1|1|1|0.5|0.33|3|443|275; (*GB) gi|225448932|ref|XP_002267178.1| (e_value=1e-93) PREDICTED:

NbS00001041g0113.1 NbS00001041g0113.1 protein AED:0.17 eAED:0.20 QI:0|1|0|1|1|1|2|0|462

NbS00011116g0014.1 NbS00011116g0014.1 protein AED:0.21 eAED:0.24 QI:14|0.5|0.4|1|0.75|0.8|5|1520|206; (*GB) gi|332038856|gb|ACX54788.2| (e_value=3e-47) putative arg

NbS00008510g0009.1 NbS00008510g0009.1 protein AED:0.00 eAED:0.00 QI:263|0.90|0.75|1|0.36|0.41|12|56|602; (*GB) gi|359476554|ref|XP_002267871.2| (e_value=0.0) PREDIC

NbS00016035g0004.1 NbS00016035g0004.1 protein AED:0.29 eAED:0.29 QI:177|0.8|0.66|1|0.6|0.33|6|0|468; (*GB) gi|133711805|gb|ABO36623.1| (e_value=0.0) putative RNA-bi

NbS00000215g0010.1 NbS00000215g0010.1 protein AED:0.11 eAED:0.11 QI:211|1|1|1|0.85|0.8|15|450|605; (*GB) gi|225442531|ref|XP_002284134.1| (e_value=0.0) PREDICTED: r

NbS00002703g0009.1 NbS00002703g0009.1 protein AED:0.34 eAED:0.35 QI:0|0.8|0.66|1|1|0.83|6|0|443; (*GB) gi|77745479|gb|ABB02638.1| (e_value=0.0) 26S proteasome subun

NbS00011278g0018.1 NbS00011278g0018.1 protein AED:0.22 eAED:0.22 QI:213|1|1|1|0.75|1|5|391|127; (*GB) gi|255548958|ref|XP_002515535.1| (e_value=4e-64) conserved hyp

NbS00004739g0001.1 NbS00004739g0001.1 protein AED:0.17 eAED:0.17 QI:0|1|0.66|1|1|1|3|0|251; (*GB) gi|100801744|emb|CAK24966.1| (e_value=2e-175) chlorophyll a/b bind

NbS00014198g0009.1 NbS00014198g0009.1 protein AED:0.34 eAED:0.34 QI:244|0.83|0.73|1|0.88|0.78|19|0|804; (*GB) gi|225463508|ref|XP_002263911.1| (e_value=0.0) PREDICT

NbS00002201g0002.1 NbS00002201g0002.1 protein AED:0.26 eAED:0.28 QI:0|0.9|0.72|1|1|1|11|0|453; (*GB) gi|356559428|ref|XP_003548001.1| (e_value=0.0) PREDICTED: V-typ

NbS00017034g0014.1 NbS00017034g0014.1 protein AED:0.20 eAED:0.20 QI:0|0.8|0.83|0.83|0.8|0.83|6|26|160; (*GB) gi|225454123|ref|XP_002269748.1| (e_value=9e-100) PREDI

NbS00028158g0015.1 NbS00028158g0015.1 protein AED:0.44 eAED:0.54 QI:0|0.75|0.4|1|1|1|5|0|169; (*GB) gi|388500696|gb|AFK38414.1| (e_value=1e-63) unknown [Lotus japon

NbS00005109g0003.1 NbS00005109g0003.1 protein AED:0.14 eAED:0.14 QI:170|1|0.5|1|0|0.5|2|0|256; (*GB) gi|40949663|gb|AAR97545.1| (e_value=8e-149) germin-like protein

NbS00001671g0002.1 NbS00001671g0002.1 protein ; (*GB) gi|77745458|gb|ABB02628.1| (e_value=3e-104) triose phosphate isomerase cytosolic isoform-like [Solanum tuber

NbS00019265g0001.1 NbS00019265g0001.1 protein AED:0.29 eAED:0.34 QI:99|0.6|0.66|1|1|1|6|0|422; (*GB) gi|115765|sp|P10708.1|CB12_SOLLC (e_value=3e-151) RecName: Full

NbS00005125g0015.1 NbS00005125g0015.1 protein AED:0.27 eAED:0.27 QI:600|0.91|0.84|1|0.83|0.69|13|286|356; (*GB) gi|304368145|gb|ADM26718.1| (e_value=0.0) glycolate

NbS00046932g0004.1 NbS00046932g0004.1 protein AED:0.40 eAED:0.41 QI:0|0.8|0.5|1|0.6|0.66|6|0|302; (*GB) gi|225441674|ref|XP_002282707.1| (e_value=1e-174) PREDICTED:

NbS00000884g0011.1 NbS00000884g0011.1 protein AED:0.32 eAED:0.32 QI:206|0.8|0.66|1|0.4|0.5|6|0|313; (*GB) gi|2500521|sp|Q40468.1|IF415_TOBAC (e_value=1e-151) RecNam

NbS00024271g0003.1 NbS00024271g0003.1 protein AED:0.06 eAED:0.06 QI:464|1|0.66|1|0|0.33|3|0|534; (*GB) gi|147882995|gb|ABQ51814.1| (e_value=0.0) RAN GTPase-activati

NbS00009678g0004.1 NbS00009678g0004.1 protein AED:0.24 eAED:0.23 QI:224|0.85|0.66|1|1|1|15|0|1042; (*GB) gi|359478503|ref|XP_002276796.2| (e_value=0.0) PREDICTED: p

NbS00029754g0004.1 NbS00029754g0004.1 protein AED:0.25 eAED:0.26 QI:246|0.4|0.36|1|0.9|0.81|11|0|636; (*GB) gi|357454461|ref|XP_003597511.1| (e_value=5e-50) hypothe

NbS00027428g0011.1 NbS00027428g0011.1 protein AED:0.20 eAED:0.20 QI:0|0|0|1|1|1|2|0|723; (*GB) gi|359475106|ref|XP_003631587.1| (e_value=0.0) PREDICTED: DEAD-box AT

NbS00002801g0002.1 NbS00002801g0002.1 protein

NbS00014581g0001.1 NbS00014581g0001.1 protein AED:0.17 eAED:0.17 QI:214|0.83|0.71|1|0.83|0.71|7|0|390; (*GB) gi|4827251|dbj|BAA77604.1| (e_value=0.0) plastidic aldc

NbS00020253g0010.1 NbS00020253g0010.1 protein AED:0.25 eAED:0.25 QI:0|0.85|0.5|1|0.85|0.75|8|0|669; (*GB) gi|296086337|emb|CBI31778.3| (e_value=0.0) unnamed protein

NbS00000058g0018.1 NbS00000058g0018.1 protein AED:0.02 eAED:0.02 QI:0|1|0|1|1|1|2|0|221; (*GB) gi|30013659|gb|AAP03872.1| (e_value=2e-130) putative photosystem I su

NbS00029951g0016.1 NbS00029951g0016.1 protein AED:0.12 eAED:0.12 QI:0|1|0.72|1|0.7|0.72|11|0|1065; (*GB) gi|297733757|emb|CBI15004.3| (e_value=0.0) unnamed protein

NbS00013165g0006.1 NbS00013165g0006.1 protein AED:0.11 eAED:0.11 QI:247|1|1|1|0.66|0.7|10|613|960; (*GB) gi|359496309|ref|XP_002264751.2| (e_value=0.0) PREDICTED: E

NbS00016832g0010.1 NbS00016832g0010.1 protein AED:0.10 eAED:0.11 QI:0|0.5|0.33|1|0.5|0.33|3|0|902; (*GB) gi|255544686|ref|XP_002513404.1| (e_value=0.0) eukaryotic t

NbS00017365g0003.1 NbS00017365g0003.1 protein AED:0.22 eAED:0.22 QI:0|1|0.6|1|1|1|5|0|481; (*GB) gi|255561431|ref|XP_002521726.1| (e_value=0.0) srpk, putative [Rici

NbS00034416g0005.1 NbS00034416g0005.1 protein AED:0.15 eAED:0.48 QI:0|0|0|1|0|0|5|0|377; (*GB) gi|312370165|gb|ADQ74440.1| (e_value=5e-71) ribulose-1,5-bisphosphate

NbS00049664g0004.1 NbS00049664g0004.1 protein AED:0.29 eAED:0.29 QI:0|0.6|0.33|0.66|1|0.83|6|0|236; (*GB) gi|584795|sp|Q08436.1|PMA3_NICPL (e_value=1e-107) RecName:

NbS00040143g0002.1 NbS00040143g0002.1 protein AED:0.18 eAED:0.18 QI:0|0|0|0.75|0|0.25|4|0|319; (*GB) gi|11465942|ref|NP_054484.1| (e_value=1e-124) ATP synthase CF0

NbS00014198g0003.1 NbS00014198g0003.1 protein

NbS00026008g0003.1 NbS00026008g0003.1 protein AED:0.26 eAED:0.26 QI:130|1|1|1|1|1|6|514|394; (*GB) gi|164459314|gb|ABY57764.1| (e_value=0.0) extracellular Ca2+ sens

NbS00003479g0020.1 NbS00003479g0020.1 protein AED:0.28 eAED:0.28 QI:190|1|1|1|0.75|0.6|5|184|363; (*GB) gi|396589|emb|CAA52461.1| (e_value=0.0) catechol O-methyltra

NbS00022724g0010.1 NbS00022724g0010.1 protein AED:0.41 eAED:0.41 QI:0|0.83|0.71|1|0.66|0.71|7|141|139; (*GB) gi|363808256|ref|NP_001242493.1| (e_value=4e-92) unchar

NbS00033933g0011.1 NbS00033933g0011.1 protein AED:0.39 eAED:0.39 QI:0|0|0|1|0|0|3|0|181; (*GB) gi|113205233|gb|AAT39304.2| (e_value=1e-15) Transposon MuDR mudrA-lik

NbS00012538g0018.1 NbS00012538g0018.1 protein AED:0.32 eAED:0.32 QI:0|0|0|1|0|0|5|0|257; (*GB) gi|357470629|ref|XP_003605599.1| (e_value=9e-89) Apocytochrome f [Med

NbS00006841g0003.1 NbS00006841g0003.1 protein AED:0.24 eAED:0.24 QI:284|1|1|1|0.66|0.75|4|120|287; (*GB) gi|115291793|gb|ABI93215.1| (e_value=0.0) water channel prc

NbS00001471g0009.1 NbS00001471g0009.1 protein AED:0.21 eAED:0.22 QI:0|0.87|0.77|1|1|1|9|428|607; (*GB) gi|356555871|ref|XP_003546253.1| (e_value=0.0) PREDICTED: lys

NbS00021709g0005.1 NbS00021709g0005.1 protein AED:0.27 eAED:0.27 QI:208|0.75|0.88|1|0.5|0.44|9|304|415; (*GB) gi|225435464|ref|XP_002285466.1| (e_value=0.0) PREDICT

NbS00054324g0011.1 NbS00054324g0011.1 protein AED:0.09 eAED:0.09 QI:199|0.88|0.81|1|0.65|0.59|27|513|1505; (*GB) gi|296087564|emb|CBI34153.3| (e_value=0.0) unnamed

NbS00018164g0006.1 NbS00018164g0006.1 protein ; (*GB) gi|297738947|emb|CBI28192.3| (e_value=5e-127) unnamed protein product [Vitis vinifera];; (*TAIR) AT5G51200.1

NbS00012577g0009.1 NbS00012577g0009.1 protein AED:0.13 eAED:0.13 QI:321|1|1|1|0.9|0.81|11|196|765; (*SWP) sp|Q42699|METE_CATRO (e_value=0.0) 5-methyltetrahydropterc

NbS00019818g0001.1 NbS00019818g0001.1 protein AED:0.28 eAED:0.28 QI:0|1|0.5|1|1|1|2|0|332; (*GB) gi|384038815|gb|AFH57998.1| (e_value=0.0) chloroplast PsbO2 precurs

NbS00002113g0001.1 NbS00002113g0001.1 protein AED:0.00 eAED:0.05 QI:0|-1|0|1|-1|1|1|0|394; (*SWP) sp|Q00874|DR100_ARATH (e_value=1e-127) DNA-damage-repair/toleratic

NbS00016451g0014.1 NbS00016451g0014.1 protein AED:0.11 eAED:0.11 QI:268|0.94|0.88|1|0.88|0.83|18|330|1024; (*GB) gi|359487657|ref|XP_002278468.2| (e_value=0.0) PRED

NbS00002808g0033.1 NbS00002808g0033.1 protein AED:0.14 eAED:0.14 QI:0|1|0.94|1|0.88|0.94|18|425|576; (*GB) gi|225433255|ref|XP_002285452.1| (e_value=0.0) PREDICTED:

NbS00006379g0022.1 NbS00006379g0022.1 protein AED:0.09 eAED:0.09 QI:0|0.75|0.8|0.8|1|1|5|273|133; (*GB) gi|76161008|gb|ABA40467.1| (e_value=5e-73) glycoprotein-like

NbS00035965g0001.1 NbS00035965g0001.1 protein AED:0.18 eAED:0.19 QI:0|0|0|0.33|1|1|3|0|161; (*GB) gi|18394828|ref|NP_564105.1| (e_value=2e-19) S-phase kinase-associ
NbS00011397g0001.1 NbS00011397g0001.1 protein AED:0.61 eAED:0.61 QI:0|0|0|0.5|1|1|2|0|130; (*SWP) sp|A1XQR9|RUXE_PIG (e_value=3e-31) Small nuclear ribonucleoprotein
NbS00020307g0016.1 NbS00020307g0016.1 protein AED:0.13 eAED:0.13 QI:0|0.33|0.25|0.75|0.66|0.25|4|0|741; (*GB) gi|75249421|sp|Q93YF5.1|SUVH1_TOBAC (e_value=0.0) RecN
NbS00027551g0008.1 NbS00027551g0008.1 protein AED:0.09 eAED:0.10 QI:319|0.53|0.48|1|0.84|0.81|27|401|2351; (*GB) gi|359482368|ref|XP_003632762.1| (e_value=0.0) PRED
NbS00029120g0005.1 NbS00029120g0005.1 protein AED:0.30 eAED:0.35 QI:0|0.4|0.16|1|1|1|6|0|502; (*GB) gi|225442557|ref|XP_002284272.1| (e_value=0.0) PREDICTED: unchar
NbS00004321g0109.1 NbS00004321g0109.1 protein AED:0.14 eAED:0.14 QI:204|1|0.66|1|1|1|3|255|287
NbS00000561g0021.1 NbS00000561g0021.1 protein AED:0.23 eAED:0.34 QI:0|0|0|1|0.2|0.16|6|0|324; (*GB) gi|19911171|dbj|BAB86912.1| (e_value=5e-70) putative cytochrome
NbS00008412g0009.1 NbS00008412g0009.1 protein AED:0.13 eAED:0.13 QI:0|0.75|0.55|1|0.75|0.55|9|0|802; (*GB) gi|194396261|gb|ACF60500.1| (e_value=0.0) plastid transke
NbS00018705g0023.1 NbS00018705g0023.1 protein AED:0.30 eAED:0.31 QI:0|0.66|0.75|1|0.33|0.75|4|388|71; (*GB) gi|242050850|ref|XP_002463169.1| (e_value=7e-39) hypothe
NbS00006168g0008.1 NbS00006168g0008.1 protein AED:0.11 eAED:0.11 QI:168|0|0|1|0|0|2|0|473; (*SWP) sp|Q9LHE3|ASPG2_ARATH (e_value=6e-108) Protein ASPARTIC PROTEASE I
NbS00005023g0005.1 NbS00005023g0005.1 protein AED:0.11 eAED:0.11 QI:134|1|1|1|0.8|0.83|6|218|290; (*GB) gi|71370259|gb|AAZ30377.1| (e_value=0.0) PHB2 [Nicotiana ben
NbS00001184g0009.1 NbS00001184g0009.1 protein AED:0.24 eAED:0.25 QI:0|0.25|0.2|1|1|1|5|0|443; (*GB) gi|17402469|emb|CAD13177.1| (e_value=0.0) alpha-tubulin [Nicotia
NbS00059407g0001.1 NbS00059407g0001.1 protein AED:0.31 eAED:0.31 QI:0|0|0|1|1|1|3|0|138; (*SWP) sp|P06005|PSBD_SPIOL (e_value=4e-44) Photosystem II D2 protein OS=Sp
NbS00017399g0001.1 NbS00017399g0001.1 protein AED:0.12 eAED:0.12 QI:0|0.75|0.2|1|1|1|5|0|372; (*GB) gi|399212|sp|P31541.1|CLPAA_SOLLC (e_value=0.0) RecName: Full=AT
NbS00005732g0004.1 NbS00005732g0004.1 protein AED:0.15 eAED:0.15 QI:0|0.2|0|0.83|1|1|6|0|478; (*GB) gi|255543963|ref|XP_002513044.1| (e_value=3e-173) Aspartic prote
NbS00006675g0003.1 NbS00006675g0003.1 protein AED:0.09 eAED:0.09 QI:0|0|0|0.66|1|1|3|416|592; (*GB) gi|225470260|ref|XP_002264285.1| (e_value=0.0) PREDICTED: U3 sma
NbS00043662g0002.1 NbS00043662g0002.1 protein ; (*GB) gi|296089862|emb|CBI39681.3| (e_value=5e-45) unnamed protein product [Vitis vinifera];; (*SWP) sp|Q8VYE4|PTR
NbS00027742g0013.1 NbS00027742g0013.1 protein AED:0.00 eAED:0.01 QI:0|1|1|1|1|1|7|309|363; (*GB) gi|77416977|gb|ABA81884.1| (e_value=4e-80) nuclear RNA binding prot
NbS00005060g0011.1 NbS00005060g0011.1 protein AED:0.28 eAED:0.49 QI:0|0|0|1|0|0|2|0|110; (*GB) gi|356525523|ref|XP_003531374.1| (e_value=1e-24) PREDICTED: cytochrom
NbS00009355g0003.1 NbS00009355g0003.1 protein AED:0.19 eAED:0.19 QI:0|-1|0|1|-1|1|1|0|585; (*GB) gi|307135979|gb|ADN33838.1| (e_value=0.0) pre-mRNA splicing factor
NbS00046532g0005.1 NbS00046532g0005.1 protein AED:0.23 eAED:0.23 QI:236|1|1|1|1|1|10|251|775; (*GB) gi|224116492|ref|XP_002317314.1| (e_value=0.0) predicted protein
NbS00000211g0112.1 NbS00000211g0112.1 protein AED:0.16 eAED:0.16 QI:0|0.88|0.8|1|0.88|0.8|10|0|389
NbS00056997g0003.1 NbS00056997g0003.1 protein AED:0.27 eAED:0.27 QI:218|1|1|1|1|1|3|277|931; (*GB) gi|8919178|emb|CAB96077.1| (e_value=0.0) alpha-glucosidase [Solan
NbS00020649g0003.1 NbS00020649g0003.1 protein AED:0.03 eAED:0.03 QI:46|1|1|1|1|1|3|317|655; (*SWP) sp|P09114|ILVB2_TOBAC (e_value=0.0) Acetolactate synthase 2, chlc
NbS00040938g0004.1 NbS00040938g0004.1 protein AED:0.11 eAED:0.11 QI:0|0|0|1|0.5|0.66|3|0|334; (*TAIR) AT4G29310.1 (e_value=2e-132) | Symbols: | Protein of unknown
NbS00007750g0014.1 NbS00007750g0014.1 protein AED:0.40 eAED:0.40 QI:0|0.75|0.6|1|0.75|0.6|5|0|199; (*GB) gi|351723391|ref|NP_001237789.1| (e_value=4e-117) uncharact
NbS00000250g0010.1 NbS00000250g0010.1 protein AED:0.23 eAED:0.23 QI:0|0.83|0.78|0.89|0.77|0.78|19|437|448; (*GB) gi|238814974|gb|ACR56690.1| (e_value=0.0) enolase [
NbS00002556g0019.1 NbS00002556g0019.1 protein AED:0.14 eAED:0.14 QI:0|1|0.33|1|1|1|3|0|298; (*SWP) sp|Q9FL12|DEGP9_ARATH (e_value=1e-127) Protease Do-like 9 OS=Arab
NbS00016013g0004.1 NbS00016013g0004.1 protein AED:0.27 eAED:0.27 QI:0|1|0.66|1|1|0.66|3|0|319; (*GB) gi|255555327|ref|XP_002518700.1| (e_value=0.0) DNA-directed RNA
NbS00005419g0006.1 NbS00005419g0006.1 protein
NbS00004972g0108.1 NbS00004972g0108.1 protein AED:0.17 eAED:0.17 QI:133|0|0.5|1|1|1|2|0|460
NbS00006241g0007.1 NbS00006241g0007.1 protein AED:0.11 eAED:0.19 QI:0|0.5|0.33|1|0|0|3|31|414; (*GB) gi|225470070|ref|XP_002269458.1| (e_value=1e-33) PREDICTED: unc
NbS00010046g0020.1 NbS00010046g0020.1 protein AED:0.25 eAED:0.25 QI:0|0.76|0.66|0.94|0.64|0.66|18|0|588; (*GB) gi|171854667|dbj|BAG16523.1| (e_value=0.0) putative N
NbS00001581g0012.1 NbS00001581g0012.1 protein AED:0.09 eAED:0.10 QI:0|1|0.6|1|0.75|0.8|5|0|220; (*GB) gi|242044476|ref|XP_002460109.1| (e_value=3e-117) hypothetical

Select Summary Report

Format As [Select Summary (protein hits)] Help

Significance threshold p< 0.05 Max. number of hits AUTO

Standard scoring ○ MudPIT scoring ◉ Ions score or expect cut-off 30 Show sub-sets 0

Show pop-ups ◉ Suppress pop-ups ○ Require bold red ☐

Re-Search ◉ All queries ○ Unassigned ○ Below homology threshold ○ Below identity threshold

1. NbS00023525g0004.1 **Mass:** 15039 **Score:** 679 **Matches:** 33(32) **Sequences:** 8(8) **emPAI:** 10.63

NbS00023525g0004.1 protein AED:0.27 eAED:0.28 QI:0|0|0|0.5|1|1|2|0|137; (*GB) gi|356576357|ref|XP_003556299.1| (e_value=4e-60) PREDICTED: probable histone H2B.1-like [Glycine max];; (*SWP) sp|Q1S9:

Query	Observed	Mr(expt)	Mr(calc)	ppm	Miss	Score	Expect	Rank	Unique	Peptide
8662	342.2093	682.4040	682.4054	-1.98	0	31	0.018	3	U	K.IYIFK.V
9248	395.7066	789.3987	789.3981	0.84	0	42	0.0067	1	U	K.LAQESSR.L 9247
9413	408.7324	815.4502	815.4501	0.08	0	34	0.048	1	U	R.EIQTAVR.L
9495	414.7142	827.4139	827.4137	0.23	0	39	0.0052	1	U	K.HAVSEGTK.A
10285	470.2971	938.5796	938.5800	-0.45	0	59	2.2e-005	1	U	R.LVLPGELAK.H
12247	394.2150	1179.6231	1179.6248	-1.45	0	48	0.001	1	U	K.QVHPDIGISSK.A 12253
12250	590.8193	1179.6240	1179.6248	-0.67	0	(33)	0.031	1	U	K.QVHPDIGISSK.A
17526	865.4164	1728.8182	1728.8215	-1.89	0	96	1.4e-008	1	U	K.AMGIMNSFINDIFEK.L 17527
17619	873.4161	1744.8177	1744.8164	0.76	0	(39)	0.0068	1	U	K.AMGIMNSFINDIFEK.L 17606 17617 17631

17636	873.4166	1744.8187	1744.8164	1.31	0	(67)	9.4e-006	1	U	K.AMGIMNSFINDIFEK.L 17615 17622 17624 17626 17628 17629 17637 17638 17639 17641 17647
17751	881.4135	1760.8125	1760.8113	0.65	0	(71)	3.8e-006	1	U	K.AMGIMNSFINDIFEK.L 17745 17747 17748
20595	834.4117	2500.2132	2500.2090	1.69	1	(41)	0.0034	1	U	K.AMGIMNSFINDIFEKLAQESSR.L
20623	839.7422	2516.2047	2516.2039	0.33	1	47	0.00083	1	U	K.AMGIMNSFINDIFEKLAQESSR.L

Proteins matching the same set of peptides:

NbC24219583g0001.1 **Mass:** 11803 **Score:** 679 **Matches:** 33(32) **Sequences:** 8(8)
NbC24219583g0001.1 protein AED:0.00 eAED:0.00 QI:0|-1|0|1|-1|1|1|0|107; (*GB) gi|82400146|gb|ABB72812.1| (e_value=1e-61) histone H2B-like protein [Solanum tuberosum];; (*SWP) sp|Q1S9I9|H2B1_MEDTR

NbS00000666g0001.1 **Mass:** 15719 **Score:** 679 **Matches:** 33(32) **Sequences:** 8(8)
NbS00000666g0001.1 protein AED:0.15 eAED:0.15 QI:0|-1|0|1|-1|1|1|0|145; (*GB) gi|82400146|gb|ABB72812.1| (e_value=5e-61) histone H2B-like protein [Solanum tuberosum];; (*SWP) sp|Q1S9I9|H2B1_MEDTR

NbS00000678g0001.1 **Mass:** 15849 **Score:** 679 **Matches:** 33(32) **Sequences:** 8(8)
NbS00000678g0001.1 protein AED:0.22 eAED:0.22 QI:0|-1|0|1|-1|1|1|0|147; (*GB) gi|82400146|gb|ABB72812.1| (e_value=5e-61) histone H2B-like protein [Solanum tuberosum];; (*SWP) sp|Q1S9I9|H2B1_MEDTR

NbS00000678g0002.1 **Mass:** 15863 **Score:** 679 **Matches:** 33(32) **Sequences:** 8(8)
NbS00000678g0002.1 protein AED:0.22 eAED:0.22 QI:0|-1|0|1|-1|1|1|0|147; (*GB) gi|82400146|gb|ABB72812.1| (e_value=5e-61) histone H2B-like protein [Solanum tuberosum];; (*SWP) sp|Q1S9I9|H2B1_MEDTR

NbS00000678g0009.1 **Mass:** 16874 **Score:** 679 **Matches:** 33(32) **Sequences:** 8(8)
NbS00000678g0009.1 protein AED:0.29 eAED:0.29 QI:0|0|0|0.5|1|1|2|0|156; (*GB) gi|388514209|gb|AFK45166.1| (e_value=1e-55) unknown [Lotus japonicus];; (*SWP) sp|Q1SU99|H2B3_MEDTR (e_value=5e-57) Pro

NbS00006411g0002.1 **Mass:** 15863 **Score:** 679 **Matches:** 33(32) **Sequences:** 8(8)
NbS00006411g0002.1 protein AED:0.22 eAED:0.22 QI:0|-1|0|1|-1|1|1|0|147; (*GB) gi|82400146|gb|ABB72812.1| (e_value=5e-61) histone H2B-like protein [Solanum tuberosum];; (*SWP) sp|Q1S9I9|H2B1_MEDTR

NbS00007991g0006.1 **Mass:** 17158 **Score:** 679 **Matches:** 33(32) **Sequences:** 8(8)
NbS00007991g0006.1 protein AED:0.08 eAED:0.08 QI:0|0|0|1|1|1|2|0|157; (*GB) gi|146336941|gb|ABQ23584.1| (e_value=2e-57) putative histone [Medicago truncatula];; (*SWP) sp|Q1S9I9|H2B1_MEDTR (e_value

NbS00008452g0101.1 **Mass:** 15935 **Score:** 679 **Matches:** 33(32) **Sequences:** 8(8)
NbS00008452g0101.1 protein AED:0.16 eAED:0.16 QI:0|1|0.5|1|0|0|2|347|146

NbS00017233g0003.1 **Mass:** 16025 **Score:** 679 **Matches:** 33(32) **Sequences:** 8(8)
NbS00017233g0003.1 protein AED:0.20 eAED:0.20 QI:0|-1|0|1|-1|1|1|0|146; (*SWP) sp|P93354|H2B_TOBAC (e_value=4e-62) Histone H2B OS=Nicotiana tabacum GN=HIS2B PE=2 SV=3;; (*TAIR) AT5G59910.1 (e_value

NbS00020161g0001.1 **Mass:** 16125 **Score:** 679 **Matches:** 33(32) **Sequences:** 8(8)
NbS00020161g0001.1 protein AED:0.20 eAED:0.20 QI:0|-1|0|1|-1|1|1|0|147; (*GB) gi|82400146|gb|ABB72812.1| (e_value=3e-60) histone H2B-like protein [Solanum tuberosum];; (*SWP) sp|Q1S9I9|H2B1_MEDTR

NbS00027179g0002.1 **Mass:** 15949 **Score:** 679 **Matches:** 33(32) **Sequences:** 8(8)
NbS00027179g0002.1 protein AED:0.27 eAED:0.27 QI:0|-1|0|1|-1|1|1|0|146; (*GB) gi|388522051|gb|AFK49087.1| (e_value=1e-60) unknown [Lotus japonicus];; (*SWP) sp|Q1S9I9|H2B1_MEDTR (e_value=6e-62) Pro

NbS00028800g0003.1 **Mass:** 16210 **Score:** 679 **Matches:** 33(32) **Sequences:** 8(8)
NbS00028800g0003.1 protein AED:0.16 eAED:0.16 QI:0|-1|0|1|-1|1|1|0|148; (*GB) gi|118484226|gb|ABK93993.1| (e_value=3e-60) unknown [Populus trichocarpa];; (*SWP) sp|Q1S9I9|H2B1_MEDTR (e_value=1e-61)

NbS00032978g0007.1 **Mass:** 15457 **Score:** 679 **Matches:** 33(32) **Sequences:** 8(8)
NbS00032978g0007.1 protein AED:0.01 eAED:0.01 QI:0|-1|0|1|-1|1|1|0|141; (*GB) gi|82400146|gb|ABB72812.1| (e_value=2e-60) histone H2B-like protein [Solanum tuberosum];; (*SWP) sp|Q1S9I9|H2B1_MEDTR

NbS00037466g0010.1 **Mass:** 20264 **Score:** 679 **Matches:** 33(32) **Sequences:** 8(8)
NbS00037466g0010.1 protein AED:0.02 eAED:0.03 QI:0|0|0|1|1|1|2|0|185; (*GB) gi|388514209|gb|AFK45166.1| (e_value=3e-60) unknown [Lotus japonicus];; (*SWP) sp|Q1S9I9|H2B1_MEDTR (e_value=2e-61) Proba

---

2. NbS00031648g0011.1 **Mass:** 99402 **Score:** 503 **Matches:** 29(28) **Sequences:** 22(22) **emPAI:** 1.11
NbS00031648g0011.1 protein AED:0.23 eAED:0.23 QI:280|1|1|1|0.95|0.91|23|300|916; (*GB) gi|225465253|ref|XP_002268311.1| (e_value=0.0) PREDICTED: dynamin-2B [Vitis vinifera];; (*SWP) sp|Q9LQ55|DRP2I

Query	Observed	Mr(expt)	Mr(calc)	ppm	Miss	Score	Expect	Rank	Unique	Peptide
9224	394.7368	787.4590	787.4592	-0.25	0	35	0.026	1	U	K.APNLVFK.I
10557	485.2841	968.5536	968.5542	-0.64	0	35	0.012	1	U	K.LIDLPGVDK.G
10679	493.7562	985.4979	985.4981	-0.23	0	32	0.035	1	U	R.HFDINNVK.R
10788	501.7953	1001.5760	1001.5757	0.32	0	43	0.0039	1	U	K.SIVLQIDSK.S 10789
11876	570.3427	1138.6709	1138.6710	-0.10	0	41	0.0019	1	U	R.LPNLLSGLQGK.S 11879
12058	581.8027	1161.5909	1161.5886	2.00	0	63	4.7e-005	1	U	K.MVVALVDMER.V 12057
12159	586.8173	1171.6201	1171.6197	0.33	0	41	0.0052	1	U	K.SQVVQDELVR.L 12158
12753	620.3198	1238.6250	1238.6255	-0.41	0	38	0.012	1	U	R.RPVDPEEELR.W
12891	628.3302	1254.6458	1254.6456	0.23	0	38	0.01	1	U	K.LYSSISAQSTAK.I
14056	682.3300	1362.6455	1362.6449	0.42	0	49	0.00083	1	U	R.LGEQMVNSAEGTK.A 14058
14086	683.8420	1365.6695	1365.6677	1.32	0	35	0.022	1	U	K.VVTSFEGNFPNR.I
14268	695.8845	1389.7544	1389.7504	2.88	0	34	0.027	1	U	R.EVVAIASAALDGFK.T
14775	725.8502	1449.6858	1449.6848	0.67	0	54	0.00026	1	U	R.SAFDAAANGPSSLSR.Y
14814	729.3777	1456.7409	1456.7409	0.02	0	39	0.0091	1	U	K.IEELLQEDQNVK.R
16030	789.9294	1577.8443	1577.8413	1.90	0	33	0.024	1	U	R.SSTFLNVVAIGGTGAGK.S
16537	807.4300	1612.8454	1612.8420	2.12	1	40	0.0054	1	U	K.IEELLQEDQNVKR.R
16916	548.9699	1643.8879	1643.8882	-0.21	0	52	0.00031	1	U	R.GYVEAVLNSLAANVPK.A
17578	870.4871	1738.9597	1738.9577	1.12	0	38	0.0058	1	U	R.VLVDLVSSAANATPGLGR.Y
17771	881.9219	1761.8292	1761.8268	1.35	0	38	0.01	1	U	K.QAAALLADEDVDETSSK.R

18608	640.3171	1917.9294	1917.9279	0.77	1	(56)	0.00015	1	U	K.QAAALLADEDVDETSSKR.S
18609	959.9720	1917.9294	1917.9279	0.77	1	60	5.3e-005	1	U	K.QAAALLADEDVDETSSKR.S
18705	968.0101	1934.0057	1934.0037	1.06	0	40	0.0054	1	U	R.IVLEADGYQPYLISPEK.G 18703
19464	702.0441	2103.1106	2103.1073	1.56	0	62	2.5e-005	1	U	K.SAVLNSLVGHPALPTGEGGATR.A

3. gi|297262447|ref|XP_001098182.2| **Mass:** 65341 **Score:** 439 **Matches:** 21(20) **Sequences:** 12(12) **emPAI:** 0.80

gi|297262447|ref|XP_001098182.2| PREDICTED: keratin, type II cytoskeletal 1-like isoform 6 [Macaca mulatta]

Query	Observed	Mr(expt)	Mr(calc)	ppm	Miss	Score	Expect	Rank	Unique	Peptide
9527	416.7478	831.4811	831.4814	-0.42	0	39	0.011	1	U	K.SISISVAR.G 9529
9752	437.7529	873.4912	873.4920	-0.90	0	38	0.015	1	U	R.SLVNLGGSK.S
10588	487.2690	972.5235	972.5240	-0.45	0	58	0.00014	1		K.IEISELNR.V 10590
12232	590.3032	1178.5919	1178.5931	-1.04	0	85	2.4e-007	1	U	K.YEELQITAGR.H 12231 12234
13120	639.3583	1276.7020	1276.7027	-0.52	0	42	0.0036	1	U	K.LALDLEIATYR.T 13119 13126
13454	651.8615	1301.7085	1301.7078	0.50	0	85	2.2e-007	1	U	R.SLDLDSIIAEVK.A 13458
13983	679.3519	1356.6893	1356.6885	0.61	0	37	0.014	1	U	K.LNDLEDALQQAK.E
14203	692.3500	1382.6854	1382.6830	1.76	0	34	0.023	1	U	K.SLNNQFASFIDK.V
14298	697.3707	1392.7268	1392.7249	1.37	1	31	0.049	1	U	R.TNAENEFVTIKK.D
14970	738.3790	1474.7434	1474.7416	1.20	0	70	7.3e-006	1	U	K.WELLQQVDTSTR.T 14968
14973	738.3962	1474.7778	1474.7780	-0.12	0	69	9.6e-006	1		R.FLEQQNQVLQTK.W 14975
19122	665.3309	1992.9710	1992.9693	0.82	0	35	0.018	1	U	R.THNLEPYFESFINNLR.R

4. NbS00021492g0001.1 **Mass:** 72107 **Score:** 314 **Matches:** 16(16) **Sequences:** 11(11) **emPAI:** 0.63

NbS00021492g0001.1 protein AED:0.22 eAED:0.25 QI:0|0.70|0.72|0.94|0.94|0.88|18|255|639; (*SWP) sp|Q39828|SDL5A_SOYBN (e_value=0.0) Dynamin-related protein 5A OS=Glycine max PE=2 SV=1;; (*TAIR) AT5[illegible]

Query	Observed	Mr(expt)	Mr(calc)	ppm	Miss	Score	Expect	Rank	Unique	Peptide
10173	463.7404	925.4662	925.4658	0.48	0	51	0.00043	1	U	R.FTDFAAVR.K
7448	509.7900	1017.5654	1017.5706	-5.08	0	41	0.007	1	U	K.SSVLESIVGK.D
11410	541.8327	1081.6509	1081.6495	1.23	0	41	0.0016	1		R.IPGLQSLINK.T
11803	377.9085	1130.7038	1130.7036	0.14	0	35	0.0048	1		R.RPLVLQLHR.L
13593	658.8572	1315.6998	1315.6983	1.14	0	55	0.00024	1	U	K.TIIELENELSR.L 13591 13592 13594
13724	664.8782	1327.7419	1327.7401	1.37	0	54	0.00018	1		K.LQFPWIGVVNR.S 13723
13860	673.3418	1344.6690	1344.6674	1.25	0	50	0.00077	1	U	R.AAQGEIDSVAWAK.-
14120	686.8573	1371.7000	1371.6994	0.48	0	47	0.0013	1	U	R.VEVNGAAVESLER.M
17335	850.4393	1698.8640	1698.8611	1.73	0	35	0.017	2	U	K.QLGTLLDEDPAIMQR.R 17334
18250	620.9844	1859.9313	1859.9299	0.77	1	37	0.013	1	U	K.IDLMDKGTDAVDILEGR.A
19256	1023.5138	2045.0130	2045.0099	1.53	0	41	0.0038	1	U	K.VAVEGQSESIVADIENMVR.S

5. gi|28317|emb|CAA32649.1| **Mass:** 59720 **Score:** 310 **Matches:** 16(16) **Sequences:** 12(12) **emPAI:** 0.90

gi|28317|emb|CAA32649.1| unnamed protein product [Homo sapiens]

Query	Observed	Mr(expt)	Mr(calc)	ppm	Miss	Score	Expect	Rank	Unique	Peptide
9333	404.2033	806.3920	806.3923	-0.31	0	41	0.0074	1	U	R.LAADDFR.L 9334
11468	545.7685	1089.5224	1089.5237	-1.14	0	32	0.044	1	U	K.VTMQNLNDR.L
11613	555.2488	1108.4831	1108.4825	0.55	0	41	0.0034	1	U	K.DAEAWFNEK.S
12096	583.2958	1164.5771	1164.5775	-0.30	0	40	0.0089	1	U	R.LENEIQTYR.S
8154	594.3500	1186.6854	1186.6921	-5.62	1	32	0.055	1	U	R.RVLDELTLTK.A
12961	631.8026	1261.5907	1261.5899	0.64	0	57	0.00013	1	U	R.SLLEGEGSSGGGGR.G 12960
14079	683.3240	1364.6335	1364.6320	1.10	0	40	0.0058	1	U	R.SQYEQLAEQNR.K
14185	691.3292	1380.6438	1380.6408	2.13	0	60	5.4e-005	1	U	R.ALEESNYELEGK.I
14266	695.8453	1389.6761	1389.6736	1.85	0	65	2.3e-005	1	U	K.QSLEASLAETEGR.Y 14265
17386	854.3906	1706.7667	1706.7649	1.05	0	53	0.0002	1	U	K.GSLGGGFSSGGFSGGSFSR.G 17384
19139	998.9832	1995.9518	1995.9636	-5.95	0	36	0.014	1	U	K.ELTTEIDNNIEQISSYK.S
21179	1018.2136	3051.6190	3051.6200	-0.31	1	50	0.00022	1	U	K.TIDDLKNQILNLTTDNANILLQIDNAR.L

6. NbS00056353g0008.1 **Mass:** 62335 **Score:** 291 **Matches:** 16(16) **Sequences:** 13(13) **emPAI:** 1.05

NbS00056353g0008.1 protein AED:0.25 eAED:0.25 QI:0|0.92|0.85|1|0.92|0.92|14|0|554; (*SWP) sp|Q9FNX5|DRP1E_ARATH (e_value=0.0) Dynamin-related protein 1E OS=Arabidopsis thaliana GN=DRP1E PE=1 SV=1;;

Query	Observed	Mr(expt)	Mr(calc)	ppm	Miss	Score	Expect	Rank	Unique	Peptide

Query	Observed	Mr(expt)	Mr(calc)	ppm	Miss	Score	Expect	Rank	Unique	Peptide
10083	457.7586	913.5027	913.5021	0.57	0	35	0.02	1		K.HLEQFIK.A
10412	477.7555	953.4964	953.4971	-0.75	0	52	0.00041	1		R.FTDFAVVR.K
11122	523.7946	1045.5746	1045.5768	-2.11	0	35	0.027	1		K.SSVLESIVGR.D
11430	543.3325	1084.6504	1084.6492	1.09	0	39	0.0044	1		R.IPSITSLINK.S 11428
12330	595.2942	1188.5738	1188.5775	-3.09	0	32	0.044	1	U	R.DEIDSVAWVR.-
13614	440.2511	1317.7316	1317.7306	0.73	0	38	0.0087	1		R.LQHPWVGIVNR.S
16008	788.9283	1575.8421	1575.8409	0.78	0	56	0.00016	1		R.IYGVFDNQLPAALR.K
17252	843.4251	1684.8357	1684.8341	0.91	0	66	1.6e-005	1	U	K.ALAELLDEDPALMER.R
18151	921.4585	1840.9024	1840.8989	1.92	0	47	0.0013	1		R.IGSNVSSYVNMVSDTLR.N
18230	929.4543	1856.8940	1856.8938	0.10	0	(38)	0.0094	1		R.IGSNVSSYVNMVSDTLR.N
18245	620.6559	1858.9458	1858.9458	-0.01	1	37	0.01	1		K.LDLMDKGTNALDVLEGR.A 18246
18339	938.9868	1875.9590	1875.9578	0.64	0	35	0.017	1		R.FPSLQSTIAAASNEALEK.F
19459	1052.5187	2103.0228	2103.0154	3.52	0	46	0.0011	1		K.VAVDGQSETIVEDIENMVR.T
21264	1047.9334	3140.7782	3140.7849	-2.13	0	55	2.4e-005	1		K.QISPVPIHLSIYSPNVVNLTLIDLPGLTK.V

7. NbS00008178g0018.1 **Mass:** 15524 **Score:** 276 **Matches:** 12(12) **Sequences:** 4(4) **emPAI:** 1.69
NbS00008178g0018.1 protein AED:0.03 eAED:0.03 QI:116|1|1|1|1|1|2|157|148; (*GB) gi|27529852|dbj|BAC53941.1| (e_value=4e-56) H2A histone [Nicotiana tabacum];; (*SWP) sp|P25469|H2A1_SOLLC (e_value=1e

Query	Observed	Mr(expt)	Mr(calc)	ppm	Miss	Score	Expect	Rank	Unique	Peptide
9340	404.2633	806.5121	806.5127	-0.73	0	50	0.00012	1		R.HVLLAVR.N 9338 9339 9342 9347 9348
10329	472.7685	943.5224	943.5240	-1.61	0	76	2.2e-006	1		K.AGLQFPVGR.I 10331
20019	758.1266	2271.3579	2271.3566	0.59	0	(35)	0.0015	1	U	K.LLSGVTIASGGVLPNINPVLLPK.K
20021	1136.6870	2271.3595	2271.3566	1.27	0	60	5.9e-006	1	U	K.LLSGVTIASGGVLPNINPVLLPK.K 20022
21182	1019.9117	3056.7132	3056.7121	0.36	1	34	0.0044	1	U	R.NDEELGKLLSGVTIASGGVLPNINPVLLPK.K

Proteins matching the same set of peptides:
NbS00020235g0008.1 **Mass:** 15471 **Score:** 276 **Matches:** 12(12) **Sequences:** 4(4)
NbS00020235g0008.1 protein AED:0.06 eAED:0.06 QI:118|1|1|1|1|1|2|157|148; (*GB) gi|27529852|dbj|BAC53941.1| (e_value=7e-58) H2A histone [Nicotiana tabacum];; (*SWP) sp|P25469|H2A1_SOLLC (e_value=3e

8. gi|148727309|ref|NP_001092039.1| **Mass:** 66230 **Score:** 276 **Matches:** 12(12) **Sequences:** 8(8) **emPAI:** 0.47
gi|148727309|ref|NP_001092039.1| keratin, type II cytoskeletal 2 epidermal [Pan troglodytes]

Query	Observed	Mr(expt)	Mr(calc)	ppm	Miss	Score	Expect	Rank	Unique	Peptide
10588	487.2690	972.5235	972.5240	-0.45	0	58	0.00014	1		K.IEISELNR.V 10590
11594	554.2752	1106.5359	1106.5356	0.24	0	50	0.00094	1	U	K.AQYEEIAQR.S 11593
12351	597.3109	1192.6072	1192.6088	-1.38	0	47	0.0017	1	U	K.YEELQVTVGR.H
12881	627.8077	1253.6009	1253.6001	0.70	0	61	4.9e-005	1	U	R.GFSSGSAVVSGGSR.R 12880
13732	665.3669	1328.7192	1328.7187	0.37	0	66	1.5e-005	1	U	R.NLDLDSIIAEVK.A
14852	730.9032	1459.7918	1459.7922	-0.26	0	44	0.0023	1	U	K.VDLLNQEIEFLK.V
14973	738.3962	1474.7778	1474.7780	-0.12	0	69	9.6e-006	1		R.FLEQQNQVLQTK.W 14975
21184	1022.1714	3063.4923	3063.4819	3.41	0	34	0.014	1	U	K.VLYDAEISQIHQSVTDTNVILSMDNSR.N

9. gi|291410763|ref|XP_002721657.1| **Mass:** 27347 **Score:** 273 **Matches:** 13(13) **Sequences:** 6(6) **emPAI:** 1.51
gi|291410763|ref|XP_002721657.1| PREDICTED: histone cluster 1, H2ag-like [Oryctolagus cuniculus]

Query	Observed	Mr(expt)	Mr(calc)	ppm	Miss	Score	Expect	Rank	Unique	Peptide
10329	472.7685	943.5224	943.5240	-1.61	0	76	2.2e-006	1		R.AGLQFPVGR.V 10331
12243	590.8141	1179.6137	1179.6135	0.19	0	69	9.8e-006	1		R.ISGLIYEETR.G 12240 12244
13531	655.8550	1309.6955	1309.6952	0.27	0	51	0.00052	1		K.TVTAMDVVYALK.R
13689	442.5898	1324.7475	1324.7463	0.94	0	(34)	0.013	1		R.DNIQGITKPAIR.R 13686
13691	663.3811	1324.7477	1324.7463	1.04	0	59	4.3e-005	1		R.DNIQGITKPAIR.R
13702	663.8500	1325.6854	1325.6901	-3.50	0	(41)	0.0046	1		K.TVTAMDVVYALK.R 13703
14901	733.9053	1465.7960	1465.7963	-0.20	1	36	0.012	1		K.TVTAMDVVYALKR.Q
16017	526.6346	1576.8821	1576.8824	-0.21	1	37	0.0083	1		R.ISGLIYEETRGVLK.V

10. NbS00054358g0003.1 **Mass:** 62948 **Score:** 264 **Matches:** 15(15) **Sequences:** 12(12) **emPAI:** 0.94
NbS00054358g0003.1 protein AED:0.12 eAED:0.14 QI:177|0.91|0.84|1|0.91|0.84|13|0|563; (*GB) gi|359489241|ref|XP_003633899.1| (e_value=0.0) PREDICTED: dynamin-related protein 1E [Vitis vinifera];; (

Query	Observed	Mr(expt)	Mr(calc)	ppm	Miss	Score	Expect	Rank	Unique	Peptide

10083	457.7586	913.5027	913.5021	0.57	0	35	0.02	1		K.HLEQFIK.A
10412	477.7555	953.4964	953.4971	-0.75	0	52	0.00041	1		R.FTDFAVVR.K
10623	**489.7722**	**977.5298**	**977.5294**	**0.41**	**0**	**35**	**0.026**	**1**	**U**	**R.YLSVQNVR.K**
11229	**530.8032**	**1059.5919**	**1059.5924**	**-0.48**	**0**	**54**	**0.0003**	**1**	**U**	**K.SSVLESIVAR.D**
11430	543.3325	1084.6504	1084.6492	1.09	0	39	0.0044	1		R.IPSITSLINK.S 11428
13614	440.2511	1317.7316	1317.7306	0.73	0	38	0.0087	1		R.LQHPWVGIVNR.S
16008	788.9283	1575.8421	1575.8409	0.78	0	56	0.00016	1		R.IYGVFDNQLPAALR.K
18151	921.4585	1840.9024	1840.8989	1.92	0	47	0.0013	1		R.IGSNVSSYVNMVSDTLR.N
18230	929.4543	1856.8940	1856.8938	0.10	0	(38)	0.0094	1		R.IGSNVSSYVNMVSDTLR.N
18245	620.6559	1858.9458	1858.9458	-0.01	1	37	0.01	1		K.LDLMDKGTNALDVLEGR.A 18246
18339	938.9868	1875.9590	1875.9578	0.64	0	35	0.017	1		R.FPSLQSTIAAASNEALEK.F
19459	1052.5187	2103.0228	2103.0154	3.52	0	46	0.0011	1		K.VAVDGQSETIVEDIENMVR.T
21264	1047.9334	3140.7782	3140.7849	-2.13	0	55	2.4e-005	1		K.QISPVPIHLSIYSPNVVNLTLIDLPGLTK.V

11. NbS00000471g0009.1 **Mass:** 32669 **Score:** 250 **Matches:** 10(9) **Sequences:** 6(6) **emPAI:** 0.97

NbS00000471g0009.1 protein AED:0.15 eAED:0.15 QI:0|0|0.33|1|1|1|3|0|301; (*GB) gi|115802|sp|P27494.1|CB23_TOBAC (e_value=0.0) RecName: Full=Chlorophyll a-b binding protein 36, chloroplastic; AltNar

Query	Observed	Mr(expt)	Mr(calc)	ppm	Miss	Score	Expect	Rank	Unique	Peptide
10655	**492.2527**	**982.4909**	**982.4913**	**-0.32**	**0**	**54**	**0.00028**	**1**		**K.FGEAVWFK.A** 7239 7240
11527	**549.7927**	**1097.5708**	**1097.5717**	**-0.84**	**0**	**55**	**0.00021**	**1**	**U**	**R.VGGGPLGEGLDK.I** 11528
13682	**442.5630**	**1324.6671**	**1324.6670**	**0.12**	**1**	**31**	**0.054**	**1**		**R.NRELEVIHCR.W**
18421	**946.4951**	**1890.9756**	**1890.9736**	**1.04**	**0**	**43**	**0.0027**	**1**	**U**	**R.WAMLGALGCVFPEILSK.N**
20375	**1203.5947**	**2405.1749**	**2405.1791**	**-1.74**	**0**	**64**	**1.6e-005**	**1**	**U**	**K.IYPGGAFDPLGLADDPEAFAELK.V**
20376	**802.7337**	**2405.1793**	**2405.1791**	**0.09**	**0**	**(64)**	**1.7e-005**	**1**	**U**	**K.IYPGGAFDPLGLADDPEAFAELK.V**
21567	**1348.2837**	**4041.8292**	**4041.8221**	**1.76**	**0**	**38**	**0.0023**	**1**	**U**	**K.YLGPFSEQTPSYLTGEFPGDYGWDTAGLSADPETFAR.N**

Proteins matching the same set of peptides:

NbS00050671g0012.1 **Mass:** 28761 **Score:** 250 **Matches:** 10(9) **Sequences:** 6(6)

NbS00050671g0012.1 protein AED:0.04 eAED:0.04 QI:0|1|0.5|1|1|1|2|0|265; (*GB) gi|115802|sp|P27494.1|CB23_TOBAC (e_value=0.0) RecName: Full=Chlorophyll a-b binding protein 36, chloroplastic; AltName

12. NbS00001169g0153.1 **Mass:** 36448 **Score:** 240 **Matches:** 9(9) **Sequences:** 7(7) **emPAI:** 0.84

NbS00001169g0153.1 protein AED:0.25 eAED:0.25 QI:0|0.5|0.4|1|1|1|5|0|341

Query	Observed	Mr(expt)	Mr(calc)	ppm	Miss	Score	Expect	Rank	Unique	Peptide
9536	**417.2350**	**832.4554**	**832.4555**	**-0.21**	**0**	**38**	**0.017**	**1**		**K.VAINGFGR.I**
10855	**505.8340**	**1009.6534**	**1009.6535**	**-0.19**	**0**	**52**	**4.7e-005**	**1**	**U**	**K.AVALVLPSLK.G** 10854
12883	**627.8100**	**1253.6054**	**1253.6040**	**1.12**	**0**	**57**	**0.00012**	**1**	**U**	**K.TFAEEVNAAFR.E** 12882
14213	**692.8942**	**1383.7739**	**1383.7722**	**1.26**	**0**	**65**	**1.3e-005**	**1**		**R.AAALNIVPTSTGAAK.A**
14932	**735.9022**	**1469.7898**	**1469.7878**	**1.32**	**0**	**33**	**0.031**	**1**	**U**	**R.VVDLADIVANQWK.-**
16533	**807.4235**	**1612.8325**	**1612.8308**	**1.03**	**0**	**62**	**4.1e-005**	**1**	**U**	**K.DSPLDVIAINDTGGVK.Q**
17883	**893.9142**	**1785.8139**	**1785.8111**	**1.61**	**0**	**54**	**0.00017**	**1**	**U**	**K.VIAWYDNEWGYSQR.V**

13. NbS00027807g0002.1 **Mass:** 45317 **Score:** 235 **Matches:** 10(9) **Sequences:** 7(7) **emPAI:** 0.76

NbS00027807g0002.1 protein AED:0.05 eAED:0.05 QI:0|1|0.5|1|0|0|2|953|413; (*GB) gi|68566313|sp|Q40450.2|EFTUA_NICSY (e_value=0.0) RecName: Full=Elongation factor TuA, chloroplastic; Short=EF-TuA; I

Query	Observed	Mr(expt)	Mr(calc)	ppm	Miss	Score	Expect	Rank	Unique	Peptide
11402	**541.3187**	**1080.6228**	**1080.6219**	**0.80**	**0**	**48**	**0.00056**	**1**	**U**	**K.FEAIVYVLK.K**
13712	**664.3345**	**1326.6544**	**1326.6490**	**4.09**	**0**	**34**	**0.024**	**1**	**U**	**R.STTVTGVEMFQK.I**
14667	**718.3400**	**1434.6654**	**1434.6626**	**1.91**	**1**	**54**	**0.00024**	**1**	**U**	**K.KYDEIDAAPEER.A** 8844
14732	**722.9039**	**1443.7933**	**1443.7908**	**1.73**	**0**	**30**	**0.05**	**1**	**U**	**K.QVGVPNMVVFLNK.Q**
17336	**850.4565**	**1698.8984**	**1698.8974**	**0.58**	**0**	**83**	**2.4e-007**	**1**	**U**	**K.ILDEAMAGDNVGLLLR.G**
18019	**905.9454**	**1809.8763**	**1809.8745**	**1.03**	**0**	**42**	**0.0033**	**1**	**U**	**R.GITINTATVEYETENR.H** 18021
19643	**723.0433**	**2166.1082**	**2166.1031**	**2.35**	**0**	**(36)**	**0.012**	**1**	**U**	**R.QTELPFLMAIEDVFSITGR.G**
19644	**1084.0631**	**2166.1117**	**2166.1031**	**3.96**	**0**	**58**	**6.6e-005**	**1**	**U**	**R.QTELPFLMAIEDVFSITGR.G**

14. NbS00004898g0002.1 **Mass:** 11402 **Score:** 229 **Matches:** 12(12) **Sequences:** 6(6) **emPAI:** 7.22

NbS00004898g0002.1 protein AED:0.00 eAED:0.00 QI:0|-1|0|1|-1|1|1|0|103; (*GB) gi|195617694|gb|ACG30677.1| (e_value=1e-51) histone H4 [Zea mays];; (*SWP) sp|P0CG89|H4_SOYBN (e_value=1e-52) Histone I

Query	Observed	Mr(expt)	Mr(calc)	ppm	Miss	Score	Expect	Rank	Unique	Peptide

10797	**502.2994**	**1002.5842**	**1002.5862**	**-1.99**	**0**	**46**	**0.0014**	**1**		**K.IFLENVIR.D**
12243	590.8141	1179.6137	1179.6135	0.19	0	69	9.8e-006	1		R.ISGLIYEETR.G 12240 12244
13531	655.8550	1309.6955	1309.6952	0.27	0	51	0.00052	1		K.TVTAMDVVYALK.R
13689	442.5898	1324.7475	1324.7463	0.94	0	(34)	0.013	1		R.DNIQGITKPAIR.R 13686
13691	663.3811	1324.7477	1324.7463	1.04	0	59	4.3e-005	1		R.DNIQGITKPAIR.R
13702	663.8500	1325.6854	1325.6901	-3.50	0	(41)	0.0046	1		K.TVTAMDVVYALK.R 13703
14901	733.9053	1465.7960	1465.7963	-0.20	1	36	0.012	1		K.TVTAMDVVYALKR.Q
16017	526.6346	1576.8821	1576.8824	-0.21	1	37	0.0083	1		R.ISGLIYEETRGVLK.I

Proteins matching the same set of peptides:
NbS00007760g0001.1 **Mass:** 11402 **Score:** 229 **Matches:** 12(12) **Sequences:** 6(6)
NbS00007760g0001.1 protein AED:0.00 eAED:0.00 QI:286|1|0.5|1|0|0|2|0|103; (*GB) gi|195617694|gb|ACG30677.1| (e_value=1e-51) histone H4 [Zea mays];; (*SWP) sp|P0CG89|H4_SOYBN (e_value=1e-52) Histone
NbS00010526g0004.1 **Mass:** 11402 **Score:** 229 **Matches:** 12(12) **Sequences:** 6(6)
NbS00010526g0004.1 protein AED:0.35 eAED:0.35 QI:0|-1|0|1|-1|1|1|0|103; (*GB) gi|195617694|gb|ACG30677.1| (e_value=1e-51) histone H4 [Zea mays];; (*SWP) sp|P0CG89|H4_SOYBN (e_value=1e-52) Histone
NbS00013276g0001.1 **Mass:** 11402 **Score:** 229 **Matches:** 12(12) **Sequences:** 6(6)
NbS00013276g0001.1 protein AED:0.23 eAED:0.23 QI:0|-1|0|1|-1|1|1|0|103; (*SWP) sp|P0CG89|H4_SOYBN (e_value=1e-52) Histone H4 OS=Glycine max PE=3 SV=1;; (*TAIR) AT5G59970.1 (e_value=9e-54) | Symbols
NbS00033043g0001.1 **Mass:** 11402 **Score:** 229 **Matches:** 12(12) **Sequences:** 6(6)
NbS00033043g0001.1 protein AED:0.23 eAED:0.23 QI:0|-1|0|1|-1|1|1|0|103; (*GB) gi|195617694|gb|ACG30677.1| (e_value=1e-51) histone H4 [Zea mays];; (*SWP) sp|P0CG89|H4_SOYBN (e_value=1e-52) Histone
NbS00034995g0002.1 **Mass:** 11748 **Score:** 229 **Matches:** 12(12) **Sequences:** 6(6)
NbS00034995g0002.1 protein AED:0.23 eAED:0.23 QI:37|0|0|0.5|1|1|2|0|106; (*GB) gi|308799807|ref|XP_003074685.1| (e_value=1e-50) Histone H4 (ISS) [Ostreococcus tauri];; (*SWP) sp|P0CG89|H4_SOYBN (e_
NbS00037466g0001.1 **Mass:** 11402 **Score:** 229 **Matches:** 12(12) **Sequences:** 6(6)
NbS00037466g0001.1 protein AED:0.28 eAED:0.28 QI:0|-1|0|1|-1|1|1|0|103; (*GB) gi|195617694|gb|ACG30677.1| (e_value=1e-51) histone H4 [Zea mays];; (*SWP) sp|P0CG89|H4_SOYBN (e_value=1e-52) Histone
NbS00045876g0001.1 **Mass:** 11402 **Score:** 229 **Matches:** 12(12) **Sequences:** 6(6)
NbS00045876g0001.1 protein AED:0.00 eAED:0.00 QI:0|-1|0|1|-1|1|1|0|103; (*GB) gi|195617694|gb|ACG30677.1| (e_value=1e-51) histone H4 [Zea mays];; (*SWP) sp|P0CG89|H4_SOYBN (e_value=1e-52) Histone
NbS00048006g0001.1 **Mass:** 11402 **Score:** 229 **Matches:** 12(12) **Sequences:** 6(6)
NbS00048006g0001.1 protein AED:0.09 eAED:0.10 QI:282|0|0.5|1|0|0.5|2|0|103; (*GB) gi|195617694|gb|ACG30677.1| (e_value=1e-51) histone H4 [Zea mays];; (*SWP) sp|P0CG89|H4_SOYBN (e_value=1e-52) Histo
NbS00056894g0001.1 **Mass:** 11402 **Score:** 229 **Matches:** 12(12) **Sequences:** 6(6)
NbS00056894g0001.1 protein AED:0.20 eAED:0.20 QI:0|-1|0|1|-1|1|1|0|103; (*SWP) sp|P0CG89|H4_SOYBN (e_value=1e-52) Histone H4 OS=Glycine max PE=3 SV=1;; (*TAIR) AT5G59970.1 (e_value=9e-54) | Symbols
NbC25918569g0001.1 **Mass:** 11402 **Score:** 229 **Matches:** 12(12) **Sequences:** 6(6)
NbC25918569g0001.1 protein AED:0.00 eAED:0.00 QI:0|-1|0|1|-1|1|1|0|103; (*GB) gi|195617694|gb|ACG30677.1| (e_value=1e-51) histone H4 [Zea mays];; (*SWP) sp|P0CG89|H4_SOYBN (e_value=1e-52) Histone
NbC25948449g0001.1 **Mass:** 11402 **Score:** 229 **Matches:** 12(12) **Sequences:** 6(6)
NbC25948449g0001.1 protein AED:0.24 eAED:0.24 QI:0|-1|0|1|-1|1|1|0|103; (*GB) gi|195617694|gb|ACG30677.1| (e_value=1e-51) histone H4 [Zea mays];; (*SWP) sp|P0CG89|H4_SOYBN (e_value=1e-52) Histone
NbS00001548g0007.1 **Mass:** 22455 **Score:** 229 **Matches:** 12(12) **Sequences:** 6(6)
NbS00001548g0007.1 protein AED:0.25 eAED:0.25 QI:0|0|0|1|0|0.5|2|0|202; (*SWP) sp|P0CG89|H4_SOYBN (e_value=2e-50) Histone H4 OS=Glycine max PE=3 SV=1;; (*TAIR) AT5G59970.1 (e_value=2e-51) | Symbols
NbS00046909g0009.1 **Mass:** 26576 **Score:** 229 **Matches:** 12(12) **Sequences:** 6(6)
NbS00046909g0009.1 protein AED:0.12 eAED:0.15 QI:52|0|0|1|0|0.33|3|151|237; (*GB) gi|195617694|gb|ACG30677.1| (e_value=4e-50) histone H4 [Zea mays];; (*SWP) sp|P0CG89|H4_SOYBN (e_value=8e-51) Histo

---

15. NbS00001942g0006.1 **Mass:** 15769 **Score:** 226 **Matches:** 10(10) **Sequences:** 3(3) **emPAI:** 0.79
NbS00001942g0006.1 protein AED:0.00 eAED:0.00 QI:75|1|1|1|1|1|2|198|151; (*GB) gi|351725389|ref|NP_001235555.1| (e_value=1e-63) uncharacterized protein LOC100305705 [Glycine max];; (*SWP) sp|Q6L50

Query	Observed	Mr(expt)	Mr(calc)	ppm	Miss	Score	Expect	Rank	Unique	Peptide
9340	404.2633	806.5121	806.5127	-0.73	0	50	0.00012	1		R.HVLLAVR.N 9338 9339 9342 9347 9348
10329	472.7685	943.5224	943.5240	-1.61	0	76	2.2e-006	1		K.AGLQFPVGR.I 10331
20207	**774.1422**	**2319.4048**	**2319.4042**	**0.26**	**0**	**42**	**0.00018**	**1**	**U**	**K.LLAGVTIAHGGVLPNINPILLPK.K** 20206

Proteins matching the same set of peptides:
NbS00012269g0005.1 **Mass:** 15797 **Score:** 226 **Matches:** 10(10) **Sequences:** 3(3)
NbS00012269g0005.1 protein AED:0.02 eAED:0.02 QI:75|1|1|1|1|1|2|181|151; (*GB) gi|351725389|ref|NP_001235555.1| (e_value=1e-63) uncharacterized protein LOC100305705 [Glycine max];; (*SWP) sp|Q2HU6
NbS00024367g0008.1 **Mass:** 15926 **Score:** 226 **Matches:** 10(10) **Sequences:** 3(3)
NbS00024367g0008.1 protein AED:0.03 eAED:0.03 QI:74|1|1|1|1|1|2|309|151; (*GB) gi|255584772|ref|XP_002533104.1| (e_value=5e-54) histone h2a, putative [Ricinus communis];; (*SWP) sp|Q2HU65|H2A2_MED
NbS00032978g0018.1 **Mass:** 15912 **Score:** 226 **Matches:** 10(10) **Sequences:** 3(3)
NbS00032978g0018.1 protein AED:0.04 eAED:0.04 QI:72|1|1|1|1|1|2|205|151; (*GB) gi|6009909|dbj|BAA85117.1| (e_value=1e-53) histone H2A-like protein [Solanum melongena];; (*SWP) sp|Q2HU65|H2A2_MEDTR

---

16. NbS00010297g0001.1 **Mass:** 15303 **Score:** 226 **Matches:** 10(10) **Sequences:** 3(3) **emPAI:** 0.82
NbS00010297g0001.1 protein AED:0.04 eAED:0.04 QI:122|1|1|1|1|1|2|350|144; (*GB) gi|225439749|ref|XP_002274570.1| (e_value=6e-44) PREDICTED: probable histone H2A.4 isoform 1 [Vitis vinifera];; (*SW

Query	Observed	Mr(expt)	Mr(calc)	ppm	Miss	Score	Expect	Rank	Unique	Peptide
9340	404.2633	806.5121	806.5127	-0.73	0	50	0.00012	1		R.HVLLAVR.N 9338 9339 9342 9347 9348

Query	Observed	Mr(expt)	Mr(calc)	ppm	Miss	Score	Expect	Rank	Unique	Peptide
10329	472.7685	943.5224	943.5240	-1.61	0	76	2.2e-006	1		K.AGLQFPVGR.I 10331
20207	774.1422	2319.4048	2319.4042	0.26	0	42	0.00018	1	U	K.LLAGVTIAHGGVLPNINPLLLPK.K 20206

17. NbS00017066g0003.1 **Mass:** 21609 **Score:** 221 **Matches:** 4(4) **Sequences:** 3(3) **emPAI:** 0.78

NbS00017066g0003.1 protein AED:0.01 eAED:0.01 QI:0|-1|0|1|-1|1|1|0|208; (*GB) gi|222051768|dbj|BAH15357.1| (e_value=3e-127) germin like protein [Nicotiana tabacum];; (*SWP) sp|Q9ZRA4|AB19A_PRUPE (e

Query	Observed	Mr(expt)	Mr(calc)	ppm	Miss	Score	Expect	Rank	Unique	Peptide
15633	775.9191	1549.8237	1549.8239	-0.15	0	108	9.8e-010	1	U	K.LVEATTFLDEATIK.K
17201	560.3130	1677.9173	1677.9189	-0.93	1	42	0.003	1	U	K.LVEATTFLDEATIKK.L
19781	734.0676	2199.1811	2199.1800	0.47	0	(64)	1.1e-005	1	U	K.AAVTPAFAAQFPGLNGLGLSAAR.L
19782	1100.5980	2199.1815	2199.1800	0.67	0	77	5.7e-007	1	U	K.AAVTPAFAAQFPGLNGLGLSAAR.L

Proteins matching the same set of peptides:

NbS00021913g0003.1 **Mass:** 21582 **Score:** 221 **Matches:** 4(4) **Sequences:** 3(3)

NbS00021913g0003.1 protein AED:0.01 eAED:0.01 QI:0|-1|0|1|-1|1|1|0|208; (*GB) gi|222051768|dbj|BAH15357.1| (e_value=6e-127) germin like protein [Nicotiana tabacum];; (*SWP) sp|Q9ZRA4|AB19A_PRUPE (e

18. NbS00033686g0006.1 **Mass:** 69818 **Score:** 215 **Matches:** 14(14) **Sequences:** 12(12) **emPAI:** 0.82

NbS00033686g0006.1 protein AED:0.16 eAED:0.16 QI:197|1|1|1|1|1|15|214|623; (*GB) gi|225453246|ref|XP_002265511.1| (e_value=0.0) PREDICTED: dynamin-related protein 1E isoform 1 [Vitis vinifera];; (

Query	Observed	Mr(expt)	Mr(calc)	ppm	Miss	Score	Expect	Rank	Unique	Peptide
9732	436.2722	870.5299	870.5287	1.37	0	32	0.032	1	U	K.SLIGLVNR.I
10763	499.7684	997.5222	997.5233	-1.11	0	32	0.038	1	U	R.FTDFTLVR.R
11122	523.7946	1045.5746	1045.5768	-2.11	0	35	0.027	1		K.SSVLESIVGR.D
12369	598.3269	1194.6393	1194.6397	-0.35	0	51	0.0005	1	U	R.LIEGALNYFR.G
13555	657.3528	1312.6911	1312.6874	2.82	0	31	0.049	1	U	K.VAVEEQPESVVK.D
13614	440.2511	1317.7316	1317.7306	0.73	0	38	0.0087	1		R.LQHPWVGIVNR.S
15546	513.9434	1538.8084	1538.8093	-0.56	0	(32)	0.032	1	U	R.GPAEASVDAVHFVLK.E
15548	770.4125	1538.8105	1538.8093	0.80	0	46	0.0013	1	U	R.GPAEASVDAVHFVLK.E
16008	788.9283	1575.8421	1575.8409	0.78	0	56	0.00016	1		R.IYGVFDNQLPAALR.K
17038	829.4212	1656.8279	1656.8280	-0.08	0	30	0.06	1	U	K.ALAELLDEDPALMEK.R
18225	928.4641	1854.9137	1854.9146	-0.48	0	41	0.0051	1	U	R.IGSNVSSYVNMVTDTLR.N
18245	620.6559	1858.9458	1858.9458	-0.01	1	37	0.01	1		K.LDLMDKGTNALDVLEGR.A 18246
21264	1047.9334	3140.7782	3140.7849	-2.13	0	55	2.4e-005	1		K.QISPVPIHLSIYSPNVVNLTLIDLPGLTK.V

19. NbS00000987g0004.1 **Mass:** 28363 **Score:** 200 **Matches:** 9(8) **Sequences:** 5(5) **emPAI:** 1.17

NbS00000987g0004.1 protein AED:0.21 eAED:0.21 QI:0|-1|0|1|-1|1|1|0|266; (*GB) gi|3036948|dbj|BAA25392.1| (e_value=0.0) light harvesting chlorophyll a/b-binding protein [Nicotiana sylvestris];; (*SW

Query	Observed	Mr(expt)	Mr(calc)	ppm	Miss	Score	Expect	Rank	Unique	Peptide
10655	492.2527	982.4909	982.4913	-0.32	0	54	0.00028	1		K.FGEAVWFK.A 7239 7240
13682	442.5630	1324.6671	1324.6670	0.12	1	31	0.054	1		K.NRELEVIHCR.W
16262	535.2672	1602.7796	1602.7791	0.36	0	36	0.016	1	U	K.AKPVSSGSPWYGPDR.V
16265	802.3995	1602.7844	1602.7791	3.33	0	(33)	0.037	1	U	K.AKPVSSGSPWYGPDR.V
18493	952.5001	1902.9856	1902.9848	0.40	0	82	3.8e-007	1		R.WAMLGALGCVFPELLAR.N
18614	960.4972	1918.9798	1918.9797	0.05	0	(38)	0.0085	1		R.WAMLGALGCVFPELLAR.N
21478	1251.9829	3752.9269	3752.9189	2.13	1	43	0.001	1		R.VAGGPLGEVVDPLYPGGSFDPLGLAEDPEAFAELKVK.E

20. NbS00011945g0012.1 **Mass:** 22544 **Score:** 196 **Matches:** 9(9) **Sequences:** 5(5) **emPAI:** 1.30

NbS00011945g0012.1 protein AED:0.16 eAED:0.16 QI:0|0|0|1|0|0.5|2|147|203; (*GB) gi|195617694|gb|ACG30677.1| (e_value=3e-50) histone H4 [Zea mays];; (*SWP) sp|P0CG89|H4_SOYBN (e_value=8e-51) Histone

Query	Observed	Mr(expt)	Mr(calc)	ppm	Miss	Score	Expect	Rank	Unique	Peptide
10797	502.2994	1002.5842	1002.5862	-1.99	0	46	0.0014	1		K.IFLENVIR.D
12243	590.8141	1179.6137	1179.6135	0.19	0	69	9.8e-006	1		R.ISGLIYEETR.G 12240 12244
13689	442.5898	1324.7475	1324.7463	0.94	0	(34)	0.013	1		R.DNIQGITKPAIR.R 13686
13691	663.3811	1324.7477	1324.7463	1.04	0	59	4.3e-005	1		R.DNIQGITKPAIR.R
13704	663.8527	1325.6908	1325.6901	0.51	0	38	0.011	1	U	K.TVTAMDVVYSLK.R
16017	526.6346	1576.8821	1576.8824	-0.21	1	37	0.0083	1		R.ISGLIYEETRGVLK.I

Proteins matching the same set of peptides:

NbS00050385g0006.1 **Mass:** 24229 **Score:** 196 **Matches:** 9(9) **Sequences:** 5(5)
NbS00050385g0006.1 protein AED:0.19 eAED:0.23 QI:29|0|0|1|0|0.33|3|0|217; (*GB) gi|195617694|gb|ACG30677.1| (e_value=4e-50) histone H4 [Zea mays];; (*SWP) sp|P0CG89|H4_SOYBN (e_value=1e-50) Histone

---

21. NbS00010009g0002.1 **Mass:** 118617 **Score:** 184 **Matches:** 8(7) **Sequences:** 7(6) **emPAI:** 0.24
NbS00010009g0002.1 protein AED:0.20 eAED:0.20 QI:0|1|1|1|1|1|21|408|1058; (*SWP) sp|O04379|AGO1_ARATH (e_value=0.0) Protein argonaute 1 OS=Arabidopsis thaliana GN=AGO1 PE=1 SV=1;; (*TAIR) AT1G4841(

Query	Observed	Mr(expt)	Mr(calc)	ppm	Miss	Score	Expect	Rank	Unique	Peptide
11449	544.3091	1086.6036	1086.6033	0.27	0	45	0.0021	1	U	R.NTVLVDALSR.R
11581	553.2794	1104.5442	1104.5451	-0.88	0	40	0.0083	1	U	R.ELTFPVDER.G
14484	707.8441	1413.6736	1413.6736	-0.03	0	39	0.0065	1	U	K.ITLIDDDDGPGGAR.R
14543	710.8962	1419.7778	1419.7762	1.13	0	31	0.041	1		K.SLYTAGPLPFVQK.D
14593	476.5848	1426.7326	1426.7317	0.64	1	31	0.057	1		R.RQPLGEGLESWR.G
18374	627.0155	1878.0247	1878.0211	1.93	0	(50)	0.00038	1		R.QADAPQEALQVLDIVLR.E
18375	940.0204	1878.0262	1878.0211	2.75	0	83	1.7e-007	1		R.QADAPQEALQVLDIVLR.E
18521	955.9556	1909.8966	1909.8948	0.95	0	32	0.031	1	U	R.ICETELGIVSQCCLTK.H

Proteins matching the same set of peptides:
NbS00034990g0010.1 **Mass:** 119669 **Score:** 184 **Matches:** 8(7) **Sequences:** 7(6)
NbS00034990g0010.1 protein AED:0.15 eAED:0.15 QI:0|0.95|0.95|1|0.95|0.90|21|286|1067; (*GB) gi|84688906|gb|ABC61502.1| (e_value=0.0) AGO1-1, partial [Nicotiana benthamiana];; (*SWP) sp|Q7XSA2|AGO1I

---

22. NbS00014580g0002.1 **Mass:** 28269 **Score:** 182 **Matches:** 8(7) **Sequences:** 5(5) **emPAI:** 0.95
NbS00014580g0002.1 protein AED:0.38 eAED:0.38 QI:0|-1|0|1|-1|1|1|0|265; (*GB) gi|3036944|dbj|BAA25389.1| (e_value=0.0) light harvesting chlorophyll a/b-binding protein [Nicotiana sylvestris];; (*SW

Query	Observed	Mr(expt)	Mr(calc)	ppm	Miss	Score	Expect	Rank	Unique	Peptide
10655	492.2527	982.4909	982.4913	-0.32	0	54	0.00028	1		K.FGEAVWFK.A 7239 7240
13682	442.5630	1324.6671	1324.6670	0.12	1	31	0.054	1		K.NRELEVIHCR.W
18054	606.6444	1816.9114	1816.9108	0.33	0	33	0.028	1	U	K.TVAKPVASSSPWYGPDR.V
18493	952.5001	1902.9856	1902.9848	0.40	0	82	3.8e-007	1		R.WAMLGALGCVFPELLAR.N
18614	960.4972	1918.9798	1918.9797	0.05	0	(38)	0.0085	1		R.WAMLGALGCVFPELLAR.N
21478	1251.9829	3752.9269	3752.9189	2.13	1	43	0.001	1		R.VAGGPLGEVVDPLYPGGSFDPLGLAEDPEAFAELKVK.E

---

23. NbS00000867g0011.1 **Mass:** 30015 **Score:** 179 **Matches:** 6(6) **Sequences:** 5(5) **emPAI:** 0.69
NbS00000867g0011.1 protein AED:0.34 eAED:0.34 QI:171|0.9|0.90|1|0.7|0.63|11|450|270; (*GB) gi|359492084|ref|XP_002284740.2| (e_value=1e-70) PREDICTED: uncharacterized protein LOC100254082 [Vitis v:

Query	Observed	Mr(expt)	Mr(calc)	ppm	Miss	Score	Expect	Rank	Unique	Peptide
9911	447.7314	893.4483	893.4494	-1.31	0	35	0.029	1	U	K.GSDEIVFK.A
13293	643.8153	1285.6161	1285.6150	0.85	0	46	0.0015	1	U	R.AETPIDENEIR.I
17820	886.3932	1770.7718	1770.7697	1.20	0	72	1.8e-006	1	U	K.VLTDFDYDGEGSPSGGR.R 17822
19207	1014.9052	2027.7959	2027.7956	0.14	0	41	0.00041	1	U	R.GFAGNGFMSAEYDDGGYDR.N
21453	1226.9769	3677.9090	3677.9040	1.35	0	45	0.0005	1		R.IVGLHQITSITSTDITDTWEPLEEGLLPLETTR.H

Proteins matching the same set of peptides:
NbS00030156g0009.1 **Mass:** 31162 **Score:** 179 **Matches:** 6(6) **Sequences:** 5(5)
NbS00030156g0009.1 protein AED:0.29 eAED:0.29 QI:170|1|0.9|1|0.77|0.7|10|232|281; (*GB) gi|358248282|ref|NP_001239854.1| (e_value=2e-70) uncharacterized protein LOC100818986 [Glycine max];; (*SWP)

---

24. NbS00010583g0001.1 **Mass:** 22083 **Score:** 176 **Matches:** 4(4) **Sequences:** 2(2) **emPAI:** 0.76
NbS00010583g0001.1 protein AED:0.25 eAED:0.25 QI:0|-1|0|1|-1|1|1|0|210; (*GB) gi|31711507|dbj|BAC77634.1| (e_value=2e-132) 24K germin like protein [Nicotiana tabacum];; (*SWP) sp|O04011|ABP20_PRUPI

Query	Observed	Mr(expt)	Mr(calc)	ppm	Miss	Score	Expect	Rank	Unique	Peptide
8744	349.2341	696.4536	696.4534	0.38	0	42	0.00062	1	U	K.LNPLIK.A
20066	762.7400	2285.1981	2285.1991	-0.40	0	87	6.9e-008	1	U	K.AAVTPAFAPQFPGLNGLGISMAR.L
20068	1143.6085	2285.2025	2285.1991	1.50	0	(54)	0.00013	1	U	K.AAVTPAFAPQFPGLNGLGISMAR.L
20129	768.0723	2301.1952	2301.1940	0.51	0	(49)	0.00052	1	U	K.AAVTPAFAPQFPGLNGLGISMAR.L

Proteins matching the same set of peptides:
NbC25250105g0001.1 **Mass:** 20517 **Score:** 176 **Matches:** 4(4) **Sequences:** 2(2)
NbC25250105g0001.1 protein AED:0.01 eAED:0.01 QI:0|-1|1|1|-1|1|1|0|194; (*GB) gi|31711507|dbj|BAC77634.1| (e_value=1e-120) 24K germin like protein [Nicotiana tabacum];; (*SWP) sp|O04011|ABP20_PRUPI

---

25. NbS00007511g0005.1 **Mass:** 117195 **Score:** 168 **Matches:** 8(8) **Sequences:** 6(6) **emPAI:** 0.21
NbS00007511g0005.1 protein AED:0.26 eAED:0.26 QI:0|0.62|0.55|0.88|1|1|9|0|1084; (*GB) gi|359477631|ref|XP_002274485.2| (e_value=0.0) PREDICTED: uncharacterized protein LOC100252874 [Vitis vinifera]

Query	Observed	Mr(expt)	Mr(calc)	ppm	Miss	Score	Expect	Rank	Unique	Peptide
9471	412.7709	823.5272	823.5279	-0.90	0	42	0.00082	1	U	K.LAAIPIAR.N
10598	488.2636	974.5126	974.5106	2.02	0	42	0.0072	1	U	R.LNDILEMK.R
12621	613.8591	1225.7036	1225.7030	0.45	0	59	4.5e-005	1	U	K.TPILVATDVAAR.G
13774	668.8238	1335.6330	1335.6315	1.14	0	53	0.00032	1		R.MLDMGFEPQIR.K
13943	676.8201	1351.6256	1351.6264	-0.63	0	(40)	0.0059	1		R.MLDMGFEPQIR.K
17932	897.4805	1792.9465	1792.9431	1.88	0	40	0.0056	1	U	R.NPVQVNIGNVDQLAANK.S
18242	930.4455	1858.8764	1858.8737	1.45	0	42	0.0033	1	U	R.AGATGISYTFLSDQDWK.Y 18241

26. NbS00004085g0015.1 **Mass:** 23594 **Score:** 162 **Matches:** 7(7) **Sequences:** 6(6) **emPAI:** 1.21
NbS00004085g0015.1 protein AED:0.29 eAED:0.29 QI:92|0.25|0.2|1|1|1|5|0|225; (*GB) gi|110377793|gb|ABG73417.1| (e_value=7e-87) chloroplast pigment-binding protein CP26 [Nicotiana tabacum];; (*SWP) s

Query	Observed	Mr(expt)	Mr(calc)	ppm	Miss	Score	Expect	Rank	Unique	Peptide
10566	485.7643	969.5140	969.5131	0.89	0	43	0.0028	1	U	K.DPDQAAILK.V
13260	642.3465	1282.6784	1282.6768	1.24	0	39	0.0075	1	U	K.AVAVAPADEELAK.W
14460	706.3954	1410.7763	1410.7718	3.22	1	52	0.0003	1	U	K.KAVAVAPADEELAK.W
8986	742.3400	1482.6654	1482.6602	3.54	0	34	0.039	1	U	K.FGADCGPEAVWFK.T
9623	848.9500	1695.8854	1695.8832	1.34	0	55	0.00017	1	U	K.TGALLLDGNTLNYFGK.N 9622
18136	919.4706	1836.9267	1836.9233	1.89	0	53	0.00028	1	U	R.WAMLGAAGFVIPEAFNK.F

27. NbS00014977g0008.1 **Mass:** 125165 **Score:** 155 **Matches:** 8(6) **Sequences:** 6(5) **emPAI:** 0.23
NbS00014977g0008.1 protein AED:0.16 eAED:0.16 QI:16|0.95|0.90|1|0.95|0.90|22|432|1125; (*GB) gi|84688908|gb|ABC61503.1| (e_value=0.0) AGO1-2, partial [Nicotiana benthamiana];; (*SWP) sp|Q7XSA2|AGO1

Query	Observed	Mr(expt)	Mr(calc)	ppm	Miss	Score	Expect	Rank	Unique	Peptide
10933	510.2979	1018.5812	1018.5811	0.05	0	32	0.039	1	U	K.QYLANVALK.I
11449	544.3091	1086.6036	1086.6033	0.27	0	45	0.0021	1	U	R.NTVLVDAISR.R
12653	410.5667	1228.6784	1228.6775	0.71	1	(30)	0.062	1	U	K.ISDKLAQVEAR.I
12654	615.3467	1228.6788	1228.6775	1.05	1	40	0.0073	1	U	K.ISDKLAQVEAR.I
14543	710.8962	1419.7778	1419.7762	1.13	0	31	0.041	1		K.SLYTAGPLPFVQK.D
14593	476.5848	1426.7326	1426.7317	0.64	1	31	0.057	1		R.RQPLGEGLESWR.G
18374	627.0155	1878.0247	1878.0211	1.93	0	(50)	0.00038	1		K.QADAPQEALQVLDIVLR.E
18375	940.0204	1878.0262	1878.0211	2.75	0	83	1.7e-007	1		K.QADAPQEALQVLDIVLR.E

28. NbS00035687g0007.1 **Mass:** 28986 **Score:** 151 **Matches:** 4(4) **Sequences:** 4(4) **emPAI:** 0.55
NbS00035687g0007.1 protein AED:0.23 eAED:0.23 QI:300|1|0.75|1|1|1|4|0|274; (*GB) gi|84620802|gb|ABC59515.1| (e_value=9e-120) chloroplast photosystem II 22 kDa component [Nicotiana benthamiana];; (

Query	Observed	Mr(expt)	Mr(calc)	ppm	Miss	Score	Expect	Rank	Unique	Peptide
10017	453.2456	904.4767	904.4767	0.07	0	46	0.0025	1	U	K.ANELFVGR.L
16055	790.9207	1579.8268	1579.8246	1.36	0	96	1.5e-008	1	U	K.SALGLSEGGPLFGFTK.A
16077	792.8988	1583.7831	1583.7832	-0.07	0	32	0.034	1		K.VEDGIFGTSGGIGFTK.Q
17673	874.9847	1747.9548	1747.9542	0.33	0	49	0.00057	1		R.VAMIGFAASLLGEAITGK.G

29. NbS00000671g0042.1 **Mass:** 63379 **Score:** 146 **Matches:** 6(6) **Sequences:** 5(5) **emPAI:** 0.29
NbS00000671g0042.1 protein AED:0.13 eAED:0.13 QI:0|0.83|0.57|1|1|0.85|7|0|571; (*GB) gi|729623|sp|Q03685.1|BIP5_TOBAC (e_value=0.0) RecName: Full=Luminal-binding protein 5; Short=BiP 5; AltName: Fu

Query	Observed	Mr(expt)	Mr(calc)	ppm	Miss	Score	Expect	Rank	Unique	Peptide
11992	578.3276	1154.6406	1154.6408	-0.14	0	56	0.00011	1	U	K.DAGVIAGLNVAR.I 11993
12004	579.3195	1156.6245	1156.6241	0.37	0	40	0.0081	1	U	K.FDLTGIAPAPR.G
14255	695.3284	1388.6423	1388.6394	2.08	0	33	0.026	1	U	R.NSLETYVYNMR.N
15197	750.9053	1499.7961	1499.7944	1.16	0	39	0.0075	1	U	K.NQIDEIVLVGGSTR.I
17087	830.4522	1658.8899	1658.8879	1.19	0	51	0.00043	1		R.IINEPTAAAIAYGLDK.K

Proteins matching the same set of peptides:
NbS00040865g0006.1 **Mass:** 66719 **Score:** 146 **Matches:** 6(6) **Sequences:** 5(5)
NbS00040865g0006.1 protein AED:0.18 eAED:0.18 QI:84|0.85|0.62|0.87|1|0.87|8|0|601; (*GB) gi|729623|sp|Q03685.1|BIP5_TOBAC (e_value=0.0) RecName: Full=Luminal-binding protein 5; Short=BiP 5; AltName

30. NbS00007993g0305.1 **Mass:** 30118 **Score:** 146 **Matches:** 5(5) **Sequences:** 4(4) **emPAI:** 0.52

NbS00007993g0305.1 protein AED:0.00 eAED:0.00 QI:0|-1|0|1|-1|1|1|0|261

Query	Observed	Mr(expt)	Mr(calc)	ppm	Miss	Score	Expect	Rank	Unique	Peptide
11557	551.3292	1100.6438	1100.6441	-0.29	0	62	3.5e-005	1	U	K.VLAELLSASAK.D 11558
12285	592.3381	1182.6616	1182.6608	0.66	0	48	0.00078	1	U	K.NQITAEAIVPK.N
14914	734.8226	1467.6307	1467.6334	-1.82	0	38	0.0052	1	U	R.GDISSNDCLSCIK.D
17184	838.4255	1674.8365	1674.8334	1.88	0	53	0.0003	1		R.YELYPFFFPLDPK.E

---

31. NbS00019305g0027.1 **Mass:** 59772 **Score:** 140 **Matches:** 5(4) **Sequences:** 5(4) **emPAI:** 0.31

NbS00019305g0027.1 protein AED:0.37 eAED:0.37 QI:0|1|0.88|1|1|1|9|0|556; (*GB) gi|3676296|gb|AAD03392.1| (e_value=0.0) mitochondrial ATPase beta subunit [Nicotiana sylvestris];; (*SWP) sp|P17614|A

Query	Observed	Mr(expt)	Mr(calc)	ppm	Miss	Score	Expect	Rank	Unique	Peptide
10602	488.2848	974.5550	974.5549	0.04	0	46	0.002	1		K.IGLFGGAGVGK.T
12176	587.3353	1172.6560	1172.6554	0.54	0	31	0.054	1	U	K.VVDLLAPYQR.G
14439	705.4092	1408.8038	1408.8038	-0.02	0	59	4e-005	1	U	R.VLNTGSPITVPVGR.A
19748	1093.5803	2185.1461	2185.1379	3.75	0	48	0.00073	1	U	R.IPSAVGYQPTLATDLGGLQER.I
20858	897.4503	2689.3291	2689.3302	-0.41	1	46	0.00093	1	U	K.NLQDIIAILGMDELSEDDKMTVAR.A

Proteins matching the same set of peptides:

NbS00020973g0010.1 **Mass:** 58453 **Score:** 140 **Matches:** 5(4) **Sequences:** 5(4)

NbS00020973g0010.1 protein AED:0.23 eAED:0.23 QI:0|0.87|0.77|1|0.87|1|9|0|545; (*GB) gi|3676296|gb|AAD03392.1| (e_value=0.0) mitochondrial ATPase beta subunit [Nicotiana sylvestris];; (*SWP) sp|P1

---

32. NbS00043180g0003.1 **Mass:** 52871 **Score:** 138 **Matches:** 6(6) **Sequences:** 5(5) **emPAI:** 0.44

NbS00043180g0003.1 protein AED:0.10 eAED:0.10 QI:0|1|0.5|1|1|1|4|944|486; (*GB) gi|113461|sp|P27081.1|ADT2_SOLTU (e_value=0.0) RecName: Full=ADP,ATP carrier protein, mitochondrial; AltName: Full=AI

Query	Observed	Mr(expt)	Mr(calc)	ppm	Miss	Score	Expect	Rank	Unique	Peptide
8160	596.3200	1190.6254	1190.6295	-3.43	0	36	0.038	1		R.AVAGAGVLAGYDK.L
12725	618.8333	1235.6521	1235.6510	0.88	0	51	0.00066	1	U	K.TLASDGIAGLYR.G
14028	680.8639	1359.7132	1359.7068	4.75	0	41	0.0065	1		K.LLIQNQDEMIK.A
14743	723.8748	1445.7351	1445.7343	0.52	0	44	0.0029	1		R.YFPTQALNFAFK.D
18526	638.3334	1911.9783	1911.9764	0.97	0	(45)	0.0017	1		K.GLAAFATDFLMGGVSAAVSK.T
18527	956.9980	1911.9814	1911.9764	2.60	0	47	0.00093	1		K.GLAAFATDFLMGGVSAAVSK.T

Proteins matching the same set of peptides:

NbS00043181g0003.1 **Mass:** 52686 **Score:** 138 **Matches:** 6(6) **Sequences:** 5(5)

NbS00043181g0003.1 protein AED:0.20 eAED:0.20 QI:0|1|0.5|1|1|1|4|682|483; (*GB) gi|113461|sp|P27081.1|ADT2_SOLTU (e_value=0.0) RecName: Full=ADP,ATP carrier protein, mitochondrial; AltName: Full=AI

---

33. NbS00009714g0011.1 **Mass:** 45483 **Score:** 131 **Matches:** 5(5) **Sequences:** 4(4) **emPAI:** 0.32

NbS00009714g0011.1 protein AED:0.20 eAED:0.20 QI:131|0.83|0.71|1|0.83|0.57|7|463|410; (*SWP) sp|Q40565|RCA2_TOBAC (e_value=0.0) Ribulose bisphosphate carboxylase/oxygenase activase 2, chloroplastic

Query	Observed	Mr(expt)	Mr(calc)	ppm	Miss	Score	Expect	Rank	Unique	Peptide
11866	569.8533	1137.6921	1137.6910	0.96	0	38	0.0019	1	U	K.VPLILGVWGGK.G
17564	868.9083	1735.8021	1735.8013	0.44	0	65	1.4e-005	1		K.GLVQDFSDDQQDITR.G 17568
18391	941.9896	1881.9646	1881.9625	1.09	0	41	0.0044	1		K.IVDTFPGQSIDFFGALR.A
18506	953.9849	1905.9552	1905.9506	2.41	0	36	0.015	1		K.LLEYGNMLVQEQENVK.R

Proteins matching the same set of peptides:

NbS00047700g0013.1 **Mass:** 81330 **Score:** 131 **Matches:** 5(5) **Sequences:** 4(4)

NbS00047700g0013.1 protein AED:0.19 eAED:0.19 QI:131|0.6|0.63|1|0.8|0.54|11|307|726; (*GB) gi|12643758|sp|Q40565.1|RCA2_TOBAC (e_value=0.0) RecName: Full=Ribulose bisphosphate carboxylase/oxygenase

---

34. NbS00019623g0001.1 **Mass:** 49575 **Score:** 127 **Matches:** 5(5) **Sequences:** 4(4) **emPAI:** 0.29

NbS00019623g0001.1 protein AED:0.21 eAED:0.21 QI:0|1|0.5|1|1|1|2|0|447; (*GB) gi|3869088|dbj|BAA34348.1| (e_value=0.0) elongation factor-1 alpha [Nicotiana paniculata];; (*SWP) sp|P17786|EF1A_SOLL

Query	Observed	Mr(expt)	Mr(calc)	ppm	Miss	Score	Expect	Rank	Unique	Peptide
10089	457.7871	913.5596	913.5597	-0.08	0	56	0.00011	1	U	R.QTVAVGVIK.S
10600	488.2791	974.5436	974.5437	-0.12	0	39	0.012	1	U	R.LPLQDVYK.I
7489	513.3100	1024.6054	1024.6030	2.44	0	63	2.7e-005	1	U	K.IGGIGTVPVGR.V 10973
10978	513.7479	1025.4812	1025.4818	-0.60	0	45	0.0016	1	U	R.STNLDWYK.G

Proteins matching the same set of peptides:

NbS00023178g0001.1 **Mass:** 45458 **Score:** 127 **Matches:** 5(5) **Sequences:** 4(4)
NbS00023178g0001.1 protein AED:0.03 eAED:0.03 QI:0|0|0|1|1|1|3|0|413; (*SWP) sp|Q40034|EF1A2_HORVU (e_value=0.0) Elongation factor 1-alpha OS=Hordeum vulgare GN=BLT63 PE=1 SV=1;; (*TAIR) AT5G60390.
NbS00024768g0013.1 **Mass:** 49347 **Score:** 127 **Matches:** 5(5) **Sequences:** 4(4)
NbS00024768g0013.1 protein AED:0.17 eAED:0.19 QI:3|0.33|0|1|0.66|0.75|4|0|443; (*GB) gi|3869088|dbj|BAA34348.1| (e_value=0.0) elongation factor-1 alpha [Nicotiana paniculata];; (*SWP) sp|P17786|EF1
NbS00037026g0004.1 **Mass:** 49587 **Score:** 127 **Matches:** 5(5) **Sequences:** 4(4)
NbS00037026g0004.1 protein AED:0.17 eAED:0.17 QI:0|1|0.5|1|1|1|2|0|447; (*GB) gi|3869088|dbj|BAA34348.1| (e_value=0.0) elongation factor-1 alpha [Nicotiana paniculata];; (*SWP) sp|P43643|EF1A_TOBAC
NbS00060738g0002.1 **Mass:** 50846 **Score:** 127 **Matches:** 5(5) **Sequences:** 4(4)
NbS00060738g0002.1 protein AED:0.07 eAED:0.09 QI:0|0.33|0.25|1|1|1|4|0|458; (*SWP) sp|P43643|EF1A_TOBAC (e_value=0.0) Elongation factor 1-alpha OS=Nicotiana tabacum PE=2 SV=1;; (*TAIR) AT5G60390.3
NbS00026055g0002.1 **Mass:** 40879 **Score:** 127 **Matches:** 5(5) **Sequences:** 4(4)
NbS00026055g0002.1 protein AED:0.04 eAED:0.06 QI:0|-1|0|1|-1|1|1|0|373; (*GB) gi|1864017|dbj|BAA09709.1| (e_value=0.0) elongation factor-1 alpha [Nicotiana tabacum];; (*SWP) sp|P43643|EF1A_TOBAC (e
NbS00007372g0013.1 **Mass:** 54761 **Score:** 127 **Matches:** 5(5) **Sequences:** 4(4)
NbS00007372g0013.1 protein AED:0.21 eAED:0.21 QI:0|0|0|1|0.5|0.66|3|0|494; (*GB) gi|1864017|dbj|BAA09709.1| (e_value=0.0) elongation factor-1 alpha [Nicotiana tabacum];; (*SWP) sp|P43643|EF1A_TOBAC
NbS00024768g0017.1 **Mass:** 101076 **Score:** 127 **Matches:** 5(5) **Sequences:** 4(4)
NbS00024768g0017.1 protein AED:0.14 eAED:0.15 QI:0|0|0.33|1|0|0|6|0|911; (*GB) gi|297849086|ref|XP_002892424.1| (e_value=0.0) T6D22.2 [Arabidopsis lyrata subsp. lyrata];; (*SWP) sp|P17786|EF1A_SOLI

---

35. NbS00019862g0010.1 **Mass:** 72039 **Score:** 127 **Matches:** 6(6) **Sequences:** 4(4) **emPAI:** 0.20
NbS00019862g0010.1 protein AED:0.10 eAED:0.10 QI:0|0.92|1|1|0.92|0.93|15|328|640; (*GB) gi|5931765|emb|CAB56619.1| (e_value=0.0) phragmoplastin [Nicotiana tabacum];; (*SWP) sp|Q39828|SDL5A_SOYBN (e

Query	Observed	Mr(expt)	Mr(calc)	ppm	Miss	Score	Expect	Rank	Unique	Peptide
11410	541.8327	1081.6509	1081.6495	1.23	0	41	0.0016	1		R.IPGLQSLINK.T
11803	377.9085	1130.7038	1130.7036	0.14	0	35	0.0048	1		R.RPLVLQLHR.L
13724	664.8782	1327.7419	1327.7401	1.37	0	54	0.00018	1		K.LQFPWIGVVNR.S 13723
17335	850.4393	1698.8640	1698.8974	-19.68	1	37	0.01	1	U	K.QLGTLLDEDPAIMKR.R 17334

---

36. NbS00042109g0018.1 **Mass:** 27332 **Score:** 126 **Matches:** 5(5) **Sequences:** 5(5) **emPAI:** 0.78
NbS00042109g0018.1 protein AED:0.00 eAED:0.00 QI:0|1|0.5|1|1|1|2|0|256; (*SWP) sp|P27524|CB4A_SOLLC (e_value=3e-163) Chlorophyll a-b binding protein CP24 10A, chloroplastic OS=Solanum lycopersicum

Query	Observed	Mr(expt)	Mr(calc)	ppm	Miss	Score	Expect	Rank	Unique	Peptide
18401	**944.4307**	**1886.8468**	**1886.8435**	**1.73**	**0**	**46**	**0.001**	**1**	**U**	**K.TAENFANFTGEQGYPGGK.F**
20443	**1213.6121**	**2425.2096**	**2425.2053**	**1.75**	**0**	**56**	**0.00012**	**1**	**U**	**K.FFDPLSLAGTLQDGVYIPDTEK.L**
20488	**1220.5682**	**2439.1219**	**2439.1172**	**1.95**	**0**	**31**	**0.03**	**1**	**U**	**R.WVDFFNPESQSVEWATPWSK.T**
20989	**942.1530**	**2823.4372**	**2823.4331**	**1.47**	**1**	**36**	**0.0086**	**1**	**U**	**K.FFDPLSLAGTLQDGVYIPDTEKLER.L**
21418	**1172.9136**	**3515.7189**	**3515.7249**	**-1.72**	**1**	**38**	**0.0044**	**1**	**U**	**K.GGGNLVDPEWLDGSLPGDYGFDPLGLGKDPAFLK.W**

---

37. NbS00010295g0007.1 **Mass:** 13224 **Score:** 121 **Matches:** 2(2) **Sequences:** 1(1) **emPAI:** 0.26
NbS00010295g0007.1 protein AED:0.12 eAED:0.12 QI:0|0|0|0.5|1|1|2|0|119; (*SWP) sp|P69569|RBL_EUPES (e_value=5e-22) Ribulose bisphosphate carboxylase large chain (Fragment) OS=Euphorbia esula GN=rbc

Query	Observed	Mr(expt)	Mr(calc)	ppm	Miss	Score	Expect	Rank	Unique	Peptide
10945	**511.2694**	**1020.5242**	**1020.5240**	**0.20**	**0**	**79**	**1.1e-006**	**1**	**U**	**K.DTDILAAFR.V** 10942

Proteins matching the same set of peptides:
NbS00047753g0001.1 **Mass:** 13332 **Score:** 121 **Matches:** 2(2) **Sequences:** 1(1)
NbS00047753g0001.1 protein AED:0.28 eAED:0.28 QI:0|0|0|0.5|1|0|2|0|115; (*GB) gi|306481964|dbj|BAJ17086.1| (e_value=9e-37) ribulose-1,5-bisphosphate carboxylase/oxygenase large subunit [Solanum sp.

---

38. NbS00029393g0012.1 **Mass:** 49230 **Score:** 117 **Matches:** 4(4) **Sequences:** 3(3) **emPAI:** 0.21
NbS00029393g0012.1 protein AED:0.08 eAED:0.08 QI:319|0.42|0.37|1|1|1|8|307|444; (*GB) gi|12643757|sp|Q40460.1|RCA1_TOBAC (e_value=0.0) RecName: Full=Ribulose bisphosphate carboxylase/oxygenase act:

Query	Observed	Mr(expt)	Mr(calc)	ppm	Miss	Score	Expect	Rank	Unique	Peptide
17272	**845.4299**	**1688.8453**	**1688.8410**	**2.56**	**0**	**45**	**0.0018**	**1**		**K.LLNSFDGPPTFEQPK.M**
17564	868.9083	1735.8021	1735.8013	0.44	0	65	1.4e-005	1		K.GLVQDFSDDQQDITR.G 17568
18506	953.9849	1905.9552	1905.9506	2.41	0	36	0.015	1		K.LLEYGNMLVQEQENVK.R

Proteins matching the same set of peptides:
NbS00044990g0002.1 **Mass:** 49599 **Score:** 117 **Matches:** 4(4) **Sequences:** 3(3)
NbS00044990g0002.1 protein AED:0.04 eAED:0.04 QI:333|0.66|0.57|1|1|1|7|326|449; (*SWP) sp|Q40460|RCA1_TOBAC (e_value=0.0) Ribulose bisphosphate carboxylase/oxygenase activase 1, chloroplastic OS=N:

---

39. NbS00033593g0008.1 **Mass:** 32632 **Score:** 112 **Matches:** 3(3) **Sequences:** 3(3) **emPAI:** 0.34
NbS00033593g0008.1 protein AED:0.04 eAED:0.05 QI:310|0|0.5|1|0|0|2|0|280; (*GB) gi|388491254|gb|AFK33693.1| (e_value=3e-98) unknown [Lotus japonicus];; (*SWP) sp|Q9LV60|CRR55_ARATH (e_value=1e-77)

Query	Observed	Mr(expt)	Mr(calc)	ppm	Miss	Score	Expect	Rank	Unique	Peptide

Query	Observed	Mr(expt)	Mr(calc)	ppm	Miss	Score	Expect	Rank	Unique	Peptide
11918	**573.3424**	**1144.6703**	**1144.6703**	**-0.05**	**0**	**41**	**0.0036**	**1**	**U**	**K.VLAELLSVSSK.D**
12145	**586.3204**	**1170.6262**	**1170.6244**	**1.50**	**0**	**61**	**5.2e-005**	**1**	**U**	**K.NQITAEATVPK.N**
17184	838.4255	1674.8365	1674.8334	1.88	0	53	0.0003	1		R.YELYPFFFPLDPK.E

40. NbS00008911g0002.1 **Mass:** 47595 **Score:** 110 **Matches:** 6(6) **Sequences:** 4(4) **emPAI:** 0.31
NbS00008911g0002.1 protein AED:0.35 eAED:0.35 QI:460|0.77|0.7|1|1|1|10|229|443; (*GB) gi|327198779|emb|CBL43264.1| (e_value=0.0) glyceraldehyde-3-phosphate dehydrogenase [Solanum tuberosum];; (*SWI

Query	Observed	Mr(expt)	Mr(calc)	ppm	Miss	Score	Expect	Rank	Unique	Peptide
9536	417.2350	832.4554	832.4555	-0.21	0	38	0.017	1		K.VAINGFGR.I
11298	**534.3447**	**1066.6749**	**1066.6750**	**-0.11**	**0**	**38**	**0.0012**	**1**	**U**	**K.AVSLVLPQLK.G** 11299
14213	692.8942	1383.7739	1383.7722	1.26	0	65	1.3e-005	1		R.AAALNIVPTSTGAAK.A
16531	**807.4235**	**1612.8324**	**1612.8308**	**0.95**	**0**	**34**	**0.023**	**1**	**U**	**K.DSPLDVIVVNDSGGVK.N** 16533

Proteins matching the same set of peptides:
NbS00033331g0008.1 **Mass:** 45260 **Score:** 110 **Matches:** 6(6) **Sequences:** 4(4)
NbS00033331g0008.1 protein AED:0.30 eAED:0.30 QI:475|0.77|0.8|1|0.77|0.7|10|324|419; (*GB) gi|327198779|emb|CBL43264.1| (e_value=0.0) glyceraldehyde-3-phosphate dehydrogenase [Solanum tuberosum];;

41. NbS00002894g0003.1 **Mass:** 42068 **Score:** 108 **Matches:** 5(5) **Sequences:** 4(4) **emPAI:** 0.46
NbS00002894g0003.1 protein AED:0.02 eAED:0.02 QI:0|1|0.5|1|1|1|2|0|386; (*GB) gi|78191448|gb|ABB29945.1| (e_value=0.0) ADP/ATP translocator-like [Solanum tuberosum];; (*SWP) sp|P25083|ADT1_SOLTU (e

Query	Observed	Mr(expt)	Mr(calc)	ppm	Miss	Score	Expect	Rank	Unique	Peptide
8160	596.3200	1190.6254	1190.6295	-3.43	0	36	0.038	1		R.AVAGAGVLAGYDK.L
14028	680.8639	1359.7132	1359.7068	4.75	0	41	0.0065	1	U	K.LLIQNQDEMLK.A
14743	723.8748	1445.7351	1445.7343	0.52	0	44	0.0029	1		R.YFPTQALNFAFK.D
18526	638.3334	1911.9783	1911.9764	0.97	0	(45)	0.0017	1		K.GLAAFATDFLMGGVSAAVSK.T
18527	956.9980	1911.9814	1911.9764	2.60	0	47	0.00093	1		K.GLAAFATDFLMGGVSAAVSK.T

Proteins matching the same set of peptides:
NbS00017949g0009.1 **Mass:** 42022 **Score:** 108 **Matches:** 5(5) **Sequences:** 4(4)
NbS00017949g0009.1 protein AED:0.29 eAED:0.29 QI:81|1|1|1|0.66|0.75|4|419|386; (*GB) gi|78191448|gb|ABB29945.1| (e_value=0.0) ADP/ATP translocator-like [Solanum tuberosum];; (*SWP) sp|P25083|ADT1_S
NbS00020014g0003.1 **Mass:** 44842 **Score:** 108 **Matches:** 5(5) **Sequences:** 4(4)
NbS00020014g0003.1 protein AED:0.26 eAED:0.29 QI:0|0.66|0.75|1|1|1|4|459|410; (*GB) gi|78191448|gb|ABB29945.1| (e_value=0.0) ADP/ATP translocator-like [Solanum tuberosum];; (*SWP) sp|P25083|ADT1_SO

42. NbS00010663g0016.1 **Mass:** 67391 **Score:** 107 **Matches:** 4(4) **Sequences:** 3(3) **emPAI:** 0.21
NbS00010663g0016.1 protein AED:0.20 eAED:0.20 QI:7|1|0.88|1|1|1|9|298|622; (*GB) gi|224135925|ref|XP_002322195.1| (e_value=0.0) predicted protein [Populus trichocarpa];; (*SWP) sp|Q8W4R3|RH30_ARATH

Query	Observed	Mr(expt)	Mr(calc)	ppm	Miss	Score	Expect	Rank	Unique	Peptide
11905	**572.2983**	**1142.5820**	**1142.5819**	**0.08**	**0**	**45**	**0.0021**	**1**		**R.DDLDNLTLPK.A**
13353	**647.3357**	**1292.6568**	**1292.6612**	**-3.40**	**0**	**56**	**0.00019**	**1**		**R.VTYLVLDEADR.M**
13774	668.8238	1335.6330	1335.6315	1.14	0	53	0.00032	1		R.MLDMGFEPQIR.K
13943	676.8201	1351.6256	1351.6264	-0.63	0	(40)	0.0059	1		R.MLDMGFEPQIR.K

43. NbS00004793g0016.1 **Mass:** 31922 **Score:** 106 **Matches:** 3(3) **Sequences:** 2(2) **emPAI:** 0.35
NbS00004793g0016.1 protein AED:0.15 eAED:0.27 QI:0|0|0|1|1|1|2|0|286; (*SWP) sp|P27492|CB21_TOBAC (e_value=9e-155) Chlorophyll a-b binding protein 16, chloroplastic OS=Nicotiana tabacum GN=CAB16 PE

Query	Observed	Mr(expt)	Mr(calc)	ppm	Miss	Score	Expect	Rank	Unique	Peptide
13682	442.5630	1324.6671	1324.6670	0.12	1	31	0.054	1		K.NRELEVIHCR.W
18493	952.5001	1902.9856	1902.9848	0.40	0	82	3.8e-007	1	U	R.WAMLGALGCVFPEILAR.N
18614	960.4972	1918.9798	1918.9797	0.05	0	(38)	0.0085	1	U	R.WAMLGALGCVFPEILAR.N

44. NbS00000634g0101.1 **Mass:** 15316 **Score:** 102 **Matches:** 8(8) **Sequences:** 4(4) **emPAI:** 1.72
NbS00000634g0101.1 protein AED:0.21 eAED:0.21 QI:0|-1|0|1|-1|1|1|0|136

Query	Observed	Mr(expt)	Mr(calc)	ppm	Miss	Score	Expect	Rank	Unique	Peptide
8714	**344.7211**	**687.4277**	**687.4279**	**-0.28**	**1**	**35**	**0.018**	**1**		**R.KQLATK.A**
9511	**416.2498**	**830.4850**	**830.4861**	**-1.35**	**0**	**57**	**0.00014**	**1**		**K.STELLIR.K** 9517
10897	**339.5380**	**1015.5922**	**1015.5927**	**-0.53**	**0**	**40**	**0.0055**	**1**	**U**	**R.FRPGTVALR.E** 10898 10899
10900	**508.8036**	**1015.5927**	**1015.5927**	**-0.01**	**0**	**(30)**	**0.055**	**1**	**U**	**R.FRPGTVALR.E**
21392	**1146.5680**	**3436.6822**	**3436.6973**	**-4.40**	**0**	**36**	**0.0056**	**1**	**U**	**R.FQSSAVAALQEAAEAYLVGLFEDTNLCAIHAK.R**

Proteins matching the same set of peptides:

NbS00002567g0001.1 **Mass:** 15316 **Score:** 102 **Matches:** 8(8) **Sequences:** 4(4)
NbS00002567g0001.1 protein AED:0.20 eAED:0.20 QI:0|-1|0|1|-1|1|1|0|136; (*GB) gi|300681467|emb|CBH32561.1| (e_value=8e-79) histone H3, expressed [Triticum aestivum];; (*SWP) sp|P68428|H32_WHEAT (e_

NbS00007322g0001.1 **Mass:** 15316 **Score:** 102 **Matches:** 8(8) **Sequences:** 4(4)
NbS00007322g0001.1 protein AED:0.09 eAED:0.09 QI:0|1|0.5|1|0|0|2|203|136; (*GB) gi|300681467|emb|CBH32561.1| (e_value=8e-79) histone H3, expressed [Triticum aestivum];; (*SWP) sp|P68428|H32_WHEAT

NbS00009503g0001.1 **Mass:** 15316 **Score:** 102 **Matches:** 8(8) **Sequences:** 4(4)
NbS00009503g0001.1 protein AED:0.19 eAED:0.19 QI:0|-1|0|1|-1|1|1|0|136; (*GB) gi|300681467|emb|CBH32561.1| (e_value=8e-79) histone H3, expressed [Triticum aestivum];; (*SWP) sp|P68428|H32_WHEAT (e_

NbS00010093g0001.1 **Mass:** 15316 **Score:** 102 **Matches:** 8(8) **Sequences:** 4(4)
NbS00010093g0001.1 protein AED:0.19 eAED:0.19 QI:0|-1|0|1|-1|1|1|0|136; (*SWP) sp|P68428|H32_WHEAT (e_value=3e-80) Histone H3.2 OS=Triticum aestivum PE=1 SV=2;; (*TAIR) AT5G65360.1 (e_value=2e-81)

NbS00014992g0013.1 **Mass:** 56465 **Score:** 102 **Matches:** 8(8) **Sequences:** 4(4)
NbS00014992g0013.1 protein AED:0.12 eAED:0.14 QI:0|0|0|0.66|1|1|3|0|505; (*GB) gi|351722020|ref|NP_001238509.1| (e_value=2e-72) uncharacterized protein LOC100306416 [Glycine max];; (*SWP) sp|P6842[illegible]

NbS00020349g0004.1 **Mass:** 14009 **Score:** 102 **Matches:** 8(8) **Sequences:** 4(4)
NbS00020349g0004.1 protein AED:0.03 eAED:0.03 QI:0|-1|0|1|-1|1|1|0|125; (*SWP) sp|P68428|H32_WHEAT (e_value=1e-72) Histone H3.2 OS=Triticum aestivum PE=1 SV=2;; (*TAIR) AT5G65360.1 (e_value=1e-73)

NbS00026286g0015.1 **Mass:** 20246 **Score:** 102 **Matches:** 8(8) **Sequences:** 4(4)
NbS00026286g0015.1 protein AED:0.06 eAED:0.06 QI:0|0|0|1|0|0|2|0|178; (*GB) gi|156350379|ref|XP_001622258.1| (e_value=8e-77) predicted protein [Nematostella vectensis];; (*SWP) sp|P68428|H32_WHEAT

---

45. NbS00003380g0115.1 **Mass:** 26058 **Score:** 102 **Matches:** 4(4) **Sequences:** 3(3) **emPAI:** 0.44
NbS00003380g0115.1 protein AED:0.12 eAED:0.12 QI:355|0.66|0.85|1|0.33|0.42|7|134|238

Query	Observed	Mr(expt)	Mr(calc)	ppm	Miss	Score	Expect	Rank	Unique	Peptide
11475	546.2915	1090.5685	1090.5658	2.40	0	34	0.043	1		K.NPALYGELSK.G
12712	618.3084	1234.6021	1234.6016	0.46	0	53	0.00036	1		R.NIANMVPAYDK.E
18427	946.5195	1891.0244	1891.0203	2.14	0	40	0.0038	1	U	K.EAVNVSLGNLLTYPFVR.E 18426

---

46. NbS00010743g0014.1 **Mass:** 32611 **Score:** 102 **Matches:** 6(6) **Sequences:** 6(6) **emPAI:** 0.79
NbS00010743g0014.1 protein AED:0.24 eAED:0.24 QI:0|1|1|1|1|1|8|290|288; (*GB) gi|77416949|gb|ABA81870.1| (e_value=2e-155) unknown [Solanum tuberosum];; (*SWP) sp|P43333|RU2A_ARATH (e_value=2e-117)

Query	Observed	Mr(expt)	Mr(calc)	ppm	Miss	Score	Expect	Rank	Unique	Peptide
12097	583.3047	1164.5948	1164.5927	1.80	0	34	0.035	1		K.LENFPYLNR.L
12767	621.3485	1240.6825	1240.6815	0.74	0	34	0.021	1	U	R.INPNIGEFLPK.L
13123	639.3588	1276.7031	1276.7027	0.33	0	31	0.041	1		K.FLSLLDNNITK.R
15196	750.9052	1499.7958	1499.7943	0.94	0	39	0.0074	1	U	K.AAIVNSQTLEEVAR.L
18201	925.5380	1849.0614	1849.0560	2.89	0	48	0.00028	1	U	R.LTNLVEIDPLASLPNLK.F
18252	930.9737	1859.9328	1859.9265	3.42	0	31	0.052	1		R.SGQLPADLNIGDYDIAAK.K

---

47. NbS00011757g0115.1 **Mass:** 64716 **Score:** 98 **Matches:** 3(3) **Sequences:** 2(2) **emPAI:** 0.16
NbS00011757g0115.1 protein AED:0.33 eAED:0.33 QI:0|0.57|0.37|1|0.85|0.75|8|0|590

Query	Observed	Mr(expt)	Mr(calc)	ppm	Miss	Score	Expect	Rank	Unique	Peptide
12391	599.8424	1197.6703	1197.6717	-1.23	0	63	2.2e-005	1	U	R.SPVLVATDVAAR.G
13774	668.8238	1335.6330	1335.6315	1.14	0	53	0.00032	1		R.MLDMGFEPQIR.K
13943	676.8201	1351.6256	1351.6264	-0.63	0	(40)	0.0059	1		R.MLDMGFEPQIR.K

Proteins matching the same set of peptides:

NbS00015643g0012.1 **Mass:** 82196 **Score:** 98 **Matches:** 3(3) **Sequences:** 2(2)
NbS00015643g0012.1 protein AED:0.18 eAED:0.21 QI:213|1|0.9|1|0.77|0.8|10|0|749; (*SWP) sp|P46942|DB10_NICSY (e_value=0.0) ATP-dependent RNA helicase-like protein DB10 OS=Nicotiana sylvestris PE=2 [illegible]

NbS00023296g0019.1 **Mass:** 77422 **Score:** 98 **Matches:** 3(3) **Sequences:** 2(2)
NbS00023296g0019.1 protein AED:0.21 eAED:0.21 QI:24|0.87|0.66|1|1|1|9|0|708; (*SWP) sp|P46942|DB10_NICSY (e_value=0.0) ATP-dependent RNA helicase-like protein DB10 OS=Nicotiana sylvestris PE=2 SV=[illegible]

NbS00003134g0011.1 **Mass:** 79899 **Score:** 98 **Matches:** 3(3) **Sequences:** 2(2)
NbS00003134g0011.1 protein AED:0.32 eAED:0.33 QI:0|0.75|0.33|0.88|0.75|0.66|9|0|720; (*SWP) sp|P46942|DB10_NICSY (e_value=0.0) ATP-dependent RNA helicase-like protein DB10 OS=Nicotiana sylvestris [illegible]

---

48. NbS00045545g0004.1 **Mass:** 64751 **Score:** 98 **Matches:** 4(4) **Sequences:** 4(4) **emPAI:** 0.22
NbS00045545g0004.1 protein AED:0.13 eAED:0.13 QI:0|0.78|0.73|0.86|0.92|0.86|15|481|616; (*SWP) sp|Q7XTT4|NUCL2_ORYSJ (e_value=6e-73) Nucleolin 2 OS=Oryza sativa subsp. japonica GN=Os04g0620700 PE=[illegible]

Query	Observed	Mr(expt)	Mr(calc)	ppm	Miss	Score	Expect	Rank	Unique	Peptide
10849	505.2694	1008.5243	1008.5240	0.25	0	33	0.034	1	U	R.SEGTTIFVR.G
13412	649.8574	1297.7002	1297.6990	0.91	0	40	0.0052	1	U	K.ALELNGQDLLGR.A
14546	474.5598	1420.6577	1420.6582	-0.38	1	51	0.00048	1	U	R.GFDKNEAEDQIR.S

20261	782.7352	2345.1839	2345.1863	-1.03	0	59	5.2e-005	1	U	K.ALELDGSEVGGYNLTVQEAKPR.G

49. NbS00006116g0019.1 **Mass:** 55149 **Score:** 95 **Matches:** 4(4) **Sequences:** 4(4) **emPAI:** 0.26
NbS00006116g0019.1 protein AED:0.27 eAED:0.27 QI:568|1|1|1|1|1|7|0|473; (*GB) gi|219560127|gb|ACL27272.1| (e_value=0.0) catalase [Nicotiana benthamiana];; (*SWP) sp|P49315|CATA1_NICPL (e_value=0.0)

Query	Observed	Mr(expt)	Mr(calc)	ppm	Miss	Score	Expect	Rank	Unique	Peptide
11854	568.8430	1135.6715	1135.6714	0.11	0	34	0.01	1		R.APGVQTPVIVR.F
8354	635.7800	1269.5454	1269.5513	-4.63	0	31	0.021	1	U	R.DEEIDYFPSR.Y
15159	748.4188	1494.8230	1494.8194	2.37	0	39	0.0061	1	U	R.LGPNYLQLPANAPK.C
19064	992.9999	1983.9853	1983.9843	0.51	0	45	0.0016	1	U	R.EGNFDLVGNNFPVFFIR.D

Proteins matching the same set of peptides:
NbS00012784g0015.1 **Mass:** 51219 **Score:** 95 **Matches:** 4(4) **Sequences:** 4(4)
NbS00012784g0015.1 protein AED:0.24 eAED:0.24 QI:310|0.71|0.75|0.87|0.85|0.87|8|0|437; (*GB) gi|2459684|gb|AAB71764.1| (e_value=0.0) catalase 1 [Nicotiana tabacum];; (*SWP) sp|P49315|CATA1_NICPL (e

50. sp|TRYP_PIG| **Mass:** 25078 **Score:** 95 **Matches:** 3(3) **Sequences:** 1(1) **emPAI:** 0.13
sp|TRYP_PIG|

Query	Observed	Mr(expt)	Mr(calc)	ppm	Miss	Score	Expect	Rank	Unique	Peptide
9578	421.7584	841.5023	841.5022	0.17	0	55	0.00015	1	U	R.VATVSLPR.S 9575 9576

Proteins matching the same set of peptides:
gi|3318722|pdb|1AN1|E **Mass:** 24142 **Score:** 95 **Matches:** 3(3) **Sequences:** 1(1)
gi|3318722|pdb|1AN1|E Chain E, Leech-Derived Tryptase InhibitorTRYPSIN COMPLEX

51. NbS00002621g0212.1 **Mass:** 26223 **Score:** 95 **Matches:** 4(3) **Sequences:** 3(2) **emPAI:** 0.43
NbS00002621g0212.1 protein AED:0.19 eAED:0.19 QI:122|0.8|1|1|1|1|6|306|255

Query	Observed	Mr(expt)	Mr(calc)	ppm	Miss	Score	Expect	Rank	Unique	Peptide
12874	626.8693	1251.7241	1251.7227	1.14	0	58	4.4e-005	1	U	R.ILALNASYFLK.A 12873
16867	819.4128	1636.8111	1636.8056	3.35	1	30	0.059	1	U	R.ISVQNEDGTKVEYR.V
18440	632.0314	1893.0725	1893.0724	0.05	0	30	0.021	1	U	K.LAAAVLGGVDDIWIKPGAK.V

Proteins matching the same set of peptides:
NbS00030218g0006.1 **Mass:** 26271 **Score:** 95 **Matches:** 4(3) **Sequences:** 3(2)
NbS00030218g0006.1 protein AED:0.28 eAED:0.28 QI:126|0.83|1|1|0.83|0.71|7|182|255; (*SWP) sp|Q22053|FBRL_CAEEL (e_value=1e-70) rRNA 2'-O-methyltransferase fibrillarin OS=Caenorhabditis elegans GN=

52. NbS00006644g0116.1 **Mass:** 121848 **Score:** 93 **Matches:** 5(5) **Sequences:** 5(5) **emPAI:** 0.14
NbS00006644g0116.1 protein AED:0.00 eAED:0.00 QI:364|1|1|1|1|1|3|541|1093

Query	Observed	Mr(expt)	Mr(calc)	ppm	Miss	Score	Expect	Rank	Unique	Peptide
13460	651.8625	1301.7105	1301.7092	1.05	0	45	0.002	1	U	K.NIFSAVQLPTGR.F
13936	676.3353	1350.6561	1350.6568	-0.52	0	44	0.0027	1	U	R.WALIDFSSQER.N
16826	816.8694	1631.7242	1631.7216	1.62	0	31	0.028	1	U	R.EYIGEFNENNFTR.R
17376	568.9578	1703.8515	1703.8519	-0.25	1	37	0.013	1	U	R.DKLFADNPGQFPIDK.T
19889	742.6985	2225.0738	2225.0641	4.39	1	32	0.032	1	U	K.LFADNPGQFPIDKTAYDGEK.N

53. NbS00020769g0006.1 **Mass:** 26377 **Score:** 92 **Matches:** 3(3) **Sequences:** 3(3) **emPAI:** 0.43
NbS00020769g0006.1 protein AED:0.25 eAED:0.25 QI:307|0.66|1|1|0.66|0.75|4|448|250; (*GB) gi|84620804|gb|ABC59516.1| (e_value=5e-102) chloroplast photosystem II 22 kDa component [Nicotiana benthami

Query	Observed	Mr(expt)	Mr(calc)	ppm	Miss	Score	Expect	Rank	Unique	Peptide
14495	708.3572	1414.6999	1414.6980	1.34	0	53	0.00034	1	U	K.FIDDPTPPTGLDK.A
16077	792.8988	1583.7831	1583.7832	-0.07	0	32	0.034	1		K.VEDGIFGTSGGIGFTK.Q
17673	874.9847	1747.9548	1747.9542	0.33	0	49	0.00057	1		R.VAMIGFAASLLGEAITGK.G

54. NbS00000681g0006.1 **Mass:** 33521 **Score:** 91 **Matches:** 3(3) **Sequences:** 3(3) **emPAI:** 0.33
NbS00000681g0006.1 protein AED:0.29 eAED:0.30 QI:89|0.75|0.88|1|0.75|0.77|9|282|301; (*GB) gi|358248282|ref|NP_001239854.1| (e_value=7e-62) uncharacterized protein LOC100818986 [Glycine max];; (*T

Query	Observed	Mr(expt)	Mr(calc)	ppm	Miss	Score	Expect	Rank	Unique	Peptide
12899	628.8279	1255.6413	1255.6408	0.42	0	52	0.00039	1	U	R.VETPIDANEIR.I

16981	827.4346	1652.8547	1652.8522	1.52	0	45	0.0018	1	U	K.NNIGYQPPIPADQVK.V
21453	1226.9769	3677.9090	3677.9040	1.35	0	45	0.0005	1		R.IVGLHQITSITSTDITDTWEPLEEGLLPLETTR.H

Proteins matching the same set of peptides:
NbS00014238g0013.1 **Mass:** 27940 **Score:** 91 **Matches:** 3(3) **Sequences:** 3(3)
NbS00014238g0013.1 protein AED:0.37 eAED:0.37 QI:89|1|1|1|0.87|0.88|9|404|254; (*GB) gi|358248282|ref|NP_001239854.1| (e_value=1e-64) uncharacterized protein LOC100818986 [Glycine max];; (*SWP) sp

55. NbS00021398g0012.1 **Mass:** 92487 **Score:** 88 **Matches:** 4(4) **Sequences:** 4(4) **emPAI:** 0.15
NbS00021398g0012.1 protein AED:0.33 eAED:0.35 QI:0|0.81|0.75|1|0.81|0.75|12|0|838; (*GB) gi|225450401|ref|XP_002278318.1| (e_value=0.0) PREDICTED: DEAD-box ATP-dependent RNA helicase 3, chloroplast

Query	Observed	Mr(expt)	Mr(calc)	ppm	Miss	Score	Expect	Rank	Unique	Peptide
12268	591.3425	1180.6704	1180.6703	0.06	0	33	0.02	1	U	K.LYALLTATSK.R
13049	635.8729	1269.7312	1269.7292	1.54	0	50	0.00042	1	U	R.LIDLINNNTLK.L
13666	661.8843	1321.7540	1321.7493	3.55	0	41	0.003	1	U	R.TILSDLVTVYAK.G
18828	980.9984	1959.9822	1959.9789	1.65	0	30	0.05	1	U	K.YLNNPLTIDLVGDQEEK.L

56. NbS00001988g0005.1 **Mass:** 66344 **Score:** 85 **Matches:** 5(5) **Sequences:** 4(4) **emPAI:** 0.27
NbS00001988g0005.1 protein AED:0.30 eAED:0.30 QI:0|1|0.83|1|1|1|6|0|610; (*GB) gi|225441549|ref|XP_002281113.1| (e_value=0.0) PREDICTED: DEAD-box ATP-dependent RNA helicase 37-like [Vitis vinifera]

Query	Observed	Mr(expt)	Mr(calc)	ppm	Miss	Score	Expect	Rank	Unique	Peptide
10442	479.2423	956.4701	956.4716	-1.59	0	33	0.027	1	U	K.FSYQTGVR.V
11522	549.3195	1096.6245	1096.6241	0.38	0	41	0.0031	1	U	R.GVDILVATPGR.L
12323	594.8315	1187.6485	1187.6510	-2.08	0	33	0.035	1	U	R.VGSSTDLILQR.V
13774	668.8238	1335.6330	1335.6315	1.14	0	53	0.00032	1		R.MLDMGFEPQIR.R
13943	676.8201	1351.6256	1351.6264	-0.63	0	(40)	0.0059	1		R.MLDMGFEPQIR.R

57. NbS00001538g0002.1 **Mass:** 88204 **Score:** 85 **Matches:** 2(2) **Sequences:** 2(2) **emPAI:** 0.08
NbS00001538g0002.1 protein AED:0.19 eAED:0.19 QI:248|1|1|1|1|1|11|261|788; (*SWP) sp|Q7TP47|HNRPQ_RAT (e_value=2e-31) Heterogeneous nuclear ribonucleoprotein Q OS=Rattus norvegicus GN=Syncrip PE=2

Query	Observed	Mr(expt)	Mr(calc)	ppm	Miss	Score	Expect	Rank	Unique	Peptide
15931	783.8380	1565.6614	1565.6594	1.25	0	60	2.2e-005	1	U	R.GSDVGGSSYSSAYSSR.G
19339	1034.9998	2067.9850	2067.9823	1.28	0	46	0.0012	1	U	K.VSFADSFIDPGDEIMAQVK.T

58. NbS00001559g0021.1 **Mass:** 90961 **Score:** 84 **Matches:** 3(3) **Sequences:** 3(3) **emPAI:** 0.11
NbS00001559g0021.1 protein AED:0.25 eAED:0.31 QI:138|0.5|0.53|1|0.85|0.93|15|300|837; (*GB) gi|356513635|ref|XP_003525517.1| (e_value=0.0) PREDICTED: DEAD-box ATP-dependent RNA helicase 30-like [G

Query	Observed	Mr(expt)	Mr(calc)	ppm	Miss	Score	Expect	Rank	Unique	Peptide
11905	572.2983	1142.5820	1142.5819	0.08	0	45	0.0021	1		R.DDLDNLTLPK.A
13353	647.3357	1292.6568	1292.6612	-3.40	0	56	0.00019	1		R.VTYLVLDEADR.M
14690	719.8535	1437.6925	1437.6922	0.19	0	36	0.017	1	U	R.ANISSTCIYGGAPK.G

59. NbS00021832g0023.1 **Mass:** 9182 **Score:** 83 **Matches:** 2(2) **Sequences:** 1(1) **emPAI:** 0.38
NbS00021832g0023.1 protein AED:0.35 eAED:0.35 QI:0|0|0|1|0|0|2|0|80; (*GB) gi|305671961|gb|ADM63350.1| (e_value=2e-44) photosystem II cp47 protein [Aralidium pinnatifidum];; (*SWP) sp|A4QLM0|PSBB_I

Query	Observed	Mr(expt)	Mr(calc)	ppm	Miss	Score	Expect	Rank	Unique	Peptide
13329	645.8387	1289.6628	1289.6615	0.99	0	67	1.6e-005	1	U	R.AQLGEIFELDR.A 13328

60. NbS00001259g0009.1 **Mass:** 35402 **Score:** 83 **Matches:** 4(3) **Sequences:** 4(3) **emPAI:** 0.43
NbS00001259g0009.1 protein AED:0.04 eAED:0.04 QI:128|0.75|0.77|1|0.75|0.88|9|0|318; (*GB) gi|357505379|ref|XP_003622978.1| (e_value=2e-55) Ribonuclease P protein subunit p25 [Medicago truncatula];;

Query	Observed	Mr(expt)	Mr(calc)	ppm	Miss	Score	Expect	Rank	Unique	Peptide
10446	479.3018	956.5891	956.5906	-1.59	0	40	0.0038	1		K.TVAIAEIIK.E
14721	722.3306	1442.6467	1442.6426	2.84	0	32	0.026	1	U	R.AVYNVGNEDSYGR.E
15457	765.3967	1528.7788	1528.7773	0.98	0	31	0.055	1	U	R.NYISYATSLLQEK.R
16908	822.4012	1642.7879	1642.7852	1.67	0	52	0.00037	1	U	R.YNYQPQQQQPPPR.H

61. NbS00017935g0003.1 **Mass:** 31122 **Score:** 83 **Matches:** 5(5) **Sequences:** 4(4) **emPAI:** 0.50
NbS00017935g0003.1 protein AED:0.00 eAED:0.00 QI:240|1|0.5|1|1|1|2|0|284; (*SWP) sp|Q9XF88|CB4B_ARATH (e_value=4e-162) Chlorophyll a-b binding protein CP29.2, chloroplastic OS=Arabidopsis thaliana

Query	Observed	Mr(expt)	Mr(calc)	ppm	Miss	Score	Expect	Rank	Unique	Peptide

10684	493.7976	985.5807	985.5808	-0.10	0	36	0.016	1	U	K.ATLQLAEIK.H 10685
10999	515.2880	1028.5615	1028.5614	0.05	0	43	0.0043	1	U	K.NLAGDIIGTR.T
17713	878.4397	1754.8648	1754.8628	1.17	0	47	0.001	1	U	K.STPFQPYSEVFGLQR.F
19128	997.4999	1992.9853	1992.9833	1.02	0	42	0.0036	1	U	R.LYPGGSFFDPLGLAADPEK.K

Proteins matching the same set of peptides:
NbS00027305g0008.1 **Mass:** 31084 **Score:** 83 **Matches:** 5(5) **Sequences:** 4(4)
NbS00027305g0008.1 protein AED:0.31 eAED:0.31 QI:0|1|0.5|1|1|1|2|0|284; (*GB) gi|110377766|gb|ABG73415.1| (e_value=0.0) chloroplast pigment-binding protein CP29 [Nicotiana tabacum];; (*SWP) sp|Q9XI

---

62. NbS00022486g0001.1 **Mass:** 20485 **Score:** 82 **Matches:** 4(4) **Sequences:** 4(4) **emPAI:** 0.83
NbS00022486g0001.1 protein AED:0.21 eAED:0.21 QI:0|1|0.66|1|1|1|3|0|181; (*GB) gi|132118|sp|P26573.1|RBS8_NICPL (e_value=4e-116) RecName: Full=Ribulose bisphosphate carboxylase small chain 8B, chlo

Query	Observed	Mr(expt)	Mr(calc)	ppm	Miss	Score	Expect	Rank	Unique	Peptide
10238	467.2611	932.5076	932.5080	-0.39	0	57	0.00018	1		R.IIGFDNVR.Q
19885	742.3556	2224.0449	2224.0392	2.60	0	34	0.017	1		K.LPMFGCTDATQVLAEVEEAK.K
20247	779.7203	2336.1390	2336.1392	-0.09	1	31	0.041	1		K.LPMFGCTDATQVLAEVEEAKK.A
21143	1001.1924	3000.5553	3000.5583	-0.98	1	30	0.025	1	U	K.KYETLSYLPDLSQEQLLSEVEYLLK.N

Proteins matching the same set of peptides:
NbS00027369g0007.1 **Mass:** 20455 **Score:** 82 **Matches:** 4(4) **Sequences:** 4(4)
NbS00027369g0007.1 protein AED:0.16 eAED:0.16 QI:0|1|0.66|1|1|1|3|0|181; (*GB) gi|132118|sp|P26573.1|RBS8_NICPL (e_value=2e-114) RecName: Full=Ribulose bisphosphate carboxylase small chain 8B, chlo
NbS00041372g0008.1 **Mass:** 22328 **Score:** 82 **Matches:** 4(4) **Sequences:** 4(4)
NbS00041372g0008.1 protein AED:0.22 eAED:0.22 QI:8|0.66|0.5|1|1|1|4|0|197; (*SWP) sp|P26573|RBS8_NICPL (e_value=5e-110) Ribulose bisphosphate carboxylase small chain 8B, chloroplastic OS=Nicotiana

---

63. NbS00004956g0015.1 **Mass:** 9825 **Score:** 81 **Matches:** 4(4) **Sequences:** 2(2) **emPAI:** 1.47
NbS00004956g0015.1 protein AED:0.25 eAED:0.30 QI:0|0|0|1|0|0|2|0|84; (*GB) gi|42718201|gb|AAS38532.1| (e_value=7e-41) ribulose-1,5-bisphosphate carboxylase/oxygenase large subunit [Hygrolembidium a

Query	Observed	Mr(expt)	Mr(calc)	ppm	Miss	Score	Expect	Rank	Unique	Peptide
10050	455.7261	909.4377	909.4378	-0.18	0	47	0.0012	1	U	R.AVYECLR.G 10052
14883	489.2555	1464.7447	1464.7474	-1.80	0	(36)	0.019	1	U	K.TFQGPPHGIQVER.D
14890	733.3820	1464.7494	1464.7474	1.36	0	44	0.0026	1	U	K.TFQGPPHGIQVER.D

---

64. NbS00016159g0006.1 **Mass:** 32702 **Score:** 79 **Matches:** 3(3) **Sequences:** 3(3) **emPAI:** 0.34
NbS00016159g0006.1 protein AED:0.34 eAED:0.36 QI:356|0.57|0.5|1|0.71|0.62|8|0|302; (*GB) gi|115473|sp|P27141.1|CAHC_TOBAC (e_value=3e-179) RecName: Full=Carbonic anhydrase, chloroplastic; AltName:

Query	Observed	Mr(expt)	Mr(calc)	ppm	Miss	Score	Expect	Rank	Unique	Peptide
11475	546.2915	1090.5685	1090.5658	2.40	0	34	0.043	1		K.NPALYGELSK.G
12712	618.3084	1234.6021	1234.6016	0.46	0	53	0.00036	1		R.NIANMVPAYDK.T
19963	1127.0426	2252.0707	2252.0671	1.58	0	33	0.023	1	U	K.GLMSLPADGSESTAFIEDWVK.I

---

65. NbS00014345g0007.1 **Mass:** 127872 **Score:** 78 **Matches:** 3(3) **Sequences:** 3(3) **emPAI:** 0.08
NbS00014345g0007.1 protein AED:0.10 eAED:0.10 QI:698|0.5|0.61|0.76|0.75|0.76|13|0|1156; (*GB) gi|359472595|ref|XP_002281257.2| (e_value=0.0) PREDICTED: nuclear pore complex protein Nup155-like [Vit

Query	Observed	Mr(expt)	Mr(calc)	ppm	Miss	Score	Expect	Rank	Unique	Peptide
11644	557.2827	1112.5508	1112.5502	0.48	0	32	0.035	1	U	R.SLLEDFFSR.F
15878	779.3906	1556.7666	1556.7682	-1.07	0	35	0.019	1	U	R.VVSGVESVGDEDIPR.A
17218	841.5186	1681.0227	1681.0218	0.50	0	52	4.4e-005	1	U	R.LLLPLWELPVFITK.G

---

66. NbS00036430g0009.1 **Mass:** 48651 **Score:** 77 **Matches:** 2(2) **Sequences:** 2(2) **emPAI:** 0.14
NbS00036430g0009.1 protein AED:0.35 eAED:0.35 QI:0|0|0|1|0|0|4|0|448; (*GB) gi|113170490|ref|YP_717281.1| (e_value=2e-170) Atp1 [Ostreococcus tauri];; (*SWP) sp|B3PQ70|ATPA_RHIE6 (e_value=2e-158) A

Query	Observed	Mr(expt)	Mr(calc)	ppm	Miss	Score	Expect	Rank	Unique	Peptide
10583	486.7774	971.5403	971.5400	0.32	0	44	0.0034	1		R.VVSVGDGIAR.V
12335	595.3251	1188.6356	1188.6350	0.52	0	61	6.1e-005	1	U	R.AAELTSLLESR.I

---

67. NbS00004717g0103.1 **Mass:** 13622 **Score:** 76 **Matches:** 2(2) **Sequences:** 1(1) **emPAI:** 0.25
NbS00004717g0103.1 protein AED:0.03 eAED:0.03 QI:0|-1|0|1|-1|1|1|0|128

Query	Observed	Mr(expt)	Mr(calc)	ppm	Miss	Score	Expect	Rank	Unique	Peptide
9375	407.2633	812.5121	812.5120	0.17	0	58	4.8e-005	1	U	

K.ANVLGIVK.L 4556

Proteins matching the same set of peptides:
NbS00017897g0003.1 **Mass:** 13705 **Score:** 76 **Matches:** 2(2) **Sequences:** 1(1)
NbS00017897g0003.1 protein AED:0.00 eAED:0.00 QI:176|0|0.5|1|0|0.5|2|349|129; (*GB) gi|118488240|gb|ABK95939.1| (e_value=2e-36) unknown [Populus trichocarpa];; (*SWP) sp|P14009|14KD_DAUCA (e_value=

---

68. NbS00028679g0001.1 **Mass:** 34422 **Score:** 76 **Matches:** 2(2) **Sequences:** 2(2) **emPAI:** 0.20
NbS00028679g0001.1 protein AED:0.08 eAED:0.08 QI:281|0.88|0.9|1|0.88|0.8|10|299|305; (*GB) gi|255542956|ref|XP_002512541.1| (e_value=0.0) NAD dependent epimerase/dehydratase, putative [Ricinus comm

Query	Observed	Mr(expt)	Mr(calc)	ppm	Miss	Score	Expect	Rank	Unique	Peptide
15382	759.9122	1517.8099	1517.8089	0.64	0	68	1e-005	1	U	K.DLATAFIQVLGNEK.A
18102	914.9387	1827.8629	1827.8639	-0.56	0	30	0.049	1	U	K.SSLSAEGFDVVYDINGR.E

---

69. NbS00011040g0001.1 **Mass:** 28504 **Score:** 74 **Matches:** 3(3) **Sequences:** 2(2) **emPAI:** 0.25
NbS00011040g0001.1 protein AED:0.00 eAED:0.03 QI:0|1|0.66|1|0|0|3|164|270; (*GB) gi|255578051|ref|XP_002529896.1| (e_value=1e-58) small nuclear ribonucleoprotein-associated protein, putative [Ricir

Query	Observed	Mr(expt)	Mr(calc)	ppm	Miss	Score	Expect	Rank	Unique	Peptide
9864	442.8002	883.5858	883.5855	0.42	0	35	0.005	1	U	R.TLGLVLLR.G
11541	550.7813	1099.5481	1099.5484	-0.31	0	49	0.00091	1	U	K.MLQYINYR.M 7868

Proteins matching the same set of peptides:
NbS00014429g0006.1 **Mass:** 33726 **Score:** 74 **Matches:** 3(3) **Sequences:** 2(2)
NbS00014429g0006.1 protein AED:0.21 eAED:0.23 QI:0|0|0|1|1|1|2|0|322; (*GB) gi|255578055|ref|XP_002529898.1| (e_value=4e-54) small nuclear ribonucleoprotein-associated protein, putative [Ricinus co

---

70. NbS00047378g0007.1 **Mass:** 80254 **Score:** 73 **Matches:** 2(2) **Sequences:** 2(2) **emPAI:** 0.08
NbS00047378g0007.1 protein AED:0.08 eAED:0.08 QI:0|0.9|0.90|1|0.9|0.90|11|3|723; (*GB) gi|255561268|ref|XP_002521645.1| (e_value=0.0) arsenite-resistance protein, putative [Ricinus communis];; (*SW

Query	Observed	Mr(expt)	Mr(calc)	ppm	Miss	Score	Expect	Rank	Unique	Peptide
14304	697.8046	1393.5947	1393.5939	0.59	0	44	0.0011	1		R.FGYDYQPGYER.E
14917	734.9095	1467.8044	1467.8045	-0.08	0	51	0.00036	1	U	R.VQIDVEQAQALVR.K

---

71. NbS00010277g0007.1 **Mass:** 59557 **Score:** 73 **Matches:** 4(3) **Sequences:** 4(3) **emPAI:** 0.17
NbS00010277g0007.1 protein AED:0.15 eAED:0.15 QI:45|1|1|1|0.81|0.75|12|537|515; (*GB) gi|350538893|ref|NP_001234620.1| (e_value=0.0) beta-mannosidase enzyme precursor [Solanum lycopersicum];; (*SWI

Query	Observed	Mr(expt)	Mr(calc)	ppm	Miss	Score	Expect	Rank	Unique	Peptide
9851	441.7275	881.4405	881.4395	1.04	0	31	0.054	1	U	R.FSISWSR.I
11166	526.7716	1051.5287	1051.5298	-1.11	0	45	0.0024	1	U	K.LSFDTGGLSR.E
12077	582.3079	1162.6012	1162.6023	-0.94	0	34	0.038	1	U	R.GPSIWDTFIK.R
16887	820.9260	1639.8374	1639.8359	0.92	0	37	0.012	1	U	R.VVAALGYDTGFFAPGR.C

---

72. NbS00006224g0007.1 **Mass:** 16356 **Score:** 72 **Matches:** 4(3) **Sequences:** 3(2) **emPAI:** 0.46
NbS00006224g0007.1 protein AED:0.32 eAED:0.43 QI:327|0.6|0.66|0.83|0.6|0.66|6|0|143; (*GB) gi|319903976|gb|ADV77190.1| (e_value=2e-51) histone H3 [Boergesenia forbesii];; (*SWP) sp|Q71H73|H33_VITVI

Query	Observed	Mr(expt)	Mr(calc)	ppm	Miss	Score	Expect	Rank	Unique	Peptide
8714	344.7211	687.4277	687.4279	-0.28	1	35	0.018	1		R.KQLATK.A
9511	416.2498	830.4850	830.4861	-1.35	0	57	0.00014	1		K.STELLIR.K 9517
11029	344.8696	1031.5870	1031.5876	-0.62	0	30	0.059	1	U	R.YRPGTVALR.E

---

73. NbS00004238g0018.1 **Mass:** 34571 **Score:** 72 **Matches:** 5(5) **Sequences:** 5(5) **emPAI:** 0.58
NbS00004238g0018.1 protein AED:0.16 eAED:0.16 QI:156|0.87|0.88|1|0.87|0.77|9|398|304; (*GB) gi|77416949|gb|ABA81870.1| (e_value=2e-140) unknown [Solanum tuberosum];; (*SWP) sp|P43333|RU2A_ARATH (e_

Query	Observed	Mr(expt)	Mr(calc)	ppm	Miss	Score	Expect	Rank	Unique	Peptide
12097	583.3047	1164.5948	1164.5927	1.80	0	34	0.035	1		K.LENFPYLNR.L
13123	639.3588	1276.7031	1276.7027	0.33	0	31	0.041	1		K.FLSLLDNNITK.R
15357	757.9134	1513.8122	1513.8100	1.48	0	31	0.047	1	U	K.AAILNSQTLEEVAR.L
18146	920.5265	1839.0384	1839.0353	1.70	0	34	0.0098	1	U	R.LTNLVEIDPLASLSNLK.F
18252	930.9737	1859.9328	1859.9265	3.42	0	31	0.052	1		R.SGQLPADLNIGDYDIAAK.K

---

74. NbS00000485g0008.1 **Mass:** 26716 **Score:** 70 **Matches:** 2(2) **Sequences:** 2(2) **emPAI:** 0.26

NbS00000485g0008.1 protein AED:0.27 eAED:0.27 QI:81|1|1|1|0.8|0.83|6|329|238; (*GB) gi|225442156|ref|XP_002275541.1| (e_value=6e-155) PREDICTED: 40S ribosomal protein S3-3 [Vitis vinifera];; (*SWP)

Query	Observed	Mr(expt)	Mr(calc)	ppm	Miss	Score	Expect	Rank	Unique	Peptide
9289	399.7632	797.5118	797.5123	-0.62	0	34	0.0092	1	U	K.LLGGLAVR.R
8806	712.3400	1422.6654	1422.6627	1.95	0	63	8.4e-005	1	U	R.ELAEDGYSGVEVR.V

Proteins matching the same set of peptides:

NbS00003420g0016.1 **Mass:** 26587 **Score:** 70 **Matches:** 2(2) **Sequences:** 2(2)
NbS00003420g0016.1 protein AED:0.15 eAED:0.15 QI:81|1|1|1|0.8|1|6|333|237; (*GB) gi|358248410|ref|NP_001239621.1| (e_value=5e-156) uncharacterized protein LOC100808705 [Glycine max];; (*SWP) sp|Q9S
NbS00035854g0012.1 **Mass:** 29045 **Score:** 70 **Matches:** 2(2) **Sequences:** 2(2)
NbS00035854g0012.1 protein AED:0.09 eAED:0.09 QI:143|1|0.85|1|0.83|0.71|7|0|260; (*GB) gi|225444782|ref|XP_002279950.1| (e_value=5e-151) PREDICTED: 40S ribosomal protein S3-3 [Vitis vinifera];; (*S

---

75. NbS00027201g0002.1 **Mass:** 20557 **Score:** 70 **Matches:** 4(3) **Sequences:** 4(3) **emPAI:** 0.58
NbS00027201g0002.1 protein AED:0.33 eAED:0.33 QI:0|1|0.75|1|1|1|4|0|180; (*GB) gi|59800169|sp|P69249.1|RBS_TOBAC (e_value=9e-123) RecName: Full=Ribulose bisphosphate carboxylase small chain, chloro

Query	Observed	Mr(expt)	Mr(calc)	ppm	Miss	Score	Expect	Rank	Unique	Peptide
10238	467.2611	932.5076	932.5080	-0.39	0	57	0.00018	1		R.IIGFDNVR.Q
10807	502.7691	1003.5236	1003.5239	-0.33	0	32	0.062	1	U	K.AYPQAWIR.I
19885	742.3556	2224.0449	2224.0392	2.60	0	34	0.017	1		K.LPMFGCTDATQVLAEVEEAK.K
20247	779.7203	2336.1390	2336.1392	-0.09	1	31	0.041	1		K.LPMFGCTDATQVLAEVEEAKK.A

Proteins matching the same set of peptides:

NbS00037852g0006.1 **Mass:** 20576 **Score:** 70 **Matches:** 4(3) **Sequences:** 4(3)
NbS00037852g0006.1 protein AED:0.20 eAED:0.21 QI:0|1|0.75|1|1|1|4|0|180; (*GB) gi|132118|sp|P26573.1|RBS8_NICPL (e_value=7e-123) RecName: Full=Ribulose bisphosphate carboxylase small chain 8B, chlo

---

76. NbS00002044g0005.1 **Mass:** 29321 **Score:** 69 **Matches:** 2(1) **Sequences:** 2(1) **emPAI:** 0.24
NbS00002044g0005.1 protein AED:0.28 eAED:0.28 QI:0|1|0.66|1|1|1|3|0|273; (*GB) gi|226872|prf||1609235A (e_value=3e-155) chlorophyll a/b binding protein;; (*SWP) sp|P27522|CB13_SOLLC (e_value=4e-15

Query	Observed	Mr(expt)	Mr(calc)	ppm	Miss	Score	Expect	Rank	Unique	Peptide
8423	646.3400	1290.6654	1290.6720	-5.11	0	37	0.06	1	U	K.WLAYGEIINGR.F
16808	815.4556	1628.8967	1628.8960	0.45	0	58	6.8e-005	1	U	R.FAMLGAAGAIAPEILGK.A

Proteins matching the same set of peptides:

NbS00006820g0015.1 **Mass:** 29146 **Score:** 69 **Matches:** 2(1) **Sequences:** 2(1)
NbS00006820g0015.1 protein AED:0.07 eAED:0.08 QI:0|1|0.66|1|1|1|3|0|273; (*GB) gi|226872|prf||1609235A (e_value=1e-149) chlorophyll a/b binding protein;; (*SWP) sp|P27522|CB13_SOLLC (e_value=3e-15
NbS00010402g0001.1 **Mass:** 22688 **Score:** 69 **Matches:** 2(1) **Sequences:** 2(1)
NbS00010402g0001.1 protein AED:0.03 eAED:0.03 QI:315|0.5|0.66|1|0|0.66|3|354|209; (*GB) gi|226872|prf||1609235A (e_value=3e-129) chlorophyll a/b binding protein;; (*SWP) sp|P27522|CB13_SOLLC (e_va
NbS00021892g0003.1 **Mass:** 26702 **Score:** 69 **Matches:** 2(1) **Sequences:** 2(1)
NbS00021892g0003.1 protein AED:0.04 eAED:0.04 QI:191|1|1|1|0.5|0.4|5|817|252; (*GB) gi|226872|prf||1609235A (e_value=2e-124) chlorophyll a/b binding protein;; (*SWP) sp|P27522|CB13_SOLLC (e_value=

---

77. NbS00005969g0002.1 **Mass:** 63583 **Score:** 68 **Matches:** 3(3) **Sequences:** 3(3) **emPAI:** 0.16
NbS00005969g0002.1 protein AED:0.07 eAED:0.07 QI:0|0.5|0.55|0.88|1|1|9|177|561; (*GB) gi|225456270|ref|XP_002283518.1| (e_value=0.0) PREDICTED: nucleolar protein 56-like [Vitis vinifera];; (*SWP) s

Query	Observed	Mr(expt)	Mr(calc)	ppm	Miss	Score	Expect	Rank	Unique	Peptide
10477	481.2713	960.5280	960.5280	-0.03	0	40	0.0093	1	U	K.FSLGLAEPK.L
17258	843.9406	1685.8666	1685.8658	0.44	0	39	0.0069	1	U	K.ASMGQDLSPVDLINVK.M
17558	867.8985	1733.7824	1733.7818	0.35	0	40	0.0041	1	U	R.LDCFLDTSTTTFGEK.L

Proteins matching the same set of peptides:

NbS00023900g0001.1 **Mass:** 64240 **Score:** 68 **Matches:** 3(3) **Sequences:** 3(3)
NbS00023900g0001.1 protein AED:0.11 eAED:0.11 QI:0|0.85|0.75|1|1|1|8|178|567; (*SWP) sp|O94514|NOP56_SCHPO (e_value=0.0) Nucleolar protein 56 OS=Schizosaccharomyces pombe (strain 972 / ATCC 24843)

---

78. NbS00010087g0103.1 **Mass:** 25594 **Score:** 68 **Matches:** 2(2) **Sequences:** 2(2) **emPAI:** 0.28
NbS00010087g0103.1 protein AED:0.35 eAED:0.35 QI:0|0|0|0.66|1|1|3|0|235

Query	Observed	Mr(expt)	Mr(calc)	ppm	Miss	Score	Expect	Rank	Unique	Peptide
12389	599.3511	1196.6876	1196.6877	-0.10	0	39	0.0039	1	U	K.DAGVIAGLNVLR.I
17087	830.4522	1658.8899	1658.8879	1.19	0	51	0.00043	1		R.IINEPTAAAIAYGLDK.K

Proteins matching the same set of peptides:

NbS00016136g0003.1 **Mass:** 58764 **Score:** 68 **Matches:** 2(2) **Sequences:** 2(2)
NbS00016136g0003.1 protein AED:0.24 eAED:0.24 QI:0|0|0|1|1|1|3|0|533; (*GB) gi|392465167|dbj|BAM24707.1| (e_value=0.0) Heat shock protein 70 [Nicotiana tabacum];; (*SWP) sp|P27322|HSP72_SOLLC (e_va
gi|109070524|ref|XP_001113329.1| **Mass:** 70722 **Score:** 68 **Matches:** 2(2) **Sequences:** 2(2)
gi|109070524|ref|XP_001113329.1| PREDICTED: heat shock 70 kDa protein 1-like isoform 2 [Macaca mulatta]

79. NbC24305910g0003.1 **Mass:** 11284 **Score:** 68 **Matches:** 3(3) **Sequences:** 3(3) **emPAI:** 1.22
NbC24305910g0003.1 protein AED:0.03 eAED:0.03 QI:0|1|0.5|1|1|1|2|0|101; (*GB) gi|12643758|sp|Q40565.1|RCA2_TOBAC (e_value=6e-65) RecName: Full=Ribulose bisphosphate carboxylase/oxygenase activase 2

Query	Observed	Mr(expt)	Mr(calc)	ppm	Miss	Score	Expect	Rank	Unique	Peptide
17272	845.4299	1688.8453	1688.8410	2.56	0	45	0.0018	1		K.LLNSFDGPPTFEQPK.M
18391	941.9896	1881.9646	1881.9625	1.09	0	41	0.0044	1		K.IVDTFPGQSIDFFGALR.A
18506	953.9849	1905.9552	1905.9506	2.41	0	36	0.015	1		K.LLEYGNMLVQEQENVK.R

80. NbS00012584g0001.1 **Mass:** 40901 **Score:** 68 **Matches:** 4(4) **Sequences:** 3(3) **emPAI:** 0.26
NbS00012584g0001.1 protein AED:0.12 eAED:0.12 QI:0|-1|0|1|-1|1|1|0|370; (*GB) gi|231610|sp|P29790.1|ATPG_TOBAC (e_value=0.0) RecName: Full=ATP synthase gamma chain, chloroplastic; AltName: Full=F-A

Query	Observed	Mr(expt)	Mr(calc)	ppm	Miss	Score	Expect	Rank	Unique	Peptide
12202	**588.8176**	**1175.6206**	**1175.6186**	**1.67**	**0**	**36**	**0.021**	**1**	**U**	**K.FLEGSNLPTAK.D**
14005	**679.8682**	**1357.7219**	**1357.7201**	**1.33**	**0**	**40**	**0.0073**	**1**	**U**	**R.ALQESLASELAAR.M** 14004
16986	**552.2661**	**1653.7763**	**1653.7702**	**3.73**	**1**	**31**	**0.039**	**1**	**U**	**R.MSAMSAATDNASELKK.N**

Proteins matching the same set of peptides:
NbS00023165g0008.1 **Mass:** 43624 **Score:** 68 **Matches:** 4(4) **Sequences:** 3(3)
NbS00023165g0008.1 protein AED:0.11 eAED:0.12 QI:0|0|0|1|0|0.5|2|0|394; (*GB) gi|231610|sp|P29790.1|ATPG_TOBAC (e_value=0.0) RecName: Full=ATP synthase gamma chain, chloroplastic; AltName: Full=F-A

81. NbS00036785g0006.1 **Mass:** 38193 **Score:** 68 **Matches:** 1(1) **Sequences:** 1(1) **emPAI:** 0.09
NbS00036785g0006.1 protein AED:0.27 eAED:0.32 QI:267|0.88|0.8|1|0.77|0.6|10|263|336; (*GB) gi|356572914|ref|XP_003554610.1| (e_value=0.0) PREDICTED: uncharacterized protein At1g09340, chloroplastic

Query	Observed	Mr(expt)	Mr(calc)	ppm	Miss	Score	Expect	Rank	Unique	Peptide
15382	759.9122	1517.8099	1517.8453	-23.33	1	68	1e-005	1	U	K.DLATAFIKVLGNEK.A

82. NbS00027670g0006.1 **Mass:** 59238 **Score:** 67 **Matches:** 3(3) **Sequences:** 3(3) **emPAI:** 0.18
NbS00027670g0006.1 protein AED:0.24 eAED:0.24 QI:614|0.93|0.93|1|1|1|16|163|536; (*SWP) sp|P50433|GLYM_SOLTU (e_value=0.0) Serine hydroxymethyltransferase, mitochondrial OS=Solanum tuberosum PE=2 S

Query	Observed	Mr(expt)	Mr(calc)	ppm	Miss	Score	Expect	Rank	Unique	Peptide
11477	**546.3143**	**1090.6141**	**1090.6135**	**0.59**	**0**	**35**	**0.018**	**1**	**U**	**K.LIVAGASAYAR.L**
17889	**894.4221**	**1786.8297**	**1786.8261**	**2.01**	**0**	**30**	**0.041**	**1**	**U**	**R.LNESTGYIDYDQLEK.S**
20319	**1189.6219**	**2377.2293**	**2377.2264**	**1.25**	**0**	**43**	**0.0019**	**1**	**U**	**K.QLNAPLEEVDPEIADIIELEK.A**

83. NbS00042812g0008.1 **Mass:** 20462 **Score:** 67 **Matches:** 3(3) **Sequences:** 3(3) **emPAI:** 0.58
NbS00042812g0008.1 protein AED:0.19 eAED:0.19 QI:0|1|1|1|1|1|4|386|180; (*GB) gi|132118|sp|P26573.1|RBS8_NICPL (e_value=3e-119) RecName: Full=Ribulose bisphosphate carboxylase small chain 8B, chlor

Query	Observed	Mr(expt)	Mr(calc)	ppm	Miss	Score	Expect	Rank	Unique	Peptide
10238	467.2611	932.5076	932.5080	-0.39	0	57	0.00018	1		R.IIGFDNVR.Q
11133	**524.7642**	**1047.5138**	**1047.5138**	**0.00**	**0**	**32**	**0.05**	**1**	**U**	**K.EYPQAWVR.I**
19992	**755.7147**	**2264.1222**	**2264.1181**	**1.80**	**1**	**33**	**0.026**	**1**	**U**	**K.LPMFGCTDATQVLAEVGEAKK.E**

Proteins matching the same set of peptides:
NbC25292626g0004.1 **Mass:** 18446 **Score:** 67 **Matches:** 3(3) **Sequences:** 3(3)
NbC25292626g0004.1 protein AED:0.08 eAED:0.08 QI:0|1|0.5|1|1|1|4|0|159; (*GB) gi|132118|sp|P26573.1|RBS8_NICPL (e_value=2e-112) RecName: Full=Ribulose bisphosphate carboxylase small chain 8B, chlor

84. NbS00009983g0008.1 **Mass:** 42145 **Score:** 67 **Matches:** 2(2) **Sequences:** 2(2) **emPAI:** 0.16
NbS00009983g0008.1 protein AED:0.11 eAED:0.11 QI:0|0|0|1|0|0.5|2|0|384; (*GB) gi|108864705|gb|ABG22608.1| (e_value=0.0) Heat shock cognate 70 kDa protein, putative, expressed [Oryza sativa Japonica

Query	Observed	Mr(expt)	Mr(calc)	ppm	Miss	Score	Expect	Rank	Unique	Peptide
8281	**616.3300**	**1230.6454**	**1230.6391**	**5.20**	**0**	**41**	**0.017**	**1**	**U**	**K.DAGVISGLNVMR.I**
17087	830.4522	1658.8899	1658.8879	1.19	0	51	0.00043	1		R.IINEPTAAAIAYGLDK.K

Proteins matching the same set of peptides:
NbS00022307g0013.1 **Mass:** 58674 **Score:** 67 **Matches:** 2(2) **Sequences:** 2(2)

NbS00022307g0013.1 protein AED:0.22 eAED:0.22 QI:0|0|0|1|0.66|0.5|4|0|536; (*GB) gi|108864705|gb|ABG22608.1| (e_value=0.0) Heat shock cognate 70 kDa protein, putative, expressed [Oryza sativa Japor

NbS00025223g0018.1 **Mass:** 65424 **Score:** 67 **Matches:** 2(2) **Sequences:** 2(2)

NbS00025223g0018.1 protein AED:0.09 eAED:0.10 QI:0|0|0|0.6|0.75|0.6|5|0|591; (*GB) gi|108864706|gb|ABG22609.1| (e_value=0.0) Heat shock cognate 70 kDa protein, putative, expressed [Oryza sativa Jap

NbS00027735g0001.1 **Mass:** 70349 **Score:** 67 **Matches:** 2(2) **Sequences:** 2(2)

NbS00027735g0001.1 protein AED:0.18 eAED:0.18 QI:0|0|0|0.5|1|1|2|0|633; (*GB) gi|123650|sp|P09189.1|HSP7C_PETHY (e_value=0.0) RecName: Full=Heat shock cognate 70 kDa protein;; (*SWP) sp|P09189|HSP

NbS00037714g0003.1 **Mass:** 53453 **Score:** 67 **Matches:** 2(2) **Sequences:** 2(2)

NbS00037714g0003.1 protein AED:0.12 eAED:0.12 QI:0|0|0|0.75|1|1|4|0|487; (*GB) gi|38325815|gb|AAR17080.1| (e_value=0.0) heat shock protein 70-3 [Nicotiana tabacum];; (*SWP) sp|P27322|HSP72_SOLLC (e

---

85. NbS00059497g0003.1 **Mass:** 77510 **Score:** 65 **Matches:** 3(3) **Sequences:** 1(1) **emPAI:** 0.04

NbS00059497g0003.1 protein AED:0.17 eAED:0.17 QI:0|1|1|1|1|1|3|550|697; (*SWP) sp|Q653H7|ARFR_ORYSJ (e_value=0.0) Auxin response factor 18 OS=Oryza sativa subsp. japonica GN=ARF18 PE=2 SV=1;; (*TA

Query	Observed	Mr(expt)	Mr(calc)	ppm	Miss	Score	Expect	Rank	Unique	Peptide
9576	421.7583	841.5020	841.5022	-0.19	0	51	0.00044	1	U	R.GLTVSLPR.D 9575 9578

---

86. NbS00051197g0005.1 **Mass:** 98617 **Score:** 64 **Matches:** 3(3) **Sequences:** 3(3) **emPAI:** 0.10

NbS00051197g0005.1 protein AED:0.14 eAED:0.15 QI:0|0.88|0.8|1|1|1|10|0|891; (*SWP) sp|Q9FFQ1|RH31_ARATH (e_value=0.0) DEAD-box ATP-dependent RNA helicase 31 OS=Arabidopsis thaliana GN=RH31 PE=2 SV=

Query	Observed	Mr(expt)	Mr(calc)	ppm	Miss	Score	Expect	Rank	Unique	Peptide
12214	589.3141	1176.6137	1176.6139	-0.10	0	44	0.0035	1	U	R.LVELANEFSR.T
14521	709.8967	1417.7788	1417.7777	0.79	0	30	0.056	1	U	K.STGLILVTSDVSAR.G
15588	772.4218	1542.8291	1542.8253	2.46	0	38	0.0092	1	U	R.ELATQAAAEANTLLK.Y

---

87. NbS00036674g0013.1 **Mass:** 196356 **Score:** 64 **Matches:** 2(2) **Sequences:** 2(2) **emPAI:** 0.03

NbS00036674g0013.1 protein AED:0.14 eAED:0.17 QI:0|0.5|0.4|1|0.5|0.4|5|0|1730; (*GB) gi|225433894|ref|XP_002266580.1| (e_value=0.0) PREDICTED: U5 small nuclear ribonucleoprotein 200 kDa helicase [V

Query	Observed	Mr(expt)	Mr(calc)	ppm	Miss	Score	Expect	Rank	Unique	Peptide
12317	594.3533	1186.6921	1186.6921	0.01	0	33	0.024	1	U	R.IVALSTSLANAK.D
12775	621.8094	1241.6043	1241.6041	0.22	0	55	0.00021	1	U	K.SGYFQVTDLGR.I

---

88. NbS00011860g0002.1 **Mass:** 87858 **Score:** 63 **Matches:** 2(2) **Sequences:** 2(2) **emPAI:** 0.08

NbS00011860g0002.1 protein AED:0.14 eAED:0.14 QI:0|0.83|0.76|0.84|1|1|13|0|788; (*GB) gi|255561268|ref|XP_002521645.1| (e_value=0.0) arsenite-resistance protein, putative [Ricinus communis];; (*SWI

Query	Observed	Mr(expt)	Mr(calc)	ppm	Miss	Score	Expect	Rank	Unique	Peptide
14304	697.8046	1393.5947	1393.5939	0.59	0	44	0.0011	1		R.FGYDYQPGYER.E
14328	698.8995	1395.7844	1395.7834	0.70	0	46	0.00096	1	U	R.VQIDVGQAQALVR.K

---

89. gi|119581148|gb|EAW60744.1| **Mass:** 57754 **Score:** 62 **Matches:** 4(4) **Sequences:** 3(3) **emPAI:** 0.25

gi|119581148|gb|EAW60744.1| keratin 9 (epidermolytic palmoplantar keratoderma) [Homo sapiens]

Query	Observed	Mr(expt)	Mr(calc)	ppm	Miss	Score	Expect	Rank	Unique	Peptide
9952	449.2109	896.4072	896.4062	1.11	0	33	0.027	1	U	R.MTLDDFR.I
10068	457.2086	912.4027	912.4011	1.78	0	(32)	0.02	1	U	R.MTLDDFR.I
11228	530.7858	1059.5571	1059.5560	1.01	0	41	0.0076	1	U	K.TLLDIDNTR.M
20614	837.3825	2509.1257	2509.1245	0.49	0	40	0.0033	1	U	K.EIETYHNLLEGGQEDFESSGAGK.I

---

90. NbS00007742g0006.1 **Mass:** 33872 **Score:** 61 **Matches:** 2(2) **Sequences:** 2(2) **emPAI:** 0.21

NbS00007742g0006.1 protein AED:0.40 eAED:0.42 QI:0|0.5|0.42|1|1|1|7|0|319; (*GB) gi|94466659|emb|CAJ44458.1| (e_value=2e-110) ALY protein [Nicotiana benthamiana];; (*SWP) sp|Q9JJW6|REFP2_MOUSE (e_v

Query	Observed	Mr(expt)	Mr(calc)	ppm	Miss	Score	Expect	Rank	Unique	Peptide
14401	469.5748	1405.7025	1405.7024	0.08	0	35	0.024	1		R.YNNVQLDGKPMK.I
17689	875.9547	1749.8949	1749.8897	2.93	0	52	0.00039	1	U	K.VYVSNLDVGVTNSDIR.V

Proteins matching the same set of peptides:

NbS00036740g0010.1 **Mass:** 28237 **Score:** 61 **Matches:** 2(2) **Sequences:** 2(2)

NbS00036740g0010.1 protein AED:0.03 eAED:0.03 QI:330|0.83|0.85|1|0.5|0.42|7|0|263; (*GB) gi|94466659|emb|CAJ44458.1| (e_value=3e-116) ALY protein [Nicotiana benthamiana];; (*SWP) sp|B5FXN8|THOC4_T

---

91. NbS00008510g0008.1 **Mass:** 222396 **Score:** 61 **Matches:** 3(3) **Sequences:** 3(3) **emPAI:** 0.04

NbS00008510g0008.1 protein AED:0.13 eAED:0.13 QI:0|0.89|0.86|0.98|0.93|0.94|50|0|2033; (*GB) gi|296085156|emb|CBI28651.3| (e_value=0.0) unnamed protein product [Vitis vinifera];; (*SWP) sp|Q92616|

Query	Observed	Mr(expt)	Mr(calc)	ppm	Miss	Score	Expect	Rank	Unique	Peptide
9720	435.7736	869.5327	869.5334	-0.85	0	35	0.0095	1	U	R.AIIEVLGR.E

10701	495.2929	988.5712	988.5706	0.64	0	49	0.00071	1	U	R.GAAFGLAGVVK.G
14523	709.8979	1417.7812	1417.7817	-0.33	0	31	0.046	1	U	R.SPIVGDVAYGTLVK.L

92. NbS00003662g0021.1 **Mass:** 29426 **Score:** 61 **Matches:** 1(1) **Sequences:** 1(1) **emPAI:** 0.11
NbS00003662g0021.1 protein AED:0.24 eAED:0.34 QI:0|0|0|1|0|0|3|0|257; (*GB) gi|78102516|ref|YP_358657.1| (e_value=6e-112) ATP synthase CF1 alpha subunit [Nicotiana sylvestris];; (*SWP) sp|Q3C1H4|A

Query	Observed	Mr(expt)	Mr(calc)	ppm	Miss	Score	Expect	Rank	Unique	Peptide
15107	744.9046	1487.7947	1487.7944	0.19	0	61	4.9e-005	1	U	K.ASSVAQVVTTLQER.G

93. NbS00001413g0059.1 **Score:** 60 **Matches:** 2(2) **Sequences:** 1(1) **emPAI:** 0.05
NbS00001413g0059.1 protein AED:0.42 eAED:0.46 QI:0|0|0|0.8|0.25|0.6|5|0|613; (*GB) gi|68164803|ref|YP_247599.1| (e_value=3e-131) photosystem I P700 chlorophyll a apoprotein A1 [Cucumis sativus];;

Query	Observed	Mr(expt)	Mr(calc)	ppm	Miss	Score	Expect	Rank	Unique	Peptide
10038	454.7276	907.4407	907.4399	0.86	0	58	0.00012	1	U	R.YNDLLDR.V 10037

Proteins matching the same set of peptides:
NbS00007581g0003.1 **Score:** 60 **Matches:** 2(2) **Sequences:** 1(1)
NbS00015599g0013.1 **Mass:** 37788 **Score:** 60 **Matches:** 2(2) **Sequences:** 1(1)
NbS00015599g0013.1 protein AED:0.22 eAED:0.25 QI:0|0|0|1|0|0|4|0|335; (*GB) gi|157325530|ref|YP_001468308.1| (e_value=9e-174) photosystem I P700 chlorophyll a apoprotein A1 [Ipomoea purpurea];; (*S
NbS00020555g0002.1 **Score:** 60 **Matches:** 2(2) **Sequences:** 1(1)

94. gi|297692195|ref|XP_002823453.1| **Mass:** 62271 **Score:** 59 **Matches:** 2(2) **Sequences:** 2(2) **emPAI:** 0.11
gi|297692195|ref|XP_002823453.1| PREDICTED: LOW QUALITY PROTEIN: ATP synthase subunit beta, mitochondrial-like [Pongo abelii]

Query	Observed	Mr(expt)	Mr(calc)	ppm	Miss	Score	Expect	Rank	Unique	Peptide
10602	488.2848	974.5550	974.5549	0.04	0	46	0.002	1		K.IGLFGGAGVGK.T
14672	718.3826	1434.7506	1434.7467	2.71	0	34	0.026	1	U	R.FTQAGSEVSALLGR.I

95. NbS00000548g0008.1 **Mass:** 40225 **Score:** 58 **Matches:** 3(2) **Sequences:** 3(2) **emPAI:** 0.27
NbS00000548g0008.1 protein AED:0.29 eAED:0.30 QI:0|0.8|0.5|1|1|0.66|6|0|363; (*GB) gi|255573386|ref|XP_002527619.1| (e_value=2e-130) Ras-GTPase-activating protein-binding protein, putative [Ricinus

Query	Observed	Mr(expt)	Mr(calc)	ppm	Miss	Score	Expect	Rank	Unique	Peptide
12649	615.3195	1228.6245	1228.6241	0.34	0	39	0.0084	1	U	K.GYFVLNDVFR.Y
14713	721.8698	1441.7250	1441.7242	0.53	0	31	0.057	1	U	K.FTQTFFLAPQDK.G
15078	743.3399	1484.6653	1484.6639	0.90	0	30	0.035	1	U	K.NINDMICSLDYK.N

Proteins matching the same set of peptides:
NbS00030703g0003.1 **Mass:** 53003 **Score:** 58 **Matches:** 3(2) **Sequences:** 3(2)
NbS00030703g0003.1 protein AED:0.29 eAED:0.29 QI:0|0.71|0.5|1|0.71|0.75|8|366|482; (*GB) gi|255573386|ref|XP_002527619.1| (e_value=9e-142) Ras-GTPase-activating protein-binding protein, putative [I

96. NbS00015187g0009.1 **Mass:** 56801 **Score:** 58 **Matches:** 2(2) **Sequences:** 2(2) **emPAI:** 0.12
NbS00015187g0009.1 protein AED:0.20 eAED:0.20 QI:141|0.94|0.94|1|0.72|0.84|19|64|510; (*GB) gi|356504541|ref|XP_003521054.1| (e_value=0.0) PREDICTED: actin-related protein 4-like [Glycine max];; (

Query	Observed	Mr(expt)	Mr(calc)	ppm	Miss	Score	Expect	Rank	Unique	Peptide
12497	605.8333	1209.6520	1209.6506	1.11	0	50	0.0006	1	U	R.LYVGSQALGFR.R
20669	853.4442	2557.3106	2557.3024	3.20	0	30	0.038	1	U	R.ATSLVVDSGGGSTTVAPVHDGYVLQK.A

97. NbS00038786g0002.1 **Mass:** 114358 **Score:** 57 **Matches:** 4(3) **Sequences:** 4(3) **emPAI:** 0.12
NbS00038786g0002.1 protein AED:0.22 eAED:0.22 QI:290|1|1|1|0.89|0.85|20|385|1023; (*GB) gi|359474892|ref|XP_002276432.2| (e_value=0.0) PREDICTED: pentatricopeptide repeat-containing protein At4g34

Query	Observed	Mr(expt)	Mr(calc)	ppm	Miss	Score	Expect	Rank	Unique	Peptide
11291	534.2763	1066.5381	1066.5369	1.13	0	30	0.062	1	U	K.ALEIFSEMK.R
11380	540.2833	1078.5520	1078.5546	-2.44	0	38	0.012	1	U	K.ALELYEDVK.G
16769	812.3931	1622.7717	1622.7722	-0.34	0	36	0.015	1	U	K.ACTLGEPVLSYNSGR.L
19715	1093.0137	2184.0128	2184.0124	0.20	0	36	0.011	1	U	K.LESPFSWEQTDLNFSQTR.L

98. NbS00020253g0009.1 **Mass:** 28540 **Score:** 57 **Matches:** 2(2) **Sequences:** 2(2) **emPAI:** 0.25
NbS00020253g0009.1 protein AED:0.18 eAED:0.18 QI:226|1|1|1|1|1|3|156|265; (*GB) gi|115794|sp|P27489.1|CB23_SOLLC (e_value=2e-176) RecName: Full=Chlorophyll a-b binding protein 13, chloroplastic; A

Query	Observed	Mr(expt)	Mr(calc)	ppm	Miss	Score	Expect	Rank	Unique	Peptide
18406	944.4901	1886.9655	1886.9634	1.13	0	37	0.012	1	U	R.WAMLGALGCITPEVLEK.W
21470	1250.6167	3748.8283	3748.8261	0.58	0	41	0.0017	1	U	R.INGLPGVGEGNNLYPGGQYFDPLGLADDPTTFAELK.V

---

99. >gi|74181742|dbj|BAE32582.1| **Mass:** 55528 **Score:** 57 **Matches:** 2(2) **Sequences:** 2(2) **emPAI:** 0.12
>gi|74181742|dbj|BAE32582.1| unnamed protein product [Mus musculus]

Query	Observed	Mr(expt)	Mr(calc)	ppm	Miss	Score	Expect	Rank	Unique	Peptide
10583	486.7774	971.5403	971.5400	0.32	0	44	0.0034	1		R.VVSVGDGIAR.V
20165	770.0579	2307.1519	2307.1495	1.05	0	34	0.021	1	U	R.EVAAFAQFGSDLDAATQALLNR.G

---

100. NbS00003163g0002.1 **Mass:** 22262 **Score:** 56 **Matches:** 1(1) **Sequences:** 1(1) **emPAI:** 0.15
NbS00003163g0002.1 protein ; (*GB) gi|146188483|emb|CAK12837.1| (e_value=1e-52) ribulose 1,5 biphosphate carboxylase/oxygenase [Liparia genistoides];; (*SWP) sp|P48709|RBL_NICDE (e_value=2e-52) Rib

Query	Observed	Mr(expt)	Mr(calc)	ppm	Miss	Score	Expect	Rank	Unique	Peptide
9885	445.2441	888.4737	888.4739	-0.17	0	56	0.0003	1	U	R.VALEACVK.A

---

101. NbS00003291g0003.1 **Mass:** 25216 **Score:** 55 **Matches:** 3(2) **Sequences:** 3(2) **emPAI:** 0.45
NbS00003291g0003.1 protein AED:0.07 eAED:0.07 QI:121|0.83|0.85|1|1|1|7|382|226; (*TAIR) AT3G07030.1 (e_value=4e-47) | Symbols: | Alba DNA/RNA-binding protein | chr3:2223001-2225254 RVERSE LENGTH=4(

Query	Observed	Mr(expt)	Mr(calc)	ppm	Miss	Score	Expect	Rank	Unique	Peptide
10446	479.3018	956.5891	956.5906	-1.59	0	40	0.0038	1		K.TVAIAEIIK.R
11649	557.3531	1112.6917	1112.6917	0.03	1	32	0.015	1	U	K.TVAIAEIIKR.R
15167	748.8846	1495.7546	1495.7532	0.97	0	31	0.05	1	U	R.NYQPQLQQQPPR.Q

Proteins matching the same set of peptides:
NbS00045053g0006.1 **Mass:** 26901 **Score:** 55 **Matches:** 3(2) **Sequences:** 3(2)
NbS00045053g0006.1 protein AED:0.04 eAED:0.04 QI:121|1|0.87|1|0.71|0.87|8|378|242; (*SWP) sp|Q8N5L8|RP25L_HUMAN (e_value=3e-17) Ribonuclease P protein subunit p25-like protein OS=Homo sapiens GN=RI

---

102. NbS00025223g0014.1 **Mass:** 15188 **Score:** 54 **Matches:** 3(3) **Sequences:** 3(3) **emPAI:** 0.83
NbS00025223g0014.1 protein AED:0.37 eAED:0.37 QI:0|1|0.33|1|1|0.66|3|0|136; (*GB) gi|51490663|emb|CAG26902.1| (e_value=3e-63) ALY protein [Nicotiana benthamiana];; (*SWP) sp|Q86V81|THOC4_HUMAN (e_v

Query	Observed	Mr(expt)	Mr(calc)	ppm	Miss	Score	Expect	Rank	Unique	Peptide
14401	469.5748	1405.7025	1405.7024	0.08	0	35	0.024	1		K.YNNVQLDGKPMK.I
17175	837.9102	1673.8059	1673.7995	3.79	0	33	0.028	1	U	R.VENITAEDLDADLEK.Y
17549	866.9915	1731.9685	1731.9672	0.76	0	36	0.009	1	U	K.IEFAGPNIGAPALPPLR.N

---

103. NbS00036843g0004.1 **Mass:** 42375 **Score:** 54 **Matches:** 2(2) **Sequences:** 2(2) **emPAI:** 0.16
NbS00036843g0004.1 protein AED:0.04 eAED:0.04 QI:8|0.5|0.66|1|0.5|0.33|3|0|387; (*SWP) sp|P25083|ADT1_SOLTU (e_value=0.0) ADP,ATP carrier protein, mitochondrial OS=Solanum tuberosum GN=ANT PE=2 SV=

Query	Observed	Mr(expt)	Mr(calc)	ppm	Miss	Score	Expect	Rank	Unique	Peptide
14028	680.8639	1359.7132	1359.7068	4.75	0	41	0.0065	1		K.LLIQNQDEMIK.A
18527	956.9980	1911.9814	1911.9765	2.60	0	38	0.0085	2	U	K.GVSGFAIDFLMGGVSAAVSK.T

---

104. NbS00013798g0018.1 **Mass:** 7199 **Score:** 53 **Matches:** 2(2) **Sequences:** 1(1) **emPAI:** 0.49
NbS00013798g0018.1 protein AED:0.47 eAED:0.47 QI:0|0|0|1|0|0.5|2|0|68; (*GB) gi|55977763|sp|P00823.2|ATPA_TOBAC (e_value=7e-32) RecName: Full=ATP synthase subunit alpha, chloroplastic; AltName: Ful

Query	Observed	Mr(expt)	Mr(calc)	ppm	Miss	Score	Expect	Rank	Unique	Peptide
14502	708.8962	1415.7778	1415.7772	0.40	0	48	0.00084	1	U	R.IAQIPVSEAYLGR.V 14504

Proteins matching the same set of peptides:
NbS00014241g0002.1 **Mass:** 21890 **Score:** 53 **Matches:** 2(2) **Sequences:** 1(1)
NbS00014241g0002.1 protein AED:0.09 eAED:0.09 QI:0|0|0|1|0|0|2|0|201; (*SWP) sp|P00823|ATPA_TOBAC (e_value=5e-67) ATP synthase subunit alpha, chloroplastic OS=Nicotiana tabacum GN=atpA PE=2 SV=2;;

---

105. NbS00019153g0015.1 **Mass:** 157163 **Score:** 53 **Matches:** 2(1) **Sequences:** 2(1) **emPAI:** 0.04
NbS00019153g0015.1 protein AED:0.14 eAED:0.14 QI:159|0.84|0.88|1|0.96|0.92|27|471|1387; (*GB) gi|125535486|gb|EAY81974.1| (e_value=0.0) hypothetical protein OsI_37152 [Oryza sativa Indica Group];;

Query	Observed	Mr(expt)	Mr(calc)	ppm	Miss	Score	Expect	Rank	Unique	Peptide
8356	636.3300	1270.6454	1270.6445	0.76	0	30	0.23	1	U	K.VDSELIYAYAK.I
14551	711.8438	1421.6729	1421.6722	0.56	0	48	0.00098	1	U	K.GNMQLFSVDQQR.S

Proteins matching the same set of peptides:
NbS00032250g0020.1 **Mass:** 128864 **Score:** 53 **Matches:** 2(1) **Sequences:** 2(1)
NbS00032250g0020.1 protein AED:0.17 eAED:0.17 QI:0|0.88|0.85|1|0.84|0.74|27|1333|1143; (*GB) gi|225434540|ref|XP_002276855.1| (e_value=0.0) PREDICTED: clathrin heavy chain 2 [Vitis vinifera];; (*SW
NbS00036207g0006.1 **Mass:** 171808 **Score:** 53 **Matches:** 2(1) **Sequences:** 2(1)
NbS00036207g0006.1 protein AED:0.15 eAED:0.16 QI:380|0.72|0.76|0.96|0.88|0.96|26|437|1514; (*GB) gi|125535486|gb|EAY81974.1| (e_value=0.0) hypothetical protein OsI_37152 [Oryza sativa Indica Group]
NbS00045842g0003.1 **Mass:** 172178 **Score:** 53 **Matches:** 2(1) **Sequences:** 2(1)
NbS00045842g0003.1 protein AED:0.13 eAED:0.13 QI:341|0.84|0.84|1|0.96|0.96|26|489|1512; (*GB) gi|125535486|gb|EAY81974.1| (e_value=0.0) hypothetical protein OsI_37152 [Oryza sativa Indica Group];;
NbS00048496g0006.1 **Mass:** 165591 **Score:** 53 **Matches:** 2(1) **Sequences:** 2(1)
NbS00048496g0006.1 protein AED:0.11 eAED:0.12 QI:303|0.88|0.85|1|0.76|0.77|27|0|1461; (*GB) gi|125535486|gb|EAY81974.1| (e_value=0.0) hypothetical protein OsI_37152 [Oryza sativa Indica Group];; (

---

106. NbS00044222g0007.1 **Mass:** 69788 **Score:** 53 **Matches:** 2(2) **Sequences:** 2(2) **emPAI:** 0.10
NbS00044222g0007.1 protein AED:0.20 eAED:0.20 QI:1972|0.73|0.68|0.87|0.73|0.62|16|0|619; (*GB) gi|225463033|ref|XP_002267199.1| (e_value=0.0) PREDICTED: 116 kDa U5 small nuclear ribonucleoprotein c

Query	Observed	Mr(expt)	Mr(calc)	ppm	Miss	Score	Expect	Rank	Unique	Peptide
10169	463.2780	924.5414	924.5392	2.30	0	30	0.03	1	U	K.IALEPLNR.G
14444	705.8574	1409.7003	1409.6973	2.13	0	49	0.00077	1	U	R.VYSGEIMAGQTVR.V

---

107. NbS00002935g0006.1 **Mass:** 54788 **Score:** 53 **Matches:** 2(2) **Sequences:** 2(2) **emPAI:** 0.12
NbS00002935g0006.1 protein AED:0.24 eAED:0.28 QI:9|0.53|0.35|1|0.84|0.71|14|0|493; (*GB) gi|6715512|gb|AAF26445.1| (e_value=0.0) vacuolar H+-ATPase B subunit [Nicotiana tabacum];; (*SWP) sp|Q9SZN1

Query	Observed	Mr(expt)	Mr(calc)	ppm	Miss	Score	Expect	Rank	Unique	Peptide
11454	544.7922	1087.5699	1087.5696	0.31	0	38	0.013	1	U	K.TPVSLDMLGR.I
17450	858.4694	1714.9243	1714.9254	-0.63	0	36	0.013	1	U	R.VTLFLNLANDPTIER.I

Proteins matching the same set of peptides:
NbS00006432g0020.1 **Mass:** 58076 **Score:** 53 **Matches:** 2(2) **Sequences:** 2(2)
NbS00006432g0020.1 protein AED:0.14 eAED:0.14 QI:0|0.85|0.86|1|0.78|0.66|15|354|521; (*GB) gi|357507939|ref|XP_003624258.1| (e_value=0.0) V-type ATP synthase beta chain [Medicago truncatula];; (*SW
NbS00007553g0003.1 **Mass:** 28303 **Score:** 53 **Matches:** 2(2) **Sequences:** 2(2)
NbS00007553g0003.1 protein AED:0.28 eAED:0.28 QI:132|0.93|0.93|1|0.66|0.62|16|917|258; (*GB) gi|224105123|ref|XP_002313695.1| (e_value=1e-175) predicted protein [Populus trichocarpa];; (*SWP) sp|Q
NbS00010495g0008.1 **Mass:** 62704 **Score:** 53 **Matches:** 2(2) **Sequences:** 2(2)
NbS00010495g0008.1 protein AED:0.05 eAED:0.05 QI:0|0.86|0.93|0.93|0.73|0.81|16|352|560; (*GB) gi|302141720|emb|CBI18923.3| (e_value=0.0) unnamed protein product [Vitis vinifera];; (*SWP) sp|Q9SZN1

---

108. NbS00006964g0008.1 **Mass:** 79801 **Score:** 52 **Matches:** 2(2) **Sequences:** 2(2) **emPAI:** 0.08
NbS00006964g0008.1 protein AED:0.23 eAED:0.23 QI:394|0.87|1|1|0.93|0.88|17|339|721; (*GB) gi|255552828|ref|XP_002517457.1| (e_value=0.0) H(\+)-transporting atpase plant/fungi plasma membrane type,

Query	Observed	Mr(expt)	Mr(calc)	ppm	Miss	Score	Expect	Rank	Unique	Peptide
7420	503.7900	1005.5654	1005.5607	4.68	0	38	0.011	1	U	R.VFGVSTLQR.T
11786	565.3041	1128.5936	1128.5927	0.76	0	45	0.0024	1	U	R.AWDLVLEQR.I

Proteins matching the same set of peptides:
NbS00022217g0004.1 **Mass:** 78168 **Score:** 52 **Matches:** 2(2) **Sequences:** 2(2)
NbS00022217g0004.1 protein AED:0.21 eAED:0.21 QI:283|0.93|1|1|0.93|0.88|17|503|702; (*GB) gi|350535937|ref|NP_001234477.1| (e_value=0.0) plasma membrane H+-ATPase [Solanum lycopersicum];; (*SWP) sp

---

109. NbS00004901g0005.1 **Mass:** 43708 **Score:** 52 **Matches:** 2(2) **Sequences:** 2(2) **emPAI:** 0.16
NbS00004901g0005.1 protein AED:0.05 eAED:0.05 QI:0|1|0.5|1|1|1|4|0|391; (*GB) gi|231503|sp|P30171.1|ACT11_SOLTU (e_value=0.0) RecName: Full=Actin-97;; (*SWP) sp|P30171|ACT11_SOLTU (e_value=0.0) Act

Query	Observed	Mr(expt)	Mr(calc)	ppm	Miss	Score	Expect	Rank	Unique	Peptide
17845	887.9539	1773.8932	1773.8897	1.95	0	45	0.002	1	U	K.NYELPDGQVITIGAER.F
18303	935.4821	1868.9497	1868.9407	4.80	0	30	0.051	1	U	K.LAYIALDYEQELETAK.T

Proteins matching the same set of peptides:
NbS00011031g0002.1 **Mass:** 41871 **Score:** 52 **Matches:** 2(2) **Sequences:** 2(2)
NbS00011031g0002.1 protein AED:0.14 eAED:0.14 QI:236|1|1|1|0.75|0.6|5|154|377; (*GB) gi|231503|sp|P30171.1|ACT11_SOLTU (e_value=0.0) RecName: Full=Actin-97;; (*SWP) sp|P30171|ACT11_SOLTU (e_value=(
NbC23512151g0001.1 **Mass:** 8878 **Score:** 52 **Matches:** 2(2) **Sequences:** 2(2)
NbC23512151g0001.1 protein AED:0.00 eAED:0.00 QI:0|-1|1|1|-1|0|1|0|78; (*GB) gi|380294407|gb|AFD50698.1| (e_value=2e-50) actin, partial [Plectranthus hilliardiae x Plectranthus saccatus];; (*SWP) s

---

110. NbS00003075g0011.1 **Mass:** 15245 **Score:** 51 **Matches:** 2(2) **Sequences:** 2(2) **emPAI:** 0.50

NbS00003075g0011.1 protein AED:0.28 eAED:0.28 QI:0|1|0.66|1|1|1|3|0|143; (*GB) gi|2499967|sp|Q41229.1|PSAEB_NICSY (e_value=1e-59) RecName: Full=Photosystem I reaction center subunit IV B, chloropla

Query	Observed	Mr(expt)	Mr(calc)	ppm	Miss	Score	Expect	Rank	Unique	Peptide
15339	757.3855	1512.7564	1512.7533	2.10	0	33	0.035	1	U	K.GVGSVVAVDQDPNTR.Y
19189	1010.9977	2019.9808	2019.9749	2.95	0	37	0.01	1	U	R.AAEEAAPPAATATAEGEAPPAK.A

---

111. NbS00024580g0005.1 **Mass:** 35587 **Score:** 51 **Matches:** 3(3) **Sequences:** 3(3) **emPAI:** 0.31

NbS00024580g0005.1 protein AED:0.21 eAED:0.21 QI:87|0.83|0.85|1|0.83|0.85|7|278|321; (*SWP) sp|Q641W4|RFC2_RAT (e_value=5e-157) Replication factor C subunit 2 OS=Rattus norvegicus GN=Rfc2 PE=2 SV=1

Query	Observed	Mr(expt)	Mr(calc)	ppm	Miss	Score	Expect	Rank	Unique	Peptide
11009	515.7794	1029.5443	1029.5455	-1.14	0	37	0.015	1		R.LSDQEILGR.L
18897	657.6769	1970.0090	1970.0069	1.08	1	33	0.026	1		K.EAVLELNASDDRGIDVVR.N
19308	1030.0426	2058.0707	2058.0674	1.60	0	32	0.034	1	U	K.ALYDLGYSPTDIITTLFR.I

---

112. NbS00018023g0003.1 **Mass:** 48157 **Score:** 51 **Matches:** 3(3) **Sequences:** 3(3) **emPAI:** 0.22

NbS00018023g0003.1 protein AED:0.25 eAED:0.25 QI:0|1|0.75|1|1|1|4|510|435; (*GB) gi|297845476|ref|XP_002890619.1| (e_value=2e-95) hypothetical protein ARALYDRAFT_472693 [Arabidopsis lyrata subsp. l

Query	Observed	Mr(expt)	Mr(calc)	ppm	Miss	Score	Expect	Rank	Unique	Peptide
12071	582.3026	1162.5907	1162.5903	0.28	0	35	0.032	1	U	R.IVQMEESLSK.G
12924	629.8488	1257.6830	1257.6816	1.08	0	33	0.038	1	U	K.SAVLAGIEELEK.L
16159	797.8988	1593.7831	1593.7854	-1.50	0	37	0.012	1	U	K.SLQQSDLTATMLMR.T

---

113. NbS00009996g0026.1 **Mass:** 31165 **Score:** 50 **Matches:** 3(3) **Sequences:** 3(3) **emPAI:** 0.35

NbS00009996g0026.1 protein AED:0.33 eAED:0.33 QI:87|0.83|0.71|1|0.66|0.85|7|314|282; (*GB) gi|224074887|ref|XP_002304476.1| (e_value=7e-172) predicted protein [Populus trichocarpa];; (*SWP) sp|Q05I

Query	Observed	Mr(expt)	Mr(calc)	ppm	Miss	Score	Expect	Rank	Unique	Peptide
11009	515.7794	1029.5443	1029.5455	-1.14	0	37	0.015	1		R.LSDQEILGR.L
13850	672.8427	1343.6709	1343.6681	2.06	0	38	0.012	1	U	K.VSDIVGNEDAVAR.L
18897	657.6769	1970.0090	1970.0069	1.08	1	33	0.026	1		K.EAVLELNASDDRGIDVVR.N

---

114. NbS00010860g0014.1 **Mass:** 9991 **Score:** 50 **Matches:** 2(2) **Sequences:** 2(2) **emPAI:** 0.82

NbS00010860g0014.1 protein AED:0.05 eAED:0.05 QI:88|1|1|1|1|1|5|242|88; (*GB) gi|225468340|ref|XP_002272246.1| (e_value=5e-54) PREDICTED: probable small nuclear ribonucleoprotein F [Vitis vinifera]

Query	Observed	Mr(expt)	Mr(calc)	ppm	Miss	Score	Expect	Rank	Unique	Peptide
11158	526.2721	1050.5296	1050.5280	1.53	0	37	0.016	1	U	R.CNNVLYLR.G
15983	787.8275	1573.6405	1573.6380	1.58	1	36	0.0032	1	U	R.GVPEDEELDDADRD.-

Proteins matching the same set of peptides:

NbS00029443g0003.1 **Mass:** 9991 **Score:** 50 **Matches:** 2(2) **Sequences:** 2(2)

NbS00029443g0003.1 protein AED:0.25 eAED:0.25 QI:64|1|1|1|1|1|5|228|88; (*GB) gi|225468340|ref|XP_002272246.1| (e_value=5e-54) PREDICTED: probable small nuclear ribonucleoprotein F [Vitis vinifera]

---

115. NbS00001587g0026.1 **Mass:** 107641 **Score:** 50 **Matches:** 1(1) **Sequences:** 1(1) **emPAI:** 0.03

NbS00001587g0026.1 protein AED:0.19 eAED:0.20 QI:234|0.91|0.83|1|0.82|0.83|24|284|993; (*SWP) sp|P35601|RFC1_MOUSE (e_value=5e-125) Replication factor C subunit 1 OS=Mus musculus GN=Rfc1 PE=1 SV=2;

Query	Observed	Mr(expt)	Mr(calc)	ppm	Miss	Score	Expect	Rank	Unique	Peptide
14826	729.8816	1457.7486	1457.7474	0.85	0	50	0.0007	1	U	K.ELVSNESLGANIGR.S

Proteins matching the same set of peptides:

NbS00012440g0009.1 **Mass:** 110612 **Score:** 50 **Matches:** 1(1) **Sequences:** 1(1)

NbS00012440g0009.1 protein AED:0.21 eAED:0.21 QI:69|0.95|0.85|1|0.95|0.95|21|282|1019; (*GB) gi|296083902|emb|CBI24290.3| (e_value=0.0) unnamed protein product [Vitis vinifera];; (*SWP) sp|P35601|F

---

116. NbS00013764g0007.1 **Mass:** 53134 **Score:** 50 **Matches:** 2(2) **Sequences:** 2(2) **emPAI:** 0.13

NbS00013764g0007.1 protein AED:0.11 eAED:0.11 QI:116|0.85|0.87|1|0.85|0.75|8|606|455; (*GB) gi|1705613|sp|P49319.2|CATA1_TOBAC (e_value=0.0) RecName: Full=Catalase isozyme 1; AltName: Full=Salicyl:

Query	Observed	Mr(expt)	Mr(calc)	ppm	Miss	Score	Expect	Rank	Unique	Peptide
11421	542.7758	1083.5371	1083.5349	2.02	0	39	0.0085	1	U	R.IFAYADTQR.H
11854	568.8430	1135.6715	1135.6714	0.11	0	34	0.01	1		R.APGVQTPVIVR.F

---

117. NbS00003763g0016.1 **Mass:** 99171 **Score:** 48 **Matches:** 3(3) **Sequences:** 3(3) **emPAI:** 0.10

NbS00003763g0016.1 protein AED:0.10 eAED:0.12 QI:0|0.92|0.8|0.93|1|1|15|0|884; (*GB) gi|225441896|ref|XP_002284404.1| (e_value=0.0) PREDICTED: uncharacterized protein At2g41620 [Vitis vinifera];;

Query	Observed	Mr(expt)	Mr(calc)	ppm	Miss	Score	Expect	Rank	Unique	Peptide
11337	536.8218	1071.6290	1071.6288	0.20	0	32	0.036	1	U	K.QLLTELLSR.D
11755	563.3181	1124.6217	1124.6190	2.41	0	30	0.042	1	U	R.DGGIDILLGPR.G
17499	575.6262	1723.8567	1723.8530	2.15	1	36	0.014	1	U	R.LRDYGVLDFDAVDAR.R

Proteins matching the same set of peptides:
NbS00021908g0010.1 **Mass:** 102988 **Score:** 48 **Matches:** 3(3) **Sequences:** 3(3)
NbS00021908g0010.1 protein AED:0.11 eAED:0.11 QI:0|0.71|0.73|1|0.85|0.8|15|307|915; (*SWP) sp|O22224|Y2162_ARATH (e_value=0.0) Uncharacterized protein At2g41620 OS=Arabidopsis thaliana GN=At2g4162(

118. NbS00005390g0012.1 **Mass:** 12600 **Score:** 48 **Matches:** 1(1) **Sequences:** 1(1) **emPAI:** 0.27
NbS00005390g0012.1 protein AED:0.37 eAED:0.37 QI:218|1|1|1|1|1|4|363|114; (*GB) gi|296086730|emb|CBI32365.3| (e_value=4e-61) unnamed protein product [Vitis vinifera];; (*SWP) sp|P62315|SMD1_MOUSE

Query	Observed	Mr(expt)	Mr(calc)	ppm	Miss	Score	Expect	Rank	Unique	Peptide
12939	630.3463	1258.6780	1258.6768	0.88	0	48	0.0012	1	U	K.LNNETVSIELK.N

Proteins matching the same set of peptides:
NbS00006569g0006.1 **Mass:** 12600 **Score:** 48 **Matches:** 1(1) **Sequences:** 1(1)
NbS00006569g0006.1 protein AED:0.30 eAED:0.30 QI:0|0.66|0.5|0.75|1|1|4|226|114; (*GB) gi|255559488|ref|XP_002520764.1| (e_value=5e-61) small nuclear ribonucleoprotein sm d1, putative [Ricinus commu
NbS00054309g0007.1 **Mass:** 12600 **Score:** 48 **Matches:** 1(1) **Sequences:** 1(1)
NbS00054309g0007.1 protein AED:0.40 eAED:0.40 QI:0|0.66|0.5|0.75|1|1|4|443|114; (*GB) gi|255559488|ref|XP_002520764.1| (e_value=5e-61) small nuclear ribonucleoprotein sm d1, putative [Ricinus commu

119. NbS00015339g0013.1 **Mass:** 102746 **Score:** 48 **Matches:** 2(1) **Sequences:** 2(1) **emPAI:** 0.06
NbS00015339g0013.1 protein AED:0.20 eAED:0.20 QI:0|0.95|0.80|1|1|0.95|21|0|931; (*GB) gi|584795|sp|Q08436.1|PMA3_NICPL (e_value=0.0) RecName: Full=Plasma membrane ATPase 3; AltName: Full=Proton pu

Query	Observed	Mr(expt)	Mr(calc)	ppm	Miss	Score	Expect	Rank	Unique	Peptide
11786	565.3041	1128.5936	1128.5927	0.76	0	45	0.0024	1	U	K.AWDLVIEQR.I
13599	659.3470	1316.6795	1316.6758	2.81	0	30	0.074	1	U	R.GVDADTVVLMAAR.A

120. NbS00003616g0011.1 **Mass:** 44827 **Score:** 47 **Matches:** 1(1) **Sequences:** 1(1) **emPAI:** 0.07
NbS00003616g0011.1 protein AED:0.18 eAED:0.19 QI:0|0.5|0.33|1|1|1|3|0|390; (*SWP) sp|Q9LVA0|BAG7_ARATH (e_value=1e-62) BAG family molecular chaperone regulator 7 OS=Arabidopsis thaliana GN=BAG7 PE=

Query	Observed	Mr(expt)	Mr(calc)	ppm	Miss	Score	Expect	Rank	Unique	Peptide
14145	687.8649	1373.7153	1373.7151	0.18	0	47	0.0014	1	U	R.VGLGTELQSLSDR.V

Proteins matching the same set of peptides:
NbS00037609g0006.1 **Mass:** 45007 **Score:** 47 **Matches:** 1(1) **Sequences:** 1(1)
NbS00037609g0006.1 protein AED:0.10 eAED:0.10 QI:0|0.5|0.33|1|1|1|3|0|390; (*GB) gi|255539763|ref|XP_002510946.1| (e_value=5e-109) conserved hypothetical protein [Ricinus communis];; (*SWP) sp|Q9LV

121. NbS00004261g0112.1 **Mass:** 48827 **Score:** 47 **Matches:** 2(1) **Sequences:** 2(1) **emPAI:** 0.14
NbS00004261g0112.1 protein AED:0.31 eAED:0.34 QI:6|0.92|0.92|1|0.76|0.71|14|341|438

Query	Observed	Mr(expt)	Mr(calc)	ppm	Miss	Score	Expect	Rank	Unique	Peptide
10258	468.7378	935.4610	935.4613	-0.34	0	31	0.07	1	U	K.FSWGVANR.G
20752	869.4541	2605.3405	2605.3428	-0.89	0	40	0.0038	1	U	K.VVSEVPWFGIEQEYTLLQQNVK.W

Proteins matching the same set of peptides:
NbS00017344g0018.1 **Mass:** 47768 **Score:** 47 **Matches:** 2(1) **Sequences:** 2(1)
NbS00017344g0018.1 protein AED:0.28 eAED:0.28 QI:6|1|0.84|1|1|1|13|0|432; (*GB) gi|40457328|gb|AAR86719.1| (e_value=0.0) glutamine synthetase GS58 [Nicotiana attenuata];; (*SWP) sp|O22506|GLNA2_DAU

122. NbS00001859g0006.1 **Mass:** 58802 **Score:** 47 **Matches:** 2(2) **Sequences:** 2(2) **emPAI:** 0.11
NbS00001859g0006.1 protein AED:0.10 eAED:0.10 QI:0|0.42|0.5|1|0.85|0.75|8|0|558; (*GB) gi|313585890|gb|ADR71054.1| (e_value=0.0) phosphoglycerate kinase [Nicotiana benthamiana];; (*SWP) sp|Q42961|I

Query	Observed	Mr(expt)	Mr(calc)	ppm	Miss	Score	Expect	Rank	Unique	Peptide
11548	550.8306	1099.6466	1099.6488	-2.06	0	31	0.039	1	U	K.IGVIESLLEK.C
11750	563.2759	1124.5372	1124.5350	1.99	0	42	0.0041	1	U	K.TFNDALDTTK.T

Proteins matching the same set of peptides:
NbS00006821g0002.1 **Mass:** 53479 **Score:** 47 **Matches:** 2(2) **Sequences:** 2(2)
NbS00006821g0002.1 protein AED:0.20 eAED:0.20 QI:0|0.5|0.57|1|1|1|7|278|508; (*SWP) sp|Q42961|PGKH_TOBAC (e_value=0.0) Phosphoglycerate kinase, chloroplastic OS=Nicotiana tabacum PE=2 SV=1;; (*TAII

123. NbS00028776g0007.1 **Mass:** 54087 **Score:** 47 **Matches:** 2(2) **Sequences:** 2(2) **emPAI:** 0.13
NbS00028776g0007.1 protein AED:0.22 eAED:0.37 QI:0|0.6|0.16|1|0.8|0.66|6|0|494; (*GB) gi|262192739|gb|ACY30439.1| (e_value=4e-163) hypothetical protein [Nicotiana tabacum];; (*TAIR) AT4G37130.1 (e_

Query	Observed	Mr(expt)	Mr(calc)	ppm	Miss	Score	Expect	Rank	Unique	Peptide
15142	747.3696	1492.7246	1492.7191	3.64	0	31	0.053	1	U	R.SIQELGGVSTSMER.Q
18237	929.9276	1857.8407	1857.8381	1.40	0	33	0.02	1	U	R.LYDSSVANDGFELDASR.S

124. NbS00013589g0010.1 **Mass:** 44368 **Score:** 46 **Matches:** 1(1) **Sequences:** 1(1) **emPAI:** 0.07
NbS00013589g0010.1 protein AED:0.15 eAED:0.18 QI:0|0.33|0|0.75|0.66|0.5|4|0|390; (*SWP) sp|Q9LMR3|TYRA2_ARATH (e_value=4e-132) Arogenate dehydrogenase 2, chloroplastic OS=Arabidopsis thaliana GN=TY

Query	Observed	Mr(expt)	Mr(calc)	ppm	Miss	Score	Expect	Rank	Unique	Peptide
12705	617.8554	1233.6963	1233.6969	-0.51	0	46	0.0012	1	U	R.NTLFVDVLSVK.E

Proteins matching the same set of peptides:
NbS00030505g0002.1 **Mass:** 43114 **Score:** 46 **Matches:** 1(1) **Sequences:** 1(1)
NbS00030505g0002.1 protein AED:0.11 eAED:0.11 QI:0|-1|0|1|-1|1|1|0|376; (*GB) gi|325516252|gb|ADZ24704.1| (e_value=0.0) arogenate dehydrogenase 1 [Solanum pennellii];; (*SWP) sp|Q9LMR3|TYRA2_ARATH
NbS00054171g0001.1 **Mass:** 45565 **Score:** 46 **Matches:** 1(1) **Sequences:** 1(1)
NbS00054171g0001.1 protein AED:0.13 eAED:0.13 QI:0|-1|0|1|-1|1|1|0|394; (*GB) gi|325516254|gb|ADZ24705.1| (e_value=4e-179) arogenate dehydrogenase 2 [Solanum pennellii];; (*SWP) sp|Q9LMR3|TYRA2_ARA

125. NbS00019903g0005.1 **Mass:** 162134 **Score:** 46 **Matches:** 2(2) **Sequences:** 2(2) **emPAI:** 0.04
NbS00019903g0005.1 protein AED:0.20 eAED:0.20 QI:0|1|1|1|1|1|20|174|1434; (*GB) gi|75326590|sp|Q76CU2.1|PDR1_TOBAC (e_value=0.0) RecName: Full=Pleiotropic drug resistance protein 1; AltName: Full=N

Query	Observed	Mr(expt)	Mr(calc)	ppm	Miss	Score	Expect	Rank	Unique	Peptide
12822	623.3335	1244.6524	1244.6513	0.89	0	35	0.026	1	U	R.GGQEIYVGPLGR.H
18364	939.9758	1877.9371	1877.9371	0.03	0	39	0.007	1	U	K.AAATEGQEANVVTDYVLK.I

Proteins matching the same set of peptides:
NbS00038421g0004.1 **Mass:** 170425 **Score:** 46 **Matches:** 2(2) **Sequences:** 2(2)
NbS00038421g0004.1 protein AED:0.16 eAED:0.16 QI:204|0.9|0.90|1|1|1|21|174|1502; (*GB) gi|75326590|sp|Q76CU2.1|PDR1_TOBAC (e_value=0.0) RecName: Full=Pleiotropic drug resistance protein 1; AltName:

126. NbS00056603g0002.1 **Mass:** 41513 **Score:** 46 **Matches:** 1(1) **Sequences:** 1(1) **emPAI:** 0.08
NbS00056603g0002.1 protein AED:0.27 eAED:0.27 QI:0|0.33|0.5|1|1|1|4|0|363; (*GB) gi|340501186|gb|EGR27996.1| (e_value=0.0) hypothetical protein IMG5_185210 [Ichthyophthirius multifiliis];; (*SWP) s

Query	Observed	Mr(expt)	Mr(calc)	ppm	Miss	Score	Expect	Rank	Unique	Peptide
8510	663.8300	1325.6454	1325.6364	6.83	0	46	0.0018	1	U	R.INVYFNEASGGR.Y

127. NbS00004937g0014.1 **Mass:** 29484 **Score:** 45 **Matches:** 2(2) **Sequences:** 2(2) **emPAI:** 0.24
NbS00004937g0014.1 protein AED:0.10 eAED:0.10 QI:88|1|1|1|0.57|1|8|322|272; (*GB) gi|356566431|ref|XP_003551435.1| (e_value=3e-54) PREDICTED: uncharacterized protein LOC100804648 [Glycine max];; (*

Query	Observed	Mr(expt)	Mr(calc)	ppm	Miss	Score	Expect	Rank	Unique	Peptide
10446	479.3018	956.5891	956.5906	-1.59	0	40	0.0038	1		K.TVAIAEIIK.E
16999	828.4247	1654.8349	1654.8315	2.11	0	32	0.038	1	U	R.NYISYATALLQDQR.T

128. gi|3336842|emb|CAA76847.1| **Mass:** 71274 **Score:** 45 **Matches:** 1(1) **Sequences:** 1(1) **emPAI:** 0.05
gi|3336842|emb|CAA76847.1| bovine serum albumin [Bos taurus]

Query	Observed	Mr(expt)	Mr(calc)	ppm	Miss	Score	Expect	Rank	Unique	Peptide
12078	582.3193	1162.6241	1162.6234	0.65	0	45	0.0023	1	U	K.LVNELTEFAK.T

129. NbS00061449g0009.1 **Mass:** 50334 **Score:** 45 **Matches:** 1(1) **Sequences:** 1(1) **emPAI:** 0.07
NbS00061449g0009.1 protein AED:0.21 eAED:0.23 QI:76|1|0.9|1|0.77|0.9|10|0|443; (*SWP) sp|Q8RXH2|NUP85_ARATH (e_value=2e-164) Nuclear pore complex protein Nup85 OS=Arabidopsis thaliana GN=NUP85 PE=2

Query	Observed	Mr(expt)	Mr(calc)	ppm	Miss	Score	Expect	Rank	Unique	Peptide
14441	705.8317	1409.6488	1409.6423	4.63	0	45	0.0016	1	U	K.LSNSGYEEVDAAR.W

130. NbS00047628g0009.1 **Mass:** 52762 **Score:** 45 **Matches:** 2(2) **Sequences:** 2(2) **emPAI:** 0.13
NbS00047628g0009.1 protein AED:0.27 eAED:0.27 QI:381|1|1|1|1|1|10|557|477; (*GB) gi|217074984|gb|ACJ85852.1| (e_value=0.0) unknown [Medicago truncatula];; (*SWP) sp|Q8VXQ2|ALDH_CRAPL (e_value=0.0)

Query	Observed	Mr(expt)	Mr(calc)	ppm	Miss	Score	Expect	Rank	Unique	Peptide
12906	628.8464	1255.6783	1255.6772	0.89	0	38	0.01	1	U	K.SPAVVDSNINLK.V

13021	633.8984	1265.7822	1265.7819	0.22	0	33	0.005	1	U	K.ALLNGNILGLIR.A

131. NbS00001849g0017.1 **Mass:** 8189 **Score:** 45 **Matches:** 2(2) **Sequences:** 2(2) **emPAI:** 1.04
NbS00001849g0017.1 protein AED:0.76 eAED:1.00 QI:0|0|0|0.66|0.5|0.66|3|0|72; (*GB) gi|94466657|emb|CAJ44457.1| (e_value=3e-26) ALY protein [Nicotiana benthamiana];; (*ITAG) Solyc10g086400.1.1 (e_va

Query	Observed	Mr(expt)	Mr(calc)	ppm	Miss	Score	Expect	Rank	Unique	Peptide
14401	469.5748	1405.7025	1405.7024	0.08	0	35	0.024	1		K.YNNVQLDGKPMK.I
17549	866.9915	1731.9685	1731.9672	0.76	0	36	0.009	1	U	K.IEFAGPNIGAPALPPIR.N

Proteins matching the same set of peptides:
NbS00004226g0001.1 **Mass:** 16820 **Score:** 45 **Matches:** 2(2) **Sequences:** 2(2)
NbS00004226g0001.1 protein ; (*GB) gi|94466657|emb|CAJ44457.1| (e_value=2e-33) ALY protein [Nicotiana benthamiana];; (*TAIR) AT5G02530.2 (e_value=1e-14) | Symbols: | RNA-binding (RRM/RBD/RNP motifs

132. NbS00008453g0109.1 **Mass:** 25017 **Score:** 45 **Matches:** 1(1) **Sequences:** 1(1) **emPAI:** 0.13
NbS00008453g0109.1 protein AED:0.28 eAED:0.44 QI:0|0|0|0.66|0.5|0.33|3|0|219

Query	Observed	Mr(expt)	Mr(calc)	ppm	Miss	Score	Expect	Rank	Unique	Peptide
20046	1141.5461	2281.0777	2281.0903	-5.51	0	45	0.0014	1	U	R.DVFAGIDPDLDAQVEFGAFQK.I

133. NbS00004515g0209.1 **Mass:** 96322 **Score:** 44 **Matches:** 2(1) **Sequences:** 2(1) **emPAI:** 0.03
NbS00004515g0209.1 protein AED:0.13 eAED:0.13 QI:829|0.9|0.90|1|0.6|0.63|11|284|876

Query	Observed	Mr(expt)	Mr(calc)	ppm	Miss	Score	Expect	Rank	Unique	Peptide
10771	500.7745	999.5345	999.5349	-0.42	0	31	0.055	1	U	K.GAVENLLER.S
15509	767.8845	1533.7545	1533.7522	1.48	0	44	0.0026	1	U	R.LEQGSLTGESEAVSK.T

Proteins matching the same set of peptides:
NbS00057740g0006.1 **Mass:** 92218 **Score:** 44 **Matches:** 2(1) **Sequences:** 2(1)
NbS00057740g0006.1 protein AED:0.13 eAED:0.15 QI:827|0.69|0.64|1|0.46|0.28|14|279|835; (*GB) gi|147858184|emb|CAN79679.1| (e_value=0.0) hypothetical protein VITISV_034639 [Vitis vinifera];; (*SWP)

134. NbS00002447g0019.1 **Mass:** 51420 **Score:** 44 **Matches:** 1(1) **Sequences:** 1(1) **emPAI:** 0.06
NbS00002447g0019.1 protein AED:0.23 eAED:0.23 QI:0|0.69|0.71|1|0.92|1|14|107|458; (*GB) gi|225451717|ref|XP_002279575.1| (e_value=0.0) PREDICTED: ribosome biogenesis protein WDR12 homolog [Vitis v:

Query	Observed	Mr(expt)	Mr(calc)	ppm	Miss	Score	Expect	Rank	Unique	Peptide
14709	721.4055	1440.7964	1440.7936	1.90	0	44	0.0017	1	U	K.APTTSIAIPSNLTR.F

Proteins matching the same set of peptides:
NbS00014981g0007.1 **Mass:** 48451 **Score:** 44 **Matches:** 1(1) **Sequences:** 1(1)
NbS00014981g0007.1 protein AED:0.23 eAED:0.23 QI:0|0.76|0.78|1|1|1|14|263|431; (*GB) gi|225451717|ref|XP_002279575.1| (e_value=0.0) PREDICTED: ribosome biogenesis protein WDR12 homolog [Vitis vini:

135. NbS00030630g0007.1 **Mass:** 31403 **Score:** 44 **Matches:** 1(1) **Sequences:** 1(1) **emPAI:** 0.11
NbS00030630g0007.1 protein

Query	Observed	Mr(expt)	Mr(calc)	ppm	Miss	Score	Expect	Rank	Unique	Peptide
6494	443.2700	884.5254	884.5192	7.09	1	44	0.0033	1	U	K.ILQRGNGK.W

136. NbS00013799g0011.1 **Mass:** 77842 **Score:** 44 **Matches:** 1(1) **Sequences:** 1(1) **emPAI:** 0.04
NbS00013799g0011.1 protein AED:0.34 eAED:0.34 QI:205|0.88|0.88|1|0.76|0.77|18|0|685; (*GB) gi|225427716|ref|XP_002265086.1| (e_value=0.0) PREDICTED: vacuolar proton translocating ATPase 100 kDa sul

Query	Observed	Mr(expt)	Mr(calc)	ppm	Miss	Score	Expect	Rank	Unique	Peptide
11544	550.8067	1099.5989	1099.5986	0.27	0	44	0.0031	1	U	R.GNLLQTIGER.Y

137. NbS00001525g0121.1 **Mass:** 62315 **Score:** 43 **Matches:** 1(1) **Sequences:** 1(1) **emPAI:** 0.05
NbS00001525g0121.1 protein AED:0.18 eAED:0.18 QI:3|0.62|0.22|1|0.75|0.77|9|0|561

Query	Observed	Mr(expt)	Mr(calc)	ppm	Miss	Score	Expect	Rank	Unique	Peptide
10584	486.7775	971.5405	971.5400	0.55	0	43	0.0039	1	U	K.IGAATALEAR.A

Proteins matching the same set of peptides:

NbS00017675g0018.1 **Mass:** 79118 **Score:** 43 **Matches:** 1(1) **Sequences:** 1(1)
NbS00017675g0018.1 protein AED:0.22 eAED:0.23 QI:3|0.44|0.2|1|0.77|0.8|10|0|714; (*GB) gi|356565758|ref|XP_003551104.1| (e_value=0.0) PREDICTED: lysosomal beta glucosidase-like [Glycine max];; (*SW

---

138. NbS00002899g0003.1 **Mass:** 71041 **Score:** 43 **Matches:** 1(1) **Sequences:** 1(1) **emPAI:** 0.05
NbS00002899g0003.1 protein AED:0.26 eAED:0.26 QI:254|1|0.85|1|1|1|7|0|618; (*GB) gi|147770817|emb|CAN63166.1| (e_value=0.0) hypothetical protein VITISV_040077 [Vitis vinifera];; (*SWP) sp|Q940J9|PM

Query	Observed	Mr(expt)	Mr(calc)	ppm	Miss	Score	Expect	Rank	Unique	Peptide
11997	578.8320	1155.6495	1155.6499	-0.35	0	43	0.0026	1	U	R.DGILLLELDR.V

Proteins matching the same set of peptides:
NbS00005008g0017.1 **Mass:** 77590 **Score:** 43 **Matches:** 1(1) **Sequences:** 1(1)
NbS00005008g0017.1 protein AED:0.20 eAED:0.20 QI:389|0.71|0.62|1|1|1|8|280|667; (*GB) gi|359488775|ref|XP_002271722.2| (e_value=0.0) PREDICTED: probable methyltransferase PMT9-like [Vitis vinifera]
NbS00033243g0006.1 **Mass:** 70994 **Score:** 43 **Matches:** 1(1) **Sequences:** 1(1)
NbS00033243g0006.1 protein AED:0.15 eAED:0.15 QI:0|1|1|1|1|1|7|335|619; (*GB) gi|147770817|emb|CAN63166.1| (e_value=0.0) hypothetical protein VITISV_040077 [Vitis vinifera];; (*SWP) sp|Q940J9|PMT8_
NbS00046503g0001.1 **Mass:** 29765 **Score:** 43 **Matches:** 1(1) **Sequences:** 1(1)
NbS00046503g0001.1 protein AED:0.14 eAED:0.14 QI:0|1|1|1|1|1|2|330|261; (*GB) gi|157849758|gb|ABV89662.1| (e_value=2e-155) dehydration-responsive protein-related [Brassica rapa];; (*SWP) sp|Q8VZV7
NbS00052811g0006.1 **Mass:** 70663 **Score:** 43 **Matches:** 1(1) **Sequences:** 1(1)
NbS00052811g0006.1 protein AED:0.10 eAED:0.10 QI:0|0.85|0.62|1|1|1|8|0|615; (*GB) gi|356501308|ref|XP_003519467.1| (e_value=0.0) PREDICTED: probable methyltransferase PMT8-like [Glycine max];; (*SW
NbS00057125g0003.1 **Mass:** 73477 **Score:** 43 **Matches:** 1(1) **Sequences:** 1(1)
NbS00057125g0003.1 protein AED:0.27 eAED:0.27 QI:284|1|0.75|1|0.85|0.75|8|0|640; (*GB) gi|147770817|emb|CAN63166.1| (e_value=0.0) hypothetical protein VITISV_040077 [Vitis vinifera];; (*SWP) sp|Q9

---

139. NbS00011076g0013.1 **Mass:** 17587 **Score:** 43 **Matches:** 1(1) **Sequences:** 1(1) **emPAI:** 0.19
NbS00011076g0013.1 protein AED:0.25 eAED:0.25 QI:82|1|1|1|1|1|4|551|152; (*GB) gi|40287492|gb|AAR83860.1| (e_value=8e-97) putative ribosomal protein [Capsicum annuum];; (*SWP) sp|P34788|RS18_ARATH

Query	Observed	Mr(expt)	Mr(calc)	ppm	Miss	Score	Expect	Rank	Unique	Peptide
13812	670.8289	1339.6433	1339.6442	-0.68	0	43	0.003	1	U	K.FSQVTSNALDMK.L

Proteins matching the same set of peptides:
NbS00021597g0018.1 **Mass:** 14577 **Score:** 43 **Matches:** 1(1) **Sequences:** 1(1)
NbS00021597g0018.1 protein AED:0.36 eAED:0.36 QI:0|1|0.33|1|1|1|3|0|126; (*GB) gi|76573321|gb|ABA46765.1| (e_value=1e-75) unknown [Solanum tuberosum];; (*SWP) sp|P34788|RS18_ARATH (e_value=7e-73)
NbS00023066g0009.1 **Mass:** 22652 **Score:** 43 **Matches:** 1(1) **Sequences:** 1(1)
NbS00023066g0009.1 protein AED:0.15 eAED:0.15 QI:0|0.75|0.6|1|0.75|0.8|5|433|196; (*GB) gi|40287492|gb|AAR83860.1| (e_value=2e-93) putative ribosomal protein [Capsicum annuum];; (*SWP) sp|P34788|RS
NbS00025041g0010.1 **Mass:** 17560 **Score:** 43 **Matches:** 1(1) **Sequences:** 1(1)
NbS00025041g0010.1 protein AED:0.22 eAED:0.22 QI:85|1|1|1|1|1|4|295|152; (*GB) gi|76573321|gb|ABA46765.1| (e_value=2e-97) unknown [Solanum tuberosum];; (*SWP) sp|P34788|RS18_ARATH (e_value=4e-92)
NbS00056355g0003.1 **Mass:** 17569 **Score:** 43 **Matches:** 1(1) **Sequences:** 1(1)
NbS00056355g0003.1 protein AED:0.03 eAED:0.03 QI:0|0.66|0.25|0.75|1|1|4|0|152; (*SWP) sp|P34788|RS18_ARATH (e_value=4e-92) 40S ribosomal protein S18 OS=Arabidopsis thaliana GN=RPS18A PE=1 SV=1;; (

---

140. NbS00002372g0015.1 **Mass:** 50828 **Score:** 43 **Matches:** 1(1) **Sequences:** 1(1) **emPAI:** 0.06
NbS00002372g0015.1 protein AED:0.22 eAED:0.23 QI:207|0.66|0.5|1|1|1|4|0|452; (*GB) gi|110083391|dbj|BAE97400.1| (e_value=8e-141) heat shock protein 90 [Nicotiana tabacum];; (*SWP) sp|P36181|HSP80_

Query	Observed	Mr(expt)	Mr(calc)	ppm	Miss	Score	Expect	Rank	Unique	Peptide
8328	628.8500	1255.6854	1255.6884	-2.38	0	43	0.003	1	U	K.ADLVNNLGTIAR.S

Proteins matching the same set of peptides:
NbS00006303g0102.1 **Mass:** 37715 **Score:** 43 **Matches:** 1(1) **Sequences:** 1(1)
NbS00006303g0102.1 protein AED:0.26 eAED:0.26 QI:0|0|0|1|1|1|3|0|331
NbS00010856g0013.1 **Mass:** 38170 **Score:** 43 **Matches:** 1(1) **Sequences:** 1(1)
NbS00010856g0013.1 protein AED:0.30 eAED:0.30 QI:0|0.66|0.5|1|1|1|4|0|340; (*GB) gi|118778687|ref|XP_308799.3| (e_value=9e-155) AGAP006959-PA [Anopheles gambiae str. PEST];; (*SWP) sp|P51819|HSP83_
NbS00011481g0021.1 **Mass:** 37362 **Score:** 43 **Matches:** 1(1) **Sequences:** 1(1)
NbS00011481g0021.1 protein AED:0.26 eAED:0.26 QI:148|0.66|0.5|1|1|1|4|0|334; (*GB) gi|118778687|ref|XP_308799.3| (e_value=2e-154) AGAP006959-PA [Anopheles gambiae str. PEST];; (*SWP) sp|P36181|HSP
NbS00014845g0001.1 **Mass:** 52186 **Score:** 43 **Matches:** 1(1) **Sequences:** 1(1)
NbS00014845g0001.1 protein AED:0.19 eAED:0.19 QI:0|0.66|0.25|1|1|1|4|0|461; (*GB) gi|47219165|emb|CAG01828.1| (e_value=1e-174) unnamed protein product [Tetraodon nigroviridis];; (*SWP) sp|P36181|HS
NbS00021897g0010.1 **Mass:** 67830 **Score:** 43 **Matches:** 1(1) **Sequences:** 1(1)
NbS00021897g0010.1 protein AED:0.13 eAED:0.13 QI:0|0.33|0.25|1|0.66|0.5|4|0|595; (*GB) gi|110083391|dbj|BAE97400.1| (e_value=2e-133) heat shock protein 90 [Nicotiana tabacum];; (*SWP) sp|P36181|HS
NbS00025260g0001.1 **Mass:** 52067 **Score:** 43 **Matches:** 1(1) **Sequences:** 1(1)
NbS00025260g0001.1 protein AED:0.23 eAED:0.23 QI:0|0.66|0.25|1|1|1|4|0|460; (*GB) gi|47219165|emb|CAG01828.1| (e_value=3e-173) unnamed protein product [Tetraodon nigroviridis];; (*SWP) sp|P36181|HS
NbS00040564g0006.1 **Mass:** 37610 **Score:** 43 **Matches:** 1(1) **Sequences:** 1(1)
NbS00040564g0006.1 protein AED:0.26 eAED:0.26 QI:149|0.33|0.25|1|1|1|4|0|338; (*GB) gi|158286538|ref|XP_308797.3| (e_value=1e-134) AGAP006961-PA [Anopheles gambiae str. PEST];; (*SWP) sp|P36181|HS
NbS00047419g0011.1 **Mass:** 34548 **Score:** 43 **Matches:** 1(1) **Sequences:** 1(1)

NbS00047419g0011.1 protein AED:0.33 eAED:0.33 QI:0|0.66|0.5|1|0.66|0.75|4|0|310; (*GB) gi|255545176|ref|XP_002513649.1| (e_value=1e-131) heat shock protein, putative [Ricinus communis];; (*SWP) sp

---

141. NbC24809697g0001.1 **Mass:** 18911 **Score:** 43 **Matches:** 1(1) **Sequences:** 1(1) **emPAI:** 0.18
NbC24809697g0001.1 protein ; (*GB) gi|324388030|gb|ADY38792.1| (e_value=1e-59) hypothetical protein MA29G21.11 [Coffea arabica];; (*TAIR) AT5G28500.2 (e_value=1e-52) | Symbols: | unknown protein; I

Query	Observed	Mr(expt)	Mr(calc)	ppm	Miss	Score	Expect	Rank	Unique	Peptide
19990	1132.5487	2263.0829	2263.0968	-6.17	0	43	0.0023	1	U	R.EGFTPSTLEEITGLTGVDQNR.L

---

142. NbS00002401g0015.1 **Mass:** 115645 **Score:** 42 **Matches:** 1(1) **Sequences:** 1(1) **emPAI:** 0.03
NbS00002401g0015.1 protein AED:0.14 eAED:0.14 QI:244|0.81|0.82|1|0.77|0.78|28|441|989; (*SWP) sp|Q8RWY3|ISW2_ARATH (e_value=0.0) Putative chromatin-remodeling complex ATPase chain OS=Arabidopsis th

Query	Observed	Mr(expt)	Mr(calc)	ppm	Miss	Score	Expect	Rank	Unique	Peptide
13398	648.8870	1295.7595	1295.7561	2.61	0	42	0.0013	1	U	K.LALDALVIQQGR.L

Proteins matching the same set of peptides:
NbS00023277g0001.1 **Mass:** 123826 **Score:** 42 **Matches:** 1(1) **Sequences:** 1(1)
NbS00023277g0001.1 protein AED:0.17 eAED:0.17 QI:296|0.78|0.70|1|0.95|0.91|24|69|1063; (*GB) gi|255551667|ref|XP_002516879.1| (e_value=0.0) helicase, putative [Ricinus communis];; (*SWP) sp|Q8RWY3
NbS00054126g0008.1 **Mass:** 124250 **Score:** 42 **Matches:** 1(1) **Sequences:** 1(1)
NbS00054126g0008.1 protein AED:0.07 eAED:0.07 QI:135|0.95|0.96|1|0.91|1|25|358|1063; (*GB) gi|255551667|ref|XP_002516879.1| (e_value=0.0) helicase, putative [Ricinus communis];; (*SWP) sp|Q8RWY3|IS

---

143. NbS00045861g0008.1 **Mass:** 37461 **Score:** 42 **Matches:** 1(1) **Sequences:** 1(1) **emPAI:** 0.09
NbS00045861g0008.1 protein AED:0.26 eAED:0.26 QI:38|1|0.91|1|0.54|0.75|12|0|334; (*GB) gi|225459625|ref|XP_002285874.1| (e_value=0.0) PREDICTED: replication factor C subunit 4 isoform 1 [Vitis vin

Query	Observed	Mr(expt)	Mr(calc)	ppm	Miss	Score	Expect	Rank	Unique	Peptide
11376	539.7861	1077.5577	1077.5567	0.94	0	42	0.0048	1	U	K.NFAAVAVGSSR.Q

Proteins matching the same set of peptides:
NbS00054666g0018.1 **Mass:** 38823 **Score:** 42 **Matches:** 1(1) **Sequences:** 1(1)
NbS00054666g0018.1 protein AED:0.30 eAED:0.31 QI:0|0.81|0.75|0.91|0.72|0.83|12|0|348; (*GB) gi|255558610|ref|XP_002520330.1| (e_value=0.0) replication factor C / DNA polymerase III gamma-tau subun

---

144. NbS00005183g0002.1 **Mass:** 64758 **Score:** 42 **Matches:** 1(1) **Sequences:** 1(1) **emPAI:** 0.05
NbS00005183g0002.1 protein AED:0.18 eAED:0.18 QI:0|0.69|0.57|0.92|1|1|14|0|607; (*GB) gi|1762130|gb|AAB39827.1| (e_value=0.0) chaperonin-60 beta subunit [Solanum tuberosum];; (*SWP) sp|P08927|RUBB_

Query	Observed	Mr(expt)	Mr(calc)	ppm	Miss	Score	Expect	Rank	Unique	Peptide
12277	591.8600	1181.7054	1181.7020	2.91	0	42	0.0016	1	U	K.LADLVGVTLGPK.G

Proteins matching the same set of peptides:
NbS00034791g0001.1 **Mass:** 64590 **Score:** 42 **Matches:** 1(1) **Sequences:** 1(1)
NbS00034791g0001.1 protein AED:0.18 eAED:0.18 QI:0|0.73|0.68|1|1|1|16|447|608; (*GB) gi|1762130|gb|AAB39827.1| (e_value=0.0) chaperonin-60 beta subunit [Solanum tuberosum];; (*SWP) sp|Q9LJE4|CPNB2_

---

145. gi|355708779|gb|AES03376.1| **Mass:** 21988 **Score:** 42 **Matches:** 2(2) **Sequences:** 2(2) **emPAI:** 0.33
gi|355708779|gb|AES03376.1| Group Os04g0486600 [Mustela putorius furo]

Query	Observed	Mr(expt)	Mr(calc)	ppm	Miss	Score	Expect	Rank	Unique	Peptide
9534	417.2343	832.4540	832.4555	-1.90	0	36	0.023	1	U	K.IGINGFGR.I
13485	653.3286	1304.6427	1304.6435	-0.62	0	35	0.026	1	U	K.DAPMFVVGVNEK.E

---

146. NbS00001900g0005.1 **Mass:** 38043 **Score:** 42 **Matches:** 1(1) **Sequences:** 1(1) **emPAI:** 0.09
NbS00001900g0005.1 protein AED:0.34 eAED:0.34 QI:0|0|0|0.75|1|1|4|0|334; (*GB) gi|18000042|gb|AAL54878.1|AF004232_1 (e_value=2e-50) hydroxy-methyl-glutaryl-coenzyme A reductase [Nicotiana tabacum];

Query	Observed	Mr(expt)	Mr(calc)	ppm	Miss	Score	Expect	Rank	Unique	Peptide
9471	412.7709	823.5272	823.5531	-31.46	0	42	0.00082	1	U	R.LAALLPVK.M

---

147. NbS00011440g0007.1 **Mass:** 66640 **Score:** 42 **Matches:** 1(1) **Sequences:** 1(1) **emPAI:** 0.05
NbS00011440g0007.1 protein AED:0.30 eAED:0.32 QI:0|0.87|0.55|1|0.87|0.88|9|0|608; (*TAIR) AT1G67840.1 (e_value=0.0) | Symbols: CSK | chloroplast sensor kinase | chr1:25434156-25436839 FORWARD LENGT

Query	Observed	Mr(expt)	Mr(calc)	ppm	Miss	Score	Expect	Rank	Unique	Peptide
8849	360.1958	718.3770	718.3973	-28.33	0	42	0.0096	1	U	R.GSLSSIR.T

Proteins matching the same set of peptides:

NbS00017144g0007.1 **Score:** 42 **Matches:** 1(1) **Sequences:** 1(1)

---

148. NbS00000172g0008.1 **Mass:** 28107 **Score:** 41 **Matches:** 1(1) **Sequences:** 1(1) **emPAI:** 0.12
NbS00000172g0008.1 protein AED:0.09 eAED:0.09 QI:94|0.66|0.75|1|0.33|0|4|260|260; (*SWP) sp|P0C582|M2OM_NEUCR (e_value=7e-53) Putative mitochondrial 2-oxoglutarate/malate carrier protein OS=Neurosp

Query	Observed	Mr(expt)	Mr(calc)	ppm	Miss	Score	Expect	Rank	Unique	Peptide
13567	**657.8460**	**1313.6775**	**1313.6761**	**1.00**	**0**	**41**	**0.0051**	**1**	**U**	**R.MQADATLPLAQR.R**

Proteins matching the same set of peptides:
NbS00003287g0001.1 **Mass:** 29238 **Score:** 41 **Matches:** 1(1) **Sequences:** 1(1)
NbS00003287g0001.1 protein AED:0.38 eAED:0.38 QI:0|0.75|0.4|1|1|1|5|0|271; (*GB) gi|19913105|emb|CAC84545.1| (e_value=0.0) dicarboxylate/tricarboxylate carrier [Nicotiana tabacum];; (*SWP) sp|P0C58
NbS00056566g0003.1 **Mass:** 32051 **Score:** 41 **Matches:** 1(1) **Sequences:** 1(1)
NbS00056566g0003.1 protein AED:0.31 eAED:0.31 QI:246|1|1|1|1|1|6|596|297; (*GB) gi|19913105|emb|CAC84545.1| (e_value=0.0) dicarboxylate/tricarboxylate carrier [Nicotiana tabacum];; (*SWP) sp|P0C582

---

149. NbS00016433g0017.1 **Mass:** 41368 **Score:** 41 **Matches:** 1(1) **Sequences:** 1(1) **emPAI:** 0.08
NbS00016433g0017.1 protein AED:0.23 eAED:0.24 QI:195|1|0.9|1|1|1|10|0|371; (*GB) gi|255543841|ref|XP_002512983.1| (e_value=0.0) clathrin binding protein, putative [Ricinus communis];; (*TAIR) AT4G3

Query	Observed	Mr(expt)	Mr(calc)	ppm	Miss	Score	Expect	Rank	Unique	Peptide
11983	**577.8298**	**1153.6450**	**1153.6455**	**-0.45**	**0**	**41**	**0.0032**	**1**	**U**	**K.ALGTQVAEPLR.A**

Proteins matching the same set of peptides:
NbS00019391g0012.1 **Mass:** 41160 **Score:** 41 **Matches:** 1(1) **Sequences:** 1(1)
NbS00019391g0012.1 protein AED:0.09 eAED:0.09 QI:72|0.88|0.9|1|0.88|1|10|207|369; (*TAIR) AT4G34660.1 (e_value=0.0) | Symbols: | SH3 domain-containing protein | chr4:16545595-16548294 RVERSE LENGTH
NbS00031419g0012.1 **Mass:** 36635 **Score:** 41 **Matches:** 1(1) **Sequences:** 1(1)
NbS00031419g0012.1 protein AED:0.19 eAED:0.19 QI:79|0.66|0.6|1|0.77|0.8|10|0|329; (*GB) gi|255543841|ref|XP_002512983.1| (e_value=4e-160) clathrin binding protein, putative [Ricinus communis];; (*T

---

150. NbS00007805g0017.1 **Mass:** 45463 **Score:** 41 **Matches:** 1(1) **Sequences:** 1(1) **emPAI:** 0.07
NbS00007805g0017.1 protein AED:0.20 eAED:0.20 QI:0|1|0.6|1|1|1|5|0|406; (*GB) gi|255555933|ref|XP_002519002.1| (e_value=0.0) phosphoribulose kinase, putative [Ricinus communis];; (*SWP) sp|P26302|I

Query	Observed	Mr(expt)	Mr(calc)	ppm	Miss	Score	Expect	Rank	Unique	Peptide
14941	**736.8389**	**1471.6632**	**1471.6653**	**-1.45**	**0**	**41**	**0.0029**	**1**	**U**	**R.ANDFDLMYEQVK.A**

Proteins matching the same set of peptides:
NbS00031448g0006.1 **Mass:** 52071 **Score:** 41 **Matches:** 1(1) **Sequences:** 1(1)
NbS00031448g0006.1 protein AED:0.22 eAED:0.24 QI:0|0.4|0.33|1|1|1|6|0|464; (*GB) gi|224138316|ref|XP_002326572.1| (e_value=0.0) predicted protein [Populus trichocarpa];; (*SWP) sp|P25697|KPPR_ARATH

---

151. NbS00033277g0006.1 **Score:** 41 **Matches:** 1(1) **Sequences:** 1(1) **emPAI:** 0.26
NbS00033277g0006.1 protein AED:0.56 eAED:0.56 QI:0|0|0|0.16|1|1|6|0|118

Query	Observed	Mr(expt)	Mr(calc)	ppm	Miss	Score	Expect	Rank	Unique	Peptide
9511	416.2498	830.4850	830.4861	-1.35	0	41	0.0062	2	U	K.SETLILR.Q

---

152. NbS00013334g0009.1 **Score:** 41 **Matches:** 1(1) **Sequences:** 1(1) **emPAI:** 0.04
NbS00013334g0009.1 protein AED:0.05 eAED:0.05 QI:325|0.71|0.75|1|0.57|0.62|8|274|699; (*GB) gi|297742094|emb|CBI33881.3| (e_value=3e-155) unnamed protein product [Vitis vinifera];; (*SWP) sp|Q9FPS

Query	Observed	Mr(expt)	Mr(calc)	ppm	Miss	Score	Expect	Rank	Unique	Peptide
10945	511.2694	1020.5242	1020.4845	39.0	0	41	0.0074	2	U	R.GQQLMAAMR.G

---

153. NbS00030026g0014.1 **Mass:** 128602 **Score:** 41 **Matches:** 1(1) **Sequences:** 1(1) **emPAI:** 0.03
NbS00030026g0014.1 protein AED:0.18 eAED:0.23 QI:0|0.66|0.52|1|0.66|0.72|25|0|1122; (*GB) gi|68300799|gb|AAY89342.1| (e_value=0.0) RNA polymerase II second largest subunit [Nicotiana sylvestris];;

Query	Observed	Mr(expt)	Mr(calc)	ppm	Miss	Score	Expect	Rank	Unique	Peptide
20016	**758.0761**	**2271.2063**	**2271.1973**	**3.97**	**0**	**41**	**0.0029**	**1**	**U**	**R.MDTLAYVLYYPQKPLVTTR.A**

Proteins matching the same set of peptides:
NbS00031742g0005.1 **Mass:** 137187 **Score:** 41 **Matches:** 1(1) **Sequences:** 1(1)
NbS00031742g0005.1 protein AED:0.08 eAED:0.08 QI:0|0.88|0.84|1|0.83|0.89|19|0|1197; (*GB) gi|350534526|ref|NP_001233889.1| (e_value=0.0) DNA-directed RNA polymerase II subunit RPB2 [Solanum lycope
NbS00046031g0008.1 **Mass:** 140521 **Score:** 41 **Matches:** 1(1) **Sequences:** 1(1)
NbS00046031g0008.1 protein AED:0.09 eAED:0.09 QI:0|0.94|0.85|1|0.84|0.85|20|0|1226; (*GB) gi|350534526|ref|NP_001233889.1| (e_value=0.0) DNA-directed RNA polymerase II subunit RPB2 [Solanum lycope

---

154. NbS00000529g0002.1 **Mass:** 12164 **Score:** 41 **Matches:** 2(2) **Sequences:** 1(1) **emPAI:** 0.28
NbS00000529g0002.1 protein AED:0.44 eAED:0.48 QI:0|0.66|0.5|1|1|1|4|0|104; (*GB) gi|224068340|ref|XP_002302713.1| (e_value=7e-69) predicted protein [Populus trichocarpa];; (*SWP) sp|Q9VI10|SMD2_DRO

Query	Observed	Mr(expt)	Mr(calc)	ppm	Miss	Score	Expect	Rank	Unique	Peptide
12666	616.3148	1230.6150	1230.6139	0.87	0	35	0.027	1	U	K.NNTQVLINCR.N 12665

Proteins matching the same set of peptides:
NbS00008935g0003.1 **Mass:** 12678 **Score:** 41 **Matches:** 2(2) **Sequences:** 1(1)
NbS00008935g0003.1 protein AED:0.00 eAED:0.00 QI:0|-1|0|1|-1|1|1|0|108; (*GB) gi|224068340|ref|XP_002302713.1| (e_value=2e-71) predicted protein [Populus trichocarpa];; (*SWP) sp|Q9VI10|SMD2_DROME
NbS00010757g0002.1 **Mass:** 12636 **Score:** 41 **Matches:** 2(2) **Sequences:** 1(1)
NbS00010757g0002.1 protein AED:0.41 eAED:0.42 QI:185|1|0.6|1|1|1|5|0|108; (*GB) gi|224068340|ref|XP_002302713.1| (e_value=5e-72) predicted protein [Populus trichocarpa];; (*SWP) sp|Q9VI10|SMD2_DRO

---

155. NbS00001711g0013.1 **Mass:** 36166 **Score:** 40 **Matches:** 1(1) **Sequences:** 1(1) **emPAI:** 0.09
NbS00001711g0013.1 protein AED:0.26 eAED:0.26 QI:422|0.85|0.87|1|0.85|0.75|8|401|334; (*TAIR) AT4G17520.1 (e_value=2e-31) | Symbols: | Hyaluronan / mRNA binding family | chr4:9771496-9773313 FORWAR

Query	Observed	Mr(expt)	Mr(calc)	ppm	Miss	Score	Expect	Rank	Unique	Peptide
11560	551.7996	1101.5846	1101.5852	-0.56	0	40	0.0085	1	U	K.GLLALMAEER.K

---

156. NbS00009108g0010.1 **Mass:** 46202 **Score:** 40 **Matches:** 1(1) **Sequences:** 1(1) **emPAI:** 0.07
NbS00009108g0010.1 protein AED:0.35 eAED:0.35 QI:142|0.5|0.33|1|1|0.66|3|0|414; (*GB) gi|255549002|ref|XP_002515557.1| (e_value=0.0) conserved hypothetical protein [Ricinus communis];; (*TAIR) AT5(

Query	Observed	Mr(expt)	Mr(calc)	ppm	Miss	Score	Expect	Rank	Unique	Peptide
19304	1028.9618	2055.9090	2055.8990	4.89	0	40	0.0033	1	U	K.VGDASNSCEGSMIVEAFAGR.D

Proteins matching the same set of peptides:
NbC25222459g0001.1 **Mass:** 12824 **Score:** 40 **Matches:** 1(1) **Sequences:** 1(1)
NbC25222459g0001.1 protein ; (*GB) gi|255549002|ref|XP_002515557.1| (e_value=2e-68) conserved hypothetical protein [Ricinus communis];; (*TAIR) AT5G11420.1 (e_value=5e-64) | Symbols: | Protein of u

---

157. NbS00001513g0001.1 **Mass:** 63897 **Score:** 40 **Matches:** 1(1) **Sequences:** 1(1) **emPAI:** 0.05
NbS00001513g0001.1 protein AED:0.13 eAED:0.14 QI:0|0|0|1|1|1|2|0|575; (*GB) gi|147792200|emb|CAN62034.1| (e_value=8e-171) hypothetical protein VITISV_014731 [Vitis vinifera];; (*SWP) sp|F4K1B1|RPA

Query	Observed	Mr(expt)	Mr(calc)	ppm	Miss	Score	Expect	Rank	Unique	Peptide
12170	587.3185	1172.6225	1172.6401	-14.96	1	40	0.0089	1	U	K.ENGDLGLSKLK.I

Proteins matching the same set of peptides:
NbS00023022g0001.1 **Mass:** 76447 **Score:** 40 **Matches:** 1(1) **Sequences:** 1(1)
NbS00023022g0001.1 protein AED:0.22 eAED:0.22 QI:0|0.8|0.66|1|1|1|6|0|685; (*GB) gi|147792200|emb|CAN62034.1| (e_value=0.0) hypothetical protein VITISV_014731 [Vitis vinifera];; (*SWP) sp|F4K1B1|RI
NbS00053401g0008.1 **Mass:** 46469 **Score:** 40 **Matches:** 1(1) **Sequences:** 1(1)
NbS00053401g0008.1 protein AED:0.36 eAED:0.36 QI:0|0.5|0|1|0|0.2|5|0|414; (*GB) gi|147792200|emb|CAN62034.1| (e_value=8e-100) hypothetical protein VITISV_014731 [Vitis vinifera];; (*SWP) sp|F4K1B1

---

158. NbS00034421g0001.1 **Mass:** 65483 **Score:** 40 **Matches:** 1(1) **Sequences:** 1(1) **emPAI:** 0.05
NbS00034421g0001.1 protein AED:0.12 eAED:0.20 QI:0|0|0|1|1|1|3|0|591; (*GB) gi|225448505|ref|XP_002272996.1| (e_value=0.0) PREDICTED: U-box domain-containing protein 13 [Vitis vinifera];; (*SWP) sp

Query	Observed	Mr(expt)	Mr(calc)	ppm	Miss	Score	Expect	Rank	Unique	Peptide
10797	502.2994	1002.5842	1002.5896	-5.34	1	40	0.0062	2	U	R.LMIKQNLK.Q

Proteins matching the same set of peptides:
NbS00048470g0001.1 **Score:** 40 **Matches:** 1(1) **Sequences:** 1(1)

---

159. NbS00000131g0001.1 **Mass:** 24798 **Score:** 39 **Matches:** 1(1) **Sequences:** 1(1) **emPAI:** 0.14
NbS00000131g0001.1 protein AED:0.33 eAED:0.33 QI:158|1|1|1|1|1|2|235|224; (*GB) gi|3024505|sp|Q40522.1|RB11D_TOBAC (e_value=2e-152) RecName: Full=Ras-related protein Rab11D;; (*SWP) sp|Q40522|RB11I

Query	Observed	Mr(expt)	Mr(calc)	ppm	Miss	Score	Expect	Rank	Unique	Peptide
13089	637.8094	1273.6043	1273.6051	-0.60	0	39	0.0071	1	U	K.AQIWDTAGQER.Y

Proteins matching the same set of peptides:
NbS00000385g0008.1 **Mass:** 22296 **Score:** 39 **Matches:** 1(1) **Sequences:** 1(1)
NbS00000385g0008.1 protein AED:0.09 eAED:0.09 QI:0|0.5|0|1|1|1|3|0|201; (*SWP) sp|Q01111|YPT3_NICPL (e_value=3e-141) Ras-related protein YPT3 OS=Nicotiana plumbaginifolia GN=YPT3 PE=2 SV=1;; (*TAII
NbS00000538g0012.1 **Mass:** 25089 **Score:** 39 **Matches:** 1(1) **Sequences:** 1(1)
NbS00000538g0012.1 protein AED:0.16 eAED:0.16 QI:824|1|1|1|0.5|0.33|3|357|225; (*GB) gi|224139494|ref|XP_002323139.1| (e_value=3e-141) predicted protein [Populus trichocarpa];; (*SWP) sp|Q9FGK5|RA

NbS00001483g0110.1 **Mass:** 27762 **Score:** 39 **Matches:** 1(1) **Sequences:** 1(1)
NbS00001483g0110.1 protein AED:0.04 eAED:0.04 QI:309|1|1|1|1|1|2|195|250
NbS00001567g0018.1 **Mass:** 24238 **Score:** 39 **Matches:** 1(1) **Sequences:** 1(1)
NbS00001567g0018.1 protein AED:0.11 eAED:0.11 QI:37|1|1|1|1|1|2|256|217; (*GB) gi|225443742|ref|XP_002268815.1| (e_value=4e-140) PREDICTED: ras-related protein RABA1d [Vitis vinifera];; (*SWP) sp|(
NbS00001679g0002.1 **Mass:** 11279 **Score:** 39 **Matches:** 1(1) **Sequences:** 1(1)
NbS00001679g0002.1 protein AED:0.44 eAED:0.65 QI:0|0|0|0.5|1|1|2|0|103; (*SWP) sp|Q40191|RB11A_LOTJA (e_value=5e-47) Ras-related protein Rab11A OS=Lotus japonicus GN=RAB11A PE=2 SV=1;; (*TAIR) AT5(
NbS00002322g0004.1 **Mass:** 29739 **Score:** 39 **Matches:** 1(1) **Sequences:** 1(1)
NbS00002322g0004.1 protein AED:0.27 eAED:0.28 QI:0|1|0.5|1|1|1|2|0|265; (*GB) gi|356538966|ref|XP_003537971.1| (e_value=6e-130) PREDICTED: ras-related protein Rab11C-like [Glycine max];; (*SWP) sp
NbS00002579g0015.1 **Mass:** 25976 **Score:** 39 **Matches:** 1(1) **Sequences:** 1(1)
NbS00002579g0015.1 protein AED:0.17 eAED:0.17 QI:0|0.5|0.33|1|0|0.33|3|264|232; (*GB) gi|15230422|ref|NP_187823.1| (e_value=8e-137) RAB GTPase-like protein A4D [Arabidopsis thaliana];; (*SWP) sp|Q
NbS00002846g0005.1 **Mass:** 24122 **Score:** 39 **Matches:** 1(1) **Sequences:** 1(1)
NbS00002846g0005.1 protein AED:0.03 eAED:0.03 QI:0|1|0.5|1|1|1|2|0|216; (*GB) gi|255568357|ref|XP_002525153.1| (e_value=2e-143) protein with unknown function [Ricinus communis];; (*SWP) sp|Q9FJH0|I
NbS00003427g0011.1 **Mass:** 25483 **Score:** 39 **Matches:** 1(1) **Sequences:** 1(1)
NbS00003427g0011.1 protein AED:0.12 eAED:0.12 QI:155|1|1|1|1|1|2|162|225; (*GB) gi|224135525|ref|XP_002322095.1| (e_value=2e-124) predicted protein [Populus trichocarpa];; (*SWP) sp|Q9C9U7|RAA6A_AI
NbS00003555g0011.1 **Mass:** 24506 **Score:** 39 **Matches:** 1(1) **Sequences:** 1(1)
NbS00003555g0011.1 protein AED:0.35 eAED:0.35 QI:130|1|1|1|1|1|2|207|218; (*GB) gi|623584|gb|AAA74116.1| (e_value=8e-153) putative, partial [Nicotiana tabacum];; (*SWP) sp|Q9SIP0|RAA5D_ARATH (e_va]
NbS00005133g0002.1 **Mass:** 26462 **Score:** 39 **Matches:** 1(1) **Sequences:** 1(1)
NbS00005133g0002.1 protein AED:0.28 eAED:0.28 QI:0|0|0|0.66|1|1|3|0|236; (*GB) gi|255570960|ref|XP_002526431.1| (e_value=5e-136) protein with unknown function [Ricinus communis];; (*SWP) sp|O04486
NbS00006900g0014.1 **Mass:** 24444 **Score:** 39 **Matches:** 1(1) **Sequences:** 1(1)
NbS00006900g0014.1 protein AED:0.21 eAED:0.21 QI:371|1|1|1|1|1|2|561|217; (*SWP) sp|Q40521|RB11B_TOBAC (e_value=1e-150) Ras-related protein Rab11B OS=Nicotiana tabacum GN=RAB11B PE=2 SV=1;; (*TAIR)
NbS00007216g0004.1 **Mass:** 24091 **Score:** 39 **Matches:** 1(1) **Sequences:** 1(1)
NbS00007216g0004.1 protein AED:0.32 eAED:0.32 QI:0|0|0|1|0|0.5|2|0|217; (*SWP) sp|Q9FGK5|RAA5A_ARATH (e_value=7e-128) Ras-related protein RABA5a OS=Arabidopsis thaliana GN=RABA5A PE=2 SV=1;; (*TAII
NbS00008309g0123.1 **Mass:** 24207 **Score:** 39 **Matches:** 1(1) **Sequences:** 1(1)
NbS00008309g0123.1 protein AED:0.05 eAED:0.05 QI:0|1|0|1|0|0.5|2|0|216
NbS00008330g0011.1 **Mass:** 24310 **Score:** 39 **Matches:** 1(1) **Sequences:** 1(1)
NbS00008330g0011.1 protein AED:0.10 eAED:0.10 QI:118|1|1|1|1|1|2|517|218; (*GB) gi|267527|sp|Q01111.1|YPT3_NICPL (e_value=2e-160) RecName: Full=Ras-related protein YPT3;; (*SWP) sp|Q01111|YPT3_NICI
NbS00010093g0004.1 **Mass:** 24980 **Score:** 39 **Matches:** 1(1) **Sequences:** 1(1)
NbS00010093g0004.1 protein AED:0.00 eAED:0.00 QI:427|1|1|1|1|1|2|453|225; (*SWP) sp|P25766|RLGP1_ORYSJ (e_value=9e-130) Ras-related protein RGP1 OS=Oryza sativa subsp. japonica GN=RGP1 PE=2 SV=2;;
NbS00010683g0013.1 **Mass:** 24400 **Score:** 39 **Matches:** 1(1) **Sequences:** 1(1)
NbS00010683g0013.1 protein AED:0.18 eAED:0.18 QI:194|1|1|1|1|1|2|448|217; (*GB) gi|3024504|sp|Q40521.1|RB11B_TOBAC (e_value=6e-158) RecName: Full=Ras-related protein Rab11B;; (*SWP) sp|Q40521|RB11I
NbS00011637g0008.1 **Mass:** 24229 **Score:** 39 **Matches:** 1(1) **Sequences:** 1(1)
NbS00011637g0008.1 protein AED:0.37 eAED:0.37 QI:0|1|0.5|1|1|1|2|0|217; (*SWP) sp|Q9FK68|RAA1C_ARATH (e_value=2e-138) Ras-related protein RABA1c OS=Arabidopsis thaliana GN=RABA1C PE=1 SV=1;; (*TAII
NbS00015488g0001.1 **Mass:** 24198 **Score:** 39 **Matches:** 1(1) **Sequences:** 1(1)
NbS00015488g0001.1 protein AED:0.07 eAED:0.07 QI:0|1|0.5|1|1|1|2|0|216; (*GB) gi|255568357|ref|XP_002525153.1| (e_value=2e-142) protein with unknown function [Ricinus communis];; (*SWP) sp|Q9FJH0|I
NbS00016997g0006.1 **Mass:** 25214 **Score:** 39 **Matches:** 1(1) **Sequences:** 1(1)
NbS00016997g0006.1 protein AED:0.06 eAED:0.06 QI:0|1|0.5|1|1|1|2|0|225; (*GB) gi|15230422|ref|NP_187823.1| (e_value=4e-132) RAB GTPase-like protein A4D [Arabidopsis thaliana];; (*SWP) sp|Q9LH50|RAI
NbS00019047g0011.1 **Mass:** 24013 **Score:** 39 **Matches:** 1(1) **Sequences:** 1(1)
NbS00019047g0011.1 protein AED:0.35 eAED:0.35 QI:0|1|0.5|1|1|1|2|0|216; (*GB) gi|3024506|sp|Q40523.1|RB11A_TOBAC (e_value=7e-140) RecName: Full=Ras-related protein Rab11A;; (*SWP) sp|Q40523|RB11A_[illegible]
NbS00019362g0006.1 **Mass:** 23912 **Score:** 39 **Matches:** 1(1) **Sequences:** 1(1)
NbS00019362g0006.1 protein AED:0.31 eAED:0.31 QI:0|1|1|1|1|1|0.5|2|0|214; (*GB) gi|255568357|ref|XP_002525153.1| (e_value=7e-140) protein with unknown function [Ricinus communis];; (*SWP) sp|Q9FJH0|I
NbS00022531g0003.1 **Mass:** 27867 **Score:** 39 **Matches:** 1(1) **Sequences:** 1(1)
NbS00022531g0003.1 protein AED:0.02 eAED:0.02 QI:240|1|1|1|1|1|2|185|250; (*GB) gi|356559913|ref|XP_003548240.1| (e_value=6e-129) PREDICTED: ras-related protein RABA3-like [Glycine max];; (*SWP) sp
NbS00023419g0305.1 **Mass:** 24096 **Score:** 39 **Matches:** 1(1) **Sequences:** 1(1)
NbS00023419g0305.1 protein AED:0.07 eAED:0.07 QI:0|1|0|1|1|1|2|0|214
NbS00025740g0001.1 **Mass:** 24065 **Score:** 39 **Matches:** 1(1) **Sequences:** 1(1)
NbS00025740g0001.1 protein AED:0.25 eAED:0.25 QI:0|1|0.5|1|1|1|2|0|216; (*SWP) sp|Q40193|RB11C_LOTJA (e_value=4e-143) Ras-related protein Rab11C OS=Lotus japonicus GN=RAB11C PE=2 SV=1;; (*TAIR) AT1
NbS00025808g0010.1 **Mass:** 24519 **Score:** 39 **Matches:** 1(1) **Sequences:** 1(1)
NbS00025808g0010.1 protein AED:0.02 eAED:0.02 QI:182|1|1|1|1|1|2|202|218; (*GB) gi|623584|gb|AAA74116.1| (e_value=3e-152) putative, partial [Nicotiana tabacum];; (*SWP) sp|Q9SIP0|RAA5D_ARATH (e_va]
NbS00026726g0021.1 **Mass:** 23963 **Score:** 39 **Matches:** 1(1) **Sequences:** 1(1)
NbS00026726g0021.1 protein AED:0.33 eAED:0.33 QI:0|1|0.5|1|1|1|2|0|216; (*GB) gi|3024506|sp|Q40523.1|RB11A_TOBAC (e_value=1e-142) RecName: Full=Ras-related protein Rab11A;; (*SWP) sp|Q40523|RB11A_[illegible]
NbS00027690g0004.1 **Mass:** 24023 **Score:** 39 **Matches:** 1(1) **Sequences:** 1(1)
NbS00027690g0004.1 protein AED:0.04 eAED:0.04 QI:0|1|0|1|1|1|2|0|214; (*GB) gi|255566524|ref|XP_002524247.1| (e_value=4e-137) protein with unknown function [Ricinus communis];; (*SWP) sp|Q40193|RB]
NbS00031627g0001.1 **Mass:** 28097 **Score:** 39 **Matches:** 1(1) **Sequences:** 1(1)
NbS00031627g0001.1 protein AED:0.48 eAED:0.48 QI:0|1|0.5|1|1|1|2|0|254; (*GB) gi|224139494|ref|XP_002323139.1| (e_value=8e-136) predicted protein [Populus trichocarpa];; (*SWP) sp|Q9FGK5|RAA5A_ARA[illegible]
NbS00035925g0004.1 **Mass:** 24277 **Score:** 39 **Matches:** 1(1) **Sequences:** 1(1)
NbS00035925g0004.1 protein AED:0.23 eAED:0.23 QI:0|1|0.5|1|1|1|2|0|217; (*GB) gi|225443554|ref|XP_002277579.1| (e_value=8e-152) PREDICTED: ras-related protein RABA1f [Vitis vinifera];; (*SWP) sp|Q
NbS00036841g0006.1 **Mass:** 14114 **Score:** 39 **Matches:** 1(1) **Sequences:** 1(1)
NbS00036841g0006.1 protein AED:0.17 eAED:0.17 QI:0|0|0|1|1|1|2|0|126; (*GB) gi|224139494|ref|XP_002323139.1| (e_value=3e-61) predicted protein [Populus trichocarpa];; (*SWP) sp|Q9FGK5|RAA5A_ARATH

NbS00038712g0001.1 **Mass:** 24081 **Score:** 39 **Matches:** 1(1) **Sequences:** 1(1)
NbS00038712g0001.1 protein AED:0.04 eAED:0.04 QI:0|1|0|1|1|1|2|0|214; (*GB) gi|255566524|ref|XP_002524247.1| (e_value=3e-136) protein with unknown function [Ricinus communis];; (*SWP) sp|Q39434|RB2
NbS00050706g0003.1 **Mass:** 31828 **Score:** 39 **Matches:** 1(1) **Sequences:** 1(1)
NbS00050706g0003.1 protein AED:0.14 eAED:0.14 QI:0|0.5|0.33|1|1|1|3|0|282; (*SWP) sp|Q9SIP0|RAA5D_ARATH (e_value=5e-97) Ras-related protein RABA5d OS=Arabidopsis thaliana GN=RABA5D PE=2 SV=1;; (*TA
NbS00056647g0007.1 **Mass:** 24034 **Score:** 39 **Matches:** 1(1) **Sequences:** 1(1)
NbS00056647g0007.1 protein AED:0.33 eAED:0.33 QI:262|1|1|1|1|1|2|256|215; (*GB) gi|255570960|ref|XP_002526431.1| (e_value=2e-141) protein with unknown function [Ricinus communis];; (*SWP) sp|O0448
NbS00057294g0003.1 **Mass:** 24691 **Score:** 39 **Matches:** 1(1) **Sequences:** 1(1)
NbS00057294g0003.1 protein AED:0.40 eAED:0.40 QI:0|1|0|1|1|1|2|0|222; (*GB) gi|226088538|dbj|BAH37015.1| (e_value=9e-164) NtRab11D [Nicotiana tabacum];; (*SWP) sp|Q40522|RB11D_TOBAC (e_value=1e-16
NbS00059792g0002.1 **Mass:** 27384 **Score:** 39 **Matches:** 1(1) **Sequences:** 1(1)
NbS00059792g0002.1 protein AED:0.04 eAED:0.04 QI:0|1|0.5|1|1|1|2|0|247; (*GB) gi|226088538|dbj|BAH37015.1| (e_value=1e-156) NtRab11D [Nicotiana tabacum];; (*SWP) sp|Q40522|RB11D_TOBAC (e_value=1e-1

---

160. NbS00010015g0012.1 **Mass:** 64087 **Score:** 39 **Matches:** 1(1) **Sequences:** 1(1) **emPAI:** 0.05
NbS00010015g0012.1 protein AED:0.30 eAED:0.33 QI:231|0.66|0.76|0.92|0.75|0.69|13|305|569; (*SWP) sp|Q9ZR40|U2A2B_NICPL (e_value=0.0) Splicing factor U2af large subunit B OS=Nicotiana plumbaginifol

Query	Observed	Mr(expt)	Mr(calc)	ppm	Miss	Score	Expect	Rank	Unique	Peptide
12043	**580.8177**	**1159.6209**	**1159.6237**	**-2.38**	**0**	**39**	**0.0095**	**1**	**U**	**R.ELLESFGPLR.G**

Proteins matching the same set of peptides:
NbS00012053g0112.1 **Mass:** 52943 **Score:** 39 **Matches:** 1(1) **Sequences:** 1(1)
NbS00012053g0112.1 protein AED:0.21 eAED:0.23 QI:1389|0.68|0.58|1|0.81|0.82|17|0|487
NbS00014587g0003.1 **Mass:** 33660 **Score:** 39 **Matches:** 1(1) **Sequences:** 1(1)
NbS00014587g0003.1 protein AED:0.22 eAED:0.22 QI:49|1|1|1|1|1|6|259|311; (*GB) gi|75338884|sp|Q9ZR40.1|U2A2B_NICPL (e_value=0.0) RecName: Full=Splicing factor U2af large subunit B; AltName: Full=N
NbS00032951g0002.1 **Mass:** 57352 **Score:** 39 **Matches:** 1(1) **Sequences:** 1(1)
NbS00032951g0002.1 protein AED:0.18 eAED:0.18 QI:1156|0.76|0.78|0.92|0.84|0.78|14|0|527; (*GB) gi|75338884|sp|Q9ZR40.1|U2A2B_NICPL (e_value=0.0) RecName: Full=Splicing factor U2af large subunit B;

---

161. NbS00053814g0004.1 **Score:** 39 **Matches:** 1(1) **Sequences:** 1(1) **emPAI:** 0.06
NbS00053814g0004.1 protein AED:0.08 eAED:0.08 QI:0|0.71|0.75|0.87|0.85|0.75|8|0|460; (*GB) gi|359473220|ref|XP_003631270.1| (e_value=4e-107) PREDICTED: transcription factor bHLH90-like [Vitis vini

Query	Observed	Mr(expt)	Mr(calc)	ppm	Miss	Score	Expect	Rank	Unique	Peptide
9511	416.2498	830.4850	830.4749	12.2	0	39	0.0093	3	U	R.SSLIELIG.-

---

162. NbS00042606g0008.1 **Mass:** 62001 **Score:** 39 **Matches:** 1(1) **Sequences:** 1(1) **emPAI:** 0.05
NbS00042606g0008.1 protein AED:0.13 eAED:0.13 QI:139|1|0.75|1|0.66|0.75|4|0|562; (*GB) gi|359484596|ref|XP_002281155.2| (e_value=1e-144) PREDICTED: uncharacterized protein LOC100266187 [Vitis vini

Query	Observed	Mr(expt)	Mr(calc)	ppm	Miss	Score	Expect	Rank	Unique	Peptide
11783	**564.8291**	**1127.6437**	**1127.6438**	**-0.10**	**0**	**39**	**0.0064**	**1**	**U**	**K.IGDIIEELVK.S**

---

163. NbS00009748g0101.1 **Mass:** 67150 **Score:** 39 **Matches:** 2(1) **Sequences:** 2(1) **emPAI:** 0.10
NbS00009748g0101.1 protein AED:0.12 eAED:0.12 QI:0|-1|0|1|-1|1|1|0|600

Query	Observed	Mr(expt)	Mr(calc)	ppm	Miss	Score	Expect	Rank	Unique	Peptide
9733	**436.7362**	**871.4579**	**871.4552**	**3.14**	**0**	**30**	**0.069**	**1**	**U**	**K.TPAEWLR.N**
12936	**630.3415**	**1258.6684**	**1258.6704**	**-1.53**	**0**	**34**	**0.031**	**1**	**U**	**K.VTGNLIVCIDR.A**

Proteins matching the same set of peptides:
NbS00012890g0001.1 **Mass:** 60900 **Score:** 39 **Matches:** 2(1) **Sequences:** 2(1)
NbS00012890g0001.1 protein AED:0.20 eAED:0.21 QI:0|0|0|0.5|1|1|2|0|545; (*GB) gi|225440338|ref|XP_002270073.1| (e_value=0.0) PREDICTED: H/ACA ribonucleoprotein complex subunit 4 [Vitis vinifera];;

---

164. NbS00028472g0001.1 **Mass:** 55355 **Score:** 39 **Matches:** 1(1) **Sequences:** 1(1) **emPAI:** 0.06
NbS00028472g0001.1 protein AED:0.09 eAED:0.09 QI:0|0.5|0|1|1|1|3|0|497; (*GB) gi|147858961|emb|CAN80826.1| (e_value=0.0) hypothetical protein VITISV_015453 [Vitis vinifera];; (*SWP) sp|Q9DE27|RUVB

Query	Observed	Mr(expt)	Mr(calc)	ppm	Miss	Score	Expect	Rank	Unique	Peptide
11906	**572.3048**	**1142.5951**	**1142.5931**	**1.69**	**0**	**39**	**0.0094**	**1**	**U**	**R.GLGLDSSLEPR.L**

Proteins matching the same set of peptides:
NbS00059728g0105.1 **Mass:** 48062 **Score:** 39 **Matches:** 1(1) **Sequences:** 1(1)
NbS00059728g0105.1 protein AED:0.08 eAED:0.08 QI:0|0.5|0|1|0.5|0.33|3|0|432

---

165. NbS00001148g0004.1 **Mass:** 36834 **Score:** 39 **Matches:** 1(1) **Sequences:** 1(1) **emPAI:** 0.09
NbS00001148g0004.1 protein AED:0.25 eAED:0.26 QI:0|0.66|0.5|1|0|0|4|455|319; (*GB) gi|226506550|ref|NP_001141544.1| (e_value=2e-158) uncharacterized protein LOC100273658 [Zea mays];; (*SWP) sp|P18(

Query	Observed	Mr(expt)	Mr(calc)	ppm	Miss	Score	Expect	Rank	Unique	Peptide
11882	570.3505	1138.6864	1138.6862	0.11	0	39	0.0025	1	U	K.LAVNLIPFPR.L

Proteins matching the same set of peptides:
NbS00006464g0001.1 **Mass:** 42428 **Score:** 39 **Matches:** 1(1) **Sequences:** 1(1)
NbS00006464g0001.1 protein AED:0.20 eAED:0.29 QI:848|0.75|0.8|1|0.25|0.2|5|200|374; (*GB) gi|8928423|sp|Q9ZPN7.1|TBB4_ELEIN (e_value=0.0) RecName: Full=Tubulin beta-4 chain; AltName: Full=Beta-4-tu

166. NbS00006130g0035.1 **Mass:** 57611 **Score:** 38 **Matches:** 1(1) **Sequences:** 1(1) **emPAI:** 0.06
NbS00006130g0035.1 protein AED:0.02 eAED:0.02 QI:1363|1|1|1|0.75|0.4|5|684|545; (*GB) gi|555655|gb|AAA50196.1| (e_value=2e-123) DNA-binding protein [Nicotiana tabacum];; (*TAIR) AT3G18035.1 (e_valu

Query	Observed	Mr(expt)	Mr(calc)	ppm	Miss	Score	Expect	Rank	Unique	Peptide
15050	741.8477	1481.6809	1481.6780	1.93	0	38	0.0079	1	U	K.STTVDAAAGSSMGTAR.R

167. NbS00010382g0016.1 **Mass:** 30925 **Score:** 38 **Matches:** 1(1) **Sequences:** 1(1) **emPAI:** 0.11
NbS00010382g0016.1 protein AED:0.27 eAED:0.27 QI:0|1|0.5|1|1|1|2|0|280; (*GB) gi|357521321|ref|XP_003630949.1| (e_value=9e-153) 40S ribosomal protein S2 [Medicago truncatula];; (*SWP) sp|Q93VB8|RS2

Query	Observed	Mr(expt)	Mr(calc)	ppm	Miss	Score	Expect	Rank	Unique	Peptide
18244	930.4705	1858.9264	1858.9312	-2.62	1	38	0.0089	1	U	K.VIVYSTEEAPAPERVEA.-

Proteins matching the same set of peptides:
NbS00016839g0002.1 **Mass:** 32051 **Score:** 38 **Matches:** 1(1) **Sequences:** 1(1)
NbS00016839g0002.1 protein AED:0.03 eAED:0.03 QI:0|0.5|0|1|1|1|3|0|289; (*GB) gi|357521321|ref|XP_003630949.1| (e_value=3e-149) 40S ribosomal protein S2 [Medicago truncatula];; (*SWP) sp|Q93VB8|RS2

168. NbS00010129g0001.1 **Mass:** 35285 **Score:** 38 **Matches:** 1(1) **Sequences:** 1(1) **emPAI:** 0.09
NbS00010129g0001.1 protein AED:0.16 eAED:0.16 QI:0|0|0.5|1|1|1|2|131|318; (*SWP) sp|P23547|E13G_TOBAC (e_value=0.0) Glucan endo-1,3-beta-glucosidase, acidic isoform GI9 OS=Nicotiana tabacum GN=PR2

Query	Observed	Mr(expt)	Mr(calc)	ppm	Miss	Score	Expect	Rank	Unique	Peptide
14411	703.8947	1405.7748	1405.7718	2.12	0	38	0.0075	1	U	K.SFTNPIIQFLAR.H

Proteins matching the same set of peptides:
NbC25742659g0003.1 **Mass:** 37798 **Score:** 38 **Matches:** 1(1) **Sequences:** 1(1)
NbC25742659g0003.1 protein AED:0.11 eAED:0.11 QI:96|1|1|1|1|1|2|0|342; (*GB) gi|119012|sp|P23547.1|E13G_TOBAC (e_value=0.0) RecName: Full=Glucan endo-1,3-beta-glucosidase, acidic isoform GI9; AltNa

169. NbS00007190g0001.1 **Mass:** 30678 **Score:** 38 **Matches:** 1(1) **Sequences:** 1(1) **emPAI:** 0.11
NbS00007190g0001.1 protein AED:0.08 eAED:0.08 QI:179|1|1|1|0.5|0.33|3|443|275; (*GB) gi|225448932|ref|XP_002267178.1| (e_value=1e-93) PREDICTED: heterogeneous nuclear ribonucleoprotein G [Vitis vir

Query	Observed	Mr(expt)	Mr(calc)	ppm	Miss	Score	Expect	Rank	Unique	Peptide
8740	697.3700	1392.7254	1392.7249	0.39	0	38	0.023	1	U	R.IFVGGLSSDITER.Q

Proteins matching the same set of peptides:
NbS00029940g0009.1 **Mass:** 30714 **Score:** 38 **Matches:** 1(1) **Sequences:** 1(1)
NbS00029940g0009.1 protein AED:0.36 eAED:0.36 QI:205|1|1|1|0.5|0.66|3|523|275; (*GB) gi|225448932|ref|XP_002267178.1| (e_value=6e-80) PREDICTED: heterogeneous nuclear ribonucleoprotein G [Vitis vir

170. NbS00001041g0113.1 **Mass:** 49557 **Score:** 38 **Matches:** 1(1) **Sequences:** 1(1) **emPAI:** 0.07
NbS00001041g0113.1 protein AED:0.17 eAED:0.20 QI:0|1|0|1|1|1|2|0|462

Query	Observed	Mr(expt)	Mr(calc)	ppm	Miss	Score	Expect	Rank	Unique	Peptide
10880	507.8220	1013.6294	1013.6274	1.99	0	38	0.0051	1	U	R.AGGIFLPLVK.S

Proteins matching the same set of peptides:
NbS00014068g0001.1 **Mass:** 59919 **Score:** 38 **Matches:** 1(1) **Sequences:** 1(1)
NbS00014068g0001.1 protein AED:0.13 eAED:0.13 QI:0|1|0.33|1|1|1|3|0|561; (*GB) gi|255544892|ref|XP_002513507.1| (e_value=0.0) 2-oxoglutarate/malate translocator, chloroplast precursor, putative [R:

171. NbS00011116g0014.1 **Mass:** 23633 **Score:** 38 **Matches:** 1(1) **Sequences:** 1(1) **emPAI:** 0.14
NbS00011116g0014.1 protein AED:0.21 eAED:0.24 QI:14|0.5|0.4|1|0.75|0.8|5|1520|206; (*GB) gi|332038856|gb|ACX54788.2| (e_value=3e-47) putative arginine/serine-rich splicing factor [Arachis diogoi];;

Query	Observed	Mr(expt)	Mr(calc)	ppm	Miss	Score	Expect	Rank	Unique	Peptide
7508	516.7800	1031.5454	1031.5400	5.29	0	38	0.016	1	U	R.VYVGNLDPR.V

Proteins matching the same set of peptides:
NbS00014757g0107.1 **Mass:** 4268 **Score:** 38 **Matches:** 1(1) **Sequences:** 1(1)
NbS00014757g0107.1 protein AED:0.41 eAED:0.41 QI:0|0|0|0.5|0|0|2|0|35
NbS00016773g0019.1 **Mass:** 19793 **Score:** 38 **Matches:** 1(1) **Sequences:** 1(1)
NbS00016773g0019.1 protein AED:0.44 eAED:0.67 QI:0|0.25|0.4|0.8|1|1|5|0|170; (*GB) gi|359476412|ref|XP_002272657.2| (e_value=2e-31) PREDICTED: serine/arginine-rich splicing factor 7-like isoform 1
NbS00035033g0004.1 **Mass:** 24393 **Score:** 38 **Matches:** 1(1) **Sequences:** 1(1)
NbS00035033g0004.1 protein AED:0.33 eAED:0.33 QI:203|0.6|0.66|1|0.4|0.5|6|0|212; (*GB) gi|359476412|ref|XP_002272657.2| (e_value=3e-32) PREDICTED: serine/arginine-rich splicing factor 7-like isofo

---

172. NbS00008510g0009.1 **Mass:** 68372 **Score:** 37 **Matches:** 1(1) **Sequences:** 1(1) **emPAI:** 0.05
NbS00008510g0009.1 protein AED:0.00 eAED:0.00 QI:263|0.90|0.75|1|0.36|0.41|12|56|602; (*GB) gi|359476554|ref|XP_002267871.2| (e_value=0.0) PREDICTED: translational activator GCN1 isoform 1 [Vitis v

Query	Observed	Mr(expt)	Mr(calc)	ppm	Miss	Score	Expect	Rank	Unique	Peptide
13663	661.8604	1321.7063	1321.7030	2.45	0	37	0.012	1	U	K.GLSDAFIPLFSR.L

Proteins matching the same set of peptides:
NbS00014161g0001.1 **Mass:** 106962 **Score:** 37 **Matches:** 1(1) **Sequences:** 1(1)
NbS00014161g0001.1 protein AED:0.06 eAED:0.06 QI:717|0.88|0.94|0.94|0.83|0.89|19|10|950; (*GB) gi|296085156|emb|CBI28651.3| (e_value=0.0) unnamed protein product [Vitis vinifera];; (*SWP) sp|Q9261

---

173. NbS00016035g0004.1 **Mass:** 53453 **Score:** 37 **Matches:** 1(1) **Sequences:** 1(1) **emPAI:** 0.06
NbS00016035g0004.1 protein AED:0.29 eAED:0.29 QI:177|0.8|0.66|1|0.6|0.33|6|0|468; (*GB) gi|133711805|gb|ABO36623.1| (e_value=0.0) putative RNA-binding protein [Solanum lycopersicum];; (*SWP) sp|Q5S

Query	Observed	Mr(expt)	Mr(calc)	ppm	Miss	Score	Expect	Rank	Unique	Peptide
12327	595.2738	1188.5331	1188.5299	2.69	0	37	0.0087	1	U	R.AGIDYESSYGK.A

Proteins matching the same set of peptides:
NbS00016036g0005.1 **Mass:** 69810 **Score:** 37 **Matches:** 1(1) **Sequences:** 1(1)
NbS00016036g0005.1 protein AED:0.17 eAED:0.17 QI:177|1|0.8|1|0.75|0.6|5|0|619; (*GB) gi|133711805|gb|ABO36623.1| (e_value=0.0) putative RNA-binding protein [Solanum lycopersicum];; (*SWP) sp|Q5SNN
NbS00020983g0010.1 **Mass:** 54065 **Score:** 37 **Matches:** 1(1) **Sequences:** 1(1)
NbS00020983g0010.1 protein AED:0.12 eAED:0.12 QI:181|1|1|1|1|1|4|445|475; (*GB) gi|133711805|gb|ABO36623.1| (e_value=0.0) putative RNA-binding protein [Solanum lycopersicum];; (*SWP) sp|Q5SNN4|C3H

---

174. NbS00000215g0010.1 **Mass:** 64850 **Score:** 37 **Matches:** 1(1) **Sequences:** 1(1) **emPAI:** 0.05
NbS00000215g0010.1 protein AED:0.11 eAED:0.11 QI:211|1|1|1|0.85|0.8|15|450|605; (*GB) gi|225442531|ref|XP_002284134.1| (e_value=0.0) PREDICTED: ruBisCO large subunit-binding protein subunit beta, c

Query	Observed	Mr(expt)	Mr(calc)	ppm	Miss	Score	Expect	Rank	Unique	Peptide
13520	655.3821	1308.7497	1308.7514	-1.26	0	37	0.0064	1	U	K.VVAAGANPIQITR.G

Proteins matching the same set of peptides:
NbS00007027g0009.1 **Mass:** 64681 **Score:** 37 **Matches:** 1(1) **Sequences:** 1(1)
NbS00007027g0009.1 protein AED:0.13 eAED:0.13 QI:197|1|1|1|0.92|0.86|15|612|605; (*GB) gi|225442531|ref|XP_002284134.1| (e_value=0.0) PREDICTED: ruBisCO large subunit-binding protein subunit beta,

---

175. NbS00002703g0009.1 **Mass:** 49449 **Score:** 37 **Matches:** 1(1) **Sequences:** 1(1) **emPAI:** 0.07
NbS00002703g0009.1 protein AED:0.34 eAED:0.35 QI:0|0.8|0.66|1|1|0.83|6|0|443; (*GB) gi|77745479|gb|ABB02638.1| (e_value=0.0) 26S proteasome subunit 4-like [Solanum tuberosum];; (*SWP) sp|Q9SZD4|PRS

Query	Observed	Mr(expt)	Mr(calc)	ppm	Miss	Score	Expect	Rank	Unique	Peptide
12734	619.3292	1236.6439	1236.6462	-1.90	0	37	0.013	1	U	K.AVANSTSATFLR.V

Proteins matching the same set of peptides:
NbS00012174g0133.1 **Mass:** 53108 **Score:** 37 **Matches:** 1(1) **Sequences:** 1(1)
NbS00012174g0133.1 protein AED:0.32 eAED:0.35 QI:0|0.57|0.5|0.87|0.85|0.87|8|0|476
NbS00039466g0005.1 **Mass:** 50733 **Score:** 37 **Matches:** 1(1) **Sequences:** 1(1)
NbS00039466g0005.1 protein AED:0.18 eAED:0.18 QI:0|0.8|0.66|1|1|1|6|0|456; (*GB) gi|77745479|gb|ABB02638.1| (e_value=0.0) 26S proteasome subunit 4-like [Solanum tuberosum];; (*SWP) sp|Q9SZD4|PRS4A_

---

176. NbS00011278g0018.1 **Mass:** 14306 **Score:** 37 **Matches:** 1(1) **Sequences:** 1(1) **emPAI:** 0.24
NbS00011278g0018.1 protein AED:0.22 eAED:0.22 QI:213|1|1|1|0.75|1|5|391|127; (*GB) gi|255548958|ref|XP_002515535.1| (e_value=4e-64) conserved hypothetical protein [Ricinus communis];; (*SWP) sp|O22

Query	Observed	Mr(expt)	Mr(calc)	ppm	Miss	Score	Expect	Rank	Unique	Peptide
12554	609.3328	1216.6511	1216.6492	1.56	0	37	0.017	1	U	K.SLFFYVNLSK.R

Proteins matching the same set of peptides:
NbS00017711g0009.1 **Mass:** 14248 **Score:** 37 **Matches:** 1(1) **Sequences:** 1(1)
NbS00017711g0009.1 protein AED:0.20 eAED:0.20 QI:209|1|1|1|0.5|0.28|7|390|127; (*GB) gi|255548958|ref|XP_002515535.1| (e_value=1e-64) conserved hypothetical protein [Ricinus communis];; (*SWP) sp|(

---

177. NbS00004739g0001.1 **Mass:** 27728 **Score:** 37 **Matches:** 1(1) **Sequences:** 1(1) **emPAI:** 0.12
NbS00004739g0001.1 protein AED:0.17 eAED:0.17 QI:0|1|0.66|1|1|1|3|0|251; (*GB) gi|100801744|emb|CAK24966.1| (e_value=2e-175) chlorophyll a/b binding protein [Solanum tuberosum];; (*SWP) sp|Q9SQL2|(

Query	Observed	Mr(expt)	Mr(calc)	ppm	Miss	Score	Expect	Rank	Unique	Peptide
13580	658.3364	1314.6583	1314.6568	1.13	0	37	0.015	1	U	K.NPGSVNQDPIFK.N

Proteins matching the same set of peptides:
NbS00030208g0012.1 **Mass:** 25902 **Score:** 37 **Matches:** 1(1) **Sequences:** 1(1)
NbS00030208g0012.1 protein AED:0.05 eAED:0.05 QI:0|0.5|0|1|1|1|3|0|233; (*GB) gi|100801744|emb|CAK24966.1| (e_value=1e-158) chlorophyll a/b binding protein [Solanum tuberosum];; (*SWP) sp|Q9SQL2|CI
NbS00043913g0001.1 **Mass:** 27579 **Score:** 37 **Matches:** 1(1) **Sequences:** 1(1)
NbS00043913g0001.1 protein AED:0.36 eAED:0.36 QI:170|1|0.66|1|1|1|3|0|250; (*GB) gi|100801744|emb|CAK24966.1| (e_value=7e-166) chlorophyll a/b binding protein [Solanum tuberosum];; (*SWP) sp|P2752
NbS00052312g0004.1 **Mass:** 27591 **Score:** 37 **Matches:** 1(1) **Sequences:** 1(1)
NbS00052312g0004.1 protein AED:0.28 eAED:0.28 QI:166|1|0.66|1|0.5|0.66|3|0|250; (*SWP) sp|P27521|CA4_ARATH (e_value=1e-154) Chlorophyll a-b binding protein 4, chloroplastic OS=Arabidopsis thaliana

---

178. NbS00014198g0009.1 **Mass:** 88975 **Score:** 37 **Matches:** 1(1) **Sequences:** 1(1) **emPAI:** 0.04
NbS00014198g0009.1 protein AED:0.34 eAED:0.34 QI:244|0.83|0.73|1|0.88|0.78|19|0|804; (*GB) gi|225463508|ref|XP_002263911.1| (e_value=0.0) PREDICTED: uridine kinase-like protein 4 [Vitis vinifera];;

Query	Observed	Mr(expt)	Mr(calc)	ppm	Miss	Score	Expect	Rank	Unique	Peptide
14827	729.8826	1457.7506	1457.7474	2.20	0	37	0.015	1	U	R.LASINVENIESNR.Q

Proteins matching the same set of peptides:
NbS00058166g0004.1 **Mass:** 33122 **Score:** 37 **Matches:** 1(1) **Sequences:** 1(1)
NbS00058166g0004.1 protein AED:0.26 eAED:0.26 QI:0|0.5|0.33|1|1|0.66|3|0|307; (*GB) gi|255557204|ref|XP_002519633.1| (e_value=0.0) fructose-bisphosphate aldolase, putative [Ricinus communis];; (*SW

---

179. NbS00002201g0002.1 **Mass:** 51957 **Score:** 36 **Matches:** 1(1) **Sequences:** 1(1) **emPAI:** 0.06
NbS00002201g0002.1 protein AED:0.26 eAED:0.28 QI:0|0.9|0.72|1|1|1|11|0|453; (*GB) gi|356559428|ref|XP_003548001.1| (e_value=0.0) PREDICTED: V-type proton ATPase subunit H-like isoform 1 [Glycine ma

Query	Observed	Mr(expt)	Mr(calc)	ppm	Miss	Score	Expect	Rank	Unique	Peptide
14607	715.4013	1428.7881	1428.7824	3.95	0	36	0.014	1	U	R.VLITILDTSSDPR.T

---

180. NbS00017034g0014.1 **Mass:** 18705 **Score:** 36 **Matches:** 2(2) **Sequences:** 2(2) **emPAI:** 0.39
NbS00017034g0014.1 protein AED:0.20 eAED:0.20 QI:0|0.8|0.83|0.83|0.8|0.83|6|26|160; (*GB) gi|225454123|ref|XP_002269748.1| (e_value=9e-100) PREDICTED: cleavage and polyadenylation specificity facto

Query	Observed	Mr(expt)	Mr(calc)	ppm	Miss	Score	Expect	Rank	Unique	Peptide
15889	779.9327	1557.8508	1557.8515	-0.44	0	32	0.032	1	U	R.YGPVISTIPQQLSR.F
17917	895.4969	1788.9793	1788.9774	1.09	0	31	0.029	1	U	K.LLAVPLFELYDNVQR.Y

Proteins matching the same set of peptides:
NbS00018945g0009.1 **Mass:** 16278 **Score:** 36 **Matches:** 2(2) **Sequences:** 2(2)
NbS00018945g0009.1 protein AED:0.30 eAED:0.30 QI:161|0.5|0.8|1|0.5|0.4|5|27|139; (*GB) gi|315259982|gb|ADT92189.1| (e_value=7e-72) cleavage and polyadenylation specificity factor 5 [Zea mays];; (*S

---

181. NbS00028158g0015.1 **Mass:** 18033 **Score:** 36 **Matches:** 1(1) **Sequences:** 1(1) **emPAI:** 0.19
NbS00028158g0015.1 protein AED:0.44 eAED:0.54 QI:0|0.75|0.4|1|1|1|5|0|169; (*GB) gi|388500696|gb|AFK38414.1| (e_value=1e-63) unknown [Lotus japonicus];; (*SWP) sp|O04616|Y4115_ARATH (e_value=4e-59)

Query	Observed	Mr(expt)	Mr(calc)	ppm	Miss	Score	Expect	Rank	Unique	Peptide
12301	594.3087	1186.6028	1186.6081	-4.50	0	36	0.02	1	U	K.ELAEDIEQLK.K

---

182. NbS00005109g0003.1 **Mass:** 27168 **Score:** 36 **Matches:** 1(1) **Sequences:** 1(1) **emPAI:** 0.12
NbS00005109g0003.1 protein AED:0.14 eAED:0.14 QI:170|1|0.5|1|0|0.5|2|0|256; (*GB) gi|40949663|gb|AAR97545.1| (e_value=8e-149) germin-like protein [Nicotiana attenuata];; (*SWP) sp|Q94EG3|NEC1_NICLS

Query	Observed	Mr(expt)	Mr(calc)	ppm	Miss	Score	Expect	Rank	Unique	Peptide
8481	656.8900	1311.7654	1311.7511	11.0	0	36	0.0075	1	U	K.VPGLNTLGVSLSR.I

Proteins matching the same set of peptides:
NbS00014664g0013.1 **Mass:** 19059 **Score:** 36 **Matches:** 1(1) **Sequences:** 1(1)
NbS00014664g0013.1 protein AED:0.18 eAED:0.18 QI:0|0.33|0|0.75|0.66|0.75|4|0|180; (*GB) gi|40949663|gb|AAR97545.1| (e_value=2e-90) germin-like protein [Nicotiana attenuata];; (*SWP) sp|Q9SPV5|NEC1_

NbS00018956g0020.1 **Mass:** 21706 **Score:** 36 **Matches:** 1(1) **Sequences:** 1(1)
NbS00018956g0020.1 protein AED:0.42 eAED:0.44 QI:0|1|0|1|1|0|2|0|205; (*GB) gi|40949663|gb|AAR97545.1| (e_value=5e-123) germin-like protein [Nicotiana attenuata];; (*SWP) sp|Q9SPV5|NEC1_NICPL (e_va
NbS00050534g0006.1 **Mass:** 48065 **Score:** 36 **Matches:** 1(1) **Sequences:** 1(1)
NbS00050534g0006.1 protein AED:0.19 eAED:0.19 QI:63|0.33|0.25|1|0.33|0.25|4|0|456; (*SWP) sp|Q94EG3|NEC1_NICLS (e_value=3e-106) Nectarin-1 OS=Nicotiana langsdorffii x Nicotiana sanderae GN=NECI PE=

---

183. NbS00001671g0002.1 **Mass:** 17124 **Score:** 36 **Matches:** 1(1) **Sequences:** 1(1) **emPAI:** 0.20
NbS00001671g0002.1 protein ; (*GB) gi|77745458|gb|ABB02628.1| (e_value=3e-104) triose phosphate isomerase cytosolic isoform-like [Solanum tuberosum];; (*SWP) sp|P48495|TPIS_PETHY (e_value=4e-105) T

Query	Observed	Mr(expt)	Mr(calc)	ppm	Miss	Score	Expect	Rank	Unique	Peptide
14391	703.3648	1404.7151	1404.7096	3.86	0	36	0.019	1	U	R.ESGSTIDVVAAQTK.A

Proteins matching the same set of peptides:
NbS00011031g0007.1 **Mass:** 27347 **Score:** 36 **Matches:** 1(1) **Sequences:** 1(1)
NbS00011031g0007.1 protein AED:0.24 eAED:0.24 QI:143|1|1|1|0.87|0.77|9|710|254; (*GB) gi|77745458|gb|ABB02628.1| (e_value=2e-161) triose phosphate isomerase cytosolic isoform-like [Solanum tuberosu

---

184. NbS00019265g0001.1 **Mass:** 46832 **Score:** 36 **Matches:** 1(1) **Sequences:** 1(1) **emPAI:** 0.07
NbS00019265g0001.1 protein AED:0.29 eAED:0.34 QI:99|0.6|0.66|1|1|1|6|0|422; (*GB) gi|115765|sp|P10708.1|CB12_SOLLC (e_value=3e-151) RecName: Full=Chlorophyll a-b binding protein 7, chloroplastic; A

Query	Observed	Mr(expt)	Mr(calc)	ppm	Miss	Score	Expect	Rank	Unique	Peptide
20859	1347.1499	2692.2853	2692.2810	1.59	0	36	0.0095	1	U	K.LTGTDVGYPGGLWFDPLGWGSGSPEK.I

Proteins matching the same set of peptides:
NbS00036008g0006.1 **Mass:** 31337 **Score:** 36 **Matches:** 1(1) **Sequences:** 1(1)
NbS00036008g0006.1 protein AED:0.22 eAED:0.27 QI:311|0.75|0.6|1|0.75|0.6|5|0|291; (*GB) gi|115765|sp|P10708.1|CB12_SOLLC (e_value=4e-172) RecName: Full=Chlorophyll a-b binding protein 7, chloroplas
NbS00048043g0004.1 **Mass:** 32195 **Score:** 36 **Matches:** 1(1) **Sequences:** 1(1)
NbS00048043g0004.1 protein AED:0.22 eAED:0.28 QI:313|1|0.75|1|1|1|4|0|297; (*GB) gi|115765|sp|P10708.1|CB12_SOLLC (e_value=7e-158) RecName: Full=Chlorophyll a-b binding protein 7, chloroplastic; Al
NbS00053209g0004.1 **Mass:** 31908 **Score:** 36 **Matches:** 1(1) **Sequences:** 1(1)
NbS00053209g0004.1 protein AED:0.23 eAED:0.28 QI:319|1|0.5|1|1|1|4|0|295; (*GB) gi|115765|sp|P10708.1|CB12_SOLLC (e_value=2e-157) RecName: Full=Chlorophyll a-b binding protein 7, chloroplastic; Alt

---

185. NbS00005125g0015.1 **Mass:** 38864 **Score:** 36 **Matches:** 1(1) **Sequences:** 1(1) **emPAI:** 0.09
NbS00005125g0015.1 protein AED:0.27 eAED:0.27 QI:600|0.91|0.84|1|0.83|0.69|13|286|356; (*GB) gi|304368145|gb|ADM26718.1| (e_value=0.0) glycolate oxidase [Nicotiana benthamiana];; (*SWP) sp|P05414|G

Query	Observed	Mr(expt)	Mr(calc)	ppm	Miss	Score	Expect	Rank	Unique	Peptide
11207	528.8068	1055.5990	1055.5975	1.39	0	36	0.014	1	U	K.AIALTVDTPR.L

Proteins matching the same set of peptides:
NbS00024535g0016.1 **Mass:** 38256 **Score:** 36 **Matches:** 1(1) **Sequences:** 1(1)
NbS00024535g0016.1 protein AED:0.23 eAED:0.23 QI:299|1|1|1|0.81|0.75|12|251|349; (*GB) gi|304368145|gb|ADM26718.1| (e_value=0.0) glycolate oxidase [Nicotiana benthamiana];; (*SWP) sp|P05414|GOX_SPI
NbS00025736g0004.1 **Mass:** 38849 **Score:** 36 **Matches:** 1(1) **Sequences:** 1(1)
NbS00025736g0004.1 protein AED:0.28 eAED:0.28 QI:605|0.91|0.84|1|0.83|0.76|13|359|356; (*GB) gi|304368145|gb|ADM26718.1| (e_value=0.0) glycolate oxidase [Nicotiana benthamiana];; (*SWP) sp|P05414|G
NbS00043092g0003.1 **Mass:** 42275 **Score:** 36 **Matches:** 1(1) **Sequences:** 1(1)
NbS00043092g0003.1 protein AED:0.16 eAED:0.16 QI:995|0.81|0.75|1|0.90|0.83|12|0|384; (*GB) gi|304368145|gb|ADM26718.1| (e_value=0.0) glycolate oxidase [Nicotiana benthamiana];; (*SWP) sp|P05414|GOX
NbS00060838g0004.1 **Mass:** 40433 **Score:** 36 **Matches:** 1(1) **Sequences:** 1(1)
NbS00060838g0004.1 protein AED:0.12 eAED:0.12 QI:170|1|1|1|0.81|0.83|12|394|368; (*GB) gi|366984550|gb|AEX09184.1| (e_value=0.0) glycolate oxidase [Gossypium hirsutum];; (*SWP) sp|O49506|GLO5_ARATH

---

186. NbS00046932g0004.1 **Score:** 36 **Matches:** 1(1) **Sequences:** 1(1) **emPAI:** 0.10
NbS00046932g0004.1 protein AED:0.40 eAED:0.41 QI:0|0.8|0.5|1|0.6|0.66|6|0|302; (*GB) gi|225441674|ref|XP_002282707.1| (e_value=1e-174) PREDICTED: uncharacterized protein C4orf29 homolog [Vitis vin

Query	Observed	Mr(expt)	Mr(calc)	ppm	Miss	Score	Expect	Rank	Unique	Peptide
8744	349.2341	696.4536	696.4534	0.36	0	36	0.0028	2	U	R.LGGPLLK.E

---

187. NbS00000884g0011.1 **Mass:** 35229 **Score:** 36 **Matches:** 1(1) **Sequences:** 1(1) **emPAI:** 0.09
NbS00000884g0011.1 protein AED:0.32 eAED:0.32 QI:206|0.8|0.66|1|0.4|0.5|6|0|313; (*GB) gi|2500521|sp|Q40468.1|IF415_TOBAC (e_value=1e-151) RecName: Full=Eukaryotic initiation factor 4A-15; Short=eI

Query	Observed	Mr(expt)	Mr(calc)	ppm	Miss	Score	Expect	Rank	Unique	Peptide
15860	778.3623	1554.7101	1554.7058	2.74	0	36	0.012	1	U	K.MFVLDEADEMLSR.G

Proteins matching the same set of peptides:
NbS00004767g0011.1 **Mass:** 19305 **Score:** 36 **Matches:** 1(1) **Sequences:** 1(1)
NbS00004767g0011.1 protein AED:0.23 eAED:0.23 QI:0|0.5|0|1|0.5|0.33|3|0|165; (*GB) gi|386870481|gb|AFJ42571.1| (e_value=7e-64) elongation initiation factor 4A [Sesamum indicum];; (*SWP) sp|P35683|I

NbS00006310g0007.1 **Mass:** 36965 **Score:** 36 **Matches:** 1(1) **Sequences:** 1(1)
NbS00006310g0007.1 protein AED:0.13 eAED:0.13 QI:2|0.75|0.4|1|0.5|0.6|5|0|328; (*GB) gi|1170508|sp|P41381.1|IF4A8_TOBAC (e_value=5e-168) RecName: Full=Eukaryotic initiation factor 4A-8; Short=eIF-
NbS00007314g0014.1 **Mass:** 47532 **Score:** 36 **Matches:** 1(1) **Sequences:** 1(1)
NbS00007314g0014.1 protein AED:0.02 eAED:0.02 QI:0|0.66|0.25|1|1|1|4|0|416; (*GB) gi|1170508|sp|P41381.1|IF4A8_TOBAC (e_value=0.0) RecName: Full=Eukaryotic initiation factor 4A-8; Short=eIF-4A-8;
NbS00007954g0001.1 **Mass:** 39610 **Score:** 36 **Matches:** 1(1) **Sequences:** 1(1)
NbS00007954g0001.1 protein AED:0.25 eAED:0.25 QI:217|0.4|0.5|1|0.8|0.83|6|0|349; (*GB) gi|379067355|gb|AFC90091.1| (e_value=0.0) eukaryotic initiation factor 4A-14 [Nicotiana benthamiana];; (*SWP)
NbS00007954g0009.1 **Mass:** 34697 **Score:** 36 **Matches:** 1(1) **Sequences:** 1(1)
NbS00007954g0009.1 protein AED:0.32 eAED:0.32 QI:0|0.6|0.5|0.83|1|0.83|6|0|307; (*GB) gi|2500519|sp|Q40466.1|IF413_TOBAC (e_value=5e-155) RecName: Full=Eukaryotic initiation factor 4A-13; Short=eI
NbS00009080g0017.1 **Mass:** 48157 **Score:** 36 **Matches:** 1(1) **Sequences:** 1(1)
NbS00009080g0017.1 protein AED:0.38 eAED:0.38 QI:206|0.66|0.42|1|0.83|0.71|7|0|424; (*GB) gi|255560725|ref|XP_002521376.1| (e_value=9e-74) dead box ATP-dependent RNA helicase, putative [Ricinus co
NbS00021221g0007.1 **Mass:** 34363 **Score:** 36 **Matches:** 1(1) **Sequences:** 1(1)
NbS00021221g0007.1 protein AED:0.31 eAED:0.32 QI:161|0.75|0.6|1|0.5|0.4|5|0|305; (*GB) gi|2500519|sp|Q40466.1|IF413_TOBAC (e_value=7e-141) RecName: Full=Eukaryotic initiation factor 4A-13; Short=e
NbS00023364g0004.1 **Mass:** 34265 **Score:** 36 **Matches:** 1(1) **Sequences:** 1(1)
NbS00023364g0004.1 protein AED:0.30 eAED:0.30 QI:393|0.75|0.8|1|0.5|0.8|5|0|303; (*GB) gi|2500518|sp|Q40465.1|IF411_TOBAC (e_value=3e-143) RecName: Full=Eukaryotic initiation factor 4A-11; Short=e
NbS00025364g0011.1 **Mass:** 47086 **Score:** 36 **Matches:** 1(1) **Sequences:** 1(1)
NbS00025364g0011.1 protein AED:0.02 eAED:0.02 QI:10|1|1|1|1|1|4|286|413; (*GB) gi|2500517|sp|Q40471.1|IF4A9_TOBAC (e_value=0.0) RecName: Full=Eukaryotic initiation factor 4A-9; Short=eIF-4A-9; Alt
NbS00032509g0005.1 **Mass:** 28265 **Score:** 36 **Matches:** 1(1) **Sequences:** 1(1)
NbS00032509g0005.1 protein AED:0.17 eAED:0.17 QI:0|1|0|1|1|1|2|0|242; (*GB) gi|283049400|gb|ADB07168.1| (e_value=2e-156) DEAD-box RNA helicase-like protein [Prunus persica];; (*SWP) sp|P35683|IF4A
NbS00036362g0029.1 **Mass:** 35274 **Score:** 36 **Matches:** 1(1) **Sequences:** 1(1)
NbS00036362g0029.1 protein AED:0.33 eAED:0.33 QI:0|0.5|0.2|0.8|0.5|0.6|5|0|313; (*GB) gi|2500521|sp|Q40468.1|IF415_TOBAC (e_value=8e-153) RecName: Full=Eukaryotic initiation factor 4A-15; Short=eI
NbS00044851g0011.1 **Mass:** 47086 **Score:** 36 **Matches:** 1(1) **Sequences:** 1(1)
NbS00044851g0011.1 protein AED:0.16 eAED:0.16 QI:8|1|1|1|1|1|4|266|413; (*GB) gi|2500517|sp|Q40471.1|IF4A9_TOBAC (e_value=0.0) RecName: Full=Eukaryotic initiation factor 4A-9; Short=eIF-4A-9; AltN
NbS00048154g0003.1 **Mass:** 47160 **Score:** 36 **Matches:** 1(1) **Sequences:** 1(1)
NbS00048154g0003.1 protein AED:0.27 eAED:0.27 QI:218|1|0.8|1|0.75|0.8|5|0|413; (*GB) gi|379067355|gb|AFC90091.1| (e_value=0.0) eukaryotic initiation factor 4A-14 [Nicotiana benthamiana];; (*SWP) s
NbS00050065g0003.1 **Mass:** 61798 **Score:** 36 **Matches:** 1(1) **Sequences:** 1(1)
NbS00050065g0003.1 protein AED:0.07 eAED:0.07 QI:19|1|1|1|0.66|1|4|274|538; (*GB) gi|379054892|gb|AFC88837.1| (e_value=0.0) initiation factor 4A-3-like protein, partial [Miscanthus sinensis];; (*S
gi|90081060|dbj|BAE90010.1| **Mass:** 46613 **Score:** 36 **Matches:** 1(1) **Sequences:** 1(1)
gi|90081060|dbj|BAE90010.1| unnamed protein product [Macaca fascicularis]

---

188. NbS00024271g0003.1 **Mass:** 58558 **Score:** 36 **Matches:** 1(1) **Sequences:** 1(1) **emPAI:** 0.06
NbS00024271g0003.1 protein AED:0.06 eAED:0.06 QI:464|1|0.66|1|0|0.33|3|0|534; (*GB) gi|147882995|gb|ABQ51814.1| (e_value=0.0) RAN GTPase-activating protein 1 [Nicotiana benthamiana];; (*SWP) sp|Q9

Query	Observed	Mr(expt)	Mr(calc)	ppm	Miss	Score	Expect	Rank	Unique	Peptide
17271	845.4188	1688.8231	1688.8152	4.68	0	36	0.016	1	U	R.VGSEGGVALSQALGECR.N

Proteins matching the same set of peptides:
NbS00025668g0003.1 **Mass:** 58558 **Score:** 36 **Matches:** 1(1) **Sequences:** 1(1)
NbS00025668g0003.1 protein AED:0.11 eAED:0.11 QI:329|1|0.5|1|0|0.5|2|0|534; (*SWP) sp|Q9LE82|RAGP1_ARATH (e_value=0.0) RAN GTPase-activating protein 1 OS=Arabidopsis thaliana GN=RANGAP1 PE=1 SV=1;;
NbC24094968g0001.1 **Mass:** 10234 **Score:** 36 **Matches:** 1(1) **Sequences:** 1(1)
NbC24094968g0001.1 protein ; (*GB) gi|156229392|emb|CAM28208.1| (e_value=1e-56) Ran GTPase activating protein [Nicotiana benthamiana];; (*SWP) sp|Q9LE82|RAGP1_ARATH (e_value=5e-42) RAN GTPase-acti

---

189. NbS00009678g0004.1 **Mass:** 115817 **Score:** 36 **Matches:** 1(1) **Sequences:** 1(1) **emPAI:** 0.03
NbS00009678g0004.1 protein AED:0.24 eAED:0.23 QI:224|0.85|0.66|1|1|1|15|0|1042; (*GB) gi|359478503|ref|XP_002276796.2| (e_value=0.0) PREDICTED: protein TIC110, chloroplastic-like [Vitis vinifera];;

Query	Observed	Mr(expt)	Mr(calc)	ppm	Miss	Score	Expect	Rank	Unique	Peptide
16120	795.4041	1588.7937	1588.7944	-0.46	0	36	0.018	1	U	R.LAQAVTSGDLEAAESK.A

---

190. NbS00029754g0004.1 **Score:** 36 **Matches:** 1(1) **Sequences:** 1(1) **emPAI:** 0.05
NbS00029754g0004.1 protein AED:0.25 eAED:0.26 QI:246|0.4|0.36|1|0.9|0.81|11|0|636; (*GB) gi|357454461|ref|XP_003597511.1| (e_value=5e-50) hypothetical protein MTR_2g098830 [Medicago truncatula];;

Query	Observed	Mr(expt)	Mr(calc)	ppm	Miss	Score	Expect	Rank	Unique	Peptide
11229	530.8032	1059.5919	1059.5448	44.5	1	36	0.021	2	U	R.EDKAEELVK.S

---

191. NbS00027428g0011.1 **Mass:** 84144 **Score:** 35 **Matches:** 1(1) **Sequences:** 1(1) **emPAI:** 0.04
NbS00027428g0011.1 protein AED:0.20 eAED:0.20 QI:0|0|0|1|1|1|2|0|723; (*GB) gi|359475106|ref|XP_003631587.1| (e_value=0.0) PREDICTED: DEAD-box ATP-dependent RNA helicase 21-like [Vitis vinifera];;

Query	Observed	Mr(expt)	Mr(calc)	ppm	Miss	Score	Expect	Rank	Unique	Peptide
13117	639.3451	1276.6756	1276.6776	-1.50	0	35	0.021	1	U	R.YNVLVATDVAGR.G

Proteins matching the same set of peptides:
NbS00035002g0004.1 **Mass:** 83277 **Score:** 35 **Matches:** 1(1) **Sequences:** 1(1)
NbS00035002g0004.1 protein AED:0.32 eAED:0.32 QI:0|-1|0|1|-1|1|1|0|715; (*GB) gi|359475106|ref|XP_003631587.1| (e_value=0.0) PREDICTED: DEAD-box ATP-dependent RNA helicase 21-like [Vitis vinifera];
NbS00041320g0012.1 **Mass:** 37734 **Score:** 35 **Matches:** 1(1) **Sequences:** 1(1)
NbS00041320g0012.1 protein AED:0.17 eAED:0.19 QI:0|0|0|1|1|1|2|0|330; (*GB) gi|358347102|ref|XP_003637601.1| (e_value=2e-120) DEAD-box ATP-dependent RNA helicase [Medicago truncatula];; (*SWP) sp|I

---

192. NbS00002801g0002.1 **Score:** 35 **Matches:** 1(1) **Sequences:** 1(1) **emPAI:** 0.20
NbS00002801g0002.1 protein

Query	Observed	Mr(expt)	Mr(calc)	ppm	Miss	Score	Expect	Rank	Unique	Peptide
10590	487.2706	972.5266	972.5128	14.2	0	35	0.026	2	U	K.LELSTADPK.A

---

193. NbS00014581g0001.1 **Mass:** 42279 **Score:** 35 **Matches:** 2(2) **Sequences:** 2(2) **emPAI:** 0.16
NbS00014581g0001.1 protein AED:0.17 eAED:0.17 QI:214|0.83|0.71|1|0.83|0.71|7|0|390; (*GB) gi|4827251|dbj|BAA77604.1| (e_value=0.0) plastidic aldolase NPALDP1 [Nicotiana paniculata];; (*SWP) sp|Q94

Query	Observed	Mr(expt)	Mr(calc)	ppm	Miss	Score	Expect	Rank	Unique	Peptide
11438	543.7992	1085.5838	1085.5829	0.88	0	32	0.043	1	U	R.ANANSLAQLGK.Y
14240	694.3635	1386.7124	1386.7103	1.52	0	32	0.052	1	U	R.LASIGLENTEANR.Q

---

194. NbS00020253g0010.1 **Mass:** 71608 **Score:** 35 **Matches:** 1(1) **Sequences:** 1(1) **emPAI:** 0.05
NbS00020253g0010.1 protein AED:0.25 eAED:0.25 QI:0|0.85|0.5|1|0.85|0.75|8|0|669; (*GB) gi|296086337|emb|CBI31778.3| (e_value=0.0) unnamed protein product [Vitis vinifera];; (*SWP) sp|Q9LUW5|RH53_AI

Query	Observed	Mr(expt)	Mr(calc)	ppm	Miss	Score	Expect	Rank	Unique	Peptide
13839	671.9020	1341.7894	1341.7867	1.99	0	35	0.0095	1	U	K.LGISQEIISALAK.K

Proteins matching the same set of peptides:
NbS00027294g0010.1 **Mass:** 71059 **Score:** 35 **Matches:** 1(1) **Sequences:** 1(1)
NbS00027294g0010.1 protein AED:0.26 eAED:0.28 QI:0|1|0.71|1|1|0.85|7|0|666; (*GB) gi|296086337|emb|CBI31778.3| (e_value=0.0) unnamed protein product [Vitis vinifera];; (*SWP) sp|Q9LUW5|RH53_ARATH

---

195. NbS00000058g0018.1 **Mass:** 24519 **Score:** 35 **Matches:** 1(1) **Sequences:** 1(1) **emPAI:** 0.14
NbS00000058g0018.1 protein AED:0.02 eAED:0.02 QI:0|1|0|1|1|1|2|0|221; (*GB) gi|30013659|gb|AAP03872.1| (e_value=2e-130) putative photosystem I subunit III precursor [Nicotiana tabacum];; (*SWP) sp

Query	Observed	Mr(expt)	Mr(calc)	ppm	Miss	Score	Expect	Rank	Unique	Peptide
12605	612.8636	1223.7127	1223.7125	0.19	0	35	0.0079	1	U	K.EIIIDVPLANK.L

Proteins matching the same set of peptides:
NbS00009051g0007.1 **Mass:** 27636 **Score:** 35 **Matches:** 1(1) **Sequences:** 1(1)
NbS00009051g0007.1 protein AED:0.31 eAED:0.31 QI:0|0.5|0|1|1|1|3|0|249; (*GB) gi|30013659|gb|AAP03872.1| (e_value=1e-120) putative photosystem I subunit III precursor [Nicotiana tabacum];; (*SWP) s
NbS00035792g0016.1 **Mass:** 24757 **Score:** 35 **Matches:** 1(1) **Sequences:** 1(1)
NbS00035792g0016.1 protein AED:0.06 eAED:0.06 QI:0|0|0|1|1|1|2|0|224; (*GB) gi|30013659|gb|AAP03872.1| (e_value=3e-103) putative photosystem I subunit III precursor [Nicotiana tabacum];; (*SWP) sp
NbS00061093g0007.1 **Mass:** 22605 **Score:** 35 **Matches:** 1(1) **Sequences:** 1(1)
NbS00061093g0007.1 protein AED:0.06 eAED:0.06 QI:0|0|0|1|0|0.5|2|0|206; (*GB) gi|30013659|gb|AAP03872.1| (e_value=3e-90) putative photosystem I subunit III precursor [Nicotiana tabacum];; (*SWP) sp

---

196. NbS00029951g0016.1 **Mass:** 119791 **Score:** 35 **Matches:** 1(1) **Sequences:** 1(1) **emPAI:** 0.03
NbS00029951g0016.1 protein AED:0.12 eAED:0.12 QI:0|1|0.72|1|0.7|0.72|11|0|1065; (*GB) gi|297733757|emb|CBI15004.3| (e_value=0.0) unnamed protein product [Vitis vinifera];; (*TAIR) AT3G48200.1 (e_va

Query	Observed	Mr(expt)	Mr(calc)	ppm	Miss	Score	Expect	Rank	Unique	Peptide
14225	462.5931	1384.7575	1384.7463	8.10	1	35	0.017	1	U	R.KLESGFALLDHR.L

---

197. NbS00013165g0006.1 **Mass:** 110128 **Score:** 35 **Matches:** 1(1) **Sequences:** 1(1) **emPAI:** 0.03
NbS00013165g0006.1 protein AED:0.11 eAED:0.11 QI:247|1|1|1|0.66|0.7|10|613|960; (*GB) gi|359496309|ref|XP_002264751.2| (e_value=0.0) PREDICTED: E3 ubiquitin-protein ligase HOS1-like [Vitis vinifera

Query	Observed	Mr(expt)	Mr(calc)	ppm	Miss	Score	Expect	Rank	Unique	Peptide
10912	509.3078	1016.6011	1016.6019	-0.73	0	35	0.012	1	U	R.AAVDILFLR.G

Proteins matching the same set of peptides:
NbS00020121g0006.1 **Mass:** 112853 **Score:** 35 **Matches:** 1(1) **Sequences:** 1(1)
NbS00020121g0006.1 protein AED:0.03 eAED:0.03 QI:260|0.9|0.81|1|0.5|0.45|11|409|982; (*GB) gi|359496309|ref|XP_002264751.2| (e_value=0.0) PREDICTED: E3 ubiquitin-protein ligase HOS1-like [Vitis vir

198. NbS00016832g0010.1 **Mass:** 101765 **Score:** 35 **Matches:** 1(1) **Sequences:** 1(1) **emPAI:** 0.03
NbS00016832g0010.1 protein AED:0.10 eAED:0.11 QI:0|0.5|0.33|1|0.5|0.33|3|0|902; (*GB) gi|255544686|ref|XP_002513404.1| (e_value=0.0) eukaryotic translation elongation factor, putative [Ricinus comm

Query	Observed	Mr(expt)	Mr(calc)	ppm	Miss	Score	Expect	Rank	Unique	Peptide
19982	753.0720	2256.1942	2256.1961	-0.84	0	35	0.012	1	U	K.STLTDSLVAAAGIIAQEVAGDVR.M

Proteins matching the same set of peptides:
NbS00030487g0018.1 **Mass:** 98682 **Score:** 35 **Matches:** 1(1) **Sequences:** 1(1)
NbS00030487g0018.1 protein AED:0.08 eAED:0.11 QI:0|1|0.5|1|1|1|2|0|876; (*GB) gi|388540218|gb|AFK64817.1| (e_value=0.0) translation elongation factor 2 [Prunus persica];; (*SWP) sp|O23755|EF2_BETVU
NbS00034885g0004.1 **Mass:** 87594 **Score:** 35 **Matches:** 1(1) **Sequences:** 1(1)
NbS00034885g0004.1 protein AED:0.02 eAED:0.02 QI:0|0.33|0.25|0.75|0.66|0.5|4|0|779; (*GB) gi|388540218|gb|AFK64817.1| (e_value=0.0) translation elongation factor 2 [Prunus persica];; (*SWP) sp|O23

199. NbS00017365g0003.1 **Mass:** 53904 **Score:** 35 **Matches:** 1(1) **Sequences:** 1(1) **emPAI:** 0.06
NbS00017365g0003.1 protein AED:0.22 eAED:0.22 QI:0|1|0.6|1|1|1|5|0|481; (*GB) gi|255561431|ref|XP_002521726.1| (e_value=0.0) srpk, putative [Ricinus communis];; (*SWP) sp|P36616|DSK1_SCHPO (e_value

Query	Observed	Mr(expt)	Mr(calc)	ppm	Miss	Score	Expect	Rank	Unique	Peptide
4401	402.2100	1203.6082	1203.5778	25.2	1	35	0.058	1	U	R.ASMGGAGGGAKANR.S

200. NbS00034416g0005.1 **Mass:** 43834 **Score:** 35 **Matches:** 2(1) **Sequences:** 1(1) **emPAI:** 0.08
NbS00034416g0005.1 protein AED:0.15 eAED:0.48 QI:0|0|0|1|0|0|5|0|377; (*GB) gi|312370165|gb|ADQ74440.1| (e_value=5e-71) ribulose-1,5-bisphosphate carboxylase/oxygenase large subunit [Physochlaina p

Query	Observed	Mr(expt)	Mr(calc)	ppm	Miss	Score	Expect	Rank	Unique	Peptide
12311	396.5598	1186.6575	1186.6571	0.38	0	31	0.05	1	U	R.DNGLLLHIHR.E 12310

201. NbS00049664g0004.1 **Mass:** 25462 **Score:** 35 **Matches:** 1(1) **Sequences:** 1(1) **emPAI:** 0.13
NbS00049664g0004.1 protein AED:0.29 eAED:0.29 QI:0|0.6|0.33|0.66|1|0.83|6|0|236; (*GB) gi|584795|sp|Q08436.1|PMA3_NICPL (e_value=1e-107) RecName: Full=Plasma membrane ATPase 3; AltName: Full=Proton

Query	Observed	Mr(expt)	Mr(calc)	ppm	Miss	Score	Expect	Rank	Unique	Peptide
15886	779.9205	1557.8264	1557.8250	0.89	0	35	0.02	1	U	K.IDQSALTGESLPVTK.G

202. NbS00040143g0002.1 **Mass:** 35938 **Score:** 34 **Matches:** 1(1) **Sequences:** 1(1) **emPAI:** 0.09
NbS00040143g0002.1 protein AED:0.18 eAED:0.18 QI:0|0|0|0.75|0|0.25|4|0|319; (*GB) gi|11465942|ref|NP_054484.1| (e_value=1e-124) ATP synthase CF0 A subunit [Nicotiana tabacum];; (*SWP) sp|P69372|ATP

Query	Observed	Mr(expt)	Mr(calc)	ppm	Miss	Score	Expect	Rank	Unique	Peptide
17702	876.9246	1751.8347	1751.8301	2.62	0	34	0.02	1	U	K.WLGGMLTNWSTTETR.L

203. NbS00014198g0003.1 **Mass:** 12038 **Score:** 34 **Matches:** 1(1) **Sequences:** 1(1) **emPAI:** 0.29
NbS00014198g0003.1 protein

Query	Observed	Mr(expt)	Mr(calc)	ppm	Miss	Score	Expect	Rank	Unique	Peptide
11019	516.7620	1031.5095	1031.4818	26.8	0	34	0.03	1	U	R.SSHANITMR.R

204. NbS00026008g0003.1 **Mass:** 41957 **Score:** 34 **Matches:** 1(1) **Sequences:** 1(1) **emPAI:** 0.08
NbS00026008g0003.1 protein AED:0.26 eAED:0.26 QI:130|1|1|1|1|1|6|514|394; (*GB) gi|164459314|gb|ABY57764.1| (e_value=0.0) extracellular Ca2+ sensing receptor [Nicotiana tabacum];; (*SWP) sp|Q9FN48

Query	Observed	Mr(expt)	Mr(calc)	ppm	Miss	Score	Expect	Rank	Unique	Peptide
16090	793.8713	1585.7281	1585.7294	-0.79	0	34	0.016	1	U	K.GSNIVIMDSYSDSAK.T

205. NbS00003479g0020.1 **Mass:** 40228 **Score:** 34 **Matches:** 1(1) **Sequences:** 1(1) **emPAI:** 0.08
NbS00003479g0020.1 protein AED:0.28 eAED:0.28 QI:190|1|1|1|0.75|0.6|5|184|363; (*GB) gi|396589|emb|CAA52461.1| (e_value=0.0) catechol O-methyltransferase [Nicotiana tabacum];; (*SWP) sp|Q9FQY8|COMT

Query	Observed	Mr(expt)	Mr(calc)	ppm	Miss	Score	Expect	Rank	Unique	Peptide
14736	723.3981	1444.7817	1444.7847	-2.05	0	34	0.023	1	U	K.SALELDLLELMAK.A

Proteins matching the same set of peptides:
NbS00045109g0006.1 **Mass:** 40090 **Score:** 34 **Matches:** 1(1) **Sequences:** 1(1)
NbS00045109g0006.1 protein AED:0.02 eAED:0.02 QI:182|1|1|1|1|1|4|383|363; (*GB) gi|396589|emb|CAA52461.1| (e_value=0.0) catechol O-methyltransferase [Nicotiana tabacum];; (*SWP) sp|Q9FQY8|COMT1_CAP

206. NbS00022724g0010.1 **Mass:** 15603 **Score:** 34 **Matches:** 1(1) **Sequences:** 1(1) **emPAI:** 0.22

NbS00022724g0010.1 protein AED:0.41 eAED:0.41 QI:0|0.83|0.71|1|0.66|0.71|7|141|139; (*GB) gi|363808256|ref|NP_001242493.1| (e_value=4e-92) uncharacterized protein LOC100807064 [Glycine max];; (*SWI

Query	Observed	Mr(expt)	Mr(calc)	ppm	Miss	Score	Expect	Rank	Unique	Peptide
13093	638.3398	1274.6650	1274.6619	2.43	0	34	0.03	1	U	R.AGEIVVFNVDGR.E

Proteins matching the same set of peptides:
NbS00023720g0013.1 **Mass:** 17607 **Score:** 34 **Matches:** 1(1) **Sequences:** 1(1)
NbS00023720g0013.1 protein AED:0.41 eAED:0.41 QI:3|0.71|0.75|0.87|0.57|0.62|8|142|156; (*GB) gi|224070829|ref|XP_002303254.1| (e_value=2e-92) predicted protein [Populus trichocarpa];; (*SWP) sp|Q9W

207. NbS00033933g0011.1 **Mass:** 21166 **Score:** 34 **Matches:** 1(1) **Sequences:** 1(1) **emPAI:** 0.16
NbS00033933g0011.1 protein AED:0.39 eAED:0.39 QI:0|0|0|1|0|0|3|0|181; (*GB) gi|113205233|gb|AAT39304.2| (e_value=1e-15) Transposon MuDR mudrA-like protein, putative [Solanum demissum];; (*ITAG) Sol

Query	Observed	Mr(expt)	Mr(calc)	ppm	Miss	Score	Expect	Rank	Unique	Peptide
9069	380.2345	758.4544	758.4174	48.8	0	34	0.04	1	U	K.DALAIEK.K

208. NbS00012538g0018.1 **Mass:** 28682 **Score:** 34 **Matches:** 1(1) **Sequences:** 1(1) **emPAI:** 0.12
NbS00012538g0018.1 protein AED:0.32 eAED:0.32 QI:0|0|0|1|0|0|5|0|257; (*GB) gi|357470629|ref|XP_003605599.1| (e_value=9e-89) Apocytochrome f [Medicago truncatula];; (*SWP) sp|Q8S8W4|CYF_ATRBE (e_va

Query	Observed	Mr(expt)	Mr(calc)	ppm	Miss	Score	Expect	Rank	Unique	Peptide
10836	504.2652	1006.5158	1006.5157	0.11	0	34	0.038	1	U	R.IPYDMQLK.R

Proteins matching the same set of peptides:
NbS00013560g0008.1 **Mass:** 31705 **Score:** 34 **Matches:** 1(1) **Sequences:** 1(1)
NbS00013560g0008.1 protein AED:0.29 eAED:0.35 QI:0|0|0|1|0|0|5|0|281; (*SWP) sp|P02376|RR19_TOBAC (e_value=9e-41) 30S ribosomal protein S19, chloroplastic OS=Nicotiana tabacum GN=rps19 PE=3 SV=3;;
NbS00038749g0001.1 **Mass:** 7885 **Score:** 34 **Matches:** 1(1) **Sequences:** 1(1)
NbS00038749g0001.1 protein ; (*GB) gi|89280648|ref|YP_514865.1| (e_value=1e-29) cytochrome f [Solanum lycopersicum];; (*SWP) sp|Q2VEG4|CYF_SOLTU (e_value=4e-31) Apocytochrome f OS=Solanum tuberosum

209. NbS00006841g0003.1 **Mass:** 31001 **Score:** 34 **Matches:** 1(1) **Sequences:** 1(1) **emPAI:** 0.11
NbS00006841g0003.1 protein AED:0.24 eAED:0.24 QI:284|1|1|1|0.66|0.75|4|120|287; (*GB) gi|115291793|gb|ABI93215.1| (e_value=0.0) water channel protein [Nicotiana tabacum];; (*SWP) sp|Q08451|PIP1_SOI

Query	Observed	Mr(expt)	Mr(calc)	ppm	Miss	Score	Expect	Rank	Unique	Peptide
14029	680.8649	1359.7153	1359.7147	0.47	0	34	0.03	1	U	R.LGGGANIVNPGYTK.G

210. NbS00001471g0009.1 **Mass:** 66057 **Score:** 34 **Matches:** 1(1) **Sequences:** 1(1) **emPAI:** 0.05
NbS00001471g0009.1 protein AED:0.21 eAED:0.22 QI:0|0.87|0.77|1|1|1|9|428|607; (*GB) gi|356555871|ref|XP_003546253.1| (e_value=0.0) PREDICTED: lysosomal beta glucosidase-like [Glycine max];; (*SWP)

Query	Observed	Mr(expt)	Mr(calc)	ppm	Miss	Score	Expect	Rank	Unique	Peptide
10777	500.7928	999.5710	999.5713	-0.25	0	34	0.026	1	U	R.IGAATALEVR.A

Proteins matching the same set of peptides:
NbS00011140g0006.1 **Mass:** 76355 **Score:** 34 **Matches:** 1(1) **Sequences:** 1(1)
NbS00011140g0006.1 protein AED:0.16 eAED:0.17 QI:0|0.75|0.44|1|1|1|9|0|696; (*GB) gi|3582436|dbj|BAA33065.1| (e_value=0.0) beta-D-glucan exohydrolase [Nicotiana tabacum];; (*SWP) sp|Q23892|GLUA_DIC
NbS00011492g0006.1 **Mass:** 64535 **Score:** 34 **Matches:** 1(1) **Sequences:** 1(1)
NbS00011492g0006.1 protein AED:0.08 eAED:0.09 QI:0|0|0|1|1|1|2|0|589; (*GB) gi|3582436|dbj|BAA33065.1| (e_value=0.0) beta-D-glucan exohydrolase [Nicotiana tabacum];; (*SWP) sp|Q23892|GLUA_DICDI (e_
NbS00012136g0003.1 **Mass:** 68565 **Score:** 34 **Matches:** 1(1) **Sequences:** 1(1)
NbS00012136g0003.1 protein AED:0.20 eAED:0.20 QI:210|1|1|1|1|1|9|248|627; (*GB) gi|3582436|dbj|BAA33065.1| (e_value=0.0) beta-D-glucan exohydrolase [Nicotiana tabacum];; (*SWP) sp|Q23892|GLUA_DICDI
NbS00014718g0006.1 **Mass:** 77426 **Score:** 34 **Matches:** 1(1) **Sequences:** 1(1)
NbS00014718g0006.1 protein AED:0.13 eAED:0.13 QI:0|0.55|0.5|1|0.55|0.4|10|0|705; (*GB) gi|3582436|dbj|BAA33065.1| (e_value=0.0) beta-D-glucan exohydrolase [Nicotiana tabacum];; (*SWP) sp|Q23892|GLU
NbS00015529g0004.1 **Mass:** 72833 **Score:** 34 **Matches:** 1(1) **Sequences:** 1(1)
NbS00015529g0004.1 protein AED:0.05 eAED:0.05 QI:316|0.66|0.8|1|1|1|10|257|665; (*GB) gi|225436114|ref|XP_002278363.1| (e_value=0.0) PREDICTED: lysosomal beta glucosidase-like isoform 1 [Vitis vin
NbS00024745g0008.1 **Mass:** 79490 **Score:** 34 **Matches:** 1(1) **Sequences:** 1(1)
NbS00024745g0008.1 protein AED:0.11 eAED:0.11 QI:182|0.77|0.7|1|1|1|10|339|722; (*GB) gi|356533037|ref|XP_003535075.1| (e_value=0.0) PREDICTED: lysosomal beta glucosidase-like [Glycine max];; (*SWI
NbS00033441g0004.1 **Mass:** 84959 **Score:** 34 **Matches:** 1(1) **Sequences:** 1(1)
NbS00033441g0004.1 protein AED:0.22 eAED:0.23 QI:0|0.63|0.5|0.91|0.90|0.83|12|0|754; (*GB) gi|255565893|ref|XP_002523935.1| (e_value=0.0) hydrolase, hydrolyzing O-glycosyl compounds, putative [Ric
NbS00058677g0004.1 **Mass:** 70975 **Score:** 34 **Matches:** 1(1) **Sequences:** 1(1)
NbS00058677g0004.1 protein AED:0.06 eAED:0.06 QI:138|0.88|0.9|1|0.77|0.8|10|177|645; (*GB) gi|3582436|dbj|BAA33065.1| (e_value=0.0) beta-D-glucan exohydrolase [Nicotiana tabacum];; (*SWP) sp|Q2389
NbC25936038g0001.1 **Mass:** 18915 **Score:** 34 **Matches:** 1(1) **Sequences:** 1(1)
NbC25936038g0001.1 protein ; (*GB) gi|255565897|ref|XP_002523937.1| (e_value=8e-76) hydrolase, hydrolyzing O-glycosyl compounds, putative [Ricinus communis];; (*TAIR) AT5G04885.1 (e_value=6e-68) |
NbC26143923g0005.1 **Mass:** 60960 **Score:** 34 **Matches:** 1(1) **Sequences:** 1(1)

NbC26143923g0005.1 protein AED:0.16 eAED:0.19 QI:0|0.77|0.7|1|0.77|0.8|10|555|557; (*GB) gi|3582436|dbj|BAA33065.1| (e_value=0.0) beta-D-glucan exohydrolase [Nicotiana tabacum];; (*SWP) sp|Q56078|I

---

211. NbS00021709g0005.1 **Mass:** 46410 **Score:** 34 **Matches:** 1(1) **Sequences:** 1(1) **emPAI:** 0.07
NbS00021709g0005.1 protein AED:0.27 eAED:0.27 QI:208|0.75|0.88|1|0.5|0.44|9|304|415; (*GB) gi|225435464|ref|XP_002285466.1| (e_value=0.0) PREDICTED: aldehyde dehydrogenase family 3 member F1 [Vitis

Query	Observed	Mr(expt)	Mr(calc)	ppm	Miss	Score	Expect	Rank	Unique	Peptide
10577	486.2854	970.5563	970.5560	0.37	0	34	0.026	1	U	R.SQLQNLLR.L

---

212. NbS00054324g0011.1 **Mass:** 170142 **Score:** 34 **Matches:** 1(1) **Sequences:** 1(1) **emPAI:** 0.02
NbS00054324g0011.1 protein AED:0.09 eAED:0.09 QI:199|0.88|0.81|1|0.65|0.59|27|513|1505; (*GB) gi|296087564|emb|CBI34153.3| (e_value=0.0) unnamed protein product [Vitis vinifera];; (*SWP) sp|Q9Z0W3

Query	Observed	Mr(expt)	Mr(calc)	ppm	Miss	Score	Expect	Rank	Unique	Peptide
13989	679.3690	1356.7234	1356.7249	-1.11	0	34	0.027	1	U	R.TPNISSEEVILR.L

---

213. NbS00018164g0006.1 **Mass:** 34002 **Score:** 34 **Matches:** 1(1) **Sequences:** 1(1) **emPAI:** 0.10
NbS00018164g0006.1 protein ; (*GB) gi|297738947|emb|CBI28192.3| (e_value=5e-127) unnamed protein product [Vitis vinifera];; (*TAIR) AT5G51200.1 (e_value=6e-115) | Symbols: | Protein of unknown func

Query	Observed	Mr(expt)	Mr(calc)	ppm	Miss	Score	Expect	Rank	Unique	Peptide
18714	969.0182	1936.0218	1936.0266	-2.45	0	34	0.022	1	U	R.AVVLDQGLEPDLVADVQR.F

Proteins matching the same set of peptides:
NbS00006888g0007.1 **Mass:** 202732 **Score:** 34 **Matches:** 1(1) **Sequences:** 1(1)
NbS00006888g0007.1 protein AED:0.26 eAED:0.27 QI:36|0.83|0.86|0.97|0.88|0.93|44|185|1777; (*SWP) sp|Q92621|NU205_HUMAN (e_value=3e-37) Nuclear pore complex protein Nup205 OS=Homo sapiens GN=NUP205

---

214. NbS00012577g0009.1 **Mass:** 84751 **Score:** 34 **Matches:** 1(1) **Sequences:** 1(1) **emPAI:** 0.04
NbS00012577g0009.1 protein AED:0.13 eAED:0.13 QI:321|1|1|1|0.9|0.81|11|196|765; (*SWP) sp|Q42699|METE_CATRO (e_value=0.0) 5-methyltetrahydropteroyltriglutamate--homocysteine methyltransferase OS=Ca

Query	Observed	Mr(expt)	Mr(calc)	ppm	Miss	Score	Expect	Rank	Unique	Peptide
14806	728.4150	1454.8154	1454.8093	4.22	0	34	0.016	1	U	K.AGISVIQIDEAALR.E

Proteins matching the same set of peptides:
NbS00044592g0017.1 **Mass:** 88066 **Score:** 34 **Matches:** 1(1) **Sequences:** 1(1)
NbS00044592g0017.1 protein AED:0.22 eAED:0.22 QI:367|0.90|0.83|1|0.81|0.83|12|0|795; (*GB) gi|8439545|gb|AAF74983.1|AF082893_1 (e_value=0.0) methionine synthase [Solanum tuberosum];; (*SWP) sp|Q42(

---

215. NbS00019818g0001.1 **Mass:** 35404 **Score:** 34 **Matches:** 1(1) **Sequences:** 1(1) **emPAI:** 0.09
NbS00019818g0001.1 protein AED:0.28 eAED:0.28 QI:0|1|0.5|1|1|1|2|0|332; (*GB) gi|384038815|gb|AFH57998.1| (e_value=0.0) chloroplast PsbO2 precursor [Nicotiana benthamiana];; (*SWP) sp|Q40459|PSBO_

Query	Observed	Mr(expt)	Mr(calc)	ppm	Miss	Score	Expect	Rank	Unique	Peptide
11392	540.7825	1079.5504	1079.5499	0.47	0	34	0.035	1	U	R.LTFDEIQSK.T

Proteins matching the same set of peptides:
NbS00032545g0003.1 **Mass:** 35462 **Score:** 34 **Matches:** 1(1) **Sequences:** 1(1)
NbS00032545g0003.1 protein AED:0.18 eAED:0.18 QI:0|1|0.5|1|1|1|2|0|332; (*GB) gi|384038813|gb|AFH57997.1| (e_value=0.0) chloroplast PsbO1 precursor [Nicotiana benthamiana];; (*SWP) sp|Q40459|PSBO_

---

216. NbS00002113g0001.1 **Mass:** 43922 **Score:** 33 **Matches:** 1(1) **Sequences:** 1(1) **emPAI:** 0.08
NbS00002113g0001.1 protein AED:0.00 eAED:0.05 QI:0|-1|0|1|-1|1|1|0|394; (*SWP) sp|Q00874|DR100_ARATH (e_value=1e-127) DNA-damage-repair/toleration protein DRT100 OS=Arabidopsis thaliana GN=DRT100 I

Query	Observed	Mr(expt)	Mr(calc)	ppm	Miss	Score	Expect	Rank	Unique	Peptide
11675	559.3081	1116.6017	1116.6026	-0.87	0	33	0.04	1	U	R.LSDLDLSLNK.L

---

217. NbS00016451g0014.1 **Mass:** 113558 **Score:** 33 **Matches:** 1(1) **Sequences:** 1(1) **emPAI:** 0.03
NbS00016451g0014.1 protein AED:0.11 eAED:0.11 QI:268|0.94|0.88|1|0.88|0.83|18|330|1024; (*GB) gi|359487657|ref|XP_002278468.2| (e_value=0.0) PREDICTED: kinesin-4-like [Vitis vinifera];; (*SWP) sp|(

Query	Observed	Mr(expt)	Mr(calc)	ppm	Miss	Score	Expect	Rank	Unique	Peptide
11675	559.3081	1116.6017	1116.6139	-10.93	1	33	0.04	1	U	R.LSDIDLASRK.A

Proteins matching the same set of peptides:
NbS00037039g0001.1 **Mass:** 70923 **Score:** 33 **Matches:** 1(1) **Sequences:** 1(1)
NbS00037039g0001.1 protein AED:0.20 eAED:0.20 QI:0|0.75|0.61|0.92|1|1|13|0|641; (*GB) gi|359479934|ref|XP_002269237.2| (e_value=0.0) PREDICTED: kinesin-4-like [Vitis vinifera];; (*SWP) sp|O81635|A!

---

218. NbS00002808g0033.1 **Mass:** 62817 **Score:** 33 **Matches:** 1(1) **Sequences:** 1(1) **emPAI:** 0.05
NbS00002808g0033.1 protein AED:0.14 eAED:0.14 QI:0|1|0.94|1|0.88|0.94|18|425|576; (*GB) gi|225433255|ref|XP_002285452.1| (e_value=0.0) PREDICTED: U-box domain-containing protein 72 [Vitis vinifera]

Query	Observed	Mr(expt)	Mr(calc)	ppm	Miss	Score	Expect	Rank	Unique	Peptide
9775	439.2614	876.5083	876.5069	1.56	0	33	0.027	1	U	R.VFQVASVK.A

Proteins matching the same set of peptides:
NbS00015167g0014.1 **Mass:** 62356 **Score:** 33 **Matches:** 1(1) **Sequences:** 1(1)
NbS00015167g0014.1 protein AED:0.27 eAED:0.27 QI:0|1|1|1|0.88|0.88|18|478|568; (*SWP) sp|Q94BR4|PR19A_ARATH (e_value=0.0) Pre-mRNA-processing factor 19 homolog 1 OS=Arabidopsis thaliana GN=PRP19A I

219. NbS00006379g0022.1 **Mass:** 15288 **Score:** 33 **Matches:** 1(1) **Sequences:** 1(1) **emPAI:** 0.22
NbS00006379g0022.1 protein AED:0.09 eAED:0.09 QI:0|0|0.75|0.8|0.8|1|1|5|273|133; (*GB) gi|76161008|gb|ABA40467.1| (e_value=5e-73) glycoprotein-like protein [Solanum tuberosum];; (*SWP) sp|Q9SIM4|RL14

Query	Observed	Mr(expt)	Mr(calc)	ppm	Miss	Score	Expect	Rank	Unique	Peptide
11433	543.7854	1085.5562	1085.5539	2.14	0	33	0.035	1	U	R.ALVDAPDMVR.S

Proteins matching the same set of peptides:
NbS00007773g0008.1 **Mass:** 17765 **Score:** 33 **Matches:** 1(1) **Sequences:** 1(1)
NbS00007773g0008.1 protein AED:0.46 eAED:0.46 QI:0|0.66|0.75|0.75|1|1|4|0|153; (*GB) gi|76161008|gb|ABA40467.1| (e_value=9e-63) glycoprotein-like protein [Solanum tuberosum];; (*SWP) sp|Q9SIM4|RL14
NbS00009111g0021.1 **Mass:** 19978 **Score:** 33 **Matches:** 1(1) **Sequences:** 1(1)
NbS00009111g0021.1 protein AED:0.21 eAED:0.21 QI:0|1|0.8|1|1|1|5|366|173; (*GB) gi|76161008|gb|ABA40467.1| (e_value=1e-74) glycoprotein-like protein [Solanum tuberosum];; (*SWP) sp|Q9SIM4|RL141_ARA
NbS00021394g0006.1 **Mass:** 22812 **Score:** 33 **Matches:** 1(1) **Sequences:** 1(1)
NbS00021394g0006.1 protein AED:0.40 eAED:0.49 QI:0|0.2|0.33|0.66|1|1|6|0|199; (*GB) gi|76161008|gb|ABA40467.1| (e_value=1e-59) glycoprotein-like protein [Solanum tuberosum];; (*SWP) sp|Q9T043|RL142
NbS00025105g0019.1 **Mass:** 15127 **Score:** 33 **Matches:** 1(1) **Sequences:** 1(1)
NbS00025105g0019.1 protein AED:0.09 eAED:0.09 QI:0|1|1|1|1|1|4|261|132; (*GB) gi|76161008|gb|ABA40467.1| (e_value=2e-72) glycoprotein-like protein [Solanum tuberosum];; (*SWP) sp|P55844|RL14_PEA (e
NbS00038226g0005.1 **Mass:** 15389 **Score:** 33 **Matches:** 1(1) **Sequences:** 1(1)
NbS00038226g0005.1 protein AED:0.23 eAED:0.23 QI:86|1|1|1|0.6|0.5|6|356|134; (*GB) gi|76161008|gb|ABA40467.1| (e_value=2e-75) glycoprotein-like protein [Solanum tuberosum];; (*SWP) sp|Q9T043|RL142_

220. NbS00035965g0001.1 **Mass:** 18299 **Score:** 33 **Matches:** 1(1) **Sequences:** 1(1) **emPAI:** 0.18
NbS00035965g0001.1 protein AED:0.18 eAED:0.19 QI:0|0|0|0.33|1|1|3|0|161; (*GB) gi|18394828|ref|NP_564105.1| (e_value=2e-19) S-phase kinase-associated protein 1 [Arabidopsis thaliana];; (*SWP) sp|Q9

Query	Observed	Mr(expt)	Mr(calc)	ppm	Miss	Score	Expect	Rank	Unique	Peptide
31	301.1400	900.3982	900.4301	-35.46	0	33	0.018	1	U	K.HAEVSGSSK.K

221. NbS00011397g0001.1 **Mass:** 14976 **Score:** 33 **Matches:** 1(1) **Sequences:** 1(1) **emPAI:** 0.23
NbS00011397g0001.1 protein AED:0.61 eAED:0.61 QI:0|0|0|0.5|1|1|2|0|130; (*SWP) sp|A1XQR9|RUXE_PIG (e_value=3e-31) Small nuclear ribonucleoprotein E OS=Sus scrofa GN=SNRPE PE=3 SV=1;; (*TAIR) AT4G3

Query	Observed	Mr(expt)	Mr(calc)	ppm	Miss	Score	Expect	Rank	Unique	Peptide
12456	602.8405	1203.6665	1203.6652	1.08	0	33	0.035	1	U	R.IQIWLFEQK.D

Proteins matching the same set of peptides:
NbS00011643g0015.1 **Mass:** 10334 **Score:** 33 **Matches:** 1(1) **Sequences:** 1(1)
NbS00011643g0015.1 protein AED:0.07 eAED:0.07 QI:151|1|1|1|0.8|0.83|6|432|88; (*GB) gi|356508935|ref|XP_003523208.1| (e_value=2e-56) PREDICTED: small nuclear ribonucleoprotein E-like [Glycine max];
NbS00034990g0011.1 **Mass:** 14072 **Score:** 33 **Matches:** 1(1) **Sequences:** 1(1)
NbS00034990g0011.1 protein AED:0.13 eAED:0.13 QI:153|0.8|0.83|1|0.8|0.66|6|389|118; (*GB) gi|356562483|ref|XP_003549500.1| (e_value=1e-53) PREDICTED: small nuclear ribonucleoprotein E [Glycine max]
NbS00046899g0003.1 **Mass:** 9817 **Score:** 33 **Matches:** 1(1) **Sequences:** 1(1)
NbS00046899g0003.1 protein AED:0.19 eAED:0.19 QI:0|0|0|1|1|1|2|0|84; (*GB) gi|356508935|ref|XP_003523208.1| (e_value=4e-50) PREDICTED: small nuclear ribonucleoprotein E-like [Glycine max];; (*SWP)
NbS00056919g0001.1 **Mass:** 9341 **Score:** 33 **Matches:** 1(1) **Sequences:** 1(1)
NbS00056919g0001.1 protein AED:0.17 eAED:0.17 QI:148|0.8|0.83|1|0.8|0.83|6|459|79; (*SWP) sp|A1XQR9|RUXE_PIG (e_value=1e-33) Small nuclear ribonucleoprotein E OS=Sus scrofa GN=SNRPE PE=3 SV=1;; (*T

222. NbS00020307g0016.1 **Mass:** 82868 **Score:** 33 **Matches:** 2(2) **Sequences:** 2(2) **emPAI:** 0.08
NbS00020307g0016.1 protein AED:0.13 eAED:0.13 QI:0|0.33|0.25|0.75|0.66|0.25|4|0|741; (*GB) gi|75249421|sp|Q93YF5.1|SUVH1_TOBAC (e_value=0.0) RecName: Full=Histone-lysine N-methyltransferase, H3 lys

Query	Observed	Mr(expt)	Mr(calc)	ppm	Miss	Score	Expect	Rank	Unique	Peptide
10568	485.7704	969.5262	969.5244	1.86	0	31	0.041	1	U	R.GVIDVTNPR.G
11617	555.3221	1108.6296	1108.6281	1.40	0	31	0.033	1	U	R.ILLAYDVFR.R

223. NbS00027551g0008.1 **Mass:** 274788 **Score:** 33 **Matches:** 1(1) **Sequences:** 1(1) **emPAI:** 0.01
NbS00027551g0008.1 protein AED:0.09 eAED:0.10 QI:319|0.53|0.48|1|0.84|0.81|27|401|2351; (*GB) gi|359482368|ref|XP_003632762.1| (e_value=0.0) PREDICTED: pre-mRNA-processing-splicing factor 8-like is

Query	Observed	Mr(expt)	Mr(calc)	ppm	Miss	Score	Expect	Rank	Unique	Peptide
9329	403.2446	804.4746	804.4745	0.06	0	33	0.026	1	U	K.FGDLILK.A

224. NbS00029120g0005.1 **Mass:** 58336 **Score:** 33 **Matches:** 1(1) **Sequences:** 1(1) **emPAI:** 0.06
NbS00029120g0005.1 protein AED:0.30 eAED:0.35 QI:0|0.4|0.16|1|1|1|6|0|502; (*GB) gi|225442557|ref|XP_002284272.1| (e_value=0.0) PREDICTED: uncharacterized protein LOC100258278 [Vitis vinifera];; (

Query	Observed	Mr(expt)	Mr(calc)	ppm	Miss	Score	Expect	Rank	Unique	Peptide
3243	369.5300	1105.5682	1105.5914	-20.97	1	33	0.051	1	U	-.MLERVLSSR.R

225. NbS00004321g0109.1 **Mass:** 30943 **Score:** 33 **Matches:** 1(1) **Sequences:** 1(1) **emPAI:** 0.11
NbS00004321g0109.1 protein AED:0.14 eAED:0.14 QI:204|1|0.66|1|1|1|3|255|287

Query	Observed	Mr(expt)	Mr(calc)	ppm	Miss	Score	Expect	Rank	Unique	Peptide
11576	552.7762	1103.5379	1103.5400	-1.85	0	33	0.043	1	U	K.AFQSAYYVR.Y

Proteins matching the same set of peptides:
NbS00004583g0003.1 **Mass:** 17907 **Score:** 33 **Matches:** 1(1) **Sequences:** 1(1)
NbS00004583g0003.1 protein AED:0.19 eAED:0.22 QI:0|0.5|0|1|1|1|3|0|164; (*GB) gi|5327263|emb|CAB46351.1| (e_value=6e-92) major intrinsic protein 2 [Solanum tuberosum];; (*SWP) sp|Q8H5N9|PIP21_ORYSJ
NbS00029456g0006.1 **Mass:** 33754 **Score:** 33 **Matches:** 1(1) **Sequences:** 1(1)
NbS00029456g0006.1 protein AED:0.22 eAED:0.22 QI:128|0.66|0.5|1|1|1|4|245|312; (*GB) gi|78191436|gb|ABB29939.1| (e_value=7e-178) major intrinsic protein 2-like [Solanum tuberosum];; (*SWP) sp|Q9ATN

226. NbS00000561g0021.1 **Mass:** 37924 **Score:** 33 **Matches:** 1(1) **Sequences:** 1(1) **emPAI:** 0.09
NbS00000561g0021.1 protein AED:0.23 eAED:0.34 QI:0|0|0|1|0.2|0.16|6|0|324; (*GB) gi|19911171|dbj|BAB86912.1| (e_value=5e-70) putative cytochrome P450 [Solanum tuberosum];; (*SWP) sp|Q05047|C72A1_CA

Query	Observed	Mr(expt)	Mr(calc)	ppm	Miss	Score	Expect	Rank	Unique	Peptide
15054	494.9284	1481.7633	1481.8089	-30.79	1	33	0.036	1	U	R.NEVLQIIGKDEPK.F

Proteins matching the same set of peptides:
NbS00000561g0024.1 **Mass:** 54217 **Score:** 33 **Matches:** 1(1) **Sequences:** 1(1)
NbS00000561g0024.1 protein AED:0.26 eAED:0.26 QI:0|0.2|0.16|1|0.4|0.33|6|0|466; (*GB) gi|242053687|ref|XP_002455989.1| (e_value=1e-82) hypothetical protein SORBIDRAFT_03g028560 [Sorghum bicolor];;
NbS00000561g0025.1 **Mass:** 34036 **Score:** 33 **Matches:** 1(1) **Sequences:** 1(1)
NbS00000561g0025.1 protein AED:0.24 eAED:0.27 QI:0|0|0|1|0.33|0.25|4|0|295; (*GB) gi|4376203|gb|AAB05376.3| (e_value=4e-98) putative cytochrome P-450 [Nicotiana plumbaginifolia];; (*SWP) sp|Q05047
NbS00052797g0005.1 **Mass:** 22017 **Score:** 33 **Matches:** 1(1) **Sequences:** 1(1)
NbS00052797g0005.1 protein AED:0.35 eAED:0.35 QI:0|0|0|1|1|1|2|0|194; (*SWP) sp|Q05047|C72A1_CATRO (e_value=4e-66) Secologanin synthase OS=Catharanthus roseus GN=CYP72A1 PE=2 SV=1;; (*TAIR) AT3G14

227. NbS00008412g0009.1 **Mass:** 86673 **Score:** 32 **Matches:** 1(1) **Sequences:** 1(1) **emPAI:** 0.04
NbS00008412g0009.1 protein AED:0.13 eAED:0.13 QI:0|0.75|0.55|1|0.75|0.55|9|0|802; (*GB) gi|194396261|gb|ACF60500.1| (e_value=0.0) plastid transketolase [Nicotiana tabacum];; (*SWP) sp|Q43848|TKTC_S

Query	Observed	Mr(expt)	Mr(calc)	ppm	Miss	Score	Expect	Rank	Unique	Peptide
12588	611.3279	1220.6413	1220.6401	0.99	0	32	0.043	1	U	K.VTTTIGFGSPNK.A

Proteins matching the same set of peptides:
NbS00044448g0012.1 **Mass:** 82518 **Score:** 32 **Matches:** 1(1) **Sequences:** 1(1)
NbS00044448g0012.1 protein AED:0.24 eAED:0.25 QI:232|0.85|0.87|1|1|1|8|0|762; (*GB) gi|194396261|gb|ACF60500.1| (e_value=0.0) plastid transketolase [Nicotiana tabacum];; (*SWP) sp|Q43848|TKTC_SOLTU

228. NbS00018705g0023.1 **Mass:** 7957 **Score:** 32 **Matches:** 1(1) **Sequences:** 1(1) **emPAI:** 0.44
NbS00018705g0023.1 protein AED:0.30 eAED:0.31 QI:0|0.66|0.75|1|0.33|0.75|4|388|71; (*GB) gi|242050850|ref|XP_002463169.1| (e_value=7e-39) hypothetical protein SORBIDRAFT_02g039000 [Sorghum bicolor]

Query	Observed	Mr(expt)	Mr(calc)	ppm	Miss	Score	Expect	Rank	Unique	Peptide
15852	777.9285	1553.8424	1553.8413	0.69	0	32	0.027	1	U	R.GNSVVTIEALEPVAR.A

Proteins matching the same set of peptides:
NbS00024371g0006.1 **Mass:** 8908 **Score:** 32 **Matches:** 1(1) **Sequences:** 1(1)
NbS00024371g0006.1 protein AED:0.00 eAED:0.00 QI:102|1|1|1|1|1|4|366|80; (*GB) gi|242050850|ref|XP_002463169.1| (e_value=4e-48) hypothetical protein SORBIDRAFT_02g039000 [Sorghum bicolor];; (*SWP)

229. NbS00006168g0008.1 **Mass:** 51305 **Score:** 32 **Matches:** 1(1) **Sequences:** 1(1) **emPAI:** 0.06
NbS00006168g0008.1 protein AED:0.11 eAED:0.11 QI:168|0|0|1|0|0|2|0|473; (*SWP) sp|Q9LHE3|ASPG2_ARATH (e_value=6e-108) Protein ASPARTIC PROTEASE IN GUARD CELL 2 OS=Arabidopsis thaliana GN=ASPG2 PE=2

Query	Observed	Mr(expt)	Mr(calc)	ppm	Miss	Score	Expect	Rank	Unique	Peptide
17914	895.4501	1788.8856	1788.8854	0.11	0	32	0.035	1	U	K.LDAAGDGGVIVDSGTSVTR.L

Proteins matching the same set of peptides:
NbS00019115g0001.1 **Mass:** 53150 **Score:** 32 **Matches:** 1(1) **Sequences:** 1(1)
NbS00019115g0001.1 protein AED:0.03 eAED:0.03 QI:0|-1|0|1|-1|1|1|0|492; (*GB) gi|255564685|ref|XP_002523337.1| (e_value=0.0) Aspartic proteinase nepenthesin-1 precursor, putative [Ricinus communis]

---

230. NbS00005023g0005.1 **Mass:** 31867 **Score:** 32 **Matches:** 1(1) **Sequences:** 1(1) **emPAI:** 0.10
NbS00005023g0005.1 protein AED:0.11 eAED:0.11 QI:134|1|1|1|0.8|0.83|6|218|290; (*GB) gi|71370259|gb|AAZ30377.1| (e_value=0.0) PHB2 [Nicotiana benthamiana];; (*SWP) sp|Q54Q31|PHB2_DICDI (e_value=8e-

Query	Observed	Mr(expt)	Mr(calc)	ppm	Miss	Score	Expect	Rank	Unique	Peptide
19174	1007.5618	2013.1090	2013.1007	4.12	0	32	0.019	1	U	K.SAQLIGQSIANNPAFITLR.K

Proteins matching the same set of peptides:
NbS00015766g0001.1 **Mass:** 37124 **Score:** 32 **Matches:** 1(1) **Sequences:** 1(1)
NbS00015766g0001.1 protein AED:0.29 eAED:0.29 QI:0|0.6|0.66|0.83|1|1|6|219|337; (*GB) gi|71370259|gb|AAZ30377.1| (e_value=1e-179) PHB2 [Nicotiana benthamiana];; (*SWP) sp|Q54Q31|PHB2_DICDI (e_value

---

231. NbS00001184g0009.1 **Mass:** 49183 **Score:** 32 **Matches:** 1(1) **Sequences:** 1(1) **emPAI:** 0.07
NbS00001184g0009.1 protein AED:0.24 eAED:0.25 QI:0|0.25|0.2|1|1|1|5|0|443; (*GB) gi|17402469|emb|CAD13177.1| (e_value=0.0) alpha-tubulin [Nicotiana tabacum];; (*SWP) sp|P46259|TBA1_PEA (e_value=0.(

Query	Observed	Mr(expt)	Mr(calc)	ppm	Miss	Score	Expect	Rank	Unique	Peptide
10782	501.2852	1000.5559	1000.5553	0.58	0	32	0.048	1	U	K.DVNAAVATIK.T

Proteins matching the same set of peptides:
NbS00001594g0015.1 **Mass:** 62334 **Score:** 32 **Matches:** 1(1) **Sequences:** 1(1)
NbS00001594g0015.1 protein AED:0.04 eAED:0.04 QI:64|0.5|0.6|1|0.75|0.6|5|0|553; (*GB) gi|268619136|gb|ACZ13344.1| (e_value=0.0) tubulin alpha chain [Bursaphelenchus xylophilus];; (*SWP) sp|Q9ZRR5|
NbS00003471g0210.1 **Mass:** 46317 **Score:** 32 **Matches:** 1(1) **Sequences:** 1(1)
NbS00003471g0210.1 protein AED:0.09 eAED:0.09 QI:260|1|0.75|1|0.66|0.5|4|0|414
NbS00003775g0004.1 **Mass:** 57025 **Score:** 32 **Matches:** 1(1) **Sequences:** 1(1)
NbS00003775g0004.1 protein AED:0.20 eAED:0.27 QI:205|0.57|0.75|1|0.85|0.75|8|433|509; (*GB) gi|225435758|ref|XP_002285721.1| (e_value=0.0) PREDICTED: tubulin alpha-1 chain [Vitis vinifera];; (*SWP)
NbS00006458g0003.1 **Mass:** 55859 **Score:** 32 **Matches:** 1(1) **Sequences:** 1(1)
NbS00006458g0003.1 protein AED:0.07 eAED:0.07 QI:12|0.33|0.25|1|0.66|0.5|4|0|500; (*GB) gi|17402471|emb|CAD13178.1| (e_value=0.0) alpha-tubulin [Nicotiana tabacum];; (*SWP) sp|P33629|TBA_PRUDU (e_v
NbS00026888g0002.1 **Mass:** 33863 **Score:** 32 **Matches:** 1(1) **Sequences:** 1(1)
NbS00026888g0002.1 protein AED:0.13 eAED:0.20 QI:0|0|0|0.5|1|1|2|0|298; (*SWP) sp|P14641|TBA2_MAIZE (e_value=1e-141) Tubulin alpha-2 chain OS=Zea mays GN=TUBA2 PE=3 SV=1;; (*TAIR) AT4G14960.1 (e_va
NbS00027138g0007.1 **Mass:** 48793 **Score:** 32 **Matches:** 1(1) **Sequences:** 1(1)
NbS00027138g0007.1 protein AED:0.29 eAED:0.30 QI:0|0.6|0.66|1|0.8|0.83|6|219|437; (*GB) gi|225435758|ref|XP_002285721.1| (e_value=0.0) PREDICTED: tubulin alpha-1 chain [Vitis vinifera];; (*SWP) sp
NbS00031544g0010.1 **Mass:** 50360 **Score:** 32 **Matches:** 1(1) **Sequences:** 1(1)
NbS00031544g0010.1 protein AED:0.10 eAED:0.10 QI:4|1|1|1|1|1|4|217|450; (*GB) gi|386870485|gb|AFJ42573.1| (e_value=0.0) alpha-tubulin [Sesamum indicum];; (*SWP) sp|P33629|TBA_PRUDU (e_value=0.0) Tu
NbS00038051g0004.1 **Mass:** 55743 **Score:** 32 **Matches:** 1(1) **Sequences:** 1(1)
NbS00038051g0004.1 protein AED:0.04 eAED:0.04 QI:0|0|0|1|1|1|3|0|493; (*GB) gi|17402469|emb|CAD13177.1| (e_value=0.0) alpha-tubulin [Nicotiana tabacum];; (*SWP) sp|P33629|TBA_PRUDU (e_value=0.0) Tu
NbS00043519g0004.1 **Mass:** 55523 **Score:** 32 **Matches:** 1(1) **Sequences:** 1(1)
NbS00043519g0004.1 protein AED:0.10 eAED:0.10 QI:0|0.4|0.16|1|1|1|6|0|496; (*GB) gi|54036492|sp|Q6VAF9.1|TBA4_GOSHI (e_value=0.0) RecName: Full=Tubulin alpha-4 chain; AltName: Full=Alpha-4-tubulin;
NbS00058547g0002.1 **Mass:** 58434 **Score:** 32 **Matches:** 1(1) **Sequences:** 1(1)
NbS00058547g0002.1 protein AED:0.08 eAED:0.08 QI:4|0.75|0.4|1|1|1|5|0|526; (*GB) gi|348515729|ref|XP_003445392.1| (e_value=0.0) PREDICTED: tubulin alpha chain-like [Oreochromis niloticus];; (*SWP)
NbC24729591g0004.1 **Mass:** 15383 **Score:** 32 **Matches:** 1(1) **Sequences:** 1(1)
NbC24729591g0004.1 protein AED:0.03 eAED:0.07 QI:0|0|0|1|1|1|2|0|134; (*GB) gi|359497525|ref|XP_003635553.1| (e_value=5e-65) PREDICTED: tubulin alpha-1 chain-like, partial [Vitis vinifera];; (*SWP)
NbS00050901g0019.1 **Mass:** 57748 **Score:** 32 **Matches:** 1(1) **Sequences:** 1(1)
NbS00050901g0019.1 protein AED:0.02 eAED:0.02 QI:0|0|0|1|0.5|0.33|3|0|513; (*GB) gi|77745465|gb|ABB02631.1| (e_value=0.0) unknown [Solanum tuberosum];; (*SWP) sp|P33629|TBA_PRUDU (e_value=0.0) Tubu

---

232. NbS00059407g0001.1 **Mass:** 15599 **Score:** 32 **Matches:** 1(1) **Sequences:** 1(1) **emPAI:** 0.22
NbS00059407g0001.1 protein AED:0.31 eAED:0.31 QI:0|0|0|1|1|1|3|0|138; (*SWP) sp|P06005|PSBD_SPIOL (e_value=4e-44) Photosystem II D2 protein OS=Spinacia oleracea GN=psbD PE=1 SV=3;; (*ITAG) ATCG002

Query	Observed	Mr(expt)	Mr(calc)	ppm	Miss	Score	Expect	Rank	Unique	Peptide
11084	521.3064	1040.5982	1040.5978	0.41	0	32	0.026	1	U	K.NILLNEGIR.A

---

233. NbS00017399g0001.1 **Mass:** 41631 **Score:** 32 **Matches:** 1(1) **Sequences:** 1(1) **emPAI:** 0.08
NbS00017399g0001.1 protein AED:0.12 eAED:0.12 QI:0|0.75|0.2|1|1|1|5|0|372; (*GB) gi|399212|sp|P31541.1|CLPAA_SOLLC (e_value=0.0) RecName: Full=ATP-dependent Clp protease ATP-binding subunit clpA ho

Query	Observed	Mr(expt)	Mr(calc)	ppm	Miss	Score	Expect	Rank	Unique	Peptide
12027	580.3010	1158.5874	1158.5880	-0.58	0	32	0.054	1	U	K.AIDLIDEAGSR.V

Proteins matching the same set of peptides:

NbS00017400g0001.1 **Mass:** 66531 **Score:** 32 **Matches:** 1(1) **Sequences:** 1(1)
NbS00017400g0001.1 protein AED:0.39 eAED:0.39 QI:0|0.75|0.2|1|1|1|5|0|588; (*SWP) sp|P31541|CLPAA_SOLLC (e_value=0.0) ATP-dependent Clp protease ATP-binding subunit clpA homolog CD4A, chloroplastic

NbS00017400g0010.1 **Mass:** 41631 **Score:** 32 **Matches:** 1(1) **Sequences:** 1(1)
NbS00017400g0010.1 protein AED:0.12 eAED:0.12 QI:0|1|0.4|1|1|1|5|0|372; (*SWP) sp|P31541|CLPAA_SOLLC (e_value=0.0) ATP-dependent Clp protease ATP-binding subunit clpA homolog CD4A, chloroplastic OS

NbS00004792g0016.1 **Mass:** 104270 **Score:** 32 **Matches:** 1(1) **Sequences:** 1(1)
NbS00004792g0016.1 protein AED:0.26 eAED:0.27 QI:7|0.55|0.4|1|0.77|0.7|10|0|938; (*GB) gi|399213|sp|P31542.1|CLPAB_SOLLC (e_value=0.0) RecName: Full=ATP-dependent Clp protease ATP-binding subunit c

NbS00017399g0022.1 **Mass:** 100907 **Score:** 32 **Matches:** 1(1) **Sequences:** 1(1)
NbS00017399g0022.1 protein AED:0.20 eAED:0.21 QI:7|0.66|0.6|1|0.55|0.6|10|0|906; (*GB) gi|399213|sp|P31542.1|CLPAB_SOLLC (e_value=0.0) RecName: Full=ATP-dependent Clp protease ATP-binding subunit c

NbS00017400g0004.1 **Mass:** 102872 **Score:** 32 **Matches:** 1(1) **Sequences:** 1(1)
NbS00017400g0004.1 protein AED:0.33 eAED:0.33 QI:0|0.7|0.54|0.90|1|1|11|0|928; (*SWP) sp|P31542|CLPAB_SOLLC (e_value=0.0) ATP-dependent Clp protease ATP-binding subunit clpA homolog CD4B, chloropla

NbS00020863g0003.1 **Mass:** 59028 **Score:** 32 **Matches:** 1(1) **Sequences:** 1(1)
NbS00020863g0003.1 protein AED:0.25 eAED:0.25 QI:0|0.62|0.33|1|1|1|9|0|535; (*GB) gi|399213|sp|P31542.1|CLPAB_SOLLC (e_value=0.0) RecName: Full=ATP-dependent Clp protease ATP-binding subunit clpA h

NbS00024706g0002.1 **Mass:** 100869 **Score:** 32 **Matches:** 1(1) **Sequences:** 1(1)
NbS00024706g0002.1 protein AED:0.27 eAED:0.27 QI:0|0.87|0.66|1|1|1|9|0|908; (*GB) gi|399213|sp|P31542.1|CLPAB_SOLLC (e_value=0.0) RecName: Full=ATP-dependent Clp protease ATP-binding subunit clpA h

---

234. NbS00005732g0004.1 **Mass:** 51476 **Score:** 32 **Matches:** 1(1) **Sequences:** 1(1) **emPAI:** 0.06
NbS00005732g0004.1 protein AED:0.15 eAED:0.15 QI:0|0.2|0|0.83|1|1|6|0|478; (*GB) gi|255543963|ref|XP_002513044.1| (e_value=3e-173) Aspartic proteinase nepenthesin-1 precursor, putative [Ricinus cor

Query	Observed	Mr(expt)	Mr(calc)	ppm	Miss	Score	Expect	Rank	Unique	Peptide
14149	688.3683	1374.7220	1374.7184	2.67	0	32	0.047	1	U	R.SSFYYVNLVGVK.V

Proteins matching the same set of peptides:

NbS00037255g0003.1 **Mass:** 47713 **Score:** 32 **Matches:** 1(1) **Sequences:** 1(1)
NbS00037255g0003.1 protein AED:0.15 eAED:0.15 QI:0|1|0.66|1|1|1|3|0|438; (*GB) gi|255543963|ref|XP_002513044.1| (e_value=0.0) Aspartic proteinase nepenthesin-1 precursor, putative [Ricinus communis

---

235. NbS00006675g0003.1 **Mass:** 66696 **Score:** 32 **Matches:** 1(1) **Sequences:** 1(1) **emPAI:** 0.05
NbS00006675g0003.1 protein AED:0.09 eAED:0.09 QI:0|0|0|0.66|1|1|3|416|592; (*GB) gi|225470260|ref|XP_002264285.1| (e_value=0.0) PREDICTED: U3 small nucleolar RNA-associated protein 18 homolog [Vit:

Query	Observed	Mr(expt)	Mr(calc)	ppm	Miss	Score	Expect	Rank	Unique	Peptide
16891	821.4570	1640.8994	1640.8985	0.54	0	32	0.028	1	U	K.LVGDILQSNEDLVVK.S

Proteins matching the same set of peptides:

NbS00009753g0001.1 **Mass:** 26465 **Score:** 32 **Matches:** 1(1) **Sequences:** 1(1)
NbS00009753g0001.1 protein AED:0.26 eAED:0.26 QI:309|1|0.5|1|0|0|2|0|235; (*SWP) sp|Q9FMU5|UTP18_ARATH (e_value=7e-69) U3 small nucleolar RNA-associated protein 18 homolog OS=Arabidopsis thaliana (

NbS00017899g0002.1 **Mass:** 65190 **Score:** 32 **Matches:** 1(1) **Sequences:** 1(1)
NbS00017899g0002.1 protein AED:0.11 eAED:0.11 QI:0|-1|0|1|-1|1|1|0|583; (*GB) gi|297735496|emb|CBI17936.3| (e_value=0.0) unnamed protein product [Vitis vinifera];; (*SWP) sp|Q9FMU5|UTP18_ARATH (e_v

---

236. NbS00043662g0002.1 **Score:** 32 **Matches:** 1(1) **Sequences:** 1(1) **emPAI:** 0.16
NbS00043662g0002.1 protein ; (*GB) gi|296089862|emb|CBI39681.3| (e_value=5e-45) unnamed protein product [Vitis vinifera];; (*SWP) sp|Q8VYE4|PTR12_ARATH (e_value=4e-25) Probable peptide/nitrate tran

Query	Observed	Mr(expt)	Mr(calc)	ppm	Miss	Score	Expect	Rank	Unique	Peptide
9517	416.2504	830.4862	830.4862	0.02	0	32	0.051	2	U	R.TITDLLR.R

---

237. NbS00027742g0013.1 **Mass:** 39244 **Score:** 32 **Matches:** 1(1) **Sequences:** 1(1) **emPAI:** 0.08
NbS00027742g0013.1 protein AED:0.00 eAED:0.01 QI:0|1|1|1|1|1|7|309|363; (*GB) gi|77416977|gb|ABA81884.1| (e_value=4e-80) nuclear RNA binding protein-like [Solanum tuberosum];; (*TAIR) AT5G47210.1

Query	Observed	Mr(expt)	Mr(calc)	ppm	Miss	Score	Expect	Rank	Unique	Peptide
14026	680.8597	1359.7048	1359.7034	1.01	0	32	0.049	1	U	K.IEDVGQFPSLGAK.-

---

238. NbS00005060g0011.1 **Mass:** 13073 **Score:** 31 **Matches:** 1(1) **Sequences:** 1(1) **emPAI:** 0.26
NbS00005060g0011.1 protein AED:0.28 eAED:0.49 QI:0|0|0|1|0|0|2|0|110; (*GB) gi|356525523|ref|XP_003531374.1| (e_value=1e-24) PREDICTED: cytochrome P450 71A1-like [Glycine max];; (*SWP) sp|P24465|C

Query	Observed	Mr(expt)	Mr(calc)	ppm	Miss	Score	Expect	Rank	Unique	Peptide
10897	339.5380	1015.5922	1015.5913	0.82	1	31	0.041	2	U	R.KEEVSILAK.S

---

239. NbS00009355g0003.1 **Mass:** 66874 **Score:** 31 **Matches:** 1(1) **Sequences:** 1(1) **emPAI:** 0.05
NbS00009355g0003.1 protein AED:0.19 eAED:0.19 QI:0|-1|0|1|-1|1|1|0|585; (*GB) gi|307135979|gb|ADN33838.1| (e_value=0.0) pre-mRNA splicing factor [Cucumis melo subsp. melo];; (*SWP) sp|Q9DC48|PRP17_

Query	Observed	Mr(expt)	Mr(calc)	ppm	Miss	Score	Expect	Rank	Unique	Peptide
8449	651.8800	1301.7454	1301.7384	5.45	0	31	0.032	1	U	R.VWEYGIPVVIK.Y

Proteins matching the same set of peptides:
NbS00039920g0014.1 **Mass:** 69257 **Score:** 31 **Matches:** 1(1) **Sequences:** 1(1)
NbS00039920g0014.1 protein AED:0.18 eAED:0.18 QI:0|0|0|0.5|0|0|4|0|608; (*GB) gi|307135979|gb|ADN33838.1| (e_value=0.0) pre-mRNA splicing factor [Cucumis melo subsp. melo];; (*SWP) sp|Q9DC48|PRP17_

240. NbS00046532g0005.1 **Mass:** 83793 **Score:** 31 **Matches:** 1(1) **Sequences:** 1(1) **emPAI:** 0.04
NbS00046532g0005.1 protein AED:0.23 eAED:0.23 QI:236|1|1|1|1|1|10|251|775; (*GB) gi|224116492|ref|XP_002317314.1| (e_value=0.0) predicted protein [Populus trichocarpa];; (*SWP) sp|O65351|SUBL_ARATH

Query	Observed	Mr(expt)	Mr(calc)	ppm	Miss	Score	Expect	Rank	Unique	Peptide
11944	575.3174	1148.6203	1148.6190	1.20	0	31	0.055	1	U	R.AGGIGYILGNSK.A

241. NbS00000211g0112.1 **Mass:** 43382 **Score:** 31 **Matches:** 1(0) **Sequences:** 1(0) **emPAI:** 0.08
NbS00000211g0112.1 protein AED:0.16 eAED:0.16 QI:0|0.88|0.8|1|0.88|0.8|10|0|389

Query	Observed	Mr(expt)	Mr(calc)	ppm	Miss	Score	Expect	Rank	Unique	Peptide
9336	404.2216	806.4286	806.4286	-0.11	0	31	0.073	1	U	R.LDFAVSR.E

Proteins matching the same set of peptides:
NbS00029128g0009.1 **Mass:** 39264 **Score:** 31 **Matches:** 1(0) **Sequences:** 1(0)
NbS00029128g0009.1 protein AED:0.23 eAED:0.23 QI:0|0.75|0.66|1|0.87|0.77|9|1155|348; (*GB) gi|6899972|emb|CAB71293.1| (e_value=0.0) chloroplast ferredoxin-NADP+ oxidoreductase precursor [Capsicum a

242. NbS00056997g0003.1 **Mass:** 105427 **Score:** 31 **Matches:** 1(1) **Sequences:** 1(1) **emPAI:** 0.03
NbS00056997g0003.1 protein AED:0.27 eAED:0.27 QI:218|1|1|1|1|1|3|277|931; (*GB) gi|8919178|emb|CAB96077.1| (e_value=0.0) alpha-glucosidase [Solanum tuberosum];; (*SWP) sp|Q9S7Y7|XYL1_ARATH (e_value

Query	Observed	Mr(expt)	Mr(calc)	ppm	Miss	Score	Expect	Rank	Unique	Peptide
9735	436.7637	871.5129	871.5127	0.22	0	31	0.062	1	U	K.VTVLGLDR.I

243. NbS00020649g0003.1 **Mass:** 72073 **Score:** 31 **Matches:** 1(1) **Sequences:** 1(1) **emPAI:** 0.05
NbS00020649g0003.1 protein AED:0.03 eAED:0.03 QI:46|1|1|1|1|1|3|317|655; (*SWP) sp|P09114|ILVB2_TOBAC (e_value=0.0) Acetolactate synthase 2, chloroplastic OS=Nicotiana tabacum GN=ALS SURB PE=1 SV=1

Query	Observed	Mr(expt)	Mr(calc)	ppm	Miss	Score	Expect	Rank	Unique	Peptide
1961	328.5400	982.5982	982.6175	-19.66	0	31	0.02	1	U	K.ILLEQIVR.L

244. NbS00040938g0004.1 **Mass:** 36439 **Score:** 31 **Matches:** 1(1) **Sequences:** 1(1) **emPAI:** 0.09
NbS00040938g0004.1 protein AED:0.11 eAED:0.11 QI:0|0|0|1|0.5|0.66|3|0|334; (*TAIR) AT4G29310.1 (e_value=2e-132) | Symbols: | Protein of unknown function (DUF1005) | chr4:14437892-14439609 RVERSE LE

Query	Observed	Mr(expt)	Mr(calc)	ppm	Miss	Score	Expect	Rank	Unique	Peptide
1961	328.5400	982.5982	982.6426	-45.24	0	31	0.02	1	U	R.LIIEALALK.L

245. NbS00007750g0014.1 **Mass:** 22429 **Score:** 31 **Matches:** 1(1) **Sequences:** 1(1) **emPAI:** 0.15
NbS00007750g0014.1 protein AED:0.40 eAED:0.40 QI:0|0.75|0.6|1|0.75|0.6|5|0|199; (*GB) gi|351723391|ref|NP_001237789.1| (e_value=4e-117) uncharacterized protein LOC100500164 [Glycine max];; (*SWP) s

Query	Observed	Mr(expt)	Mr(calc)	ppm	Miss	Score	Expect	Rank	Unique	Peptide
12371	598.3615	1194.7085	1194.7084	0.03	0	31	0.017	1	U	R.APLGQNTLLLR.G

Proteins matching the same set of peptides:
NbS00013932g0015.1 **Mass:** 27872 **Score:** 31 **Matches:** 1(1) **Sequences:** 1(1)
NbS00013932g0015.1 protein AED:0.35 eAED:0.35 QI:0|0.4|0.5|1|0.8|0.66|6|0|249; (*GB) gi|351723391|ref|NP_001237789.1| (e_value=4e-114) uncharacterized protein LOC100500164 [Glycine max];; (*SWP) sp
NbS00021061g0110.1 **Mass:** 20934 **Score:** 31 **Matches:** 1(1) **Sequences:** 1(1)
NbS00021061g0110.1 protein AED:0.40 eAED:0.40 QI:0|1|0.75|1|1|1|4|0|187
NbS00025676g0010.1 **Mass:** 20946 **Score:** 31 **Matches:** 1(1) **Sequences:** 1(1)
NbS00025676g0010.1 protein AED:0.34 eAED:0.34 QI:0|1|0.75|1|1|1|4|0|187; (*GB) gi|351723391|ref|NP_001237789.1| (e_value=7e-113) uncharacterized protein LOC100500164 [Glycine max];; (*SWP) sp|P4279

246. NbS00000250g0010.1 **Mass:** 48508 **Score:** 31 **Matches:** 1(1) **Sequences:** 1(1) **emPAI:** 0.07
NbS00000250g0010.1 protein AED:0.23 eAED:0.23 QI:0|0.83|0.78|0.89|0.77|0.78|19|437|448; (*GB) gi|238814974|gb|ACR56690.1| (e_value=0.0) enolase [Nicotiana tabacum];; (*SWP) sp|P26300|ENO_SOLLC (e_v

Query	Observed	Mr(expt)	Mr(calc)	ppm	Miss	Score	Expect	Rank	Unique	Peptide
18033	907.4319	1812.8492	1812.8530	-2.08	0	31	0.039	1	U	R.IEEELGSDAVYAGASFR.K

Proteins matching the same set of peptides:
NbS00026102g0006.1 **Mass:** 50192 **Score:** 31 **Matches:** 1(1) **Sequences:** 1(1)
NbS00026102g0006.1 protein AED:0.19 eAED:0.19 QI:0|0.86|0.87|1|0.93|0.87|16|429|461; (*GB) gi|238814974|gb|ACR56690.1| (e_value=0.0) enolase [Nicotiana tabacum];; (*SWP) sp|P26300|ENO_SOLLC (e_valu

---

247. NbS00002556g0019.1 **Mass:** 32181 **Score:** 31 **Matches:** 1(0) **Sequences:** 1(0) **emPAI:** 0.10
NbS00002556g0019.1 protein AED:0.14 eAED:0.14 QI:0|1|0.33|1|1|1|3|0|298; (*SWP) sp|Q9FL12|DEGP9_ARATH (e_value=1e-127) Protease Do-like 9 OS=Arabidopsis thaliana GN=DEGP9 PE=2 SV=1;; (*TAIR) AT5G4(

Query	Observed	Mr(expt)	Mr(calc)	ppm	Miss	Score	Expect	Rank	Unique	Peptide
16546	**808.9141**	**1615.8137**	**1615.8166**	**-1.77**	**0**	**31**	**0.056**	**1**	**U**	**R.VASLEGTTVNNADVAR.V**

---

248. NbS00016013g0004.1 **Mass:** 36087 **Score:** 31 **Matches:** 1(1) **Sequences:** 1(1) **emPAI:** 0.09
NbS00016013g0004.1 protein AED:0.27 eAED:0.27 QI:0|1|0.66|1|1|0.66|3|0|319; (*GB) gi|255555327|ref|XP_002518700.1| (e_value=0.0) DNA-directed RNA polymerase II subunit, putative [Ricinus communis];

Query	Observed	Mr(expt)	Mr(calc)	ppm	Miss	Score	Expect	Rank	Unique	Peptide
19815	**735.9817**	**2204.9232**	**2204.9644**	**-18.68**	**0**	**31**	**0.014**	**1**	**U**	**R.LSDDTVEADDQFGELGAHMR.G**

Proteins matching the same set of peptides:
NbS00035513g0006.1 **Mass:** 36000 **Score:** 31 **Matches:** 1(1) **Sequences:** 1(1)
NbS00035513g0006.1 protein AED:0.30 eAED:0.30 QI:0|0.5|0.66|1|1|1|3|0|318; (*GB) gi|255555327|ref|XP_002518700.1| (e_value=0.0) DNA-directed RNA polymerase II subunit, putative [Ricinus communis];;

---

249. NbS00005419g0006.1 **Score:** 31 **Matches:** 1(1) **Sequences:** 1(1) **emPAI:** 0.10
NbS00005419g0006.1 protein

Query	Observed	Mr(expt)	Mr(calc)	ppm	Miss	Score	Expect	Rank	Unique	Peptide
8744	349.2341	696.4536	696.4534	0.36	0	31	0.0094	3	U	K.IGGLPIK.G

---

250. NbS00004972g0108.1 **Mass:** 53046 **Score:** 30 **Matches:** 1(1) **Sequences:** 1(1) **emPAI:** 0.06
NbS00004972g0108.1 protein AED:0.17 eAED:0.17 QI:133|0|0.5|1|1|1|2|0|460

Query	Observed	Mr(expt)	Mr(calc)	ppm	Miss	Score	Expect	Rank	Unique	Peptide
10797	502.2994	1002.5842	1002.6147	-30.45	0	30	0.052	3	U	R.IMLVGLLTK.N

Proteins matching the same set of peptides:
NbS00015392g0110.1 **Score:** 30 **Matches:** 1(1) **Sequences:** 1(1)

---

251. NbS00006241g0007.1 **Score:** 30 **Matches:** 1(1) **Sequences:** 1(1) **emPAI:** 0.07
NbS00006241g0007.1 protein AED:0.11 eAED:0.19 QI:0|0.5|0.33|1|0|0|3|31|414; (*GB) gi|225470070|ref|XP_002269458.1| (e_value=1e-33) PREDICTED: uncharacterized protein LOC100259805 [Vitis vinifera];;

Query	Observed	Mr(expt)	Mr(calc)	ppm	Miss	Score	Expect	Rank	Unique	Peptide
10679	493.7562	985.4979	985.5305	-33.11	1	30	0.055	2	U	K.KGVGGGGGGGVK.R

Proteins matching the same set of peptides:
NbS00040287g0001.1 **Score:** 30 **Matches:** 1(1) **Sequences:** 1(1)

---

252. NbS00010046g0020.1 **Mass:** 63701 **Score:** 30 **Matches:** 1(1) **Sequences:** 1(1) **emPAI:** 0.05
NbS00010046g0020.1 protein AED:0.25 eAED:0.25 QI:0|0.76|0.66|0.94|0.64|0.66|18|0|588; (*GB) gi|171854667|dbj|BAG16523.1| (e_value=0.0) putative NADPH oxidoreductase [Capsicum chinense];; (*SWP) sp

Query	Observed	Mr(expt)	Mr(calc)	ppm	Miss	Score	Expect	Rank	Unique	Peptide
19303	**686.0511**	**2055.1316**	**2055.1252**	**3.12**	**0**	**30**	**0.025**	**1**	**U**	**R.LPLDSGFEAVGIIAAIGDAVK.N**

---

253. NbS00001581g0012.1 **Mass:** 24564 **Score:** 30 **Matches:** 1(1) **Sequences:** 1(1) **emPAI:** 0.14
NbS00001581g0012.1 protein AED:0.09 eAED:0.10 QI:0|1|0.6|1|0.75|0.8|5|0|220; (*GB) gi|242044476|ref|XP_002460109.1| (e_value=3e-117) hypothetical protein SORBIDRAFT_02g022890 [Sorghum bicolor];; (

Query	Observed	Mr(expt)	Mr(calc)	ppm	Miss	Score	Expect	Rank	Unique	Peptide
10245	**467.2797**	**932.5448**	**932.5443**	**0.48**	**0**	**30**	**0.057**	**1**	**U**	**K.YLAGLGIAR.Q**

Proteins matching the same set of peptides:
NbS00010885g0004.1 **Mass:** 34023 **Score:** 30 **Matches:** 1(1) **Sequences:** 1(1)

NbS00010885g0004.1 protein AED:0.24 eAED:0.24 QI:142|1|0.8|1|1|1|5|0|305; (*GB) gi|46452120|gb|AAS98165.1| (e_value=0.0) hypersensitive-induced reaction protein [Capsicum annuum];; (*SWP) sp|Q9FM1[illegible]

NbS00026918g0022.1 **Mass:** 31656 **Score:** 30 **Matches:** 1(1) **Sequences:** 1(1)

NbS00026918g0022.1 protein AED:0.29 eAED:0.30 QI:284|1|0.83|1|0.8|0.83|6|0|285; (*GB) gi|46452120|gb|AAS98165.1| (e_value=0.0) hypersensitive-induced reaction protein [Capsicum annuum];; (*SWP) sp

---

Mascot: http://www.matrixscience.com/
