# Supplementary material for: Alternative splicing regulation in plants by SP7-like effectors from symbiotic arbuscular mycorrhizal fungi
Source: Nat Commun. 2024 Aug 19;15:7107. doi: 10.1038/s41467-024-51512-5 (PMC11333574; doi:10.1038/s41467-024-51512-5)
Supplement: Supplementary file 10 — Source Data [file 41467_2024_51512_MOESM10_ESM.zip › Requena_8071-1_validation (RiSP7+SP).pdf]

(MATRIX SCIENCE) Mascot Search Results

```
User           :
Email          :
Search title   : Requena_8071
MS data file   : \\Server3\user\Kunden_Projekte\Requena_8071\8071\8071_1.mgf
Database       : Jessi 1 (4 sequences; 1794 residues)
Timestamp      : 1 Dec 2016 at 12:26:05 GMT
Significant hits: eGFP
                  SP7full
```

Probability Based Mowse Score

Ions score is -10*Log(P), where P is the probability that the observed match is a random event.
Individual ions scores > 5 indicate identity or extensive homology ($p<0.05$).
Protein scores are derived from ions scores as a non-probabilistic basis for ranking protein hits.

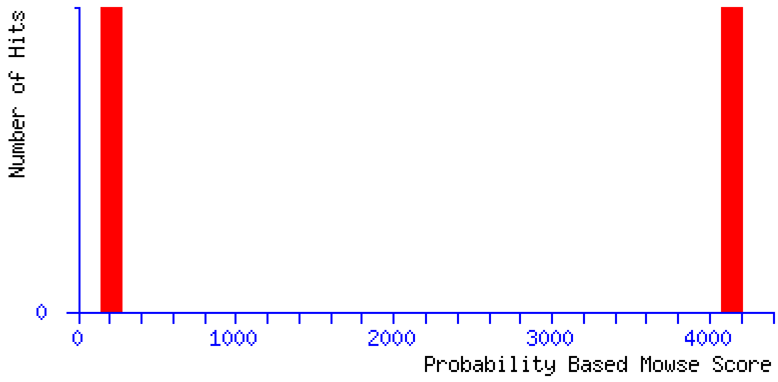

Peptide Summary Report

Format As | Peptide Summary | H

Significance threshold p< 0.05 | Max. number of hits AUTO

Standard scoring ○ MudPIT scoring ● | Ions score cut-off 20 | S

Show pop-ups ● Suppress pop-ups ○ | Sort unassigned Decreasing Score | R

Select All | Select None | Search Selected | ☐ Error tolerant | Archive Report

1. eGFP **Mass:** 27039 **Score:** 4

☐ Check to include this hit in error tolerant search or archive report

	Query	Observed	Mr(expt)	Mr(calc)	Delta	Miss	Score	Expect	Rank	Pe
☑	8203	602.2800	601.2727	601.2707	0.0020	0	24	0.00021	1	K.
☑	8470	328.1940	654.3734	654.3741	-0.0007	0	23	0.00024	1	R.
☑	8472	328.1942	654.3738	654.3741	-0.0003	0	(20)	0.00046	1	R.
☑	11145	525.7634	1049.5122	1049.5141	-0.0019	0	(34)	1.9e-005	1	K.
☑	11146	525.7637	1049.5129	1049.5141	-0.0012	0	72	3.1e-009	1	K.
☑	13006	633.7911	1265.5677	1265.5710	-0.0033	0	(65)	1.5e-008	1	K.
☑	13007	633.7922	1265.5698	1265.5710	-0.0012	0	(39)	7e-006	1	K.
	13008	633.7925	1265.5704	1265.5710	-0.0006	0	(65)	1.6e-008	1	K.

13009	633.7927	1265.5709	1265.5710	-0.0001	0	84	1.8e-010	1	K.
13010	633.7930	1265.5715	1265.5710	0.0005	0	(24)	0.00019	1	K.
13012	633.7933	1265.5721	1265.5710	0.0011	0	(33)	2.8e-005	1	K.
13013	633.7935	1265.5725	1265.5710	0.0015	0	(24)	0.00018	1	K.
13195	641.7897	1281.5648	1281.5659	-0.0011	0	(22)	0.00034	1	K.
13205	641.7902	1281.5658	1281.5659	-0.0001	0	(26)	0.00014	1	K.
13207	641.7902	1281.5659	1281.5659	-0.0000	0	(23)	0.00023	1	K.
13209	641.7903	1281.5661	1281.5659	0.0002	0	(31)	4.3e-005	1	K.
13211	641.7903	1281.5661	1281.5659	0.0002	0	(20)	0.00045	1	K.
13214	641.7904	1281.5663	1281.5659	0.0004	0	(21)	0.00035	1	K.
13217	641.7905	1281.5665	1281.5659	0.0006	0	(47)	9.8e-007	1	K.
13218	641.7905	1281.5665	1281.5659	0.0006	0	(47)	1e-006	1	K.
13219	641.7905	1281.5665	1281.5659	0.0006	0	(61)	3.8e-008	1	K.
13220	641.7905	1281.5665	1281.5659	0.0006	0	(48)	8.9e-007	1	K.
13226	641.7906	1281.5667	1281.5659	0.0008	0	(26)	0.00013	1	K.
13228	641.7906	1281.5667	1281.5659	0.0008	0	(46)	1.3e-006	1	K.
13230	641.7907	1281.5669	1281.5659	0.0010	0	(23)	0.00023	1	K.
13232	641.7907	1281.5669	1281.5659	0.0010	0	(42)	2.8e-006	1	K.
13236	641.7908	1281.5670	1281.5659	0.0011	0	(41)	4.3e-006	1	K.
13237	641.7908	1281.5670	1281.5659	0.0011	0	(25)	0.00017	1	K.
13239	641.7908	1281.5671	1281.5659	0.0012	0	(23)	0.00024	1	K.
13242	641.7909	1281.5672	1281.5659	0.0013	0	(22)	0.00034	1	K.
13243	641.7910	1281.5674	1281.5659	0.0015	0	(39)	5.8e-006	1	K.
13245	641.7911	1281.5676	1281.5659	0.0017	0	(27)	0.00011	1	K.
13247	641.7911	1281.5677	1281.5659	0.0018	0	(31)	4.3e-005	1	K.
13248	641.7913	1281.5680	1281.5659	0.0021	0	(37)	1.1e-005	1	K.
13252	641.7915	1281.5685	1281.5659	0.0026	0	(27)	0.00011	1	K.
13877	449.8902	1346.6489	1346.6506	-0.0018	1	(21)	0.00037	1	R.
13879	449.8905	1346.6497	1346.6506	-0.0009	1	(28)	7.2e-005	1	R.
13890	674.3328	1346.6510	1346.6506	0.0004	1	46	1.1e-006	1	R.
13892	674.3330	1346.6515	1346.6506	0.0008	1	(28)	8e-005	1	R.
13893	674.3330	1346.6515	1346.6506	0.0008	1	(40)	4.6e-006	1	R.
13894	674.3330	1346.6515	1346.6506	0.0008	1	(26)	0.00012	1	R.
13896	674.3332	1346.6518	1346.6506	0.0012	1	(31)	3.7e-005	1	R.
13897	674.3332	1346.6518	1346.6506	0.0012	1	(25)	0.00015	1	R.
13898	674.3333	1346.6521	1346.6506	0.0015	1	(34)	1.8e-005	1	R.
13899	674.3333	1346.6521	1346.6506	0.0015	1	(23)	0.00028	1	R.
13902	674.3336	1346.6526	1346.6506	0.0019	1	(38)	8.8e-006	1	R.
13904	674.3339	1346.6532	1346.6506	0.0026	1	(37)	1e-005	1	R.
14990	493.2593	1476.7562	1476.7572	-0.0010	1	(28)	7.8e-005	1	R.
14991	493.2596	1476.7569	1476.7572	-0.0003	1	(31)	4.3e-005	1	R.
14992	739.3858	1476.7571	1476.7572	-0.0001	1	(23)	0.00027	1	R.
14995	739.3862	1476.7579	1476.7572	0.0007	1	52	3.3e-007	1	R.
15001	493.2601	1476.7584	1476.7572	0.0012	1	(35)	1.4e-005	1	R.
15004	493.2603	1476.7590	1476.7572	0.0018	1	(51)	4.3e-007	1	R.
15005	739.3868	1476.7591	1476.7572	0.0019	1	(27)	9.5e-005	1	R.
15006	739.3868	1476.7591	1476.7572	0.0019	1	(31)	4.2e-005	1	R.
15007	739.3872	1476.7599	1476.7572	0.0027	1	(46)	1.3e-006	1	R.

15220	752.3300	1502.6454	1502.6524	-0.0070	0	(22)	0.00029	1	K.
15224	752.3322	1502.6499	1502.6524	-0.0026	0	(20)	0.00048	1	K.
15225	752.3324	1502.6502	1502.6524	-0.0022	0	(25)	0.00017	1	K.
15227	752.3327	1502.6509	1502.6524	-0.0016	0	(79)	6.4e-010	1	K.
15230	752.3331	1502.6516	1502.6524	-0.0008	0	(22)	0.00034	1	K.
15231	752.3331	1502.6517	1502.6524	-0.0007	0	(37)	9.5e-006	1	K.
15233	752.3331	1502.6517	1502.6524	-0.0007	0	(23)	0.00028	1	K.
15234	752.3332	1502.6518	1502.6524	-0.0006	0	(32)	3.3e-005	1	K.
15235	752.3333	1502.6520	1502.6524	-0.0005	0	(69)	6.1e-009	1	K.
15237	752.3333	1502.6521	1502.6524	-0.0004	0	(48)	7.9e-007	1	K.
15239	752.3335	1502.6524	1502.6524	0.0000	0	110	5.4e-013	1	K.
15241	752.3336	1502.6526	1502.6524	0.0001	0	(53)	2.6e-007	1	K.
15242	752.3336	1502.6527	1502.6524	0.0002	0	(83)	2.6e-010	1	K.
15245	752.3337	1502.6528	1502.6524	0.0004	0	(64)	1.8e-008	1	K.
15248	752.3339	1502.6532	1502.6524	0.0007	0	(96)	1.2e-011	1	K.
15249	752.3339	1502.6532	1502.6524	0.0007	0	(21)	0.00041	1	K.
15250	752.3339	1502.6533	1502.6524	0.0009	0	(25)	0.00015	1	K.
15251	752.3339	1502.6533	1502.6524	0.0009	0	(33)	2.6e-005	1	K.
15252	752.3340	1502.6534	1502.6524	0.0010	0	(64)	1.9e-008	1	K.
15253	752.3340	1502.6534	1502.6524	0.0010	0	(73)	2.5e-009	1	K.
15254	752.3340	1502.6535	1502.6524	0.0011	0	(70)	5e-009	1	K.
15258	752.3342	1502.6539	1502.6524	0.0015	0	(69)	6.7e-009	1	K.
15261	752.3344	1502.6542	1502.6524	0.0017	0	(47)	1.1e-006	1	K.
15263	752.3345	1502.6544	1502.6524	0.0020	0	(28)	7.7e-005	1	K.
15264	752.3345	1502.6544	1502.6524	0.0020	0	(29)	5.9e-005	1	K.
15266	752.3346	1502.6546	1502.6524	0.0022	0	(72)	3.3e-009	1	K.
15273	752.3352	1502.6559	1502.6524	0.0034	0	(52)	3.4e-007	1	K.
15498	511.9471	1532.8193	1532.8198	-0.0005	1	(23)	0.00023	1	K.
15500	767.4171	1532.8197	1532.8198	-0.0001	1	27	0.0001	1	K.
15501	511.9472	1532.8199	1532.8198	0.0001	1	(27)	0.00011	1	K.
15571	514.9348	1541.7824	1541.7837	-0.0013	1	55	1.6e-007	1	K.
15573	514.9349	1541.7830	1541.7837	-0.0008	1	(45)	1.7e-006	1	K.
15574	514.9349	1541.7830	1541.7837	-0.0008	1	(26)	0.00014	1	K.
15576	514.9354	1541.7843	1541.7837	0.0005	1	(48)	7.4e-007	1	K.
15579	514.9358	1541.7855	1541.7837	0.0018	1	(22)	0.00035	1	K.
18807	653.6619	1957.9640	1957.9632	0.0008	1	(44)	1.8e-006	1	K.
18808	653.6620	1957.9643	1957.9632	0.0011	1	54	2.2e-007	1	K.
18813	653.6623	1957.9650	1957.9632	0.0019	1	(37)	1.1e-005	1	K.
18818	653.6625	1957.9656	1957.9632	0.0024	1	(21)	0.00036	1	K.
18943	658.6402	1972.8988	1972.8988	-0.0001	0	47	1.1e-006	1	K.
18945	987.4581	1972.9017	1972.8988	0.0029	0	(22)	0.00032	1	K.
18947	987.4585	1972.9024	1972.8988	0.0036	0	(27)	9.1e-005	1	K.
19897	744.0243	2229.0510	2229.0524	-0.0013	1	(35)	1.7e-005	1	K.
19899	744.0250	2229.0532	2229.0524	0.0009	1	36	1.2e-005	1	K.
20472	813.0899	2436.2479	2436.2535	-0.0057	0	(40)	9e-006	1	K.
20474	813.0919	2436.2539	2436.2535	0.0004	0	(40)	1e-005	1	K.
20475	813.0928	2436.2565	2436.2535	0.0030	0	(26)	0.00026	1	K.
20476	1219.1359	2436.2572	2436.2535	0.0036	0	53	4.8e-007	1	K.

20682	856.1031	2565.2876	2565.2858	0.0018	0	34	2e-005	1	R.
20716	861.4332	2581.2779	2581.2807	-0.0029	0	(31)	3.9e-005	1	R.

2. SP7full **Mass:** 57161 **Score:** 2

Check to include this hit in error tolerant search or archive report

Query	Observed	Mr(expt)	Mr(calc)	Delta	Miss	Score	Expect	Rank	Pep
10224	466.6852	931.3559	931.3559	0.0000	0	23	0.0042	1	K.D
17727	879.8939	1757.7733	1757.7744	-0.0011	1	32	9.9e-005	1	R.S
17728	586.9324	1757.7753	1757.7744	0.0009	1	(30)	0.00014	1	R.S
17902	596.9357	1787.7854	1787.7849	0.0004	1	43	5.3e-006	1	R.S
17903	894.9001	1787.7856	1787.7849	0.0007	1	(37)	2.2e-005	1	R.S
18174	923.3606	1844.7066	1844.7013	0.0054	1	28	0.00032	1	K.D
18329	625.9119	1874.7140	1874.7118	0.0021	1	25	0.0016	1	K.D
18330	938.3656	1874.7167	1874.7118	0.0048	1	36	0.00013	1	K.D

Peptide matches not assigned to protein hits: (no details means no match)

Query	Observed	Mr(expt)	Mr(calc)	Delta	Miss	Score	Expect	Rank	Pep
15236	752.3333	1502.6520	1502.6524	-0.0005	0	20	0.00051	1	FS\
14994	739.3860	1476.7575	1476.7572	0.0004	1	20	0.00052	1	AE\
15272	752.3351	1502.6556	1502.6524	0.0032	0	20	0.00054	1	FS\
9028	752.3300	1502.6454	1502.6524	-0.0070	0	20	0.00055	1	FS\
13901	674.3335	1346.6524	1346.6506	0.0018	1	20	0.00055	1	TII
9244	395.6819	789.3492	789.3479	0.0013	0	19	0.00057	1	YPI
14996	493.2599	1476.7580	1476.7572	0.0008	1	19	0.0006	1	AE\
13213	641.7904	1281.5663	1281.5659	0.0004	0	19	0.00062	1	SAI
15277	752.3359	1502.6573	1502.6524	0.0049	0	19	0.00062	1	FS\
13192	641.7892	1281.5638	1281.5659	-0.0021	0	19	0.00063	1	SAI
15502	511.9473	1532.8201	1532.8198	0.0003	1	19	0.00064	1	FE(
15267	752.3346	1502.6546	1502.6524	0.0022	0	19	0.00068	1	FS\
8346	633.7900	1265.5654	1265.5710	-0.0055	0	19	0.0007	1	SAI
13883	449.8907	1346.6504	1346.6506	-0.0002	1	18	0.00075	1	TII
13199	641.7899	1281.5652	1281.5659	-0.0007	0	18	0.00076	1	SAI
13235	641.7908	1281.5670	1281.5659	0.0011	0	18	0.00082	1	SAI
18815	653.6624	1957.9652	1957.9632	0.0020	1	18	0.00088	1	FS\
18814	653.6623	1957.9650	1957.9632	0.0019	1	18	0.00089	1	FS\
13231	641.7907	1281.5669	1281.5659	0.0010	0	17	0.0009	1	SAI
13889	674.3328	1346.6510	1346.6506	0.0004	1	17	0.0009	1	TII
13208	641.7903	1281.5660	1281.5659	0.0001	0	17	0.0009	1	SAI
13222	641.7906	1281.5666	1281.5659	0.0007	0	17	0.00092	1	SAI
13210	641.7903	1281.5661	1281.5659	0.0002	0	17	0.00098	1	SAI
19089	663.9727	1988.8963	1988.8938	0.0026	0	17	0.001	1	LE\
13238	641.7908	1281.5670	1281.5659	0.0011	0	17	0.001	1	SAI
15580	771.9004	1541.7862	1541.7837	0.0025	1	17	0.001	1	GII
13215	641.7905	1281.5664	1281.5659	0.0005	0	16	0.0012	1	SAI
13251	641.7915	1281.5685	1281.5659	0.0026	0	16	0.0012	1	SAI

13888	449.8909	1346.6509	1346.6506	0.0003	1	16	0.0012	1	TII
1345	320.6700	639.3254	639.3340	-0.0086	1	16	0.011	1	SSY
18806	653.6618	1957.9636	1957.9632	0.0004	1	16	0.0013	1	FSV
13188	641.7889	1281.5633	1281.5659	-0.0026	0	16	0.0013	1	SAI
15269	752.3348	1502.6550	1502.6524	0.0026	0	16	0.0013	1	FSV
13200	641.7899	1281.5653	1281.5659	-0.0006	0	16	0.0013	1	SAI
13225	641.7906	1281.5666	1281.5659	0.0007	0	16	0.0014	1	SAI
15575	771.8989	1541.7832	1541.7837	-0.0006	1	15	0.0015	1	GII
13221	641.7905	1281.5665	1281.5659	0.0006	0	15	0.0016	1	SAI
13185	641.7881	1281.5616	1281.5659	-0.0043	0	15	0.0017	1	SAI
13212	641.7904	1281.5663	1281.5659	0.0004	0	15	0.0017	1	SAI
11147	525.7643	1049.5140	1049.5141	-0.0001	0	15	0.0017	1	FE(
15572	514.9348	1541.7826	1541.7837	-0.0011	1	15	0.0018	1	GII
18811	979.9897	1957.9648	1957.9632	0.0016	1	15	0.0018	1	FSV
19898	744.0248	2229.0525	2229.0524	0.0001	1	15	0.0018	1	LEY
13189	641.7889	1281.5633	1281.5659	-0.0026	0	14	0.0018	1	SAI
15497	511.9460	1532.8162	1532.8198	-0.0036	1	14	0.0018	1	FE(
20473	813.0909	2436.2508	2436.2535	-0.0027	0	14	0.0037	1	GEI
18804	653.6614	1957.9623	1957.9632	-0.0009	1	14	0.0019	1	FSV
13014	633.7936	1265.5726	1265.5710	0.0016	0	14	0.0019	1	SAI
15003	493.2601	1476.7586	1476.7572	0.0014	1	14	0.0019	1	AEV
16272	535.5623	1603.6651	1603.6638	0.0014	1	14	0.006	1	SAS
13216	641.7905	1281.5664	1281.5659	0.0005	0	14	0.0021	1	SAI
13246	641.7911	1281.5676	1281.5659	0.0017	0	14	0.0021	1	SAI
13881	449.8907	1346.6502	1346.6506	-0.0004	1	14	0.0021	1	TII
19093	663.9733	1988.8980	1988.8938	0.0042	0	14	0.0022	1	LEY
15226	752.3325	1502.6505	1502.6524	-0.0019	0	13	0.0022	1	FSV
15240	752.3335	1502.6524	1502.6524	0.0000	0	13	0.0022	1	FSV
13227	641.7906	1281.5667	1281.5659	0.0008	0	13	0.0023	1	SAI
13244	641.7910	1281.5674	1281.5659	0.0015	0	13	0.0023	1	SAI
1958	328.1900	654.3654	654.3741	-0.0086	0	13	0.0047	1	TII
13204	641.7901	1281.5656	1281.5659	-0.0003	0	13	0.0024	1	SAI
17899	596.9318	1787.7735	1787.7849	-0.0115	1	13	0.0048	1	SAI
15244	752.3336	1502.6527	1502.6524	0.0002	0	13	0.0024	1	FSV
14997	493.2599	1476.7580	1476.7572	0.0008	1	13	0.0025	1	AEV
13190	641.7890	1281.5635	1281.5659	-0.0025	0	13	0.0027	1	SAI
15274	752.3353	1502.6561	1502.6524	0.0037	0	12	0.0029	1	FSV
8026	579.3100	578.3027	578.3064	-0.0037	0	12	0.003	1	GII
17842	592.2423	1773.7051	1773.7005	0.0046	1	12	0.0031	1	DDY
20748	866.7660	2597.2761	2597.2756	0.0005	0	12	0.0033	1	DHI
2562	334.6800	667.3454	667.3401	0.0053	1	12	0.034	1	SSY
13187	641.7888	1281.5630	1281.5659	-0.0029	0	12	0.0035	1	SAI
13193	641.7894	1281.5643	1281.5659	-0.0016	0	11	0.0036	1	SAI
15002	739.3865	1476.7584	1476.7572	0.0012	1	11	0.0036	1	AEV
13016	633.7941	1265.5736	1265.5710	0.0026	0	11	0.0038	1	SAI
13240	641.7908	1281.5671	1281.5659	0.0012	0	11	0.0038	1	SAI
13229	641.7906	1281.5667	1281.5659	0.0008	0	11	0.0039	1	SAI
13018	633.7949	1265.5753	1265.5710	0.0043	0	11	0.0039	1	SAI
18173	615.9085	1844.7037	1844.7013	0.0024	1	11	0.016	1	DDI

19088	995.4553	1988.8961	1988.8938	0.0023	0	11	0.0042	1	LE[illegible]
15578	771.8999	1541.7853	1541.7837	0.0015	1	11	0.0044	1	GI[illegible]
13197	641.7897	1281.5649	1281.5659	-0.0010	0	11	0.0045	1	SA[illegible]
13891	674.3328	1346.6511	1346.6506	0.0005	1	10	0.0045	1	TI[illegible]
15503	767.4177	1532.8209	1532.8198	0.0011	1	10	0.0046	1	FE[illegible]
13198	641.7898	1281.5650	1281.5659	-0.0009	0	10	0.0047	1	SA[illegible]
13223	641.7906	1281.5666	1281.5659	0.0007	0	10	0.0047	1	SA[illegible]
19087	663.9725	1988.8958	1988.8938	0.0020	0	10	0.0047	1	LE[illegible]
18819	653.6626	1957.9660	1957.9632	0.0028	1	10	0.0048	1	FS[illegible]
15271	752.3349	1502.6552	1502.6524	0.0028	0	10	0.005	1	FS[illegible]
1957	328.1900	654.3654	654.3741	-0.0086	0	10	0.01	1	TI[illegible]
8471	328.1941	654.3736	654.3741	-0.0005	0	10	0.0052	1	TI[illegible]
13903	674.3336	1346.6526	1346.6506	0.0019	1	10	0.0052	1	TI[illegible]
18816	653.6624	1957.9654	1957.9632	0.0022	1	10	0.0052	1	FS[illegible]
8790	352.2085	702.4024	702.4024	0.0000	1	10	0.0053	1	TR[illegible]
9062	379.6932	757.3719	757.3718	0.0000	1	10	0.0054	1	DP[illegible]
15577	514.9355	1541.7848	1541.7837	0.0011	1	10	0.0055	1	GI[illegible]
6903	449.8900	1346.6482	1346.6506	-0.0024	1	9	0.0058	1	TI[illegible]
9242	395.6811	789.3476	789.3479	-0.0004	0	9	0.0059	1	YP[illegible]
17904	596.9363	1787.7872	1787.7849	0.0022	1	9	0.012	1	SA[illegible]
13641	660.8164	1319.6183	1319.6429	-0.0247	1	9	0.0062	1	ET[illegible]
7460	511.9600	1532.8582	1532.8198	0.0384	1	9	0.0063	1	FE[illegible]
13194	641.7896	1281.5646	1281.5659	-0.0014	0	9	0.0067	1	SA[illegible]
18805	653.6616	1957.9629	1957.9632	-0.0003	1	9	0.0068	1	FS[illegible]
1346	320.6700	639.3254	639.3340	-0.0086	1	9	0.062	1	SS[illegible]
18946	658.6413	1972.9021	1972.8988	0.0032	0	9	0.0071	1	LE[illegible]
9998	451.2588	900.5030	900.4665	0.0366	1	8	0.0076	1	RL[illegible]
9313	802.8400	1603.6654	1603.6638	0.0017	1	8	0.024	1	SA[illegible]
18821	653.6633	1957.9682	1957.9632	0.0050	1	8	0.0086	1	FS[illegible]
13191	641.7891	1281.5636	1281.5659	-0.0023	0	8	0.0086	1	SA[illegible]
13233	641.7907	1281.5669	1281.5659	0.0010	0	8	0.0089	1	SA[illegible]
13249	641.7913	1281.5680	1281.5659	0.0021	0	7	0.0091	1	SA[illegible]
13017	633.7941	1265.5737	1265.5710	0.0027	0	7	0.0095	1	SA[illegible]
15262	752.3344	1502.6543	1502.6524	0.0018	0	7	0.0095	1	FS[illegible]
13882	449.8907	1346.6502	1346.6506	-0.0004	1	7	0.0097	1	TI[illegible]
11357	538.2327	1074.4509	1074.4506	0.0003	0	7	0.019	1	DS[illegible]
15504	511.9477	1532.8213	1532.8198	0.0015	1	7	0.011	1	FE[illegible]
18822	653.6636	1957.9691	1957.9632	0.0059	1	7	0.011	1	FS[illegible]
13885	449.8908	1346.6505	1346.6506	-0.0001	1	7	0.011	1	TI[illegible]
19900	744.0251	2229.0534	2229.0524	0.0010	1	6	0.011	1	LE[illegible]
10549	323.5276	967.5610	967.4359	0.1251	1	6	0.011	1	DD[illegible]
13201	641.7900	1281.5654	1281.5659	-0.0005	0	6	0.012	1	SA[illegible]
17729	586.9325	1757.7757	1757.7744	0.0013	1	6	0.035	1	SA[illegible]
11356	538.2324	1074.4502	1074.4506	-0.0004	0	6	0.024	1	DS[illegible]
8591	334.6771	667.3397	667.3401	-0.0005	1	6	0.062	1	SS[illegible]
17900	596.9342	1787.7808	1787.7849	-0.0042	1	6	0.025	1	SA[illegible]
15268	752.3348	1502.6550	1502.6524	0.0026	0	6	0.013	1	FS[illegible]
18812	653.6622	1957.9649	1957.9632	0.0017	1	6	0.013	1	FS[illegible]

10121	307.1833	918.5281	918.5286	-0.0005	1	6	0.014	1	NGI
18820	979.9909	1957.9673	1957.9632	0.0041	1	5	0.014	1	FSV
19942	749.3554	2245.0444	2245.0473	-0.0029	1	5	0.015	1	LEY
15275	752.3356	1502.6567	1502.6524	0.0043	0	5	0.015	1	FSV
15499	511.9471	1532.8194	1532.8198	-0.0004	1	5	0.016	1	FEG
13878	449.8904	1346.6493	1346.6506	-0.0013	1	5	0.016	1	TII
7056	466.6800	931.3454	931.3559	-0.0105	0	5	0.28	1	DDI
10122	460.2721	918.5296	918.5286	0.0009	1	5	0.017	1	NGI
13184	641.7877	1281.5608	1281.5659	-0.0051	0	5	0.017	1	SAI
13250	641.7913	1281.5680	1281.5659	0.0021	0	5	0.017	1	SAI
19943	749.3569	2245.0488	2245.0473	0.0015	1	5	0.017	1	LEY
15243	752.3336	1502.6527	1502.6524	0.0002	0	5	0.018	1	FSV
15265	752.3345	1502.6545	1502.6524	0.0021	0	5	0.018	1	FSV
8202	301.6423	601.2701	601.2707	-0.0007	0	4	0.018	1	DPI
15278	752.3365	1502.6585	1502.6524	0.0061	0	4	0.019	1	FSV
15276	752.3358	1502.6571	1502.6524	0.0046	0	4	0.019	1	FSV
4017	379.6900	757.3654	757.3718	-0.0064	1	4	0.019	1	DPI
341	306.6700	611.3254	611.3279	-0.0024	1	4	0.23	1	KSS
8469	655.3800	654.3727	654.3741	-0.0013	0	4	0.02	1	TII
18809	653.6620	1957.9643	1957.9632	0.0011	1	4	0.021	1	FSV
13015	633.7938	1265.5731	1265.5710	0.0021	0	4	0.021	1	SAI
4993	426.5500	1276.6282	1276.7139	-0.0857	1	4	0.022	1	SLY
13202	641.7900	1281.5655	1281.5659	-0.0004	0	4	0.022	1	SAI
15223	752.3320	1502.6495	1502.6524	-0.0029	0	3	0.022	1	FSV
15222	752.3315	1502.6485	1502.6524	-0.0039	0	3	0.023	1	FSV
13224	641.7906	1281.5666	1281.5659	0.0007	0	3	0.024	1	SAI
13011	633.7931	1265.5716	1265.5710	0.0006	0	3	0.024	1	SAI
18817	653.6624	1957.9654	1957.9632	0.0022	1	3	0.025	1	FSV
19091	663.9730	1988.8972	1988.8938	0.0035	0	3	0.025	1	LEY
15270	752.3348	1502.6551	1502.6524	0.0027	0	3	0.025	1	FSV
15221	752.3308	1502.6471	1502.6524	-0.0054	0	3	0.026	1	FSV
19086	663.9693	1988.8861	1988.8938	-0.0077	0	3	0.027	1	LEY
14998	493.2599	1476.7580	1476.7572	0.0008	1	3	0.027	1	AEV
15228	752.3327	1502.6509	1502.6524	-0.0016	0	3	0.027	1	FSV
19094	663.9738	1988.8996	1988.8938	0.0059	0	3	0.028	1	LEY
13183	641.7869	1281.5593	1281.5659	-0.0066	0	2	0.029	1	SAI
21117	988.8420	2963.5043	2963.3067	0.1976	1	2	0.03	1	FII
6444	439.1900	876.3654	876.4301	-0.0646	0	2	0.03	1	VSS
15232	752.3331	1502.6517	1502.6524	-0.0007	0	2	0.03	1	FSV
5964	432.7500	863.4854	863.4422	0.0432	0	2	0.03	1	LST
19095	663.9739	1988.9000	1988.8938	0.0062	0	2	0.032	1	LEY
20429	807.7380	2420.1921	2420.2845	-0.0924	1	2	0.033	1	VVI
13234	641.7907	1281.5669	1281.5659	0.0010	0	2	0.033	1	SAI
15257	752.3342	1502.6538	1502.6524	0.0013	0	2	0.033	1	FSV
19092	663.9731	1988.8974	1988.8938	0.0037	0	1	0.036	1	LEY
18823	979.9918	1957.9691	1957.9632	0.0059	1	1	0.036	1	FSV
7873	551.8100	1101.6054	1101.5740	0.0315	0	1	0.037	1	TEG
19090	663.9727	1988.8963	1988.8938	0.0026	0	1	0.038	1	LEY

☑										
☑	15229	752.3328	1502.6511	1502.6524	-0.0013	0	1	0.038	1	FS\
☑	4557	407.2700	812.5254	812.4180	0.1074	1	1	0.039	1	NY(
☑	15283	752.4103	1502.8061	1502.6524	0.1537	0	1	0.04	1	FS\
☑	9488	414.2189	826.4233	826.3895	0.0338	0	1	0.041	1	FGI
☑	13203	641.7900	1281.5655	1281.5659	-0.0004	0	1	0.041	1	SAI
☑	15246	752.3337	1502.6529	1502.6524	0.0005	0	1	0.042	1	FS\
☑	14993	493.2597	1476.7573	1476.7572	0.0001	1	1	0.042	1	AE\
☑	20717	861.4342	2581.2808	2581.2807	0.0001	0	1	0.043	1	DHI
☑	15506	767.4187	1532.8229	1532.8198	0.0031	1	1	0.043	1	FE(
☑	7250	493.2600	1476.7582	1476.7572	0.0010	1	1	0.044	1	AE\
☑	13196	641.7897	1281.5648	1281.5659	-0.0011	0	0	0.045	1	SAI
☑	19941	749.3554	2245.0444	2245.0473	-0.0029	1	0	0.046	1	LE\
☑	10551	323.5277	967.5613	967.4359	0.1254	1	0	0.047	1	DD(
☑	15255	752.3341	1502.6537	1502.6524	0.0012	0	0	0.047	1	FS\
☑	6464	440.9500	1319.8282	1319.6429	0.1853	1	0	0.049	1	ET(
☑	13206	641.7902	1281.5658	1281.5659	-0.0001	0	0	0.049	1	SAI
☑	1	300.0600	299.0527							
☑	2	300.2000	299.1927							
☑	3	300.2000	299.1927							
☑	4	300.2000	299.1927							
☑	5	300.2000	299.1927							
☑	6	300.2000	299.1927							
☑	7	300.2000	299.1927							
☑	8	300.2000	299.1927							
☑	9	300.2100	299.2027							
☑	10	300.2900	299.2827							
☑	11	301.0300	300.0227							
☑	12	301.0300	300.0227							
☑	13	301.0300	300.0227							
☑	14	301.0300	300.0227							
☑	15	301.0300	300.0227							
☑	16	301.0300	300.0227							
☑	17	301.0300	300.0227							
☑	18	301.1400	300.1327							
☑	19	301.1400	300.1327							
☑	20	301.1400	300.1327							
☑	21	301.1400	300.1327							
☑	22	301.1400	300.1327							
☑	23	301.1400	300.1327							
☑	24	301.1400	300.1327							
☑	25	301.1400	300.1327							
☑	26	301.1400	300.1327							
☑	27	301.1400	300.1327							
☑	28	301.1400	300.1327							
☑	29	301.1400	300.1327							
☑	30	301.1400	300.1327							
☑	31	301.1400	300.1327							
	32	301.1400	300.1327							

☑			
☑	33	301.1400	300.1327
☑	34	301.1400	300.1327
☑	35	301.1400	300.1327
☑	36	301.1400	300.1327
☑	37	301.1400	300.1327
☑	38	301.1400	300.1327
☑	39	301.1400	300.1327
☑	40	301.1400	300.1327
☑	41	301.1400	300.1327
☑	42	301.1400	300.1327
☑	43	301.1400	300.1327
☑	44	301.1400	300.1327
☑	45	301.1400	300.1327
☑	46	301.1400	300.1327
☑	47	301.1400	300.1327
☑	48	301.1400	300.1327
☑	49	301.1400	300.1327
☑	50	301.1400	300.1327
☑	51	301.1400	300.1327
☑	52	301.1400	300.1327
☑	53	301.1400	300.1327
☑	54	301.1400	300.1327
☑	55	301.1400	300.1327
☑	56	301.1400	300.1327
☑	57	301.1400	300.1327
☑	58	301.1400	300.1327
☑	59	301.1400	300.1327
☑	60	301.1400	300.1327
☑	61	301.1400	300.1327
☑	62	301.1400	300.1327
☑	63	301.1400	300.1327
☑	64	301.1400	300.1327
☑	65	301.1400	300.1327
☑	66	301.1400	300.1327
☑	67	301.1400	300.1327
☑	68	301.1400	300.1327
☑	69	301.1400	300.1327
☑	70	301.1400	300.1327
☑	71	301.1400	300.1327
☑	72	301.1400	300.1327
☑	73	301.1400	300.1327
☑	74	301.1400	300.1327
☑	75	301.1400	300.1327
☑	76	301.1400	300.1327
☑	77	301.1400	300.1327
☑	78	301.1400	300.1327
☑	79	301.1400	300.1327
	80	301.1400	300.1327

☑			
☑	81	301.1400	300.1327
☑	82	301.1400	300.1327
☑	83	301.1400	300.1327
☑	84	301.1400	300.1327
☑	85	301.1400	300.1327
☑	86	301.1400	300.1327
☑	87	301.1400	300.1327
☑	88	301.1400	300.1327
☑	89	301.1400	300.1327
☑	90	301.1400	300.1327
☑	91	301.1400	300.1327
☑	92	301.1400	300.1327
☑	93	301.1400	300.1327
☑	94	301.1400	300.1327
☑	95	301.1400	300.1327
☑	96	301.1400	300.1327
☑	97	301.1400	300.1327
☑	98	301.1400	300.1327
☑	99	301.1400	300.1327
☑	100	301.1400	300.1327
☑	101	301.1400	300.1327
☑	102	301.1400	300.1327
☑	103	301.1400	300.1327
☑	104	301.1400	300.1327
☑	105	301.1400	300.1327
☑	106	301.1400	300.1327
☑	107	301.1400	300.1327
☑	108	301.1400	300.1327
☑	109	301.1400	300.1327
☑	110	301.1400	300.1327
☑	111	301.1400	300.1327
☑	112	301.1400	300.1327
☑	113	301.1400	300.1327
☑	114	301.1400	300.1327
☑	115	301.1400	300.1327
☑	116	301.1400	300.1327
☑	117	301.1400	300.1327
☑	118	301.1400	300.1327
☑	119	301.1400	300.1327
☑	120	301.1400	300.1327
☑	121	301.1400	300.1327
☑	122	301.1400	300.1327
☑	123	301.1400	300.1327
☑	124	301.1400	300.1327
☑	125	301.1400	300.1327
☑	126	301.1400	300.1327
☑	127	301.1400	300.1327
	128	301.1400	300.1327
