# Supplementary material for: Alternative splicing regulation in plants by SP7-like effectors from symbiotic arbuscular mycorrhizal fungi
Source: Nat Commun. 2024 Aug 19;15:7107. doi: 10.1038/s41467-024-51512-5 (PMC11333574; doi:10.1038/s41467-024-51512-5)
Supplement: Supplementary file 10 — Source Data [file 41467_2024_51512_MOESM10_ESM.zip › Requena_8071-2_ProteinSummary (RiSP7ΓêåSP).pdf]

Mascot Search Results

```
User                    :
Email                   :
Search title            : 8071-2
MS data file            : 8071_2.mgf
Database                : TopLab_8071 8071_20160923 (76598 sequences; 22305840 residues)
Timestamp               : 1 Dec 2016 at 13:51:36 GMT
Enzyme                  : Trypsin
Fixed modifications     : Carbamidomethyl (C)
Variable modifications  : Oxidation (M)
Mass values             : Monoisotopic
Protein Mass            : Unrestricted
Peptide Mass Tolerance  : ± 50 ppm
Fragment Mass Tolerance: ± 0.6 Da
Max Missed Cleavages    : 1
Instrument type         : Default
Number of queries       : 21295
Protein hits            : gi|297262447|ref|XP_001098182.2| gi|297262447|ref|XP_001098182.2| PREDICTED: keratin, type II cytoskeletal 1-like isoform 6 [Macaca mulatta]
                          NbS00031648g0011.1               NbS00031648g0011.1 protein AED:0.23 eAED:0.23 QI:280|1|1|1|0.95|0.91|23|300|916; (*GB) gi|225465253|ref|XP_002268311.1| (e_value=0.0) PREDICTED:
                          gi|28317|emb|CAA32649.1|         gi|28317|emb|CAA32649.1| unnamed protein product [Homo sapiens]
                          gi|291410763|ref|XP_002721657.1| gi|291410763|ref|XP_002721657.1| PREDICTED: histone cluster 1, H2ag-like [Oryctolagus cuniculus]
                          gi|148727309|ref|NP_001092039.1| gi|148727309|ref|NP_001092039.1| keratin, type II cytoskeletal 2 epidermal [Pan troglodytes]
                          NbS00000666g0001.1               NbS00000666g0001.1 protein AED:0.15 eAED:0.15 QI:0|-1|0|1|-1|1|1|0|145; (*GB) gi|82400146|gb|ABB72812.1| (e_value=5e-61) histone H2B-like protein
                          NbS00004898g0002.1               NbS00004898g0002.1 protein AED:0.00 eAED:0.00 QI:0|-1|0|1|-1|1|1|0|103; (*GB) gi|195617694|gb|ACG30677.1| (e_value=1e-51) histone H4 [Zea mays];;
                          NbS00056353g0008.1               NbS00056353g0008.1 protein AED:0.25 eAED:0.25 QI:0|0.92|0.85|1|0.92|0.92|14|0|554; (*SWP) sp|Q9FNX5|DRP1E_ARATH (e_value=0.0) Dynamin-related prc
                          NbS00000987g0004.1               NbS00000987g0004.1 protein AED:0.21 eAED:0.21 QI:0|-1|0|1|-1|1|1|0|266; (*GB) gi|3036948|dbj|BAA25392.1| (e_value=0.0) light harvesting chlorophy
                          NbS00000867g0011.1               NbS00000867g0011.1 protein AED:0.34 eAED:0.34 QI:171|0.9|0.90|1|0.7|0.63|11|450|270; (*GB) gi|359492084|ref|XP_002284740.2| (e_value=1e-70) PREDI
                          NbS00054358g0003.1               NbS00054358g0003.1 protein AED:0.12 eAED:0.14 QI:177|0.91|0.84|1|0.91|0.84|13|0|563; (*GB) gi|359489241|ref|XP_003633899.1| (e_value=0.0) PREDICT
                          NbS00000471g0009.1               NbS00000471g0009.1 protein AED:0.15 eAED:0.15 QI:0|0|0.33|1|1|1|3|0|301; (*GB) gi|115802|sp|P27494.1|CB23_TOBAC (e_value=0.0) RecName: Full=Chlor
                          NbS00017066g0003.1               NbS00017066g0003.1 protein AED:0.01 eAED:0.01 QI:0|-1|0|1|-1|1|1|0|208; (*GB) gi|222051768|dbj|BAH15357.1| (e_value=3e-127) germin like protein [
                          NbS00021492g0001.1               NbS00021492g0001.1 protein AED:0.22 eAED:0.25 QI:0|0.70|0.72|0.94|0.94|0.88|18|255|639; (*SWP) sp|Q39828|SDL5A_SOYBN (e_value=0.0) Dynamin-relate
                          NbS00001169g0153.1               NbS00001169g0153.1 protein AED:0.25 eAED:0.25 QI:0|0.5|0.4|1|1|1|5|0|341
                          NbS00010009g0002.1               NbS00010009g0002.1 protein AED:0.20 eAED:0.20 QI:0|1|1|1|1|1|21|408|1058; (*SWP) sp|O04379|AGO1_ARATH (e_value=0.0) Protein argonaute 1 OS=Arabid
                          NbS00033686g0006.1               NbS00033686g0006.1 protein AED:0.16 eAED:0.16 QI:197|1|1|1|1|1|15|214|623; (*GB) gi|225453246|ref|XP_002265511.1| (e_value=0.0) PREDICTED: dynami
                          NbS00014977g0008.1               NbS00014977g0008.1 protein AED:0.16 eAED:0.16 QI:16|0.95|0.90|1|0.95|0.90|22|432|1125; (*GB) gi|84688908|gb|ABC61503.1| (e_value=0.0) AGO1-2, par
                          NbS00010295g0007.1               NbS00010295g0007.1 protein AED:0.12 eAED:0.12 QI:0|0|0|0.5|1|1|2|0|119; (*SWP) sp|P69569|RBL_EUPES (e_value=5e-22) Ribulose bisphosphate carboxyl
                          NbS00000681g0006.1               NbS00000681g0006.1 protein AED:0.29 eAED:0.30 QI:89|0.75|0.88|1|0.75|0.77|9|282|301; (*GB) gi|358248282|ref|NP_001239854.1| (e_value=7e-62) uncha
                          NbS00004085g0015.1               NbS00004085g0015.1 protein AED:0.29 eAED:0.29 QI:92|0.25|0.2|1|1|1|5|0|225; (*GB) gi|110377793|gb|ABG73417.1| (e_value=7e-87) chloroplast pigment
                          NbS00001942g0006.1               NbS00001942g0006.1 protein AED:0.00 eAED:0.00 QI:75|1|1|1|1|1|2|198|151; (*GB) gi|351725389|ref|NP_001235555.1| (e_value=1e-63) uncharacterized p
                          NbS00006820g0015.1               NbS00006820g0015.1 protein AED:0.07 eAED:0.08 QI:0|1|0.66|1|1|1|3|0|273; (*GB) gi|226872|prf||1609235A (e_value=1e-149) chlorophyll a/b binding p
                          NbS00002621g0212.1               NbS00002621g0212.1 protein AED:0.19 eAED:0.19 QI:122|0.8|1|1|1|1|6|306|255
                          NbS00007993g0305.1               NbS00007993g0305.1 protein AED:0.00 eAED:0.00 QI:0|-1|0|1|-1|1|1|0|261
                          NbS00010743g0014.1               NbS00010743g0014.1 protein AED:0.24 eAED:0.24 QI:0|1|1|1|1|1|8|290|288; (*GB) gi|77416949|gb|ABA81870.1| (e_value=2e-155) unknown [Solanum tuberc
                          NbS00001988g0005.1               NbS00001988g0005.1 protein AED:0.30 eAED:0.30 QI:0|1|0.83|1|1|1|6|0|610; (*GB) gi|225441549|ref|XP_002281113.1| (e_value=0.0) PREDICTED: DEAD-box
                          NbS00012584g0001.1               NbS00012584g0001.1 protein AED:0.12 eAED:0.12 QI:0|-1|0|1|-1|1|1|0|370; (*GB) gi|231610|sp|P29790.1|ATPG_TOBAC (e_value=0.0) RecName: Full=ATP sy
                          NbS00017935g0003.1               NbS00017935g0003.1 protein AED:0.00 eAED:0.00 QI:240|1|0.5|1|1|1|2|0|284; (*SWP) sp|Q9XF88|CB4B_ARATH (e_value=4e-162) Chlorophyll a-b binding pr
                          NbS00004793g0016.1               NbS00004793g0016.1 protein AED:0.15 eAED:0.27 QI:0|0|0|1|1|1|2|0|286; (*SWP) sp|P27492|CB21_TOBAC (e_value=9e-155) Chlorophyll a-b binding protei
                          NbS00000634g0101.1               NbS00000634g0101.1 protein AED:0.21 eAED:0.21 QI:0|-1|0|1|-1|1|1|0|136
                          NbS00035687g0007.1               NbS00035687g0007.1 protein AED:0.23 eAED:0.23 QI:300|1|0.75|1|1|1|4|0|274; (*GB) gi|84620802|gb|ABC59515.1| (e_value=9e-120) chloroplast photosys
                          NbS00001559g0021.1               NbS00001559g0021.1 protein AED:0.25 eAED:0.31 QI:138|0.5|0.53|1|0.85|0.93|15|300|837; (*GB) gi|356513635|ref|XP_003525517.1| (e_value=0.0) PREDIC
                          NbS00009714g0011.1               NbS00009714g0011.1 protein AED:0.20 eAED:0.20 QI:131|0.83|0.71|1|0.83|0.57|7|463|410; (*SWP) sp|Q40565|RCA2_TOBAC (e_value=0.0) Ribulose bisphosp
                          NbS00025223g0014.1               NbS00025223g0014.1 protein AED:0.37 eAED:0.37 QI:0|1|0.33|1|1|0.66|3|0|136; (*GB) gi|51490663|emb|CAG26902.1| (e_value=3e-63) ALY protein [Nicoti
                          NbS00010583g0001.1               NbS00010583g0001.1 protein AED:0.25 eAED:0.25 QI:0|-1|0|1|-1|1|1|0|210; (*GB) gi|31711507|dbj|BAC77634.1| (e_value=2e-132) 24K germin like protei
                          gi|119581148|gb|EAW60744.1|      gi|119581148|gb|EAW60744.1| keratin 9 (epidermolytic palmoplantar keratoderma) [Homo sapiens]
                          NbS00001849g0017.1               NbS00001849g0017.1 protein AED:0.76 eAED:1.00 QI:0|0|0|0.66|0.5|0.66|3|0|72; (*GB) gi|94466657|emb|CAJ44457.1| (e_value=3e-26) ALY protein [Nicot
                          NbS00029393g0012.1               NbS00029393g0012.1 protein AED:0.08 eAED:0.08 QI:319|0.42|0.37|1|1|1|8|307|444; (*GB) gi|12643757|sp|Q40460.1|RCA1_TOBAC (e_value=0.0) RecName: F
                          NbS00042109g0018.1               NbS00042109g0018.1 protein AED:0.00 eAED:0.00 QI:0|1|0.5|1|1|1|2|0|256; (*SWP) sp|P27524|CB4A_SOLLC (e_value=3e-163) Chlorophyll a-b binding prot
                          NbS00027807g0002.1               NbS00027807g0002.1 protein AED:0.05 eAED:0.05 QI:0|1|0.5|1|0|0|2|953|413; (*GB) gi|68566313|sp|Q40450.2|EFTUA_NICSY (e_value=0.0) RecName: Full=E
                          NbS00002894g0003.1               NbS00002894g0003.1 protein AED:0.02 eAED:0.02 QI:0|1|0.5|1|1|1|2|0|386; (*GB) gi|78191448|gb|ABB29945.1| (e_value=0.0) ADP/ATP translocator-like
                          NbS00007511g0005.1               NbS00007511g0005.1 protein AED:0.26 eAED:0.26 QI:0|0.62|0.55|0.88|1|1|9|0|1084; (*GB) gi|359477631|ref|XP_002274485.2| (e_value=0.0) PREDICTED: u
```

NbS00056669g0006.1 NbS00056669g0006.1 protein AED:0.08 eAED:0.08 QI:167|1|1|1|1|1|5|1743|252; (*SWP) sp|B5FXN8|THOC4_TAEGU (e_value=4e-36) THO complex subunit 4 OS=
NbS00028679g0001.1 NbS00028679g0001.1 protein AED:0.08 eAED:0.08 QI:281|0.88|0.9|1|0.88|0.8|10|299|305; (*GB) gi|255542956|ref|XP_002512541.1| (e_value=0.0) NAD dep
NbS00036785g0006.1 NbS00036785g0006.1 protein AED:0.27 eAED:0.32 QI:267|0.88|0.8|1|0.77|0.6|10|263|336; (*GB) gi|356572914|ref|XP_003554610.1| (e_value=0.0) PREDICT
NbS00030061g0007.1 NbS00030061g0007.1 protein AED:0.13 eAED:0.13 QI:243|1|1|1|0.85|0.75|8|1441|373; (*GB) gi|77416977|gb|ABA81884.1| (e_value=7e-108) nuclear RNA bi
NbS00010663g0016.1 NbS00010663g0016.1 protein AED:0.20 eAED:0.20 QI:7|1|0.88|1|1|1|9|298|622; (*GB) gi|224135925|ref|XP_002322195.1| (e_value=0.0) predicted protein
NbS00024580g0005.1 NbS00024580g0005.1 protein AED:0.21 eAED:0.21 QI:87|0.83|0.85|1|0.83|0.85|7|278|321; (*SWP) sp|Q641W4|RFC2_RAT (e_value=5e-157) Replication facto
NbS00003075g0011.1 NbS00003075g0011.1 protein AED:0.28 eAED:0.28 QI:0|1|0.66|1|1|1|3|0|143; (*GB) gi|2499967|sp|Q41229.1|PSAEB_NICSY (e_value=1e-59) RecName: Full=P
NbS00019623g0001.1 NbS00019623g0001.1 protein AED:0.21 eAED:0.21 QI:0|1|0.5|1|1|1|2|0|447; (*GB) gi|3869088|dbj|BAA34348.1| (e_value=0.0) elongation factor-1 alpha
sp|TRYP_PIG| sp|TRYP_PIG|
NbS00006644g0116.1 NbS00006644g0116.1 protein AED:0.00 eAED:0.00 QI:364|1|1|1|1|1|3|541|1093
NbS00003134g0011.1 NbS00003134g0011.1 protein AED:0.32 eAED:0.33 QI:0|0.75|0.33|0.88|0.75|0.66|9|0|720; (*SWP) sp|P46942|DB10_NICSY (e_value=0.0) ATP-dependent RNA
NbS00003763g0016.1 NbS00003763g0016.1 protein AED:0.10 eAED:0.12 QI:0|0.92|0.8|0.93|1|1|15|0|884; (*GB) gi|225441896|ref|XP_002284404.1| (e_value=0.0) PREDICTED: un
NbS00004956g0015.1 NbS00004956g0015.1 protein AED:0.25 eAED:0.30 QI:0|0|0|1|0|0|2|0|84; (*GB) gi|42718201|gb|AAS38532.1| (e_value=7e-41) ribulose-1,5-bisphosphate c
NbS00001373g0014.1 NbS00001373g0014.1 protein AED:0.25 eAED:0.25 QI:0|0.87|0.64|1|0.75|0.82|17|0|866; (*GB) gi|225431788|ref|XP_002271622.1| (e_value=0.0) PREDICTED
NbS00058945g0001.1 NbS00058945g0001.1 protein AED:0.25 eAED:0.26 QI:0|0.8|0.5|1|1|1|6|0|457; (*TAIR) AT5G58470.2 (e_value=1e-51) | Symbols: TAF15b | TBP-associated
NbS00016433g0017.1 NbS00016433g0017.1 protein AED:0.23 eAED:0.24 QI:195|1|0.9|1|1|1|10|0|371; (*GB) gi|255543841|ref|XP_002512983.1| (e_value=0.0) clathrin binding
NbS00027647g0011.1 NbS00027647g0011.1 protein AED:0.16 eAED:0.17 QI:0|0.9|0.72|1|0.9|0.72|11|0|816; (*GB) gi|297745425|emb|CBI40505.3| (e_value=0.0) unnamed protein
NbS00021832g0023.1 NbS00021832g0023.1 protein AED:0.35 eAED:0.35 QI:0|0|0|1|0|0|2|0|80; (*GB) gi|305671961|gb|ADM63350.1| (e_value=2e-44) photosystem II cp47 protei
NbS00007972g0020.1 NbS00007972g0020.1 protein AED:0.25 eAED:0.25 QI:0|1|0.66|1|1|1|3|0|145; (*GB) gi|407353|dbj|BAA04633.1| (e_value=1e-68) PSI-H precursor [Nicotia
NbS00036430g0009.1 NbS00036430g0009.1 protein AED:0.35 eAED:0.35 QI:0|0|0|1|0|0|4|0|448; (*GB) gi|113170490|ref|YP_717281.1| (e_value=2e-170) Atp1 [Ostreococcus tau
NbS00008911g0002.1 NbS00008911g0002.1 protein AED:0.35 eAED:0.35 QI:460|0.77|0.7|1|1|1|10|229|443; (*GB) gi|327198779|emb|CBL43264.1| (e_value=0.0) glyceraldehyde-3
NbS00001161g0103.1 NbS00001161g0103.1 protein AED:0.03 eAED:0.04 QI:203|0.5|0.66|1|0|0|3|2345|542
gi|297692195|ref|XP_002823453.1| gi|297692195|ref|XP_002823453.1| PREDICTED: LOW QUALITY PROTEIN: ATP synthase subunit beta, mitochondrial-like [Pongo abelii]
NbS00027428g0011.1 NbS00027428g0011.1 protein AED:0.20 eAED:0.20 QI:0|0|0|1|1|1|2|0|723; (*GB) gi|359475106|ref|XP_003631587.1| (e_value=0.0) PREDICTED: DEAD-box AT
NbS00019265g0001.1 NbS00019265g0001.1 protein AED:0.29 eAED:0.34 QI:99|0.6|0.66|1|1|1|6|0|422; (*GB) gi|115765|sp|P10708.1|CB12_SOLLC (e_value=3e-151) RecName: Full
NbS00020769g0006.1 NbS00020769g0006.1 protein AED:0.25 eAED:0.25 QI:307|0.66|1|1|0.66|0.75|4|448|250; (*GB) gi|84620804|gb|ABC59516.1| (e_value=5e-102) chloroplast
NbS00042812g0008.1 NbS00042812g0008.1 protein AED:0.19 eAED:0.19 QI:0|1|1|1|1|1|4|386|180; (*GB) gi|132118|sp|P26573.1|RBS8_NICPL (e_value=3e-119) RecName: Full=Rib
NbC24305910g0003.1 NbC24305910g0003.1 protein AED:0.03 eAED:0.03 QI:0|1|0.5|1|1|1|2|0|101; (*GB) gi|12643758|sp|Q40565.1|RCA2_TOBAC (e_value=6e-65) RecName: Full=Ri
NbS00005125g0015.1 NbS00005125g0015.1 protein AED:0.27 eAED:0.27 QI:600|0.91|0.84|1|0.83|0.69|13|286|356; (*GB) gi|304368145|gb|ADM26718.1| (e_value=0.0) glycolate
NbS00006841g0003.1 NbS00006841g0003.1 protein AED:0.24 eAED:0.24 QI:284|1|1|1|0.66|0.75|4|120|287; (*GB) gi|115291793|gb|ABI93215.1| (e_value=0.0) water channel prc
NbS00016136g0003.1 NbS00016136g0003.1 protein AED:0.24 eAED:0.24 QI:0|0|0|1|1|1|3|0|533; (*GB) gi|392465167|dbj|BAM24707.1| (e_value=0.0) Heat shock protein 70 [Nic
NbS00003826g0005.1 NbS00003826g0005.1 protein AED:0.03 eAED:0.03 QI:80|0.87|0.88|1|0.87|0.88|9|279|511; (*GB) gi|1345684|sp|P49317.1|CATA3_NICPL (e_value=0.0) RecNa
NbS00019305g0027.1 NbS00019305g0027.1 protein AED:0.37 eAED:0.37 QI:0|1|0.88|1|1|1|9|0|556; (*GB) gi|3676296|gb|AAD03392.1| (e_value=0.0) mitochondrial ATPase beta
NbS00009638g0019.1 NbS00009638g0019.1 protein AED:0.11 eAED:0.11 QI:333|1|1|1|1|1|3|176|181; (*GB) gi|132118|sp|P26573.1|RBS8_NICPL (e_value=6e-118) RecName: Full=R
NbS00019862g0010.1 NbS00019862g0010.1 protein AED:0.10 eAED:0.10 QI:0|0.92|1|1|0.92|0.93|15|328|640; (*GB) gi|5931765|emb|CAB56619.1| (e_value=0.0) phragmoplastin [
NbS00012365g0017.1 NbS00012365g0017.1 protein AED:0.10 eAED:0.10 QI:65|1|0.87|1|1|1|8|334|389; (*GB) gi|255558636|ref|XP_002520343.1| (e_value=0.0) replication fact
NbS00003662g0021.1 NbS00003662g0021.1 protein AED:0.24 eAED:0.34 QI:0|0|0|1|0|0|3|0|257; (*GB) gi|78102516|ref|YP_358657.1| (e_value=6e-112) ATP synthase CF1 alpha
NbS00052235g0008.1 NbS00052235g0008.1 protein AED:0.09 eAED:0.09 QI:76|0.66|0.5|1|1|1|4|0|542; (*GB) gi|350537917|ref|NP_001234829.1| (e_value=0.0) ascorbate oxidas
NbS00001148g0004.1 NbS00001148g0004.1 protein AED:0.25 eAED:0.26 QI:0|0.66|0.5|1|0|0|4|455|319; (*GB) gi|226506550|ref|NP_001141544.1| (e_value=2e-158) uncharacteri
NbS00000565g0021.1 NbS00000565g0021.1 protein AED:0.30 eAED:0.33 QI:204|0.83|0.94|1|0.77|0.84|19|0|515; (*SWP) sp|Q8R3N6|THOC1_MOUSE (e_value=1e-53) THO complex sub
NbS00001859g0006.1 NbS00001859g0006.1 protein AED:0.10 eAED:0.10 QI:0|0.42|0.5|1|0.85|0.75|8|0|558; (*GB) gi|313585890|gb|ADR71054.1| (e_value=0.0) phosphoglycerate
NbS00027742g0013.1 NbS00027742g0013.1 protein AED:0.00 eAED:0.01 QI:0|1|1|1|1|1|7|309|363; (*GB) gi|77416977|gb|ABA81884.1| (e_value=4e-80) nuclear RNA binding prot
NbS00035166g0003.1 NbS00035166g0003.1 protein AED:0.07 eAED:0.07 QI:0|1|0.66|1|1|1|3|0|439; (*GB) gi|13785209|emb|CAC37356.1| (e_value=0.0) putative membrane protei
NbS00045545g0004.1 NbS00045545g0004.1 protein AED:0.13 eAED:0.13 QI:0|0.78|0.73|0.86|0.92|0.86|15|481|616; (*SWP) sp|Q7XTT4|NUCL2_ORYSJ (e_value=6e-73) Nucleolin 2
NbS00000139g0007.1 NbS00000139g0007.1 protein AED:0.24 eAED:0.24 QI:362|1|0.88|1|1|1|9|573|324; (*GB) gi|543867|sp|P26360.2|ATPG3_IPOBA (e_value=0.0) RecName: Full=
NbS00002125g0002.1 NbS00002125g0002.1 protein AED:0.22 eAED:0.22 QI:0|-1|0|1|-1|1|1|0|229; (*GB) gi|47971184|dbj|BAD22534.1| (e_value=7e-143) harpin inducing protei
NbS00000529g0002.1 NbS00000529g0002.1 protein AED:0.44 eAED:0.48 QI:0|0.66|0.5|1|1|1|4|0|104; (*GB) gi|224068340|ref|XP_002302713.1| (e_value=7e-69) predicted prote
NbS00004081g0106.1 NbS00004081g0106.1 protein AED:0.15 eAED:0.16 QI:0|1|0.75|1|1|1|4|0|260
NbS00003163g0002.1 NbS00003163g0002.1 protein ; (*GB) gi|146188483|emb|CAK12837.1| (e_value=1e-52) ribulose 1,5 biphosphate carboxylase/oxygenase [Liparia genistc
NbS00003380g0115.1 NbS00003380g0115.1 protein AED:0.12 eAED:0.12 QI:355|0.66|0.85|1|0.33|0.42|7|134|238
NbS00005988g0006.1 NbS00005988g0006.1 protein AED:0.37 eAED:0.39 QI:248|0|0.5|1|1|1|2|0|118; (*GB) gi|2497757|sp|Q42952.1|NLTP1_TOBAC (e_value=3e-62) RecName: Full=
NbS00003291g0003.1 NbS00003291g0003.1 protein AED:0.07 eAED:0.07 QI:121|0.83|0.85|1|1|1|7|382|226; (*TAIR) AT3G07030.1 (e_value=4e-47) | Symbols: | Alba DNA/RNA-bi
NbS00005976g0007.1 NbS00005976g0007.1 protein AED:0.18 eAED:0.18 QI:185|0.87|0.88|1|0.87|0.88|9|435|268; (*GB) gi|145323882|ref|NP_001077530.1| (e_value=2e-169) gly
>gi|74181742|dbj|BAE32582.1| >gi|74181742|dbj|BAE32582.1| unnamed protein product [Mus musculus]
NbS00010046g0020.1 NbS00010046g0020.1 protein AED:0.25 eAED:0.25 QI:0|0.76|0.66|0.94|0.64|0.66|18|0|588; (*GB) gi|171854667|dbj|BAG16523.1| (e_value=0.0) putative N
NbS00000058g0018.1 NbS00000058g0018.1 protein AED:0.02 eAED:0.02 QI:0|1|0|1|1|1|2|0|221; (*GB) gi|30013659|gb|AAP03872.1| (e_value=2e-130) putative photosystem I su
NbS00059497g0003.1 NbS00059497g0003.1 protein AED:0.17 eAED:0.17 QI:0|1|1|1|1|1|3|550|697; (*SWP) sp|Q653H7|ARFR_ORYSJ (e_value=0.0) Auxin response factor 18 OS=Ory
NbS00033024g0001.1 NbS00033024g0001.1 protein AED:0.17 eAED:0.17 QI:0|0.85|0.87|1|1|1|8|260|540; (*GB) gi|255554849|ref|XP_002518462.1| (e_value=0.0) multicopper ox

<table>
<tr><td>NbS00002372g0015.1</td><td>NbS00002372g0015.1 protein AED:0.22 eAED:0.23 QI:207|0.66|0.5|1|1|1|4|0|452; (*GB) gi|110083391|dbj|BAE97400.1| (e_value=8e-141) heat shock prote</td></tr>
<tr><td>NbS00008558g0004.1</td><td>NbS00008558g0004.1 protein AED:0.13 eAED:0.13 QI:0|1|0.8|1|1|1|5|0|398; (*GB) gi|4827253|dbj|BAA77603.1| (e_value=0.0) plastidic aldolase [Nicoti</td></tr>
<tr><td>NbS00001259g0009.1</td><td>NbS00001259g0009.1 protein AED:0.04 eAED:0.04 QI:128|0.75|0.77|1|0.75|0.88|9|0|318; (*GB) gi|357505379|ref|XP_003622978.1| (e_value=2e-55) Ribonu</td></tr>
<tr><td>NbS00014241g0002.1</td><td>NbS00014241g0002.1 protein AED:0.09 eAED:0.09 QI:0|0|0|1|0|0|2|0|201; (*SWP) sp|P00823|ATPA_TOBAC (e_value=5e-67) ATP synthase subunit alpha, chl</td></tr>
<tr><td>NbS00007742g0006.1</td><td>NbS00007742g0006.1 protein AED:0.40 eAED:0.42 QI:0|0.5|0.42|1|1|1|7|0|319; (*GB) gi|94466659|emb|CAJ44458.1| (e_value=2e-110) ALY protein [Nicoti</td></tr>
<tr><td>NbS00001538g0002.1</td><td>NbS00001538g0002.1 protein AED:0.19 eAED:0.19 QI:248|1|1|1|1|1|11|261|788; (*SWP) sp|Q7TP47|HNRPQ_RAT (e_value=2e-31) Heterogeneous nuclear ribon</td></tr>
<tr><td>NbS00001587g0026.1</td><td>NbS00001587g0026.1 protein AED:0.19 eAED:0.20 QI:234|0.91|0.83|1|0.82|0.83|24|284|993; (*SWP) sp|P35601|RFC1_MOUSE (e_value=5e-125) Replication f</td></tr>
<tr><td>NbS00000548g0008.1</td><td>NbS00000548g0008.1 protein AED:0.29 eAED:0.30 QI:0|0.8|0.5|1|1|0.66|6|0|363; (*GB) gi|255573386|ref|XP_002527619.1| (e_value=2e-130) Ras-GTPase-a</td></tr>
<tr><td>NbS00023006g0207.1</td><td>NbS00023006g0207.1 protein</td></tr>
<tr><td>NbS00001970g0004.1</td><td>NbS00001970g0004.1 protein AED:0.12 eAED:0.12 QI:0|1|0.8|1|1|1|5|253|296; (*GB) gi|82400118|gb|ABB72798.1| (e_value=3e-171) 40S ribosomal protein</td></tr>
<tr><td>NbS00010947g0011.1</td><td>NbS00010947g0011.1 protein AED:0.21 eAED:0.21 QI:0|1|0.88|1|0.87|0.77|9|197|534; (*SWP) sp|Q9FZL3|MGDG_TOBAC (e_value=0.0) Probable monogalactosy</td></tr>
<tr><td>NbS00059407g0001.1</td><td>NbS00059407g0001.1 protein AED:0.31 eAED:0.31 QI:0|0|0|1|1|1|3|0|138; (*SWP) sp|P06005|PSBD_SPIOL (e_value=4e-44) Photosystem II D2 protein OS=Sp</td></tr>
<tr><td>NbS00016165g0010.1</td><td>NbS00016165g0010.1 protein AED:0.19 eAED:0.19 QI:75|0|0.5|1|1|1|2|0|264; (*GB) gi|121663827|dbj|BAF44533.1| (e_value=9e-166) class IV chitinase [</td></tr>
<tr><td>NbS00002028g0009.1</td><td>NbS00002028g0009.1 protein AED:0.09 eAED:0.09 QI:0|0|0|0.5|1|1|2|0|201; (*GB) gi|225452646|ref|XP_002281856.1| (e_value=4e-122) PREDICTED: protei</td></tr>
<tr><td>NbS00000215g0010.1</td><td>NbS00000215g0010.1 protein AED:0.11 eAED:0.11 QI:211|1|1|1|0.85|0.8|15|450|605; (*GB) gi|225442531|ref|XP_002284134.1| (e_value=0.0) PREDICTED: r</td></tr>
<tr><td>NbS00018705g0023.1</td><td>NbS00018705g0023.1 protein AED:0.30 eAED:0.31 QI:0|0.66|0.75|1|0.33|0.75|4|388|71; (*GB) gi|242050850|ref|XP_002463169.1| (e_value=7e-39) hypothe</td></tr>
<tr><td>NbS00000456g0109.1</td><td>NbS00000456g0109.1 protein AED:0.20 eAED:0.20 QI:215|1|1|1|0.6|0.66|6|685|132</td></tr>
<tr><td>NbS00000485g0008.1</td><td>NbS00000485g0008.1 protein AED:0.27 eAED:0.27 QI:81|1|1|1|0.8|0.83|6|329|238; (*GB) gi|225442156|ref|XP_002275541.1| (e_value=6e-155) PREDICTED:</td></tr>
<tr><td>NbS00007843g0002.1</td><td>NbS00007843g0002.1 protein AED:0.14 eAED:0.14 QI:0|0.76|0.78|1|0.84|0.85|14|2042|813; (*GB) gi|359489218|ref|XP_002270340.2| (e_value=0.0) PREDIC</td></tr>
<tr><td>NbS00008232g0008.1</td><td>NbS00008232g0008.1 protein AED:0.05 eAED:0.09 QI:72|0.85|0.86|1|0.71|0.86|15|519|570; (*GB) gi|359483518|ref|XP_002272702.2| (e_value=0.0) PREDIC</td></tr>
<tr><td>NbS00027615g0006.1</td><td>NbS00027615g0006.1 protein AED:0.24 eAED:0.24 QI:233|1|1|1|1|1|6|475|276; (*GB) gi|161788876|dbj|BAF95072.1| (e_value=0.0) voltage-dependent anic</td></tr>
<tr><td>NbS00004901g0005.1</td><td>NbS00004901g0005.1 protein AED:0.05 eAED:0.05 QI:0|1|0.5|1|1|1|4|0|391; (*GB) gi|231503|sp|P30171.1|ACT11_SOLTU (e_value=0.0) RecName: Full=Actin</td></tr>
<tr><td>NbS00033277g0006.1</td><td>NbS00033277g0006.1 protein AED:0.56 eAED:0.56 QI:0|0|0|0.16|1|1|6|0|118</td></tr>
<tr><td>NbS00001471g0009.1</td><td>NbS00001471g0009.1 protein AED:0.21 eAED:0.22 QI:0|0.87|0.77|1|1|1|9|428|607; (*GB) gi|356555871|ref|XP_003546253.1| (e_value=0.0) PREDICTED: lys</td></tr>
<tr><td>NbS00013115g0003.1</td><td>NbS00013115g0003.1 protein AED:0.19 eAED:0.19 QI:3|0.33|0.5|1|1|1|4|273|152; (*GB) gi|357125783|ref|XP_003564569.1| (e_value=1e-85) PREDICTED: 40</td></tr>
<tr><td>NbS00041069g0013.1</td><td>NbS00041069g0013.1 protein AED:0.01 eAED:0.02 QI:0|0.5|0.2|1|0.75|0.6|5|0|1975; (*GB) gi|296087607|emb|CBI34863.3| (e_value=0.0) unnamed protein</td></tr>
<tr><td>NbS00030095g0005.1</td><td>NbS00030095g0005.1 protein AED:0.14 eAED:0.14 QI:300|0.85|0.75|1|1|1|8|368|607; (*GB) gi|225423849|ref|XP_002281060.1| (e_value=0.0) PREDICTED: u</td></tr>
<tr><td>NbS00008510g0008.1</td><td>NbS00008510g0008.1 protein AED:0.13 eAED:0.13 QI:0|0.89|0.86|0.98|0.93|0.94|50|0|2033; (*GB) gi|296085156|emb|CBI28651.3| (e_value=0.0) unnamed p</td></tr>
<tr><td>NbS00014998g0013.1</td><td>NbS00014998g0013.1 protein AED:0.33 eAED:0.33 QI:0|0.83|0.57|1|0.66|0.57|7|0|574; (*GB) gi|359493612|ref|XP_003634635.1| (e_value=0.0) PREDICTED:</td></tr>
<tr><td>NbS00027609g0007.1</td><td>NbS00027609g0007.1 protein AED:0.23 eAED:0.24 QI:0|0.88|0.84|1|0.94|0.84|19|0|601; (*GB) gi|316980592|dbj|BAJ51944.1| (e_value=0.0) NADP-dependen</td></tr>
<tr><td>NbS00012784g0015.1</td><td>NbS00012784g0015.1 protein AED:0.24 eAED:0.24 QI:310|0.71|0.75|0.87|0.85|0.87|8|0|437; (*GB) gi|2459684|gb|AAB71764.1| (e_value=0.0) catalase 1 [</td></tr>
<tr><td>NbS00015187g0009.1</td><td>NbS00015187g0009.1 protein AED:0.20 eAED:0.20 QI:141|0.94|0.94|1|0.72|0.84|19|64|510; (*GB) gi|356504541|ref|XP_003521054.1| (e_value=0.0) PREDIC</td></tr>
<tr><td>NbS00013071g0001.1</td><td>NbS00013071g0001.1 protein AED:0.14 eAED:0.14 QI:0|0.66|0.25|1|1|1|4|0|360; (*SWP) sp|Q9XIV8|PERN1_TOBAC (e_value=0.0) Peroxidase N1 OS=Nicotiana</td></tr>
<tr><td>NbS00020307g0016.1</td><td>NbS00020307g0016.1 protein AED:0.13 eAED:0.13 QI:0|0.33|0.25|0.75|0.66|0.25|4|0|741; (*GB) gi|75249421|sp|Q93YF5.1|SUVH1_TOBAC (e_value=0.0) RecN</td></tr>
<tr><td>NbS00001594g0015.1</td><td>NbS00001594g0015.1 protein AED:0.04 eAED:0.04 QI:64|0.5|0.6|1|0.75|0.6|5|0|553; (*GB) gi|268619136|gb|ACZ13344.1| (e_value=0.0) tubulin alpha cha</td></tr>
<tr><td>NbS00015419g0005.1</td><td>NbS00015419g0005.1 protein AED:0.32 eAED:0.32 QI:0|1|1|1|0.75|0.6|5|174|287; (*GB) gi|2114050|dbj|BAA20076.1| (e_value=0.0) water channel protein</td></tr>
<tr><td>NbS00061216g0001.1</td><td>NbS00061216g0001.1 protein AED:0.17 eAED:0.17 QI:0|-1|0|1|-1|1|1|0|179; (*SWP) sp|P11670|PRB1_TOBAC (e_value=3e-109) Basic form of pathogenesis-r</td></tr>
<tr><td>NbS00014940g0015.1</td><td>NbS00014940g0015.1 protein AED:0.19 eAED:0.20 QI:221|0.95|0.77|1|0.90|0.95|22|289|1422; (*GB) gi|296086345|emb|CBI31934.3| (e_value=6e-179) unnam</td></tr>
<tr><td>NbS00011860g0002.1</td><td>NbS00011860g0002.1 protein AED:0.14 eAED:0.14 QI:0|0.83|0.76|0.84|1|1|13|0|788; (*GB) gi|255561268|ref|XP_002521645.1| (e_value=0.0) arsenite-res</td></tr>
<tr><td>NbS00048220g0003.1</td><td>NbS00048220g0003.1 protein AED:0.08 eAED:0.08 QI:242|1|0.92|1|0.91|0.92|13|593|685; (*GB) gi|359483452|ref|XP_002270330.2| (e_value=0.0) PREDICTE</td></tr>
<tr><td>NbS00002188g0022.1</td><td>NbS00002188g0022.1 protein AED:0.32 eAED:0.32 QI:0|1|0.5|1|1|1|2|0|227; (*GB) gi|464621|sp|P34091.1|RL6_MESCR (e_value=7e-104) RecName: Full=60S</td></tr>
<tr><td>NbS00015227g0002.1</td><td>NbS00015227g0002.1 protein AED:0.14 eAED:0.16 QI:0|-1|0|1|-1|1|1|0|171; (*GB) gi|237783971|gb|ACR19782.1| (e_value=5e-91) ATP synthase CF1 beta s</td></tr>
<tr><td>NbS00027670g0006.1</td><td>NbS00027670g0006.1 protein AED:0.24 eAED:0.24 QI:614|0.93|0.93|1|1|1|16|163|536; (*SWP) sp|P50433|GLYM_SOLTU (e_value=0.0) Serine hydroxymethyltr</td></tr>
<tr><td>NbS00001709g0013.1</td><td>NbS00001709g0013.1 protein AED:0.15 eAED:0.15 QI:227|1|1|1|0.85|0.75|8|809|341; (*GB) gi|21388546|emb|CAD33241.1| (e_value=0.0) putative mitochon</td></tr>
<tr><td>NbS00019391g0012.1</td><td>NbS00019391g0012.1 protein AED:0.09 eAED:0.09 QI:72|0.88|0.9|1|0.88|1|10|207|369; (*TAIR) AT4G34660.1 (e_value=0.0) | Symbols: | SH3 domain-cont</td></tr>
<tr><td>NbS00031017g0008.1</td><td>NbS00031017g0008.1 protein AED:0.42 eAED:0.42 QI:0|0|0|1|0|0|2|0|197; (*GB) gi|190607593|gb|ACE79459.1| (e_value=4e-63) NBS-coding resistance gen</td></tr>
<tr><td>NbS00003075g0012.1</td><td>NbS00003075g0012.1 protein AED:0.17 eAED:0.17 QI:419|0.94|0.88|1|0.88|0.91|36|397|1540; (*GB) gi|116047943|gb|ABJ53197.1| (e_value=0.0) myosin XI</td></tr>
<tr><td>NbS00002044g0008.1</td><td>NbS00002044g0008.1 protein AED:0.19 eAED:0.19 QI:375|1|1|1|0.5|0.57|7|290|211; (*GB) gi|356555789|ref|XP_003546212.1| (e_value=2e-134) PREDICTED:</td></tr>
<tr><td>NbS00000710g0004.1</td><td>NbS00000710g0004.1 protein AED:0.10 eAED:0.12 QI:195|0.77|0.7|1|0.77|0.5|10|0|501; (*SWP) sp|O94260|G3BP_SCHPO (e_value=3e-21) Putative G3BP-like</td></tr>
<tr><td>NbS00022054g0014.1</td><td>NbS00022054g0014.1 protein AED:0.15 eAED:0.17 QI:48|1|0.71|1|1|1|7|0|661; (*GB) gi|225444019|ref|XP_002281594.1| (e_value=0.0) PREDICTED: cleavag</td></tr>
<tr><td>NbS00007600g0009.1</td><td>NbS00007600g0009.1 protein AED:0.75 eAED:0.75 QI:0|0|0|0.04|0.83|0.88|25|0|918; (*SWP) sp|Q9M3G7|ATM_ARATH (e_value=0.0) Serine/threonine-protein</td></tr>
<tr><td>NbS00002523g0003.1</td><td>NbS00002523g0003.1 protein AED:0.18 eAED:0.18 QI:0|-1|0|1|-1|1|1|0|704; (*GB) gi|75249421|sp|Q93YF5.1|SUVH1_TOBAC (e_value=0.0) RecName: Full=His</td></tr>
<tr><td>NbS00010454g0009.1</td><td>NbS00010454g0009.1 protein AED:0.20 eAED:0.20 QI:0|1|0.5|1|1|1|2|0|333; (*SWP) sp|Q40459|PSBO_TOBAC (e_value=0.0) Oxygen-evolving enhancer protei</td></tr>
<tr><td>NbS00001075g0007.1</td><td>NbS00001075g0007.1 protein AED:0.16 eAED:0.16 QI:0|0.54|0.41|0.91|1|1|12|133|554; (*GB) gi|20522008|dbj|BAB92011.1| (e_value=0.0) pleiotropic dru</td></tr>
<tr><td>NbS00010523g0001.1</td><td>NbS00010523g0001.1 protein AED:0.13 eAED:0.14 QI:0|0.80|0.68|1|1|1|22|0|1481; (*GB) gi|75331994|sp|Q949G3.1|PDR1_NICPL (e_value=0.0) RecName: Ful</td></tr>
<tr><td>NbS00004717g0103.1</td><td>NbS00004717g0103.1 protein AED:0.03 eAED:0.03 QI:0|-1|0|1|-1|1|1|0|128</td></tr>
<tr><td>NbS00017222g0017.1</td><td>NbS00017222g0017.1 protein AED:0.07 eAED:0.07 QI:314|0.66|0.57|1|0.16|0.14|7|435|963; (*GB) gi|255559969|ref|XP_002521003.1| (e_value=7e-93) hypc</td></tr>
<tr><td>NbS00004226g0004.1</td><td>NbS00004226g0004.1 protein ; (*GB) gi|94466657|emb|CAJ44457.1| (e_value=4e-18) ALY protein [Nicotiana benthamiana];; (*ITAG) Solyc10g086400.1.1</td></tr>
<tr><td>NbS00000361g0007.1</td><td>NbS00000361g0007.1 protein AED:0.06 eAED:0.06 QI:0|0.83|0.85|1|0.83|0.71|7|414|546; (*GB) gi|350539281|ref|NP_001234129.1| (e_value=0.0) calnexin</td></tr>
</table>

NbS00008764g0022.1	NbS00008764g0022.1 protein AED:0.14 eAED:0.14 QI:72|1|1|1|0.6|0.66|6|317|286; (*GB) gi|255558218|ref|XP_002520136.1| (e_value=0.0) Protein PPLZ12
NbS00002134g0112.1	NbS00002134g0112.1 protein AED:0.04 eAED:0.13 QI:0|1|0.33|1|1|1|3|0|218
NbS00028158g0015.1	NbS00028158g0015.1 protein AED:0.44 eAED:0.54 QI:0|0.75|0.4|1|1|1|5|0|169; (*GB) gi|388500696|gb|AFK38414.1| (e_value=1e-63) unknown [Lotus japon
NbS00011040g0001.1	NbS00011040g0001.1 protein AED:0.00 eAED:0.03 QI:0|1|0.66|1|0|0|3|164|270; (*GB) gi|255578051|ref|XP_002529896.1| (e_value=1e-58) small nuclear r
NbS00005419g0006.1	NbS00005419g0006.1 protein
NbS00009707g0011.1	NbS00009707g0011.1 protein AED:0.22 eAED:0.22 QI:246|1|1|1|0|0.14|7|394|181; (*GB) gi|115501471|dbj|BAF34209.1| (e_value=7e-133) ADP ribosylation
NbS00004739g0001.1	NbS00004739g0001.1 protein AED:0.17 eAED:0.17 QI:0|1|0.66|1|1|1|3|0|251; (*GB) gi|100801744|emb|CAK24966.1| (e_value=2e-175) chlorophyll a/b bind
NbS00002935g0006.1	NbS00002935g0006.1 protein AED:0.24 eAED:0.28 QI:9|0.53|0.35|1|0.84|0.71|14|0|493; (*GB) gi|6715512|gb|AAF26445.1| (e_value=0.0) vacuolar H+-ATPa
NbS00001581g0007.1	NbS00001581g0007.1 protein AED:0.31 eAED:0.31 QI:0|0.8|0.66|1|1|1|6|0|270; (*GB) gi|161788874|dbj|BAF95071.1| (e_value=4e-156) voltage-dependent
NbS00005939g0108.1	NbS00005939g0108.1 protein AED:0.21 eAED:0.21 QI:0|1|1|1|1|1|8|398|765
NbS00007972g0032.1	NbS00007972g0032.1 protein AED:0.25 eAED:0.25 QI:0|0.77|0.5|1|0.77|0.9|10|0|571; (*GB) gi|350534776|ref|NP_001234159.1| (e_value=0.0) pollen-spec
NbS00002844g0211.1	NbS00002844g0211.1 protein AED:0.10 eAED:0.10 QI:261|1|1|1|1|1|18|392|575
NbS00008163g0010.1	NbS00008163g0010.1 protein AED:0.09 eAED:0.09 QI:186|0.87|0.88|1|1|1|9|300|633; (*GB) gi|359479362|ref|XP_002277357.2| (e_value=0.0) PREDICTED: r
NbS00019145g0009.1	NbS00019145g0009.1 protein AED:0.20 eAED:0.20 QI:118|0.77|0.78|1|1|1|19|310|609; (*GB) gi|255554262|ref|XP_002518171.1| (e_value=0.0) chaperonin-
NbS00009748g0101.1	NbS00009748g0101.1 protein AED:0.12 eAED:0.12 QI:0|-1|0|1|-1|1|1|0|600
NbS00005120g0006.1	NbS00005120g0006.1 protein
NbS00008453g0109.1	NbS00008453g0109.1 protein AED:0.28 eAED:0.44 QI:0|0|0|0.66|0.5|0.33|3|0|219
NbS00003332g0002.1	NbS00003332g0002.1 protein AED:0.22 eAED:0.22 QI:0|0.90|0.83|0.91|0.90|0.83|12|422|580; (*GB) gi|255561807|ref|XP_002521913.1| (e_value=0.0) PLE,
NbS00010101g0016.1	NbS00010101g0016.1 protein AED:0.09 eAED:0.09 QI:12|1|1|1|0.37|0.22|9|70|546; (*GB) gi|296084540|emb|CBI25561.3| (e_value=0.0) unnamed protein pr
NbS00010498g0007.1	NbS00010498g0007.1 protein AED:0.12 eAED:0.12 QI:0|1|1|1|0.5|1|3|646|228; (*GB) gi|384038831|gb|AFH58006.1| (e_value=5e-128) chloroplast PsbQ2 pr
NbS00014411g0036.1	NbS00014411g0036.1 protein AED:0.22 eAED:0.22 QI:406|0.87|0.92|1|0.83|0.88|25|199|921; (*GB) gi|297743300|emb|CBI36167.3| (e_value=0.0) unnamed p
gi|14198259|gb|AAH08189.1|	gi|14198259|gb|AAH08189.1| Unknown (protein for IMAGE:3584589), partial [Mus musculus]
NbS00010129g0001.1	NbS00010129g0001.1 protein AED:0.16 eAED:0.16 QI:0|0|0.5|1|1|1|2|131|318; (*SWP) sp|P23547|E13G_TOBAC (e_value=0.0) Glucan endo-1,3-beta-glucosid
NbS00010282g0017.1	NbS00010282g0017.1 protein AED:0.32 eAED:0.32 QI:0|0.5|0.8|1|0.75|0.8|5|368|516; (*GB) gi|551259|emb|CAA57428.1| (e_value=0.0) beta-fructofuranos
NbS00004795g0001.1	NbS00004795g0001.1 protein AED:0.06 eAED:0.06 QI:293|1|0.88|1|0.75|0.77|9|0|380; (*GB) gi|255561457|ref|XP_002521739.1| (e_value=1e-163) auxin:hy
NbS00036843g0004.1	NbS00036843g0004.1 protein AED:0.04 eAED:0.04 QI:8|0.5|0.66|1|0.5|0.33|3|0|387; (*SWP) sp|P25083|ADT1_SOLTU (e_value=0.0) ADP,ATP carrier protein
NbS00002113g0001.1	NbS00002113g0001.1 protein AED:0.00 eAED:0.05 QI:0|-1|0|1|-1|1|1|0|394; (*SWP) sp|Q00874|DR100_ARATH (e_value=1e-127) DNA-damage-repair/toleratic
NbS00059593g0005.1	NbS00059593g0005.1 protein AED:0.21 eAED:0.21 QI:386|1|1|1|0.87|0.88|9|535|469; (*GB) gi|296083732|emb|CBI23721.3| (e_value=1e-157) unnamed prote
NbS00001259g0107.1	NbS00001259g0107.1 protein AED:0.28 eAED:0.28 QI:192|0.66|0.4|1|1|1|10|0|433
NbS00005007g0108.1	NbS00005007g0108.1 protein AED:0.09 eAED:0.09 QI:0|1|0.75|1|1|1|4|444|693
NbS00007190g0001.1	NbS00007190g0001.1 protein AED:0.08 eAED:0.08 QI:179|1|1|1|0.5|0.33|3|443|275; (*GB) gi|225448932|ref|XP_002267178.1| (e_value=1e-93) PREDICTED:
NbS00005969g0002.1	NbS00005969g0002.1 protein AED:0.07 eAED:0.07 QI:0|0.5|0.55|0.88|1|1|9|177|561; (*GB) gi|225456270|ref|XP_002283518.1| (e_value=0.0) PREDICTED: n
NbC24284457g0001.1	NbC24284457g0001.1 protein
NbS00011397g0001.1	NbS00011397g0001.1 protein AED:0.61 eAED:0.61 QI:0|0|0|0.5|1|1|2|0|130; (*SWP) sp|A1XQR9|RUXE_PIG (e_value=3e-31) Small nuclear ribonucleoprotein
NbS00005851g0011.1	NbS00005851g0011.1 protein AED:0.18 eAED:0.18 QI:3|0.89|0.85|1|0.78|0.9|20|310|678; (*GB) gi|224062133|ref|XP_002300771.1| (e_value=0.0) predicte
NbS00001525g0121.1	NbS00001525g0121.1 protein AED:0.18 eAED:0.18 QI:3|0.62|0.22|1|0.75|0.77|9|0|561
NbS00003153g0003.1	NbS00003153g0003.1 protein AED:0.15 eAED:0.15 QI:171|0.94|0.94|1|0.88|0.89|19|592|920; (*SWP) sp|O82188|RDR5_ARATH (e_value=0.0) Probable RNA-dep
NbS00014412g0011.1	NbS00014412g0011.1 protein AED:0.27 eAED:0.27 QI:0|1|0|1|1|1|2|0|213; (*GB) gi|42559164|sp|O80360.1|RK3_TOBAC (e_value=2e-131) RecName: Full=50S
NbS00001563g0013.1	NbS00001563g0013.1 protein AED:0.00 eAED:0.03 QI:0|1|0.66|1|1|1|3|0|297; (*GB) gi|225451038|ref|XP_002284939.1| (e_value=4e-145) PREDICTED: UPF06
NbS00002808g0033.1	NbS00002808g0033.1 protein AED:0.14 eAED:0.14 QI:0|1|0.94|1|0.88|0.94|18|425|576; (*GB) gi|225433255|ref|XP_002285452.1| (e_value=0.0) PREDICTED:

Select Summary Report

Format As [Select Summary (protein hits)] Help

Significance threshold p< [0.05] Max. number of hits [AUTO]

Standard scoring ○ MudPIT scoring ◉ Ions score or expect cut-off [30] Show sub-sets [0]

Show pop-ups ◉ Suppress pop-ups ○ Require bold red ☐

Re-Search ◉ All queries ○ Unassigned ○ Below homology threshold ○ Below identity threshold

1. gi|297262447|ref|XP_001098182.2| **Mass:** 65341 **Score:** 412 **Matches:** 17(16) **Sequences:** 13(12) **emPAI:** 0.89

gi|297262447|ref|XP_001098182.2| PREDICTED: keratin, type II cytoskeletal 1-like isoform 6 [Macaca mulatta]

Query	Observed	Mr(expt)	Mr(calc)	ppm	Miss	Score	Expect	Rank	Unique	Peptide
10175	416.7481	831.4817	831.4814	0.39	0	30	0.076	1	U	K.SISISVAR.G
10440	437.7529	873.4912	873.4920	-0.83	0	36	0.023	1	U	R.SLVNLGGSK.S
11334	487.2692	972.5238	972.5240	-0.20	0	41	0.0071	1		K.IEISELNR.V 11335

12689	571.2639	1140.5132	1140.5121	0.93	0	54	0.00018	1	U	R.DYQELMNTK.L
13014	590.3038	1178.5931	1178.5931	-0.01	0	63	3.7e-005	1	U	K.YEELQITAGR.H
14183	651.8609	1301.7072	1301.7078	-0.44	0	90	6.6e-008	1	U	R.SLDLDSIIAEVK.A
14878	692.3495	1382.6845	1382.6830	1.11	0	73	3.1e-006	1	U	K.SLNNQFASFIDK.V 14876
14959	465.2488	1392.7245	1392.7249	-0.26	1	37	0.014	1	U	R.TNAENEFVTIKK.D
14964	697.3709	1392.7273	1392.7249	1.72	1	(34)	0.026	1	U	R.TNAENEFVTIKK.D
15556	738.3786	1474.7427	1474.7416	0.71	0	68	1.2e-005	1	U	K.WELLQQVDTSTR.T
15562	738.3974	1474.7802	1474.7780	1.53	0	59	8.3e-005	1		R.FLEQQNQVLQTK.W 15561
16532	800.4189	1598.8233	1598.8264	-1.89	1	38	0.011	1	U	K.NKLNDLEDALQQAK.E
18898	648.0012	1940.9817	1940.9803	0.70	1	57	0.00012	1	U	K.LNDLEDALQQAKEDLAR.L
20746	906.3678	2716.0816	2716.0771	1.64	0	31	0.0039	1	U	R.GSYGSGGGSYGSGGGGGGHGSYGSGSSSGGYR.G

---

2. NbS00031648g0011.1 **Mass:** 99402 **Score:** 411 **Matches:** 24(23) **Sequences:** 18(18) **emPAI:** 0.85

NbS00031648g0011.1 protein AED:0.23 eAED:0.23 QI:280|1|1|1|0.95|0.91|23|300|916; (*GB) gi|225465253|ref|XP_002268311.1| (e_value=0.0) PREDICTED: dynamin-2B [Vitis vinifera];; (*SWP) sp|Q9LQ55|DRP2[illegible]

Query	Observed	Mr(expt)	Mr(calc)	ppm	Miss	Score	Expect	Rank	Unique	Peptide
9838	394.7368	787.4590	787.4592	-0.25	0	34	0.033	1	U	K.APNLVFK.I
11053	473.2634	944.5123	944.5113	1.06	0	36	0.025	1	U	K.ALALELCR.E
11294	485.2842	968.5539	968.5542	-0.33	0	32	0.027	1	U	K.LIDLPGVDK.G
12677	570.3426	1138.6706	1138.6710	-0.32	0	35	0.0078	1	U	R.LPNLLSGLQGK.S 12679
12864	581.8028	1161.5910	1161.5886	2.10	0	40	0.009	1	U	K.MVVALVDMER.V 12863
12952	586.8170	1171.6194	1171.6197	-0.29	0	39	0.0099	1	U	K.SQVVQDELVR.L 12954
13650	628.3307	1254.6468	1254.6456	1.01	0	56	0.00018	1	U	K.LYSSISAQSTAK.I
14743	682.3307	1362.6468	1362.6449	1.40	0	48	0.001	1	U	R.LGEQMVNSAEGTK.A
14765	683.8415	1365.6684	1365.6677	0.52	0	43	0.0033	1	U	K.VVTSFEGNFPNR.I
15388	725.8498	1449.6850	1449.6848	0.16	0	36	0.016	1	U	R.SAFDAAANGPSSLSR.Y
15431	729.3767	1456.7389	1456.7409	-1.40	0	42	0.0044	1	U	K.IEELLQEDQNVK.R
15616	740.4354	1478.8563	1478.8569	-0.41	0	49	0.00026	1	U	K.VLAAVQALLSGQGPR.S 15618
15617	493.9596	1478.8569	1478.8569	-0.03	0	(42)	0.0013	1	U	K.VLAAVQALLSGQGPR.S
15823	752.4070	1502.7995	1502.7981	0.99	0	33	0.029	1	U	K.TAGPEGEITAGFLLK.R
16410	789.9280	1577.8415	1577.8413	0.12	0	31	0.048	1	U	R.SSTFLNVVAIGGTGAGK.S
16850	538.6214	1612.8424	1612.8420	0.22	1	31	0.053	1	U	K.IEELLQEDQNVKR.R
18781	959.9725	1917.9304	1917.9279	1.28	1	31	0.045	1	U	K.QAAALLADEDVDETSSKR.S
18870	968.0101	1934.0057	1934.0037	1.06	0	59	5.8e-005	1	U	R.IVLEADGYQPYLISPEK.G 18869
19583	702.0439	2103.1100	2103.1073	1.30	0	52	0.00028	1	U	K.SAVLNSLVGHPALPTGEGGATR.A

---

3. gi|28317|emb|CAA32649.1| **Mass:** 59720 **Score:** 356 **Matches:** 17(16) **Sequences:** 13(12) **emPAI:** 0.90

gi|28317|emb|CAA32649.1| unnamed protein product [Homo sapiens]

Query	Observed	Mr(expt)	Mr(calc)	ppm	Miss	Score	Expect	Rank	Unique	Peptide
9970	404.2033	806.3921	806.3923	-0.16	0	40	0.0091	1	U	R.LAADDFR.L
11496	497.2540	992.4934	992.4927	0.77	0	35	0.027	1	U	K.YENEVALR.Q
12273	545.7693	1089.5241	1089.5237	0.42	0	68	1.1e-005	1	U	K.VTMQNLNDR.L 12274
12451	555.2491	1108.4836	1108.4825	1.00	0	35	0.014	1	U	K.DAEAWFNEK.S 12449
12900	583.2960	1164.5775	1164.5775	0.02	0	36	0.02	1	U	R.LENEIQTYR.S
13210	601.3124	1200.6102	1200.6098	0.30	0	40	0.0072	1	U	R.QSVEADINGLR.R
13489	412.2317	1233.6731	1233.6717	1.18	1	31	0.058	1	U	R.LKYENEVALR.Q
13709	631.8025	1261.5904	1261.5899	0.45	0	48	0.00095	1	U	R.SLLEGEGSSGGGGR.G
14762	683.3241	1364.6336	1364.6320	1.19	0	61	4.5e-005	1	U	R.SQYEQLAEQNR.K
9250	691.3300	1380.6454	1380.6408	3.34	0	57	0.00035	1	U	R.ALEESNYELEGK.I 14856 14857
14933	695.8447	1389.6748	1389.6736	0.88	0	96	2e-008	1	U	K.QSLEASLAETEGR.Y
15700	498.5828	1492.7266	1492.7270	-0.28	1	43	0.0037	1	U	R.SQYEQLAEQNRK.D
20951	1018.2158	3051.6256	3051.6200	1.85	1	34	0.0088	1	U	K.TIDDLKNQILNLTTDNANILLQIDNAR.L

---

4. gi|291410763|ref|XP_002721657.1| **Mass:** 27347 **Score:** 259 **Matches:** 13(12) **Sequences:** 5(5) **emPAI:** 1.24

gi|291410763|ref|XP_002721657.1| PREDICTED: histone cluster 1, H2ag-like [Oryctolagus cuniculus]

Query	Observed	Mr(expt)	Mr(calc)	ppm	Miss	Score	Expect	Rank	Unique	Peptide
11041	472.7688	943.5230	943.5240	-0.96	0	71	8e-006	1		

Query	Observed	Mr(expt)	Mr(calc)	ppm	Miss	Score	Expect	Rank	Unique	Peptide
										R.AGLQFPVGR.V 11042
8414	590.8100	1179.6054	1179.6135	-6.85	0	64	2.8e-005	1		R.ISGLIYEETR.G 8413 13019
14262	655.8555	1309.6965	1309.6952	1.02	0	60	6.2e-005	1		K.TVTAMDVVYALK.R 14264
14406	442.5890	1324.7453	1324.7463	-0.72	0	56	0.0001	1		R.DNIQGITKPAIR.R 14405
14408	663.3812	1324.7479	1324.7463	1.23	0	(41)	0.0026	1		R.DNIQGITKPAIR.R 14407
14422	663.8524	1325.6902	1325.6901	0.05	0	(37)	0.012	1		K.TVTAMDVVYALK.R
15499	733.9054	1465.7962	1465.7963	-0.03	1	58	7.8e-005	1		K.TVTAMDVVYALKR.Q

---

5. gi|148727309|ref|NP_001092039.1| **Mass:** 66230 **Score:** 248 **Matches:** 12(12) **Sequences:** 8(8) **emPAI:** 0.47

gi|148727309|ref|NP_001092039.1| keratin, type II cytoskeletal 2 epidermal [Pan troglodytes]

Query	Observed	Mr(expt)	Mr(calc)	ppm	Miss	Score	Expect	Rank	Unique	Peptide
11334	487.2692	972.5238	972.5240	-0.20	0	41	0.0071	1		K.IEISELNR.V 11335
12109	534.7553	1067.4961	1067.4957	0.32	0	34	0.023	1	U	K.DVDNAYMIK.V
12436	554.2755	1106.5365	1106.5356	0.79	0	45	0.0029	1	U	K.AQYEEIAQR.S 12434
13636	627.8077	1253.6008	1253.6001	0.60	0	77	1.4e-006	1	U	R.GFSSGSAVVSGGSR.R
14449	665.3668	1328.7190	1328.7187	0.19	0	75	2.1e-006	1	U	R.NLDLDSIIAEVK.A
15462	730.9042	1459.7938	1459.7922	1.08	0	39	0.0075	1	U	K.VDLLNQEIEFLK.V 15461
15562	738.3974	1474.7802	1474.7780	1.53	0	59	8.3e-005	1		R.FLEQQNQVLQTK.W 15561
20955	1022.1700	3063.4883	3063.4819	2.09	0	36	0.0071	1	U	K.VLYDAEISQIHQSVTDTNVILSMDNSR.N

---

6. NbS00000666g0001.1 **Mass:** 15719 **Score:** 239 **Matches:** 10(9) **Sequences:** 4(4) **emPAI:** 2.22

NbS00000666g0001.1 protein AED:0.15 eAED:0.15 QI:0|-1|0|1|-1|1|1|0|145; (*GB) gi|82400146|gb|ABB72812.1| (e_value=5e-61) histone H2B-like protein [Solanum tuberosum];; (*SWP) sp|Q1S9I9|H2B1_MEDTR

Query	Observed	Mr(expt)	Mr(calc)	ppm	Miss	Score	Expect	Rank	Unique	Peptide
10147	414.7142	827.4139	827.4137	0.15	0	35	0.013	1	U	K.HAVSEGTK.A
10995	470.2967	938.5789	938.5800	-1.23	0	38	0.0028	1	U	R.LVLPGELAK.H
13026	394.2153	1179.6240	1179.6248	-0.68	0	44	0.0023	1	U	K.QVHPDIGISSK.A 13027
13032	590.8199	1179.6253	1179.6248	0.47	0	(30)	0.063	1	U	K.QVHPDIGISSK.A
17730	865.4201	1728.8257	1728.8215	2.41	0	(53)	0.00028	1	U	K.AMGIMNSFINDIFEK.L
17816	873.4164	1744.8182	1744.8164	1.04	0	88	7.9e-008	1	U	K.AMGIMNSFINDIFEK.L 17822
17935	881.4137	1760.8128	1760.8113	0.86	0	(51)	0.00037	1	U	K.AMGIMNSFINDIFEK.L 17930

Proteins matching the same set of peptides:

NbS00000678g0001.1 **Mass:** 15849 **Score:** 239 **Matches:** 10(9) **Sequences:** 4(4)
NbS00000678g0001.1 protein AED:0.22 eAED:0.22 QI:0|-1|0|1|-1|1|1|0|147; (*GB) gi|82400146|gb|ABB72812.1| (e_value=5e-61) histone H2B-like protein [Solanum tuberosum];; (*SWP) sp|Q1S9I9|H2B1_MEDTR

NbS00000678g0002.1 **Mass:** 15863 **Score:** 239 **Matches:** 10(9) **Sequences:** 4(4)
NbS00000678g0002.1 protein AED:0.22 eAED:0.22 QI:0|-1|0|1|-1|1|1|0|147; (*GB) gi|82400146|gb|ABB72812.1| (e_value=5e-61) histone H2B-like protein [Solanum tuberosum];; (*SWP) sp|Q1S9I9|H2B1_MEDTR

NbS00000678g0009.1 **Mass:** 16874 **Score:** 239 **Matches:** 10(9) **Sequences:** 4(4)
NbS00000678g0009.1 protein AED:0.29 eAED:0.29 QI:0|0|0|0.5|1|1|2|0|156; (*GB) gi|388514209|gb|AFK45166.1| (e_value=1e-55) unknown [Lotus japonicus];; (*SWP) sp|Q1SU99|H2B3_MEDTR (e_value=5e-57) Prc

NbS00001050g0001.1 **Mass:** 16027 **Score:** 239 **Matches:** 10(9) **Sequences:** 4(4)
NbS00001050g0001.1 protein AED:0.13 eAED:0.14 QI:0|-1|0|1|-1|1|1|0|147; (*GB) gi|388522051|gb|AFK49087.1| (e_value=3e-60) unknown [Lotus japonicus];; (*SWP) sp|P40283|H2B11_ARATH (e_value=2e-61) H:

NbS00006411g0002.1 **Mass:** 15863 **Score:** 239 **Matches:** 10(9) **Sequences:** 4(4)
NbS00006411g0002.1 protein AED:0.22 eAED:0.22 QI:0|-1|0|1|-1|1|1|0|147; (*GB) gi|82400146|gb|ABB72812.1| (e_value=5e-61) histone H2B-like protein [Solanum tuberosum];; (*SWP) sp|Q1S9I9|H2B1_MEDTR

NbS00007991g0006.1 **Mass:** 17158 **Score:** 239 **Matches:** 10(9) **Sequences:** 4(4)
NbS00007991g0006.1 protein AED:0.08 eAED:0.08 QI:0|0|0|1|1|1|2|0|157; (*GB) gi|146336941|gb|ABQ23584.1| (e_value=2e-57) putative histone [Medicago truncatula];; (*SWP) sp|Q1S9I9|H2B1_MEDTR (e_value

NbS00008452g0101.1 **Mass:** 15935 **Score:** 239 **Matches:** 10(9) **Sequences:** 4(4)
NbS00008452g0101.1 protein AED:0.16 eAED:0.16 QI:0|1|0.5|1|0|0|2|347|146

NbS00017233g0003.1 **Mass:** 16025 **Score:** 239 **Matches:** 10(9) **Sequences:** 4(4)
NbS00017233g0003.1 protein AED:0.20 eAED:0.20 QI:0|-1|0|1|-1|1|1|0|146; (*SWP) sp|P93354|H2B_TOBAC (e_value=4e-62) Histone H2B OS=Nicotiana tabacum GN=HIS2B PE=2 SV=3;; (*TAIR) AT5G59910.1 (e_value

NbS00020161g0001.1 **Mass:** 16125 **Score:** 239 **Matches:** 10(9) **Sequences:** 4(4)
NbS00020161g0001.1 protein AED:0.20 eAED:0.20 QI:0|-1|0|1|-1|1|1|0|147; (*GB) gi|82400146|gb|ABB72812.1| (e_value=3e-60) histone H2B-like protein [Solanum tuberosum];; (*SWP) sp|Q1S9I9|H2B1_MEDTR

NbS00023525g0004.1 **Mass:** 15039 **Score:** 239 **Matches:** 10(9) **Sequences:** 4(4)
NbS00023525g0004.1 protein AED:0.27 eAED:0.28 QI:0|0|0|0.5|1|1|2|0|137; (*GB) gi|356576357|ref|XP_003556299.1| (e_value=4e-60) PREDICTED: probable histone H2B.1-like [Glycine max];; (*SWP) sp|Q1S9:

NbS00027179g0002.1 **Mass:** 15949 **Score:** 239 **Matches:** 10(9) **Sequences:** 4(4)
NbS00027179g0002.1 protein AED:0.27 eAED:0.27 QI:0|-1|0|1|-1|1|1|0|146; (*GB) gi|388522051|gb|AFK49087.1| (e_value=1e-60) unknown [Lotus japonicus];; (*SWP) sp|Q1S9I9|H2B1_MEDTR (e_value=6e-62) Prc

NbS00028800g0003.1 **Mass:** 16210 **Score:** 239 **Matches:** 10(9) **Sequences:** 4(4)
NbS00028800g0003.1 protein AED:0.16 eAED:0.16 QI:0|-1|0|1|-1|1|1|0|148; (*GB) gi|118484226|gb|ABK93993.1| (e_value=3e-60) unknown [Populus trichocarpa];; (*SWP) sp|Q1S9I9|H2B1_MEDTR (e_value=1e-61)

NbS00032978g0007.1 **Mass:** 15457 **Score:** 239 **Matches:** 10(9) **Sequences:** 4(4)
NbS00032978g0007.1 protein AED:0.01 eAED:0.01 QI:0|-1|0|1|-1|1|1|0|141; (*GB) gi|82400146|gb|ABB72812.1| (e_value=2e-60) histone H2B-like protein [Solanum tuberosum];; (*SWP) sp|Q1S9I9|H2B1_MEDTR
NbS00037466g0010.1 **Mass:** 20264 **Score:** 239 **Matches:** 10(9) **Sequences:** 4(4)
NbS00037466g0010.1 protein AED:0.02 eAED:0.03 QI:0|0|0|1|1|1|2|0|185; (*GB) gi|388514209|gb|AFK45166.1| (e_value=3e-60) unknown [Lotus japonicus];; (*SWP) sp|Q1S9I9|H2B1_MEDTR (e_value=2e-61) Proba
NbS00046472g0002.1 **Mass:** 15732 **Score:** 239 **Matches:** 10(9) **Sequences:** 4(4)
NbS00046472g0002.1 protein AED:0.00 eAED:0.00 QI:0|-1|0|1|-1|1|1|0|143; (*GB) gi|225435052|ref|XP_002284344.1| (e_value=3e-64) PREDICTED: probable histone H2B.1-like [Vitis vinifera];; (*SWP) sp|O2
NbC24219583g0001.1 **Mass:** 11803 **Score:** 239 **Matches:** 10(9) **Sequences:** 4(4)
NbC24219583g0001.1 protein AED:0.00 eAED:0.00 QI:0|-1|0|1|-1|1|1|0|107; (*GB) gi|82400146|gb|ABB72812.1| (e_value=1e-61) histone H2B-like protein [Solanum tuberosum];; (*SWP) sp|Q1S9I9|H2B1_MEDTR
NbC25892755g0001.1 **Mass:** 15725 **Score:** 239 **Matches:** 10(9) **Sequences:** 4(4)
NbC25892755g0001.1 protein AED:0.00 eAED:0.00 QI:0|-1|0|1|-1|1|1|0|143; (*GB) gi|224065198|ref|XP_002301712.1| (e_value=3e-75) histone 2 [Populus trichocarpa];; (*SWP) sp|Q1S9I9|H2B1_MEDTR (e_value

---

7. NbS00004898g0002.1 **Mass:** 11402 **Score:** 226 **Matches:** 12(11) **Sequences:** 5(5) **emPAI:** 5.32
NbS00004898g0002.1 protein AED:0.00 eAED:0.00 QI:0|-1|0|1|-1|1|1|0|103; (*GB) gi|195617694|gb|ACG30677.1| (e_value=1e-51) histone H4 [Zea mays];; (*SWP) sp|P0CG89|H4_SOYBN (e_value=1e-52) Histone H

Query	Observed	Mr(expt)	Mr(calc)	ppm	Miss	Score	Expect	Rank	Unique	Peptide
**11580**	**502.2999**	**1002.5852**	**1002.5862**	**-1.01**	**0**	**42**	**0.0034**	**1**	**U**	**K.IFLENVIR.D**
8414	590.8100	1179.6054	1179.6135	-6.85	0	64	2.8e-005	1		R.ISGLIYEETR.G 8413 13019
14262	655.8555	1309.6965	1309.6952	1.02	0	60	6.2e-005	1		K.TVTAMDVVYALK.R 14264
14406	442.5890	1324.7453	1324.7463	-0.72	0	56	0.0001	1		R.DNIQGITKPAIR.R 14405
14408	663.3812	1324.7479	1324.7463	1.23	0	(41)	0.0026	1		R.DNIQGITKPAIR.R 14407
14422	663.8524	1325.6902	1325.6901	0.05	0	(37)	0.012	1		K.TVTAMDVVYALK.R
15499	733.9054	1465.7962	1465.7963	-0.03	1	58	7.8e-005	1		K.TVTAMDVVYALKR.Q

Proteins matching the same set of peptides:
NbS00007760g0001.1 **Mass:** 11402 **Score:** 226 **Matches:** 12(11) **Sequences:** 5(5)
NbS00007760g0001.1 protein AED:0.00 eAED:0.00 QI:286|1|0.5|1|0|0|2|0|103; (*GB) gi|195617694|gb|ACG30677.1| (e_value=1e-51) histone H4 [Zea mays];; (*SWP) sp|P0CG89|H4_SOYBN (e_value=1e-52) Histone
NbS00009197g0102.1 **Mass:** 11420 **Score:** 226 **Matches:** 12(11) **Sequences:** 5(5)
NbS00009197g0102.1 protein AED:0.26 eAED:0.26 QI:0|-1|0|1|-1|1|1|0|103
NbS00010526g0004.1 **Mass:** 11402 **Score:** 226 **Matches:** 12(11) **Sequences:** 5(5)
NbS00010526g0004.1 protein AED:0.35 eAED:0.35 QI:0|-1|0|1|-1|1|1|0|103; (*GB) gi|195617694|gb|ACG30677.1| (e_value=1e-51) histone H4 [Zea mays];; (*SWP) sp|P0CG89|H4_SOYBN (e_value=1e-52) Histone H
NbS00013276g0001.1 **Mass:** 11402 **Score:** 226 **Matches:** 12(11) **Sequences:** 5(5)
NbS00013276g0001.1 protein AED:0.23 eAED:0.23 QI:0|-1|0|1|-1|1|1|0|103; (*SWP) sp|P0CG89|H4_SOYBN (e_value=1e-52) Histone H4 OS=Glycine max PE=3 SV=1;; (*TAIR) AT5G59970.1 (e_value=9e-54) | Symbols
NbS00033043g0001.1 **Mass:** 11402 **Score:** 226 **Matches:** 12(11) **Sequences:** 5(5)
NbS00033043g0001.1 protein AED:0.23 eAED:0.23 QI:0|-1|0|1|-1|1|1|0|103; (*GB) gi|195617694|gb|ACG30677.1| (e_value=1e-51) histone H4 [Zea mays];; (*SWP) sp|P0CG89|H4_SOYBN (e_value=1e-52) Histone H
NbS00034995g0002.1 **Mass:** 11748 **Score:** 226 **Matches:** 12(11) **Sequences:** 5(5)
NbS00034995g0002.1 protein AED:0.23 eAED:0.23 QI:37|0|0|0.5|1|1|2|0|106; (*GB) gi|308799807|ref|XP_003074685.1| (e_value=1e-50) Histone H4 (ISS) [Ostreococcus tauri];; (*SWP) sp|P0CG89|H4_SOYBN (e_
NbS00037466g0001.1 **Mass:** 11402 **Score:** 226 **Matches:** 12(11) **Sequences:** 5(5)
NbS00037466g0001.1 protein AED:0.28 eAED:0.28 QI:0|-1|0|1|-1|1|1|0|103; (*GB) gi|195617694|gb|ACG30677.1| (e_value=1e-51) histone H4 [Zea mays];; (*SWP) sp|P0CG89|H4_SOYBN (e_value=1e-52) Histone H
NbS00045876g0001.1 **Mass:** 11402 **Score:** 226 **Matches:** 12(11) **Sequences:** 5(5)
NbS00045876g0001.1 protein AED:0.00 eAED:0.00 QI:0|-1|0|1|-1|1|1|0|103; (*GB) gi|195617694|gb|ACG30677.1| (e_value=1e-51) histone H4 [Zea mays];; (*SWP) sp|P0CG89|H4_SOYBN (e_value=1e-52) Histone H
NbS00048006g0001.1 **Mass:** 11402 **Score:** 226 **Matches:** 12(11) **Sequences:** 5(5)
NbS00048006g0001.1 protein AED:0.09 eAED:0.10 QI:282|0|0.5|1|0|0.5|2|0|103; (*GB) gi|195617694|gb|ACG30677.1| (e_value=1e-51) histone H4 [Zea mays];; (*SWP) sp|P0CG89|H4_SOYBN (e_value=1e-52) Histo
NbS00056894g0001.1 **Mass:** 11402 **Score:** 226 **Matches:** 12(11) **Sequences:** 5(5)
NbS00056894g0001.1 protein AED:0.20 eAED:0.20 QI:0|-1|0|1|-1|1|1|0|103; (*SWP) sp|P0CG89|H4_SOYBN (e_value=1e-52) Histone H4 OS=Glycine max PE=3 SV=1;; (*TAIR) AT5G59970.1 (e_value=9e-54) | Symbols
NbC25918569g0001.1 **Mass:** 11402 **Score:** 226 **Matches:** 12(11) **Sequences:** 5(5)
NbC25918569g0001.1 protein AED:0.00 eAED:0.00 QI:0|-1|0|1|-1|1|1|0|103; (*GB) gi|195617694|gb|ACG30677.1| (e_value=1e-51) histone H4 [Zea mays];; (*SWP) sp|P0CG89|H4_SOYBN (e_value=1e-52) Histone H
NbC25948449g0001.1 **Mass:** 11402 **Score:** 226 **Matches:** 12(11) **Sequences:** 5(5)
NbC25948449g0001.1 protein AED:0.24 eAED:0.24 QI:0|-1|0|1|-1|1|1|0|103; (*GB) gi|195617694|gb|ACG30677.1| (e_value=1e-51) histone H4 [Zea mays];; (*SWP) sp|P0CG89|H4_SOYBN (e_value=1e-52) Histone H
NbS00001548g0007.1 **Mass:** 22455 **Score:** 226 **Matches:** 12(11) **Sequences:** 5(5)
NbS00001548g0007.1 protein AED:0.25 eAED:0.25 QI:0|0|0|1|0|0.5|2|0|202; (*SWP) sp|P0CG89|H4_SOYBN (e_value=2e-50) Histone H4 OS=Glycine max PE=3 SV=1;; (*TAIR) AT5G59970.1 (e_value=2e-51) | Symbols
NbS00046909g0009.1 **Mass:** 26576 **Score:** 226 **Matches:** 12(11) **Sequences:** 5(5)
NbS00046909g0009.1 protein AED:0.12 eAED:0.15 QI:52|0|0|1|0|0.33|3|151|237; (*GB) gi|195617694|gb|ACG30677.1| (e_value=4e-50) histone H4 [Zea mays];; (*SWP) sp|P0CG89|H4_SOYBN (e_value=8e-51) Histo

---

8. NbS00056353g0008.1 **Mass:** 62335 **Score:** 222 **Matches:** 14(13) **Sequences:** 10(9) **emPAI:** 0.67
NbS00056353g0008.1 protein AED:0.25 eAED:0.25 QI:0|0.92|0.85|1|0.92|0.92|14|0|554; (*SWP) sp|Q9FNX5|DRP1E_ARATH (e_value=0.0) Dynamin-related protein 1E OS=Arabidopsis thaliana GN=DRP1E PE=1 SV=1;;

Query	Observed	Mr(expt)	Mr(calc)	ppm	Miss	Score	Expect	Rank	Unique	Peptide
7523	**491.2400**	**980.4654**	**980.4749**	**-9.67**	**0**	**43**	**0.0067**	**1**		**K.NVDMIYAR.R**

12232	543.3323	1084.6500	1084.6492	0.75	0	54	0.00015	1		R.IPSITSLINK.S 12231
13097	595.2956	1188.5766	1188.5775	-0.73	0	42	0.0049	1	U	R.DEIDSVAWVR.- 13096
14344	440.2511	1317.7316	1317.7306	0.73	0	35	0.018	1		R.LQHPWVGIVNR.S 14342
14747	682.3403	1362.6660	1362.6627	2.43	1	31	0.063	1		K.EISDETDRITGK.T
16383	787.9161	1573.8177	1573.8140	2.35	0	58	0.00011	1		K.QSLLNYFYTQIGK.K
16395	788.9288	1575.8431	1575.8409	1.40	0	39	0.0076	1		R.IYGVFDNQLPAALR.K
17499	843.3942	1684.7738	1684.7733	0.29	0	36	0.011	1		R.EYFATSPDYGHLASK.M
18420	620.6567	1858.9484	1858.9458	1.37	1	42	0.0035	1		K.LDLMDKGTNALDVLEGR.A 18419
18440	621.6155	1861.8246	1861.8231	0.81	0	39	0.0043	1		K.TDDGQQEYAEFGHLPR.R

---

9. NbS00000987g0004.1 **Mass:** 28363 **Score:** 221 **Matches:** 8(8) **Sequences:** 4(4) **emPAI:** 0.74

NbS00000987g0004.1 protein AED:0.21 eAED:0.21 QI:0|-1|0|1|-1|1|1|0|266; (*GB) gi|3036948|dbj|BAA25392.1| (e_value=0.0) light harvesting chlorophyll a/b-binding protein [Nicotiana sylvestris];; (*SW

Query	Observed	Mr(expt)	Mr(calc)	ppm	Miss	Score	Expect	Rank	Unique	Peptide
11414	492.2528	982.4910	982.4913	-0.26	0	61	4.6e-005	1		K.FGEAVWFK.A 7535 7536
11982	528.2692	1054.5238	1054.5229	0.79	0	36	0.015	1		R.ELEVIHCR.W
16569	535.2670	1602.7793	1602.7791	0.13	0	(40)	0.0061	1	U	K.AKPVSSGSPWYGPDR.V
16570	802.3972	1602.7798	1602.7791	0.44	0	45	0.0023	1	U	K.AKPVSSGSPWYGPDR.V 16571
18662	952.5016	1902.9887	1902.9848	2.06	0	87	1.1e-007	1	U	R.WAMLGALGCVFPELLAR.N

Proteins matching the same set of peptides:

NbS00002523g0102.1 **Mass:** 28567 **Score:** 221 **Matches:** 8(8) **Sequences:** 4(4)
NbS00002523g0102.1 protein AED:0.30 eAED:0.30 QI:0|-1|0|1|-1|1|1|0|267
NbS00002523g0104.1 **Mass:** 28492 **Score:** 221 **Matches:** 8(8) **Sequences:** 4(4)
NbS00002523g0104.1 protein AED:0.30 eAED:0.30 QI:0|-1|0|1|-1|1|1|0|267
NbS00003625g0001.1 **Mass:** 28598 **Score:** 221 **Matches:** 8(8) **Sequences:** 4(4)
NbS00003625g0001.1 protein AED:0.17 eAED:0.17 QI:0|-1|0|1|-1|1|1|0|267; (*GB) gi|3036955|dbj|BAA25396.1| (e_value=0.0) light harvesting chlorophyll a/b-binding protein [Nicotiana sylvestris];; (*SW
NbS00003625g0003.1 **Mass:** 28448 **Score:** 221 **Matches:** 8(8) **Sequences:** 4(4)
NbS00003625g0003.1 protein AED:0.19 eAED:0.19 QI:0|-1|0|1|-1|1|1|0|267; (*GB) gi|3036955|dbj|BAA25396.1| (e_value=0.0) light harvesting chlorophyll a/b-binding protein [Nicotiana sylvestris];; (*SW
NbS00006290g0001.1 **Mass:** 28564 **Score:** 221 **Matches:** 8(8) **Sequences:** 4(4)
NbS00006290g0001.1 protein AED:0.21 eAED:0.21 QI:0|-1|0|1|-1|1|1|0|267; (*GB) gi|3036955|dbj|BAA25396.1| (e_value=0.0) light harvesting chlorophyll a/b-binding protein [Nicotiana sylvestris];; (*SW
NbS00009017g0002.1 **Mass:** 28464 **Score:** 221 **Matches:** 8(8) **Sequences:** 4(4)
NbS00009017g0002.1 protein AED:0.21 eAED:0.21 QI:0|-1|0|1|-1|1|1|0|267; (*GB) gi|3036955|dbj|BAA25396.1| (e_value=0.0) light harvesting chlorophyll a/b-binding protein [Nicotiana sylvestris];; (*SW
NbS00016807g0007.1 **Mass:** 33516 **Score:** 221 **Matches:** 8(8) **Sequences:** 4(4)
NbS00016807g0007.1 protein AED:0.33 eAED:0.40 QI:0|0|0|1|0.5|0.66|3|0|315; (*GB) gi|3036955|dbj|BAA25396.1| (e_value=1e-167) light harvesting chlorophyll a/b-binding protein [Nicotiana sylvestris];
NbS00046604g0001.1 **Mass:** 28532 **Score:** 221 **Matches:** 8(8) **Sequences:** 4(4)
NbS00046604g0001.1 protein AED:0.31 eAED:0.31 QI:0|-1|0|1|-1|1|1|0|267; (*GB) gi|115825|sp|P07369.1|CB2G_SOLLC (e_value=0.0) RecName: Full=Chlorophyll a-b binding protein 3C, chloroplastic; AltName
NbC25792767g0001.1 **Mass:** 15882 **Score:** 221 **Matches:** 8(8) **Sequences:** 4(4)
NbC25792767g0001.1 protein AED:0.05 eAED:0.05 QI:0|-1|1|1|-1|1|1|0|149; (*GB) gi|3036953|dbj|BAA25395.1| (e_value=3e-103) light harvesting chlorophyll a/b-binding protein [Nicotiana sylvestris];;
NbS00016045g0013.1 **Mass:** 61743 **Score:** 221 **Matches:** 8(8) **Sequences:** 4(4)
NbS00016045g0013.1 protein AED:0.20 eAED:0.21 QI:0|0|0|0.85|0.66|0.57|7|0|569; (*GB) gi|115805|sp|P27495.1|CB24_TOBAC (e_value=2e-158) RecName: Full=Chlorophyll a-b binding protein 40, chloroplast:

---

10. NbS00000867g0011.1 **Mass:** 30015 **Score:** 203 **Matches:** 7(7) **Sequences:** 6(6) **emPAI:** 0.88

NbS00000867g0011.1 protein AED:0.34 eAED:0.34 QI:171|0.9|0.90|1|0.7|0.63|11|450|270; (*GB) gi|359492084|ref|XP_002284740.2| (e_value=1e-70) PREDICTED: uncharacterized protein LOC100254082 [Vitis v:

Query	Observed	Mr(expt)	Mr(calc)	ppm	Miss	Score	Expect	Rank	Unique	Peptide
10611	447.7323	893.4500	893.4494	0.67	0	49	0.0011	1	U	K.GSDEIVFK.A
14024	643.8152	1285.6159	1285.6150	0.75	0	33	0.031	1	U	R.AETPIDENEIR.I
16305	780.9033	1559.7920	1559.7905	0.94	0	39	0.0076	1	U	R.SYITYAMTLLQEK.G 16304
17991	886.3939	1770.7732	1770.7697	1.96	0	82	1.9e-007	1	U	K.VLTDFDYDGEGSPSGGR.R
19339	1014.9046	2027.7947	2027.7956	-0.46	0	47	0.0001	1	U	R.GFAGNGFMSAEYDDGGYDR.N
21148	1226.9807	3677.9203	3677.9040	4.44	0	51	0.00013	1		R.IVGLHQITSITSTDITDTWEPLEEGLLPLETTR.H

Proteins matching the same set of peptides:

NbS00030156g0009.1 **Mass:** 31162 **Score:** 203 **Matches:** 7(7) **Sequences:** 6(6)
NbS00030156g0009.1 protein AED:0.29 eAED:0.29 QI:170|1|0.9|1|0.77|0.7|10|232|281; (*GB) gi|358248282|ref|NP_001239854.1| (e_value=2e-70) uncharacterized protein LOC100818986 [Glycine max];; (*SWP)

---

11. NbS00054358g0003.1 **Mass:** 62948 **Score:** 200 **Matches:** 13(12) **Sequences:** 10(9) **emPAI:** 0.66

NbS00054358g0003.1 protein AED:0.12 eAED:0.14 QI:177|0.91|0.84|1|0.91|0.84|13|0|563; (*GB) gi|359489241|ref|XP_003633899.1| (e_value=0.0) PREDICTED: dynamin-related protein 1E [Vitis vinifera];; (

Query	Observed	Mr(expt)	Mr(calc)	ppm	Miss	Score	Expect	Rank	Unique	Peptide
7523	491.2400	980.4654	980.4749	-9.67	0	43	0.0067	1		K.NVDMIYAR.R
12032	530.8037	1059.5929	1059.5924	0.44	0	35	0.022	1	U	K.SSVLESIVAR.D
12232	543.3323	1084.6500	1084.6492	0.75	0	54	0.00015	1		R.IPSITSLINK.S 12231
14344	440.2511	1317.7316	1317.7306	0.73	0	35	0.018	1		R.LQHPWVGIVNR.S 14342
14747	682.3403	1362.6660	1362.6627	2.43	1	31	0.063	1		K.EISDETDRITGK.T
16383	787.9161	1573.8177	1573.8140	2.35	0	58	0.00011	1		K.QSLLNYFYTQIGK.K
16395	788.9288	1575.8431	1575.8409	1.40	0	39	0.0076	1		R.IYGVFDNQLPAALR.K
17499	843.3942	1684.7738	1684.7733	0.29	0	36	0.011	1		R.EYFATSPDYGHLASK.M
18420	620.6567	1858.9484	1858.9458	1.37	1	42	0.0035	1		K.LDLMDKGTNALDVLEGR.A 18419
18440	621.6155	1861.8246	1861.8231	0.81	0	39	0.0043	1		K.TDDGQQEYAEFGHLPR.R

---

12. NbS00000471g0009.1 **Mass:** 32669 **Score:** 194 **Matches:** 7(7) **Sequences:** 4(4) **emPAI:** 0.47

NbS00000471g0009.1 protein AED:0.15 eAED:0.15 QI:0|0|0.33|1|1|1|3|0|301; (*GB) gi|115802|sp|P27494.1|CB23_TOBAC (e_value=0.0) RecName: Full=Chlorophyll a-b binding protein 36, chloroplastic; AltNar

Query	Observed	Mr(expt)	Mr(calc)	ppm	Miss	Score	Expect	Rank	Unique	Peptide
11414	492.2528	982.4910	982.4913	-0.26	0	61	4.6e-005	1		K.FGEAVWFK.A 7535 7536
11982	528.2692	1054.5238	1054.5229	0.79	0	36	0.015	1		R.ELEVIHCR.W
12341	549.7931	1097.5716	1097.5717	-0.06	0	59	8.6e-005	1	U	R.VGGGPLGEGLDK.I 12343
20322	802.7346	2405.1820	2405.1791	1.23	0	65	1.3e-005	1	U	K.IYPGGAFDPLGLADDPEAFAELK.V

Proteins matching the same set of peptides:

NbS00032129g0003.1 **Mass:** 28757 **Score:** 194 **Matches:** 7(7) **Sequences:** 4(4)

NbS00032129g0003.1 protein AED:0.10 eAED:0.10 QI:62|1|1|1|1|1|2|290|265; (*SWP) sp|P12062|CB26_PETSP (e_value=0.0) Chlorophyll a-b binding protein 37, chloroplastic OS=Petunia sp. GN=CAB37 PE=3 SV=

NbS00050671g0012.1 **Mass:** 28761 **Score:** 194 **Matches:** 7(7) **Sequences:** 4(4)

NbS00050671g0012.1 protein AED:0.04 eAED:0.04 QI:0|1|0.5|1|1|1|2|0|265; (*GB) gi|115802|sp|P27494.1|CB23_TOBAC (e_value=0.0) RecName: Full=Chlorophyll a-b binding protein 36, chloroplastic; AltName

---

13. NbS00017066g0003.1 **Mass:** 21609 **Score:** 188 **Matches:** 3(3) **Sequences:** 2(2) **emPAI:** 0.54

NbS00017066g0003.1 protein AED:0.01 eAED:0.01 QI:0|-1|0|1|-1|1|1|0|208; (*GB) gi|222051768|dbj|BAH15357.1| (e_value=3e-127) germin like protein [Nicotiana tabacum];; (*SWP) sp|Q9ZRA4|AB19A_PRUPE (e

Query	Observed	Mr(expt)	Mr(calc)	ppm	Miss	Score	Expect	Rank	Unique	Peptide
16100	775.9189	1549.8232	1549.8239	-0.46	0	84	2.5e-007	1	U	K.LVEATTFLDEATIK.K
19826	734.0677	2199.1812	2199.1800	0.55	0	75	9.2e-007	1	U	K.AAVTPAFAAQFPGLNGLGLSAAR.L
19828	1100.5981	2199.1817	2199.1800	0.78	0	(71)	2.7e-006	1	U	K.AAVTPAFAAQFPGLNGLGLSAAR.L

Proteins matching the same set of peptides:

NbS00021913g0003.1 **Mass:** 21582 **Score:** 188 **Matches:** 3(3) **Sequences:** 2(2)

NbS00021913g0003.1 protein AED:0.01 eAED:0.01 QI:0|-1|0|1|-1|1|1|0|208; (*GB) gi|222051768|dbj|BAH15357.1| (e_value=6e-127) germin like protein [Nicotiana tabacum];; (*SWP) sp|Q9ZRA4|AB19A_PRUPE (e

---

14. NbS00021492g0001.1 **Mass:** 72107 **Score:** 174 **Matches:** 10(9) **Sequences:** 7(7) **emPAI:** 0.37

NbS00021492g0001.1 protein AED:0.22 eAED:0.25 QI:0|0.70|0.72|0.94|0.94|0.88|18|255|639; (*SWP) sp|Q39828|SDL5A_SOYBN (e_value=0.0) Dynamin-related protein 5A OS=Glycine max PE=2 SV=1;; (*TAIR) AT5(

Query	Observed	Mr(expt)	Mr(calc)	ppm	Miss	Score	Expect	Rank	Unique	Peptide
10882	463.7400	925.4655	925.4658	-0.31	0	31	0.042	1	U	R.FTDFAAVR.K
8965	658.8600	1315.7054	1315.6983	5.43	0	45	0.0026	1	U	K.TIIELENELSR.L 14318
14445	664.8800	1327.7454	1327.7401	4.03	0	37	0.0094	1		K.LQFPWIGVVNR.S
14562	673.3413	1344.6681	1344.6674	0.53	0	45	0.0024	1	U	R.AAQGEIDSVAWAK.- 14561
14796	686.8572	1371.6998	1371.6994	0.30	0	56	0.00016	1	U	R.VEVNGAAVESLER.M 14795
17218	549.6428	1645.9065	1645.9039	1.54	1	32	0.028	1	U	K.SSVLESIVGKDFLPR.G
17574	567.2951	1698.8635	1698.8611	1.43	0	33	0.028	1	U	K.QLGTLLDEDPAIMQR.R

---

15. NbS00001169g0153.1 **Mass:** 36448 **Score:** 162 **Matches:** 8(8) **Sequences:** 6(6) **emPAI:** 0.69

NbS00001169g0153.1 protein AED:0.25 eAED:0.25 QI:0|0.5|0.4|1|1|1|5|0|341

Query	Observed	Mr(expt)	Mr(calc)	ppm	Miss	Score	Expect	Rank	Unique	Peptide
10179	417.2351	832.4557	832.4555	0.23	0	37	0.019	1		K.VAINGFGR.I
11635	505.8340	1009.6535	1009.6535	-0.07	0	48	0.0001	1	U	K.AVALVLPSLK.G 11634
13639	627.8104	1253.6062	1253.6040	1.71	0	44	0.0026	1	U	K.TFAEEVNAAFR.E 13640

14892	692.8936	1383.7727	1383.7722	0.38	0	41	0.0037	1		R.AAALNIVPTSTGAAK.A
16847	807.4235	1612.8325	1612.8308	1.03	0	57	0.00013	1	U	K.DSPLDVIAINDTGGVK.Q
17789	581.3165	1740.9276	1740.9258	1.03	1	41	0.0036	1	U	R.KDSPLDVIAINDTGGVK.Q

---

16. NbS00010009g0002.1 **Mass:** 118617 **Score:** 142 **Matches:** 6(6) **Sequences:** 5(5) **emPAI:** 0.18

NbS00010009g0002.1 protein AED:0.20 eAED:0.20 QI:0|1|1|1|1|1|21|408|1058; (*SWP) sp|O04379|AGO1_ARATH (e_value=0.0) Protein argonaute 1 OS=Arabidopsis thaliana GN=AGO1 PE=1 SV=1;; (*TAIR) AT1G4841(

Query	Observed	Mr(expt)	Mr(calc)	ppm	Miss	Score	Expect	Rank	Unique	Peptide
12256	544.3094	1086.6043	1086.6033	0.95	0	49	0.001	1	U	R.NTVLVDALSR.R
12948	391.5440	1171.6101	1171.6098	0.27	0	38	0.01	1		K.YAGLVSAQAHR.Q
15128	707.8444	1413.6743	1413.6736	0.49	0	47	0.001	1	U	K.ITLIDDDDGPGAR.R
15226	476.5845	1426.7318	1426.7317	0.06	1	32	0.041	1		R.RQPLGEGLESWR.G
18548	627.0142	1878.0208	1878.0211	-0.12	0	(45)	0.0012	1		R.QADAPQEALQVLDIVLR.E
18549	940.0191	1878.0237	1878.0211	1.38	0	72	2.4e-006	1		R.QADAPQEALQVLDIVLR.E

Proteins matching the same set of peptides:

NbS00034990g0010.1 **Mass:** 119669 **Score:** 142 **Matches:** 6(6) **Sequences:** 5(5)

NbS00034990g0010.1 protein AED:0.15 eAED:0.15 QI:0|0.95|0.95|1|0.95|0.90|21|286|1067; (*GB) gi|84688906|gb|ABC61502.1| (e_value=0.0) AGO1-1, partial [Nicotiana benthamiana];; (*SWP) sp|Q7XSA2|AGO1I

---

17. NbS00033686g0006.1 **Mass:** 69818 **Score:** 141 **Matches:** 10(10) **Sequences:** 8(8) **emPAI:** 0.44

NbS00033686g0006.1 protein AED:0.16 eAED:0.16 QI:197|1|1|1|1|1|15|214|623; (*GB) gi|225453246|ref|XP_002265511.1| (e_value=0.0) PREDICTED: dynamin-related protein 1E isoform 1 [Vitis vinifera];; (

Query	Observed	Mr(expt)	Mr(calc)	ppm	Miss	Score	Expect	Rank	Unique	Peptide
7523	491.2400	980.4654	980.4749	-9.67	0	43	0.0067	1		K.NVDMIYAR.R
11545	499.7679	997.5212	997.5233	-2.09	0	32	0.037	1	U	R.FTDFTLVR.R
13143	598.3274	1194.6402	1194.6397	0.46	0	35	0.018	1	U	R.LIEGALNYFR.G
14344	440.2511	1317.7316	1317.7306	0.73	0	35	0.018	1		R.LQHPWVGIVNR.S 14342
16024	770.4088	1538.8031	1538.8093	-4.04	0	39	0.0074	1	U	R.GPAEASVDAVHFVLK.E
16395	788.9288	1575.8431	1575.8409	1.40	0	39	0.0076	1		R.IYGVFDNQLPAALR.K
17307	829.4229	1656.8313	1656.8280	1.98	0	35	0.02	1	U	K.ALAELLDEDPALMEK.R
18420	620.6567	1858.9484	1858.9458	1.37	1	42	0.0035	1		K.LDLMDKGTNALDVLEGR.A 18419

---

18. NbS00014977g0008.1 **Mass:** 125165 **Score:** 125 **Matches:** 6(6) **Sequences:** 5(5) **emPAI:** 0.17

NbS00014977g0008.1 protein AED:0.16 eAED:0.16 QI:16|0.95|0.90|1|0.95|0.90|22|432|1125; (*GB) gi|84688908|gb|ABC61503.1| (e_value=0.0) AGO1-2, partial [Nicotiana benthamiana];; (*SWP) sp|Q7XSA2|AGO1

Query	Observed	Mr(expt)	Mr(calc)	ppm	Miss	Score	Expect	Rank	Unique	Peptide
12256	544.3094	1086.6043	1086.6033	0.95	0	49	0.001	1	U	R.NTVLVDAISR.R
12948	391.5440	1171.6101	1171.6098	0.27	0	38	0.01	1		K.YAGLVSAQAHR.Q
15226	476.5845	1426.7318	1426.7317	0.06	1	32	0.041	1		R.RQPLGEGLESWR.G
16449	528.9374	1583.7903	1583.7903	-0.02	1	34	0.026	1	U	K.ITLIDDEDGPGARR.E
18548	627.0142	1878.0208	1878.0211	-0.12	0	(45)	0.0012	1		K.QADAPQEALQVLDIVLR.E
18549	940.0191	1878.0237	1878.0211	1.38	0	72	2.4e-006	1		K.QADAPQEALQVLDIVLR.E

---

19. NbS00010295g0007.1 **Mass:** 13224 **Score:** 125 **Matches:** 3(3) **Sequences:** 1(1) **emPAI:** 0.26

NbS00010295g0007.1 protein AED:0.12 eAED:0.12 QI:0|0|0|0.5|1|1|2|0|119; (*SWP) sp|P69569|RBL_EUPES (e_value=5e-22) Ribulose bisphosphate carboxylase large chain (Fragment) OS=Euphorbia esula GN=rbc

Query	Observed	Mr(expt)	Mr(calc)	ppm	Miss	Score	Expect	Rank	Unique	Peptide
11726	511.2682	1020.5218	1020.5240	-2.13	0	74	3.7e-006	1	U	K.DTDILAAFR.V 11729 11731

Proteins matching the same set of peptides:

NbS00047753g0001.1 **Mass:** 13332 **Score:** 125 **Matches:** 3(3) **Sequences:** 1(1)

NbS00047753g0001.1 protein AED:0.28 eAED:0.28 QI:0|0|0|0.5|1|0|2|0|115; (*GB) gi|306481964|dbj|BAJ17086.1| (e_value=9e-37) ribulose-1,5-bisphosphate carboxylase/oxygenase large subunit [Solanum sp.

---

20. NbS00000681g0006.1 **Mass:** 33521 **Score:** 119 **Matches:** 4(4) **Sequences:** 3(3) **emPAI:** 0.33

NbS00000681g0006.1 protein AED:0.29 eAED:0.30 QI:89|0.75|0.88|1|0.75|0.77|9|282|301; (*GB) gi|358248282|ref|NP_001239854.1| (e_value=7e-62) uncharacterized protein LOC100818986 [Glycine max];; (*TA

Query	Observed	Mr(expt)	Mr(calc)	ppm	Miss	Score	Expect	Rank	Unique	Peptide
13658	628.8279	1255.6413	1255.6408	0.42	0	53	0.00037	1	U	R.VETPIDANEIR.I 13659
20445	1233.6110	2465.2074	2465.2036	1.54	0	38	0.0065	1	U	R.SYITYAMTLLQENTSDEIVFK.A
21148	1226.9807	3677.9203	3677.9040	4.44	0	51	0.00013	1		R.IVGLHQITSITSTDITDTWEPLEEGLLPLETTR.H

Proteins matching the same set of peptides:
NbS00014238g0013.1 **Mass:** 27940 **Score:** 119 **Matches:** 4(4) **Sequences:** 3(3)
NbS00014238g0013.1 protein AED:0.37 eAED:0.37 QI:89|1|1|1|0.87|0.88|9|404|254; (*GB) gi|358248282|ref|NP_001239854.1| (e_value=1e-64) uncharacterized protein LOC100818986 [Glycine max];; (*SWP) sp

21. NbS00004085g0015.1 **Mass:** 23594 **Score:** 119 **Matches:** 5(5) **Sequences:** 3(3) **emPAI:** 0.49
NbS00004085g0015.1 protein AED:0.29 eAED:0.29 QI:92|0.25|0.2|1|1|1|5|0|225; (*GB) gi|110377793|gb|ABG73417.1| (e_value=7e-87) chloroplast pigment-binding protein CP26 [Nicotiana tabacum];; (*SWP) s

Query	Observed	Mr(expt)	Mr(calc)	ppm	Miss	Score	Expect	Rank	Unique	Peptide
11306	485.7644	969.5143	969.5131	1.26	0	45	0.0018	1	U	K.DPDQAAILK.V
15637	742.3377	1482.6609	1482.6602	0.45	0	44	0.0016	1	U	K.FGADCGPEAVWFK.T
17551	848.9487	1695.8828	1695.8832	-0.23	0	49	0.00075	1	U	K.TGALLLDGNTLNYFGK.N 17552 17554

Proteins matching the same set of peptides:
NbS00008488g0031.1 **Mass:** 15157 **Score:** 119 **Matches:** 5(5) **Sequences:** 3(3)
NbS00008488g0031.1 protein AED:0.34 eAED:0.21 QI:689|0.75|0.4|1|0.75|0.8|5|0|143; (*GB) gi|261351266|gb|ACX71300.1| (e_value=1e-88) chloroplast pigment-binding protein CP26 [Capsicum annuum];; (*SW

22. NbS00001942g0006.1 **Mass:** 15769 **Score:** 109 **Matches:** 5(5) **Sequences:** 2(2) **emPAI:** 0.48
NbS00001942g0006.1 protein AED:0.00 eAED:0.00 QI:75|1|1|1|1|1|2|198|151; (*GB) gi|351725389|ref|NP_001235555.1| (e_value=1e-63) uncharacterized protein LOC100305705 [Glycine max];; (*SWP) sp|Q6L50(

Query	Observed	Mr(expt)	Mr(calc)	ppm	Miss	Score	Expect	Rank	Unique	Peptide
9977	404.2633	806.5121	806.5127	-0.66	0	38	0.0019	1	U	R.HVLLAVR.N 9979 9982
11041	472.7688	943.5230	943.5240	-0.96	0	71	8e-006	1		K.AGLQFPVGR.I 11042

Proteins matching the same set of peptides:
NbS00008178g0018.1 **Mass:** 15524 **Score:** 109 **Matches:** 5(5) **Sequences:** 2(2)
NbS00008178g0018.1 protein AED:0.03 eAED:0.03 QI:116|1|1|1|1|1|2|157|148; (*GB) gi|27529852|dbj|BAC53941.1| (e_value=4e-56) H2A histone [Nicotiana tabacum];; (*SWP) sp|P25469|H2A1_SOLLC (e_value=1e
NbS00010297g0001.1 **Mass:** 15303 **Score:** 109 **Matches:** 5(5) **Sequences:** 2(2)
NbS00010297g0001.1 protein AED:0.04 eAED:0.04 QI:122|1|1|1|1|1|2|350|144; (*GB) gi|225439749|ref|XP_002274570.1| (e_value=6e-44) PREDICTED: probable histone H2A.4 isoform 1 [Vitis vinifera];; (*SWI
NbS00012269g0005.1 **Mass:** 15797 **Score:** 109 **Matches:** 5(5) **Sequences:** 2(2)
NbS00012269g0005.1 protein AED:0.02 eAED:0.02 QI:75|1|1|1|1|1|2|181|151; (*GB) gi|351725389|ref|NP_001235555.1| (e_value=1e-63) uncharacterized protein LOC100305705 [Glycine max];; (*SWP) sp|Q2HU6
NbS00020235g0008.1 **Mass:** 15471 **Score:** 109 **Matches:** 5(5) **Sequences:** 2(2)
NbS00020235g0008.1 protein AED:0.06 eAED:0.06 QI:118|1|1|1|1|1|2|157|148; (*GB) gi|27529852|dbj|BAC53941.1| (e_value=7e-58) H2A histone [Nicotiana tabacum];; (*SWP) sp|P25469|H2A1_SOLLC (e_value=3e
NbS00024367g0008.1 **Mass:** 15926 **Score:** 109 **Matches:** 5(5) **Sequences:** 2(2)
NbS00024367g0008.1 protein AED:0.03 eAED:0.03 QI:74|1|1|1|1|1|2|309|151; (*GB) gi|255584772|ref|XP_002533104.1| (e_value=5e-54) histone h2a, putative [Ricinus communis];; (*SWP) sp|Q2HU65|H2A2_MED
NbS00032978g0018.1 **Mass:** 15912 **Score:** 109 **Matches:** 5(5) **Sequences:** 2(2)
NbS00032978g0018.1 protein AED:0.04 eAED:0.04 QI:72|1|1|1|1|1|2|205|151; (*GB) gi|6009909|dbj|BAA85117.1| (e_value=1e-53) histone H2A-like protein [Solanum melongena];; (*SWP) sp|Q2HU65|H2A2_MEDTR
NbS00056349g0008.1 **Mass:** 15304 **Score:** 109 **Matches:** 5(5) **Sequences:** 2(2)
NbS00056349g0008.1 protein AED:0.02 eAED:0.02 QI:262|1|1|1|1|1|2|424|145; (*GB) gi|225439749|ref|XP_002274570.1| (e_value=2e-53) PREDICTED: probable histone H2A.4 isoform 1 [Vitis vinifera];; (*SWI

23. NbS00006820g0015.1 **Mass:** 29146 **Score:** 107 **Matches:** 4(4) **Sequences:** 3(3) **emPAI:** 0.54
NbS00006820g0015.1 protein AED:0.07 eAED:0.08 QI:0|1|0.66|1|1|1|3|0|273; (*GB) gi|226872|prf||1609235A (e_value=1e-149) chlorophyll a/b binding protein;; (*SWP) sp|P27522|CB13_SOLLC (e_value=3e-15(

Query	Observed	Mr(expt)	Mr(calc)	ppm	Miss	Score	Expect	Rank	Unique	Peptide
11531	499.2714	996.5282	996.5280	0.16	0	43	0.0026	1	U	K.QYFLGLEK.G
17101	815.4557	1628.8969	1628.8960	0.60	0	(53)	0.0002	1	U	R.FAMLGAAGAIAPEILGK.A
17210	823.4538	1644.8930	1644.8909	1.31	0	57	9.7e-005	1	U	R.FAMLGAAGAIAPEILGK.A
20591	861.0847	2580.2321	2580.2285	1.40	1	32	0.027	1	U	K.GLGGSGDPAYPGGPFFNPLGFGKDEK.S

Proteins matching the same set of peptides:
NbS00021892g0003.1 **Mass:** 26702 **Score:** 107 **Matches:** 4(4) **Sequences:** 3(3)
NbS00021892g0003.1 protein AED:0.04 eAED:0.04 QI:191|1|1|1|0.5|0.4|5|817|252; (*GB) gi|226872|prf||1609235A (e_value=2e-124) chlorophyll a/b binding protein;; (*SWP) sp|P27522|CB13_SOLLC (e_value=

24. NbS00002621g0212.1 **Mass:** 26223 **Score:** 103 **Matches:** 4(4) **Sequences:** 3(3) **emPAI:** 0.43
NbS00002621g0212.1 protein AED:0.19 eAED:0.19 QI:122|0.8|1|1|1|1|6|306|255

Query	Observed	Mr(expt)	Mr(calc)	ppm	Miss	Score	Expect	Rank	Unique	Peptide
13629	626.8693	1251.7241	1251.7227	1.14	0	39	0.0041	1	U	R.ILALNASYFLK.A 13627

18614	632.0318	1893.0736	1893.0724	0.63	0	49	0.00024	1	U	K.LAAAVLGGVDDIWIKPGAK.V
19393	1025.0031	2047.9916	2047.9884	1.52	0	51	0.00042	1	U	K.ANCIDSTVPAEAVFAQEVK.K

Proteins matching the same set of peptides:
NbS00030218g0006.1 **Mass:** 26271 **Score:** 103 **Matches:** 4(4) **Sequences:** 3(3)
NbS00030218g0006.1 protein AED:0.28 eAED:0.28 QI:126|0.83|1|1|0.83|0.71|7|182|255; (*SWP) sp|Q22053|FBRL_CAEEL (e_value=1e-70) rRNA 2'-O-methyltransferase fibrillarin OS=Caenorhabditis elegans GN=

---

25. NbS00007993g0305.1 **Mass:** 30118 **Score:** 101 **Matches:** 5(5) **Sequences:** 3(3) **emPAI:** 0.37
NbS00007993g0305.1 protein AED:0.00 eAED:0.00 QI:0|-1|0|1|-1|1|1|0|261

Query	Observed	Mr(expt)	Mr(calc)	ppm	Miss	Score	Expect	Rank	Unique	Peptide
12379	551.3289	1100.6433	1100.6441	-0.73	0	49	0.00066	1	U	K.VLAELLSASAK.D 12380
13056	592.3379	1182.6612	1182.6608	0.35	0	43	0.0022	1	U	K.NQITAEAIVPK.N 13057
17447	838.4257	1674.8368	1674.8334	2.03	0	32	0.038	1	U	R.YELYPFFFPLDPK.E

---

26. NbS00010743g0014.1 **Mass:** 32611 **Score:** 100 **Matches:** 3(3) **Sequences:** 3(3) **emPAI:** 0.34
NbS00010743g0014.1 protein AED:0.24 eAED:0.24 QI:0|1|1|1|1|1|8|290|288; (*GB) gi|77416949|gb|ABA81870.1| (e_value=2e-155) unknown [Solanum tuberosum];; (*SWP) sp|P43333|RU2A_ARATH (e_value=2e-117)

Query	Observed	Mr(expt)	Mr(calc)	ppm	Miss	Score	Expect	Rank	Unique	Peptide
15751	750.9049	1499.7953	1499.7943	0.61	0	32	0.033	1	U	K.AAIVNSQTLEEVAR.L
18379	925.5375	1849.0605	1849.0560	2.43	0	42	0.001	1	U	R.LTNLVEIDPLASLPNLK.F
18424	930.9714	1859.9282	1859.9265	0.92	0	70	6.2e-006	1	U	R.SGQLPADLNIGDYDIAAK.K

---

27. NbS00001988g0005.1 **Mass:** 66344 **Score:** 98 **Matches:** 4(4) **Sequences:** 4(4) **emPAI:** 0.21
NbS00001988g0005.1 protein AED:0.30 eAED:0.30 QI:0|1|0.83|1|1|1|6|0|610; (*GB) gi|225441549|ref|XP_002281113.1| (e_value=0.0) PREDICTED: DEAD-box ATP-dependent RNA helicase 37-like [Vitis vinifera

Query	Observed	Mr(expt)	Mr(calc)	ppm	Miss	Score	Expect	Rank	Unique	Peptide
10266	423.7468	845.4790	845.4793	-0.37	0	32	0.059	1	U	K.VSLQMIR.Y
5602	429.2600	856.5054	856.5018	4.26	0	36	0.027	1	U	R.LVDLLER.A
13091	594.8331	1187.6516	1187.6510	0.49	0	89	9.4e-008	1	U	R.VGSSTDLILQR.V
14489	668.8245	1335.6344	1335.6315	2.14	0	36	0.02	1		R.MLDMGFEPQIR.R

---

28. NbS00012584g0001.1 **Mass:** 40901 **Score:** 98 **Matches:** 4(4) **Sequences:** 3(3) **emPAI:** 0.26
NbS00012584g0001.1 protein AED:0.12 eAED:0.12 QI:0|-1|0|1|-1|1|1|0|370; (*GB) gi|231610|sp|P29790.1|ATPG_TOBAC (e_value=0.0) RecName: Full=ATP synthase gamma chain, chloroplastic; AltName: Full=F-

Query	Observed	Mr(expt)	Mr(calc)	ppm	Miss	Score	Expect	Rank	Unique	Peptide
7816	514.8100	1027.6054	1027.6026	2.76	0	36	0.012	1	U	K.VALVVVTGDR.G 11777
14694	679.8681	1357.7217	1357.7201	1.15	0	33	0.033	1	U	R.ALQESLASELAAR.M
16444	792.4489	1582.8832	1582.8818	0.87	0	53	0.00019	1	U	K.ITGEILEIVAGADALV.-

---

29. NbS00017935g0003.1 **Mass:** 31122 **Score:** 95 **Matches:** 5(5) **Sequences:** 4(4) **emPAI:** 0.50
NbS00017935g0003.1 protein AED:0.00 eAED:0.00 QI:240|1|0.5|1|1|1|2|0|284; (*SWP) sp|Q9XF88|CB4B_ARATH (e_value=4e-162) Chlorophyll a-b binding protein CP29.2, chloroplastic OS=Arabidopsis thaliana

Query	Observed	Mr(expt)	Mr(calc)	ppm	Miss	Score	Expect	Rank	Unique	Peptide
11447	493.7979	985.5812	985.5808	0.45	0	45	0.002	1	U	K.ATLQLAEIK.H 11448
11780	515.2881	1028.5617	1028.5614	0.29	0	40	0.0083	1	U	K.NLAGDIIGTR.T
17899	878.4393	1754.8640	1754.8628	0.68	0	54	0.00021	1	U	K.STPFQPYSEVFGLQR.F
19264	997.5004	1992.9863	1992.9833	1.51	0	34	0.022	1	U	R.LYPGGSFFDPLGLAADPEK.K

Proteins matching the same set of peptides:
NbS00027305g0008.1 **Mass:** 31084 **Score:** 95 **Matches:** 5(5) **Sequences:** 4(4)
NbS00027305g0008.1 protein AED:0.31 eAED:0.31 QI:0|1|0.5|1|1|1|2|0|284; (*GB) gi|110377766|gb|ABG73415.1| (e_value=0.0) chloroplast pigment-binding protein CP29 [Nicotiana tabacum];; (*SWP) sp|Q9X

---

30. NbS00004793g0016.1 **Mass:** 31922 **Score:** 94 **Matches:** 2(2) **Sequences:** 2(2) **emPAI:** 0.22
NbS00004793g0016.1 protein AED:0.15 eAED:0.27 QI:0|0|0|1|1|1|2|0|286; (*SWP) sp|P27492|CB21_TOBAC (e_value=9e-155) Chlorophyll a-b binding protein 16, chloroplastic OS=Nicotiana tabacum GN=CAB16 P

Query	Observed	Mr(expt)	Mr(calc)	ppm	Miss	Score	Expect	Rank	Unique	Peptide
11982	528.2692	1054.5238	1054.5229	0.79	0	36	0.015	1		R.ELEVIHCR.W
18662	952.5016	1902.9887	1902.9848	2.06	0	87	1.1e-007	1	U	R.WAMLGALGCVFPEILAR.N

31. NbS00000634g0101.1 **Mass:** 15316 **Score:** 93 **Matches:** 8(7) **Sequences:** 4(4) **emPAI:** 1.23
NbS00000634g0101.1 protein AED:0.21 eAED:0.21 QI:0|-1|0|1|-1|1|1|0|136

Query	Observed	Mr(expt)	Mr(calc)	ppm	Miss	Score	Expect	Rank	Unique	Peptide
9844	394.7427	787.4708	787.4704	0.45	1	32	0.044	1	U	R.KLPFQR.L 9839
10161	416.2501	830.4856	830.4861	-0.62	0	54	0.00029	1	U	K.STELLIR.K 10160
11686	339.5382	1015.5927	1015.5927	0.01	0	36	0.014	1	U	R.FRPGTVALR.E 11687 11691
14482	668.3504	1334.6863	1334.6830	2.43	1	54	0.00029	1	U	R.EIAQDFKTDLR.F

Proteins matching the same set of peptides:
NbS00002567g0001.1 **Mass:** 15316 **Score:** 93 **Matches:** 8(7) **Sequences:** 4(4)
NbS00002567g0001.1 protein AED:0.20 eAED:0.20 QI:0|-1|0|1|-1|1|1|0|136; (*GB) gi|300681467|emb|CBH32561.1| (e_value=8e-79) histone H3, expressed [Triticum aestivum];; (*SWP) sp|P68428|H32_WHEAT (e_
NbS00007322g0001.1 **Mass:** 15316 **Score:** 93 **Matches:** 8(7) **Sequences:** 4(4)
NbS00007322g0001.1 protein AED:0.09 eAED:0.09 QI:0|1|0.5|1|0|0|2|203|136; (*GB) gi|300681467|emb|CBH32561.1| (e_value=8e-79) histone H3, expressed [Triticum aestivum];; (*SWP) sp|P68428|H32_WHEAT
NbS00009503g0001.1 **Mass:** 15316 **Score:** 93 **Matches:** 8(7) **Sequences:** 4(4)
NbS00009503g0001.1 protein AED:0.19 eAED:0.19 QI:0|-1|0|1|-1|1|1|0|136; (*GB) gi|300681467|emb|CBH32561.1| (e_value=8e-79) histone H3, expressed [Triticum aestivum];; (*SWP) sp|P68428|H32_WHEAT (e_
NbS00010093g0001.1 **Mass:** 15316 **Score:** 93 **Matches:** 8(7) **Sequences:** 4(4)
NbS00010093g0001.1 protein AED:0.19 eAED:0.19 QI:0|-1|0|1|-1|1|1|0|136; (*SWP) sp|P68428|H32_WHEAT (e_value=3e-80) Histone H3.2 OS=Triticum aestivum PE=1 SV=2;; (*TAIR) AT5G65360.1 (e_value=2e-81)
NbS00014992g0013.1 **Mass:** 56465 **Score:** 93 **Matches:** 8(7) **Sequences:** 4(4)
NbS00014992g0013.1 protein AED:0.12 eAED:0.14 QI:0|0|0|0.66|1|1|3|0|505; (*GB) gi|351722020|ref|NP_001238509.1| (e_value=2e-72) uncharacterized protein LOC100306416 [Glycine max];; (*SWP) sp|P6842[illegible]
NbS00020349g0004.1 **Mass:** 14009 **Score:** 93 **Matches:** 8(7) **Sequences:** 4(4)
NbS00020349g0004.1 protein AED:0.03 eAED:0.03 QI:0|-1|0|1|-1|1|1|0|125; (*SWP) sp|P68428|H32_WHEAT (e_value=1e-72) Histone H3.2 OS=Triticum aestivum PE=1 SV=2;; (*TAIR) AT5G65360.1 (e_value=1e-73)
NbS00026286g0015.1 **Mass:** 20246 **Score:** 93 **Matches:** 8(7) **Sequences:** 4(4)
NbS00026286g0015.1 protein AED:0.06 eAED:0.06 QI:0|0|0|1|0|0|2|0|178; (*GB) gi|156350379|ref|XP_001622258.1| (e_value=8e-77) predicted protein [Nematostella vectensis];; (*SWP) sp|P68428|H32_WHEAT

---

32. NbS00035687g0007.1 **Mass:** 28986 **Score:** 92 **Matches:** 4(4) **Sequences:** 3(3) **emPAI:** 0.39
NbS00035687g0007.1 protein AED:0.23 eAED:0.23 QI:300|1|0.75|1|1|1|4|0|274; (*GB) gi|84620802|gb|ABC59515.1| (e_value=9e-120) chloroplast photosystem II 22 kDa component [Nicotiana benthamiana];; (

Query	Observed	Mr(expt)	Mr(calc)	ppm	Miss	Score	Expect	Rank	Unique	Peptide
10697	453.2457	904.4768	904.4767	0.14	0	41	0.0087	1	U	K.ANELFVGR.L
16425	790.9203	1579.8261	1579.8246	0.97	0	61	4.9e-005	1	U	K.SALGLSEGGPLFGFTK.A 9861
17856	874.9861	1747.9577	1747.9542	2.01	0	31	0.032	1		R.VAMIGFAASLLGEAITGK.G

---

33. NbS00001559g0021.1 **Mass:** 90961 **Score:** 92 **Matches:** 5(4) **Sequences:** 5(4) **emPAI:** 0.19
NbS00001559g0021.1 protein AED:0.25 eAED:0.31 QI:138|0.5|0.53|1|0.85|0.93|15|300|837; (*GB) gi|356513635|ref|XP_003525517.1| (e_value=0.0) PREDICTED: DEAD-box ATP-dependent RNA helicase 30-like [G

Query	Observed	Mr(expt)	Mr(calc)	ppm	Miss	Score	Expect	Rank	Unique	Peptide
12708	572.2986	1142.5827	1142.5819	0.73	0	51	0.00058	1		R.DDLDNLTLPK.A
12839	580.8116	1159.6087	1159.6085	0.24	0	31	0.072	1		R.DLIGIAETGSGK.T
13569	622.8365	1243.6585	1243.6594	-0.72	0	38	0.014	1		R.SPIMIATDVAAR.G
14505	670.3318	1338.6491	1338.6489	0.16	0	35	0.02	1	U	R.IMTEEDVALYR.A
15315	719.8544	1437.6942	1437.6922	1.38	0	37	0.014	1	U	R.ANISSTCIYGGAPK.G

---

34. NbS00009714g0011.1 **Mass:** 45483 **Score:** 91 **Matches:** 4(4) **Sequences:** 3(3) **emPAI:** 0.23
NbS00009714g0011.1 protein AED:0.20 eAED:0.20 QI:131|0.83|0.71|1|0.83|0.57|7|463|410; (*SWP) sp|Q40565|RCA2_TOBAC (e_value=0.0) Ribulose bisphosphate carboxylase/oxygenase activase 2, chloroplastic

Query	Observed	Mr(expt)	Mr(calc)	ppm	Miss	Score	Expect	Rank	Unique	Peptide
12672	569.8536	1137.6927	1137.6910	1.49	0	34	0.0056	1	U	K.VPLILGVWGGK.G
17765	868.9094	1735.8042	1735.8013	1.64	0	52	0.00032	1		K.GLVQDFSDDQQDITR.G 17763
18563	941.9894	1881.9642	1881.9625	0.89	0	36	0.015	1		K.IVDTFPGQSIDFFGALR.A

Proteins matching the same set of peptides:
NbS00047700g0013.1 **Mass:** 81330 **Score:** 91 **Matches:** 4(4) **Sequences:** 3(3)
NbS00047700g0013.1 protein AED:0.19 eAED:0.19 QI:131|0.6|0.63|1|0.8|0.54|11|307|726; (*GB) gi|12643758|sp|Q40565.1|RCA2_TOBAC (e_value=0.0) RecName: Full=Ribulose bisphosphate carboxylase/oxygenase

---

35. NbS00025223g0014.1 **Mass:** 15188 **Score:** 89 **Matches:** 3(2) **Sequences:** 2(1) **emPAI:** 0.50
NbS00025223g0014.1 protein AED:0.37 eAED:0.37 QI:0|1|0.33|1|1|0.66|3|0|136; (*GB) gi|51490663|emb|CAG26902.1| (e_value=3e-63) ALY protein [Nicotiana benthamiana];; (*SWP) sp|Q86V81|THOC4_HUMAN (e_

Query	Observed	Mr(expt)	Mr(calc)	ppm	Miss	Score	Expect	Rank	Unique	Peptide
11383	490.2639	978.5132	978.5134	-0.29	0	31	0.066	1	U	K.GTAEVIFSR.R
17748	866.9918	1731.9690	1731.9672	1.04	0	59	4.3e-005	1	U	K.IEFAGPNIGAPALPPLR.N 17747

---

36. NbS00010583g0001.1 **Mass:** 22083 **Score:** 88 **Matches:** 3(3) **Sequences:** 2(2) **emPAI:** 0.53

NbS00010583g0001.1 protein AED:0.25 eAED:0.25 QI:0|-1|0|1|-1|1|1|0|210; (*GB) gi|31711507|dbj|BAC77634.1| (e_value=2e-132) 24K germin like protein [Nicotiana tabacum];; (*SWP) sp|O04011|ABP20_PRUPI

Query	Observed	Mr(expt)	Mr(calc)	ppm	Miss	Score	Expect	Rank	Unique	Peptide
9277	349.2336	696.4525	696.4534	-1.20	0	33	0.0052	1	U	K.LNPLIK.A
20042	762.7409	2285.2009	2285.1991	0.80	0	(50)	0.00038	1	U	K.AAVTPAFAPQFPGLNGLGISMAR.L
20101	768.0721	2301.1946	2301.1940	0.28	0	52	0.0003	1	U	K.AAVTPAFAPQFPGLNGLGISMAR.L

Proteins matching the same set of peptides:

NbC25250105g0001.1 **Mass:** 20517 **Score:** 88 **Matches:** 3(3) **Sequences:** 2(2)

NbC25250105g0001.1 protein AED:0.01 eAED:0.01 QI:0|-1|1|1|-1|1|1|0|194; (*GB) gi|31711507|dbj|BAC77634.1| (e_value=1e-120) 24K germin like protein [Nicotiana tabacum];; (*SWP) sp|O04011|ABP20_PRUPI

---

37. gi|119581148|gb|EAW60744.1| **Mass:** 57754 **Score:** 85 **Matches:** 6(5) **Sequences:** 5(5) **emPAI:** 0.32

gi|119581148|gb|EAW60744.1| keratin 9 (epidermolytic palmoplantar keratoderma) [Homo sapiens]

Query	Observed	Mr(expt)	Mr(calc)	ppm	Miss	Score	Expect	Rank	Unique	Peptide
12028	530.7853	1059.5560	1059.5560	-0.02	0	37	0.018	1	U	K.TLLDIDNTR.M 12031
12799	579.2992	1156.5838	1156.5836	0.16	0	34	0.028	1	U	R.QGVDADINGLR.Q
13102	595.8088	1189.6031	1189.6013	1.57	0	35	0.026	1	U	R.QVLDNLTMEK.S
20158	772.3765	2314.1076	2314.1072	0.16	1	40	0.0046	1	U	K.SDLEMQYETLQEELMALKK.N
21049	1088.8448	3263.5127	3263.5066	1.89	0	40	0.0023	1	U	K.DIENQYETQITQIEHEVSSSGQEVQSSAK.E

---

38. NbS00001849g0017.1 **Mass:** 8189 **Score:** 84 **Matches:** 2(2) **Sequences:** 1(1) **emPAI:** 0.43

NbS00001849g0017.1 protein AED:0.76 eAED:1.00 QI:0|0|0|0.66|0.5|0.66|3|0|72; (*GB) gi|94466657|emb|CAJ44457.1| (e_value=3e-26) ALY protein [Nicotiana benthamiana];; (*ITAG) Solyc10g086400.1.1 (e_va

Query	Observed	Mr(expt)	Mr(calc)	ppm	Miss	Score	Expect	Rank	Unique	Peptide
17748	866.9918	1731.9690	1731.9672	1.04	0	59	4.3e-005	1	U	K.IEFAGPNIGAPALPPIR.N 17747

Proteins matching the same set of peptides:

NbS00004226g0001.1 **Mass:** 16820 **Score:** 84 **Matches:** 2(2) **Sequences:** 1(1)

NbS00004226g0001.1 protein ; (*GB) gi|94466657|emb|CAJ44457.1| (e_value=2e-33) ALY protein [Nicotiana benthamiana];; (*TAIR) AT5G02530.2 (e_value=1e-14) | Symbols: | RNA-binding (RRM/RBD/RNP motifs

---

39. NbS00029393g0012.1 **Mass:** 49230 **Score:** 82 **Matches:** 3(3) **Sequences:** 2(2) **emPAI:** 0.14

NbS00029393g0012.1 protein AED:0.08 eAED:0.08 QI:319|0.42|0.37|1|1|1|8|307|444; (*GB) gi|12643757|sp|Q40460.1|RCA1_TOBAC (e_value=0.0) RecName: Full=Ribulose bisphosphate carboxylase/oxygenase acti

Query	Observed	Mr(expt)	Mr(calc)	ppm	Miss	Score	Expect	Rank	Unique	Peptide
17522	845.4301	1688.8457	1688.8410	2.78	0	38	0.01	1		K.LLNSFDGPPTFEQPK.M
17765	868.9094	1735.8042	1735.8013	1.64	0	52	0.00032	1		K.GLVQDFSDDQQDITR.G 17763

Proteins matching the same set of peptides:

NbS00044990g0002.1 **Mass:** 49599 **Score:** 82 **Matches:** 3(3) **Sequences:** 2(2)

NbS00044990g0002.1 protein AED:0.04 eAED:0.04 QI:333|0.66|0.57|1|1|1|7|326|449; (*SWP) sp|Q40460|RCA1_TOBAC (e_value=0.0) Ribulose bisphosphate carboxylase/oxygenase activase 1, chloroplastic OS=N

---

40. NbS00042109g0018.1 **Mass:** 27332 **Score:** 82 **Matches:** 3(3) **Sequences:** 3(3) **emPAI:** 0.41

NbS00042109g0018.1 protein AED:0.00 eAED:0.00 QI:0|1|0.5|1|1|1|2|0|256; (*SWP) sp|P27524|CB4A_SOLLC (e_value=3e-163) Chlorophyll a-b binding protein CP24 10A, chloroplastic OS=Solanum lycopersicum

Query	Observed	Mr(expt)	Mr(calc)	ppm	Miss	Score	Expect	Rank	Unique	Peptide
18582	944.4311	1886.8476	1886.8435	2.19	0	43	0.0019	1	U	K.TAENFANFTGEQGYPGGK.F
20816	942.1537	2823.4392	2823.4331	2.18	1	52	0.00023	1	U	K.FFDPLSLAGTLQDGVYIPDTEKLER.L
21121	1172.9186	3515.7339	3515.7249	2.55	1	32	0.017	1	U	K.GGGNLVDPEWLDGSLPGDYGFDPLGLGKDPAFLK.W

---

41. NbS00027807g0002.1 **Mass:** 45317 **Score:** 82 **Matches:** 3(3) **Sequences:** 3(3) **emPAI:** 0.24

NbS00027807g0002.1 protein AED:0.05 eAED:0.05 QI:0|1|0.5|1|0|0|2|953|413; (*GB) gi|68566313|sp|Q40450.2|EFTUA_NICSY (e_value=0.0) RecName: Full=Elongation factor TuA, chloroplastic; Short=EF-TuA; I

Query	Observed	Mr(expt)	Mr(calc)	ppm	Miss	Score	Expect	Rank	Unique	Peptide
17575	850.4563	1698.8980	1698.8974	0.36	0	47	0.0011	1	U	K.ILDEAMAGDNVGLLLR.G

18185	905.9480	1809.8814	1809.8745	3.86	0	30	0.052	1	U	R.GITINTATVEYETENR.H
19713	1084.0637	2166.1129	2166.1031	4.53	0	50	0.00039	1	U	R.QTELPFLMAIEDVFSITGR.G

42. NbS00002894g0003.1 **Mass:** 42068 **Score:** 81 **Matches:** 3(3) **Sequences:** 3(3) **emPAI:** 0.25

NbS00002894g0003.1 protein AED:0.02 eAED:0.02 QI:0|1|0.5|1|1|1|2|0|386; (*GB) gi|78191448|gb|ABB29945.1| (e_value=0.0) ADP/ATP translocator-like [Solanum tuberosum];; (*SWP) sp|P25083|ADT1_SOLTU (e

Query	Observed	Mr(expt)	Mr(calc)	ppm	Miss	Score	Expect	Rank	Unique	Peptide
8561	596.3200	1190.6254	1190.6295	-3.43	0	55	0.00047	1	U	R.AVAGAGVLAGYDK.L
13431	614.8140	1227.6135	1227.6136	-0.05	0	33	0.029	1	U	K.SSFDAFSQIVK.N
18691	956.9974	1911.9803	1911.9764	2.03	0	51	0.00044	1	U	K.GLAAFATDFLMGGVSAAVSK.T

Proteins matching the same set of peptides:

NbS00017949g0009.1 **Mass:** 42022 **Score:** 81 **Matches:** 3(3) **Sequences:** 3(3)

NbS00017949g0009.1 protein AED:0.29 eAED:0.29 QI:81|1|1|1|0.66|0.75|4|419|386; (*GB) gi|78191448|gb|ABB29945.1| (e_value=0.0) ADP/ATP translocator-like [Solanum tuberosum];; (*SWP) sp|P25083|ADT1_S

NbS00020014g0003.1 **Mass:** 44842 **Score:** 81 **Matches:** 3(3) **Sequences:** 3(3)

NbS00020014g0003.1 protein AED:0.26 eAED:0.29 QI:0|0.66|0.75|1|1|1|4|459|410; (*GB) gi|78191448|gb|ABB29945.1| (e_value=0.0) ADP/ATP translocator-like [Solanum tuberosum];; (*SWP) sp|P25083|ADT1_SC

43. NbS00007511g0005.1 **Mass:** 117195 **Score:** 79 **Matches:** 4(4) **Sequences:** 4(4) **emPAI:** 0.12

NbS00007511g0005.1 protein AED:0.26 eAED:0.26 QI:0|0.62|0.55|0.88|1|1|9|0|1084; (*GB) gi|359477631|ref|XP_002274485.2| (e_value=0.0) PREDICTED: uncharacterized protein LOC100252874 [Vitis vinifera]

Query	Observed	Mr(expt)	Mr(calc)	ppm	Miss	Score	Expect	Rank	Unique	Peptide
11985	528.2960	1054.5775	1054.5771	0.35	0	31	0.037	1	U	R.GADIVVATPGR.L
13411	613.8590	1225.7035	1225.7030	0.35	0	58	4.9e-005	1	U	K.TPILVATDVAAR.G
14489	668.8245	1335.6344	1335.6315	2.14	0	36	0.02	1		R.MLDMGFEPQIR.K
19362	679.6753	2036.0041	2036.0004	1.82	0	30	0.046	1		R.VVINYDFPTGIEDYVHR.I

Proteins matching the same set of peptides:

NbS00029160g0008.1 **Mass:** 117387 **Score:** 79 **Matches:** 4(4) **Sequences:** 4(4)

NbS00029160g0008.1 protein AED:0.20 eAED:0.21 QI:76|0.66|0.6|0.9|0.55|0.5|10|0|1085; (*GB) gi|359477631|ref|XP_002274485.2| (e_value=0.0) PREDICTED: uncharacterized protein LOC100252874 [Vitis vin:

44. NbS00056669g0006.1 **Mass:** 26768 **Score:** 78 **Matches:** 3(3) **Sequences:** 3(3) **emPAI:** 0.42

NbS00056669g0006.1 protein AED:0.08 eAED:0.08 QI:167|1|1|1|1|1|5|1743|252; (*SWP) sp|B5FXN8|THOC4_TAEGU (e_value=4e-36) THO complex subunit 4 OS=Taeniopygia guttata GN=ALYREF PE=2 SV=1;; (*TAIR) A

Query	Observed	Mr(expt)	Mr(calc)	ppm	Miss	Score	Expect	Rank	Unique	Peptide
11263	483.2567	964.4988	964.4978	1.05	0	39	0.012	1	U	K.GTAEVVFSR.R
18342	921.9612	1841.9078	1841.9047	1.70	0	54	0.00021	1	U	K.LYISNLDYGVSNEDIK.E
21158	1241.8961	3722.6665	3722.6696	-0.82	0	34	0.0063	1	U	K.APEAAWNHDMFAAADQAFPFGQAGGDQAASSISTGTK.L

45. NbS00028679g0001.1 **Mass:** 34422 **Score:** 77 **Matches:** 1(1) **Sequences:** 1(1) **emPAI:** 0.10

NbS00028679g0001.1 protein AED:0.08 eAED:0.08 QI:281|0.88|0.9|1|0.88|0.8|10|299|305; (*GB) gi|255542956|ref|XP_002512541.1| (e_value=0.0) NAD dependent epimerase/dehydratase, putative [Ricinus comr

Query	Observed	Mr(expt)	Mr(calc)	ppm	Miss	Score	Expect	Rank	Unique	Peptide
15892	759.9126	1517.8106	1517.8089	1.12	0	77	1.1e-006	1	U	K.DLATAFIQVLGNEK.A

46. NbS00036785g0006.1 **Mass:** 38193 **Score:** 77 **Matches:** 1(1) **Sequences:** 1(1) **emPAI:** 0.09

NbS00036785g0006.1 protein AED:0.27 eAED:0.32 QI:267|0.88|0.8|1|0.77|0.6|10|263|336; (*GB) gi|356572914|ref|XP_003554610.1| (e_value=0.0) PREDICTED: uncharacterized protein At1g09340, chloroplastic

Query	Observed	Mr(expt)	Mr(calc)	ppm	Miss	Score	Expect	Rank	Unique	Peptide
15892	759.9126	1517.8106	1517.8453	-22.85	1	77	1.1e-006	1	U	K.DLATAFIKVLGNEK.A

47. NbS00030061g0007.1 **Mass:** 40220 **Score:** 77 **Matches:** 3(2) **Sequences:** 3(2) **emPAI:** 0.27

NbS00030061g0007.1 protein AED:0.13 eAED:0.13 QI:243|1|1|1|0.85|0.75|8|1441|373; (*GB) gi|77416977|gb|ABA81884.1| (e_value=7e-108) nuclear RNA binding protein-like [Solanum tuberosum];; (*TAIR) AT

Query	Observed	Mr(expt)	Mr(calc)	ppm	Miss	Score	Expect	Rank	Unique	Peptide
12804	579.3215	1156.6284	1156.6274	0.87	0	31	0.06	1	U	K.ALMALKPEER.K
11528	997.5000	1992.9854	1992.9792	3.11	0	36	0.036	1	U	K.NYSAPSIEDAGQFPSLVAK.-
20357	1208.5505	2415.0865	2415.0826	1.61	0	54	0.00013	1	U	R.GNWGTPADDIAQETEVAVNDGEK.I

48. NbS00010663g0016.1 **Mass:** 67391 **Score:** 74 **Matches:** 4(3) **Sequences:** 4(3) **emPAI:** 0.21

NbS00010663g0016.1 protein AED:0.20 eAED:0.20 QI:7|1|0.88|1|1|1|9|298|622; (*GB) gi|224135925|ref|XP_002322195.1| (e_value=0.0) predicted protein [Populus trichocarpa];; (*SWP) sp|Q8W4R3|RH30_ARATH

Query	Observed	Mr(expt)	Mr(calc)	ppm	Miss	Score	Expect	Rank	Unique	Peptide
12708	572.2986	1142.5827	1142.5819	0.73	0	51	0.00058	1		R.DDLDNLTLPK.A
12839	580.8116	1159.6087	1159.6085	0.24	0	31	0.072	1		R.DLIGIAETGSGK.T
13569	622.8365	1243.6585	1243.6594	-0.72	0	38	0.014	1		R.SPIMIATDVAAR.G
14489	668.8245	1335.6344	1335.6315	2.14	0	36	0.02	1		R.MLDMGFEPQIR.K

---

49. NbS00024580g0005.1 **Mass:** 35587 **Score:** 74 **Matches:** 2(2) **Sequences:** 2(2) **emPAI:** 0.19

NbS00024580g0005.1 protein AED:0.21 eAED:0.21 QI:87|0.83|0.85|1|0.83|0.85|7|278|321; (*SWP) sp|Q641W4|RFC2_RAT (e_value=5e-157) Replication factor C subunit 2 OS=Rattus norvegicus GN=Rfc2 PE=2 SV=1

Query	Observed	Mr(expt)	Mr(calc)	ppm	Miss	Score	Expect	Rank	Unique	Peptide
11790	**515.7798**	**1029.5450**	**1029.5455**	**-0.43**	**0**	**53**	**0.00041**	**1**	**U**	**R.LSDQEILGR.L**
19431	**1030.0452**	**2058.0758**	**2058.0674**	**4.10**	**0**	**46**	**0.0013**	**1**	**U**	**K.ALYDLGYSPTDIITTLFR.I**

---

50. NbS00003075g0011.1 **Mass:** 15245 **Score:** 72 **Matches:** 3(3) **Sequences:** 2(2) **emPAI:** 0.50

NbS00003075g0011.1 protein AED:0.28 eAED:0.28 QI:0|1|0.66|1|1|1|3|0|143; (*GB) gi|2499967|sp|Q41229.1|PSAEB_NICSY (e_value=1e-59) RecName: Full=Photosystem I reaction center subunit IV B, chloropla

Query	Observed	Mr(expt)	Mr(calc)	ppm	Miss	Score	Expect	Rank	Unique	Peptide
15860	**757.3849**	**1512.7552**	**1512.7533**	**1.29**	**0**	**65**	**2e-005**	**1**	**U**	**K.GVGSVVAVDQDPNTR.Y**
19902	**743.0674**	**2226.1803**	**2226.1757**	**2.07**	**1**	**33**	**0.019**	**1**	**U**	**K.GVGSVVAVDQDPNTRYPVVVR.F** 19903

Proteins matching the same set of peptides:

NbS00019085g0006.1 **Mass:** 15263 **Score:** 72 **Matches:** 3(3) **Sequences:** 2(2)

NbS00019085g0006.1 protein AED:0.21 eAED:0.21 QI:205|1|1|1|1|1|3|512|143; (*GB) gi|2499967|sp|Q41229.1|PSAEB_NICSY (e_value=3e-57) RecName: Full=Photosystem I reaction center subunit IV B, chloropl

---

51. NbS00019623g0001.1 **Mass:** 49575 **Score:** 70 **Matches:** 3(2) **Sequences:** 2(1) **emPAI:** 0.07

NbS00019623g0001.1 protein AED:0.21 eAED:0.21 QI:0|1|0.5|1|1|1|2|0|447; (*GB) gi|3869088|dbj|BAA34348.1| (e_value=0.0) elongation factor-1 alpha [Nicotiana paniculata];; (*SWP) sp|P17786|EF1A_SOLLC

Query	Observed	Mr(expt)	Mr(calc)	ppm	Miss	Score	Expect	Rank	Unique	Peptide
11498	**497.2716**	**992.5286**	**992.5291**	**-0.47**	**1**	**30**	**0.08**	**2**	**U**	**K.ARYDEIVK.E**
7805	**513.3100**	**1024.6054**	**1024.6030**	**2.44**	**0**	**58**	**7.4e-005**	**1**	**U**	**K.IGGIGTVPVGR.V** 11756

Proteins matching the same set of peptides:

NbS00023178g0001.1 **Mass:** 45458 **Score:** 70 **Matches:** 3(2) **Sequences:** 2(1)

NbS00023178g0001.1 protein AED:0.03 eAED:0.03 QI:0|0|0|1|1|1|3|0|413; (*SWP) sp|Q40034|EF1A2_HORVU (e_value=0.0) Elongation factor 1-alpha OS=Hordeum vulgare GN=BLT63 PE=1 SV=1;; (*TAIR) AT5G60390.

NbS00024768g0013.1 **Mass:** 49347 **Score:** 70 **Matches:** 3(2) **Sequences:** 2(1)

NbS00024768g0013.1 protein AED:0.17 eAED:0.19 QI:3|0.33|0|1|0.66|0.75|4|0|443; (*GB) gi|3869088|dbj|BAA34348.1| (e_value=0.0) elongation factor-1 alpha [Nicotiana paniculata];; (*SWP) sp|P17786|EF1

NbS00026055g0002.1 **Mass:** 40879 **Score:** 70 **Matches:** 3(2) **Sequences:** 2(1)

NbS00026055g0002.1 protein AED:0.04 eAED:0.06 QI:0|-1|0|1|-1|1|1|0|373; (*GB) gi|1864017|dbj|BAA09709.1| (e_value=0.0) elongation factor-1 alpha [Nicotiana tabacum];; (*SWP) sp|P43643|EF1A_TOBAC (e

NbS00037026g0004.1 **Mass:** 49587 **Score:** 70 **Matches:** 3(2) **Sequences:** 2(1)

NbS00037026g0004.1 protein AED:0.17 eAED:0.17 QI:0|1|0.5|1|1|1|2|0|447; (*GB) gi|3869088|dbj|BAA34348.1| (e_value=0.0) elongation factor-1 alpha [Nicotiana paniculata];; (*SWP) sp|P43643|EF1A_TOBAC

NbS00060738g0002.1 **Mass:** 50846 **Score:** 70 **Matches:** 3(2) **Sequences:** 2(1)

NbS00060738g0002.1 protein AED:0.07 eAED:0.09 QI:0|0.33|0.25|1|1|1|4|0|458; (*SWP) sp|P43643|EF1A_TOBAC (e_value=0.0) Elongation factor 1-alpha OS=Nicotiana tabacum PE=2 SV=1;; (*TAIR) AT5G60390.3

NbS00007372g0013.1 **Mass:** 54761 **Score:** 70 **Matches:** 3(2) **Sequences:** 2(1)

NbS00007372g0013.1 protein AED:0.21 eAED:0.21 QI:0|0|0|1|0.5|0.66|3|0|494; (*GB) gi|1864017|dbj|BAA09709.1| (e_value=0.0) elongation factor-1 alpha [Nicotiana tabacum];; (*SWP) sp|P43643|EF1A_TOBAC

NbS00024768g0017.1 **Mass:** 101076 **Score:** 70 **Matches:** 3(2) **Sequences:** 2(1)

NbS00024768g0017.1 protein AED:0.14 eAED:0.15 QI:0|0|0.33|1|0|0|6|0|911; (*GB) gi|297849086|ref|XP_002892424.1| (e_value=0.0) T6D22.2 [Arabidopsis lyrata subsp. lyrata];; (*SWP) sp|P17786|EF1A_SOLL

---

52. sp|TRYP_PIG| **Mass:** 25078 **Score:** 70 **Matches:** 2(2) **Sequences:** 1(1) **emPAI:** 0.13

sp|TRYP_PIG|

Query	Observed	Mr(expt)	Mr(calc)	ppm	Miss	Score	Expect	Rank	Unique	Peptide
10227	**421.7582**	**841.5019**	**841.5022**	**-0.34**	**0**	**55**	**0.00018**	**1**	**U**	**R.VATVSLPR.S** 10223

Proteins matching the same set of peptides:

gi|3318722|pdb|1AN1|E **Mass:** 24142 **Score:** 70 **Matches:** 2(2) **Sequences:** 1(1)

gi|3318722|pdb|1AN1|E Chain E, Leech-Derived Tryptase InhibitorTRYPSIN COMPLEX

---

53. NbS00006644g0116.1 **Mass:** 121848 **Score:** 70 **Matches:** 4(3) **Sequences:** 4(3) **emPAI:** 0.11
NbS00006644g0116.1 protein AED:0.00 eAED:0.00 QI:364|1|1|1|1|1|3|541|1093

Query	Observed	Mr(expt)	Mr(calc)	ppm	Miss	Score	Expect	Rank	Unique	Peptide
14630	676.3349	1350.6552	1350.6568	-1.15	0	53	0.0004	1	U	R.WALIDFSSQER.N
15468	731.3741	1460.7336	1460.7300	2.49	0	30	0.068	1	U	K.LFADNPGQFPIDK.T
17611	568.9577	1703.8513	1703.8519	-0.35	1	30	0.056	1	U	R.DKLFADNPGQFPIDK.T
19177	660.6630	1978.9671	1978.9649	1.08	1	32	0.029	1	U	R.WALIDFSSQERNPNFR.L

54. NbS00003134g0011.1 **Mass:** 79899 **Score:** 68 **Matches:** 4(4) **Sequences:** 4(4) **emPAI:** 0.17
NbS00003134g0011.1 protein AED:0.32 eAED:0.33 QI:0|0.75|0.33|0.88|0.75|0.66|9|0|720; (*SWP) sp|P46942|DB10_NICSY (e_value=0.0) ATP-dependent RNA helicase-like protein DB10 OS=Nicotiana sylvestris I

Query	Observed	Mr(expt)	Mr(calc)	ppm	Miss	Score	Expect	Rank	Unique	Peptide
13177	599.8425	1197.6705	1197.6717	-1.03	0	32	0.03	1	U	R.SPVLVATDVAAR.G
14157	650.8577	1299.7009	1299.7034	-1.92	0	46	0.0017	1	U	R.ELATQIQAEAVK.F
14489	668.8245	1335.6344	1335.6315	2.14	0	36	0.02	1		R.MLDMGFEPQIR.K
19362	679.6753	2036.0041	2036.0004	1.82	0	30	0.046	1		K.VVINYDFPTGIEDYVHR.I

55. NbS00003763g0016.1 **Mass:** 99171 **Score:** 66 **Matches:** 3(3) **Sequences:** 3(3) **emPAI:** 0.10
NbS00003763g0016.1 protein AED:0.10 eAED:0.12 QI:0|0.92|0.8|0.93|1|1|15|0|884; (*GB) gi|225441896|ref|XP_002284404.1| (e_value=0.0) PREDICTED: uncharacterized protein At2g41620 [Vitis vinifera];;

Query	Observed	Mr(expt)	Mr(calc)	ppm	Miss	Score	Expect	Rank	Unique	Peptide
12578	563.3178	1124.6211	1124.6190	1.86	0	31	0.034	1	U	R.DGGIDILLGPR.G
12681	570.7803	1139.5461	1139.5499	-3.33	0	42	0.0039	1	U	R.TPDFATDVFK.N
13992	641.8796	1281.7447	1281.7405	3.33	0	42	0.0016	1	U	R.ENVLAQQIVLR.Q

Proteins matching the same set of peptides:
NbS00021908g0010.1 **Mass:** 102988 **Score:** 66 **Matches:** 3(3) **Sequences:** 3(3)
NbS00021908g0010.1 protein AED:0.11 eAED:0.11 QI:0|0.71|0.73|1|0.85|0.8|15|307|915; (*SWP) sp|O22224|Y2162_ARATH (e_value=0.0) Uncharacterized protein At2g41620 OS=Arabidopsis thaliana GN=At2g4162(

56. NbS00004956g0015.1 **Mass:** 9825 **Score:** 65 **Matches:** 4(3) **Sequences:** 2(2) **emPAI:** 1.47
NbS00004956g0015.1 protein AED:0.25 eAED:0.30 QI:0|0|0|1|0|0|2|0|84; (*GB) gi|42718201|gb|AAS38532.1| (e_value=7e-41) ribulose-1,5-bisphosphate carboxylase/oxygenase large subunit [Hygrolembidium a

Query	Observed	Mr(expt)	Mr(calc)	ppm	Miss	Score	Expect	Rank	Unique	Peptide
10736	455.7260	909.4375	909.4378	-0.38	0	35	0.023	1	U	R.AVYECLR.G
15484	489.2559	1464.7458	1464.7474	-1.05	0	(31)	0.048	1	U	K.TFQGPPHGIQVER.D
15489	733.3815	1464.7484	1464.7474	0.69	0	46	0.0017	1	U	K.TFQGPPHGIQVER.D 15487

57. NbS00001373g0014.1 **Mass:** 97598 **Score:** 65 **Matches:** 2(2) **Sequences:** 2(2) **emPAI:** 0.07
NbS00001373g0014.1 protein AED:0.25 eAED:0.25 QI:0|0.87|0.64|1|0.75|0.82|17|0|866; (*GB) gi|225431788|ref|XP_002271622.1| (e_value=0.0) PREDICTED: acyl-CoA dehydrogenase family member 10 isoform 1

Query	Observed	Mr(expt)	Mr(calc)	ppm	Miss	Score	Expect	Rank	Unique	Peptide
14660	678.3804	1354.7462	1354.7456	0.43	0	41	0.004	1	U	R.LLVLEAADQLDR.L
18002	887.9487	1773.8829	1773.8838	-0.52	0	43	0.0034	1	U	K.EGLWNLWIPFDSAAR.A

58. NbS00058945g0001.1 **Mass:** 45005 **Score:** 65 **Matches:** 1(1) **Sequences:** 1(1) **emPAI:** 0.07
NbS00058945g0001.1 protein AED:0.25 eAED:0.26 QI:0|0.8|0.5|1|1|1|6|0|457; (*TAIR) AT5G58470.2 (e_value=1e-51) | Symbols: TAF15b | TBP-associated factor 15B | chr5:23638566-23640854 RVERSE LENGTH=4

Query	Observed	Mr(expt)	Mr(calc)	ppm	Miss	Score	Expect	Rank	Unique	Peptide
15888	759.8582	1517.7019	1517.6998	1.39	0	65	1.7e-005	1	U	R.NAGGYDISPAEAPEK.V

59. NbS00016433g0017.1 **Mass:** 41368 **Score:** 64 **Matches:** 2(2) **Sequences:** 2(2) **emPAI:** 0.17
NbS00016433g0017.1 protein AED:0.23 eAED:0.24 QI:195|1|0.9|1|1|1|10|0|371; (*GB) gi|255543841|ref|XP_002512983.1| (e_value=0.0) clathrin binding protein, putative [Ricinus communis];; (*TAIR) AT4G

Query	Observed	Mr(expt)	Mr(calc)	ppm	Miss	Score	Expect	Rank	Unique	Peptide
8207	555.3000	1108.5854	1108.5764	8.12	0	45	0.0045	1	U	R.GVEGYIVTGSK.Q
16302	780.8764	1559.7383	1559.7362	1.34	0	50	0.00049	1	U	K.EASAAMAAVEAQQQR.L

60. NbS00027647g0011.1 **Mass:** 90299 **Score:** 63 **Matches:** 2(2) **Sequences:** 2(2) **emPAI:** 0.07
NbS00027647g0011.1 protein AED:0.16 eAED:0.17 QI:0|0.9|0.72|1|0.9|0.72|11|0|816; (*GB) gi|297745425|emb|CBI40505.3| (e_value=0.0) unnamed protein product [Vitis vinifera];; (*SWP) sp|Q9FFQ1|RH31_AI

Query	Observed	Mr(expt)	Mr(calc)	ppm	Miss	Score	Expect	Rank	Unique	Peptide
12998	589.3144	1176.6142	1176.6139	0.32	0	38	0.014	1	U	R.LVELANEFSR.T
15165	709.8986	1417.7826	1417.7777	3.46	0	52	0.00031	1	U	K.STGLILVTSDVSAR.G

Proteins matching the same set of peptides:
NbS00051197g0005.1 **Mass:** 98617 **Score:** 63 **Matches:** 2(2) **Sequences:** 2(2)
NbS00051197g0005.1 protein AED:0.14 eAED:0.15 QI:0|0.88|0.8|1|1|1|10|0|891; (*SWP) sp|Q9FFQ1|RH31_ARATH (e_value=0.0) DEAD-box ATP-dependent RNA helicase 31 OS=Arabidopsis thaliana GN=RH31 PE=2 SV=

61. NbS00021832g0023.1 **Mass:** 9182 **Score:** 62 **Matches:** 2(1) **Sequences:** 1(1) **emPAI:** 0.38
NbS00021832g0023.1 protein AED:0.35 eAED:0.35 QI:0|0|0|1|0|0|2|0|80; (*GB) gi|305671961|gb|ADM63350.1| (e_value=2e-44) photosystem II cp47 protein [Aralidium pinnatifidum];; (*SWP) sp|A4QLM0|PSBB_I

Query	Observed	Mr(expt)	Mr(calc)	ppm	Miss	Score	Expect	Rank	Unique	Peptide
14064	645.8384	1289.6623	1289.6615	0.61	0	58	0.00013	1	U	R.AQLGEIFELDR.A 14066

62. NbS00007972g0020.1 **Mass:** 15164 **Score:** 62 **Matches:** 1(1) **Sequences:** 1(1) **emPAI:** 0.22
NbS00007972g0020.1 protein AED:0.25 eAED:0.25 QI:0|1|0.66|1|1|1|3|0|145; (*GB) gi|407353|dbj|BAA04633.1| (e_value=1e-68) PSI-H precursor [Nicotiana sylvestris];; (*SWP) sp|O04006|PSAH_BRARA (e_valu

Query	Observed	Mr(expt)	Mr(calc)	ppm	Miss	Score	Expect	Rank	Unique	Peptide
14215	653.3284	1304.6422	1304.6441	-1.49	0	62	4.9e-005	1	U	K.FFETFAAPFTK.R

Proteins matching the same set of peptides:
NbS00018155g0021.1 **Mass:** 12505 **Score:** 62 **Matches:** 1(1) **Sequences:** 1(1)
NbS00018155g0021.1 protein AED:0.15 eAED:0.20 QI:0|0.5|0.66|1|1|1|3|297|115; (*GB) gi|397555|emb|CAA43841.1| (e_value=1e-42) photosystem I psaH protein [Nicotiana sylvestris];; (*SWP) sp|O04006|PSA
NbS00025048g0006.1 **Mass:** 15287 **Score:** 62 **Matches:** 1(1) **Sequences:** 1(1)
NbS00025048g0006.1 protein AED:0.42 eAED:0.52 QI:0|0.5|0|1|1|1|3|0|145; (*GB) gi|407353|dbj|BAA04633.1| (e_value=4e-61) PSI-H precursor [Nicotiana sylvestris];; (*SWP) sp|P22179|PSAH_SPIOL (e_value
NbS00040113g0008.1 **Mass:** 15303 **Score:** 62 **Matches:** 1(1) **Sequences:** 1(1)
NbS00040113g0008.1 protein AED:0.33 eAED:0.34 QI:147|1|0.66|1|1|1|3|0|145; (*GB) gi|407355|dbj|BAA04634.1| (e_value=5e-71) PSI-H precursor [Nicotiana sylvestris];; (*SWP) sp|O04006|PSAH_BRARA (e_va
NbS00042453g0009.1 **Mass:** 13176 **Score:** 62 **Matches:** 1(1) **Sequences:** 1(1)
NbS00042453g0009.1 protein AED:0.43 eAED:0.43 QI:0|0.5|0|0.66|1|1|3|0|121; (*GB) gi|359806751|ref|NP_001241299.1| (e_value=3e-41) uncharacterized protein LOC100811201 [Glycine max];; (*SWP) sp|O04(
NbS00046982g0002.1 **Mass:** 11573 **Score:** 62 **Matches:** 1(1) **Sequences:** 1(1)
NbS00046982g0002.1 protein AED:0.37 eAED:0.37 QI:0|0.5|0|0.66|1|1|3|0|107; (*GB) gi|397555|emb|CAA43841.1| (e_value=3e-41) photosystem I psaH protein [Nicotiana sylvestris];; (*SWP) sp|O04006|PSAH_

63. NbS00036430g0009.1 **Mass:** 48651 **Score:** 61 **Matches:** 2(2) **Sequences:** 2(2) **emPAI:** 0.14
NbS00036430g0009.1 protein AED:0.35 eAED:0.35 QI:0|0|0|1|0|0|4|0|448; (*GB) gi|113170490|ref|YP_717281.1| (e_value=2e-170) Atp1 [Ostreococcus tauri];; (*SWP) sp|B3PQ70|ATPA_RHIE6 (e_value=2e-158) A

Query	Observed	Mr(expt)	Mr(calc)	ppm	Miss	Score	Expect	Rank	Unique	Peptide
11762	513.8000	1025.5855	1025.5869	-1.36	0	32	0.023	1		K.AVDSLVPIGR.A
13100	595.3248	1188.6351	1188.6350	0.11	0	55	0.00024	1	U	R.AAELTSLLESR.I

64. NbS00008911g0002.1 **Mass:** 47595 **Score:** 60 **Matches:** 3(3) **Sequences:** 3(3) **emPAI:** 0.22
NbS00008911g0002.1 protein AED:0.35 eAED:0.35 QI:460|0.77|0.7|1|1|1|10|229|443; (*GB) gi|327198779|emb|CBL43264.1| (e_value=0.0) glyceraldehyde-3-phosphate dehydrogenase [Solanum tuberosum];; (*SWI

Query	Observed	Mr(expt)	Mr(calc)	ppm	Miss	Score	Expect	Rank	Unique	Peptide
10179	417.2351	832.4557	832.4555	0.23	0	37	0.019	1		K.VAINGFGR.I
14892	692.8936	1383.7727	1383.7722	0.38	0	41	0.0037	1		R.AAALNIVPTSTGAAK.A
16848	807.4245	1612.8344	1612.8308	2.24	0	33	0.034	1	U	K.DSPLDVIVVNDSGGVK.N

Proteins matching the same set of peptides:
NbS00033331g0008.1 **Mass:** 45260 **Score:** 60 **Matches:** 3(3) **Sequences:** 3(3)
NbS00033331g0008.1 protein AED:0.30 eAED:0.30 QI:475|0.77|0.8|1|0.77|0.7|10|324|419; (*GB) gi|327198779|emb|CBL43264.1| (e_value=0.0) glyceraldehyde-3-phosphate dehydrogenase [Solanum tuberosum];;

65. NbS00001161g0103.1 **Mass:** 56283 **Score:** 59 **Matches:** 1(1) **Sequences:** 1(1) **emPAI:** 0.06
NbS00001161g0103.1 protein AED:0.03 eAED:0.04 QI:203|0.5|0.66|1|0|0|3|2345|542

Query	Observed	Mr(expt)	Mr(calc)	ppm	Miss	Score	Expect	Rank	Unique	Peptide
18842	965.4376	1928.8606	1928.8534	3.70	0	59	3.8e-005	1	U	R.DSQMGNSGYQGSIQTTQK.I

66. gi|297692195|ref|XP_002823453.1| **Mass:** 62271 **Score:** 59 **Matches:** 3(3) **Sequences:** 3(3) **emPAI:** 0.17
gi|297692195|ref|XP_002823453.1| PREDICTED: LOW QUALITY PROTEIN: ATP synthase subunit beta, mitochondrial-like [Pongo abelii]

Query	Observed	Mr(expt)	Mr(calc)	ppm	Miss	Score	Expect	Rank	Unique	Peptide
11352	488.2848	974.5551	974.5549	0.16	0	32	0.047	1		K.IGLFGGAGVGK.T
15295	718.3777	1434.7408	1434.7467	-4.09	0	36	0.02	1	U	R.FTQAGSEVSALLGR.I
16548	801.4095	1600.8045	1600.8031	0.87	0	38	0.0097	1	U	K.VALVYGQMNEPPGAR.A

---

67. NbS00027428g0011.1 **Mass:** 84144 **Score:** 58 **Matches:** 2(2) **Sequences:** 2(2) **emPAI:** 0.08

NbS00027428g0011.1 protein AED:0.20 eAED:0.20 QI:0|0|0|1|1|1|2|0|723; (*GB) gi|359475106|ref|XP_003631587.1| (e_value=0.0) PREDICTED: DEAD-box ATP-dependent RNA helicase 21-like [Vitis vinifera];;

Query	Observed	Mr(expt)	Mr(calc)	ppm	Miss	Score	Expect	Rank	Unique	Peptide
12787	578.3401	1154.6656	1154.6659	-0.27	0	46	0.00097	1	U	R.NPVVVTIGTAGK.T
13868	639.3461	1276.6776	1276.6776	0.03	0	41	0.0053	1	U	R.YNVLVATDVAGR.G

Proteins matching the same set of peptides:

NbS00035002g0004.1 **Mass:** 83277 **Score:** 58 **Matches:** 2(2) **Sequences:** 2(2)

NbS00035002g0004.1 protein AED:0.32 eAED:0.32 QI:0|-1|0|1|-1|1|1|0|715; (*GB) gi|359475106|ref|XP_003631587.1| (e_value=0.0) PREDICTED: DEAD-box ATP-dependent RNA helicase 21-like [Vitis vinifera];

---

68. NbS00019265g0001.1 **Mass:** 46832 **Score:** 57 **Matches:** 1(1) **Sequences:** 1(1) **emPAI:** 0.07

NbS00019265g0001.1 protein AED:0.29 eAED:0.34 QI:99|0.6|0.66|1|1|1|6|0|422; (*GB) gi|115765|sp|P10708.1|CB12_SOLLC (e_value=3e-151) RecName: Full=Chlorophyll a-b binding protein 7, chloroplastic; A

Query	Observed	Mr(expt)	Mr(calc)	ppm	Miss	Score	Expect	Rank	Unique	Peptide
18556	941.0032	1879.9918	1879.9906	0.63	0	57	9.1e-005	1	U	R.WAMLGAAGIFIPEFLTK.I

Proteins matching the same set of peptides:

NbS00036008g0006.1 **Mass:** 31337 **Score:** 57 **Matches:** 1(1) **Sequences:** 1(1)

NbS00036008g0006.1 protein AED:0.22 eAED:0.27 QI:311|0.75|0.6|1|0.75|0.6|5|0|291; (*GB) gi|115765|sp|P10708.1|CB12_SOLLC (e_value=4e-172) RecName: Full=Chlorophyll a-b binding protein 7, chloroplas

NbS00048043g0004.1 **Mass:** 32195 **Score:** 57 **Matches:** 1(1) **Sequences:** 1(1)

NbS00048043g0004.1 protein AED:0.22 eAED:0.28 QI:313|1|0.75|1|1|1|4|0|297; (*GB) gi|115765|sp|P10708.1|CB12_SOLLC (e_value=7e-158) RecName: Full=Chlorophyll a-b binding protein 7, chloroplastic; Al

NbS00053209g0004.1 **Mass:** 31908 **Score:** 57 **Matches:** 1(1) **Sequences:** 1(1)

NbS00053209g0004.1 protein AED:0.23 eAED:0.28 QI:319|1|0.5|1|1|1|4|0|295; (*GB) gi|115765|sp|P10708.1|CB12_SOLLC (e_value=2e-157) RecName: Full=Chlorophyll a-b binding protein 7, chloroplastic; Alt

---

69. NbS00020769g0006.1 **Mass:** 26377 **Score:** 55 **Matches:** 3(3) **Sequences:** 2(2) **emPAI:** 0.27

NbS00020769g0006.1 protein AED:0.25 eAED:0.25 QI:307|0.66|1|1|0.66|0.75|4|448|250; (*GB) gi|84620804|gb|ABC59516.1| (e_value=5e-102) chloroplast photosystem II 22 kDa component [Nicotiana benthamia

Query	Observed	Mr(expt)	Mr(calc)	ppm	Miss	Score	Expect	Rank	Unique	Peptide
15141	708.3581	1414.7016	1414.6980	2.55	0	42	0.0042	1	U	K.FIDDPTPPTGLDK.A 15140
17856	874.9861	1747.9577	1747.9542	2.01	0	31	0.032	1		R.VAMIGFAASLLGEAITGK.G

---

70. NbS00042812g0008.1 **Mass:** 20462 **Score:** 54 **Matches:** 3(2) **Sequences:** 3(2) **emPAI:** 0.58

NbS00042812g0008.1 protein AED:0.19 eAED:0.19 QI:0|1|1|1|1|1|4|386|180; (*GB) gi|132118|sp|P26573.1|RBS8_NICPL (e_value=3e-119) RecName: Full=Ribulose bisphosphate carboxylase small chain 8B, chlo

Query	Observed	Mr(expt)	Mr(calc)	ppm	Miss	Score	Expect	Rank	Unique	Peptide
10948	467.2609	932.5073	932.5080	-0.72	0	36	0.022	1		R.IIGFDNVR.Q
11920	524.7642	1047.5139	1047.5138	0.12	0	31	0.064	1	U	K.EYPQAWVR.I
17650	856.9198	1711.8250	1711.8240	0.64	0	39	0.0066	1	U	R.QVQCISFIASKPDGY.-

Proteins matching the same set of peptides:

NbC25292626g0004.1 **Mass:** 18446 **Score:** 54 **Matches:** 3(2) **Sequences:** 3(2)

NbC25292626g0004.1 protein AED:0.08 eAED:0.08 QI:0|1|0.5|1|1|1|4|0|159; (*GB) gi|132118|sp|P26573.1|RBS8_NICPL (e_value=2e-112) RecName: Full=Ribulose bisphosphate carboxylase small chain 8B, chlo

---

71. NbC24305910g0003.1 **Mass:** 11284 **Score:** 53 **Matches:** 2(2) **Sequences:** 2(2) **emPAI:** 0.70

NbC24305910g0003.1 protein AED:0.03 eAED:0.03 QI:0|1|0.5|1|1|1|2|0|101; (*GB) gi|12643758|sp|Q40565.1|RCA2_TOBAC (e_value=6e-65) RecName: Full=Ribulose bisphosphate carboxylase/oxygenase activase 2

Query	Observed	Mr(expt)	Mr(calc)	ppm	Miss	Score	Expect	Rank	Unique	Peptide
17522	845.4301	1688.8457	1688.8410	2.78	0	38	0.01	1		K.LLNSFDGPPTFEQPK.M
18563	941.9894	1881.9642	1881.9625	0.89	0	36	0.015	1		K.IVDTFPGQSIDFFGALR.A

---

72. NbS00005125g0015.1 **Mass:** 38864 **Score:** 53 **Matches:** 2(2) **Sequences:** 2(2) **emPAI:** 0.18

NbS00005125g0015.1 protein AED:0.27 eAED:0.27 QI:600|0.91|0.84|1|0.83|0.69|13|286|356; (*GB) gi|304368145|gb|ADM26718.1| (e_value=0.0) glycolate oxidase [Nicotiana benthamiana];; (*SWP) sp|P05414|G

Query	Observed	Mr(expt)	Mr(calc)	ppm	Miss	Score	Expect	Rank	Unique	Peptide
11997	528.8066	1055.5986	1055.5975	1.05	0	35	0.016	1	U	K.AIALTVDTPR.L
18396	927.5426	1853.0707	1853.0662	2.38	0	38	0.0029	1	U	K.DVQWLQTITSLPILVK.G

Proteins matching the same set of peptides:

NbS00024535g0016.1 **Mass:** 38256 **Score:** 53 **Matches:** 2(2) **Sequences:** 2(2)
NbS00024535g0016.1 protein AED:0.23 eAED:0.23 QI:299|1|1|1|0.81|0.75|12|251|349; (*GB) gi|304368145|gb|ADM26718.1| (e_value=0.0) glycolate oxidase [Nicotiana benthamiana];; (*SWP) sp|P05414|GOX_SPI

NbS00025736g0004.1 **Mass:** 38849 **Score:** 53 **Matches:** 2(2) **Sequences:** 2(2)
NbS00025736g0004.1 protein AED:0.28 eAED:0.28 QI:605|0.91|0.84|1|0.83|0.76|13|359|356; (*GB) gi|304368145|gb|ADM26718.1| (e_value=0.0) glycolate oxidase [Nicotiana benthamiana];; (*SWP) sp|P05414|(

---

73. NbS00006841g0003.1 **Mass:** 31001 **Score:** 53 **Matches:** 1(1) **Sequences:** 1(1) **emPAI:** 0.11
NbS00006841g0003.1 protein AED:0.24 eAED:0.24 QI:284|1|1|1|0.66|0.75|4|120|287; (*GB) gi|115291793|gb|ABI93215.1| (e_value=0.0) water channel protein [Nicotiana tabacum];; (*SWP) sp|Q08451|PIP1_SOI

Query	Observed	Mr(expt)	Mr(calc)	ppm	Miss	Score	Expect	Rank	Unique	Peptide
14716	680.8653	1359.7160	1359.7147	1.01	0	53	0.00038	1	U	R.LGGGANIVNPGYTK.G

---

74. NbS00016136g0003.1 **Mass:** 58764 **Score:** 53 **Matches:** 2(2) **Sequences:** 2(2) **emPAI:** 0.12
NbS00016136g0003.1 protein AED:0.24 eAED:0.24 QI:0|0|0|1|1|1|3|0|533; (*GB) gi|392465167|dbj|BAM24707.1| (e_value=0.0) Heat shock protein 70 [Nicotiana tabacum];; (*SWP) sp|P27322|HSP72_SOLLC (e_va

Query	Observed	Mr(expt)	Mr(calc)	ppm	Miss	Score	Expect	Rank	Unique	Peptide
13170	599.3523	1196.6900	1196.6877	1.94	0	48	0.00054	1	U	K.DAGVIAGLNVLR.I
14686	679.8116	1357.6087	1357.6084	0.23	0	32	0.027	1	U	K.NALENYAYNMR.N

---

75. NbS00003826g0005.1 **Mass:** 59756 **Score:** 52 **Matches:** 2(2) **Sequences:** 2(2) **emPAI:** 0.11
NbS00003826g0005.1 protein AED:0.03 eAED:0.03 QI:80|0.87|0.88|1|0.87|0.88|9|279|511; (*GB) gi|1345684|sp|P49317.1|CATA3_NICPL (e_value=0.0) RecName: Full=Catalase isozyme 3;; (*SWP) sp|P49317|CATA:

Query	Observed	Mr(expt)	Mr(calc)	ppm	Miss	Score	Expect	Rank	Unique	Peptide
9579	742.3700	1482.7254	1482.7143	7.51	0	36	0.037	1	U	R.SIWISYWSQADK.T
19197	992.9990	1983.9835	1983.9843	-0.41	0	38	0.0094	1		R.EGNFDLVGNNFPVFFIR.D

Proteins matching the same set of peptides:

NbS00010881g0013.1 **Mass:** 54962 **Score:** 52 **Matches:** 2(2) **Sequences:** 2(2)
NbS00010881g0013.1 protein AED:0.15 eAED:0.15 QI:82|0.87|0.77|1|0.87|0.88|9|280|472; (*SWP) sp|P49317|CATA3_NICPL (e_value=0.0) Catalase isozyme 3 OS=Nicotiana plumbaginifolia GN=CAT3 PE=2 SV=1;;

---

76. NbS00019305g0027.1 **Mass:** 59772 **Score:** 52 **Matches:** 2(2) **Sequences:** 2(2) **emPAI:** 0.11
NbS00019305g0027.1 protein AED:0.37 eAED:0.37 QI:0|1|0.88|1|1|1|9|0|556; (*GB) gi|3676296|gb|AAD03392.1| (e_value=0.0) mitochondrial ATPase beta subunit [Nicotiana sylvestris];; (*SWP) sp|P17614|A:

Query	Observed	Mr(expt)	Mr(calc)	ppm	Miss	Score	Expect	Rank	Unique	Peptide
11352	488.2848	974.5551	974.5549	0.16	0	32	0.047	1		K.IGLFGGAGVGK.T
19798	1093.5759	2185.1373	2185.1379	-0.27	0	44	0.0019	1	U	R.IPSAVGYQPTLATDLGGLQER.I

Proteins matching the same set of peptides:

NbS00020973g0010.1 **Mass:** 58453 **Score:** 52 **Matches:** 2(2) **Sequences:** 2(2)
NbS00020973g0010.1 protein AED:0.23 eAED:0.23 QI:0|0.87|0.77|1|0.87|1|9|0|545; (*GB) gi|3676296|gb|AAD03392.1| (e_value=0.0) mitochondrial ATPase beta subunit [Nicotiana sylvestris];; (*SWP) sp|P1'

---

77. NbS00009638g0019.1 **Mass:** 20584 **Score:** 52 **Matches:** 3(3) **Sequences:** 3(3) **emPAI:** 0.58
NbS00009638g0019.1 protein AED:0.11 eAED:0.11 QI:333|1|1|1|1|1|3|176|181; (*GB) gi|132118|sp|P26573.1|RBS8_NICPL (e_value=6e-118) RecName: Full=Ribulose bisphosphate carboxylase small chain 8B, ch:

Query	Observed	Mr(expt)	Mr(calc)	ppm	Miss	Score	Expect	Rank	Unique	Peptide
10948	467.2609	932.5073	932.5080	-0.72	0	36	0.022	1		R.IIGFDNVR.Q
19897	742.3539	2224.0398	2224.0392	0.29	0	34	0.017	1	U	K.LPMFGCTDATQVLAEVEEAK.K
20202	779.7207	2336.1403	2336.1392	0.46	1	30	0.046	1	U	K.LPMFGCTDATQVLAEVEEAKK.A

Proteins matching the same set of peptides:

NbS00022486g0001.1 **Mass:** 20485 **Score:** 52 **Matches:** 3(3) **Sequences:** 3(3)
NbS00022486g0001.1 protein AED:0.21 eAED:0.21 QI:0|1|0.66|1|1|1|3|0|181; (*GB) gi|132118|sp|P26573.1|RBS8_NICPL (e_value=4e-116) RecName: Full=Ribulose bisphosphate carboxylase small chain 8B, chl(

NbS00027201g0002.1 **Mass:** 20557 **Score:** 52 **Matches:** 3(3) **Sequences:** 3(3)
NbS00027201g0002.1 protein AED:0.33 eAED:0.33 QI:0|1|0.75|1|1|1|4|0|180; (*GB) gi|59800169|sp|P69249.1|RBS_TOBAC (e_value=9e-123) RecName: Full=Ribulose bisphosphate carboxylase small chain, chlor(

NbS00027369g0007.1 **Mass:** 20455 **Score:** 52 **Matches:** 3(3) **Sequences:** 3(3)

NbS00027369g0007.1 protein AED:0.16 eAED:0.16 QI:0|1|0.66|1|1|1|3|0|181; (*GB) gi|132118|sp|P26573.1|RBS8_NICPL (e_value=2e-114) RecName: Full=Ribulose bisphosphate carboxylase small chain 8B, chlc
NbS00037852g0006.1 **Mass:** 20576 **Score:** 52 **Matches:** 3(3) **Sequences:** 3(3)
NbS00037852g0006.1 protein AED:0.20 eAED:0.21 QI:0|1|0.75|1|1|1|4|0|180; (*GB) gi|132118|sp|P26573.1|RBS8_NICPL (e_value=7e-123) RecName: Full=Ribulose bisphosphate carboxylase small chain 8B, chlc
NbS00041372g0008.1 **Mass:** 22328 **Score:** 52 **Matches:** 3(3) **Sequences:** 3(3)
NbS00041372g0008.1 protein AED:0.22 eAED:0.22 QI:8|0.66|0.5|1|1|1|4|0|197; (*SWP) sp|P26573|RBS8_NICPL (e_value=5e-110) Ribulose bisphosphate carboxylase small chain 8B, chloroplastic OS=Nicotiana
NbS00041372g0009.1 **Mass:** 15410 **Score:** 52 **Matches:** 3(3) **Sequences:** 3(3)
NbS00041372g0009.1 protein AED:0.23 eAED:0.24 QI:0|0.5|0|1|0.5|0.66|3|0|137; (*SWP) sp|P26573|RBS8_NICPL (e_value=7e-74) Ribulose bisphosphate carboxylase small chain 8B, chloroplastic OS=Nicotiana

---

78. NbS00019862g0010.1 **Mass:** 72039 **Score:** 51 **Matches:** 3(3) **Sequences:** 3(3) **emPAI:** 0.14
NbS00019862g0010.1 protein AED:0.10 eAED:0.10 QI:0|0.92|1|1|0.92|0.93|15|328|640; (*GB) gi|5931765|emb|CAB56619.1| (e_value=0.0) phragmoplastin [Nicotiana tabacum];; (*SWP) sp|Q39828|SDL5A_SOYBN (e

Query	Observed	Mr(expt)	Mr(calc)	ppm	Miss	Score	Expect	Rank	Unique	Peptide
14445	664.8800	1327.7454	1327.7401	4.03	0	37	0.0094	1		K.LQFPWIGVVNR.S
14945	**696.3443**	**1390.6740**	**1390.6728**	**0.87**	**0**	**37**	**0.016**	**1**	**U**	**R.SAQAEIDSVAWSK.-**
17574	567.2951	1698.8635	1698.8974	-19.98	1	33	0.028	1	U	K.QLGTLLDEDPAIMKR.R

---

79. NbS00012365g0017.1 **Mass:** 43392 **Score:** 51 **Matches:** 3(3) **Sequences:** 3(3) **emPAI:** 0.25
NbS00012365g0017.1 protein AED:0.10 eAED:0.10 QI:65|1|0.87|1|1|1|8|334|389; (*GB) gi|255558636|ref|XP_002520343.1| (e_value=0.0) replication factor C / DNA polymerase III gamma-tau subunit, putativ

Query	Observed	Mr(expt)	Mr(calc)	ppm	Miss	Score	Expect	Rank	Unique	Peptide
7414	**473.2900**	**944.5654**	**944.5655**	**-0.01**	**0**	**39**	**0.011**	**1**	**U**	**K.TSTILALAR.K**
11175	**478.3007**	**954.5869**	**954.5862**	**0.71**	**0**	**36**	**0.0072**	**1**	**U**	**K.GLALVDIVR.E**
13894	**640.3294**	**1278.6443**	**1278.6456**	**-1.02**	**0**	**32**	**0.055**	**1**	**U**	**K.NIIFSSSTADPK.A**

---

80. NbS00003662g0021.1 **Mass:** 29426 **Score:** 51 **Matches:** 2(1) **Sequences:** 2(1) **emPAI:** 0.24
NbS00003662g0021.1 protein AED:0.24 eAED:0.34 QI:0|0|0|1|0|0|3|0|257; (*GB) gi|78102516|ref|YP_358657.1| (e_value=6e-112) ATP synthase CF1 alpha subunit [Nicotiana sylvestris];; (*SWP) sp|Q3C1H4|A

Query	Observed	Mr(expt)	Mr(calc)	ppm	Miss	Score	Expect	Rank	Unique	Peptide
10038	**408.2344**	**814.4543**	**814.4548**	**-0.65**	**0**	**30**	**0.089**	**1**		**R.ELIIGDR.Q**
15672	**744.9041**	**1487.7937**	**1487.7944**	**-0.47**	**0**	**50**	**0.00069**	**1**	**U**	**K.ASSVAQVVTTLQER.G**

---

81. NbS00052235g0008.1 **Mass:** 61657 **Score:** 50 **Matches:** 2(1) **Sequences:** 2(1) **emPAI:** 0.11
NbS00052235g0008.1 protein AED:0.09 eAED:0.09 QI:76|0.66|0.5|1|1|1|4|0|542; (*GB) gi|350537917|ref|NP_001234829.1| (e_value=0.0) ascorbate oxidase precursor [Solanum lycopersicum];; (*SWP) sp|P247

Query	Observed	Mr(expt)	Mr(calc)	ppm	Miss	Score	Expect	Rank	Unique	Peptide
8546	**594.3300**	**1186.6454**	**1186.6492**	**-3.18**	**0**	**46**	**0.0036**	**1**	**U**	**R.VIVMLNTQNR.I**
13239	**603.3217**	**1204.6289**	**1204.6274**	**1.23**	**0**	**30**	**0.08**	**1**	**U**	**K.NYNLVNPIMK.N**

---

82. NbS00001148g0004.1 **Mass:** 36834 **Score:** 50 **Matches:** 2(2) **Sequences:** 2(2) **emPAI:** 0.19
NbS00001148g0004.1 protein AED:0.25 eAED:0.26 QI:0|0.66|0.5|1|0|0|4|455|319; (*GB) gi|226506550|ref|NP_001141544.1| (e_value=2e-158) uncharacterized protein LOC100273658 [Zea mays];; (*SWP) sp|P18(

Query	Observed	Mr(expt)	Mr(calc)	ppm	Miss	Score	Expect	Rank	Unique	Peptide
12680	**570.3515**	**1138.6885**	**1138.6862**	**1.93**	**0**	**40**	**0.002**	**1**	**U**	**K.LAVNLIPFPR.L**
13305	**608.2963**	**1214.5780**	**1214.5754**	**2.13**	**0**	**34**	**0.023**	**1**	**U**	**R.VSEQFTAMFR.R**

---

83. NbS00000565g0021.1 **Mass:** 59505 **Score:** 50 **Matches:** 1(1) **Sequences:** 1(1) **emPAI:** 0.06
NbS00000565g0021.1 protein AED:0.30 eAED:0.33 QI:204|0.83|0.94|1|0.77|0.84|19|0|515; (*SWP) sp|Q8R3N6|THOC1_MOUSE (e_value=1e-53) THO complex subunit 1 OS=Mus musculus GN=Thoc1 PE=1 SV=1;; (*TAIR)

Query	Observed	Mr(expt)	Mr(calc)	ppm	Miss	Score	Expect	Rank	Unique	Peptide
17457	**839.8962**	**1677.7778**	**1677.7747**	**1.86**	**0**	**50**	**0.00055**	**1**	**U**	**K.WADQYPNALTDTQR.V**

---

84. NbS00001859g0006.1 **Mass:** 58802 **Score:** 50 **Matches:** 3(2) **Sequences:** 3(2) **emPAI:** 0.18
NbS00001859g0006.1 protein AED:0.10 eAED:0.10 QI:0|0.42|0.5|1|0.85|0.75|8|0|558; (*GB) gi|313585890|gb|ADR71054.1| (e_value=0.0) phosphoglycerate kinase [Nicotiana benthamiana];; (*SWP) sp|Q42961|I

Query	Observed	Mr(expt)	Mr(calc)	ppm	Miss	Score	Expect	Rank	Unique	Peptide
12398	**552.3012**	**1102.5879**	**1102.5870**	**0.79**	**0**	**32**	**0.062**	**1**	**U**	**K.SVGDLTAAELK.G**
13180	**599.8734**	**1197.7322**	**1197.7333**	**-0.93**	**0**	**39**	**0.0029**	**1**	**U**	**R.LSELLGIQVVK.A**
16370	**787.4247**	**1572.8349**	**1572.8359**	**-0.63**	**0**	**36**	**0.017**	**1**	**U**	**K.GVTTIIGGGDSVAAVEK.V**

---

85. NbS00027742g0013.1 **Mass:** 39244 **Score:** 50 **Matches:** 2(2) **Sequences:** 2(2) **emPAI:** 0.18
NbS00027742g0013.1 protein AED:0.00 eAED:0.01 QI:0|1|1|1|1|1|7|309|363; (*GB) gi|77416977|gb|ABA81884.1| (e_value=4e-80) nuclear RNA binding protein-like [Solanum tuberosum];; (*TAIR) AT5G47210.1

Query	Observed	Mr(expt)	Mr(calc)	ppm	Miss	Score	Expect	Rank	Unique	Peptide
14712	680.8595	1359.7044	1359.7034	0.74	0	38	0.01	1	U	K.IEDVGQFPSLGAK.-
15203	475.6013	1423.7820	1423.7823	-0.21	0	35	0.014	1	U	K.FPSKPLPPSEAVR.E

---

86. NbS00035166g0003.1 **Mass:** 48617 **Score:** 49 **Matches:** 1(1) **Sequences:** 1(1) **emPAI:** 0.07
NbS00035166g0003.1 protein AED:0.07 eAED:0.07 QI:0|1|0.66|1|1|1|3|0|439; (*GB) gi|13785209|emb|CAC37356.1| (e_value=0.0) putative membrane protein [Solanum tuberosum];; (*SWP) sp|Q94BT2|AIR12_ARATH

Query	Observed	Mr(expt)	Mr(calc)	ppm	Miss	Score	Expect	Rank	Unique	Peptide
19669	1072.4980	2142.9815	2142.9778	1.76	0	49	0.00051	1	U	R.SSGTIDFASGQTSAAAGGASASSR.Q

---

87. NbS00045545g0004.1 **Mass:** 64751 **Score:** 49 **Matches:** 2(2) **Sequences:** 2(2) **emPAI:** 0.10
NbS00045545g0004.1 protein AED:0.13 eAED:0.13 QI:0|0.78|0.73|0.86|0.92|0.86|15|481|616; (*SWP) sp|Q7XTT4|NUCL2_ORYSJ (e_value=6e-73) Nucleolin 2 OS=Oryza sativa subsp. japonica GN=Os04g0620700 PE=2

Query	Observed	Mr(expt)	Mr(calc)	ppm	Miss	Score	Expect	Rank	Unique	Peptide
11630	505.2700	1008.5254	1008.5240	1.42	0	32	0.05	1	U	R.SEGTTIFVR.G
14137	649.8564	1297.6983	1297.6990	-0.50	0	41	0.0043	1	U	K.ALELNGQDLLGR.A

---

88. NbS00000139g0007.1 **Mass:** 35687 **Score:** 49 **Matches:** 1(1) **Sequences:** 1(1) **emPAI:** 0.09
NbS00000139g0007.1 protein AED:0.24 eAED:0.24 QI:362|1|0.88|1|1|1|9|573|324; (*GB) gi|543867|sp|P26360.2|ATPG3_IPOBA (e_value=0.0) RecName: Full=ATP synthase subunit gamma, mitochondrial; AltName:

Query	Observed	Mr(expt)	Mr(calc)	ppm	Miss	Score	Expect	Rank	Unique	Peptide
17161	820.3895	1638.7644	1638.7624	1.19	0	49	0.00075	1	U	K.LGELDSYEVEGAETK.A

Proteins matching the same set of peptides:
NbS00006501g0003.1 **Mass:** 35645 **Score:** 49 **Matches:** 1(1) **Sequences:** 1(1)
NbS00006501g0003.1 protein AED:0.21 eAED:0.21 QI:362|0.87|0.66|0.88|1|1|9|0|324; (*GB) gi|543867|sp|P26360.2|ATPG3_IPOBA (e_value=0.0) RecName: Full=ATP synthase subunit gamma, mitochondrial; AltNa
NbS00017858g0007.1 **Mass:** 17315 **Score:** 49 **Matches:** 1(1) **Sequences:** 1(1)
NbS00017858g0007.1 protein AED:0.24 eAED:0.24 QI:21|0.75|0.6|0.8|0.75|1|5|0|157; (*GB) gi|543867|sp|P26360.2|ATPG3_IPOBA (e_value=2e-100) RecName: Full=ATP synthase subunit gamma, mitochondrial; A
NbS00018418g0005.1 **Mass:** 36613 **Score:** 49 **Matches:** 1(1) **Sequences:** 1(1)
NbS00018418g0005.1 protein AED:0.19 eAED:0.19 QI:0|0.85|1|1|0.71|0.75|8|304|321; (*GB) gi|543867|sp|P26360.2|ATPG3_IPOBA (e_value=2e-146) RecName: Full=ATP synthase subunit gamma, mitochondrial; A

---

89. NbS00002125g0002.1 **Mass:** 26691 **Score:** 47 **Matches:** 1(1) **Sequences:** 1(1) **emPAI:** 0.13
NbS00002125g0002.1 protein AED:0.22 eAED:0.22 QI:0|-1|0|1|-1|1|1|0|229; (*GB) gi|47971184|dbj|BAD22534.1| (e_value=7e-143) harpin inducing protein 1-like 18 [Nicotiana tabacum];; (*SWP) sp|Q9C615|S

Query	Observed	Mr(expt)	Mr(calc)	ppm	Miss	Score	Expect	Rank	Unique	Peptide
14143	650.3337	1298.6528	1298.6506	1.67	0	47	0.0011	1	U	R.IGIYYDSIEAR.A

Proteins matching the same set of peptides:
NbS00002125g0008.1 **Mass:** 13456 **Score:** 47 **Matches:** 1(1) **Sequences:** 1(1)
NbS00002125g0008.1 protein AED:0.34 eAED:0.47 QI:0|0|0|1|1|1|2|0|118; (*GB) gi|47971184|dbj|BAD22534.1| (e_value=6e-62) harpin inducing protein 1-like 18 [Nicotiana tabacum];; (*SWP) sp|Q9C615|SYP2
NbS00002227g0002.1 **Mass:** 26701 **Score:** 47 **Matches:** 1(1) **Sequences:** 1(1)
NbS00002227g0002.1 protein AED:0.20 eAED:0.20 QI:0|-1|0|1|-1|1|1|0|229; (*GB) gi|47971184|dbj|BAD22534.1| (e_value=3e-145) harpin inducing protein 1-like 18 [Nicotiana tabacum];; (*SWP) sp|Q9C615|S
NbS00006593g0102.1 **Mass:** 26642 **Score:** 47 **Matches:** 1(1) **Sequences:** 1(1)
NbS00006593g0102.1 protein AED:0.14 eAED:0.14 QI:0|-1|0|1|-1|1|1|0|229
NbS00057808g0004.1 **Mass:** 31707 **Score:** 47 **Matches:** 1(1) **Sequences:** 1(1)
NbS00057808g0004.1 protein AED:0.28 eAED:0.28 QI:0|0|0|0.33|0|0.33|3|0|272; (*GB) gi|47971182|dbj|BAD22533.1| (e_value=2e-110) harpin inducing protein 1-like 9 [Nicotiana tabacum];; (*SWP) sp|Q9C6

---

90. NbS00000529g0002.1 **Mass:** 12164 **Score:** 47 **Matches:** 1(1) **Sequences:** 1(1) **emPAI:** 0.28
NbS00000529g0002.1 protein AED:0.44 eAED:0.48 QI:0|0.66|0.5|1|1|1|4|0|104; (*GB) gi|224068340|ref|XP_002302713.1| (e_value=7e-69) predicted protein [Populus trichocarpa];; (*SWP) sp|Q9VI10|SMD2_DRO

Query	Observed	Mr(expt)	Mr(calc)	ppm	Miss	Score	Expect	Rank	Unique	Peptide
13454	616.3138	1230.6131	1230.6139	-0.62	0	47	0.0015	1	U	K.NNTQVLINCR.N

Proteins matching the same set of peptides:
NbS00008935g0003.1 **Mass:** 12678 **Score:** 47 **Matches:** 1(1) **Sequences:** 1(1)
NbS00008935g0003.1 protein AED:0.00 eAED:0.00 QI:0|-1|0|1|-1|1|1|0|108; (*GB) gi|224068340|ref|XP_002302713.1| (e_value=2e-71) predicted protein [Populus trichocarpa];; (*SWP) sp|Q9VI10|SMD2_DROME
NbS00010757g0002.1 **Mass:** 12636 **Score:** 47 **Matches:** 1(1) **Sequences:** 1(1)

NbS00010757g0002.1 protein AED:0.41 eAED:0.42 QI:185|1|0.6|1|1|1|5|0|108; (*GB) gi|224068340|ref|XP_002302713.1| (e_value=5e-72) predicted protein [Populus trichocarpa];; (*SWP) sp|Q9VI10|SMD2_DROM

---

91. NbS00004081g0106.1 **Mass:** 29460 **Score:** 47 **Matches:** 1(1) **Sequences:** 1(1) **emPAI:** 0.11
NbS00004081g0106.1 protein AED:0.15 eAED:0.16 QI:0|1|0.75|1|1|1|4|0|260

Query	Observed	Mr(expt)	Mr(calc)	ppm	Miss	Score	Expect	Rank	Unique	Peptide
17225	824.9070	1647.7995	1647.7951	2.67	0	47	0.0013	1	U	K.VSNSLGSEELTVEER.N

Proteins matching the same set of peptides:
NbS00020600g0007.1 **Mass:** 29460 **Score:** 47 **Matches:** 1(1) **Sequences:** 1(1)
NbS00020600g0007.1 protein AED:0.15 eAED:0.15 QI:196|1|1|1|1|1|4|497|260; (*GB) gi|3023189|sp|P93343.1|1433C_TOBAC (e_value=0.0) RecName: Full=14-3-3-like protein C; AltName: Full=14-3-3-like prote

---

92. NbS00003163g0002.1 **Mass:** 22262 **Score:** 46 **Matches:** 2(2) **Sequences:** 1(1) **emPAI:** 0.15
NbS00003163g0002.1 protein ; (*GB) gi|146188483|emb|CAK12837.1| (e_value=1e-52) ribulose 1,5 biphosphate carboxylase/oxygenase [Liparia genistoides];; (*SWP) sp|P48709|RBL_NICDE (e_value=2e-52) Rib

Query	Observed	Mr(expt)	Mr(calc)	ppm	Miss	Score	Expect	Rank	Unique	Peptide
10572	445.2440	888.4734	888.4739	-0.58	0	43	0.0057	1	U	R.VALEACVK.A 10574

---

93. NbS00003380g0115.1 **Mass:** 26058 **Score:** 45 **Matches:** 2(2) **Sequences:** 2(2) **emPAI:** 0.27
NbS00003380g0115.1 protein AED:0.12 eAED:0.12 QI:355|0.66|0.85|1|0.33|0.42|7|134|238

Query	Observed	Mr(expt)	Mr(calc)	ppm	Miss	Score	Expect	Rank	Unique	Peptide
12283	546.2905	1090.5665	1090.5658	0.61	0	36	0.025	1	U	K.NPALYGELSK.G
13501	618.3089	1234.6032	1234.6016	1.35	0	34	0.035	1	U	R.NIANMVPAYDK.E

Proteins matching the same set of peptides:
NbS00016159g0006.1 **Mass:** 32702 **Score:** 45 **Matches:** 2(2) **Sequences:** 2(2)
NbS00016159g0006.1 protein AED:0.34 eAED:0.36 QI:356|0.57|0.5|1|0.71|0.62|8|0|302; (*GB) gi|115473|sp|P27141.1|CAHC_TOBAC (e_value=3e-179) RecName: Full=Carbonic anhydrase, chloroplastic; AltName:

---

94. NbS00005988g0006.1 **Mass:** 12425 **Score:** 45 **Matches:** 1(1) **Sequences:** 1(1) **emPAI:** 0.28
NbS00005988g0006.1 protein AED:0.37 eAED:0.39 QI:248|0|0.5|1|1|1|2|0|118; (*GB) gi|2497757|sp|Q42952.1|NLTP1_TOBAC (e_value=3e-62) RecName: Full=Non-specific lipid-transfer protein 1; Short=LTP 1;

Query	Observed	Mr(expt)	Mr(calc)	ppm	Miss	Score	Expect	Rank	Unique	Peptide
12965	587.3282	1172.6418	1172.6401	1.49	0	45	0.0023	1	U	K.SAAAAISGIDLGK.A

---

95. NbS00003291g0003.1 **Mass:** 25216 **Score:** 45 **Matches:** 1(1) **Sequences:** 1(1) **emPAI:** 0.13
NbS00003291g0003.1 protein AED:0.07 eAED:0.07 QI:121|0.83|0.85|1|1|1|7|382|226; (*TAIR) AT3G07030.1 (e_value=4e-47) | Symbols: | Alba DNA/RNA-binding protein | chr3:2223001-2225254 RVERSE LENGTH=4(

Query	Observed	Mr(expt)	Mr(calc)	ppm	Miss	Score	Expect	Rank	Unique	Peptide
16458	793.4152	1584.8159	1584.8147	0.73	0	45	0.0019	1	U	R.NYITYATTLLQER.R

Proteins matching the same set of peptides:
NbS00045053g0006.1 **Mass:** 26901 **Score:** 45 **Matches:** 1(1) **Sequences:** 1(1)
NbS00045053g0006.1 protein AED:0.04 eAED:0.04 QI:121|1|0.87|1|0.71|0.87|8|378|242; (*SWP) sp|Q8N5L8|RP25L_HUMAN (e_value=3e-17) Ribonuclease P protein subunit p25-like protein OS=Homo sapiens GN=RI

---

96. NbS00005976g0007.1 **Mass:** 29424 **Score:** 45 **Matches:** 2(1) **Sequences:** 2(1) **emPAI:** 0.24
NbS00005976g0007.1 protein AED:0.18 eAED:0.18 QI:185|0.87|0.88|1|0.87|0.88|9|435|268; (*GB) gi|145323882|ref|NP_001077530.1| (e_value=2e-169) glyceraldehyde 3-phosphate dehydrogenase [Arabidopsis t

Query	Observed	Mr(expt)	Mr(calc)	ppm	Miss	Score	Expect	Rank	Unique	Peptide
4747	417.2300	832.4454	832.4555	-12.12	0	32	0.13	1	U	K.IGINGFGR.I
15740	749.9296	1497.8446	1497.8403	2.85	0	34	0.016	1	U	R.VPTVDVSVVDLTVR.L

Proteins matching the same set of peptides:
NbS00007070g0009.1 **Mass:** 30214 **Score:** 45 **Matches:** 2(1) **Sequences:** 2(1)
NbS00007070g0009.1 protein AED:0.21 eAED:0.21 QI:415|0.75|0.88|0.88|0.87|0.88|9|0|275; (*GB) gi|257815169|gb|ACV69976.1| (e_value=0.0) NAD-dependent glyceraldehyde 3-P dehydrogenase [Solanum chacoe
NbS00011813g0009.1 **Mass:** 34280 **Score:** 45 **Matches:** 2(1) **Sequences:** 2(1)
NbS00011813g0009.1 protein AED:0.21 eAED:0.24 QI:185|0.63|0.83|1|0.90|0.83|12|369|310; (*GB) gi|157042763|gb|ABV02033.1| (e_value=5e-167) glyceraldehyde 3-phosphate dehydrogenase [Nicotiana langsdo
NbS00014820g0003.1 **Mass:** 31572 **Score:** 45 **Matches:** 2(1) **Sequences:** 2(1)
NbS00014820g0003.1 protein AED:0.15 eAED:0.15 QI:210|0.87|1|1|1|1|9|267|288; (*GB) gi|257815169|gb|ACV69976.1| (e_value=0.0) NAD-dependent glyceraldehyde 3-P dehydrogenase [Solanum chacoense];; (*S

---

97. >gi|74181742|dbj|BAE32582.1| **Mass:** 55528 **Score:** 44 **Matches:** 3(2) **Sequences:** 3(2) **emPAI:** 0.19
>gi|74181742|dbj|BAE32582.1| unnamed protein product [Mus musculus]

Query	Observed	Mr(expt)	Mr(calc)	ppm	Miss	Score	Expect	Rank	Unique	Peptide
10038	408.2344	814.4543	814.4548	-0.65	0	30	0.089	1		R.ELIIGDR.Q
11762	513.8000	1025.5855	1025.5869	-1.36	0	32	0.023	1		K.AVDSLVPIGR.G
11879	522.2902	1042.5659	1042.5659	0.00	0	33	0.038	1	U	R.TGSIVDVPAGK.A

98. NbS00010046g0020.1 **Mass:** 63701 **Score:** 44 **Matches:** 1(1) **Sequences:** 1(1) **emPAI:** 0.05
NbS00010046g0020.1 protein AED:0.25 eAED:0.25 QI:0|0.76|0.66|0.94|0.64|0.66|18|0|588; (*GB) gi|171854667|dbj|BAG16523.1| (e_value=0.0) putative NADPH oxidoreductase [Capsicum chinense];; (*SWP) sp

Query	Observed	Mr(expt)	Mr(calc)	ppm	Miss	Score	Expect	Rank	Unique	Peptide
17897	878.4365	1754.8585	1754.8588	-0.15	0	44	0.0022	1	U	K.VIYAGVNASDVNFSSGR.Y

Proteins matching the same set of peptides:
NbS00035112g0006.1 **Mass:** 67850 **Score:** 44 **Matches:** 1(1) **Sequences:** 1(1)
NbS00035112g0006.1 protein AED:0.21 eAED:0.22 QI:0|0.64|0.66|0.86|0.71|0.6|15|0|616; (*SWP) sp|Q8N4Q0|ZADH2_HUMAN (e_value=3e-51) Zinc-binding alcohol dehydrogenase domain-containing protein 2 OS=H

99. NbS00000058g0018.1 **Mass:** 24519 **Score:** 44 **Matches:** 2(2) **Sequences:** 2(2) **emPAI:** 0.29
NbS00000058g0018.1 protein AED:0.02 eAED:0.02 QI:0|1|0|1|1|1|2|0|221; (*GB) gi|30013659|gb|AAP03872.1| (e_value=2e-130) putative photosystem I subunit III precursor [Nicotiana tabacum];; (*SWP) sp

Query	Observed	Mr(expt)	Mr(calc)	ppm	Miss	Score	Expect	Rank	Unique	Peptide
10097	411.2475	820.4805	820.4807	-0.20	0	34	0.016	1	U	R.SYLIAVR.D
13393	612.8645	1223.7145	1223.7125	1.59	0	33	0.012	1	U	K.EIIIDVPLANK.L

Proteins matching the same set of peptides:
NbS00009051g0007.1 **Mass:** 27636 **Score:** 44 **Matches:** 2(2) **Sequences:** 2(2)
NbS00009051g0007.1 protein AED:0.31 eAED:0.31 QI:0|0.5|0|1|1|1|3|0|249; (*GB) gi|30013659|gb|AAP03872.1| (e_value=1e-120) putative photosystem I subunit III precursor [Nicotiana tabacum];; (*SWP) s
NbS00035792g0016.1 **Mass:** 24757 **Score:** 44 **Matches:** 2(2) **Sequences:** 2(2)
NbS00035792g0016.1 protein AED:0.06 eAED:0.06 QI:0|0|0|1|1|1|2|0|224; (*GB) gi|30013659|gb|AAP03872.1| (e_value=3e-103) putative photosystem I subunit III precursor [Nicotiana tabacum];; (*SWP) sp
NbS00061093g0007.1 **Mass:** 22605 **Score:** 44 **Matches:** 2(2) **Sequences:** 2(2)
NbS00061093g0007.1 protein AED:0.06 eAED:0.06 QI:0|0|0|1|0|0.5|2|0|206; (*GB) gi|30013659|gb|AAP03872.1| (e_value=3e-90) putative photosystem I subunit III precursor [Nicotiana tabacum];; (*SWP) sp

100. NbS00059497g0003.1 **Mass:** 77510 **Score:** 43 **Matches:** 2(2) **Sequences:** 1(1) **emPAI:** 0.04
NbS00059497g0003.1 protein AED:0.17 eAED:0.17 QI:0|1|1|1|1|1|3|550|697; (*SWP) sp|Q653H7|ARFR_ORYSJ (e_value=0.0) Auxin response factor 18 OS=Oryza sativa subsp. japonica GN=ARF18 PE=2 SV=1;; (*TA

Query	Observed	Mr(expt)	Mr(calc)	ppm	Miss	Score	Expect	Rank	Unique	Peptide
10227	421.7582	841.5019	841.5022	-0.34	0	40	0.0054	2	U	R.GLTVSLPR.D 10223

101. NbS00033024g0001.1 **Mass:** 61550 **Score:** 43 **Matches:** 1(1) **Sequences:** 1(1) **emPAI:** 0.05
NbS00033024g0001.1 protein AED:0.17 eAED:0.17 QI:0|0.85|0.87|1|1|1|8|260|540; (*GB) gi|255554849|ref|XP_002518462.1| (e_value=0.0) multicopper oxidase, putative [Ricinus communis];; (*SWP) sp|Q006

Query	Observed	Mr(expt)	Mr(calc)	ppm	Miss	Score	Expect	Rank	Unique	Peptide
17980	884.4437	1766.8728	1766.8727	0.05	0	43	0.0032	1	U	R.YAVNSVSYVDPDTPLK.L

102. NbS00002372g0015.1 **Mass:** 50828 **Score:** 43 **Matches:** 2(1) **Sequences:** 2(1) **emPAI:** 0.13
NbS00002372g0015.1 protein AED:0.22 eAED:0.23 QI:207|0.66|0.5|1|1|1|4|0|452; (*GB) gi|110083391|dbj|BAE97400.1| (e_value=8e-141) heat shock protein 90 [Nicotiana tabacum];; (*SWP) sp|P36181|HSP80_S

Query	Observed	Mr(expt)	Mr(calc)	ppm	Miss	Score	Expect	Rank	Unique	Peptide
12643	567.2964	1132.5783	1132.5764	1.70	0	30	0.093	1	U	K.AVENSPFLEK.L
13663	628.8492	1255.6838	1255.6884	-3.68	0	40	0.0066	1	U	K.ADLVNNLGTIAR.S

Proteins matching the same set of peptides:
NbS00014845g0001.1 **Mass:** 52186 **Score:** 43 **Matches:** 2(1) **Sequences:** 2(1)
NbS00014845g0001.1 protein AED:0.19 eAED:0.19 QI:0|0.66|0.25|1|1|1|4|0|461; (*GB) gi|47219165|emb|CAG01828.1| (e_value=1e-174) unnamed protein product [Tetraodon nigroviridis];; (*SWP) sp|P36181|HS
NbS00021897g0010.1 **Mass:** 67830 **Score:** 43 **Matches:** 2(1) **Sequences:** 2(1)
NbS00021897g0010.1 protein AED:0.13 eAED:0.13 QI:0|0.33|0.25|1|0.66|0.5|4|0|595; (*GB) gi|110083391|dbj|BAE97400.1| (e_value=2e-133) heat shock protein 90 [Nicotiana tabacum];; (*SWP) sp|P36181|HSI
NbS00025260g0001.1 **Mass:** 52067 **Score:** 43 **Matches:** 2(1) **Sequences:** 2(1)
NbS00025260g0001.1 protein AED:0.23 eAED:0.23 QI:0|0.66|0.25|1|1|1|4|0|460; (*GB) gi|47219165|emb|CAG01828.1| (e_value=3e-173) unnamed protein product [Tetraodon nigroviridis];; (*SWP) sp|P36181|HS

103. NbS00008558g0004.1 **Mass:** 43072 **Score:** 43 **Matches:** 2(1) **Sequences:** 2(1) **emPAI:** 0.16
NbS00008558g0004.1 protein AED:0.13 eAED:0.13 QI:0|1|0.8|1|1|1|5|0|398; (*GB) gi|4827253|dbj|BAA77603.1| (e_value=0.0) plastidic aldolase [Nicotiana paniculata];; (*SWP) sp|Q944G9|ALFC2_ARATH (e_va

Query	Observed	Mr(expt)	Mr(calc)	ppm	Miss	Score	Expect	Rank	Unique	Peptide
11195	479.7698	957.5250	957.5243	0.70	0	31	0.067	1	U	K.AAQDALLTR.A
14912	694.3650	1386.7154	1386.7103	3.72	0	40	0.008	1	U	R.LASIGLENTEANR.Q

Proteins matching the same set of peptides:
NbS00028064g0012.1 **Mass:** 43060 **Score:** 43 **Matches:** 2(1) **Sequences:** 2(1)
NbS00028064g0012.1 protein AED:0.27 eAED:0.27 QI:0|1|0.8|1|1|1|5|0|398; (*GB) gi|4827253|dbj|BAA77603.1| (e_value=0.0) plastidic aldolase [Nicotiana paniculata];; (*SWP) sp|Q944G9|ALFC2_ARATH (e_va

---

104. NbS00001259g0009.1 **Mass:** 35402 **Score:** 43 **Matches:** 1(1) **Sequences:** 1(1) **emPAI:** 0.09
NbS00001259g0009.1 protein AED:0.04 eAED:0.04 QI:128|0.75|0.77|1|0.75|0.88|9|0|318; (*GB) gi|357505379|ref|XP_003622978.1| (e_value=2e-55) Ribonuclease P protein subunit p25 [Medicago truncatula];;

Query	Observed	Mr(expt)	Mr(calc)	ppm	Miss	Score	Expect	Rank	Unique	Peptide
17186	822.4011	1642.7876	1642.7852	1.45	0	43	0.003	1	U	R.YNYQPQQQQPPPR.H

Proteins matching the same set of peptides:
NbS00012174g0007.1 **Mass:** 27249 **Score:** 43 **Matches:** 1(1) **Sequences:** 1(1)
NbS00012174g0007.1 protein AED:0.23 eAED:0.26 QI:0|0.85|0.75|1|0.71|0.87|8|0|245; (*GB) gi|357505379|ref|XP_003622978.1| (e_value=8e-62) Ribonuclease P protein subunit p25 [Medicago truncatula];;

---

105. NbS00014241g0002.1 **Mass:** 21890 **Score:** 42 **Matches:** 1(1) **Sequences:** 1(1) **emPAI:** 0.15
NbS00014241g0002.1 protein AED:0.09 eAED:0.09 QI:0|0|0|1|0|0|2|0|201; (*SWP) sp|P00823|ATPA_TOBAC (e_value=5e-67) ATP synthase subunit alpha, chloroplastic OS=Nicotiana tabacum GN=atpA PE=2 SV=2;;

Query	Observed	Mr(expt)	Mr(calc)	ppm	Miss	Score	Expect	Rank	Unique	Peptide
11792	515.7798	1029.5451	1029.5454	-0.29	0	42	0.0049	1	U	R.ADEISNIIR.E

---

106. NbS00007742g0006.1 **Mass:** 33872 **Score:** 42 **Matches:** 1(1) **Sequences:** 1(1) **emPAI:** 0.10
NbS00007742g0006.1 protein AED:0.40 eAED:0.42 QI:0|0.5|0.42|1|1|1|7|0|319; (*GB) gi|94466659|emb|CAJ44458.1| (e_value=2e-110) ALY protein [Nicotiana benthamiana];; (*SWP) sp|Q9JJW6|REFP2_MOUSE (e_v

Query	Observed	Mr(expt)	Mr(calc)	ppm	Miss	Score	Expect	Rank	Unique	Peptide
17869	875.9536	1749.8927	1749.8897	1.68	0	42	0.0037	1	U	K.VYVSNLDVGVTNSDIR.V

Proteins matching the same set of peptides:
NbS00036740g0010.1 **Mass:** 28237 **Score:** 42 **Matches:** 1(1) **Sequences:** 1(1)
NbS00036740g0010.1 protein AED:0.03 eAED:0.03 QI:330|0.83|0.85|1|0.5|0.42|7|0|263; (*GB) gi|94466659|emb|CAJ44458.1| (e_value=3e-116) ALY protein [Nicotiana benthamiana];; (*SWP) sp|B5FXN8|THOC4_TA

---

107. NbS00001538g0002.1 **Mass:** 88204 **Score:** 42 **Matches:** 1(1) **Sequences:** 1(1) **emPAI:** 0.04
NbS00001538g0002.1 protein AED:0.19 eAED:0.19 QI:248|1|1|1|1|1|11|261|788; (*SWP) sp|Q7TP47|HNRPQ_RAT (e_value=2e-31) Heterogeneous nuclear ribonucleoprotein Q OS=Rattus norvegicus GN=Syncrip PE=2

Query	Observed	Mr(expt)	Mr(calc)	ppm	Miss	Score	Expect	Rank	Unique	Peptide
16330	783.8364	1565.6582	1565.6594	-0.78	0	42	0.0014	1	U	R.GSDVGGSSYSSAYSSR.G

---

108. NbS00001587g0026.1 **Mass:** 107641 **Score:** 41 **Matches:** 2(2) **Sequences:** 2(2) **emPAI:** 0.06
NbS00001587g0026.1 protein AED:0.19 eAED:0.20 QI:234|0.91|0.83|1|0.82|0.83|24|284|993; (*SWP) sp|P35601|RFC1_MOUSE (e_value=5e-125) Replication factor C subunit 1 OS=Mus musculus GN=Rfc1 PE=1 SV=2;

Query	Observed	Mr(expt)	Mr(calc)	ppm	Miss	Score	Expect	Rank	Unique	Peptide
12444	554.7844	1107.5542	1107.5560	-1.68	0	37	0.018	1	U	K.SVIQYDDIR.Q
15441	729.8812	1457.7479	1457.7474	0.35	0	33	0.034	1	U	K.ELVSNESLGANIGR.S

Proteins matching the same set of peptides:
NbS00012440g0009.1 **Mass:** 110612 **Score:** 41 **Matches:** 2(2) **Sequences:** 2(2)
NbS00012440g0009.1 protein AED:0.21 eAED:0.21 QI:69|0.95|0.85|1|0.95|0.95|21|282|1019; (*GB) gi|296083902|emb|CBI24290.3| (e_value=0.0) unnamed protein product [Vitis vinifera];; (*SWP) sp|P35601|I

---

109. NbS00000548g0008.1 **Mass:** 40225 **Score:** 41 **Matches:** 1(1) **Sequences:** 1(1) **emPAI:** 0.08
NbS00000548g0008.1 protein AED:0.29 eAED:0.30 QI:0|0.8|0.5|1|1|0.66|6|0|363; (*GB) gi|255573386|ref|XP_002527619.1| (e_value=2e-130) Ras-GTPase-activating protein-binding protein, putative [Ricinus

Query	Observed	Mr(expt)	Mr(calc)	ppm	Miss	Score	Expect	Rank	Unique	Peptide
13438	615.3185	1228.6225	1228.6241	-1.25	0	41	0.0059	1	U	K.GYFVLNDVFR.Y

Proteins matching the same set of peptides:
NbS00030703g0003.1 **Mass:** 53003 **Score:** 41 **Matches:** 1(1) **Sequences:** 1(1)
NbS00030703g0003.1 protein AED:0.29 eAED:0.29 QI:0|0.71|0.5|1|0.71|0.75|8|366|482; (*GB) gi|255573386|ref|XP_002527619.1| (e_value=9e-142) Ras-GTPase-activating protein-binding protein, putative [I

110. NbS00023006g0207.1 **Mass:** 9109 **Score:** 41 **Matches:** 1(1) **Sequences:** 1(1) **emPAI:** 0.38
NbS00023006g0207.1 protein

Query	Observed	Mr(expt)	Mr(calc)	ppm	Miss	Score	Expect	Rank	Unique	Peptide
11898	523.3062	1044.5978	1044.5968	0.94	0	41	0.0054	1	U	K.VVDLLAPYR.R

111. NbS00001970g0004.1 **Mass:** 33561 **Score:** 40 **Matches:** 1(1) **Sequences:** 1(1) **emPAI:** 0.10
NbS00001970g0004.1 protein AED:0.12 eAED:0.12 QI:0|1|0.8|1|1|1|5|253|296; (*GB) gi|82400118|gb|ABB72798.1| (e_value=3e-171) 40S ribosomal protein S4-like protein [Solanum tuberosum];; (*SWP) sp|P46

Query	Observed	Mr(expt)	Mr(calc)	ppm	Miss	Score	Expect	Rank	Unique	Peptide
11087	474.7793	947.5440	947.5440	-0.04	0	40	0.0052	1	U	R.LGNVFTLGK.G

Proteins matching the same set of peptides:
NbS00004310g0005.1 **Mass:** 32971 **Score:** 40 **Matches:** 1(1) **Sequences:** 1(1)
NbS00004310g0005.1 protein AED:0.20 eAED:0.22 QI:0|0.5|0.2|0.8|1|1|5|0|289; (*GB) gi|1173257|sp|P46300.1|RS4_SOLTU (e_value=1e-166) RecName: Full=40S ribosomal protein S4;; (*SWP) sp|P46300|RS4_SOI
NbS00005958g0001.1 **Mass:** 26537 **Score:** 40 **Matches:** 1(1) **Sequences:** 1(1)
NbS00005958g0001.1 protein AED:0.20 eAED:0.20 QI:9|0.66|0.75|1|1|1|4|0|236; (*GB) gi|82400118|gb|ABB72798.1| (e_value=2e-148) 40S ribosomal protein S4-like protein [Solanum tuberosum];; (*SWP) sp|I
NbS00012660g0015.1 **Mass:** 33433 **Score:** 40 **Matches:** 1(1) **Sequences:** 1(1)
NbS00012660g0015.1 protein AED:0.29 eAED:0.37 QI:0|0.6|0.33|1|0.6|0.66|6|0|295; (*GB) gi|82400118|gb|ABB72798.1| (e_value=2e-172) 40S ribosomal protein S4-like protein [Solanum tuberosum];; (*SWP)
NbS00013392g0001.1 **Mass:** 30022 **Score:** 40 **Matches:** 1(1) **Sequences:** 1(1)
NbS00013392g0001.1 protein AED:0.17 eAED:0.17 QI:0|0.75|0.6|0.8|1|1|5|0|264; (*GB) gi|1173257|sp|P46300.1|RS4_SOLTU (e_value=4e-173) RecName: Full=40S ribosomal protein S4;; (*SWP) sp|P46300|RS4_SO
NbS00020944g0004.1 **Mass:** 32588 **Score:** 40 **Matches:** 1(1) **Sequences:** 1(1)
NbS00020944g0004.1 protein AED:0.04 eAED:0.04 QI:0|0.5|0.2|0.8|1|1|5|0|288; (*GB) gi|82400118|gb|ABB72798.1| (e_value=1e-168) 40S ribosomal protein S4-like protein [Solanum tuberosum];; (*SWP) sp|I
NbS00028790g0004.1 **Mass:** 28224 **Score:** 40 **Matches:** 1(1) **Sequences:** 1(1)
NbS00028790g0004.1 protein AED:0.16 eAED:0.17 QI:453|0.66|0.5|1|1|1|4|0|250; (*GB) gi|82400118|gb|ABB72798.1| (e_value=9e-155) 40S ribosomal protein S4-like protein [Solanum tuberosum];; (*SWP) sp
NbS00033090g0004.1 **Mass:** 34515 **Score:** 40 **Matches:** 1(1) **Sequences:** 1(1)
NbS00033090g0004.1 protein AED:0.13 eAED:0.15 QI:0|0.6|0.66|1|0.8|0.66|6|235|303; (*GB) gi|82400118|gb|ABB72798.1| (e_value=1e-166) 40S ribosomal protein S4-like protein [Solanum tuberosum];; (*SWI

112. NbS00010947g0011.1 **Mass:** 60098 **Score:** 40 **Matches:** 1(1) **Sequences:** 1(1) **emPAI:** 0.05
NbS00010947g0011.1 protein AED:0.21 eAED:0.21 QI:0|1|0.88|1|0.87|0.77|9|197|534; (*SWP) sp|Q9FZL3|MGDG_TOBAC (e_value=0.0) Probable monogalactosyldiacylglycerol synthase, chloroplastic OS=Nicotiana

Query	Observed	Mr(expt)	Mr(calc)	ppm	Miss	Score	Expect	Rank	Unique	Peptide
12149	537.2584	1072.5023	1072.5011	1.07	0	40	0.0046	1	U	R.MTYYATAPR.L

Proteins matching the same set of peptides:
NbS00013573g0012.1 **Mass:** 68150 **Score:** 40 **Matches:** 1(1) **Sequences:** 1(1)
NbS00013573g0012.1 protein AED:0.29 eAED:0.29 QI:0|0.66|0.6|0.9|0.77|0.9|10|0|606; (*GB) gi|75173454|sp|Q9FZL3.1|MGDG_TOBAC (e_value=0.0) RecName: Full=Probable monogalactosyldiacylglycerol synthas

113. NbS00059407g0001.1 **Mass:** 15599 **Score:** 40 **Matches:** 1(1) **Sequences:** 1(1) **emPAI:** 0.22
NbS00059407g0001.1 protein AED:0.31 eAED:0.31 QI:0|0|0|1|1|1|3|0|138; (*SWP) sp|P06005|PSBD_SPIOL (e_value=4e-44) Photosystem II D2 protein OS=Spinacia oleracea GN=psbD PE=1 SV=3;; (*ITAG) ATCG002

Query	Observed	Mr(expt)	Mr(calc)	ppm	Miss	Score	Expect	Rank	Unique	Peptide
11863	521.3064	1040.5982	1040.5978	0.41	0	40	0.0039	1	U	K.NILLNEGIR.A

114. NbS00016165g0010.1 **Mass:** 29526 **Score:** 40 **Matches:** 1(1) **Sequences:** 1(1) **emPAI:** 0.11
NbS00016165g0010.1 protein AED:0.19 eAED:0.19 QI:75|0|0.5|1|1|1|2|0|264; (*GB) gi|121663827|dbj|BAF44533.1| (e_value=9e-166) class IV chitinase [Nicotiana tabacum];; (*SWP) sp|Q6K8R2|CHI6_ORYSJ (e_

Query	Observed	Mr(expt)	Mr(calc)	ppm	Miss	Score	Expect	Rank	Unique	Peptide
16471	794.9031	1587.7917	1587.7893	1.53	0	40	0.0065	1	U	K.SIGFDGLNDPDIVAR.D

115. NbS00002028g0009.1 **Mass:** 21961 **Score:** 40 **Matches:** 1(1) **Sequences:** 1(1) **emPAI:** 0.15
NbS00002028g0009.1 protein AED:0.09 eAED:0.09 QI:0|0|0|0.5|1|1|2|0|201; (*GB) gi|225452646|ref|XP_002281856.1| (e_value=4e-122) PREDICTED: protein SEC13 homolog [Vitis vinifera];; (*SWP) sp|Q9D1M0

Query	Observed	Mr(expt)	Mr(calc)	ppm	Miss	Score	Expect	Rank	Unique	Peptide
13902	640.8551	1279.6957	1279.6925	2.50	0	40	0.0055	1	U	R.DVAWAPNLGLPK.T

Proteins matching the same set of peptides:
NbS00005187g0006.1 **Mass:** 32833 **Score:** 40 **Matches:** 1(1) **Sequences:** 1(1)
NbS00005187g0006.1 protein AED:0.00 eAED:0.00 QI:0|-1|0|1|-1|1|1|0|302; (*GB) gi|83283979|gb|ABC01897.1| (e_value=0.0) protein transport SEC13-like protein [Solanum tuberosum];; (*SWP) sp|Q5XFW8|SE
NbS00005404g0013.1 **Mass:** 32755 **Score:** 40 **Matches:** 1(1) **Sequences:** 1(1)
NbS00005404g0013.1 protein AED:0.31 eAED:0.31 QI:0|1|0.5|1|1|1|2|0|301; (*GB) gi|356567320|ref|XP_003551869.1| (e_value=0.0) PREDICTED: protein SEC13 homolog [Glycine max];; (*SWP) sp|Q9D1M0|SEC13_
NbS00005571g0003.1 **Mass:** 34440 **Score:** 40 **Matches:** 1(1) **Sequences:** 1(1)
NbS00005571g0003.1 protein AED:0.03 eAED:0.04 QI:0|0|0|1|1|1|2|0|317; (*GB) gi|83283979|gb|ABC01897.1| (e_value=0.0) protein transport SEC13-like protein [Solanum tuberosum];; (*SWP) sp|Q9D1M0|SEC1
NbS00023949g0003.1 **Mass:** 24534 **Score:** 40 **Matches:** 1(1) **Sequences:** 1(1)
NbS00023949g0003.1 protein AED:0.12 eAED:0.12 QI:0|0|0|1|1|1|3|0|225; (*GB) gi|83283979|gb|ABC01897.1| (e_value=1e-89) protein transport SEC13-like protein [Solanum tuberosum];; (*SWP) sp|Q3ZCC9|SE
NbS00044664g0001.1 **Mass:** 32893 **Score:** 40 **Matches:** 1(1) **Sequences:** 1(1)
NbS00044664g0001.1 protein AED:0.35 eAED:0.35 QI:0|-1|0|1|-1|1|1|0|302; (*GB) gi|83283979|gb|ABC01897.1| (e_value=0.0) protein transport SEC13-like protein [Solanum tuberosum];; (*SWP) sp|Q9D1M0|SE

---

116. NbS00000215g0010.1 **Mass:** 64850 **Score:** 40 **Matches:** 1(1) **Sequences:** 1(1) **emPAI:** 0.05
NbS00000215g0010.1 protein AED:0.11 eAED:0.11 QI:211|1|1|1|0.85|0.8|15|450|605; (*GB) gi|225442531|ref|XP_002284134.1| (e_value=0.0) PREDICTED: ruBisCO large subunit-binding protein subunit beta, c

Query	Observed	Mr(expt)	Mr(calc)	ppm	Miss	Score	Expect	Rank	Unique	Peptide
13800	635.8539	1269.6932	1269.6928	0.29	0	40	0.0062	1	U	R.DLVNVLEEAIR.G

Proteins matching the same set of peptides:
NbS00007027g0009.1 **Mass:** 64681 **Score:** 40 **Matches:** 1(1) **Sequences:** 1(1)
NbS00007027g0009.1 protein AED:0.13 eAED:0.13 QI:197|1|1|1|0.92|0.86|15|612|605; (*GB) gi|225442531|ref|XP_002284134.1| (e_value=0.0) PREDICTED: ruBisCO large subunit-binding protein subunit beta,

---

117. NbS00018705g0023.1 **Mass:** 7957 **Score:** 40 **Matches:** 1(1) **Sequences:** 1(1) **emPAI:** 0.44
NbS00018705g0023.1 protein AED:0.30 eAED:0.31 QI:0|0.66|0.75|1|0.33|0.75|4|388|71; (*GB) gi|242050850|ref|XP_002463169.1| (e_value=7e-39) hypothetical protein SORBIDRAFT_02g039000 [Sorghum bicolor]

Query	Observed	Mr(expt)	Mr(calc)	ppm	Miss	Score	Expect	Rank	Unique	Peptide
16271	777.9283	1553.8420	1553.8413	0.46	0	40	0.005	1	U	R.GNSVVTIEALEPVAR.A

Proteins matching the same set of peptides:
NbS00024371g0006.1 **Mass:** 8908 **Score:** 40 **Matches:** 1(1) **Sequences:** 1(1)
NbS00024371g0006.1 protein AED:0.00 eAED:0.00 QI:102|1|1|1|1|1|4|366|80; (*GB) gi|242050850|ref|XP_002463169.1| (e_value=4e-48) hypothetical protein SORBIDRAFT_02g039000 [Sorghum bicolor];; (*SWP)

---

118. NbS00000456g0109.1 **Mass:** 14552 **Score:** 39 **Matches:** 1(1) **Sequences:** 1(1) **emPAI:** 0.23
NbS00000456g0109.1 protein AED:0.20 eAED:0.20 QI:215|1|1|1|0.6|0.66|6|685|132

Query	Observed	Mr(expt)	Mr(calc)	ppm	Miss	Score	Expect	Rank	Unique	Peptide
12440	554.3008	1106.5870	1106.5868	0.20	0	39	0.0085	1	U	R.FMIIPDMLK.N

Proteins matching the same set of peptides:
NbS00018940g0019.1 **Mass:** 12140 **Score:** 39 **Matches:** 1(1) **Sequences:** 1(1)
NbS00018940g0019.1 protein AED:0.20 eAED:0.21 QI:9|1|0.8|1|1|1|5|626|110; (*SWP) sp|P62323|SMD3_XENLA (e_value=4e-30) Small nuclear ribonucleoprotein Sm D3 OS=Xenopus laevis GN=snrpd3 PE=2 SV=1;;
NbS00029347g0013.1 **Mass:** 9248 **Score:** 39 **Matches:** 1(1) **Sequences:** 1(1)
NbS00029347g0013.1 protein AED:0.29 eAED:0.29 QI:179|0.6|0.66|1|0.8|0.66|6|0|87; (*GB) gi|223975955|gb|ACN32165.1| (e_value=4e-25) unknown [Zea mays];; (*SWP) sp|P62323|SMD3_XENLA (e_value=3e-15) S

---

119. NbS00000485g0008.1 **Mass:** 26716 **Score:** 39 **Matches:** 2(1) **Sequences:** 2(1) **emPAI:** 0.26
NbS00000485g0008.1 protein AED:0.27 eAED:0.27 QI:81|1|1|1|0.8|0.83|6|329|238; (*GB) gi|225442156|ref|XP_002275541.1| (e_value=6e-155) PREDICTED: 40S ribosomal protein S3-3 [Vitis vinifera];; (*SWP)

Query	Observed	Mr(expt)	Mr(calc)	ppm	Miss	Score	Expect	Rank	Unique	Peptide
13833	637.8286	1273.6426	1273.6449	-1.81	0	32	0.051	1	U	R.GLCAVAQAESLR.Y
9384	712.3400	1422.6654	1422.6627	1.95	0	36	0.037	1	U	R.ELAEDGYSGVEVR.V

Proteins matching the same set of peptides:
NbS00003420g0016.1 **Mass:** 26587 **Score:** 39 **Matches:** 2(1) **Sequences:** 2(1)
NbS00003420g0016.1 protein AED:0.15 eAED:0.15 QI:81|1|1|1|0.8|1|6|333|237; (*GB) gi|358248410|ref|NP_001239621.1| (e_value=5e-156) uncharacterized protein LOC100808705 [Glycine max];; (*SWP) sp|Q9
NbS00035854g0012.1 **Mass:** 29045 **Score:** 39 **Matches:** 2(1) **Sequences:** 2(1)
NbS00035854g0012.1 protein AED:0.09 eAED:0.09 QI:143|1|0.85|1|0.83|0.71|7|0|260; (*GB) gi|225444782|ref|XP_002279950.1| (e_value=5e-151) PREDICTED: 40S ribosomal protein S3-3 [Vitis vinifera];; (*S

---

120. NbS00007843g0002.1 **Mass:** 90945 **Score:** 39 **Matches:** 1(1) **Sequences:** 1(1) **emPAI:** 0.04
NbS00007843g0002.1 protein AED:0.14 eAED:0.14 QI:0|0.76|0.78|1|0.84|0.85|14|2042|813; (*GB) gi|359489218|ref|XP_002270340.2| (e_value=0.0) PREDICTED: uncharacterized protein LOC100232913 [Vitis vir

Query	Observed	Mr(expt)	Mr(calc)	ppm	Miss	Score	Expect	Rank	Unique	Peptide
13483	617.8174	1233.6202	1233.6176	2.12	0	39	0.011	1	U	R.DVMFGVDRPAK.V

Proteins matching the same set of peptides:
NbS00010828g0006.1 **Mass:** 86749 **Score:** 39 **Matches:** 1(1) **Sequences:** 1(1)
NbS00010828g0006.1 protein AED:0.20 eAED:0.20 QI:0|0.8|0.63|0.90|0.9|0.90|11|0|770; (*GB) gi|359386142|gb|AEV43360.1| (e_value=0.0) RNA recognition motif protein 1 [Citrus sinensis];; (*SWP) sp|Q7!

---

121. NbS00008232g0008.1 **Mass:** 64319 **Score:** 39 **Matches:** 1(1) **Sequences:** 1(1) **emPAI:** 0.05
NbS00008232g0008.1 protein AED:0.05 eAED:0.09 QI:72|0.85|0.86|1|0.71|0.86|15|519|570; (*GB) gi|359483518|ref|XP_002272702.2| (e_value=0.0) PREDICTED: apoptosis inhibitor 5-like [Vitis vinifera];;

Query	Observed	Mr(expt)	Mr(calc)	ppm	Miss	Score	Expect	Rank	Unique	Peptide
17231	825.3930	1648.7715	1648.7693	1.33	0	39	0.0074	1	U	K.NLAESAPYTTPQDSR.Q

---

122. NbS00027615g0006.1 **Mass:** 29709 **Score:** 39 **Matches:** 1(1) **Sequences:** 1(1) **emPAI:** 0.11
NbS00027615g0006.1 protein AED:0.24 eAED:0.24 QI:233|1|1|1|1|1|6|475|276; (*GB) gi|161788876|dbj|BAF95072.1| (e_value=0.0) voltage-dependent anion channel [Nicotiana tabacum];; (*SWP) sp|P42055|VDA

Query	Observed	Mr(expt)	Mr(calc)	ppm	Miss	Score	Expect	Rank	Unique	Peptide
18855	966.5037	1930.9928	1930.9888	2.06	0	39	0.0072	1	U	K.FTITTYSPTGVAITSSGTK.K

Proteins matching the same set of peptides:
NbC25338748g0001.1 **Mass:** 7166 **Score:** 39 **Matches:** 1(1) **Sequences:** 1(1)
NbC25338748g0001.1 protein ; (*GB) gi|161788876|dbj|BAF95072.1| (e_value=3e-38) voltage-dependent anion channel [Nicotiana tabacum];; (*SWP) sp|P42055|VDAC1_SOLTU (e_value=2e-37) Mitochondrial oute

---

123. NbS00004901g0005.1 **Mass:** 43708 **Score:** 39 **Matches:** 1(1) **Sequences:** 1(1) **emPAI:** 0.08
NbS00004901g0005.1 protein AED:0.05 eAED:0.05 QI:0|1|0.5|1|1|1|4|0|391; (*GB) gi|231503|sp|P30171.1|ACT11_SOLTU (e_value=0.0) RecName: Full=Actin-97;; (*SWP) sp|P30171|ACT11_SOLTU (e_value=0.0) Act

Query	Observed	Mr(expt)	Mr(calc)	ppm	Miss	Score	Expect	Rank	Unique	Peptide
18003	887.9534	1773.8923	1773.8897	1.47	0	39	0.0088	1	U	K.NYELPDGQVITIGAER.F

Proteins matching the same set of peptides:
NbS00005317g0101.1 **Mass:** 41959 **Score:** 39 **Matches:** 1(1) **Sequences:** 1(1)
NbS00005317g0101.1 protein AED:0.21 eAED:0.21 QI:16|1|1|1|1|1|4|419|377
NbS00009856g0015.1 **Mass:** 40646 **Score:** 39 **Matches:** 1(1) **Sequences:** 1(1)
NbS00009856g0015.1 protein AED:0.09 eAED:0.10 QI:11|1|0.33|1|1|1|3|0|367; (*GB) gi|342837524|dbj|BAK57343.1| (e_value=0.0) actin1 [Morella rubra];; (*SWP) sp|P53492|ACT7_ARATH (e_value=0.0) Actin-
NbS00011031g0002.1 **Mass:** 41871 **Score:** 39 **Matches:** 1(1) **Sequences:** 1(1)
NbS00011031g0002.1 protein AED:0.14 eAED:0.14 QI:236|1|1|1|0.75|0.6|5|154|377; (*GB) gi|231503|sp|P30171.1|ACT11_SOLTU (e_value=0.0) RecName: Full=Actin-97;; (*SWP) sp|P30171|ACT11_SOLTU (e_value=
NbS00017585g0001.1 **Mass:** 46038 **Score:** 39 **Matches:** 1(1) **Sequences:** 1(1)
NbS00017585g0001.1 protein AED:0.09 eAED:0.09 QI:0|0.66|0.5|1|1|1|4|0|417; (*GB) gi|342837524|dbj|BAK57343.1| (e_value=0.0) actin1 [Morella rubra];; (*SWP) sp|P53492|ACT7_ARATH (e_value=0.0) Actin-
NbS00018893g0009.1 **Mass:** 48966 **Score:** 39 **Matches:** 1(1) **Sequences:** 1(1)
NbS00018893g0009.1 protein AED:0.06 eAED:0.06 QI:40|0.75|0.8|1|0.75|0.6|5|49|437; (*GB) gi|333595899|gb|AEF58501.1| (e_value=0.0) actin [Platycodon grandiflorus];; (*SWP) sp|O81221|ACT_GOSHI (e_va
NbS00021375g0001.1 **Mass:** 41973 **Score:** 39 **Matches:** 1(1) **Sequences:** 1(1)
NbS00021375g0001.1 protein AED:0.06 eAED:0.06 QI:40|0.66|0.5|0.75|1|1|4|0|377; (*GB) gi|1168322|sp|P30168.2|ACT6_SOLTU (e_value=0.0) RecName: Full=Actin-71;; (*SWP) sp|P30168|ACT6_SOLTU (e_value=0.
NbS00038296g0004.1 **Mass:** 41959 **Score:** 39 **Matches:** 1(1) **Sequences:** 1(1)
NbS00038296g0004.1 protein AED:0.20 eAED:0.20 QI:16|1|1|1|1|1|4|411|377; (*GB) gi|356509003|ref|XP_003523242.1| (e_value=0.0) PREDICTED: actin-7-like [Glycine max];; (*SWP) sp|P53492|ACT7_ARATH (e_
NbC23512151g0001.1 **Mass:** 8878 **Score:** 39 **Matches:** 1(1) **Sequences:** 1(1)
NbC23512151g0001.1 protein AED:0.00 eAED:0.00 QI:0|-1|1|1|-1|0|1|0|78; (*GB) gi|380294407|gb|AFD50698.1| (e_value=2e-50) actin, partial [Plectranthus hilliardiae x Plectranthus saccatus];; (*SWP) s
NbC24141102g0002.1 **Mass:** 9523 **Score:** 39 **Matches:** 1(1) **Sequences:** 1(1)
NbC24141102g0002.1 protein AED:0.15 eAED:0.15 QI:0|-1|0|1|-1|1|1|0|83; (*GB) gi|255684836|gb|ACU27907.1| (e_value=2e-53) actin [Nicotiana tabacum];; (*SWP) sp|P93376|ACT6_TOBAC (e_value=3e-53) Act

---

124. NbS00033277g0006.1 **Score:** 38 **Matches:** 1(1) **Sequences:** 1(1) **emPAI:** 0.26
NbS00033277g0006.1 protein AED:0.56 eAED:0.56 QI:0|0|0|0.16|1|1|6|0|118

Query	Observed	Mr(expt)	Mr(calc)	ppm	Miss	Score	Expect	Rank	Unique	Peptide
10161	416.2501	830.4856	830.4861	-0.62	0	38	0.011	2	U	K.SETLILR.Q

---

125. NbS00001471g0009.1 **Mass:** 66057 **Score:** 38 **Matches:** 1(1) **Sequences:** 1(1) **emPAI:** 0.05
NbS00001471g0009.1 protein AED:0.21 eAED:0.22 QI:0|0.87|0.77|1|1|1|9|428|607; (*GB) gi|356555871|ref|XP_003546253.1| (e_value=0.0) PREDICTED: lysosomal beta glucosidase-like [Glycine max];; (*SWP)

Query	Observed	Mr(expt)	Mr(calc)	ppm	Miss	Score	Expect	Rank	Unique	Peptide
11560	500.7933	999.5720	999.5713	0.72	0	38	0.009	1	U	R.IGAATALEVR.A

Proteins matching the same set of peptides:
NbS00011140g0006.1 **Mass:** 76355 **Score:** 38 **Matches:** 1(1) **Sequences:** 1(1)
NbS00011140g0006.1 protein AED:0.16 eAED:0.17 QI:0|0.75|0.44|1|1|1|9|0|696; (*GB) gi|3582436|dbj|BAA33065.1| (e_value=0.0) beta-D-glucan exohydrolase [Nicotiana tabacum];; (*SWP) sp|Q23892|GLUA_DI(
NbS00011492g0006.1 **Mass:** 64535 **Score:** 38 **Matches:** 1(1) **Sequences:** 1(1)
NbS00011492g0006.1 protein AED:0.08 eAED:0.09 QI:0|0|0|1|1|1|2|0|589; (*GB) gi|3582436|dbj|BAA33065.1| (e_value=0.0) beta-D-glucan exohydrolase [Nicotiana tabacum];; (*SWP) sp|Q23892|GLUA_DICDI (e_
NbS00012136g0003.1 **Mass:** 68565 **Score:** 38 **Matches:** 1(1) **Sequences:** 1(1)
NbS00012136g0003.1 protein AED:0.20 eAED:0.20 QI:210|1|1|1|1|1|9|248|627; (*GB) gi|3582436|dbj|BAA33065.1| (e_value=0.0) beta-D-glucan exohydrolase [Nicotiana tabacum];; (*SWP) sp|Q23892|GLUA_DICDI
NbS00014718g0006.1 **Mass:** 77426 **Score:** 38 **Matches:** 1(1) **Sequences:** 1(1)
NbS00014718g0006.1 protein AED:0.13 eAED:0.13 QI:0|0.55|0.5|1|0.55|0.4|10|0|705; (*GB) gi|3582436|dbj|BAA33065.1| (e_value=0.0) beta-D-glucan exohydrolase [Nicotiana tabacum];; (*SWP) sp|Q23892|GLU
NbS00015529g0004.1 **Mass:** 72833 **Score:** 38 **Matches:** 1(1) **Sequences:** 1(1)
NbS00015529g0004.1 protein AED:0.05 eAED:0.05 QI:316|0.66|0.8|1|1|1|10|257|665; (*GB) gi|225436114|ref|XP_002278363.1| (e_value=0.0) PREDICTED: lysosomal beta glucosidase-like isoform 1 [Vitis vin:
NbS00024745g0008.1 **Mass:** 79490 **Score:** 38 **Matches:** 1(1) **Sequences:** 1(1)
NbS00024745g0008.1 protein AED:0.11 eAED:0.11 QI:182|0.77|0.7|1|1|1|10|339|722; (*GB) gi|356533037|ref|XP_003535075.1| (e_value=0.0) PREDICTED: lysosomal beta glucosidase-like [Glycine max];; (*SWI
NbS00033441g0004.1 **Mass:** 84959 **Score:** 38 **Matches:** 1(1) **Sequences:** 1(1)
NbS00033441g0004.1 protein AED:0.22 eAED:0.23 QI:0|0.63|0.5|0.91|0.90|0.83|12|0|754; (*GB) gi|255565893|ref|XP_002523935.1| (e_value=0.0) hydrolase, hydrolyzing O-glycosyl compounds, putative [Ric:
NbS00058677g0004.1 **Mass:** 70975 **Score:** 38 **Matches:** 1(1) **Sequences:** 1(1)
NbS00058677g0004.1 protein AED:0.06 eAED:0.06 QI:138|0.88|0.9|1|0.77|0.8|10|177|645; (*GB) gi|3582436|dbj|BAA33065.1| (e_value=0.0) beta-D-glucan exohydrolase [Nicotiana tabacum];; (*SWP) sp|Q2389
NbC25936038g0001.1 **Mass:** 18915 **Score:** 38 **Matches:** 1(1) **Sequences:** 1(1)
NbC25936038g0001.1 protein ; (*GB) gi|255565897|ref|XP_002523937.1| (e_value=8e-76) hydrolase, hydrolyzing O-glycosyl compounds, putative [Ricinus communis];; (*TAIR) AT5G04885.1 (e_value=6e-68) |
NbC26143923g0005.1 **Mass:** 60960 **Score:** 38 **Matches:** 1(1) **Sequences:** 1(1)
NbC26143923g0005.1 protein AED:0.16 eAED:0.19 QI:0|0.77|0.7|1|0.77|0.8|10|555|557; (*GB) gi|3582436|dbj|BAA33065.1| (e_value=0.0) beta-D-glucan exohydrolase [Nicotiana tabacum];; (*SWP) sp|Q56078|I

---

126. NbS00013115g0003.1 **Mass:** 17358 **Score:** 38 **Matches:** 1(1) **Sequences:** 1(1) **emPAI:** 0.20
NbS00013115g0003.1 protein AED:0.19 eAED:0.19 QI:3|0.33|0.5|1|1|1|4|273|152; (*GB) gi|357125783|ref|XP_003564569.1| (e_value=1e-85) PREDICTED: 40S ribosomal protein S15a-1-like [Brachypodium distac

Query	Observed	Mr(expt)	Mr(calc)	ppm	Miss	Score	Expect	Rank	Unique	Peptide
7454	479.7800	957.5454	957.5495	-4.21	0	38	0.012	1	U	R.VSVLNDALK.S

Proteins matching the same set of peptides:
NbS00016589g0109.1 **Mass:** 14824 **Score:** 38 **Matches:** 1(1) **Sequences:** 1(1)
NbS00016589g0109.1 protein AED:0.10 eAED:0.10 QI:160|1|1|1|1|1|3|248|130
NbS00022778g0011.1 **Mass:** 14838 **Score:** 38 **Matches:** 1(1) **Sequences:** 1(1)
NbS00022778g0011.1 protein AED:0.22 eAED:0.22 QI:193|1|0.5|1|1|1|4|316|130; (*GB) gi|357125783|ref|XP_003564569.1| (e_value=3e-91) PREDICTED: 40S ribosomal protein S15a-1-like [Brachypodium distac
NbS00028575g0006.1 **Mass:** 14960 **Score:** 38 **Matches:** 1(1) **Sequences:** 1(1)
NbS00028575g0006.1 protein AED:0.49 eAED:0.66 QI:0|0|0|0.5|1|1|6|0|133; (*GB) gi|225458489|ref|XP_002284120.1| (e_value=3e-19) PREDICTED: probable thylakoidal processing peptidase 2, chloroplastic
NbS00031449g0003.1 **Mass:** 14824 **Score:** 38 **Matches:** 1(1) **Sequences:** 1(1)
NbS00031449g0003.1 protein AED:0.10 eAED:0.10 QI:160|1|1|1|1|1|3|248|130; (*SWP) sp|P42798|R15A1_ARATH (e_value=3e-92) 40S ribosomal protein S15a-1 OS=Arabidopsis thaliana GN=RPS15AA PE=2 SV=2;; (
NbS00031559g0011.1 **Mass:** 16073 **Score:** 38 **Matches:** 1(1) **Sequences:** 1(1)
NbS00031559g0011.1 protein AED:0.16 eAED:0.17 QI:0|0.5|0.66|1|0.5|0.66|3|430|141; (*GB) gi|357125783|ref|XP_003564569.1| (e_value=2e-87) PREDICTED: 40S ribosomal protein S15a-1-like [Brachypodium (

---

127. NbS00041069g0013.1 **Mass:** 218166 **Score:** 38 **Matches:** 1(1) **Sequences:** 1(1) **emPAI:** 0.01
NbS00041069g0013.1 protein AED:0.01 eAED:0.02 QI:0|0.5|0.2|1|0.75|0.6|5|0|1975; (*GB) gi|296087607|emb|CBI34863.3| (e_value=0.0) unnamed protein product [Vitis vinifera];; (*SWP) sp|Q5VU65|P210L_HU

Query	Observed	Mr(expt)	Mr(calc)	ppm	Miss	Score	Expect	Rank	Unique	Peptide
12245	543.8110	1085.6074	1085.6081	-0.61	0	38	0.01	1	U	K.LDLDGLATLR.V

Proteins matching the same set of peptides:
NbS00006591g0002.1 **Mass:** 213952 **Score:** 38 **Matches:** 1(1) **Sequences:** 1(1)
NbS00006591g0002.1 protein AED:0.01 eAED:0.01 QI:0|0.66|0.25|1|1|1|4|0|1936; (*GB) gi|296087607|emb|CBI34863.3| (e_value=0.0) unnamed protein product [Vitis vinifera];; (*SWP) sp|P11654|PO210_RAT

---

128. NbS00030095g0005.1 **Mass:** 64038 **Score:** 38 **Matches:** 1(1) **Sequences:** 1(1) **emPAI:** 0.05
NbS00030095g0005.1 protein AED:0.14 eAED:0.14 QI:300|0.85|0.75|1|1|1|8|368|607; (*GB) gi|225423849|ref|XP_002281060.1| (e_value=0.0) PREDICTED: uncharacterized protein LOC100242198 [Vitis vinifera]

Query	Observed	Mr(expt)	Mr(calc)	ppm	Miss	Score	Expect	Rank	Unique	Peptide
15116	707.3557	1412.6968	1412.6936	2.23	1	38	0.0098	1	U	R.KIDVETFGDFSR.Y

129. NbS00008510g0008.1 **Mass:** 222396 **Score:** 38 **Matches:** 2(2) **Sequences:** 2(2) **emPAI:** 0.03
NbS00008510g0008.1 protein AED:0.13 eAED:0.13 QI:0|0.89|0.86|0.98|0.93|0.94|50|0|2033; (*GB) gi|296085156|emb|CBI28651.3| (e_value=0.0) unnamed protein product [Vitis vinifera];; (*SWP) sp|Q92616|(

Query	Observed	Mr(expt)	Mr(calc)	ppm	Miss	Score	Expect	Rank	Unique	Peptide
10408	435.7738	869.5331	869.5334	-0.43	0	32	0.02	1	U	R.AIIEVLGR.E
16269	777.9279	1553.8412	1553.8413	-0.10	0	33	0.022	1	U	K.LLDVLNTPSEAVQR.A

130. NbS00014998g0013.1 **Mass:** 62133 **Score:** 38 **Matches:** 1(1) **Sequences:** 1(1) **emPAI:** 0.05
NbS00014998g0013.1 protein AED:0.33 eAED:0.33 QI:0|0.83|0.57|1|0.66|0.57|7|0|574; (*GB) gi|359493612|ref|XP_003634635.1| (e_value=0.0) PREDICTED: uncharacterized protein LOC100264164 [Vitis vinife

Query	Observed	Mr(expt)	Mr(calc)	ppm	Miss	Score	Expect	Rank	Unique	Peptide
19681	717.3425	2149.0056	2149.0076	-0.93	0	38	0.0079	1	U	K.LFNAIDENGDGYLSHSELR.A

Proteins matching the same set of peptides:
NbS00024319g0014.1 **Mass:** 42563 **Score:** 38 **Matches:** 1(1) **Sequences:** 1(1)
NbS00024319g0014.1 protein AED:0.14 eAED:0.14 QI:0|1|0.8|1|0.5|0.6|5|0|391; (*GB) gi|359493612|ref|XP_003634635.1| (e_value=3e-145) PREDICTED: uncharacterized protein LOC100264164 [Vitis vinifera];

131. NbS00027609g0007.1 **Mass:** 67177 **Score:** 38 **Matches:** 1(1) **Sequences:** 1(1) **emPAI:** 0.05
NbS00027609g0007.1 protein AED:0.23 eAED:0.24 QI:0|0.88|0.84|1|0.94|0.84|19|0|601; (*GB) gi|316980592|dbj|BAJ51944.1| (e_value=0.0) NADP-dependent malic enzyme [Nicotiana benthamiana];; (*SWP) sp|I

Query	Observed	Mr(expt)	Mr(calc)	ppm	Miss	Score	Expect	Rank	Unique	Peptide
14307	658.3670	1314.7195	1314.7143	3.89	0	38	0.011	1	U	K.SIQVIVVTDGER.I

Proteins matching the same set of peptides:
NbS00028220g0110.1 **Mass:** 65802 **Score:** 38 **Matches:** 1(1) **Sequences:** 1(1)
NbS00028220g0110.1 protein AED:0.18 eAED:0.18 QI:364|1|1|1|0.94|0.94|19|264|591

132. NbS00012784g0015.1 **Mass:** 51219 **Score:** 38 **Matches:** 2(1) **Sequences:** 2(1) **emPAI:** 0.13
NbS00012784g0015.1 protein AED:0.24 eAED:0.24 QI:310|0.71|0.75|0.87|0.85|0.87|8|0|437; (*GB) gi|2459684|gb|AAB71764.1| (e_value=0.0) catalase 1 [Nicotiana tabacum];; (*SWP) sp|P49315|CATA1_NICPL (e

Query	Observed	Mr(expt)	Mr(calc)	ppm	Miss	Score	Expect	Rank	Unique	Peptide
11695	509.2643	1016.5140	1016.5138	0.16	0	31	0.07	1	U	K.SLLEEEAAR.I
19197	992.9990	1983.9835	1983.9843	-0.41	0	38	0.0094	1		R.EGNFDLVGNNFPVFFIR.D

133. NbS00015187g0009.1 **Mass:** 56801 **Score:** 38 **Matches:** 1(1) **Sequences:** 1(1) **emPAI:** 0.06
NbS00015187g0009.1 protein AED:0.20 eAED:0.20 QI:141|0.94|0.94|1|0.72|0.84|19|64|510; (*GB) gi|356504541|ref|XP_003521054.1| (e_value=0.0) PREDICTED: actin-related protein 4-like [Glycine max];; (

Query	Observed	Mr(expt)	Mr(calc)	ppm	Miss	Score	Expect	Rank	Unique	Peptide
20554	853.4430	2557.3072	2557.3024	1.84	0	38	0.0071	1	U	R.ATSLVVDSGGGSTTVAPVHDGYVLQK.A

134. NbS00013071g0001.1 **Mass:** 39307 **Score:** 37 **Matches:** 1(1) **Sequences:** 1(1) **emPAI:** 0.08
NbS00013071g0001.1 protein AED:0.14 eAED:0.14 QI:0|0.66|0.25|1|1|1|4|0|360; (*SWP) sp|Q9XIV8|PERN1_TOBAC (e_value=0.0) Peroxidase N1 OS=Nicotiana tabacum GN=poxN1 PE=1 SV=1;; (*TAIR) AT5G64120.1 (e

Query	Observed	Mr(expt)	Mr(calc)	ppm	Miss	Score	Expect	Rank	Unique	Peptide
12268	545.2988	1088.5831	1088.5826	0.48	0	37	0.016	1	U	R.AESIVQSTVR.A

Proteins matching the same set of peptides:
NbS00037769g0004.1 **Mass:** 36161 **Score:** 37 **Matches:** 1(1) **Sequences:** 1(1)
NbS00037769g0004.1 protein AED:0.16 eAED:0.16 QI:0|1|0.33|1|1|1|3|0|330; (*GB) gi|75315324|sp|Q9XIV8.1|PERN1_TOBAC (e_value=0.0) RecName: Full=Peroxidase N1; AltName: Full=Peroxidase B2; AltName: I

135. NbS00020307g0016.1 **Mass:** 82868 **Score:** 37 **Matches:** 1(1) **Sequences:** 1(1) **emPAI:** 0.04
NbS00020307g0016.1 protein AED:0.13 eAED:0.13 QI:0|0.33|0.25|0.75|0.66|0.25|4|0|741; (*GB) gi|75249421|sp|Q93YF5.1|SUVH1_TOBAC (e_value=0.0) RecName: Full=Histone-lysine N-methyltransferase, H3 lys

Query	Observed	Mr(expt)	Mr(calc)	ppm	Miss	Score	Expect	Rank	Unique	Peptide
18489	935.9238	1869.8331	1869.8381	-2.67	0	37	0.007	1	U	R.VGELGNEGDDYIFDATR.I

136. NbS00001594g0015.1 **Mass:** 62334 **Score:** 37 **Matches:** 1(1) **Sequences:** 1(1) **emPAI:** 0.05
NbS00001594g0015.1 protein AED:0.04 eAED:0.04 QI:64|0.5|0.6|1|0.75|0.6|5|0|553; (*GB) gi|268619136|gb|ACZ13344.1| (e_value=0.0) tubulin alpha chain [Bursaphelenchus xylophilus];; (*SWP) sp|Q9ZRR5|T

Query	Observed	Mr(expt)	Mr(calc)	ppm	Miss	Score	Expect	Rank	Unique	Peptide
17590	851.4575	1700.9004	1700.8985	1.09	0	37	0.012	1	U	R.AVFVDLEPTVIDEVR.T

Proteins matching the same set of peptides:

NbS00002652g0220.1 **Mass:** 60332 **Score:** 37 **Matches:** 1(1) **Sequences:** 1(1)
NbS00002652g0220.1 protein AED:0.10 eAED:0.10 QI:0|0|0|0.6|1|1|5|0|537
NbS00003471g0210.1 **Mass:** 46317 **Score:** 37 **Matches:** 1(1) **Sequences:** 1(1)
NbS00003471g0210.1 protein AED:0.09 eAED:0.09 QI:260|1|0.75|1|0.66|0.5|4|0|414
NbS00006458g0003.1 **Mass:** 55859 **Score:** 37 **Matches:** 1(1) **Sequences:** 1(1)
NbS00006458g0003.1 protein AED:0.07 eAED:0.07 QI:12|0.33|0.25|1|0.66|0.5|4|0|500; (*GB) gi|17402471|emb|CAD13178.1| (e_value=0.0) alpha-tubulin [Nicotiana tabacum];; (*SWP) sp|P33629|TBA_PRUDU (e_v
NbS00008055g0007.1 **Mass:** 49267 **Score:** 37 **Matches:** 1(1) **Sequences:** 1(1)
NbS00008055g0007.1 protein AED:0.07 eAED:0.07 QI:0|0.5|0.4|1|0.5|0.4|5|0|441; (*GB) gi|134035502|gb|ABO47739.1| (e_value=0.0) alpha-tubulin [Gossypium hirsutum];; (*SWP) sp|P29510|TBA2_ARATH (e_va
NbS00026267g0008.1 **Mass:** 52560 **Score:** 37 **Matches:** 1(1) **Sequences:** 1(1)
NbS00026267g0008.1 protein AED:0.09 eAED:0.09 QI:0|0.2|0.16|1|0.6|0.5|6|0|471; (*GB) gi|17402471|emb|CAD13178.1| (e_value=0.0) alpha-tubulin [Nicotiana tabacum];; (*SWP) sp|P33629|TBA_PRUDU (e_valu
NbS00031544g0010.1 **Mass:** 50360 **Score:** 37 **Matches:** 1(1) **Sequences:** 1(1)
NbS00031544g0010.1 protein AED:0.10 eAED:0.10 QI:4|1|1|1|1|1|4|217|450; (*GB) gi|386870485|gb|AFJ42573.1| (e_value=0.0) alpha-tubulin [Sesamum indicum];; (*SWP) sp|P33629|TBA_PRUDU (e_value=0.0) Tu
NbS00031817g0012.1 **Mass:** 31630 **Score:** 37 **Matches:** 1(1) **Sequences:** 1(1)
NbS00031817g0012.1 protein AED:0.21 eAED:0.22 QI:3|0.66|0.75|1|0.66|0.75|4|0|284; (*GB) gi|390341332|ref|XP_784342.3| (e_value=3e-149) PREDICTED: tubulin alpha-1 chain-like [Strongylocentrotus purp
gi|37492|emb|CAA25855.1| **Mass:** 50810 **Score:** 37 **Matches:** 1(1) **Sequences:** 1(1)
gi|37492|emb|CAA25855.1| alpha-tubulin [Homo sapiens]
NbS00038051g0004.1 **Mass:** 55743 **Score:** 37 **Matches:** 1(1) **Sequences:** 1(1)
NbS00038051g0004.1 protein AED:0.04 eAED:0.04 QI:0|0|0|1|1|1|3|0|493; (*GB) gi|17402469|emb|CAD13177.1| (e_value=0.0) alpha-tubulin [Nicotiana tabacum];; (*SWP) sp|P33629|TBA_PRUDU (e_value=0.0) Tu
NbS00058547g0002.1 **Mass:** 58434 **Score:** 37 **Matches:** 1(1) **Sequences:** 1(1)
NbS00058547g0002.1 protein AED:0.08 eAED:0.08 QI:4|0.75|0.4|1|1|1|5|0|526; (*GB) gi|348515729|ref|XP_003445392.1| (e_value=0.0) PREDICTED: tubulin alpha chain-like [Oreochromis niloticus];; (*SWP)

---

137. NbS00015419g0005.1 **Mass:** 31000 **Score:** 37 **Matches:** 1(1) **Sequences:** 1(1) **emPAI:** 0.11
NbS00015419g0005.1 protein AED:0.32 eAED:0.32 QI:0|1|1|1|0.75|0.6|5|174|287; (*GB) gi|2114050|dbj|BAA20076.1| (e_value=0.0) water channel protein [Nicotiana excelsior];; (*SWP) sp|Q08451|PIP1_SOLLC

Query	Observed	Mr(expt)	Mr(calc)	ppm	Miss	Score	Expect	Rank	Unique	Peptide
14716	680.8653	1359.7160	1359.7147	1.00	0	37	0.015	2	U	R.LGGGANVVQPGYTK.G

Proteins matching the same set of peptides:

NbS00043512g0006.1 **Mass:** 29648 **Score:** 37 **Matches:** 1(1) **Sequences:** 1(1)
NbS00043512g0006.1 protein AED:0.15 eAED:0.15 QI:166|0.8|0.83|1|0.6|0.5|6|221|275; (*GB) gi|2114050|dbj|BAA20076.1| (e_value=0.0) water channel protein [Nicotiana excelsior];; (*SWP) sp|Q08451|PIP1
NbC25434411g0004.1 **Mass:** 14362 **Score:** 37 **Matches:** 1(1) **Sequences:** 1(1)
NbC25434411g0004.1 protein AED:0.23 eAED:0.23 QI:0|1|0|1|1|1|2|0|136; (*GB) gi|2114050|dbj|BAA20076.1| (e_value=4e-93) water channel protein [Nicotiana excelsior];; (*SWP) sp|Q08451|PIP1_SOLLC (e_v

---

138. NbS00061216g0001.1 **Mass:** 20473 **Score:** 37 **Matches:** 1(1) **Sequences:** 1(1) **emPAI:** 0.16
NbS00061216g0001.1 protein AED:0.17 eAED:0.17 QI:0|-1|0|1|-1|1|1|0|179; (*SWP) sp|P11670|PRB1_TOBAC (e_value=3e-109) Basic form of pathogenesis-related protein 1 OS=Nicotiana tabacum PE=3 SV=1;; (*

Query	Observed	Mr(expt)	Mr(calc)	ppm	Miss	Score	Expect	Rank	Unique	Peptide
14638	676.8399	1351.6653	1351.6633	1.47	0	37	0.016	1	U	R.VAAFAQNYANQR.A

---

139. NbS00014940g0015.1 **Mass:** 159676 **Score:** 37 **Matches:** 1(1) **Sequences:** 1(1) **emPAI:** 0.02
NbS00014940g0015.1 protein AED:0.19 eAED:0.20 QI:221|0.95|0.77|1|0.90|0.95|22|289|1422; (*GB) gi|296086345|emb|CBI31934.3| (e_value=6e-179) unnamed protein product [Vitis vinifera];; (*TAIR) AT2G0

Query	Observed	Mr(expt)	Mr(calc)	ppm	Miss	Score	Expect	Rank	Unique	Peptide
12750	575.2729	1148.5312	1148.5309	0.27	0	37	0.015	1	U	K.NSLEELSSDR.N

Proteins matching the same set of peptides:

NbS00014941g0006.1 **Mass:** 159674 **Score:** 37 **Matches:** 1(1) **Sequences:** 1(1)
NbS00014941g0006.1 protein AED:0.16 eAED:0.17 QI:239|0.82|0.70|1|0.73|0.75|24|320|1403; (*GB) gi|296086345|emb|CBI31934.3| (e_value=5e-161) unnamed protein product [Vitis vinifera];; (*TAIR) AT2G0

---

140. NbS00011860g0002.1 **Mass:** 87858 **Score:** 36 **Matches:** 1(1) **Sequences:** 1(1) **emPAI:** 0.04
NbS00011860g0002.1 protein AED:0.14 eAED:0.14 QI:0|0.83|0.76|0.84|1|1|13|0|788; (*GB) gi|255561268|ref|XP_002521645.1| (e_value=0.0) arsenite-resistance protein, putative [Ricinus communis];; (*SWI

Query	Observed	Mr(expt)	Mr(calc)	ppm	Miss	Score	Expect	Rank	Unique	Peptide
14984	698.8995	1395.7845	1395.7834	0.79	0	36	0.0093	1	U	R.VQIDVGQAQALVR.K

---

141. NbS00048220g0003.1 **Score:** 36 **Matches:** 1(1) **Sequences:** 1(1) **emPAI:** 0.04
NbS00048220g0003.1 protein AED:0.08 eAED:0.08 QI:242|1|0.92|1|0.91|0.92|13|593|685; (*GB) gi|359483452|ref|XP_002270330.2| (e_value=0.0) PREDICTED: E3 ubiquitin-protein ligase COP1-like [Vitis vin:

Query	Observed	Mr(expt)	Mr(calc)	ppm	Miss	Score	Expect	Rank	Unique	Peptide
11634	505.8337	1009.6529	1009.6536	-0.69	0	36	0.0016	2	U	R.LIGALVPTVK.S

142. NbS00002188g0022.1 **Mass:** 25109 **Score:** 36 **Matches:** 1(1) **Sequences:** 1(1) **emPAI:** 0.13
NbS00002188g0022.1 protein AED:0.32 eAED:0.32 QI:0|1|0.5|1|1|1|2|0|227; (*GB) gi|464621|sp|P34091.1|RL6_MESCR (e_value=7e-104) RecName: Full=60S ribosomal protein L6; AltName: Full=YL16-like;; (*SW

Query	Observed	Mr(expt)	Mr(calc)	ppm	Miss	Score	Expect	Rank	Unique	Peptide
12518	**559.3347**	**1116.6549**	**1116.6543**	**0.52**	**0**	**36**	**0.011**	**1**	**U**	**K.SGLLLVSGPFK.L**

Proteins matching the same set of peptides:
NbS00031098g0006.1 **Mass:** 25493 **Score:** 36 **Matches:** 1(1) **Sequences:** 1(1)
NbS00031098g0006.1 protein AED:0.18 eAED:0.18 QI:96|1|1|1|1|1|2|68|230; (*GB) gi|464621|sp|P34091.1|RL6_MESCR (e_value=5e-104) RecName: Full=60S ribosomal protein L6; AltName: Full=YL16-like;; (*SW

143. NbS00015227g0002.1 **Mass:** 18882 **Score:** 36 **Matches:** 1(1) **Sequences:** 1(1) **emPAI:** 0.18
NbS00015227g0002.1 protein AED:0.14 eAED:0.16 QI:0|-1|0|1|-1|1|1|0|171; (*GB) gi|237783971|gb|ACR19782.1| (e_value=5e-91) ATP synthase CF1 beta subunit [Nolana galapagensis];; (*SWP) sp|P26530|ATPB

Query	Observed	Mr(expt)	Mr(calc)	ppm	Miss	Score	Expect	Rank	Unique	Peptide
19437	**1031.0223**	**2060.0301**	**2060.0248**	**2.57**	**0**	**36**	**0.014**	**1**	**U**	**K.GIYPAVDPLDSTSTMLQPR.I**

144. NbS00027670g0006.1 **Mass:** 59238 **Score:** 36 **Matches:** 1(1) **Sequences:** 1(1) **emPAI:** 0.06
NbS00027670g0006.1 protein AED:0.24 eAED:0.24 QI:614|0.93|0.93|1|1|1|16|163|536; (*SWP) sp|P50433|GLYM_SOLTU (e_value=0.0) Serine hydroxymethyltransferase, mitochondrial OS=Solanum tuberosum PE=2 S

Query	Observed	Mr(expt)	Mr(calc)	ppm	Miss	Score	Expect	Rank	Unique	Peptide
18051	**894.4227**	**1786.8308**	**1786.8261**	**2.62**	**0**	**36**	**0.012**	**1**	**U**	**R.LNESTGYIDYDQLEK.S**

Proteins matching the same set of peptides:
NbS00042478g0005.1 **Mass:** 57495 **Score:** 36 **Matches:** 1(1) **Sequences:** 1(1)
NbS00042478g0005.1 protein AED:0.25 eAED:0.25 QI:312|1|1|1|0.93|0.93|16|436|518; (*GB) gi|1707998|sp|P50433.1|GLYM_SOLTU (e_value=0.0) RecName: Full=Serine hydroxymethyltransferase, mitochondrial;

145. NbS00001709g0013.1 **Mass:** 35836 **Score:** 35 **Matches:** 1(1) **Sequences:** 1(1) **emPAI:** 0.09
NbS00001709g0013.1 protein AED:0.15 eAED:0.15 QI:227|1|1|1|0.85|0.75|8|809|341; (*GB) gi|21388546|emb|CAD33241.1| (e_value=0.0) putative mitochondrial NAD-dependent malate dehydrogenase [Solanum tu

Query	Observed	Mr(expt)	Mr(calc)	ppm	Miss	Score	Expect	Rank	Unique	Peptide
14340	**659.8547**	**1317.6949**	**1317.6929**	**1.57**	**0**	**35**	**0.021**	**1**	**U**	**R.DDLFNINAGIVK.S**

Proteins matching the same set of peptides:
NbS00011011g0027.1 **Mass:** 38126 **Score:** 35 **Matches:** 1(1) **Sequences:** 1(1)
NbS00011011g0027.1 protein AED:0.09 eAED:0.09 QI:401|0.75|0.88|1|0.5|0.55|9|581|363; (*GB) gi|21388546|emb|CAD33241.1| (e_value=0.0) putative mitochondrial NAD-dependent malate dehydrogenase [Solan
NbS00015957g0014.1 **Mass:** 46103 **Score:** 35 **Matches:** 1(1) **Sequences:** 1(1)
NbS00015957g0014.1 protein AED:0.11 eAED:0.11 QI:0|0.71|0.62|1|0.85|0.87|8|550|434; (*SWP) sp|P17783|MDHM_CITLA (e_value=0.0) Malate dehydrogenase, mitochondrial OS=Citrullus lanatus GN=MMDH PE=1 S
NbS00022525g0113.1 **Mass:** 35306 **Score:** 35 **Matches:** 1(1) **Sequences:** 1(1)
NbS00022525g0113.1 protein AED:0.18 eAED:0.18 QI:189|0.87|0.77|1|0.75|0.66|9|554|335

146. NbS00019391g0012.1 **Mass:** 41160 **Score:** 35 **Matches:** 1(1) **Sequences:** 1(1) **emPAI:** 0.08
NbS00019391g0012.1 protein AED:0.09 eAED:0.09 QI:72|0.88|0.9|1|0.88|1|10|207|369; (*TAIR) AT4G34660.1 (e_value=0.0) | Symbols: | SH3 domain-containing protein | chr4:16545595-16548294 RVERSE LENGTH

Query	Observed	Mr(expt)	Mr(calc)	ppm	Miss	Score	Expect	Rank	Unique	Peptide
8207	555.3000	1108.5854	1108.5764	8.13	0	35	0.041	2	U	R.GVEGYIISGSK.Q

Proteins matching the same set of peptides:
NbS00031419g0012.1 **Mass:** 36635 **Score:** 35 **Matches:** 1(1) **Sequences:** 1(1)
NbS00031419g0012.1 protein AED:0.19 eAED:0.19 QI:79|0.66|0.6|1|0.77|0.8|10|0|329; (*GB) gi|255543841|ref|XP_002512983.1| (e_value=4e-160) clathrin binding protein, putative [Ricinus communis];; (*T

147. NbS00031017g0008.1 **Score:** 35 **Matches:** 1(1) **Sequences:** 1(1) **emPAI:** 0.15
NbS00031017g0008.1 protein AED:0.42 eAED:0.42 QI:0|0|0|1|0|0|2|0|197; (*GB) gi|190607593|gb|ACE79459.1| (e_value=4e-63) NBS-coding resistance gene analog [Nicotiana tabacum];; (*SWP) sp|Q6L403|R1B1

Query	Observed	Mr(expt)	Mr(calc)	ppm	Miss	Score	Expect	Rank	Unique	Peptide
11792	515.7798	1029.5451	1029.5091	35.0	0	35	0.026	2	U	R.ADELADQLR.R

148. NbS00003075g0012.1 **Mass:** 177053 **Score:** 35 **Matches:** 1(1) **Sequences:** 1(1) **emPAI:** 0.02
NbS00003075g0012.1 protein AED:0.17 eAED:0.17 QI:419|0.94|0.88|1|0.88|0.91|36|397|1540; (*GB) gi|116047943|gb|ABJ53197.1| (e_value=0.0) myosin XI-2 [Nicotiana benthamiana];; (*SWP) sp|P54697|MYOJ_I

Query	Observed	Mr(expt)	Mr(calc)	ppm	Miss	Score	Expect	Rank	Unique	Peptide
11498	497.2716	992.5286	992.5212	7.45	1	35	0.026	1	U	K.ETKEMLVK.E

Proteins matching the same set of peptides:
NbS00009118g0004.1 **Mass:** 175783 **Score:** 35 **Matches:** 1(1) **Sequences:** 1(1)
NbS00009118g0004.1 protein AED:0.21 eAED:0.22 QI:421|0.86|0.86|1|0.78|0.78|38|0|1530; (*GB) gi|116047943|gb|ABJ53197.1| (e_value=0.0) myosin XI-2 [Nicotiana benthamiana];; (*SWP) sp|P54697|MYOJ_DI(

149. NbS00002044g0008.1 **Mass:** 23361 **Score:** 35 **Matches:** 1(1) **Sequences:** 1(1) **emPAI:** 0.14
NbS00002044g0008.1 protein AED:0.19 eAED:0.19 QI:375|1|1|1|0.5|0.57|7|290|211; (*GB) gi|356555789|ref|XP_003546212.1| (e_value=2e-134) PREDICTED: ras-related protein RABB1c-like [Glycine max];; (*S

Query	Observed	Mr(expt)	Mr(calc)	ppm	Miss	Score	Expect	Rank	Unique	Peptide
16098	**775.8851**	**1549.7556**	**1549.7525**	**1.99**	**0**	**35**	**0.023**	**1**	**U**	**K.LQIWDTAGQESFR.S**

Proteins matching the same set of peptides:
NbS00004361g0010.1 **Mass:** 23353 **Score:** 35 **Matches:** 1(1) **Sequences:** 1(1)
NbS00004361g0010.1 protein AED:0.20 eAED:0.20 QI:323|1|1|1|1|1|6|340|211; (*GB) gi|16755592|gb|AAL28022.1| (e_value=2e-136) small GTPase Rab2 [Nicotiana tabacum];; (*SWP) sp|P92963|RAB1C_ARATH (e_v
NbS00010402g0006.1 **Mass:** 23375 **Score:** 35 **Matches:** 1(1) **Sequences:** 1(1)
NbS00010402g0006.1 protein AED:0.06 eAED:0.06 QI:210|1|1|1|0.66|0.57|7|290|211; (*GB) gi|356555789|ref|XP_003546212.1| (e_value=1e-132) PREDICTED: ras-related protein RABB1c-like [Glycine max];; (*
NbS00013323g0007.1 **Mass:** 23053 **Score:** 35 **Matches:** 1(1) **Sequences:** 1(1)
NbS00013323g0007.1 protein AED:0.30 eAED:0.30 QI:23|0.8|0.83|1|0.8|0.83|6|476|207; (*GB) gi|255570175|ref|XP_002526048.1| (e_value=2e-144) protein with unknown function [Ricinus communis];; (*SWP)
NbS00021088g0008.1 **Mass:** 23349 **Score:** 35 **Matches:** 1(1) **Sequences:** 1(1)
NbS00021088g0008.1 protein AED:0.04 eAED:0.04 QI:316|1|1|1|1|1|6|203|211; (*GB) gi|16755592|gb|AAL28022.1| (e_value=3e-136) small GTPase Rab2 [Nicotiana tabacum];; (*SWP) sp|P92963|RAB1C_ARATH (e_v
NbS00054942g0003.1 **Mass:** 23411 **Score:** 35 **Matches:** 1(1) **Sequences:** 1(1)
NbS00054942g0003.1 protein AED:0.31 eAED:0.31 QI:254|1|1|1|1|1|6|256|211; (*GB) gi|16755592|gb|AAL28022.1| (e_value=2e-142) small GTPase Rab2 [Nicotiana tabacum];; (*SWP) sp|P92963|RAB1C_ARATH (e_v

150. NbS00000710g0004.1 **Mass:** 54003 **Score:** 35 **Matches:** 1(1) **Sequences:** 1(1) **emPAI:** 0.06
NbS00000710g0004.1 protein AED:0.10 eAED:0.12 QI:195|0.77|0.7|1|0.77|0.5|10|0|501; (*SWP) sp|O94260|G3BP_SCHPO (e_value=3e-21) Putative G3BP-like protein OS=Schizosaccharomyces pombe (strain 972 /

Query	Observed	Mr(expt)	Mr(calc)	ppm	Miss	Score	Expect	Rank	Unique	Peptide
13049	**591.8464**	**1181.6783**	**1181.6768**	**1.28**	**0**	**35**	**0.012**	**1**	**U**	**K.AIEASPVLIGGR.Q**

151. NbS00022054g0014.1 **Mass:** 73762 **Score:** 35 **Matches:** 1(1) **Sequences:** 1(1) **emPAI:** 0.04
NbS00022054g0014.1 protein AED:0.15 eAED:0.17 QI:48|1|0.71|1|1|1|7|0|661; (*GB) gi|225444019|ref|XP_002281594.1| (e_value=0.0) PREDICTED: cleavage and polyadenylation specificity factor CPSF30-like

Query	Observed	Mr(expt)	Mr(calc)	ppm	Miss	Score	Expect	Rank	Unique	Peptide
12603	**564.8216**	**1127.6286**	**1127.6299**	**-1.11**	**0**	**35**	**0.02**	**1**	**U**	**K.TAVVLPQGTSR.Y**

152. NbS00007600g0009.1 **Mass:** 106403 **Score:** 34 **Matches:** 1(1) **Sequences:** 1(1) **emPAI:** 0.03
NbS00007600g0009.1 protein AED:0.75 eAED:0.75 QI:0|0|0|0.04|0.83|0.88|25|0|918; (*SWP) sp|Q9M3G7|ATM_ARATH (e_value=0.0) Serine/threonine-protein kinase ATM OS=Arabidopsis thaliana GN=ATM PE=2 SV=1

Query	Observed	Mr(expt)	Mr(calc)	ppm	Miss	Score	Expect	Rank	Unique	Peptide
11739	**512.2764**	**1022.5382**	**1022.5470**	**-8.65**	**0**	**34**	**0.026**	**1**	**U**	**K.QMVEIYIK.L**

153. NbS00002523g0003.1 **Mass:** 78674 **Score:** 34 **Matches:** 1(1) **Sequences:** 1(1) **emPAI:** 0.04
NbS00002523g0003.1 protein AED:0.18 eAED:0.18 QI:0|-1|0|1|-1|1|1|0|704; (*GB) gi|75249421|sp|Q93YF5.1|SUVH1_TOBAC (e_value=0.0) RecName: Full=Histone-lysine N-methyltransferase, H3 lysine-9 specif:

Query	Observed	Mr(expt)	Mr(calc)	ppm	Miss	Score	Expect	Rank	Unique	Peptide
12735	**574.3058**	**1146.5970**	**1146.5961**	**0.82**	**0**	**34**	**0.034**	**1**	**U**	**K.IYIYDGLYK.I**

Proteins matching the same set of peptides:
NbS00004793g0007.1 **Mass:** 83339 **Score:** 34 **Matches:** 1(1) **Sequences:** 1(1)
NbS00004793g0007.1 protein AED:0.17 eAED:0.17 QI:0|-1|0|1|-1|1|1|0|749; (*SWP) sp|Q93YF5|SUVH1_TOBAC (e_value=0.0) Histone-lysine N-methyltransferase, H3 lysine-9 specific SUVH1 OS=Nicotiana tabacu

154. NbS00010454g0009.1 **Mass:** 35657 **Score:** 34 **Matches:** 1(1) **Sequences:** 1(1) **emPAI:** 0.09
NbS00010454g0009.1 protein AED:0.20 eAED:0.20 QI:0|1|0.5|1|1|1|2|0|333; (*SWP) sp|Q40459|PSBO_TOBAC (e_value=0.0) Oxygen-evolving enhancer protein 1, chloroplastic OS=Nicotiana tabacum GN=PSBO PE=2

Query	Observed	Mr(expt)	Mr(calc)	ppm	Miss	Score	Expect	Rank	Unique	Peptide
5038	425.7200	849.4254	849.4232	2.61	0	34	0.038	1	U	R.GSSFLDPK.G

Proteins matching the same set of peptides:
NbS00019818g0001.1 **Mass:** 35404 **Score:** 34 **Matches:** 1(1) **Sequences:** 1(1)
NbS00019818g0001.1 protein AED:0.28 eAED:0.28 QI:0|1|0.5|1|1|1|2|0|332; (*GB) gi|384038815|gb|AFH57998.1| (e_value=0.0) chloroplast PsbO2 precursor [Nicotiana benthamiana];; (*SWP) sp|Q40459|PSBO_T
NbS00027134g0004.1 **Mass:** 39762 **Score:** 34 **Matches:** 1(1) **Sequences:** 1(1)
NbS00027134g0004.1 protein AED:0.02 eAED:0.02 QI:0|1|0.5|1|1|1|2|0|374; (*GB) gi|384038819|gb|AFH58000.1| (e_value=0.0) chloroplast PsbO4 precursor [Nicotiana benthamiana];; (*SWP) sp|Q40459|PSBO_T
NbS00032545g0003.1 **Mass:** 35462 **Score:** 34 **Matches:** 1(1) **Sequences:** 1(1)
NbS00032545g0003.1 protein AED:0.18 eAED:0.18 QI:0|1|0.5|1|1|1|2|0|332; (*GB) gi|384038813|gb|AFH57997.1| (e_value=0.0) chloroplast PsbO1 precursor [Nicotiana benthamiana];; (*SWP) sp|Q40459|PSBO_T
NbC23666742g0001.1 **Mass:** 9076 **Score:** 34 **Matches:** 1(1) **Sequences:** 1(1)
NbC23666742g0001.1 protein ; (*GB) gi|61697115|gb|AAX53163.1| (e_value=8e-52) chloroplast photosynthetic oxygen-evolving protein 33 kDa subunit [Nicotiana benthamiana];; (*SWP) sp|Q40459|PSBO_TOBAC

---

155. NbS00001075g0007.1 **Mass:** 62797 **Score:** 34 **Matches:** 1(1) **Sequences:** 1(1) **emPAI:** 0.05
NbS00001075g0007.1 protein AED:0.16 eAED:0.16 QI:0|0.54|0.41|0.91|1|1|12|133|554; (*GB) gi|20522008|dbj|BAB92011.1| (e_value=0.0) pleiotropic drug resistance like protein [Nicotiana tabacum];; (*SW

Query	Observed	Mr(expt)	Mr(calc)	ppm	Miss	Score	Expect	Rank	Unique	Peptide
13342	609.8239	1217.6332	1217.6326	0.47	0	34	0.032	1	U	R.VTTGEMLVGPSK.A

Proteins matching the same set of peptides:
NbS00019903g0005.1 **Mass:** 162134 **Score:** 34 **Matches:** 1(1) **Sequences:** 1(1)
NbS00019903g0005.1 protein AED:0.20 eAED:0.20 QI:0|1|1|1|1|1|20|174|1434; (*GB) gi|75326590|sp|Q76CU2.1|PDR1_TOBAC (e_value=0.0) RecName: Full=Pleiotropic drug resistance protein 1; AltName: Full=N
NbS00038421g0004.1 **Mass:** 170425 **Score:** 34 **Matches:** 1(1) **Sequences:** 1(1)
NbS00038421g0004.1 protein AED:0.16 eAED:0.16 QI:204|0.9|0.90|1|1|1|21|174|1502; (*GB) gi|75326590|sp|Q76CU2.1|PDR1_TOBAC (e_value=0.0) RecName: Full=Pleiotropic drug resistance protein 1; AltName:

---

156. NbS00010523g0001.1 **Mass:** 167184 **Score:** 34 **Matches:** 1(1) **Sequences:** 1(1) **emPAI:** 0.02
NbS00010523g0001.1 protein AED:0.13 eAED:0.14 QI:0|0.80|0.68|1|1|1|22|0|1481; (*GB) gi|75331994|sp|Q949G3.1|PDR1_NICPL (e_value=0.0) RecName: Full=Pleiotropic drug resistance protein 1; AltName: Fu

Query	Observed	Mr(expt)	Mr(calc)	ppm	Miss	Score	Expect	Rank	Unique	Peptide
13342	609.8239	1217.6332	1217.6326	0.47	0	34	0.032	1	U	R.VTTGEMIVGPSK.A

Proteins matching the same set of peptides:
NbS00038999g0004.1 **Mass:** 161442 **Score:** 34 **Matches:** 1(1) **Sequences:** 1(1)
NbS00038999g0004.1 protein AED:0.14 eAED:0.14 QI:0|0.94|0.95|1|0.84|0.9|20|140|1427; (*GB) gi|75331994|sp|Q949G3.1|PDR1_NICPL (e_value=0.0) RecName: Full=Pleiotropic drug resistance protein 1; AltN

---

157. NbS00004717g0103.1 **Mass:** 13622 **Score:** 34 **Matches:** 1(1) **Sequences:** 1(1) **emPAI:** 0.25
NbS00004717g0103.1 protein AED:0.03 eAED:0.03 QI:0|-1|0|1|-1|1|1|0|128

Query	Observed	Mr(expt)	Mr(calc)	ppm	Miss	Score	Expect	Rank	Unique	Peptide
10014	407.2628	812.5110	812.5120	-1.25	0	34	0.014	1	U	K.ANVLGIVK.L

Proteins matching the same set of peptides:
NbS00017897g0003.1 **Mass:** 13705 **Score:** 34 **Matches:** 1(1) **Sequences:** 1(1)
NbS00017897g0003.1 protein AED:0.00 eAED:0.00 QI:176|0|0.5|1|0|0.5|2|349|129; (*GB) gi|118488240|gb|ABK95939.1| (e_value=2e-36) unknown [Populus trichocarpa];; (*SWP) sp|P14009|14KD_DAUCA (e_value=

---

158. NbS00017222g0017.1 **Mass:** 108618 **Score:** 34 **Matches:** 1(1) **Sequences:** 1(1) **emPAI:** 0.03
NbS00017222g0017.1 protein AED:0.07 eAED:0.07 QI:314|0.66|0.57|1|0.16|0.14|7|435|963; (*GB) gi|255559969|ref|XP_002521003.1| (e_value=7e-93) hypothetical protein RCOM_0992860 [Ricinus communis];;

Query	Observed	Mr(expt)	Mr(calc)	ppm	Miss	Score	Expect	Rank	Unique	Peptide
11509	498.2434	994.4723	994.5083	-36.20	0	34	0.03	1	U	K.ADISALSYR.E

---

159. NbS00004226g0004.1 **Mass:** 14244 **Score:** 34 **Matches:** 1(1) **Sequences:** 1(1) **emPAI:** 0.24
NbS00004226g0004.1 protein ; (*GB) gi|94466657|emb|CAJ44457.1| (e_value=4e-18) ALY protein [Nicotiana benthamiana];; (*ITAG) Solyc10g086400.1.1 (e_value=3e-16) evidence_code:10F1H1E1IEG genomic_ref

Query	Observed	Mr(expt)	Mr(calc)	ppm	Miss	Score	Expect	Rank	Unique	Peptide
12828	580.3202	1158.6258	1158.6245	1.19	0	34	0.036	1	U	R.GAGGISGIETGIK.L

Proteins matching the same set of peptides:

NbS00025223g0004.1 **Mass:** 16135 **Score:** 34 **Matches:** 1(1) **Sequences:** 1(1)
NbS00025223g0004.1 protein ; (*GB) gi|51490663|emb|CAG26902.1| (e_value=4e-28) ALY protein [Nicotiana benthamiana];; (*ITAG) Solyc10g086400.1.1 (e_value=5e-19) evidence_code:10F1H1E1IEG genomic_ref

---

160. NbS00000361g0007.1 **Mass:** 62469 **Score:** 33 **Matches:** 1(1) **Sequences:** 1(1) **emPAI:** 0.05
NbS00000361g0007.1 protein AED:0.06 eAED:0.06 QI:0|0.83|0.85|1|0.83|0.71|7|414|546; (*GB) gi|350539281|ref|NP_001234129.1| (e_value=0.0) calnexin-like protein precursor [Solanum lycopersicum];; (*S

Query	Observed	Mr(expt)	Mr(calc)	ppm	Miss	Score	Expect	Rank	Unique	Peptide
15193	712.3483	1422.6821	1422.6813	0.56	0	33	0.031	1	U	R.LQDGLECGGAYLK.Y

Proteins matching the same set of peptides:
NbS00004679g0009.1 **Mass:** 70288 **Score:** 33 **Matches:** 1(1) **Sequences:** 1(1)
NbS00004679g0009.1 protein AED:0.34 eAED:0.35 QI:0|0.62|0.44|0.88|1|1|9|0|616; (*GB) gi|350539281|ref|NP_001234129.1| (e_value=0.0) calnexin-like protein precursor [Solanum lycopersicum];; (*SWP) s
NbS00010157g0101.1 **Mass:** 37572 **Score:** 33 **Matches:** 1(1) **Sequences:** 1(1)
NbS00010157g0101.1 protein AED:0.11 eAED:0.11 QI:0|0|0|0.5|1|1|4|0|327
NbS00025205g0016.1 **Mass:** 58304 **Score:** 33 **Matches:** 1(1) **Sequences:** 1(1)
NbS00025205g0016.1 protein AED:0.25 eAED:0.26 QI:0|0.66|0.57|1|1|1|7|642|508; (*GB) gi|350539281|ref|NP_001234129.1| (e_value=5e-120) calnexin-like protein precursor [Solanum lycopersicum];; (*SWP)
NbS00025205g0024.1 **Mass:** 65602 **Score:** 33 **Matches:** 1(1) **Sequences:** 1(1)
NbS00025205g0024.1 protein AED:0.09 eAED:0.09 QI:0|0.71|0.5|1|0.85|0.87|8|0|574; (*GB) gi|350539281|ref|NP_001234129.1| (e_value=0.0) calnexin-like protein precursor [Solanum lycopersicum];; (*SWP)

---

161. NbS00008764g0022.1 **Mass:** 31795 **Score:** 33 **Matches:** 1(1) **Sequences:** 1(1) **emPAI:** 0.10
NbS00008764g0022.1 protein AED:0.14 eAED:0.14 QI:72|1|1|1|0.6|0.66|6|317|286; (*GB) gi|255558218|ref|XP_002520136.1| (e_value=0.0) Protein PPLZ12, putative [Ricinus communis];; (*SWP) sp|Q9FM19|HI

Query	Observed	Mr(expt)	Mr(calc)	ppm	Miss	Score	Expect	Rank	Unique	Peptide
11122	475.2769	948.5393	948.5392	0.10	0	33	0.03	1	U	K.YLSGLGIAR.Q

Proteins matching the same set of peptides:
NbS00010140g0005.1 **Mass:** 32435 **Score:** 33 **Matches:** 1(1) **Sequences:** 1(1)
NbS00010140g0005.1 protein AED:0.06 eAED:0.06 QI:39|1|1|1|1|1|5|320|291; (*GB) gi|151347473|gb|ABS01349.1| (e_value=0.0) hypersensitive-induced response protein [Carica papaya];; (*SWP) sp|Q9FM19|H
NbS00030743g0005.1 **Mass:** 29452 **Score:** 33 **Matches:** 1(1) **Sequences:** 1(1)
NbS00030743g0005.1 protein AED:0.22 eAED:0.22 QI:125|0.8|0.66|1|0.4|0.5|6|0|264; (*GB) gi|151347473|gb|ABS01349.1| (e_value=4e-180) hypersensitive-induced response protein [Carica papaya];; (*SWP)

---

162. NbS00002134g0112.1 **Mass:** 24734 **Score:** 33 **Matches:** 1(1) **Sequences:** 1(1) **emPAI:** 0.14
NbS00002134g0112.1 protein AED:0.04 eAED:0.13 QI:0|1|0.33|1|1|1|3|0|218

Query	Observed	Mr(expt)	Mr(calc)	ppm	Miss	Score	Expect	Rank	Unique	Peptide
12933	585.8066	1169.5987	1169.5968	1.62	0	33	0.031	1	U	K.FLDGIYVSEK.G

Proteins matching the same set of peptides:
NbS00005119g0003.1 **Mass:** 22005 **Score:** 33 **Matches:** 1(1) **Sequences:** 1(1)
NbS00005119g0003.1 protein AED:0.27 eAED:0.27 QI:111|1|1|1|1|1|3|349|194; (*GB) gi|77416921|gb|ABA81856.1| (e_value=9e-133) unknown [Solanum tuberosum];; (*SWP) sp|P49209|RL91_ARATH (e_value=4e-12
NbS00005152g0006.1 **Mass:** 22032 **Score:** 33 **Matches:** 1(1) **Sequences:** 1(1)
NbS00005152g0006.1 protein AED:0.00 eAED:0.00 QI:111|1|1|1|0.5|0.33|3|349|194; (*GB) gi|77416921|gb|ABA81856.1| (e_value=2e-133) unknown [Solanum tuberosum];; (*SWP) sp|P49209|RL91_ARATH (e_value=

---

163. NbS00028158g0015.1 **Mass:** 18033 **Score:** 33 **Matches:** 1(0) **Sequences:** 1(0) **emPAI:** 0.19
NbS00028158g0015.1 protein AED:0.44 eAED:0.54 QI:0|0.75|0.4|1|1|1|5|0|169; (*GB) gi|388500696|gb|AFK38414.1| (e_value=1e-63) unknown [Lotus japonicus];; (*SWP) sp|O04616|Y4115_ARATH (e_value=4e-59)

Query	Observed	Mr(expt)	Mr(calc)	ppm	Miss	Score	Expect	Rank	Unique	Peptide
8544	594.3100	1186.6054	1186.6081	-2.23	0	33	0.094	1	U	K.ELAEDIEQLK.K

---

164. NbS00011040g0001.1 **Mass:** 28504 **Score:** 33 **Matches:** 1(1) **Sequences:** 1(1) **emPAI:** 0.12
NbS00011040g0001.1 protein AED:0.00 eAED:0.03 QI:0|1|0.66|1|0|0|3|164|270; (*GB) gi|255578051|ref|XP_002529896.1| (e_value=1e-58) small nuclear ribonucleoprotein-associated protein, putative [Rici

Query	Observed	Mr(expt)	Mr(calc)	ppm	Miss	Score	Expect	Rank	Unique	Peptide
12366	550.7816	1099.5487	1099.5484	0.24	0	33	0.032	1	U	K.MLQYINYR.M

Proteins matching the same set of peptides:
NbS00014429g0006.1 **Mass:** 33726 **Score:** 33 **Matches:** 1(1) **Sequences:** 1(1)
NbS00014429g0006.1 protein AED:0.21 eAED:0.23 QI:0|0|0|1|1|1|2|0|322; (*GB) gi|255578055|ref|XP_002529898.1| (e_value=4e-54) small nuclear ribonucleoprotein-associated protein, putative [Ricinus co

---

165. NbS00005419g0006.1 **Score:** 33 **Matches:** 1(1) **Sequences:** 1(1) **emPAI:** 0.10

NbS00005419g0006.1 protein

Query	Observed	Mr(expt)	Mr(calc)	ppm	Miss	Score	Expect	Rank	Unique	Peptide
9277	349.2336	696.4525	696.4534	-1.22	0	33	0.0056	2	U	K.IGGLPIK.G

---

166. NbS00009707g0011.1 **Mass:** 20710 **Score:** 33 **Matches:** 1(1) **Sequences:** 1(1) **emPAI:** 0.16
NbS00009707g0011.1 protein AED:0.22 eAED:0.22 QI:246|1|1|1|0|0.14|7|394|181; (*GB) gi|115501471|dbj|BAF34209.1| (e_value=7e-133) ADP ribosylation factor [Nicotiana tabacum];; (*SWP) sp|P36397|ARF1_

Query	Observed	Mr(expt)	Mr(calc)	ppm	Miss	Score	Expect	Rank	Unique	Peptide
16327	783.3849	1564.7553	1564.7522	2.01	0	33	0.034	1	U	K.NISFTVWDVGGQDK.I

Proteins matching the same set of peptides:
NbS00019729g0011.1 **Mass:** 5194 **Score:** 33 **Matches:** 1(1) **Sequences:** 1(1)
NbS00019729g0011.1 protein AED:0.61 eAED:0.80 QI:61|0|0.33|1|0|0.33|3|0|44
gi|297262255|ref|XP_001104802.2| **Mass:** 28165 **Score:** 33 **Matches:** 1(1) **Sequences:** 1(1)
gi|297262255|ref|XP_001104802.2| PREDICTED: ADP-ribosylation factor 3-like isoform 3 [Macaca mulatta]

---

167. NbS00004739g0001.1 **Mass:** 27728 **Score:** 33 **Matches:** 1(1) **Sequences:** 1(1) **emPAI:** 0.12
NbS00004739g0001.1 protein AED:0.17 eAED:0.17 QI:0|1|0.66|1|1|1|3|0|251; (*GB) gi|100801744|emb|CAK24966.1| (e_value=2e-175) chlorophyll a/b binding protein [Solanum tuberosum];; (*SWP) sp|Q9SQL2|(

Query	Observed	Mr(expt)	Mr(calc)	ppm	Miss	Score	Expect	Rank	Unique	Peptide
14302	658.3366	1314.6587	1314.6568	1.41	0	33	0.037	1	U	K.NPGSVNQDPIFK.N

Proteins matching the same set of peptides:
NbS00030208g0012.1 **Mass:** 25902 **Score:** 33 **Matches:** 1(1) **Sequences:** 1(1)
NbS00030208g0012.1 protein AED:0.05 eAED:0.05 QI:0|0.5|0|1|1|1|3|0|233; (*GB) gi|100801744|emb|CAK24966.1| (e_value=1e-158) chlorophyll a/b binding protein [Solanum tuberosum];; (*SWP) sp|Q9SQL2|CH
NbS00043913g0001.1 **Mass:** 27579 **Score:** 33 **Matches:** 1(1) **Sequences:** 1(1)
NbS00043913g0001.1 protein AED:0.36 eAED:0.36 QI:170|1|0.66|1|1|1|3|0|250; (*GB) gi|100801744|emb|CAK24966.1| (e_value=7e-166) chlorophyll a/b binding protein [Solanum tuberosum];; (*SWP) sp|P2752
NbS00052312g0004.1 **Mass:** 27591 **Score:** 33 **Matches:** 1(1) **Sequences:** 1(1)
NbS00052312g0004.1 protein AED:0.28 eAED:0.28 QI:166|1|0.66|1|0.5|0.66|3|0|250; (*SWP) sp|P27521|CA4_ARATH (e_value=1e-154) Chlorophyll a-b binding protein 4, chloroplastic OS=Arabidopsis thaliana

---

168. NbS00002935g0006.1 **Mass:** 54788 **Score:** 33 **Matches:** 1(1) **Sequences:** 1(1) **emPAI:** 0.06
NbS00002935g0006.1 protein AED:0.24 eAED:0.28 QI:9|0.53|0.35|1|0.84|0.71|14|0|493; (*GB) gi|6715512|gb|AAF26445.1| (e_value=0.0) vacuolar H+-ATPase B subunit [Nicotiana tabacum];; (*SWP) sp|Q9SZN1

Query	Observed	Mr(expt)	Mr(calc)	ppm	Miss	Score	Expect	Rank	Unique	Peptide
12261	544.7913	1087.5681	1087.5696	-1.37	0	33	0.047	1	U	K.TPVSLDMLGR.I

Proteins matching the same set of peptides:
NbS00006432g0020.1 **Mass:** 58076 **Score:** 33 **Matches:** 1(1) **Sequences:** 1(1)
NbS00006432g0020.1 protein AED:0.14 eAED:0.14 QI:0|0.85|0.86|1|0.78|0.66|15|354|521; (*GB) gi|357507939|ref|XP_003624258.1| (e_value=0.0) V-type ATP synthase beta chain [Medicago truncatula];; (*SW
NbS00007553g0003.1 **Mass:** 28303 **Score:** 33 **Matches:** 1(1) **Sequences:** 1(1)
NbS00007553g0003.1 protein AED:0.28 eAED:0.28 QI:132|0.93|0.93|1|0.66|0.62|16|917|258; (*GB) gi|224105123|ref|XP_002313695.1| (e_value=1e-175) predicted protein [Populus trichocarpa];; (*SWP) sp|Q
NbS00010495g0008.1 **Mass:** 62704 **Score:** 33 **Matches:** 1(1) **Sequences:** 1(1)
NbS00010495g0008.1 protein AED:0.05 eAED:0.05 QI:0|0.86|0.93|0.93|0.73|0.81|16|352|560; (*GB) gi|302141720|emb|CBI18923.3| (e_value=0.0) unnamed protein product [Vitis vinifera];; (*SWP) sp|Q9SZN1

---

169. NbS00001581g0007.1 **Mass:** 29112 **Score:** 33 **Matches:** 1(1) **Sequences:** 1(1) **emPAI:** 0.11
NbS00001581g0007.1 protein AED:0.31 eAED:0.31 QI:0|0.8|0.66|1|1|1|6|0|270; (*GB) gi|161788874|dbj|BAF95071.1| (e_value=4e-156) voltage-dependent anion channel [Nicotiana tabacum];; (*SWP) sp|P4205

Query	Observed	Mr(expt)	Mr(calc)	ppm	Miss	Score	Expect	Rank	Unique	Peptide
13590	623.8568	1245.6989	1245.6969	1.64	0	33	0.031	1	U	K.TIVSFVLPDQK.S

Proteins matching the same set of peptides:
NbS00016824g0007.1 **Mass:** 25084 **Score:** 33 **Matches:** 1(1) **Sequences:** 1(1)
NbS00016824g0007.1 protein AED:0.31 eAED:0.31 QI:93|0.8|0.83|1|1|1|6|0|236; (*GB) gi|161788874|dbj|BAF95071.1| (e_value=1e-156) voltage-dependent anion channel [Nicotiana tabacum];; (*SWP) sp|P420

---

170. NbS00005939g0108.1 **Mass:** 80747 **Score:** 33 **Matches:** 1(1) **Sequences:** 1(1) **emPAI:** 0.04
NbS00005939g0108.1 protein AED:0.21 eAED:0.21 QI:0|1|1|1|1|1|8|398|765

Query	Observed	Mr(expt)	Mr(calc)	ppm	Miss	Score	Expect	Rank	Unique	Peptide
15004	467.2564	1398.7473	1398.7467	0.44	1	33	0.035	1	U	K.AADVGADLVGKVER.N

Proteins matching the same set of peptides:
NbS00009733g0024.1 **Mass:** 82871 **Score:** 33 **Matches:** 1(1) **Sequences:** 1(1)
NbS00009733g0024.1 protein AED:0.22 eAED:0.22 QI:0|0.87|0.77|0.88|1|1|9|0|783; (*GB) gi|457744|emb|CAA54869.1| (e_value=0.0) inorganic pyrophosphatase [Nicotiana tabacum];; (*SWP) sp|P31414|AVP1_AI
NbS00024485g0009.1 **Mass:** 81397 **Score:** 33 **Matches:** 1(1) **Sequences:** 1(1)
NbS00024485g0009.1 protein AED:0.16 eAED:0.16 QI:0|1|0.87|1|1|1|8|0|769; (*GB) gi|457744|emb|CAA54869.1| (e_value=0.0) inorganic pyrophosphatase [Nicotiana tabacum];; (*SWP) sp|P31414|AVP1_ARATH (
NbS00025104g0012.1 **Mass:** 80481 **Score:** 33 **Matches:** 1(1) **Sequences:** 1(1)
NbS00025104g0012.1 protein AED:0.09 eAED:0.09 QI:186|1|1|1|1|1|8|378|767; (*GB) gi|1103712|emb|CAA58700.1| (e_value=0.0) inorganic pyrophosphatase [Nicotiana tabacum];; (*SWP) sp|P21616|AVP_VIGRR
NbS00030505g0009.1 **Mass:** 82888 **Score:** 33 **Matches:** 1(1) **Sequences:** 1(1)
NbS00030505g0009.1 protein AED:0.07 eAED:0.07 QI:0|1|0.75|1|1|1|8|0|790; (*GB) gi|1103712|emb|CAA58700.1| (e_value=0.0) inorganic pyrophosphatase [Nicotiana tabacum];; (*SWP) sp|P21616|AVP_VIGRR (
NbS00040188g0002.1 **Mass:** 81027 **Score:** 33 **Matches:** 1(1) **Sequences:** 1(1)
NbS00040188g0002.1 protein AED:0.20 eAED:0.20 QI:0|0.85|0.87|1|1|1|8|317|767; (*GB) gi|790479|emb|CAA58701.1| (e_value=0.0) inorganic pyrophosphatase [Nicotiana tabacum];; (*SWP) sp|P21616|AVP_VIGI
NbS00056299g0007.1 **Mass:** 84536 **Score:** 33 **Matches:** 1(1) **Sequences:** 1(1)
NbS00056299g0007.1 protein AED:0.04 eAED:0.04 QI:0|0.85|0.5|1|1|1|8|0|800; (*GB) gi|224123948|ref|XP_002330249.1| (e_value=0.0) vacuolar H+-translocating inorganic pyrophosphatase [Populus trichoca

---

171. NbS00007972g0032.1 **Score:** 33 **Matches:** 1(1) **Sequences:** 1(1) **emPAI:** 0.05
NbS00007972g0032.1 protein AED:0.25 eAED:0.25 QI:0|0.77|0.5|1|0.77|0.9|10|0|571; (*GB) gi|350534776|ref|NP_001234159.1| (e_value=0.0) pollen-specific kinase partner protein [Solanum lycopersicum];;

Query	Observed	Mr(expt)	Mr(calc)	ppm	Miss	Score	Expect	Rank	Unique	Peptide
5602	429.2600	856.5054	856.5130	-8.85	1	33	0.054	2	U	K.IVDLKNR.I

Proteins matching the same set of peptides:
NbS00025048g0008.1 **Score:** 33 **Matches:** 1(1) **Sequences:** 1(1)

---

172. NbS00002844g0211.1 **Mass:** 61594 **Score:** 33 **Matches:** 1(1) **Sequences:** 1(1) **emPAI:** 0.05
NbS00002844g0211.1 protein AED:0.10 eAED:0.10 QI:261|1|1|1|1|1|18|392|575

Query	Observed	Mr(expt)	Mr(calc)	ppm	Miss	Score	Expect	Rank	Unique	Peptide
10246	**422.2685**	**842.5225**	**842.5226**	**-0.07**	**0**	**33**	**0.03**	**1**	**U**	**K.ISGGVAVLK.I**

Proteins matching the same set of peptides:
NbS00010973g0003.1 **Mass:** 64481 **Score:** 33 **Matches:** 1(1) **Sequences:** 1(1)
NbS00010973g0003.1 protein AED:0.12 eAED:0.12 QI:275|1|0.88|1|1|1|18|212|599; (*GB) gi|255554262|ref|XP_002518171.1| (e_value=0.0) chaperonin-60kD, ch60, putative [Ricinus communis];; (*SWP) sp|Q0

---

173. NbS00008163g0010.1 **Mass:** 67295 **Score:** 33 **Matches:** 1(1) **Sequences:** 1(1) **emPAI:** 0.05
NbS00008163g0010.1 protein AED:0.09 eAED:0.09 QI:186|0.87|0.88|1|1|1|9|300|633; (*GB) gi|359479362|ref|XP_002277357.2| (e_value=0.0) PREDICTED: ruBisCO large subunit-binding protein subunit alpha,

Query	Observed	Mr(expt)	Mr(calc)	ppm	Miss	Score	Expect	Rank	Unique	Peptide
10246	422.2685	842.5225	842.5226	-0.07	0	33	0.03	1	U	K.LSGGVAVIK.V

Proteins matching the same set of peptides:
NbS00033391g0006.1 **Mass:** 59777 **Score:** 33 **Matches:** 1(1) **Sequences:** 1(1)
NbS00033391g0006.1 protein AED:0.14 eAED:0.14 QI:195|0.85|0.87|1|0.85|1|8|383|567; (*GB) gi|359479362|ref|XP_002277357.2| (e_value=0.0) PREDICTED: ruBisCO large subunit-binding protein subunit alph
NbS00040945g0003.1 **Mass:** 62276 **Score:** 33 **Matches:** 1(1) **Sequences:** 1(1)
NbS00040945g0003.1 protein AED:0.10 eAED:0.10 QI:277|1|0.87|1|1|1|8|307|589; (*GB) gi|359479362|ref|XP_002277357.2| (e_value=0.0) PREDICTED: ruBisCO large subunit-binding protein subunit alpha, ch

---

174. NbS00019145g0009.1 **Mass:** 65588 **Score:** 33 **Matches:** 1(1) **Sequences:** 1(1) **emPAI:** 0.05
NbS00019145g0009.1 protein AED:0.20 eAED:0.20 QI:118|0.77|0.78|1|1|1|19|310|609; (*GB) gi|255554262|ref|XP_002518171.1| (e_value=0.0) chaperonin-60kD, ch60, putative [Ricinus communis];; (*SWP) sp

Query	Observed	Mr(expt)	Mr(calc)	ppm	Miss	Score	Expect	Rank	Unique	Peptide
10246	422.2685	842.5225	842.5226	-0.07	0	33	0.03	1	U	K.LSGGVAVLK.V

Proteins matching the same set of peptides:
NbS00049065g0003.1 **Mass:** 66537 **Score:** 33 **Matches:** 1(1) **Sequences:** 1(1)
NbS00049065g0003.1 protein AED:0.16 eAED:0.17 QI:113|0.78|0.85|1|1|1|20|308|620; (*GB) gi|255554262|ref|XP_002518171.1| (e_value=0.0) chaperonin-60kD, ch60, putative [Ricinus communis];; (*SWP) sp

---

175. NbS00009748g0101.1 **Mass:** 67150 **Score:** 32 **Matches:** 1(1) **Sequences:** 1(1) **emPAI:** 0.05
NbS00009748g0101.1 protein AED:0.12 eAED:0.12 QI:0|-1|0|1|-1|1|1|0|600

Query	Observed	Mr(expt)	Mr(calc)	ppm	Miss	Score	Expect	Rank	Unique	Peptide

19503 694.4113 2080.2119 2080.2044 3.61 0 32 0.0063 1 U R.ALEALTGAVFQRPPLISAVK.R

Proteins matching the same set of peptides:
NbS00012890g0001.1 **Mass:** 60900 **Score:** 32 **Matches:** 1(1) **Sequences:** 1(1)
NbS00012890g0001.1 protein AED:0.20 eAED:0.21 QI:0|0|0|0.5|1|1|2|0|545; (*GB) gi|225440338|ref|XP_002270073.1| (e_value=0.0) PREDICTED: H/ACA ribonucleoprotein complex subunit 4 [Vitis vinifera];;

---

176. NbS00005120g0006.1 **Mass:** 18523 **Score:** 32 **Matches:** 1(1) **Sequences:** 1(1) **emPAI:** 0.18
NbS00005120g0006.1 protein

Query	Observed	Mr(expt)	Mr(calc)	ppm	Miss	Score	Expect	Rank	Unique	Peptide
12500	557.8274	1113.6402	1113.5852	49.4	1	32	0.032	1	U	-.MEKLSLEHK.L

---

177. NbS00008453g0109.1 **Mass:** 25017 **Score:** 32 **Matches:** 1(1) **Sequences:** 1(1) **emPAI:** 0.13
NbS00008453g0109.1 protein AED:0.28 eAED:0.44 QI:0|0|0|0.66|0.5|0.33|3|0|219

Query	Observed	Mr(expt)	Mr(calc)	ppm	Miss	Score	Expect	Rank	Unique	Peptide
20024	1141.5540	2281.0934	2281.0903	1.34	0	32	0.026	1	U	R.DVFAGIDPDLDAQVEFGAFQK.I

---

178. NbS00003332g0002.1 **Score:** 32 **Matches:** 1(1) **Sequences:** 1(1) **emPAI:** 0.05
NbS00003332g0002.1 protein AED:0.22 eAED:0.22 QI:0|0.90|0.83|0.91|0.90|0.83|12|422|580; (*GB) gi|255561807|ref|XP_002521913.1| (e_value=0.0) PLE, putative [Ricinus communis];; (*SWP) sp|Q9SIS3|MA65

Query	Observed	Mr(expt)	Mr(calc)	ppm	Miss	Score	Expect	Rank	Unique	Peptide
10611	447.7323	893.4500	893.4714	-23.92	1	32	0.051	2	U	R.MKELVMK.R

Proteins matching the same set of peptides:
NbS00013933g0004.1 **Mass:** 62499 **Score:** 32 **Matches:** 1(1) **Sequences:** 1(1)
NbS00013933g0004.1 protein AED:0.29 eAED:0.29 QI:45|1|0.8|1|1|0.9|10|0|549; (*GB) gi|255561807|ref|XP_002521913.1| (e_value=0.0) PLE, putative [Ricinus communis];; (*SWP) sp|Q9SIS3|MA656_ARATH (e_
NbS00019287g0001.1 **Score:** 32 **Matches:** 1(1) **Sequences:** 1(1)
NbS00038139g0004.1 **Score:** 32 **Matches:** 1(1) **Sequences:** 1(1)

---

179. NbS00010101g0016.1 **Score:** 32 **Matches:** 1(1) **Sequences:** 1(1) **emPAI:** 0.05
NbS00010101g0016.1 protein AED:0.09 eAED:0.09 QI:12|1|1|1|0.37|0.22|9|70|546; (*GB) gi|296084540|emb|CBI25561.3| (e_value=0.0) unnamed protein product [Vitis vinifera];; (*SWP) sp|Q9FZP1|HPSE3_ARA

Query	Observed	Mr(expt)	Mr(calc)	ppm	Miss	Score	Expect	Rank	Unique	Peptide
9913	399.7580	797.5015	797.5123	-13.54	0	32	0.015	1	U	K.VLLNAIR.A

---

180. NbS00010498g0007.1 **Mass:** 24174 **Score:** 32 **Matches:** 1(1) **Sequences:** 1(1) **emPAI:** 0.14
NbS00010498g0007.1 protein AED:0.12 eAED:0.12 QI:0|1|1|1|0.5|1|3|646|228; (*GB) gi|384038831|gb|AFH58006.1| (e_value=5e-128) chloroplast PsbQ2 precursor [Nicotiana benthamiana];; (*SWP) sp|P12301|I

Query	Observed	Mr(expt)	Mr(calc)	ppm	Miss	Score	Expect	Rank	Unique	Peptide
16584	802.9246	1603.8347	1603.8358	-0.70	0	32	0.037	1	U	R.FYLQPLTPAEAAQR.V

Proteins matching the same set of peptides:
NbS00013563g0003.1 **Mass:** 22296 **Score:** 32 **Matches:** 1(1) **Sequences:** 1(1)
NbS00013563g0003.1 protein AED:0.34 eAED:0.34 QI:0|0.66|0.5|1|1|1|4|0|212; (*GB) gi|384038829|gb|AFH58005.1| (e_value=4e-111) chloroplast PsbQ1 precursor [Nicotiana benthamiana];; (*SWP) sp|Q41932

---

181. NbS00014411g0036.1 **Mass:** 104466 **Score:** 32 **Matches:** 1(1) **Sequences:** 1(1) **emPAI:** 0.03
NbS00014411g0036.1 protein AED:0.22 eAED:0.22 QI:406|0.87|0.92|1|0.83|0.88|25|199|921; (*GB) gi|297743300|emb|CBI36167.3| (e_value=0.0) unnamed protein product [Vitis vinifera];; (*SWP) sp|Q9C827|(

Query	Observed	Mr(expt)	Mr(calc)	ppm	Miss	Score	Expect	Rank	Unique	Peptide
14375	661.3380	1320.6615	1320.6673	-4.44	0	32	0.051	1	U	R.LENTLNYGLER.V

Proteins matching the same set of peptides:
NbS00016861g0012.1 **Mass:** 115885 **Score:** 32 **Matches:** 1(1) **Sequences:** 1(1)
NbS00016861g0012.1 protein AED:0.23 eAED:0.23 QI:215|0.79|0.84|0.92|0.79|0.88|25|258|1016; (*GB) gi|297743300|emb|CBI36167.3| (e_value=0.0) unnamed protein product [Vitis vinifera];; (*SWP) sp|Q9C8
NbS00032342g0003.1 **Mass:** 104717 **Score:** 32 **Matches:** 1(1) **Sequences:** 1(1)
NbS00032342g0003.1 protein AED:0.23 eAED:0.23 QI:329|0.91|0.95|1|0.82|0.83|24|203|919; (*GB) gi|147789985|emb|CAN59846.1| (e_value=0.0) hypothetical protein VITISV_004513 [Vitis vinifera];; (*SWP)
NbS00051961g0006.1 **Mass:** 113516 **Score:** 32 **Matches:** 1(1) **Sequences:** 1(1)
NbS00051961g0006.1 protein AED:0.26 eAED:0.26 QI:0|0.69|0.66|1|0.86|0.83|24|390|994; (*SWP) sp|Q9C827|COB22_ARATH (e_value=0.0) Coatomer subunit beta'-2 OS=Arabidopsis thaliana GN=At1g52360 PE=2 SV

NbS00058604g0005.1 **Mass:** 98777 **Score:** 32 **Matches:** 1(1) **Sequences:** 1(1)
NbS00058604g0005.1 protein AED:0.24 eAED:0.24 QI:467|0.91|0.96|1|0.87|0.8|25|582|866; (*GB) gi|359482613|ref|XP_002284773.2| (e_value=0.0) PREDICTED: coatomer subunit beta'-2-like [Vitis vinifera];

182. gi|14198259|gb|AAH08189.1| **Mass:** 57189 **Score:** 32 **Matches:** 1(1) **Sequences:** 1(1) **emPAI:** 0.06
gi|14198259|gb|AAH08189.1| Unknown (protein for IMAGE:3584589), partial [Mus musculus]

Query	Observed	Mr(expt)	Mr(calc)	ppm	Miss	Score	Expect	Rank	Unique	Peptide
15943	764.4044	1526.7942	1526.7940	0.09	0	32	0.04	1	U	K.GIVDSEDLPLNISR.E

183. NbS00010129g0001.1 **Mass:** 35285 **Score:** 32 **Matches:** 1(1) **Sequences:** 1(1) **emPAI:** 0.09
NbS00010129g0001.1 protein AED:0.16 eAED:0.16 QI:0|0|0.5|1|1|1|2|131|318; (*SWP) sp|P23547|E13G_TOBAC (e_value=0.0) Glucan endo-1,3-beta-glucosidase, acidic isoform GI9 OS=Nicotiana tabacum GN=PR2

Query	Observed	Mr(expt)	Mr(calc)	ppm	Miss	Score	Expect	Rank	Unique	Peptide
15064	703.8943	1405.7740	1405.7718	1.60	0	32	0.034	1	U	K.SFTNPIIQFLAR.H

Proteins matching the same set of peptides:
NbC25742659g0003.1 **Mass:** 37798 **Score:** 32 **Matches:** 1(1) **Sequences:** 1(1)
NbC25742659g0003.1 protein AED:0.11 eAED:0.11 QI:96|1|1|1|1|1|2|0|342; (*GB) gi|119012|sp|P23547.1|E13G_TOBAC (e_value=0.0) RecName: Full=Glucan endo-1,3-beta-glucosidase, acidic isoform GI9; AltNa

184. NbS00010282g0017.1 **Mass:** 58711 **Score:** 32 **Matches:** 1(1) **Sequences:** 1(1) **emPAI:** 0.06
NbS00010282g0017.1 protein AED:0.32 eAED:0.32 QI:0|0.5|0.8|1|0.75|0.8|5|368|516; (*GB) gi|551259|emb|CAA57428.1| (e_value=0.0) beta-fructofuranosidase [Nicotiana tabacum];; (*SWP) sp|P26792|INV1_DA

Query	Observed	Mr(expt)	Mr(calc)	ppm	Miss	Score	Expect	Rank	Unique	Peptide
13124	597.2784	1192.5423	1192.5400	1.94	0	32	0.038	1	U	R.LDYGNYYASK.S

Proteins matching the same set of peptides:
NbS00033156g0002.1 **Mass:** 52216 **Score:** 32 **Matches:** 1(1) **Sequences:** 1(1)
NbS00033156g0002.1 protein AED:0.14 eAED:0.14 QI:1294|0.6|0.33|1|1|1|6|0|456; (*GB) gi|359431011|gb|AEV46316.1| (e_value=0.0) apoplastic invertase [Solanum tuberosum];; (*SWP) sp|Q39693|INV3_DAUCA
NbS00042078g0004.1 **Mass:** 53806 **Score:** 32 **Matches:** 1(1) **Sequences:** 1(1)
NbS00042078g0004.1 protein AED:0.07 eAED:0.07 QI:24|0.33|0.75|1|0.66|0.75|4|327|467; (*GB) gi|306849481|gb|ADN06440.1| (e_value=0.0) invertase [Solanum tuberosum];; (*SWP) sp|P26792|INV1_DAUCA (e_

185. NbS00004795g0001.1 **Mass:** 41688 **Score:** 32 **Matches:** 1(1) **Sequences:** 1(1) **emPAI:** 0.08
NbS00004795g0001.1 protein AED:0.06 eAED:0.06 QI:293|1|0.88|1|0.75|0.77|9|0|380; (*GB) gi|255561457|ref|XP_002521739.1| (e_value=1e-163) auxin:hydrogen symporter, putative [Ricinus communis];; (*TI

Query	Observed	Mr(expt)	Mr(calc)	ppm	Miss	Score	Expect	Rank	Unique	Peptide
20398	813.0918	2436.2536	2436.3062	-21.59	1	32	0.025	1	U	K.FVLMIQYTLPPAMNIGTFKRP.-

186. NbS00036843g0004.1 **Mass:** 42375 **Score:** 32 **Matches:** 1(1) **Sequences:** 1(1) **emPAI:** 0.08
NbS00036843g0004.1 protein AED:0.04 eAED:0.04 QI:8|0.5|0.66|1|0.5|0.33|3|0|387; (*SWP) sp|P25083|ADT1_SOLTU (e_value=0.0) ADP,ATP carrier protein, mitochondrial OS=Solanum tuberosum GN=ANT PE=2 SV=

Query	Observed	Mr(expt)	Mr(calc)	ppm	Miss	Score	Expect	Rank	Unique	Peptide
18691	956.9974	1911.9803	1911.9765	2.02	0	32	0.035	2	U	K.GVSGFAIDFLMGGVSAAVSK.T

187. NbS00002113g0001.1 **Mass:** 43922 **Score:** 31 **Matches:** 1(1) **Sequences:** 1(1) **emPAI:** 0.08
NbS00002113g0001.1 protein AED:0.00 eAED:0.05 QI:0|-1|0|1|-1|1|1|0|394; (*SWP) sp|Q00874|DR100_ARATH (e_value=1e-127) DNA-damage-repair/toleration protein DRT100 OS=Arabidopsis thaliana GN=DRT100 I

Query	Observed	Mr(expt)	Mr(calc)	ppm	Miss	Score	Expect	Rank	Unique	Peptide
9686	381.2369	760.4592	760.4595	-0.44	0	31	0.037	1	U	R.AALLAFR.A

Proteins matching the same set of peptides:
NbS00005919g0003.1 **Score:** 31 **Matches:** 1(1) **Sequences:** 1(1)
NbS00051233g0003.1 **Mass:** 19660 **Score:** 31 **Matches:** 1(1) **Sequences:** 1(1)
NbS00051233g0003.1 protein AED:0.31 eAED:0.31 QI:0|0|0|0.5|1|1|2|0|178; (*GB) gi|88604736|gb|ABD46739.1| (e_value=1e-100) leucine-rich repeat protein [Nicotiana tabacum];; (*SWP) sp|Q00874|DR100_AF

188. NbS00059593g0005.1 **Mass:** 52362 **Score:** 31 **Matches:** 1(1) **Sequences:** 1(1) **emPAI:** 0.06
NbS00059593g0005.1 protein AED:0.21 eAED:0.21 QI:386|1|1|1|0.87|0.88|9|535|469; (*GB) gi|296083732|emb|CBI23721.3| (e_value=1e-157) unnamed protein product [Vitis vinifera];; (*SWP) sp|O94260|G3BP_

Query	Observed	Mr(expt)	Mr(calc)	ppm	Miss	Score	Expect	Rank	Unique	Peptide
19358	1018.4871	2034.9597	2034.9481	5.72	0	31	0.033	1	U	R.SITEATEEASAVEDEVEVK.S

189. NbS00001259g0107.1 **Mass:** 49198 **Score:** 31 **Matches:** 1(1) **Sequences:** 1(1) **emPAI:** 0.07
NbS00001259g0107.1 protein AED:0.28 eAED:0.28 QI:192|0.66|0.4|1|1|1|10|0|433

Query	Observed	Mr(expt)	Mr(calc)	ppm	Miss	Score	Expect	Rank	Unique	Peptide
20230	784.0465	2349.1177	2349.1237	-2.56	0	31	0.033	1	U	R.YYVVTDSGDDDPINPRPGTLR.H

Proteins matching the same set of peptides:
NbS00012174g0129.1 **Mass:** 42216 **Score:** 31 **Matches:** 1(1) **Sequences:** 1(1)
NbS00012174g0129.1 protein AED:0.26 eAED:0.26 QI:190|0.83|0.57|1|1|1|7|0|373

190. NbS00005007g0108.1 **Mass:** 77527 **Score:** 31 **Matches:** 1(1) **Sequences:** 1(1) **emPAI:** 0.04
NbS00005007g0108.1 protein AED:0.09 eAED:0.09 QI:0|1|0.75|1|1|1|4|444|693

Query	Observed	Mr(expt)	Mr(calc)	ppm	Miss	Score	Expect	Rank	Unique	Peptide
12318	547.8325	1093.6505	1093.6496	0.84	0	31	0.017	1	U	R.GVLLVGPPGTGK.T

Proteins matching the same set of peptides:
NbS00006672g0030.1 **Mass:** 79846 **Score:** 31 **Matches:** 1(1) **Sequences:** 1(1)
NbS00006672g0030.1 protein AED:0.30 eAED:0.30 QI:233|0.5|0.42|1|0.5|0.71|7|0|734; (*GB) gi|350539019|ref|NP_001234370.1| (e_value=0.0) FtsH protease-like [Solanum lycopersicum];; (*SWP) sp|O80983|I
NbS00024131g0006.1 **Mass:** 76478 **Score:** 31 **Matches:** 1(1) **Sequences:** 1(1)
NbS00024131g0006.1 protein AED:0.11 eAED:0.11 QI:0|1|0.5|1|1|1|4|0|684; (*GB) gi|359488350|ref|XP_002279064.2| (e_value=0.0) PREDICTED: ATP-dependent zinc metalloprotease FtsH-like [Vitis vinifera]
NbS00033574g0005.1 **Mass:** 78703 **Score:** 31 **Matches:** 1(1) **Sequences:** 1(1)
NbS00033574g0005.1 protein AED:0.18 eAED:0.18 QI:346|0.83|0.85|1|0.66|0.71|7|485|732; (*GB) gi|350539019|ref|NP_001234370.1| (e_value=0.0) FtsH protease-like [Solanum lycopersicum];; (*SWP) sp|O809
NbS00055649g0004.1 **Mass:** 67273 **Score:** 31 **Matches:** 1(1) **Sequences:** 1(1)
NbS00055649g0004.1 protein AED:0.26 eAED:0.27 QI:237|0.5|0.42|1|0.5|0.42|7|0|614; (*GB) gi|350539019|ref|NP_001234370.1| (e_value=0.0) FtsH protease-like [Solanum lycopersicum];; (*SWP) sp|Q8LQJ8|I

191. NbS00007190g0001.1 **Mass:** 30678 **Score:** 31 **Matches:** 1(1) **Sequences:** 1(1) **emPAI:** 0.11
NbS00007190g0001.1 protein AED:0.08 eAED:0.08 QI:179|1|1|1|0.5|0.33|3|443|275; (*GB) gi|225448932|ref|XP_002267178.1| (e_value=1e-93) PREDICTED: heterogeneous nuclear ribonucleoprotein G [Vitis vir

Query	Observed	Mr(expt)	Mr(calc)	ppm	Miss	Score	Expect	Rank	Unique	Peptide
14961	697.3700	1392.7254	1392.7249	0.39	0	31	0.047	1	U	R.IFVGGLSSDITER.Q

Proteins matching the same set of peptides:
NbS00029940g0009.1 **Mass:** 30714 **Score:** 31 **Matches:** 1(1) **Sequences:** 1(1)
NbS00029940g0009.1 protein AED:0.36 eAED:0.36 QI:205|1|1|1|0.5|0.66|3|523|275; (*GB) gi|225448932|ref|XP_002267178.1| (e_value=6e-80) PREDICTED: heterogeneous nuclear ribonucleoprotein G [Vitis vir

192. NbS00005969g0002.1 **Mass:** 63583 **Score:** 31 **Matches:** 1(1) **Sequences:** 1(1) **emPAI:** 0.05
NbS00005969g0002.1 protein AED:0.07 eAED:0.07 QI:0|0.5|0.55|0.88|1|1|9|177|561; (*GB) gi|225456270|ref|XP_002283518.1| (e_value=0.0) PREDICTED: nucleolar protein 56-like [Vitis vinifera];; (*SWP) s

Query	Observed	Mr(expt)	Mr(calc)	ppm	Miss	Score	Expect	Rank	Unique	Peptide
17507	843.9419	1685.8692	1685.8658	2.03	0	31	0.046	1	U	K.ASMGQDLSPVDLINVK.M

Proteins matching the same set of peptides:
NbS00023900g0001.1 **Mass:** 64240 **Score:** 31 **Matches:** 1(1) **Sequences:** 1(1)
NbS00023900g0001.1 protein AED:0.11 eAED:0.11 QI:0|0.85|0.75|1|1|1|8|178|567; (*SWP) sp|O94514|NOP56_SCHPO (e_value=0.0) Nucleolar protein 56 OS=Schizosaccharomyces pombe (strain 972 / ATCC 24843)
NbS00031401g0006.1 **Mass:** 35669 **Score:** 31 **Matches:** 1(1) **Sequences:** 1(1)
NbS00031401g0006.1 protein AED:0.13 eAED:0.13 QI:0|0.66|0.42|0.85|0.66|0.57|7|0|316; (*GB) gi|255540297|ref|XP_002511213.1| (e_value=0.0) nucleolar protein nop56, putative [Ricinus communis];; (*SW

193. NbC24284457g0001.1 **Mass:** 12773 **Score:** 31 **Matches:** 1(0) **Sequences:** 1(0) **emPAI:** 0.27
NbC24284457g0001.1 protein

Query	Observed	Mr(expt)	Mr(calc)	ppm	Miss	Score	Expect	Rank	Unique	Peptide
11195	479.7698	957.5250	957.5495	-25.54	1	31	0.067	1	U	-.AAKADIIEK.E

194. NbS00011397g0001.1 **Mass:** 14976 **Score:** 31 **Matches:** 1(1) **Sequences:** 1(1) **emPAI:** 0.23
NbS00011397g0001.1 protein AED:0.61 eAED:0.61 QI:0|0|0|0.5|1|1|2|0|130; (*SWP) sp|A1XQR9|RUXE_PIG (e_value=3e-31) Small nuclear ribonucleoprotein E OS=Sus scrofa GN=SNRPE PE=3 SV=1;; (*TAIR) AT4G3(

Query	Observed	Mr(expt)	Mr(calc)	ppm	Miss	Score	Expect	Rank	Unique	Peptide
13235	602.8400	1203.6655	1203.6652	0.27	0	31	0.052	1	U	R.IQIWLFEQK.D

Proteins matching the same set of peptides:
NbS00011643g0015.1 **Mass:** 10334 **Score:** 31 **Matches:** 1(1) **Sequences:** 1(1)
NbS00011643g0015.1 protein AED:0.07 eAED:0.07 QI:151|1|1|1|0.8|0.83|6|432|88; (*GB) gi|356508935|ref|XP_003523208.1| (e_value=2e-56) PREDICTED: small nuclear ribonucleoprotein E-like [Glycine max];
NbS00034990g0011.1 **Mass:** 14072 **Score:** 31 **Matches:** 1(1) **Sequences:** 1(1)
NbS00034990g0011.1 protein AED:0.13 eAED:0.13 QI:153|0.8|0.83|1|0.8|0.66|6|389|118; (*GB) gi|356562483|ref|XP_003549500.1| (e_value=1e-53) PREDICTED: small nuclear ribonucleoprotein E [Glycine max]
NbS00046899g0003.1 **Mass:** 9817 **Score:** 31 **Matches:** 1(1) **Sequences:** 1(1)
NbS00046899g0003.1 protein AED:0.19 eAED:0.19 QI:0|0|0|1|1|1|2|0|84; (*GB) gi|356508935|ref|XP_003523208.1| (e_value=4e-50) PREDICTED: small nuclear ribonucleoprotein E-like [Glycine max];; (*SWP)
NbS00056919g0001.1 **Mass:** 9341 **Score:** 31 **Matches:** 1(1) **Sequences:** 1(1)
NbS00056919g0001.1 protein AED:0.17 eAED:0.17 QI:148|0.8|0.83|1|0.8|0.83|6|459|79; (*SWP) sp|A1XQR9|RUXE_PIG (e_value=1e-33) Small nuclear ribonucleoprotein E OS=Sus scrofa GN=SNRPE PE=3 SV=1;; (*

---

195. NbS00005851g0011.1 **Mass:** 74074 **Score:** 31 **Matches:** 1(1) **Sequences:** 1(1) **emPAI:** 0.04
NbS00005851g0011.1 protein AED:0.18 eAED:0.18 QI:3|0.89|0.85|1|0.78|0.9|20|310|678; (*GB) gi|224062133|ref|XP_002300771.1| (e_value=0.0) predicted protein [Populus trichocarpa];; (*SWP) sp|P04844|

Query	Observed	Mr(expt)	Mr(calc)	ppm	Miss	Score	Expect	Rank	Unique	Peptide
17274	828.4706	1654.9267	1654.9254	0.81	0	31	0.031	1	U	K.VNVNTVLGSAAPSLSVK.L

Proteins matching the same set of peptides:
NbS00033230g0212.1 **Mass:** 53328 **Score:** 31 **Matches:** 1(1) **Sequences:** 1(1)
NbS00033230g0212.1 protein AED:0.24 eAED:0.24 QI:3|0.84|0.78|1|1|0.92|14|0|493

---

196. NbS00001525g0121.1 **Mass:** 62315 **Score:** 30 **Matches:** 1(0) **Sequences:** 1(0) **emPAI:** 0.05
NbS00001525g0121.1 protein AED:0.18 eAED:0.18 QI:3|0.62|0.22|1|0.75|0.77|9|0|561

Query	Observed	Mr(expt)	Mr(calc)	ppm	Miss	Score	Expect	Rank	Unique	Peptide
11326	486.7773	971.5400	971.5400	-0.01	0	30	0.075	1	U	K.IGAATALEAR.A

Proteins matching the same set of peptides:
NbS00017675g0018.1 **Mass:** 79118 **Score:** 30 **Matches:** 1(0) **Sequences:** 1(0)
NbS00017675g0018.1 protein AED:0.22 eAED:0.23 QI:3|0.44|0.2|1|0.77|0.8|10|0|714; (*GB) gi|356565758|ref|XP_003551104.1| (e_value=0.0) PREDICTED: lysosomal beta glucosidase-like [Glycine max];; (*SW

---

197. NbS00003153g0003.1 **Mass:** 106016 **Score:** 30 **Matches:** 1(1) **Sequences:** 1(1) **emPAI:** 0.03
NbS00003153g0003.1 protein AED:0.15 eAED:0.15 QI:171|0.94|0.94|1|0.88|0.89|19|592|920; (*SWP) sp|O82188|RDR5_ARATH (e_value=0.0) Probable RNA-dependent RNA polymerase 5 OS=Arabidopsis thaliana GN=

Query	Observed	Mr(expt)	Mr(calc)	ppm	Miss	Score	Expect	Rank	Unique	Peptide
11442	493.7563	985.4981	985.5193	-21.49	0	30	0.056	1	U	K.NLGDVVGNAK.Y

---

198. NbS00014412g0011.1 **Mass:** 23512 **Score:** 30 **Matches:** 1(0) **Sequences:** 1(0) **emPAI:** 0.14
NbS00014412g0011.1 protein AED:0.27 eAED:0.27 QI:0|1|0|1|1|1|2|0|213; (*GB) gi|42559164|sp|O80360.1|RK3_TOBAC (e_value=2e-131) RecName: Full=50S ribosomal protein L3, chloroplastic; Flags: Precurs

Query	Observed	Mr(expt)	Mr(calc)	ppm	Miss	Score	Expect	Rank	Unique	Peptide
8623	607.8300	1213.6454	1213.6415	3.24	0	30	0.064	1	U	R.QLGSIGAGTTPGR.V

Proteins matching the same set of peptides:
NbS00024457g0004.1 **Mass:** 29972 **Score:** 30 **Matches:** 1(0) **Sequences:** 1(0)
NbS00024457g0004.1 protein AED:0.00 eAED:0.00 QI:0|1|0.5|1|1|1|2|0|274; (*SWP) sp|O80360|RK3_TOBAC (e_value=3e-156) 50S ribosomal protein L3, chloroplastic (Fragment) OS=Nicotiana tabacum GN=RPL3

---

199. NbS00001563g0013.1 **Mass:** 32139 **Score:** 30 **Matches:** 1(1) **Sequences:** 1(1) **emPAI:** 0.10
NbS00001563g0013.1 protein AED:0.00 eAED:0.03 QI:0|1|0.66|1|1|1|3|0|297; (*GB) gi|225451038|ref|XP_002284939.1| (e_value=4e-145) PREDICTED: UPF0603 protein At1g54780, chloroplastic [Vitis vinifera]

Query	Observed	Mr(expt)	Mr(calc)	ppm	Miss	Score	Expect	Rank	Unique	Peptide
17366	830.9692	1659.9239	1659.9196	2.62	0	30	0.036	1	U	R.LVAAIDGLPDPGGPQLK.D

Proteins matching the same set of peptides:
NbS00009383g0013.1 **Mass:** 32296 **Score:** 30 **Matches:** 1(1) **Sequences:** 1(1)
NbS00009383g0013.1 protein AED:0.00 eAED:0.03 QI:0|1|0.66|1|1|1|3|0|297; (*GB) gi|225451038|ref|XP_002284939.1| (e_value=5e-139) PREDICTED: UPF0603 protein At1g54780, chloroplastic [Vitis vinifera]

---

200. NbS00002808g0033.1 **Mass:** 62817 **Score:** 30 **Matches:** 1(1) **Sequences:** 1(1) **emPAI:** 0.05
NbS00002808g0033.1 protein AED:0.14 eAED:0.14 QI:0|1|0.94|1|0.88|0.94|18|425|576; (*GB) gi|225433255|ref|XP_002285452.1| (e_value=0.0) PREDICTED: U-box domain-containing protein 72 [Vitis vinifera]

Query	Observed	Mr(expt)	Mr(calc)	ppm	Miss	Score	Expect	Rank	Unique	Peptide
15640	742.4098	1482.8050	1482.8042	0.57	0	30	0.046	1	U	R.QIPATLASVDALER.Y

Proteins matching the same set of peptides:
NbS00015167g0014.1 **Mass:** 62356 **Score:** 30 **Matches:** 1(1) **Sequences:** 1(1)
NbS00015167g0014.1 protein AED:0.27 eAED:0.27 QI:0|1|1|1|0.88|0.88|18|478|568; (*SWP) sp|Q94BR4|PR19A_ARATH (e_value=0.0) Pre-mRNA-processing factor 19 homolog 1 OS=Arabidopsis thaliana GN=PRP19A I

Mascot: http://www.matrixscience.com/
