# Supplementary material for: Alternative splicing regulation in plants by SP7-like effectors from symbiotic arbuscular mycorrhizal fungi
Source: Nat Commun. 2024 Aug 19;15:7107. doi: 10.1038/s41467-024-51512-5 (PMC11333574; doi:10.1038/s41467-024-51512-5)
Supplement: Supplementary file 10 — Source Data [file 41467_2024_51512_MOESM10_ESM.zip › Requena_8071-2_validation (RiSP7ΓêåSP).pdf]

Mascot Search Results

```
User              :
Email             :
Search title      : 8071-2
MS data file      : \\Server3\user\Kunden_Projekte\Requena_8071\8071\8071_2.mgf
Database          : Jessi 1 (4 sequences; 1216 residues)
Timestamp         : 1 Dec 2016 at 13:49:23 GMT
Significant hits: eGFP
                  SP7delta
```

Probability Based Mowse Score

Ions score is -10*Log(P), where P is the probability that the observed match is a random event.
Individual ions scores > 4 indicate identity or extensive homology (p<0.05).
Protein scores are derived from ions scores as a non-probabilistic basis for ranking protein hits.

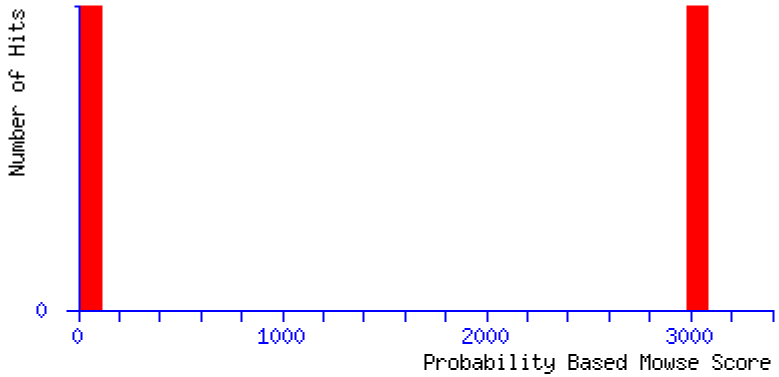

Peptide Summary Report

Format As | Peptide Summary | Help

Significance threshold p< 0.05   Max. number of hits AUTO

Standard scoring ○ MudPIT scoring ◉ Ions score cut-off 20 Show sub-sets ☐

Show pop-ups ◉ Suppress pop-ups ○ Sort unassigned Decreasing Score Require bold red ☐

Select All | Select None | Search Selected | ☐ **Error tolerant** | Archive Report

1. eGFP **Mass:** 27039 **Score:** 3029 **Queries matched:** 72

☐ Check to include this hit in error tolerant search or archive report

Query	Observed	Mr(expt)	Mr(calc)	Delta	Miss	Score	Expect	Rank	Peptide
☑ 8605	602.2800	601.2727	601.2707	0.0020	0	20	0.00048	1	K.DPNEK.R
☑ 10127	413.7100	825.4054	825.4055	-0.0001	0	36	1.4e-005	1	K.FICTTGK.L
☑ 11931	525.7641	1049.5136	1049.5141	-0.0005	0	41	4.1e-006	1	K.FEGDTLVNR.I
☑ 13751	633.7914	1265.5683	1265.5710	-0.0027	0	(64)	1.8e-008	1	K.SAMPEGYVQER.T
☑ 13752	633.7915	1265.5685	1265.5710	-0.0025	0	67	9e-009	1	K.SAMPEGYVQER.T
☑ 13754	633.7920	1265.5694	1265.5710	-0.0016	0	(30)	4.5e-005	1	K.SAMPEGYVQER.T
☑ 13756	633.7929	1265.5713	1265.5710	0.0003	0	(65)	1.6e-008	1	K.SAMPEGYVQER.T
☑ 13757	633.7930	1265.5715	1265.5710	0.0005	0	(21)	0.00043	1	K.SAMPEGYVQER.T
☑ 13957	641.7902	1281.5658	1281.5659	-0.0001	0	(58)	7.2e-008	1	K.SAMPEGYVQER.T + Oxidation (M)
☑ 13958	641.7902	1281.5659	1281.5659	-0.0000	0	(48)	7.6e-007	1	K.SAMPEGYVQER.T + Oxidation (M)
☑ 13962	641.7904	1281.5663	1281.5659	0.0004	0	(66)	1.4e-008	1	K.SAMPEGYVQER.T + Oxidation (M)
☑ 13970	641.7906	1281.5666	1281.5659	0.0007	0	(51)	3.8e-007	1	K.SAMPEGYVQER.T + Oxidation (M)
☑ 13972	641.7906	1281.5666	1281.5659	0.0007	0	(23)	0.00027	1	K.SAMPEGYVQER.T + Oxidation (M)
☑ 13980	641.7908	1281.5671	1281.5659	0.0012	0	(24)	0.00022	1	K.SAMPEGYVQER.T + Oxidation (M)
☑ 13984	641.7910	1281.5674	1281.5659	0.0015	0	(24)	0.00021	1	K.SAMPEGYVQER.T + Oxidation (M)
☑ 13989	641.7912	1281.5678	1281.5659	0.0019	0	(26)	0.00013	1	K.SAMPEGYVQER.T + Oxidation (M)
☑ 14586	674.3325	1346.6505	1346.6506	-0.0001	1	(27)	9.4e-005	1	R.TIFFKDDGNYK.T
☑ 14589	674.3327	1346.6509	1346.6506	0.0002	1	(26)	0.00012	1	R.TIFFKDDGNYK.T
☑ 14591	674.3328	1346.6510	1346.6506	0.0004	1	(44)	2e-006	1	R.TIFFKDDGNYK.T
☑ 14592	674.3328	1346.6510	1346.6506	0.0004	1	(20)	0.00047	1	R.TIFFKDDGNYK.T
☑ 14593	674.3329	1346.6513	1346.6506	0.0007	1	(24)	0.00019	1	R.TIFFKDDGNYK.T
☑ 14595	674.3330	1346.6515	1346.6506	0.0008	1	(34)	1.9e-005	1	R.TIFFKDDGNYK.T

☑ 14599	674.3331	1346.6517	1346.6506	0.0011	1	44	1.8e-006	1	R.TIFFKDDGNYK.T
☑ 14602	674.3333	1346.6521	1346.6506	0.0015	1	(42)	3.4e-006	1	R.TIFFKDDGNYK.T
☑ 14603	674.3334	1346.6522	1346.6506	0.0016	1	(39)	6.1e-006	1	R.TIFFKDDGNYK.T
☑ 14605	674.3334	1346.6522	1346.6506	0.0016	1	(37)	9.5e-006	1	R.TIFFKDDGNYK.T
☑ 14607	674.3336	1346.6527	1346.6506	0.0021	1	(20)	0.00045	1	R.TIFFKDDGNYK.T
☑ 15582	493.2596	1476.7569	1476.7572	-0.0003	1	(36)	1.3e-005	1	R.AEVKFEGDTLVNR.I
☑ 15586	739.3860	1476.7575	1476.7572	0.0004	1	(48)	8.2e-007	1	R.AEVKFEGDTLVNR.I
☑ 15587	493.2598	1476.7576	1476.7572	0.0004	1	(39)	6.1e-006	1	R.AEVKFEGDTLVNR.I
☑ 15590	493.2599	1476.7579	1476.7572	0.0007	1	(30)	5.6e-005	1	R.AEVKFEGDTLVNR.I
☑ 15592	493.2599	1476.7580	1476.7572	0.0008	1	(23)	0.00025	1	R.AEVKFEGDTLVNR.I
☑ 15593	739.3863	1476.7580	1476.7572	0.0008	1	82	3.4e-010	1	R.AEVKFEGDTLVNR.I
☑ 15597	739.3864	1476.7582	1476.7572	0.0010	1	(31)	3.6e-005	1	R.AEVKFEGDTLVNR.I
☑ 15598	739.3871	1476.7596	1476.7572	0.0024	1	(31)	4.3e-005	1	R.AEVKFEGDTLVNR.I
☑ 15768	752.3326	1502.6506	1502.6524	-0.0018	0	(25)	0.00018	1	K.FSVSGEGEGDATYGK.L
☑ 15769	752.3326	1502.6507	1502.6524	-0.0017	0	(92)	3.3e-011	1	K.FSVSGEGEGDATYGK.L
☑ 15772	752.3328	1502.6511	1502.6524	-0.0013	0	(21)	0.00042	1	K.FSVSGEGEGDATYGK.L
☑ 15774	752.3330	1502.6515	1502.6524	-0.0010	0	(27)	9.3e-005	1	K.FSVSGEGEGDATYGK.L
☑ 15775	752.3331	1502.6517	1502.6524	-0.0007	0	(44)	2.1e-006	1	K.FSVSGEGEGDATYGK.L
☑ 15777	752.3333	1502.6520	1502.6524	-0.0005	0	(27)	9.2e-005	1	K.FSVSGEGEGDATYGK.L
☑ 15778	752.3333	1502.6521	1502.6524	-0.0004	0	(22)	0.00035	1	K.FSVSGEGEGDATYGK.L
☑ 15780	752.3334	1502.6523	1502.6524	-0.0001	0	(78)	7.3e-010	1	K.FSVSGEGEGDATYGK.L
☑ 15781	752.3335	1502.6524	1502.6524	0.0000	0	(60)	4.7e-008	1	K.FSVSGEGEGDATYGK.L
☑ 15782	752.3335	1502.6524	1502.6524	0.0000	0	(36)	1.2e-005	1	K.FSVSGEGEGDATYGK.L
☑ 15784	752.3336	1502.6527	1502.6524	0.0002	0	(43)	2.4e-006	1	K.FSVSGEGEGDATYGK.L
☑ 15788	752.3337	1502.6529	1502.6524	0.0005	0	(82)	3e-010	1	K.FSVSGEGEGDATYGK.L
☑ 15791	752.3338	1502.6530	1502.6524	0.0006	0	(62)	3.3e-008	1	K.FSVSGEGEGDATYGK.L
☑ 15792	752.3338	1502.6530	1502.6524	0.0006	0	(27)	9.7e-005	1	K.FSVSGEGEGDATYGK.L
☑ 15793	752.3339	1502.6532	1502.6524	0.0007	0	(79)	6.2e-010	1	K.FSVSGEGEGDATYGK.L
☑ 15794	752.3339	1502.6532	1502.6524	0.0007	0	(52)	3.4e-007	1	K.FSVSGEGEGDATYGK.L
☑ 15795	752.3339	1502.6533	1502.6524	0.0009	0	104	2.1e-012	1	K.FSVSGEGEGDATYGK.L
☑ 15799	752.3340	1502.6535	1502.6524	0.0011	0	(63)	2.6e-008	1	K.FSVSGEGEGDATYGK.L
☑ 15801	752.3341	1502.6537	1502.6524	0.0012	0	(50)	4.7e-007	1	K.FSVSGEGEGDATYGK.L

☑ 15802	752.3341	1502.6537	1502.6524	0.0012	0	(44)	1.9e-006	1	K.FSVSGEGEGDATYGK.L
☑ 15803	752.3342	1502.6538	1502.6524	0.0013	0	(88)	7.7e-011	1	K.FSVSGEGEGDATYGK.L
☑ 15805	752.3342	1502.6538	1502.6524	0.0013	0	(30)	5.2e-005	1	K.FSVSGEGEGDATYGK.L
☑ 15806	752.3342	1502.6539	1502.6524	0.0015	0	(25)	0.00016	1	K.FSVSGEGEGDATYGK.L
☑ 15807	752.3344	1502.6542	1502.6524	0.0017	0	(54)	1.8e-007	1	K.FSVSGEGEGDATYGK.L
☑ 15808	752.3344	1502.6542	1502.6524	0.0017	0	(33)	2.5e-005	1	K.FSVSGEGEGDATYGK.L
☑ 15811	752.3345	1502.6545	1502.6524	0.0021	0	(82)	3.1e-010	1	K.FSVSGEGEGDATYGK.L
☑ 15814	752.3348	1502.6550	1502.6524	0.0026	0	(21)	0.0004	1	K.FSVSGEGEGDATYGK.L
☑ 15815	752.3351	1502.6556	1502.6524	0.0032	0	(32)	2.9e-005	1	K.FSVSGEGEGDATYGK.L
☑ 15987	767.4178	1532.8211	1532.8198	0.0013	1	22	0.00029	1	K.FEGDTLVNRIELK.G
☑ 16051	514.9349	1541.7828	1541.7837	-0.0009	1	40	5.1e-006	1	K.GIDFKEDGNILGHK.L
☑ 16053	514.9351	1541.7833	1541.7837	-0.0004	1	(32)	3.4e-005	1	K.GIDFKEDGNILGHK.L
☑ 16057	514.9353	1541.7841	1541.7837	0.0003	1	(28)	8.6e-005	1	K.GIDFKEDGNILGHK.L
☑ 16061	514.9355	1541.7848	1541.7837	0.0011	1	(24)	0.00021	1	K.GIDFKEDGNILGHK.L
☑ 16063	771.9011	1541.7876	1541.7837	0.0038	1	(31)	4.4e-005	1	K.GIDFKEDGNILGHK.L
☑ 18975	979.9897	1957.9649	1957.9632	0.0017	1	44	1.9e-006	1	K.FSVSGEGEGDATYGKLTLK.F
☑ 19105	987.4547	1972.8949	1972.8988	-0.0040	0	24	0.00019	1	K.LEYNYNSHNVYIMADK.Q
☑ 20401	1219.1364	2436.2582	2436.2535	0.0046	0	42	6.2e-006	1	K.GEELFTGVVPILVELDGDVNGHK.F

---

2. SP7delta **Mass:** 54931 **Score:** 64 **Queries matched:** 4

☐ Check to include this hit in error tolerant search or archive report

Query	Observed	Mr(expt)	Mr(calc)	Delta	Miss	Score	Expect	Rank	Peptide
☑ 8857	320.6740	639.3335	639.3340	-0.0005	1	22	0.0063	1	K.SSYKR.S
☑ 17914	879.8936	1757.7726	1757.7744	-0.0018	1	21	0.0023	1	R.SALASPGDKDDDYYGGK.D
☑ 18058	596.9353	1787.7841	1787.7849	-0.0009	1	(20)	0.0018	1	R.SALASPGDKDDDYYGSK.D
☑ 18063	894.9007	1787.7868	1787.7849	0.0019	1	24	0.00072	1	R.SALASPGDKDDDYYGSK.D

Proteins matching the same set of peptides:

SP7full **Mass:** 57161 **Score:** 64 **Queries matched:** 4

Peptide matches not assigned to protein hits: (no details means no match)

	Query	Observed	Mr(expt)	Mr(calc)	Delta	Miss	Score	Expect	Rank	Peptide
☑	18504	938.3651	1874.7155	1874.7118	0.0037	1	19	0.013	1	DDDYYGSKDDDYYGGK
☑	13750	633.7914	1265.5682	1265.5710	-0.0028	0	19	0.00057	1	SAMPEGYVQER
☑	15591	493.2599	1476.7579	1476.7572	0.0007	1	19	0.00061	1	AEVKFEGDTLVNR
☑	19108	658.6409	1972.9010	1972.8988	0.0021	0	19	0.0007	1	LEYNYNSHNVYIMADK
☑	13983	641.7909	1281.5672	1281.5659	0.0013	0	19	0.0007	1	SAMPEGYVQER + Oxidation (M)
☑	13976	641.7907	1281.5669	1281.5659	0.0010	0	19	0.00071	1	SAMPEGYVQER + Oxidation (M)
☑	13979	641.7908	1281.5671	1281.5659	0.0012	0	18	0.00074	1	SAMPEGYVQER + Oxidation (M)
☑	13969	641.7906	1281.5666	1281.5659	0.0007	0	18	0.00075	1	SAMPEGYVQER + Oxidation (M)
☑	15796	752.3340	1502.6534	1502.6524	0.0010	0	17	0.00091	1	FSVSGEGEGDATYGK
☑	13973	641.7906	1281.5667	1281.5659	0.0008	0	17	0.00092	1	SAMPEGYVQER + Oxidation (M)
☑	16058	771.8994	1541.7842	1541.7837	0.0004	1	17	0.00093	1	GIDFKEDGNILGHK
☑	9855	395.6810	789.3474	789.3479	-0.0005	0	16	0.0011	1	YPDHMK
☑	8944	328.1938	654.3731	654.3741	-0.0010	0	16	0.0012	1	TIFFK
☑	18351	923.3601	1844.7057	1844.7013	0.0044	1	16	0.0096	1	DDDYYGGKDDDYYGGK
☑	15800	752.3341	1502.6537	1502.6524	0.0012	0	16	0.0013	1	FSVSGEGEGDATYGK
☑	13963	641.7904	1281.5663	1281.5659	0.0004	0	16	0.0014	1	SAMPEGYVQER + Oxidation (M)
☑	14598	674.3331	1346.6517	1346.6506	0.0011	1	15	0.0014	1	TIFFKDDGNYK
☑	13960	641.7903	1281.5660	1281.5659	0.0001	0	15	0.0015	1	SAMPEGYVQER + Oxidation (M)
☑	15816	752.3351	1502.6557	1502.6524	0.0033	0	15	0.0015	1	FSVSGEGEGDATYGK
☑	13952	641.7899	1281.5653	1281.5659	-0.0006	0	15	0.0016	1	SAMPEGYVQER + Oxidation (M)
☑	20598	861.4341	2581.2804	2581.2807	-0.0003	0	15	0.0016	1	DHMVLLEFVTAAGITLGMDELYK + Oxidation (M)
☑	15813	752.3348	1502.6550	1502.6524	0.0026	0	15	0.0017	1	FSVSGEGEGDATYGK
☑	17917	879.8958	1757.7771	1757.7744	0.0027	1	15	0.01	1	SALASPGDKDDDYYGGK
☑	13762	633.7936	1265.5727	1265.5710	0.0017	0	14	0.0018	1	SAMPEGYVQER
☑	13988	641.7911	1281.5677	1281.5659	0.0018	0	14	0.0018	1	SAMPEGYVQER + Oxidation (M)
☑	13953	641.7900	1281.5654	1281.5659	-0.0005	0	14	0.0021	1	SAMPEGYVQER + Oxidation (M)
☑	15820	752.3363	1502.6581	1502.6524	0.0056	0	14	0.0021	1	FSVSGEGEGDATYGK
☑	11404	491.7516	981.4887	981.4879	0.0008	0	14	0.0022	1	EDGNILGHK
☑	13978	641.7908	1281.5671	1281.5659	0.0012	0	14	0.0022	1	SAMPEGYVQER + Oxidation (M)
☑	12158	538.2322	1074.4499	1074.4506	-0.0006	0	13	0.0047	1	DSTPSYDYK

☑ 15785	752.3336	1502.6527	1502.6524	0.0002	0	13	0.0025	1	FSVSGEGEGDATYGK
☑ 11932	525.7643	1049.5140	1049.5141	-0.0001	0	13	0.0025	1	FEGDTLVNR
☑ 16059	514.9354	1541.7844	1541.7837	0.0007	1	13	0.0025	1	GIDFKEDGNILGHK
☑ 15817	752.3354	1502.6562	1502.6524	0.0038	0	13	0.0028	1	FSVSGEGEGDATYGK
☑ 13965	641.7904	1281.5663	1281.5659	0.0004	0	12	0.003	1	SAMPEGYVQER + Oxidation (M)
☑ 15600	493.2613	1476.7620	1476.7572	0.0048	1	12	0.003	1	AEVKFEGDTLVNR
☑ 8943	655.3800	654.3727	654.3741	-0.0013	0	12	0.003	1	TIFFK
☑ 16062	771.8997	1541.7849	1541.7837	0.0011	1	12	0.0031	1	GIDFKEDGNILGHK
☑ 14581	674.3322	1346.6499	1346.6506	-0.0007	1	12	0.0031	1	TIFFKDDGNYK
☑ 13753	633.7917	1265.5689	1265.5710	-0.0020	0	12	0.0032	1	SAMPEGYVQER
☑ 15767	752.3325	1502.6504	1502.6524	-0.0021	0	12	0.0032	1	FSVSGEGEGDATYGK
☑ 8946	328.1942	654.3738	654.3741	-0.0003	0	12	0.0032	1	TIFFK
☑ 13967	641.7905	1281.5664	1281.5659	0.0005	0	12	0.0033	1	SAMPEGYVQER + Oxidation (M)
☑ 13941	641.7888	1281.5630	1281.5659	-0.0029	0	11	0.0037	1	SAMPEGYVQER + Oxidation (M)
☑ 13961	641.7903	1281.5661	1281.5659	0.0002	0	11	0.0037	1	SAMPEGYVQER + Oxidation (M)
☑ 13954	641.7900	1281.5654	1281.5659	-0.0005	0	11	0.0037	1	SAMPEGYVQER + Oxidation (M)
☑ 17916	586.9323	1757.7749	1757.7744	0.0006	1	11	0.023	1	SALASPGDKDDDYYGGK
☑ 14594	674.3329	1346.6513	1346.6506	0.0007	1	11	0.0039	1	TIFFKDDGNYK
☑ 13974	641.7906	1281.5667	1281.5659	0.0008	0	11	0.004	1	SAMPEGYVQER + Oxidation (M)
☑ 13977	641.7908	1281.5670	1281.5659	0.0011	0	11	0.0042	1	SAMPEGYVQER + Oxidation (M)
☑ 13946	641.7894	1281.5643	1281.5659	-0.0016	0	11	0.0043	1	SAMPEGYVQER + Oxidation (M)
☑ 13975	641.7907	1281.5669	1281.5659	0.0010	0	10	0.0047	1	SAMPEGYVQER + Oxidation (M)
☑ 15589	739.3861	1476.7577	1476.7572	0.0005	1	10	0.0047	1	AEVKFEGDTLVNR
☑ 15819	752.3354	1502.6563	1502.6524	0.0039	0	10	0.0048	1	FSVSGEGEGDATYGK
☑ 12159	538.2327	1074.4508	1074.4506	0.0002	0	10	0.01	1	DSTPSYDYK
☑ 8945	328.1939	654.3733	654.3741	-0.0007	0	10	0.005	1	TIFFK
☑ 13986	641.7910	1281.5675	1281.5659	0.0016	0	10	0.0051	1	SAMPEGYVQER + Oxidation (M)
☑ 11934	525.7826	1049.5506	1049.5141	0.0365	0	10	0.0052	1	FEGDTLVNR
☑ 13950	641.7898	1281.5650	1281.5659	-0.0009	0	10	0.0056	1	SAMPEGYVQER + Oxidation (M)
☑ 14596	674.3331	1346.6516	1346.6506	0.0010	1	9	0.0057	1	TIFFKDDGNYK
☑ 13966	641.7905	1281.5664	1281.5659	0.0005	0	9	0.0059	1	SAMPEGYVQER + Oxidation (M)
☑ 19910	744.0255	2229.0547	2229.0524	0.0023	1	9	0.006	1	LEYNYNSHNVYIMADKQK

☑ 13971	641.7906	1281.5666	1281.5659	0.0007	0	8	0.0072	1	SAMPEGYVQER + Oxidation (M)
☑ 19110	658.6410	1972.9011	1972.8988	0.0023	0	8	0.0072	1	LEYNYNSHNVYIMADK
☑ 15770	752.3327	1502.6509	1502.6524	-0.0016	0	8	0.0074	1	FSVSGEGEGDATYGK
☑ 19109	658.6410	1972.9011	1972.8988	0.0023	0	8	0.0077	1	LEYNYNSHNVYIMADK
☑ 11933	525.7665	1049.5184	1049.5141	0.0043	0	8	0.0077	1	FEGDTLVNR
☑ 13987	641.7910	1281.5675	1281.5659	0.0016	0	8	0.0088	1	SAMPEGYVQER + Oxidation (M)
☑ 15804	752.3342	1502.6538	1502.6524	0.0013	0	8	0.0088	1	FSVSGEGEGDATYGK
☑ 8858	320.6742	639.3339	639.3340	-0.0001	1	8	0.16	1	SSYKR
☑ 19106	987.4554	1972.8962	1972.8988	-0.0026	0	7	0.0091	1	LEYNYNSHNVYIMADK
☑ 14580	449.8904	1346.6492	1346.6506	-0.0014	1	7	0.0092	1	TIFFKDDGNYK
☑ 10936	466.6854	931.3563	931.3559	0.0004	0	7	0.32	1	DDDYYGGK
☑ 15789	752.3337	1502.6529	1502.6524	0.0005	0	7	0.0099	1	FSVSGEGEGDATYGK
☑ 14604	674.3334	1346.6522	1346.6506	0.0016	1	7	0.01	1	TIFFKDDGNYK
☑ 16054	514.9352	1541.7839	1541.7837	0.0002	1	7	0.011	1	GIDFKEDGNILGHK
☑ 15596	739.3864	1476.7582	1476.7572	0.0010	1	7	0.011	1	AEVKFEGDTLVNR
☑ 13949	641.7897	1281.5649	1281.5659	-0.0010	0	7	0.011	1	SAMPEGYVQER + Oxidation (M)
☑ 19909	744.0253	2229.0542	2229.0524	0.0018	1	6	0.011	1	LEYNYNSHNVYIMADKQK
☑ 15787	752.3337	1502.6528	1502.6524	0.0004	0	6	0.011	1	FSVSGEGEGDATYGK
☑ 13936	641.7875	1281.5604	1281.5659	-0.0055	0	6	0.011	1	SAMPEGYVQER + Oxidation (M)
☑ 15584	493.2597	1476.7574	1476.7572	0.0002	1	6	0.012	1	AEVKFEGDTLVNR
☑ 15585	493.2597	1476.7574	1476.7572	0.0002	1	6	0.012	1	AEVKFEGDTLVNR
☑ 13947	641.7894	1281.5643	1281.5659	-0.0016	0	6	0.013	1	SAMPEGYVQER + Oxidation (M)
☑ 13945	641.7894	1281.5642	1281.5659	-0.0017	0	6	0.013	1	SAMPEGYVQER + Oxidation (M)
☑ 10128	413.7100	825.4055	825.4055	0.0000	0	6	0.013	1	FICTTGK
☑ 10098	411.2478	820.4810	820.3868	0.0942	0	6	0.013	1	QHDFFK
☑ 13937	641.7875	1281.5604	1281.5659	-0.0055	0	6	0.014	1	SAMPEGYVQER + Oxidation (M)
☑ 19226	995.4539	1988.8933	1988.8938	-0.0005	0	6	0.014	1	LEYNYNSHNVYIMADK + Oxidation (M)
☑ 13944	641.7893	1281.5641	1281.5659	-0.0018	0	6	0.014	1	SAMPEGYVQER + Oxidation (M)
☑ 18967	979.9891	1957.9637	1957.9632	0.0005	1	5	0.014	1	FSVSGEGEGDATYGKLTLK
☑ 18062	894.9003	1787.7861	1787.7849	0.0012	1	5	0.06	1	SALASPGDKDDDYYGSK
☑ 10820	460.2714	918.5282	918.5286	-0.0005	1	5	0.015	1	NGIKVNFK
☑ 15990	767.4188	1532.8230	1532.8198	0.0032	1	5	0.016	1	FEGDTLVNRIELK

☑ 15809	752.3344	1502.6543	1502.6524	0.0018	0	5	0.016	1	FSVSGEGEGDATYGK
☑ 20567	856.1267	2565.3583	2565.2858	0.0725	0	5	0.017	1	DHMVLLEFVTAAGITLGMDELYK
☑ 15594	493.2600	1476.7581	1476.7572	0.0009	1	5	0.018	1	AEVKFEGDTLVNR
☑ 15790	752.3338	1502.6530	1502.6524	0.0006	0	4	0.018	1	FSVSGEGEGDATYGK
☑ 13938	641.7879	1281.5613	1281.5659	-0.0046	0	4	0.018	1	SAMPEGYVQER + Oxidation (M)
☑ 13759	633.7933	1265.5720	1265.5710	0.0010	0	4	0.019	1	SAMPEGYVQER
☑ 15583	493.2597	1476.7573	1476.7572	0.0001	1	4	0.019	1	AEVKFEGDTLVNR
☑ 19233	663.9727	1988.8963	1988.8938	0.0026	0	4	0.019	1	LEYNYNSHNVYIMADK + Oxidation (M)
☑ 17918	879.8963	1757.7780	1757.7744	0.0037	1	4	0.11	1	SALASPGDKDDDYYGGK
☑ 15776	752.3331	1502.6517	1502.6524	-0.0007	0	4	0.02	1	FSVSGEGEGDATYGK
☑ 13940	641.7885	1281.5624	1281.5659	-0.0036	0	4	0.02	1	SAMPEGYVQER + Oxidation (M)
☑ 15798	752.3340	1502.6534	1502.6524	0.0010	0	4	0.021	1	FSVSGEGEGDATYGK
☑ 9967	403.6792	805.3439	805.3429	0.0010	0	3	0.023	1	YPDHMK + Oxidation (M)
☑ 13990	641.7927	1281.5709	1281.5659	0.0050	0	3	0.024	1	SAMPEGYVQER + Oxidation (M)
☑ 13985	641.7910	1281.5674	1281.5659	0.0015	0	3	0.026	1	SAMPEGYVQER + Oxidation (M)
☑ 15773	752.3329	1502.6513	1502.6524	-0.0011	0	3	0.026	1	FSVSGEGEGDATYGK
☑ 15783	752.3336	1502.6527	1502.6524	0.0002	0	3	0.027	1	FSVSGEGEGDATYGK
☑ 13981	641.7908	1281.5671	1281.5659	0.0012	0	3	0.027	1	SAMPEGYVQER + Oxidation (M)
☑ 15818	752.3354	1502.6563	1502.6524	0.0039	0	3	0.028	1	FSVSGEGEGDATYGK
☑ 18503	625.9114	1874.7125	1874.7118	0.0006	1	3	0.61	1	DDDYYGSKDDDYYGGK
☑ 15779	752.3334	1502.6522	1502.6524	-0.0002	0	3	0.028	1	FSVSGEGEGDATYGK
☑ 14585	449.8907	1346.6504	1346.6506	-0.0002	1	2	0.029	1	TIFFKDDGNYK
☑ 15771	752.3327	1502.6509	1502.6524	-0.0016	0	2	0.03	1	FSVSGEGEGDATYGK
☑ 15595	493.2600	1476.7581	1476.7572	0.0009	1	2	0.031	1	AEVKFEGDTLVNR
☑ 15588	739.3861	1476.7577	1476.7572	0.0005	1	2	0.032	1	AEVKFEGDTLVNR
☑ 13959	641.7902	1281.5659	1281.5659	-0.0000	0	2	0.033	1	SAMPEGYVQER + Oxidation (M)
☑ 20400	813.0933	2436.2580	2436.2535	0.0044	0	2	0.07	1	GEELFTGVVPILVELDGDVNGHK
☑ 13939	641.7883	1281.5621	1281.5659	-0.0038	0	2	0.035	1	SAMPEGYVQER + Oxidation (M)
☑ 19504	694.9918	2081.9536	2082.0489	-0.0953	1	1	0.073	1	VSMFTCVQALIENLKDAK + Oxidation (M)
☑ 15825	752.4117	1502.8089	1502.6524	0.1565	0	1	0.038	1	FSVSGEGEGDATYGK
☑ 12676	570.3425	1138.6705	1138.6346	0.0359	0	1	0.039	1	APIAEIGLNNK
☑ 13968	641.7905	1281.5665	1281.5659	0.0006	0	1	0.039	1	SAMPEGYVQER + Oxidation (M)

☑ 20398	813.0918	2436.2536	2436.2535	0.0000	0	1	0.079	1	GEELFTGVVPILVELDGDVNGHK
☑ 13964	641.7904	1281.5663	1281.5659	0.0004	0	1	0.04	1	SAMPEGYVQER + Oxidation (M)
☑ 13755	633.7928	1265.5710	1265.5710	0.0000	0	1	0.041	1	SAMPEGYVQER
☑ 13951	641.7899	1281.5652	1281.5659	-0.0007	0	1	0.043	1	SAMPEGYVQER + Oxidation (M)
☑ 13943	641.7892	1281.5639	1281.5659	-0.0020	0	1	0.043	1	SAMPEGYVQER + Oxidation (M)
☑ 16055	514.9352	1541.7839	1541.7837	0.0002	1	1	0.044	1	GIDFKEDGNILGHK
☑ 13982	641.7909	1281.5672	1281.5659	0.0013	0	0	0.045	1	SAMPEGYVQER + Oxidation (M)
☑ 13935	641.7860	1281.5575	1281.5659	-0.0084	0	0	0.047	1	SAMPEGYVQER + Oxidation (M)
☑ 13956	641.7901	1281.5656	1281.5659	-0.0003	0	0	0.048	1	SAMPEGYVQER + Oxidation (M)
☑ 13758	633.7932	1265.5718	1265.5710	0.0008	0	0	0.049	1	SAMPEGYVQER
☑ 1	*300.0100*	*299.0027*							
☑ 2	*300.0100*	*299.0027*							
☑ 3	*300.0100*	*299.0027*							
☑ 4	*300.2000*	*299.1927*							
☑ 5	*300.2000*	*299.1927*							
☑ 6	*300.2000*	*299.1927*							
☑ 7	*300.2000*	*299.1927*							
☑ 8	*300.2100*	*299.2027*							
☑ 9	*300.2100*	*299.2027*							
☑ 10	*300.2900*	*299.2827*							
☑ 11	*301.0300*	*300.0227*							
☑ 12	*301.0300*	*300.0227*							
☑ 13	*301.0300*	*300.0227*							
☑ 14	*301.0300*	*300.0227*							
☑ 15	*301.0300*	*300.0227*							
☑ 16	*301.0300*	*300.0227*							
☑ 17	*301.1400*	*300.1327*							
☑ 18	*301.1400*	*300.1327*							
☑ 19	*301.1400*	*300.1327*							
☑ 20	*301.1400*	*300.1327*							
☑ 21	*301.1400*	*300.1327*							
☑ 22	*301.1400*	*300.1327*							
