# Supplementary material for: Alternative splicing regulation in plants by SP7-like effectors from symbiotic arbuscular mycorrhizal fungi
Source: Nat Commun. 2024 Aug 19;15:7107. doi: 10.1038/s41467-024-51512-5 (PMC11333574; doi:10.1038/s41467-024-51512-5)
Supplement: Supplementary file 10 — Source Data [file 41467_2024_51512_MOESM10_ESM.zip › Requena_8071-4_ProteinSummary (eGFP).pdf]

Mascot Search Results

```
User                     :
Email                    :
Search title             : 8071-4
MS data file             : 8071_4.mgf
Database                 : TopLab_8071 8071_20160923 (76598 sequences; 22305840 residues)
Timestamp                : 1 Dec 2016 at 14:17:27 GMT
Enzyme                   : Trypsin
Fixed modifications      : Carbamidomethyl (C)
Variable modifications   : Oxidation (M)
Mass values              : Monoisotopic
Protein Mass             : Unrestricted
Peptide Mass Tolerance   : ± 50 ppm
Fragment Mass Tolerance: ± 0.6 Da
Max Missed Cleavages     : 1
Instrument type          : Default
Number of queries        : 21020
Protein hits             : gi|297262447|ref|XP_001098182.2| gi|297262447|ref|XP_001098182.2| PREDICTED: keratin, type II cytoskeletal 1-like isoform 6 [Macaca mulatta]
                           gi|28317|emb|CAA32649.1|         gi|28317|emb|CAA32649.1| unnamed protein product [Homo sapiens]
                           gi|291410763|ref|XP_002721657.1| gi|291410763|ref|XP_002721657.1| PREDICTED: histone cluster 1, H2ag-like [Oryctolagus cuniculus]
                           NbS00000666g0001.1               NbS00000666g0001.1 protein AED:0.15 eAED:0.15 QI:0|-1|0|1|-1|1|1|0|145; (*GB) gi|82400146|gb|ABB72812.1| (e_value=5e-61) histone H2B-like protein
                           NbS00004898g0002.1               NbS00004898g0002.1 protein AED:0.00 eAED:0.00 QI:0|-1|0|1|-1|1|1|0|103; (*GB) gi|195617694|gb|ACG30677.1| (e_value=1e-51) histone H4 [Zea mays];;
                           NbS00017066g0003.1               NbS00017066g0003.1 protein AED:0.01 eAED:0.01 QI:0|-1|0|1|-1|1|1|0|208; (*GB) gi|222051768|dbj|BAH15357.1| (e_value=3e-127) germin like protein [
                           gi|148727309|ref|NP_001092039.1| gi|148727309|ref|NP_001092039.1| keratin, type II cytoskeletal 2 epidermal [Pan troglodytes]
                           NbS00000987g0004.1               NbS00000987g0004.1 protein AED:0.21 eAED:0.21 QI:0|-1|0|1|-1|1|1|0|266; (*GB) gi|3036948|dbj|BAA25392.1| (e_value=0.0) light harvesting chlorophy
                           NbS00000471g0009.1               NbS00000471g0009.1 protein AED:0.15 eAED:0.15 QI:0|0|0.33|1|1|1|3|0|301; (*GB) gi|115802|sp|P27494.1|CB23_TOBAC (e_value=0.0) RecName: Full=Chlor
                           NbS00030156g0009.1               NbS00030156g0009.1 protein AED:0.29 eAED:0.29 QI:170|1|0.9|1|0.77|0.7|10|232|281; (*GB) gi|358248282|ref|NP_001239854.1| (e_value=2e-70) uncharac
                           NbS00001169g0153.1               NbS00001169g0153.1 protein AED:0.25 eAED:0.25 QI:0|0.5|0.4|1|1|1|5|0|341
                           NbS00007993g0305.1               NbS00007993g0305.1 protein AED:0.00 eAED:0.00 QI:0|-1|0|1|-1|1|1|0|261
                           NbS00005377g0008.1               NbS00005377g0008.1 protein AED:0.46 eAED:0.46 QI:0|0|0|1|1|1|2|0|258; (*GB) gi|3036946|dbj|BAA25391.1| (e_value=2e-175) light harvesting chloroph
                           NbS00010295g0007.1               NbS00010295g0007.1 protein AED:0.12 eAED:0.12 QI:0|0|0|0.5|1|1|2|0|119; (*SWP) sp|P69569|RBL_EUPES (e_value=5e-22) Ribulose bisphosphate carboxyl
                           NbS00010583g0001.1               NbS00010583g0001.1 protein AED:0.25 eAED:0.25 QI:0|-1|0|1|-1|1|1|0|210; (*GB) gi|31711507|dbj|BAC77634.1| (e_value=2e-132) 24K germin like protei
                           NbS00004085g0015.1               NbS00004085g0015.1 protein AED:0.29 eAED:0.29 QI:92|0.25|0.2|1|1|1|5|0|225; (*GB) gi|110377793|gb|ABG73417.1| (e_value=7e-87) chloroplast pigment
                           NbS00001942g0006.1               NbS00001942g0006.1 protein AED:0.00 eAED:0.00 QI:75|1|1|1|1|1|2|198|151; (*GB) gi|351725389|ref|NP_001235555.1| (e_value=1e-63) uncharacterized p
                           NbS00035687g0007.1               NbS00035687g0007.1 protein AED:0.23 eAED:0.23 QI:300|1|0.75|1|1|1|4|0|274; (*GB) gi|84620802|gb|ABC59515.1| (e_value=9e-120) chloroplast photosys
                           NbS00007511g0005.1               NbS00007511g0005.1 protein AED:0.26 eAED:0.26 QI:0|0.62|0.55|0.88|1|1|9|0|1084; (*GB) gi|359477631|ref|XP_002274485.2| (e_value=0.0) PREDICTED: u
                           NbS00009638g0019.1               NbS00009638g0019.1 protein AED:0.11 eAED:0.11 QI:333|1|1|1|1|1|3|176|181; (*GB) gi|132118|sp|P26573.1|RBS8_NICPL (e_value=6e-118) RecName: Full=R
                           NbS00004956g0015.1               NbS00004956g0015.1 protein AED:0.25 eAED:0.30 QI:0|0|0|1|0|0|2|0|84; (*GB) gi|42718201|gb|AAS38532.1| (e_value=7e-41) ribulose-1,5-bisphosphate c
                           gi|119581148|gb|EAW60744.1|      gi|119581148|gb|EAW60744.1| keratin 9 (epidermolytic palmoplantar keratoderma) [Homo sapiens]
                           NbS00017935g0003.1               NbS00017935g0003.1 protein AED:0.00 eAED:0.00 QI:240|1|0.5|1|1|1|2|0|284; (*SWP) sp|Q9XF88|CB4B_ARATH (e_value=4e-162) Chlorophyll a-b binding pr
                           NbS00004238g0018.1               NbS00004238g0018.1 protein AED:0.16 eAED:0.16 QI:156|0.87|0.88|1|0.87|0.77|9|398|304; (*GB) gi|77416949|gb|ABA81870.1| (e_value=2e-140) unknown [
                           NbS00004744g0013.1               NbS00004744g0013.1 protein AED:0.20 eAED:0.20 QI:0|1|1|1|1|1|2|316|133; (*GB) gi|118484619|gb|ABK94182.1| (e_value=3e-58) unknown [Populus trichc
                           NbS00010009g0002.1               NbS00010009g0002.1 protein AED:0.20 eAED:0.20 QI:0|1|1|1|1|1|21|408|1058; (*SWP) sp|O04379|AGO1_ARATH (e_value=0.0) Protein argonaute 1 OS=Arabid
                           NbS00026922g0005.1               NbS00026922g0005.1 protein AED:0.33 eAED:0.34 QI:0|0.75|0.4|0.8|1|1|5|0|468; (*GB) gi|225451915|ref|XP_002282805.1| (e_value=1e-124) PREDICTED: u
                           NbS00010743g0014.1               NbS00010743g0014.1 protein AED:0.24 eAED:0.24 QI:0|1|1|1|1|1|8|290|288; (*GB) gi|77416949|gb|ABA81870.1| (e_value=2e-155) unknown [Solanum tuberc
                           NbS00043180g0003.1               NbS00043180g0003.1 protein AED:0.10 eAED:0.10 QI:0|1|0.5|1|1|1|4|944|486; (*GB) gi|113461|sp|P27081.1|ADT2_SOLTU (e_value=0.0) RecName: Full=ADP,
                           NbS00014429g0006.1               NbS00014429g0006.1 protein AED:0.21 eAED:0.23 QI:0|0|0|1|1|1|2|0|322; (*GB) gi|255578055|ref|XP_002529898.1| (e_value=4e-54) small nuclear ribonu
                           NbS00045545g0004.1               NbS00045545g0004.1 protein AED:0.13 eAED:0.13 QI:0|0.78|0.73|0.86|0.92|0.86|15|481|616; (*SWP) sp|Q7XTT4|NUCL2_ORYSJ (e_value=6e-73) Nucleolin 2
                           NbS00008911g0002.1               NbS00008911g0002.1 protein AED:0.35 eAED:0.35 QI:460|0.77|0.7|1|1|1|10|229|443; (*GB) gi|327198779|emb|CBL43264.1| (e_value=0.0) glyceraldehyde-3
                           NbS00003763g0016.1               NbS00003763g0016.1 protein AED:0.10 eAED:0.12 QI:0|0.92|0.8|0.93|1|1|15|0|884; (*GB) gi|225441896|ref|XP_002284404.1| (e_value=0.0) PREDICTED: un
                           NbS00027742g0013.1               NbS00027742g0013.1 protein AED:0.00 eAED:0.01 QI:0|1|1|1|1|1|7|309|363; (*GB) gi|77416977|gb|ABA81884.1| (e_value=4e-80) nuclear RNA binding prot
                           NbS00002894g0003.1               NbS00002894g0003.1 protein AED:0.02 eAED:0.02 QI:0|1|0.5|1|1|1|2|0|386; (*GB) gi|78191448|gb|ABB29945.1| (e_value=0.0) ADP/ATP translocator-like
                           NbS00002044g0005.1               NbS00002044g0005.1 protein AED:0.28 eAED:0.28 QI:0|1|0.66|1|1|1|3|0|273; (*GB) gi|226872|prf||1609235A (e_value=3e-155) chlorophyll a/b binding p
                           NbS00027807g0002.1               NbS00027807g0002.1 protein AED:0.05 eAED:0.05 QI:0|1|0.5|1|0|0|2|953|413; (*GB) gi|68566313|sp|Q40450.2|EFTUA_NICSY (e_value=0.0) RecName: Full=E
                           NbS00003163g0002.1               NbS00003163g0002.1 protein  ; (*GB) gi|146188483|emb|CAK12837.1| (e_value=1e-52) ribulose 1,5 biphosphate carboxylase/oxygenase [Liparia genistc
                           NbS00013071g0004.1               NbS00013071g0004.1 protein AED:0.33 eAED:0.33 QI:0|-1|0|1|-1|1|1|0|106; (*SWP) sp|P27524|CB4A_SOLLC (e_value=3e-69) Chlorophyll a-b binding prote
                           NbS00036430g0009.1               NbS00036430g0009.1 protein AED:0.35 eAED:0.35 QI:0|0|0|1|0|0|4|0|448; (*GB) gi|113170490|ref|YP_717281.1| (e_value=2e-170) Atp1 [Ostreococcus tau
                           sp|TRYP_PIG|                     sp|TRYP_PIG|
                           NbS00000634g0101.1               NbS00000634g0101.1 protein AED:0.21 eAED:0.21 QI:0|-1|0|1|-1|1|1|0|136
                           NbS00042812g0008.1               NbS00042812g0008.1 protein AED:0.19 eAED:0.19 QI:0|1|1|1|1|1|4|386|180; (*GB) gi|132118|sp|P26573.1|RBS8_NICPL (e_value=3e-119) RecName: Full=Rib
```

<table>
<tr><td>NbS00018705g0023.1</td><td>NbS00018705g0023.1 protein AED:0.30 eAED:0.31 QI:0|0.66|0.75|1|0.33|0.75|4|388|71; (*GB) gi|242050850|ref|XP_002463169.1| (e_value=7e-39) hypothe</td></tr>
<tr><td>NbS00019305g0027.1</td><td>NbS00019305g0027.1 protein AED:0.37 eAED:0.37 QI:0|1|0.88|1|1|1|9|0|556; (*GB) gi|3676296|gb|AAD03392.1| (e_value=0.0) mitochondrial ATPase beta</td></tr>
<tr><td>NbS00003075g0011.1</td><td>NbS00003075g0011.1 protein AED:0.28 eAED:0.28 QI:0|1|0.66|1|1|1|3|0|143; (*GB) gi|2499967|sp|Q41229.1|PSAEB_NICSY (e_value=1e-59) RecName: Full=P</td></tr>
<tr><td>NbS00008430g0013.1</td><td>NbS00008430g0013.1 protein AED:0.20 eAED:0.20 QI:6|0.90|0.86|0.95|0.90|0.95|22|289|1122; (*GB) gi|84688908|gb|ABC61503.1| (e_value=0.0) AGO1-2, p</td></tr>
<tr><td>NbS00001559g0021.1</td><td>NbS00001559g0021.1 protein AED:0.25 eAED:0.31 QI:138|0.5|0.53|1|0.85|0.93|15|300|837; (*GB) gi|356513635|ref|XP_003525517.1| (e_value=0.0) PREDIC</td></tr>
<tr><td>NbS00011860g0002.1</td><td>NbS00011860g0002.1 protein AED:0.14 eAED:0.14 QI:0|0.83|0.76|0.84|1|1|13|0|788; (*GB) gi|255561268|ref|XP_002521645.1| (e_value=0.0) arsenite-res</td></tr>
<tr><td>NbS00044448g0012.1</td><td>NbS00044448g0012.1 protein AED:0.24 eAED:0.25 QI:232|0.85|0.87|1|1|1|8|0|762; (*GB) gi|194396261|gb|ACF60500.1| (e_value=0.0) plastid transketola</td></tr>
<tr><td>NbS00020748g0007.1</td><td>NbS00020748g0007.1 protein AED:0.36 eAED:0.37 QI:0|0.64|0.66|0.86|0.85|0.8|15|0|555; (*GB) gi|587562|emb|CAA56520.1| (e_value=0.0) mitochondrial</td></tr>
<tr><td>NbS00019623g0001.1</td><td>NbS00019623g0001.1 protein AED:0.21 eAED:0.21 QI:0|1|0.5|1|1|1|2|0|447; (*GB) gi|3869088|dbj|BAA34348.1| (e_value=0.0) elongation factor-1 alpha</td></tr>
<tr><td>NbS00001988g0005.1</td><td>NbS00001988g0005.1 protein AED:0.30 eAED:0.30 QI:0|1|0.83|1|1|1|6|0|610; (*GB) gi|225441549|ref|XP_002281113.1| (e_value=0.0) PREDICTED: DEAD-box</td></tr>
<tr><td>NbS00000681g0006.1</td><td>NbS00000681g0006.1 protein AED:0.29 eAED:0.30 QI:89|0.75|0.88|1|0.75|0.77|9|282|301; (*GB) gi|358248282|ref|NP_001239854.1| (e_value=7e-62) uncha</td></tr>
<tr><td>NbS00035152g0024.1</td><td>NbS00035152g0024.1 protein AED:0.26 eAED:0.26 QI:0|1|0.92|1|0.69|0.78|14|405|631; (*GB) gi|359494535|ref|XP_002266085.2| (e_value=0.0) PREDICTED:</td></tr>
<tr><td>NbS00027428g0011.1</td><td>NbS00027428g0011.1 protein AED:0.20 eAED:0.20 QI:0|0|0|1|1|1|2|0|723; (*GB) gi|359475106|ref|XP_003631587.1| (e_value=0.0) PREDICTED: DEAD-box AT</td></tr>
<tr><td>NbS00015934g0007.1</td><td>NbS00015934g0007.1 protein AED:0.16 eAED:0.16 QI:0|0.66|0.28|1|0.5|0.57|7|0|612; (*GB) gi|255576310|ref|XP_002529048.1| (e_value=0.0) WRKY transc</td></tr>
<tr><td>NbS00004739g0001.1</td><td>NbS00004739g0001.1 protein AED:0.17 eAED:0.17 QI:0|1|0.66|1|1|1|3|0|251; (*GB) gi|100801744|emb|CAK24966.1| (e_value=2e-175) chlorophyll a/b bind</td></tr>
<tr><td>NbS00011643g0015.1</td><td>NbS00011643g0015.1 protein AED:0.07 eAED:0.07 QI:151|1|1|1|0.8|0.83|6|432|88; (*GB) gi|356508935|ref|XP_003523208.1| (e_value=2e-56) PREDICTED: s</td></tr>
<tr><td>NbS00030061g0007.1</td><td>NbS00030061g0007.1 protein AED:0.13 eAED:0.13 QI:243|1|1|1|0.85|0.75|8|1441|373; (*GB) gi|77416977|gb|ABA81884.1| (e_value=7e-108) nuclear RNA bi</td></tr>
<tr><td>NbS00023006g0207.1</td><td>NbS00023006g0207.1 protein</td></tr>
<tr><td>NbS00001594g0015.1</td><td>NbS00001594g0015.1 protein AED:0.04 eAED:0.04 QI:64|0.5|0.6|1|0.75|0.6|5|0|553; (*GB) gi|268619136|gb|ACZ13344.1| (e_value=0.0) tubulin alpha cha</td></tr>
<tr><td>NbS00001525g0121.1</td><td>NbS00001525g0121.1 protein AED:0.18 eAED:0.18 QI:3|0.62|0.22|1|0.75|0.77|9|0|561</td></tr>
<tr><td>>gi|74181742|dbj|BAE32582.1|</td><td>>gi|74181742|dbj|BAE32582.1| unnamed protein product [Mus musculus]</td></tr>
<tr><td>NbS00009714g0011.1</td><td>NbS00009714g0011.1 protein AED:0.20 eAED:0.20 QI:131|0.83|0.71|1|0.83|0.57|7|463|410; (*SWP) sp|Q40565|RCA2_TOBAC (e_value=0.0) Ribulose bisphosp</td></tr>
<tr><td>NbS00010545g0303.1</td><td>NbS00010545g0303.1 protein AED:0.34 eAED:0.34 QI:0|1|0.5|1|1|1|2|0|169</td></tr>
<tr><td>NbS00020769g0006.1</td><td>NbS00020769g0006.1 protein AED:0.25 eAED:0.25 QI:307|0.66|1|1|0.66|0.75|4|448|250; (*GB) gi|84620804|gb|ABC59516.1| (e_value=5e-102) chloroplast</td></tr>
<tr><td>NbS00004213g0104.1</td><td>NbS00004213g0104.1 protein AED:0.32 eAED:0.32 QI:0|-1|0|1|-1|1|1|0|132</td></tr>
<tr><td>NbS00005183g0002.1</td><td>NbS00005183g0002.1 protein AED:0.18 eAED:0.18 QI:0|0.69|0.57|0.92|1|1|14|0|607; (*GB) gi|1762130|gb|AAB39827.1| (e_value=0.0) chaperonin-60 beta</td></tr>
<tr><td>NbS00001859g0006.1</td><td>NbS00001859g0006.1 protein AED:0.10 eAED:0.10 QI:0|0.42|0.5|1|0.85|0.75|8|0|558; (*GB) gi|313585890|gb|ADR71054.1| (e_value=0.0) phosphoglycerate</td></tr>
<tr><td>NbS00010454g0009.1</td><td>NbS00010454g0009.1 protein AED:0.20 eAED:0.20 QI:0|1|0.5|1|1|1|2|0|333; (*SWP) sp|Q40459|PSBO_TOBAC (e_value=0.0) Oxygen-evolving enhancer protei</td></tr>
<tr><td>NbS00000529g0002.1</td><td>NbS00000529g0002.1 protein AED:0.44 eAED:0.48 QI:0|0.66|0.5|1|1|1|4|0|104; (*GB) gi|224068340|ref|XP_002302713.1| (e_value=7e-69) predicted prote</td></tr>
<tr><td>NbS00059497g0003.1</td><td>NbS00059497g0003.1 protein AED:0.17 eAED:0.17 QI:0|1|1|1|1|1|3|550|697; (*SWP) sp|Q653H7|ARFR_ORYSJ (e_value=0.0) Auxin response factor 18 OS=Ory</td></tr>
<tr><td>NbS00003380g0113.1</td><td>NbS00003380g0113.1 protein AED:0.20 eAED:0.20 QI:170|1|1|1|1|1|8|290|428</td></tr>
<tr><td>NbS00001538g0002.1</td><td>NbS00001538g0002.1 protein AED:0.19 eAED:0.19 QI:248|1|1|1|1|1|11|261|788; (*SWP) sp|Q7TP47|HNRPQ_RAT (e_value=2e-31) Heterogeneous nuclear ribon</td></tr>
<tr><td>NbS00007843g0002.1</td><td>NbS00007843g0002.1 protein AED:0.14 eAED:0.14 QI:0|0.76|0.78|1|0.84|0.85|14|2042|813; (*GB) gi|359489218|ref|XP_002270340.2| (e_value=0.0) PREDIC</td></tr>
<tr><td>NbS00061216g0001.1</td><td>NbS00061216g0001.1 protein AED:0.17 eAED:0.17 QI:0|-1|0|1|-1|1|1|0|179; (*SWP) sp|P11670|PRB1_TOBAC (e_value=3e-109) Basic form of pathogenesis-r</td></tr>
<tr><td>NbS00008941g0022.1</td><td>NbS00008941g0022.1 protein AED:0.25 eAED:0.25 QI:0|0.5|0.42|1|0.5|0.71|7|0|326; (*GB) gi|350535032|ref|NP_001234428.1| (e_value=0.0) signal recog</td></tr>
<tr><td>NbS00010323g0010.1</td><td>NbS00010323g0010.1 protein AED:0.16 eAED:0.16 QI:709|0.5|0.2|1|0.75|0.6|5|0|200; (*GB) gi|298205243|emb|CBI17302.3| (e_value=2e-105) unnamed prot</td></tr>
<tr><td>NbS00014997g0006.1</td><td>NbS00014997g0006.1 protein AED:0.37 eAED:0.37 QI:179|0.8|1|1|0.6|0.66|6|185|195; (*GB) gi|4774163|dbj|BAA77508.1| (e_value=2e-95) F1-ATP synthase</td></tr>
<tr><td>NbS00022525g0113.1</td><td>NbS00022525g0113.1 protein AED:0.18 eAED:0.18 QI:189|0.87|0.77|1|0.75|0.66|9|554|335</td></tr>
<tr><td>NbS00000088g0014.1</td><td>NbS00000088g0014.1 protein AED:0.33 eAED:0.33 QI:288|0.94|0.9|1|1|1|20|0|617; (*GB) gi|350537129|ref|NP_001234281.1| (e_value=0.0) vacuolar H+-AT</td></tr>
<tr><td>NbS00002621g0212.1</td><td>NbS00002621g0212.1 protein AED:0.19 eAED:0.19 QI:122|0.8|1|1|1|1|6|306|255</td></tr>
<tr><td>NbS00013798g0018.1</td><td>NbS00013798g0018.1 protein AED:0.47 eAED:0.47 QI:0|0|0|1|0|0.5|2|0|68; (*GB) gi|55977763|sp|P00823.2|ATPA_TOBAC (e_value=7e-32) RecName: Full=ATP</td></tr>
<tr><td>NbS00003380g0115.1</td><td>NbS00003380g0115.1 protein AED:0.12 eAED:0.12 QI:355|0.66|0.85|1|0.33|0.42|7|134|238</td></tr>
<tr><td>NbS00001259g0009.1</td><td>NbS00001259g0009.1 protein AED:0.04 eAED:0.04 QI:128|0.75|0.77|1|0.75|0.88|9|0|318; (*GB) gi|357505379|ref|XP_003622978.1| (e_value=2e-55) Ribonu</td></tr>
<tr><td>NbS00043723g0002.1</td><td>NbS00043723g0002.1 protein AED:0.01 eAED:0.01 QI:646|0.5|0.66|1|0.5|0.66|3|282|323; (*GB) gi|85068594|gb|ABC69377.1| (e_value=0.0) CYP81B2v2 [Nic</td></tr>
<tr><td>NbS00025246g0002.1</td><td>NbS00025246g0002.1 protein AED:0.04 eAED:0.05 QI:170|1|0.5|1|0|0.5|2|0|447; (*GB) gi|147767800|emb|CAN60219.1| (e_value=1e-95) hypothetical prote</td></tr>
<tr><td>NbS00028092g0004.1</td><td>NbS00028092g0004.1 protein AED:0.36 eAED:0.36 QI:0|0.6|0.33|1|1|1|6|0|450; (*GB) gi|555655|gb|AAA50196.1| (e_value=6e-121) DNA-binding protein [N</td></tr>
<tr><td>NbS00011451g0001.1</td><td>NbS00011451g0001.1 protein AED:0.10 eAED:0.10 QI:0|0.6|0.33|1|1|1|6|0|925; (*GB) gi|357933581|dbj|BAL15057.1| (e_value=0.0) glutamate receptor 3.</td></tr>
<tr><td>NbS00000420g0008.1</td><td>NbS00000420g0008.1 protein AED:0.16 eAED:0.16 QI:0|0.66|0.75|1|1|1|4|509|413; (*GB) gi|15223426|ref|NP_174029.1| (e_value=3e-82) uncharacterized</td></tr>
<tr><td>NbS00002298g0008.1</td><td>NbS00002298g0008.1 protein AED:0.21 eAED:0.21 QI:0|0.87|0.55|1|1|1|9|0|332; (*SWP) sp|P19446|MDHG_CITLA (e_value=0.0) Malate dehydrogenase, glyox</td></tr>
<tr><td>NbS00007897g0006.1</td><td>NbS00007897g0006.1 protein AED:0.13 eAED:0.13 QI:259|1|1|1|1|1|5|1352|252; (*GB) gi|51490665|emb|CAG26903.1| (e_value=4e-104) ALY protein [Nicoti</td></tr>
<tr><td>NbS00025302g0002.1</td><td>NbS00025302g0002.1 protein</td></tr>
<tr><td>NbS00005980g0002.1</td><td>NbS00005980g0002.1 protein AED:0.10 eAED:0.10 QI:0|0.4|0.33|1|1|1|6|0|548; (*GB) gi|350537917|ref|NP_001234829.1| (e_value=0.0) ascorbate oxidase</td></tr>
<tr><td>NbS00033391g0006.1</td><td>NbS00033391g0006.1 protein AED:0.14 eAED:0.14 QI:195|0.85|0.87|1|0.85|1|8|383|567; (*GB) gi|359479362|ref|XP_002277357.2| (e_value=0.0) PREDICTED</td></tr>
<tr><td>NbS00021832g0023.1</td><td>NbS00021832g0023.1 protein AED:0.35 eAED:0.35 QI:0|0|0|1|0|0|2|0|80; (*GB) gi|305671961|gb|ADM63350.1| (e_value=2e-44) photosystem II cp47 protei</td></tr>
<tr><td>NbS00033277g0006.1</td><td>NbS00033277g0006.1 protein AED:0.56 eAED:0.56 QI:0|0|0|0.16|1|1|6|0|118</td></tr>
<tr><td>NbS00025171g0003.1</td><td>NbS00025171g0003.1 protein AED:0.00 eAED:0.00 QI:54|1|1|1|1|1|3|6|221; (*GB) gi|3893824|gb|AAD03394.1| (e_value=4e-114) ATPase beta subunit [Nicc</td></tr>
<tr><td>NbS00005969g0002.1</td><td>NbS00005969g0002.1 protein AED:0.07 eAED:0.07 QI:0|0.5|0.55|0.88|1|1|9|177|561; (*GB) gi|225456270|ref|XP_002283518.1| (e_value=0.0) PREDICTED: n</td></tr>
<tr><td>NbS00000247g0008.1</td><td>NbS00000247g0008.1 protein AED:0.26 eAED:0.27 QI:16|0.85|0.73|1|1|1|15|0|662; (*GB) gi|416664|sp|Q03194.1|PMA4_NICPL (e_value=0.0) RecName: Full=</td></tr>
</table>

<table>
<tr><td></td><td></td></tr>
<tr><td>NbS00005125g0015.1</td><td>NbS00005125g0015.1 protein AED:0.27 eAED:0.27 QI:600|0.91|0.84|1|0.83|0.69|13|286|356; (*GB) gi|304368145|gb|ADM26718.1| (e_value=0.0) glycolate</td></tr>
<tr><td>NbS00042363g0004.1</td><td>NbS00042363g0004.1 protein AED:0.29 eAED:0.29 QI:95|1|1|1|0.25|0.2|5|264|361; (*SWP) sp|Q8LB81|GDL79_ARATH (e_value=0.0) GDSL esterase/lipase At5</td></tr>
<tr><td>NbS00003662g0021.1</td><td>NbS00003662g0021.1 protein AED:0.24 eAED:0.34 QI:0|0|0|1|0|0|3|0|257; (*GB) gi|78102516|ref|YP_358657.1| (e_value=6e-112) ATP synthase CF1 alpha</td></tr>
<tr><td>NbS00006781g0007.1</td><td>NbS00006781g0007.1 protein AED:0.08 eAED:0.08 QI:0|1|0.33|1|1|1|3|0|248; (*GB) gi|292653543|gb|ADE34289.1| (e_value=3e-125) aquaporin TIP2;3 [Gos</td></tr>
<tr><td>NbS00004222g0004.1</td><td>NbS00004222g0004.1 protein AED:0.16 eAED:0.16 QI:44|1|1|1|1|1|2|322|146; (*GB) gi|311893225|dbj|BAJ25784.1| (e_value=2e-97) putative PR-10 type p</td></tr>
<tr><td>NbS00000058g0018.1</td><td>NbS00000058g0018.1 protein AED:0.02 eAED:0.02 QI:0|1|0|1|1|1|2|0|221; (*GB) gi|30013659|gb|AAP03872.1| (e_value=2e-130) putative photosystem I su</td></tr>
<tr><td>NbS00009732g0022.1</td><td>NbS00009732g0022.1 protein AED:0.27 eAED:0.28 QI:90|0.9|0.90|1|0.9|0.81|11|248|411; (*GB) gi|50400860|sp|Q43497.1|MDAR_SOLLC (e_value=0.0) RecNam</td></tr>
<tr><td>NbS00028158g0015.1</td><td>NbS00028158g0015.1 protein AED:0.44 eAED:0.54 QI:0|0.75|0.4|1|1|1|5|0|169; (*GB) gi|388500696|gb|AFK38414.1| (e_value=1e-63) unknown [Lotus japon</td></tr>
<tr><td>NbS00013071g0001.1</td><td>NbS00013071g0001.1 protein AED:0.14 eAED:0.14 QI:0|0.66|0.25|1|1|1|4|0|360; (*SWP) sp|Q9XIV8|PERN1_TOBAC (e_value=0.0) Peroxidase N1 OS=Nicotiana</td></tr>
<tr><td>NbS00000172g0008.1</td><td>NbS00000172g0008.1 protein AED:0.09 eAED:0.09 QI:94|0.66|0.75|1|0.33|0|4|260|260; (*SWP) sp|P0C582|M2OM_NEUCR (e_value=7e-53) Putative mitochondr</td></tr>
<tr><td>NbS00005930g0008.1</td><td>NbS00005930g0008.1 protein AED:0.34 eAED:0.35 QI:0|0.5|0|1|0.5|0.66|3|0|299; (*GB) gi|359495241|ref|XP_003634942.1| (e_value=7e-112) PREDICTED: u</td></tr>
<tr><td>NbS00006448g0011.1</td><td>NbS00006448g0011.1 protein AED:0.25 eAED:0.25 QI:194|1|1|1|1|1|5|350|181; (*GB) gi|10799832|emb|CAC12883.1| (e_value=3e-116) ribosomal protein L1</td></tr>
<tr><td>NbS00015227g0002.1</td><td>NbS00015227g0002.1 protein AED:0.14 eAED:0.16 QI:0|-1|0|1|-1|1|1|0|171; (*GB) gi|237783971|gb|ACR19782.1| (e_value=5e-91) ATP synthase CF1 beta s</td></tr>
<tr><td>NbS00009537g0010.1</td><td>NbS00009537g0010.1 protein AED:0.14 eAED:0.14 QI:0|0.6|0.33|1|0.6|0.5|6|257|695; (*GB) gi|297738216|emb|CBI27417.3| (e_value=4e-139) unnamed prot</td></tr>
<tr><td>NbS00012621g0112.1</td><td>NbS00012621g0112.1 protein AED:0.39 eAED:0.39 QI:0|0.54|0.58|0.91|0.54|0.66|12|726|314</td></tr>
<tr><td>NbS00004792g0016.1</td><td>NbS00004792g0016.1 protein AED:0.26 eAED:0.27 QI:7|0.55|0.4|1|0.77|0.7|10|0|938; (*GB) gi|399213|sp|P31542.1|CLPAB_SOLLC (e_value=0.0) RecName: F</td></tr>
<tr><td>NbS00007742g0006.1</td><td>NbS00007742g0006.1 protein AED:0.40 eAED:0.42 QI:0|0.5|0.42|1|1|1|7|0|319; (*GB) gi|94466659|emb|CAJ44458.1| (e_value=2e-110) ALY protein [Nicoti</td></tr>
<tr><td>NbS00007661g0019.1</td><td>NbS00007661g0019.1 protein AED:0.18 eAED:0.18 QI:34|0.90|0.91|1|1|0.91|12|402|1023; (*GB) gi|22329468|ref|NP_172510.2| (e_value=0.0) Nucleoporin</td></tr>
<tr><td>NbS00017034g0014.1</td><td>NbS00017034g0014.1 protein AED:0.20 eAED:0.20 QI:0|0.8|0.83|0.83|0.8|0.83|6|26|160; (*GB) gi|225454123|ref|XP_002269748.1| (e_value=9e-100) PREDI</td></tr>
<tr><td>NbS00003269g0011.1</td><td>NbS00003269g0011.1 protein AED:0.29 eAED:0.29 QI:0|0.86|0.83|1|0.91|0.87|24|420|599; (*SWP) sp|Q42656|AGAL_COFAR (e_value=9e-149) Alpha-galactosi</td></tr>
<tr><td>NbS00001849g0017.1</td><td>NbS00001849g0017.1 protein AED:0.76 eAED:1.00 QI:0|0|0|0.66|0.5|0.66|3|0|72; (*GB) gi|94466657|emb|CAJ44457.1| (e_value=3e-26) ALY protein [Nicot</td></tr>
<tr><td>NbS00003461g0001.1</td><td>NbS00003461g0001.1 protein AED:0.23 eAED:0.30 QI:0|-1|0|1|-1|1|1|0|132; (*GB) gi|94466657|emb|CAJ44457.1| (e_value=9e-45) ALY protein [Nicotiana</td></tr>
<tr><td>NbS00010099g0014.1</td><td>NbS00010099g0014.1 protein AED:0.43 eAED:0.65 QI:0|0.5|0.33|1|0|0|3|29|57</td></tr>
<tr><td>NbS00009739g0005.1</td><td>NbS00009739g0005.1 protein AED:0.12 eAED:0.15 QI:183|0.78|0.93|1|0.85|0.8|15|362|489; (*GB) gi|359495798|ref|XP_002262872.2| (e_value=0.0) PREDIC</td></tr>
<tr><td>NbS00060216g0002.1</td><td>NbS00060216g0002.1 protein AED:0.22 eAED:0.42 QI:0|0.33|0.25|1|1|1|4|324|479; (*GB) gi|356556739|ref|XP_003546680.1| (e_value=9e-113) PREDICTED:</td></tr>
<tr><td>NbS00005099g0011.1</td><td>NbS00005099g0011.1 protein AED:0.26 eAED:0.26 QI:0|0.66|0.85|1|1|1|7|0|274; (*GB) gi|255552828|ref|XP_002517457.1| (e_value=2e-141) H(\+)-transpo</td></tr>
<tr><td>NbS00004507g0104.1</td><td>NbS00004507g0104.1 protein</td></tr>
<tr><td>NbS00000485g0008.1</td><td>NbS00000485g0008.1 protein AED:0.27 eAED:0.27 QI:81|1|1|1|0.8|0.83|6|329|238; (*GB) gi|225442156|ref|XP_002275541.1| (e_value=6e-155) PREDICTED:</td></tr>
<tr><td>NbS00009983g0008.1</td><td>NbS00009983g0008.1 protein AED:0.11 eAED:0.11 QI:0|0|0|1|0|0.5|2|0|384; (*GB) gi|108864705|gb|ABG22608.1| (e_value=0.0) Heat shock cognate 70 kDa</td></tr>
<tr><td>NbS00006644g0116.1</td><td>NbS00006644g0116.1 protein AED:0.00 eAED:0.00 QI:364|1|1|1|1|1|3|541|1093</td></tr>
<tr><td>NbS00011208g0006.1</td><td>NbS00011208g0006.1 protein AED:0.18 eAED:0.20 QI:0|0.81|0.83|1|1|1|12|47|443; (*GB) gi|225430786|ref|XP_002270129.1| (e_value=1e-97) PREDICTED: U</td></tr>
<tr><td>NbS00019490g0007.1</td><td>NbS00019490g0007.1 protein AED:0.32 eAED:0.34 QI:300|0.66|0.42|0.85|0.5|0.71|7|0|253; (*SWP) sp|O14327|PAB2_SCHPO (e_value=2e-38) Polyadenylate-b</td></tr>
<tr><td>NbS00003570g0008.1</td><td>NbS00003570g0008.1 protein AED:0.13 eAED:0.14 QI:0|0|0|0.6|0.5|0.6|5|0|237; (*GB) gi|40748265|gb|AAR89617.1| (e_value=1e-130) 40S ribosomal prote</td></tr>
<tr><td>NbS00004226g0004.1</td><td>NbS00004226g0004.1 protein ; (*GB) gi|94466657|emb|CAJ44457.1| (e_value=4e-18) ALY protein [Nicotiana benthamiana];; (*ITAG) Solyc10g086400.1.1</td></tr>
<tr><td>NbS00021398g0012.1</td><td>NbS00021398g0012.1 protein AED:0.33 eAED:0.35 QI:0|0.81|0.75|1|0.81|0.75|12|0|838; (*GB) gi|225450401|ref|XP_002278318.1| (e_value=0.0) PREDICTED</td></tr>
<tr><td>NbS00002113g0001.1</td><td>NbS00002113g0001.1 protein AED:0.00 eAED:0.05 QI:0|-1|0|1|-1|1|1|0|394; (*SWP) sp|Q00874|DR100_ARATH (e_value=1e-127) DNA-damage-repair/toleratic</td></tr>
<tr><td>NbS00005637g0007.1</td><td>NbS00005637g0007.1 protein AED:0.01 eAED:0.01 QI:0|1|0.5|1|1|1|2|0|892; (*GB) gi|15144509|gb|AAK84476.1| (e_value=0.0) unknown [Solanum lycopersi</td></tr>
<tr><td>NbS00006911g0001.1</td><td>NbS00006911g0001.1 protein AED:0.02 eAED:0.02 QI:286|0|0.5|1|0|0.5|2|0|422; (*GB) gi|347953950|gb|AEP33595.1| (e_value=0.0) CBL-interacting prote</td></tr>
<tr><td>NbS00018511g0004.1</td><td>NbS00018511g0004.1 protein AED:0.03 eAED:0.03 QI:152|1|1|1|0.25|0.2|5|139|338; (*GB) gi|29825611|gb|AAO92303.1| (e_value=0.0) gibberellin 2-oxida</td></tr>
<tr><td>NbS00001711g0013.1</td><td>NbS00001711g0013.1 protein AED:0.26 eAED:0.26 QI:422|0.85|0.87|1|0.85|0.75|8|401|334; (*TAIR) AT4G17520.1 (e_value=2e-31) | Symbols: | Hyalurona</td></tr>
<tr><td>NbS00010073g0013.1</td><td>NbS00010073g0013.1 protein AED:0.19 eAED:0.22 QI:0|0.66|0.75|1|0.33|0.25|4|0|220; (*SWP) sp|Q9LX82|MYB48_ARATH (e_value=5e-48) Transcription fact</td></tr>
<tr><td>NbS00013320g0008.1</td><td>NbS00013320g0008.1 protein AED:0.19 eAED:0.25 QI:82|0.66|0.69|1|0.75|0.76|13|0|616; (*GB) gi|297734868|emb|CBI17102.3| (e_value=5e-160) unnamed p</td></tr>
<tr><td>NbS00038102g0001.1</td><td>NbS00038102g0001.1 protein</td></tr>
<tr><td>NbS00018023g0003.1</td><td>NbS00018023g0003.1 protein AED:0.25 eAED:0.25 QI:0|1|0.75|1|1|1|4|510|435; (*GB) gi|297845476|ref|XP_002890619.1| (e_value=2e-95) hypothetical pr</td></tr>
<tr><td>NbS00001635g0020.1</td><td>NbS00001635g0020.1 protein AED:0.07 eAED:0.07 QI:0|0.4|0.33|1|0.8|0.5|6|0|371; (*SWP) sp|Q944G9|ALFC2_ARATH (e_value=0.0) Probable fructose-bisph</td></tr>
<tr><td>NbS00013764g0007.1</td><td>NbS00013764g0007.1 protein AED:0.11 eAED:0.11 QI:116|0.85|0.87|1|0.85|0.75|8|606|455; (*GB) gi|1705613|sp|P49319.2|CATA1_TOBAC (e_value=0.0) RecN</td></tr>
<tr><td>NbS00025410g0006.1</td><td>NbS00025410g0006.1 protein AED:0.12 eAED:0.12 QI:214|0.95|0.86|1|1|1|22|0|1037; (*GB) gi|255570428|ref|XP_002526173.1| (e_value=0.0) suppressor c</td></tr>
<tr><td>NbS00049664g0004.1</td><td>NbS00049664g0004.1 protein AED:0.29 eAED:0.29 QI:0|0.6|0.33|0.66|1|0.83|6|0|236; (*GB) gi|584795|sp|Q08436.1|PMA3_NICPL (e_value=1e-107) RecName:</td></tr>
<tr><td>NbS00002520g0008.1</td><td>NbS00002520g0008.1 protein AED:0.16 eAED:0.17 QI:128|1|0.75|1|1|1|4|0|340; (*GB) gi|384038823|gb|AFH58002.1| (e_value=0.0) chloroplast PsbP2 prec</td></tr>
<tr><td>NbS00002523g0003.1</td><td>NbS00002523g0003.1 protein AED:0.18 eAED:0.18 QI:0|-1|0|1|-1|1|1|0|704; (*GB) gi|75249421|sp|Q93YF5.1|SUVH1_TOBAC (e_value=0.0) RecName: Full=His</td></tr>
<tr><td>NbS00006116g0019.1</td><td>NbS00006116g0019.1 protein AED:0.27 eAED:0.27 QI:568|1|1|1|1|1|7|0|473; (*GB) gi|219560127|gb|ACL27272.1| (e_value=0.0) catalase [Nicotiana benth</td></tr>
<tr><td>NbS00008675g0003.1</td><td>NbS00008675g0003.1 protein AED:0.03 eAED:0.03 QI:0|0|0|1|1|1|2|0|492; (*GB) gi|75216452|sp|Q9ZS34.1|CHLP_TOBAC (e_value=0.0) RecName: Full=Gerany</td></tr>
<tr><td>NbS00007190g0001.1</td><td>NbS00007190g0001.1 protein AED:0.08 eAED:0.08 QI:179|1|1|1|0.5|0.33|3|443|275; (*GB) gi|225448932|ref|XP_002267178.1| (e_value=1e-93) PREDICTED:</td></tr>
<tr><td>NbS00000548g0008.1</td><td>NbS00000548g0008.1 protein AED:0.29 eAED:0.30 QI:0|0.8|0.5|1|1|0.66|6|0|363; (*GB) gi|255573386|ref|XP_002527619.1| (e_value=2e-130) Ras-GTPase-a</td></tr>
<tr><td>NbS00010498g0007.1</td><td>NbS00010498g0007.1 protein AED:0.12 eAED:0.12 QI:0|1|1|1|0.5|1|3|646|228; (*GB) gi|384038831|gb|AFH58006.1| (e_value=5e-128) chloroplast PsbQ2 pr</td></tr>
<tr><td>NbS00011355g0109.1</td><td>NbS00011355g0109.1 protein AED:0.07 eAED:0.07 QI:222|1|1|1|1|1|5|560|168</td></tr>
<tr><td>NbS00028210g0125.1</td><td>NbS00028210g0125.1 protein AED:0.13 eAED:0.14 QI:0|0|0|0.94|0.93|0.94|17|0|680</td></tr>
<tr><td>NbS00005651g0008.1</td><td>NbS00005651g0008.1 protein AED:0.24 eAED:0.24 QI:27|0.62|0.33|1|0.5|0.44|9|0|470; (*GB) gi|175363751|gb|ACB72462.1| (e_value=0.0) elongation fact</td></tr>
<tr><td>NbS00005673g0011.1</td><td>NbS00005673g0011.1 protein AED:0.19 eAED:0.19 QI:0|0.8|0.63|1|0.8|0.81|11|273|722; (*GB) gi|356547867|ref|XP_003542326.1| (e_value=0.0) PREDICTED</td></tr>
</table>

NbS00003717g0028.1	NbS00003717g0028.1 protein AED:0.30 eAED:0.30 QI:203|0.71|0.5|1|0.71|0.87|8|0|244; (*GB) gi|225438529|ref|XP_002279389.1| (e_value=6e-119) PREDIC
NbS00010860g0014.1	NbS00010860g0014.1 protein AED:0.05 eAED:0.05 QI:88|1|1|1|1|1|5|242|88; (*GB) gi|225468340|ref|XP_002272246.1| (e_value=5e-54) PREDICTED: probabl
NbS00022787g0008.1	NbS00022787g0008.1 protein AED:0.33 eAED:0.33 QI:0|1|0.85|1|1|1|7|0|270; (*GB) gi|21912927|emb|CAC84143.2| (e_value=0.0) thioredoxin peroxidase [
NbS00013023g0001.1	NbS00013023g0001.1 protein AED:0.21 eAED:0.21 QI:0|1|0.66|1|1|1|3|0|406; (*GB) gi|1707878|sp|P54260.1|GCST_SOLTU (e_value=0.0) RecName: Full=Amin
NbS00007101g0012.1	NbS00007101g0012.1 protein AED:0.11 eAED:0.11 QI:0|0.85|0.8|1|1|1|15|492|1014; (*GB) gi|3334200|sp|O49954.1|GCSP_SOLTU (e_value=0.0) RecName: Ful
NbS00000710g0004.1	NbS00000710g0004.1 protein AED:0.10 eAED:0.12 QI:195|0.77|0.7|1|0.77|0.5|10|0|501; (*SWP) sp|O94260|G3BP_SCHPO (e_value=3e-21) Putative G3BP-like
NbS00004213g0009.1	NbS00004213g0009.1 protein AED:0.11 eAED:0.11 QI:0|1|0.83|1|1|0.83|6|0|298; (*GB) gi|19913109|emb|CAC84547.1| (e_value=0.0) dicarboxylate/tricarb
NbS00028915g0014.1	NbS00028915g0014.1 protein AED:0.24 eAED:0.24 QI:142|0|0.33|1|0|0.33|3|0|178; (*SWP) sp|Q9SUI4|PSAL_ARATH (e_value=7e-75) Photosystem I reaction

Select Summary Report

Format As | Select Summary (protein hits) | Help

Significance threshold p< 0.05 Max. number of hits AUTO

Standard scoring ○ MudPIT scoring ◉ Ions score or expect cut-off 30 Show sub-sets 0

Show pop-ups ◉ Suppress pop-ups ○ Require bold red ☐

Re-Search ◉ All queries ○ Unassigned ○ Below homology threshold ○ Below identity threshold

1. gi|297262447|ref|XP_001098182.2| **Mass:** 65341 **Score:** 296 **Matches:** 15(12) **Sequences:** 12(11) **emPAI:** 0.80
gi|297262447|ref|XP_001098182.2| PREDICTED: keratin, type II cytoskeletal 1-like isoform 6 [Macaca mulatta]

Query	Observed	Mr(expt)	Mr(calc)	ppm	Miss	Score	Expect	Rank	Unique	Peptide
11242	437.7532	873.4919	873.4920	-0.06	0	37	0.017	1	U	R.SLVNLGGSK.S
12423	517.2617	1032.5089	1032.5087	0.15	0	30	0.084	1	U	R.TLLEGEESR.M 12422
13085	563.2755	1124.5364	1124.5349	1.27	0	31	0.046	1	U	K.AEAESLYQSK.Y
13203	571.2700	1140.5254	1140.5121	11.7	0	40	0.0055	1	U	R.DYQELMNTK.L
13505	590.3036	1178.5926	1178.5931	-0.42	0	64	3.3e-005	1	U	K.YEELQITAGR.H 13506
14128	633.3220	1264.6294	1264.6299	-0.43	0	39	0.0093	1	U	R.TNAENEFVTIK.K
14477	651.8546	1301.6946	1301.6939	0.52	1	31	0.055	1	U	K.NSKIEISELNR.V
14480	651.8613	1301.7081	1301.7078	0.21	0	66	1.6e-005	1	U	R.SLDLDSIIAEVK.A 14483
15104	692.3494	1382.6843	1382.6830	0.93	0	65	2e-005	1	U	K.SLNNQFASFIDK.V
15169	465.2489	1392.7249	1392.7249	0.00	1	34	0.026	1	U	R.TNAENEFVTIKK.D
15744	738.3786	1474.7427	1474.7416	0.71	0	72	4.5e-006	1	U	K.WELLQQVDTSTR.T
15747	738.3964	1474.7782	1474.7780	0.13	0	71	5.7e-006	1		R.FLEQQNQVLQTK.W

2. gi|28317|emb|CAA32649.1| **Mass:** 59720 **Score:** 283 **Matches:** 12(12) **Sequences:** 10(10) **emPAI:** 0.71
gi|28317|emb|CAA32649.1| unnamed protein product [Homo sapiens]

Query	Observed	Mr(expt)	Mr(calc)	ppm	Miss	Score	Expect	Rank	Unique	Peptide
10769	404.2034	806.3922	806.3923	-0.01	0	43	0.005	1	U	R.LAADDFR.L
12396	516.3035	1030.5925	1030.5910	1.44	0	35	0.017	1	U	R.VLDELTLTK.A
12840	545.7692	1089.5239	1089.5237	0.20	0	38	0.011	1	U	K.VTMQNLNDR.L 12843
12977	555.2487	1108.4829	1108.4825	0.33	0	59	5.2e-005	1	U	K.DAEAWFNEK.S
14099	631.8024	1261.5902	1261.5899	0.26	0	80	6.4e-007	1	U	R.SLLEGEGSSGGGGR.G
14919	453.2444	1356.7113	1356.7110	0.28	1	41	0.0048	1	U	R.QSVEADINGLRR.V
15086	691.3282	1380.6418	1380.6408	0.71	0	47	0.0011	1	U	R.ALEESNYELEGK.I 15088
15148	695.8451	1389.6756	1389.6736	1.50	0	73	3.4e-006	1	U	K.QSLEASLAETEGR.Y
15468	717.8903	1433.7660	1433.7626	2.32	1	37	0.012	1	U	K.IRLENEIQTYR.S
20859	1018.2161	3051.6265	3051.6200	2.15	1	35	0.0063	1	U	K.TIDDLKNQILNLTTDNANILLQIDNAR.L

3. gi|291410763|ref|XP_002721657.1| **Mass:** 27347 **Score:** 209 **Matches:** 10(10) **Sequences:** 5(5) **emPAI:** 1.00
gi|291410763|ref|XP_002721657.1| PREDICTED: histone cluster 1, H2ag-like [Oryctolagus cuniculus]

Query	Observed	Mr(expt)	Mr(calc)	ppm	Miss	Score	Expect	Rank	Unique	Peptide
11726	472.7693	943.5240	943.5240	0.07	0	51	0.00072	1		R.AGLQFPVGR.V 11722 11727
13512	590.8134	1179.6123	1179.6135	-1.06	0	67	1.6e-005	1		R.ISGLIYEETR.G 13515

Query	Observed	Mr(expt)	Mr(calc)	ppm	Miss	Score	Expect	Rank	Unique	Peptide
14558	655.8553	1309.6960	1309.6952	0.65	0	32	0.039	1		K.TVTAMDVVYALK.R
14691	442.5894	1324.7462	1324.7463	-0.03	0	(34)	0.015	1		R.DNIQGITKPAIR.R
14694	663.3809	1324.7473	1324.7463	0.76	0	48	0.00061	1		R.DNIQGITKPAIR.R 14695
15687	733.9051	1465.7956	1465.7963	-0.44	1	45	0.0017	1		K.TVTAMDVVYALKR.Q

---

4. NbS00000666g0001.1 **Mass:** 15719 **Score:** 208 **Matches:** 10(9) **Sequences:** 5(5) **emPAI:** 2.91
NbS00000666g0001.1 protein AED:0.15 eAED:0.15 QI:0|-1|0|1|-1|1|1|0|145; (*GB) gi|82400146|gb|ABB72812.1| (e_value=5e-61) histone H2B-like protein [Solanum tuberosum];; (*SWP) sp|Q1S9I9|H2B1_MEDTR

Query	Observed	Mr(expt)	Mr(calc)	ppm	Miss	Score	Expect	Rank	Unique	Peptide
10092	342.2097	682.4049	682.4054	-0.64	0	31	0.019	1	U	K.IYIFK.V
10976	414.7140	827.4134	827.4137	-0.36	0	38	0.0067	1	U	K.HAVSEGTK.A
11691	470.2967	938.5788	938.5800	-1.36	0	37	0.0037	1	U	R.LVLPGELAK.H
13527	394.2156	1179.6250	1179.6248	0.18	0	42	0.0045	1	U	K.QVHPDIGISSK.A 13520
17992	865.4194	1728.8243	1728.8215	1.64	0	68	8.3e-006	1	U	K.AMGIMNSFINDIFEK.L
18098	873.4163	1744.8181	1744.8164	0.97	0	(44)	0.0021	1	U	K.AMGIMNSFINDIFEK.L 18104
18193	881.4138	1760.8130	1760.8113	0.93	0	(55)	0.00018	1	U	K.AMGIMNSFINDIFEK.L 18191

Proteins matching the same set of peptides:
NbS00000678g0001.1 **Mass:** 15849 **Score:** 208 **Matches:** 10(9) **Sequences:** 5(5)
NbS00000678g0001.1 protein AED:0.22 eAED:0.22 QI:0|-1|0|1|-1|1|1|0|147; (*GB) gi|82400146|gb|ABB72812.1| (e_value=5e-61) histone H2B-like protein [Solanum tuberosum];; (*SWP) sp|Q1S9I9|H2B1_MEDTR
NbS00000678g0002.1 **Mass:** 15863 **Score:** 208 **Matches:** 10(9) **Sequences:** 5(5)
NbS00000678g0002.1 protein AED:0.22 eAED:0.22 QI:0|-1|0|1|-1|1|1|0|147; (*GB) gi|82400146|gb|ABB72812.1| (e_value=5e-61) histone H2B-like protein [Solanum tuberosum];; (*SWP) sp|Q1S9I9|H2B1_MEDTR
NbS00000678g0009.1 **Mass:** 16874 **Score:** 208 **Matches:** 10(9) **Sequences:** 5(5)
NbS00000678g0009.1 protein AED:0.29 eAED:0.29 QI:0|0|0|0.5|1|1|2|0|156; (*GB) gi|388514209|gb|AFK45166.1| (e_value=1e-55) unknown [Lotus japonicus];; (*SWP) sp|Q1SU99|H2B3_MEDTR (e_value=5e-57) Pro
NbS00001050g0001.1 **Mass:** 16027 **Score:** 208 **Matches:** 10(9) **Sequences:** 5(5)
NbS00001050g0001.1 protein AED:0.13 eAED:0.14 QI:0|-1|0|1|-1|1|1|0|147; (*GB) gi|388522051|gb|AFK49087.1| (e_value=3e-60) unknown [Lotus japonicus];; (*SWP) sp|P40283|H2B11_ARATH (e_value=2e-61) H
NbS00006411g0002.1 **Mass:** 15863 **Score:** 208 **Matches:** 10(9) **Sequences:** 5(5)
NbS00006411g0002.1 protein AED:0.22 eAED:0.22 QI:0|-1|0|1|-1|1|1|0|147; (*GB) gi|82400146|gb|ABB72812.1| (e_value=5e-61) histone H2B-like protein [Solanum tuberosum];; (*SWP) sp|Q1S9I9|H2B1_MEDTR
NbS00007991g0006.1 **Mass:** 17158 **Score:** 208 **Matches:** 10(9) **Sequences:** 5(5)
NbS00007991g0006.1 protein AED:0.08 eAED:0.08 QI:0|0|0|1|1|1|2|0|157; (*GB) gi|146336941|gb|ABQ23584.1| (e_value=2e-57) putative histone [Medicago truncatula];; (*SWP) sp|Q1S9I9|H2B1_MEDTR (e_value
NbS00008452g0101.1 **Mass:** 15935 **Score:** 208 **Matches:** 10(9) **Sequences:** 5(5)
NbS00008452g0101.1 protein AED:0.16 eAED:0.16 QI:0|1|0.5|1|0|0|2|347|146
NbS00017233g0003.1 **Mass:** 16025 **Score:** 208 **Matches:** 10(9) **Sequences:** 5(5)
NbS00017233g0003.1 protein AED:0.20 eAED:0.20 QI:0|-1|0|1|-1|1|1|0|146; (*SWP) sp|P93354|H2B_TOBAC (e_value=4e-62) Histone H2B OS=Nicotiana tabacum GN=HIS2B PE=2 SV=3;; (*TAIR) AT5G59910.1 (e_value
NbS00020161g0001.1 **Mass:** 16125 **Score:** 208 **Matches:** 10(9) **Sequences:** 5(5)
NbS00020161g0001.1 protein AED:0.20 eAED:0.20 QI:0|-1|0|1|-1|1|1|0|147; (*GB) gi|82400146|gb|ABB72812.1| (e_value=3e-60) histone H2B-like protein [Solanum tuberosum];; (*SWP) sp|Q1S9I9|H2B1_MEDTR
NbS00023525g0004.1 **Mass:** 15039 **Score:** 208 **Matches:** 10(9) **Sequences:** 5(5)
NbS00023525g0004.1 protein AED:0.27 eAED:0.28 QI:0|0|0|0.5|1|1|2|0|137; (*GB) gi|356576357|ref|XP_003556299.1| (e_value=4e-60) PREDICTED: probable histone H2B.1-like [Glycine max];; (*SWP) sp|Q1S9I
NbS00027179g0002.1 **Mass:** 15949 **Score:** 208 **Matches:** 10(9) **Sequences:** 5(5)
NbS00027179g0002.1 protein AED:0.27 eAED:0.27 QI:0|-1|0|1|-1|1|1|0|146; (*GB) gi|388522051|gb|AFK49087.1| (e_value=1e-60) unknown [Lotus japonicus];; (*SWP) sp|Q1S9I9|H2B1_MEDTR (e_value=6e-62) Pro
NbS00028800g0003.1 **Mass:** 16210 **Score:** 208 **Matches:** 10(9) **Sequences:** 5(5)
NbS00028800g0003.1 protein AED:0.16 eAED:0.16 QI:0|-1|0|1|-1|1|1|0|148; (*GB) gi|118484226|gb|ABK93993.1| (e_value=3e-60) unknown [Populus trichocarpa];; (*SWP) sp|Q1S9I9|H2B1_MEDTR (e_value=1e-61)
NbS00032978g0007.1 **Mass:** 15457 **Score:** 208 **Matches:** 10(9) **Sequences:** 5(5)
NbS00032978g0007.1 protein AED:0.01 eAED:0.01 QI:0|-1|0|1|-1|1|1|0|141; (*GB) gi|82400146|gb|ABB72812.1| (e_value=2e-60) histone H2B-like protein [Solanum tuberosum];; (*SWP) sp|Q1S9I9|H2B1_MEDTR
NbS00037466g0010.1 **Mass:** 20264 **Score:** 208 **Matches:** 10(9) **Sequences:** 5(5)
NbS00037466g0010.1 protein AED:0.02 eAED:0.03 QI:0|0|0|1|1|1|2|0|185; (*GB) gi|388514209|gb|AFK45166.1| (e_value=3e-60) unknown [Lotus japonicus];; (*SWP) sp|Q1S9I9|H2B1_MEDTR (e_value=2e-61) Proba
NbS00046472g0002.1 **Mass:** 15732 **Score:** 208 **Matches:** 10(9) **Sequences:** 5(5)
NbS00046472g0002.1 protein AED:0.00 eAED:0.00 QI:0|-1|0|1|-1|1|1|0|143; (*GB) gi|225435052|ref|XP_002284344.1| (e_value=3e-64) PREDICTED: probable histone H2B.1-like [Vitis vinifera];; (*SWP) sp|O2
NbC24219583g0001.1 **Mass:** 11803 **Score:** 208 **Matches:** 10(9) **Sequences:** 5(5)
NbC24219583g0001.1 protein AED:0.00 eAED:0.00 QI:0|-1|0|1|-1|1|1|0|107; (*GB) gi|82400146|gb|ABB72812.1| (e_value=1e-61) histone H2B-like protein [Solanum tuberosum];; (*SWP) sp|Q1S9I9|H2B1_MEDTR
NbC25892755g0001.1 **Mass:** 15725 **Score:** 208 **Matches:** 10(9) **Sequences:** 5(5)
NbC25892755g0001.1 protein AED:0.00 eAED:0.00 QI:0|-1|0|1|-1|1|1|0|143; (*GB) gi|224065198|ref|XP_002301712.1| (e_value=3e-75) histone 2 [Populus trichocarpa];; (*SWP) sp|Q1S9I9|H2B1_MEDTR (e_value

---

5. NbS00004898g0002.1 **Mass:** 11402 **Score:** 192 **Matches:** 9(9) **Sequences:** 5(5) **emPAI:** 3.85
NbS00004898g0002.1 protein AED:0.00 eAED:0.00 QI:0|-1|0|1|-1|1|1|0|103; (*GB) gi|195617694|gb|ACG30677.1| (e_value=1e-51) histone H4 [Zea mays];; (*SWP) sp|P0CG89|H4_SOYBN (e_value=1e-52) Histone H

Query	Observed	Mr(expt)	Mr(calc)	ppm	Miss	Score	Expect	Rank	Unique	Peptide
12181	502.2997	1002.5849	1002.5862	-1.26	0	48	0.00073	1	U	K.IFLENVIR.D 12182

13512	590.8134	1179.6123	1179.6135	-1.06	0	67	1.6e-005	1		R.ISGLIYEETR.G 13515
14558	655.8553	1309.6960	1309.6952	0.65	0	32	0.039	1		K.TVTAMDVVYALK.R
14691	442.5894	1324.7462	1324.7463	-0.03	0	(34)	0.015	1		R.DNIQGITKPAIR.R
14694	663.3809	1324.7473	1324.7463	0.76	0	48	0.00061	1		R.DNIQGITKPAIR.R 14695
15687	733.9051	1465.7956	1465.7963	-0.44	1	45	0.0017	1		K.TVTAMDVVYALKR.Q

Proteins matching the same set of peptides:

NbS00007760g0001.1 **Mass:** 11402 **Score:** 192 **Matches:** 9(9) **Sequences:** 5(5)
NbS00007760g0001.1 protein AED:0.00 eAED:0.00 QI:286|1|0.5|1|0|0|2|0|103; (*GB) gi|195617694|gb|ACG30677.1| (e_value=1e-51) histone H4 [Zea mays];; (*SWP) sp|P0CG89|H4_SOYBN (e_value=1e-52) Histone
NbS00009197g0102.1 **Mass:** 11420 **Score:** 192 **Matches:** 9(9) **Sequences:** 5(5)
NbS00009197g0102.1 protein AED:0.26 eAED:0.26 QI:0|-1|0|1|-1|1|1|0|103
NbS00010526g0004.1 **Mass:** 11402 **Score:** 192 **Matches:** 9(9) **Sequences:** 5(5)
NbS00010526g0004.1 protein AED:0.35 eAED:0.35 QI:0|-1|0|1|-1|1|1|0|103; (*GB) gi|195617694|gb|ACG30677.1| (e_value=1e-51) histone H4 [Zea mays];; (*SWP) sp|P0CG89|H4_SOYBN (e_value=1e-52) Histone H
NbS00013276g0001.1 **Mass:** 11402 **Score:** 192 **Matches:** 9(9) **Sequences:** 5(5)
NbS00013276g0001.1 protein AED:0.23 eAED:0.23 QI:0|-1|0|1|-1|1|1|0|103; (*SWP) sp|P0CG89|H4_SOYBN (e_value=1e-52) Histone H4 OS=Glycine max PE=3 SV=1;; (*TAIR) AT5G59970.1 (e_value=9e-54) | Symbols
NbS00033043g0001.1 **Mass:** 11402 **Score:** 192 **Matches:** 9(9) **Sequences:** 5(5)
NbS00033043g0001.1 protein AED:0.23 eAED:0.23 QI:0|-1|0|1|-1|1|1|0|103; (*GB) gi|195617694|gb|ACG30677.1| (e_value=1e-51) histone H4 [Zea mays];; (*SWP) sp|P0CG89|H4_SOYBN (e_value=1e-52) Histone H
NbS00034995g0002.1 **Mass:** 11748 **Score:** 192 **Matches:** 9(9) **Sequences:** 5(5)
NbS00034995g0002.1 protein AED:0.23 eAED:0.23 QI:37|0|0|0.5|1|1|2|0|106; (*GB) gi|308799807|ref|XP_003074685.1| (e_value=1e-50) Histone H4 (ISS) [Ostreococcus tauri];; (*SWP) sp|P0CG89|H4_SOYBN (e_
NbS00037466g0001.1 **Mass:** 11402 **Score:** 192 **Matches:** 9(9) **Sequences:** 5(5)
NbS00037466g0001.1 protein AED:0.28 eAED:0.28 QI:0|-1|0|1|-1|1|1|0|103; (*GB) gi|195617694|gb|ACG30677.1| (e_value=1e-51) histone H4 [Zea mays];; (*SWP) sp|P0CG89|H4_SOYBN (e_value=1e-52) Histone H
NbS00045876g0001.1 **Mass:** 11402 **Score:** 192 **Matches:** 9(9) **Sequences:** 5(5)
NbS00045876g0001.1 protein AED:0.00 eAED:0.00 QI:0|-1|0|1|-1|1|1|0|103; (*GB) gi|195617694|gb|ACG30677.1| (e_value=1e-51) histone H4 [Zea mays];; (*SWP) sp|P0CG89|H4_SOYBN (e_value=1e-52) Histone H
NbS00048006g0001.1 **Mass:** 11402 **Score:** 192 **Matches:** 9(9) **Sequences:** 5(5)
NbS00048006g0001.1 protein AED:0.09 eAED:0.10 QI:282|0|0.5|1|0|0.5|2|0|103; (*GB) gi|195617694|gb|ACG30677.1| (e_value=1e-51) histone H4 [Zea mays];; (*SWP) sp|P0CG89|H4_SOYBN (e_value=1e-52) Histo
NbS00056894g0001.1 **Mass:** 11402 **Score:** 192 **Matches:** 9(9) **Sequences:** 5(5)
NbS00056894g0001.1 protein AED:0.20 eAED:0.20 QI:0|-1|0|1|-1|1|1|0|103; (*SWP) sp|P0CG89|H4_SOYBN (e_value=1e-52) Histone H4 OS=Glycine max PE=3 SV=1;; (*TAIR) AT5G59970.1 (e_value=9e-54) | Symbols
NbC25918569g0001.1 **Mass:** 11402 **Score:** 192 **Matches:** 9(9) **Sequences:** 5(5)
NbC25918569g0001.1 protein AED:0.00 eAED:0.00 QI:0|-1|0|1|-1|1|1|0|103; (*GB) gi|195617694|gb|ACG30677.1| (e_value=1e-51) histone H4 [Zea mays];; (*SWP) sp|P0CG89|H4_SOYBN (e_value=1e-52) Histone H
NbC25948449g0001.1 **Mass:** 11402 **Score:** 192 **Matches:** 9(9) **Sequences:** 5(5)
NbC25948449g0001.1 protein AED:0.24 eAED:0.24 QI:0|-1|0|1|-1|1|1|0|103; (*GB) gi|195617694|gb|ACG30677.1| (e_value=1e-51) histone H4 [Zea mays];; (*SWP) sp|P0CG89|H4_SOYBN (e_value=1e-52) Histone H
NbS00001548g0007.1 **Mass:** 22455 **Score:** 192 **Matches:** 9(9) **Sequences:** 5(5)
NbS00001548g0007.1 protein AED:0.25 eAED:0.25 QI:0|0|0|1|0|0.5|2|0|202; (*SWP) sp|P0CG89|H4_SOYBN (e_value=2e-50) Histone H4 OS=Glycine max PE=3 SV=1;; (*TAIR) AT5G59970.1 (e_value=2e-51) | Symbols
NbS00046909g0009.1 **Mass:** 26576 **Score:** 192 **Matches:** 9(9) **Sequences:** 5(5)
NbS00046909g0009.1 protein AED:0.12 eAED:0.15 QI:52|0|0|1|0|0.33|3|151|237; (*GB) gi|195617694|gb|ACG30677.1| (e_value=4e-50) histone H4 [Zea mays];; (*SWP) sp|P0CG89|H4_SOYBN (e_value=8e-51) Histo

---

6. NbS00017066g0003.1 **Mass:** 21609 **Score:** 175 **Matches:** 5(5) **Sequences:** 2(2) **emPAI:** 0.54
NbS00017066g0003.1 protein AED:0.01 eAED:0.01 QI:0|-1|0|1|-1|1|1|0|208; (*GB) gi|222051768|dbj|BAH15357.1| (e_value=3e-127) germin like protein [Nicotiana tabacum];; (*SWP) sp|Q9ZRA4|AB19A_PRUPE (e

Query	Observed	Mr(expt)	Mr(calc)	ppm	Miss	Score	Expect	Rank	Unique	Peptide
16196	**775.9196**	**1549.8247**	**1549.8239**	**0.49**	**0**	**71**	**4.3e-006**	**1**	**U**	**K.LVEATTFLDEATIK.K** 16198
20163	**1100.5985**	**2199.1825**	**2199.1800**	**1.11**	**0**	**(51)**	**0.00026**	**1**	**U**	**K.AAVTPAFAAQFPGLNGLGLSAAR.L** 20162
20164	**734.0681**	**2199.1825**	**2199.1800**	**1.13**	**0**	**55**	**0.00011**	**1**	**U**	**K.AAVTPAFAAQFPGLNGLGLSAAR.L**

Proteins matching the same set of peptides:

NbS00021913g0003.1 **Mass:** 21582 **Score:** 175 **Matches:** 5(5) **Sequences:** 2(2)
NbS00021913g0003.1 protein AED:0.01 eAED:0.01 QI:0|-1|0|1|-1|1|1|0|208; (*GB) gi|222051768|dbj|BAH15357.1| (e_value=6e-127) germin like protein [Nicotiana tabacum];; (*SWP) sp|Q9ZRA4|AB19A_PRUPE (e

---

7. gi|148727309|ref|NP_001092039.1| **Mass:** 66230 **Score:** 160 **Matches:** 6(6) **Sequences:** 5(5) **emPAI:** 0.27
gi|148727309|ref|NP_001092039.1| keratin, type II cytoskeletal 2 epidermal [Pan troglodytes]

Query	Observed	Mr(expt)	Mr(calc)	ppm	Miss	Score	Expect	Rank	Unique	Peptide
10999	**416.2510**	**830.4875**	**830.4862**	**1.55**	**0**	**33**	**0.038**	**1**	**U**	**R.SLVGLGGTK.S**
12780	**541.8034**	**1081.5923**	**1081.5920**	**0.22**	**1**	**39**	**0.0065**	**1**	**U**	**K.FASFIDKVR.F**
12965	**554.2756**	**1106.5366**	**1106.5356**	**0.90**	**0**	**67**	**1.6e-005**	**1**	**U**	**K.AQYEEIAQR.S** 12963
14029	**627.8080**	**1253.6015**	**1253.6001**	**1.18**	**0**	**41**	**0.0053**	**1**	**U**	**R.GFSSGSAVVSGGSR.R**
15747	738.3964	1474.7782	1474.7780	0.13	0	71	5.7e-006	1		R.FLEQQNQVLQTK.W

---

8. NbS00000987g0004.1 **Mass:** 28363 **Score:** 151 **Matches:** 6(6) **Sequences:** 3(3) **emPAI:** 0.56
NbS00000987g0004.1 protein AED:0.21 eAED:0.21 QI:0|-1|0|1|-1|1|1|0|266; (*GB) gi|3036948|dbj|BAA25392.1| (e_value=0.0) light harvesting chlorophyll a/b-binding protein [Nicotiana sylvestris];; (*SW

Query	Observed	Mr(expt)	Mr(calc)	ppm	Miss	Score	Expect	Rank	Unique	Peptide
12031	492.2531	982.4916	982.4913	0.36	0	57	0.00013	1		K.FGEAVWFK.A 12029 12030
12574	528.2689	1054.5233	1054.5229	0.32	0	34	0.025	1		R.ELEVIHCR.W
16750	535.2668	1602.7785	1602.7791	-0.33	0	(37)	0.012	1	U	K.AKPVSSGSPWYGPDR.V
16752	802.3973	1602.7801	1602.7791	0.67	0	52	0.00041	1	U	K.AKPVSSGSPWYGPDR.V

Proteins matching the same set of peptides:
NbS00002523g0102.1 **Mass:** 28567 **Score:** 151 **Matches:** 6(6) **Sequences:** 3(3)
NbS00002523g0102.1 protein AED:0.30 eAED:0.30 QI:0|-1|0|1|-1|1|1|0|267
NbS00002523g0104.1 **Mass:** 28492 **Score:** 151 **Matches:** 6(6) **Sequences:** 3(3)
NbS00002523g0104.1 protein AED:0.30 eAED:0.30 QI:0|-1|0|1|-1|1|1|0|267
NbS00003625g0001.1 **Mass:** 28598 **Score:** 151 **Matches:** 6(6) **Sequences:** 3(3)
NbS00003625g0001.1 protein AED:0.17 eAED:0.17 QI:0|-1|0|1|-1|1|1|0|267; (*GB) gi|3036955|dbj|BAA25396.1| (e_value=0.0) light harvesting chlorophyll a/b-binding protein [Nicotiana sylvestris];; (*SW
NbS00003625g0003.1 **Mass:** 28448 **Score:** 151 **Matches:** 6(6) **Sequences:** 3(3)
NbS00003625g0003.1 protein AED:0.19 eAED:0.19 QI:0|-1|0|1|-1|1|1|0|267; (*GB) gi|3036955|dbj|BAA25396.1| (e_value=0.0) light harvesting chlorophyll a/b-binding protein [Nicotiana sylvestris];; (*SW
NbS00006290g0001.1 **Mass:** 28564 **Score:** 151 **Matches:** 6(6) **Sequences:** 3(3)
NbS00006290g0001.1 protein AED:0.21 eAED:0.21 QI:0|-1|0|1|-1|1|1|0|267; (*GB) gi|3036955|dbj|BAA25396.1| (e_value=0.0) light harvesting chlorophyll a/b-binding protein [Nicotiana sylvestris];; (*SW
NbS00009017g0002.1 **Mass:** 28464 **Score:** 151 **Matches:** 6(6) **Sequences:** 3(3)
NbS00009017g0002.1 protein AED:0.21 eAED:0.21 QI:0|-1|0|1|-1|1|1|0|267; (*GB) gi|3036955|dbj|BAA25396.1| (e_value=0.0) light harvesting chlorophyll a/b-binding protein [Nicotiana sylvestris];; (*SW
NbS00016807g0007.1 **Mass:** 33516 **Score:** 151 **Matches:** 6(6) **Sequences:** 3(3)
NbS00016807g0007.1 protein AED:0.33 eAED:0.40 QI:0|0|0|1|0.5|0.66|3|0|315; (*GB) gi|3036955|dbj|BAA25396.1| (e_value=1e-167) light harvesting chlorophyll a/b-binding protein [Nicotiana sylvestris];
NbS00046604g0001.1 **Mass:** 28532 **Score:** 151 **Matches:** 6(6) **Sequences:** 3(3)
NbS00046604g0001.1 protein AED:0.31 eAED:0.31 QI:0|-1|0|1|-1|1|1|0|267; (*GB) gi|115825|sp|P07369.1|CB2G_SOLLC (e_value=0.0) RecName: Full=Chlorophyll a-b binding protein 3C, chloroplastic; AltName
NbC25792767g0001.1 **Mass:** 15882 **Score:** 151 **Matches:** 6(6) **Sequences:** 3(3)
NbC25792767g0001.1 protein AED:0.05 eAED:0.05 QI:0|-1|1|1|-1|1|1|0|149; (*GB) gi|3036953|dbj|BAA25395.1| (e_value=3e-103) light harvesting chlorophyll a/b-binding protein [Nicotiana sylvestris];;
NbS00016045g0013.1 **Mass:** 61743 **Score:** 151 **Matches:** 6(6) **Sequences:** 3(3)
NbS00016045g0013.1 protein AED:0.20 eAED:0.21 QI:0|0|0|0.85|0.66|0.57|7|0|569; (*GB) gi|115805|sp|P27495.1|CB24_TOBAC (e_value=2e-158) RecName: Full=Chlorophyll a-b binding protein 40, chloroplast:

---

9. NbS00000471g0009.1 **Mass:** 32669 **Score:** 146 **Matches:** 5(5) **Sequences:** 3(3) **emPAI:** 0.34
NbS00000471g0009.1 protein AED:0.15 eAED:0.15 QI:0|0|0.33|1|1|1|3|0|301; (*GB) gi|115802|sp|P27494.1|CB23_TOBAC (e_value=0.0) RecName: Full=Chlorophyll a-b binding protein 36, chloroplastic; AltNar

Query	Observed	Mr(expt)	Mr(calc)	ppm	Miss	Score	Expect	Rank	Unique	Peptide
12031	492.2531	982.4916	982.4913	0.36	0	57	0.00013	1		K.FGEAVWFK.A 12029 12030
12574	528.2689	1054.5233	1054.5229	0.32	0	34	0.025	1		R.ELEVIHCR.W
12896	549.7900	1097.5654	1097.5717	-5.70	0	62	4.7e-005	1	U	R.VGGGPLGEGLDK.I

Proteins matching the same set of peptides:
NbS00032129g0003.1 **Mass:** 28757 **Score:** 146 **Matches:** 5(5) **Sequences:** 3(3)
NbS00032129g0003.1 protein AED:0.10 eAED:0.10 QI:62|1|1|1|1|1|2|290|265; (*SWP) sp|P12062|CB26_PETSP (e_value=0.0) Chlorophyll a-b binding protein 37, chloroplastic OS=Petunia sp. GN=CAB37 PE=3 SV=
NbS00050671g0012.1 **Mass:** 28761 **Score:** 146 **Matches:** 5(5) **Sequences:** 3(3)
NbS00050671g0012.1 protein AED:0.04 eAED:0.04 QI:0|1|0.5|1|1|1|2|0|265; (*GB) gi|115802|sp|P27494.1|CB23_TOBAC (e_value=0.0) RecName: Full=Chlorophyll a-b binding protein 36, chloroplastic; AltName

---

10. NbS00030156g0009.1 **Mass:** 31162 **Score:** 138 **Matches:** 5(4) **Sequences:** 4(3) **emPAI:** 0.35
NbS00030156g0009.1 protein AED:0.29 eAED:0.29 QI:170|1|0.9|1|0.77|0.7|10|232|281; (*GB) gi|358248282|ref|NP_001239854.1| (e_value=2e-70) uncharacterized protein LOC100818986 [Glycine max];; (*SWP)

Query	Observed	Mr(expt)	Mr(calc)	ppm	Miss	Score	Expect	Rank	Unique	Peptide
11367	447.7321	893.4496	893.4494	0.13	0	32	0.056	1	U	K.GSDEIVFK.A
14352	643.8150	1285.6155	1285.6150	0.37	0	31	0.044	1	U	R.AETPIDENEIR.I
18246	886.3937	1770.7728	1770.7697	1.75	0	75	1e-006	1	U	K.VLTDFDYDGEGSPSGGR.R 18245
19907	1037.5125	2073.0104	2073.0089	0.72	0	48	0.00079	1	U	K.ELDMSSVGYQPPLPADQVK.V

---

11. NbS00001169g0153.1 **Mass:** 36448 **Score:** 127 **Matches:** 5(5) **Sequences:** 5(5) **emPAI:** 0.55
NbS00001169g0153.1 protein AED:0.25 eAED:0.25 QI:0|0.5|0.4|1|1|1|5|0|341

Query	Observed	Mr(expt)	Mr(calc)	ppm	Miss	Score	Expect	Rank	Unique	Peptide
11522	458.2660	914.5175	914.5185	-1.14	0	38	0.015	1	U	K.VIQVVSDR.N
12230	505.8339	1009.6532	1009.6535	-0.37	0	51	5.3e-005	1	U	K.AVALVLPSLK.G

14032	627.8098	1253.6051	1253.6040	0.83	0	64	2.7e-005	1	U	K.TFAEEVNAAFR.E
15115	692.8940	1383.7734	1383.7722	0.91	0	37	0.0082	1		R.AAALNIVPTSTGAAK.A
16862	807.4238	1612.8330	1612.8308	1.34	0	38	0.01	1	U	K.DSPLDVIAINDTGGVK.Q

---

12. NbS00007993g0305.1 **Mass:** 30118 **Score:** 121 **Matches:** 4(4) **Sequences:** 3(3) **emPAI:** 0.37

NbS00007993g0305.1 protein AED:0.00 eAED:0.00 QI:0|-1|0|1|-1|1|1|0|261

Query	Observed	Mr(expt)	Mr(calc)	ppm	Miss	Score	Expect	Rank	Unique	Peptide
12921	551.3300	1100.6454	1100.6441	1.23	0	67	9.9e-006	1	U	K.VLAELLSASAK.D 12922
13542	592.3384	1182.6622	1182.6608	1.18	0	46	0.0012	1	U	K.NQITAEAIVPK.N
17680	838.4271	1674.8396	1674.8334	3.70	0	45	0.0018	1	U	R.YELYPFFFPLDPK.E

---

13. NbS00005377g0008.1 **Mass:** 27332 **Score:** 116 **Matches:** 5(5) **Sequences:** 3(3) **emPAI:** 0.41

NbS00005377g0008.1 protein AED:0.46 eAED:0.46 QI:0|0|0|1|1|1|2|0|258; (*GB) gi|3036946|dbj|BAA25391.1| (e_value=2e-175) light harvesting chlorophyll a/b-binding protein [Nicotiana sylvestris];; (*S

Query	Observed	Mr(expt)	Mr(calc)	ppm	Miss	Score	Expect	Rank	Unique	Peptide
12031	492.2531	982.4916	982.4913	0.36	0	57	0.00013	1		K.FGEAVWFK.A 12029 12030
12574	528.2689	1054.5233	1054.5229	0.32	0	34	0.025	1		R.ELEVIHCR.W
18482	606.6442	1816.9109	1816.9108	0.03	0	33	0.033	1	U	K.TVAKPVASSSPWYGPDR.V

Proteins matching the same set of peptides:

NbS00014580g0002.1 **Mass:** 28269 **Score:** 116 **Matches:** 5(5) **Sequences:** 3(3)

NbS00014580g0002.1 protein AED:0.38 eAED:0.38 QI:0|-1|0|1|-1|1|1|0|265; (*GB) gi|3036944|dbj|BAA25389.1| (e_value=0.0) light harvesting chlorophyll a/b-binding protein [Nicotiana sylvestris];; (*SW

NbS00014580g0005.1 **Mass:** 24933 **Score:** 116 **Matches:** 5(5) **Sequences:** 3(3)

NbS00014580g0005.1 protein AED:0.40 eAED:0.40 QI:0|-1|0|1|-1|1|1|0|231; (*GB) gi|3036944|dbj|BAA25389.1| (e_value=1e-167) light harvesting chlorophyll a/b-binding protein [Nicotiana sylvestris];;

---

14. NbS00010295g0007.1 **Mass:** 13224 **Score:** 106 **Matches:** 3(3) **Sequences:** 1(1) **emPAI:** 0.26

NbS00010295g0007.1 protein AED:0.12 eAED:0.12 QI:0|0|0|0.5|1|1|2|0|119; (*SWP) sp|P69569|RBL_EUPES (e_value=5e-22) Ribulose bisphosphate carboxylase large chain (Fragment) OS=Euphorbia esula GN=rbc

Query	Observed	Mr(expt)	Mr(calc)	ppm	Miss	Score	Expect	Rank	Unique	Peptide
12323	511.2683	1020.5221	1020.5240	-1.83	0	64	4e-005	1	U	K.DTDILAAFR.V 12324 12326

Proteins matching the same set of peptides:

NbS00047753g0001.1 **Mass:** 13332 **Score:** 106 **Matches:** 3(3) **Sequences:** 1(1)

NbS00047753g0001.1 protein AED:0.28 eAED:0.28 QI:0|0|0|0.5|1|0|2|0|115; (*GB) gi|306481964|dbj|BAJ17086.1| (e_value=9e-37) ribulose-1,5-bisphosphate carboxylase/oxygenase large subunit [Solanum sp.

---

15. NbS00010583g0001.1 **Mass:** 22083 **Score:** 105 **Matches:** 5(5) **Sequences:** 3(3) **emPAI:** 0.76

NbS00010583g0001.1 protein AED:0.25 eAED:0.25 QI:0|-1|0|1|-1|1|1|0|210; (*GB) gi|31711507|dbj|BAC77634.1| (e_value=2e-132) 24K germin like protein [Nicotiana tabacum];; (*SWP) sp|O04011|ABP20_PRUPE

Query	Observed	Mr(expt)	Mr(calc)	ppm	Miss	Score	Expect	Rank	Unique	Peptide
10184	349.2338	696.4531	696.4534	-0.41	0	33	0.0049	1	U	K.LNPLIK.A
19855	683.7057	2048.0954	2048.0943	0.57	0	37	0.0079	1	U	K.KPSAVTANDFVFSGLATPVK.L 19857
20313	762.7411	2285.2016	2285.1991	1.13	0	47	0.00068	1	U	K.AAVTPAFAPQFPGLNGLGISMAR.L
20350	768.0720	2301.1942	2301.1940	0.12	0	(44)	0.0015	1	U	K.AAVTPAFAPQFPGLNGLGISMAR.L

Proteins matching the same set of peptides:

NbC25250105g0001.1 **Mass:** 20517 **Score:** 105 **Matches:** 5(5) **Sequences:** 3(3)

NbC25250105g0001.1 protein AED:0.01 eAED:0.01 QI:0|-1|1|1|-1|1|1|0|194; (*GB) gi|31711507|dbj|BAC77634.1| (e_value=1e-120) 24K germin like protein [Nicotiana tabacum];; (*SWP) sp|O04011|ABP20_PRUPE

---

16. NbS00004085g0015.1 **Mass:** 23594 **Score:** 102 **Matches:** 2(2) **Sequences:** 2(2) **emPAI:** 0.30

NbS00004085g0015.1 protein AED:0.29 eAED:0.29 QI:92|0.25|0.2|1|1|1|5|0|225; (*GB) gi|110377793|gb|ABG73417.1| (e_value=7e-87) chloroplast pigment-binding protein CP26 [Nicotiana tabacum];; (*SWP) s

Query	Observed	Mr(expt)	Mr(calc)	ppm	Miss	Score	Expect	Rank	Unique	Peptide
11931	485.7641	969.5137	969.5131	0.64	0	66	1.5e-005	1	U	K.DPDQAAILK.V
17788	848.9492	1695.8838	1695.8832	0.34	0	67	1.2e-005	1	U	K.TGALLLDGNTLNYFGK.N

Proteins matching the same set of peptides:

NbS00008488g0031.1 **Mass:** 15157 **Score:** 102 **Matches:** 2(2) **Sequences:** 2(2)

NbS00008488g0031.1 protein AED:0.34 eAED:0.21 QI:689|0.75|0.4|1|0.75|0.8|5|0|143; (*GB) gi|261351266|gb|ACX71300.1| (e_value=1e-88) chloroplast pigment-binding protein CP26 [Capsicum annuum];; (*SW

---

17. NbS00001942g0006.1 **Mass:** 15769 **Score:** 102 **Matches:** 5(5) **Sequences:** 2(2) **emPAI:** 0.48
NbS00001942g0006.1 protein AED:0.00 eAED:0.00 QI:75|1|1|1|1|1|2|198|151; (*GB) gi|351725389|ref|NP_001235555.1| (e_value=1e-63) uncharacterized protein LOC100305705 [Glycine max];; (*SWP) sp|Q6L50(

Query	Observed	Mr(expt)	Mr(calc)	ppm	Miss	Score	Expect	Rank	Unique	Peptide
10775	404.2633	806.5120	806.5127	-0.81	0	42	0.00075	1	U	R.HVLLAVR.N 10782
11726	472.7693	943.5240	943.5240	0.07	0	51	0.00072	1		K.AGLQFPVGR.I 11722 11727

Proteins matching the same set of peptides:
NbS00008178g0018.1 **Mass:** 15524 **Score:** 102 **Matches:** 5(5) **Sequences:** 2(2)
NbS00008178g0018.1 protein AED:0.03 eAED:0.03 QI:116|1|1|1|1|1|2|157|148; (*GB) gi|27529852|dbj|BAC53941.1| (e_value=4e-56) H2A histone [Nicotiana tabacum];; (*SWP) sp|P25469|H2A1_SOLLC (e_value=1e
NbS00010297g0001.1 **Mass:** 15303 **Score:** 102 **Matches:** 5(5) **Sequences:** 2(2)
NbS00010297g0001.1 protein AED:0.04 eAED:0.04 QI:122|1|1|1|1|1|2|350|144; (*GB) gi|225439749|ref|XP_002274570.1| (e_value=6e-44) PREDICTED: probable histone H2A.4 isoform 1 [Vitis vinifera];; (*SWI
NbS00012269g0005.1 **Mass:** 15797 **Score:** 102 **Matches:** 5(5) **Sequences:** 2(2)
NbS00012269g0005.1 protein AED:0.02 eAED:0.02 QI:75|1|1|1|1|1|2|181|151; (*GB) gi|351725389|ref|NP_001235555.1| (e_value=1e-63) uncharacterized protein LOC100305705 [Glycine max];; (*SWP) sp|Q2HU6
NbS00020235g0008.1 **Mass:** 15471 **Score:** 102 **Matches:** 5(5) **Sequences:** 2(2)
NbS00020235g0008.1 protein AED:0.06 eAED:0.06 QI:118|1|1|1|1|1|2|157|148; (*GB) gi|27529852|dbj|BAC53941.1| (e_value=7e-58) H2A histone [Nicotiana tabacum];; (*SWP) sp|P25469|H2A1_SOLLC (e_value=3e
NbS00024367g0008.1 **Mass:** 15926 **Score:** 102 **Matches:** 5(5) **Sequences:** 2(2)
NbS00024367g0008.1 protein AED:0.03 eAED:0.03 QI:74|1|1|1|1|1|2|309|151; (*GB) gi|255584772|ref|XP_002533104.1| (e_value=5e-54) histone h2a, putative [Ricinus communis];; (*SWP) sp|Q2HU65|H2A2_MED
NbS00032978g0018.1 **Mass:** 15912 **Score:** 102 **Matches:** 5(5) **Sequences:** 2(2)
NbS00032978g0018.1 protein AED:0.04 eAED:0.04 QI:72|1|1|1|1|1|2|205|151; (*GB) gi|6009909|dbj|BAA85117.1| (e_value=1e-53) histone H2A-like protein [Solanum melongena];; (*SWP) sp|Q2HU65|H2A2_MEDTR
NbS00056349g0008.1 **Mass:** 15304 **Score:** 102 **Matches:** 5(5) **Sequences:** 2(2)
NbS00056349g0008.1 protein AED:0.02 eAED:0.02 QI:262|1|1|1|1|1|2|424|145; (*GB) gi|225439749|ref|XP_002274570.1| (e_value=2e-53) PREDICTED: probable histone H2A.4 isoform 1 [Vitis vinifera];; (*SWI

---

18. NbS00035687g0007.1 **Mass:** 28986 **Score:** 102 **Matches:** 3(3) **Sequences:** 3(3) **emPAI:** 0.39
NbS00035687g0007.1 protein AED:0.23 eAED:0.23 QI:300|1|0.75|1|1|1|4|0|274; (*GB) gi|84620802|gb|ABC59515.1| (e_value=9e-120) chloroplast photosystem II 22 kDa component [Nicotiana benthamiana];; (

Query	Observed	Mr(expt)	Mr(calc)	ppm	Miss	Score	Expect	Rank	Unique	Peptide
11437	453.2457	904.4768	904.4767	0.14	0	46	0.0025	1	U	K.ANELFVGR.L
16542	790.9201	1579.8257	1579.8246	0.66	0	78	9e-007	1	U	K.SALGLSEGGPLFGFTK.A
16565	792.8965	1583.7784	1583.7832	-3.00	0	32	0.038	1		K.VEDGIFGTSGGIGFTK.Q

---

19. NbS00007511g0005.1 **Mass:** 117195 **Score:** 101 **Matches:** 5(4) **Sequences:** 4(3) **emPAI:** 0.12
NbS00007511g0005.1 protein AED:0.26 eAED:0.26 QI:0|0.62|0.55|0.88|1|1|9|0|1084; (*GB) gi|359477631|ref|XP_002274485.2| (e_value=0.0) PREDICTED: uncharacterized protein LOC100252874 [Vitis vinifera]

Query	Observed	Mr(expt)	Mr(calc)	ppm	Miss	Score	Expect	Rank	Unique	Peptide
10522	379.2319	756.4493	756.4494	-0.07	0	30	0.07	1	U	K.IAGDLLR.N
11602	464.2904	926.5662	926.5661	0.09	1	35	0.0093	1	U	R.RLEQILR.S
13815	613.8583	1225.7021	1225.7030	-0.74	0	45	0.0011	1	U	K.TPILVATDVAAR.G 9639
18356	897.4791	1792.9436	1792.9431	0.24	0	53	0.00026	1	U	R.NPVQVNIGNVDQLAANK.S

---

20. NbS00009638g0019.1 **Mass:** 20584 **Score:** 100 **Matches:** 4(4) **Sequences:** 3(3) **emPAI:** 0.58
NbS00009638g0019.1 protein AED:0.11 eAED:0.11 QI:333|1|1|1|1|1|3|176|181; (*GB) gi|132118|sp|P26573.1|RBS8_NICPL (e_value=6e-118) RecName: Full=Ribulose bisphosphate carboxylase small chain 8B, ch

Query	Observed	Mr(expt)	Mr(calc)	ppm	Miss	Score	Expect	Rank	Unique	Peptide
11649	467.2611	932.5077	932.5080	-0.32	0	60	9.3e-005	1		R.IIGFDNVR.Q
12083	495.7612	989.5079	989.5083	-0.38	0	39	0.013	1	U	K.AYPQAWVR.I
18399	901.9451	1801.8756	1801.8709	2.60	0	39	0.0063	1	U	R.QVQCISFIAYKPEGY.- 18398

Proteins matching the same set of peptides:
NbS00022486g0001.1 **Mass:** 20485 **Score:** 100 **Matches:** 4(4) **Sequences:** 3(3)
NbS00022486g0001.1 protein AED:0.21 eAED:0.21 QI:0|1|0.66|1|1|1|3|0|181; (*GB) gi|132118|sp|P26573.1|RBS8_NICPL (e_value=4e-116) RecName: Full=Ribulose bisphosphate carboxylase small chain 8B, chl
NbS00027369g0007.1 **Mass:** 20455 **Score:** 100 **Matches:** 4(4) **Sequences:** 3(3)
NbS00027369g0007.1 protein AED:0.16 eAED:0.16 QI:0|1|0.66|1|1|1|3|0|181; (*GB) gi|132118|sp|P26573.1|RBS8_NICPL (e_value=2e-114) RecName: Full=Ribulose bisphosphate carboxylase small chain 8B, chl
NbS00041372g0008.1 **Mass:** 22328 **Score:** 100 **Matches:** 4(4) **Sequences:** 3(3)
NbS00041372g0008.1 protein AED:0.22 eAED:0.22 QI:8|0.66|0.5|1|1|1|4|0|197; (*SWP) sp|P26573|RBS8_NICPL (e_value=5e-110) Ribulose bisphosphate carboxylase small chain 8B, chloroplastic OS=Nicotiana
NbS00041372g0009.1 **Mass:** 15410 **Score:** 100 **Matches:** 4(4) **Sequences:** 3(3)
NbS00041372g0009.1 protein AED:0.23 eAED:0.24 QI:0|0.5|0|1|0.5|0.66|3|0|137; (*SWP) sp|P26573|RBS8_NICPL (e_value=7e-74) Ribulose bisphosphate carboxylase small chain 8B, chloroplastic OS=Nicotiana

---

21. NbS00004956g0015.1 **Mass:** 9825 **Score:** 98 **Matches:** 4(4) **Sequences:** 2(2) **emPAI:** 1.47
NbS00004956g0015.1 protein AED:0.25 eAED:0.30 QI:0|0|0|1|0|0|2|0|84; (*GB) gi|42718201|gb|AAS38532.1| (e_value=7e-41) ribulose-1,5-bisphosphate carboxylase/oxygenase large subunit [Hygrolembidium a

Query	Observed	Mr(expt)	Mr(calc)	ppm	Miss	Score	Expect	Rank	Unique	Peptide
11473	455.7260	909.4375	909.4378	-0.31	0	33	0.032	1	U	R.AVYECLR.G
15671	489.2560	1464.7461	1464.7474	-0.86	0	(45)	0.002	1	U	K.TFQGPPHGIQVER.D
15672	733.3809	1464.7473	1464.7474	-0.06	0	52	0.00045	1	U	K.TFQGPPHGIQVER.D 15674

22. gi|119581148|gb|EAW60744.1| **Mass:** 57754 **Score:** 96 **Matches:** 7(7) **Sequences:** 6(6) **emPAI:** 0.39
gi|119581148|gb|EAW60744.1| keratin 9 (epidermolytic palmoplantar keratoderma) [Homo sapiens]

Query	Observed	Mr(expt)	Mr(calc)	ppm	Miss	Score	Expect	Rank	Unique	Peptide
11380	449.2104	896.4063	896.4062	0.09	0	32	0.033	1	U	R.MTLDDFR.I
12620	530.7853	1059.5561	1059.5560	0.09	0	36	0.021	1	U	K.TLLDIDNTR.M 12622
13685	603.8043	1205.5941	1205.5962	-1.73	0	38	0.012	1	U	R.QVLDNLTMEK.S
18637	617.9810	1850.9210	1850.9196	0.78	1	30	0.052	1	U	K.TLNDMRQEYEQLIAK.N
20602	837.3824	2509.1255	2509.1245	0.42	0	50	0.0003	1	U	K.EIETYHNLLEGGQEDFESSGAGK.I
20890	1088.8458	3263.5156	3263.5066	2.78	0	32	0.014	1	U	K.DIENQYETQITQIEHEVSSSGQEVQSSAK.E

23. NbS00017935g0003.1 **Mass:** 31122 **Score:** 94 **Matches:** 3(3) **Sequences:** 3(3) **emPAI:** 0.36
NbS00017935g0003.1 protein AED:0.00 eAED:0.00 QI:240|1|0.5|1|1|1|2|0|284; (*SWP) sp|Q9XF88|CB4B_ARATH (e_value=4e-162) Chlorophyll a-b binding protein CP29.2, chloroplastic OS=Arabidopsis thaliana

Query	Observed	Mr(expt)	Mr(calc)	ppm	Miss	Score	Expect	Rank	Unique	Peptide
12375	515.2880	1028.5614	1028.5614	-0.07	0	55	0.00023	1	U	K.NLAGDIIGTR.T
18159	878.4395	1754.8644	1754.8628	0.89	0	54	0.00021	1	U	K.STPFQPYSEVFGLQR.F
19725	997.4997	1992.9848	1992.9833	0.78	0	40	0.0064	1	U	R.LYPGGSFFDPLGLAADPEK.K

Proteins matching the same set of peptides:
NbS00027305g0008.1 **Mass:** 31084 **Score:** 94 **Matches:** 3(3) **Sequences:** 3(3)
NbS00027305g0008.1 protein AED:0.31 eAED:0.31 QI:0|1|0.5|1|1|1|2|0|284; (*GB) gi|110377766|gb|ABG73415.1| (e_value=0.0) chloroplast pigment-binding protein CP29 [Nicotiana tabacum];; (*SWP) sp|Q9XI

24. NbS00004238g0018.1 **Mass:** 34571 **Score:** 92 **Matches:** 5(5) **Sequences:** 4(4) **emPAI:** 0.44
NbS00004238g0018.1 protein AED:0.16 eAED:0.16 QI:156|0.87|0.88|1|0.87|0.77|9|398|304; (*GB) gi|77416949|gb|ABA81870.1| (e_value=2e-140) unknown [Solanum tuberosum];; (*SWP) sp|P43333|RU2A_ARATH (e_

Query	Observed	Mr(expt)	Mr(calc)	ppm	Miss	Score	Expect	Rank	Unique	Peptide
13400	583.3046	1164.5947	1164.5927	1.69	0	34	0.035	1		K.LENFPYLNR.L
14221	639.3589	1276.7032	1276.7027	0.42	0	50	0.00058	1		K.FLSLLDNNITK.R
15993	757.9124	1513.8103	1513.8100	0.20	0	34	0.026	1	U	K.AAILNSQTLEEVAR.L 15992
18677	930.9714	1859.9282	1859.9265	0.92	0	54	0.00023	1		R.SGQLPADLNIGDYDIAAK.K

25. NbS00004744g0013.1 **Mass:** 13896 **Score:** 85 **Matches:** 4(4) **Sequences:** 2(2) **emPAI:** 0.55
NbS00004744g0013.1 protein AED:0.20 eAED:0.20 QI:0|1|1|1|1|1|2|316|133; (*GB) gi|118484619|gb|ABK94182.1| (e_value=3e-58) unknown [Populus trichocarpa];; (*SWP) sp|Q9LHQ5|H2A2_ARATH (e_value=4e-57)

Query	Observed	Mr(expt)	Mr(calc)	ppm	Miss	Score	Expect	Rank	Unique	Peptide
11032	418.7588	835.5030	835.5028	0.26	0	40	0.0025	1	U	R.HIQLAVR.N
11726	472.7693	943.5240	943.5240	0.07	0	51	0.00072	1		K.AGLQFPVGR.I 11722 11727

Proteins matching the same set of peptides:
NbS00009017g0013.1 **Mass:** 13926 **Score:** 85 **Matches:** 4(4) **Sequences:** 2(2)
NbS00009017g0013.1 protein AED:0.25 eAED:0.25 QI:55|1|1|1|1|1|2|261|133; (*GB) gi|255637355|gb|ACU19007.1| (e_value=7e-48) unknown [Glycine max];; (*SWP) sp|Q9LD28|H2A6_ARATH (e_value=1e-48) Histor
NbS00018143g0005.1 **Mass:** 13882 **Score:** 85 **Matches:** 4(4) **Sequences:** 2(2)
NbS00018143g0005.1 protein AED:0.24 eAED:0.24 QI:0|1|1|1|1|1|2|571|133; (*GB) gi|118484619|gb|ABK94182.1| (e_value=7e-59) unknown [Populus trichocarpa];; (*SWP) sp|Q9LHQ5|H2A2_ARATH (e_value=7e-57)
NbS00025215g0008.1 **Mass:** 13940 **Score:** 85 **Matches:** 4(4) **Sequences:** 2(2)
NbS00025215g0008.1 protein AED:0.23 eAED:0.24 QI:83|1|0.5|1|1|1|2|0|133; (*GB) gi|224115182|ref|XP_002316965.1| (e_value=1e-61) histone H2 [Populus trichocarpa];; (*SWP) sp|Q9C681|H2A1_ARATH (e_va
NbS00027299g0011.1 **Mass:** 14647 **Score:** 85 **Matches:** 4(4) **Sequences:** 2(2)
NbS00027299g0011.1 protein AED:0.17 eAED:0.17 QI:77|1|1|1|1|1|2|132|142; (*SWP) sp|O65759|H2AX_CICAR (e_value=4e-57) Histone H2AX OS=Cicer arietinum GN=HIS2A PE=2 SV=1;; (*TAIR) AT1G54690.1 (e_valu
NbS00028358g0003.1 **Mass:** 14638 **Score:** 85 **Matches:** 4(4) **Sequences:** 2(2)
NbS00028358g0003.1 protein AED:0.23 eAED:0.23 QI:77|1|1|1|1|1|2|146|142; (*GB) gi|350627694|gb|AEQ33640.1| (e_value=2e-59) histone H2A [Lycium barbarum];; (*SWP) sp|O65759|H2AX_CICAR (e_value=2e-5
NbS00030290g0015.1 **Mass:** 14915 **Score:** 85 **Matches:** 4(4) **Sequences:** 2(2)

NbS00030290g0015.1 protein AED:0.02 eAED:0.02 QI:125|1|1|1|1|1|2|1006|144; (*GB) gi|350627694|gb|AEQ33640.1| (e_value=3e-59) histone H2A [Lycium barbarum];; (*SWP) sp|O65759|H2AX_CICAR (e_value=4e-

NbS00035003g0001.1 **Mass:** 13912 **Score:** 85 **Matches:** 4(4) **Sequences:** 2(2)
NbS00035003g0001.1 protein AED:0.45 eAED:0.45 QI:60|1|0.5|1|1|1|2|0|133; (*SWP) sp|Q9C681|H2A1_ARATH (e_value=1e-57) Probable histone H2A.1 OS=Arabidopsis thaliana GN=At1g51060 PE=1 SV=1;; (*TAIR)

NbS00046907g0001.1 **Mass:** 15266 **Score:** 85 **Matches:** 4(4) **Sequences:** 2(2)
NbS00046907g0001.1 protein AED:0.22 eAED:0.22 QI:0|-1|0|1|-1|1|1|0|142; (*GB) gi|225451096|ref|XP_002265878.1| (e_value=2e-66) PREDICTED: histone H2AX [Vitis vinifera];; (*SWP) sp|O65759|H2AX_CICAI

NbC23758669g0001.1 **Mass:** 9985 **Score:** 85 **Matches:** 4(4) **Sequences:** 2(2)
NbC23758669g0001.1 protein AED:0.00 eAED:0.00 QI:0|-1|0|1|-1|1|1|0|90; (*GB) gi|242038317|ref|XP_002466553.1| (e_value=1e-38) hypothetical protein SORBIDRAFT_01g009820 [Sorghum bicolor];; (*SWP) sp

NbS00034921g0005.1 **Mass:** 23181 **Score:** 85 **Matches:** 4(4) **Sequences:** 2(2)
NbS00034921g0005.1 protein AED:0.11 eAED:0.11 QI:35|0.33|0.25|1|0.33|0.25|4|0|216; (*GB) gi|356504894|ref|XP_003521229.1| (e_value=2e-46) PREDICTED: histone H2AX-like [Glycine max];; (*SWP) sp|O04

---

26. NbS00010009g0002.1 **Mass:** 118617 **Score:** 85 **Matches:** 4(2) **Sequences:** 4(2) **emPAI:** 0.08
NbS00010009g0002.1 protein AED:0.20 eAED:0.20 QI:0|1|1|1|1|1|21|408|1058; (*SWP) sp|O04379|AGO1_ARATH (e_value=0.0) Protein argonaute 1 OS=Arabidopsis thaliana GN=AGO1 PE=1 SV=1;; (*TAIR) AT1G4841(

Query	Observed	Mr(expt)	Mr(calc)	ppm	Miss	Score	Expect	Rank	Unique	Peptide
8674	517.2900	1032.5654	1032.5564	8.80	0	31	0.12	2		R.ISGLTSQATR.E
12819	544.3096	1086.6047	1086.6033	1.28	0	30	0.071	1	U	R.NTVLVDALSR.R
15327	707.8443	1413.6740	1413.6736	0.32	0	63	2.7e-005	1	U	K.ITLIDDDDGPGGAR.R
15372	710.8967	1419.7788	1419.7762	1.82	0	47	0.0011	1		K.SLYTAGPLPFVQK.D

Proteins matching the same set of peptides:
NbS00034990g0010.1 **Mass:** 119669 **Score:** 85 **Matches:** 4(2) **Sequences:** 4(2)
NbS00034990g0010.1 protein AED:0.15 eAED:0.15 QI:0|0.95|0.95|1|0.95|0.90|21|286|1067; (*GB) gi|84688906|gb|ABC61502.1| (e_value=0.0) AGO1-1, partial [Nicotiana benthamiana];; (*SWP) sp|Q7XSA2|AGO1I

---

27. NbS00026922g0005.1 **Mass:** 50625 **Score:** 82 **Matches:** 2(2) **Sequences:** 2(2) **emPAI:** 0.13
NbS00026922g0005.1 protein AED:0.33 eAED:0.34 QI:0|0.75|0.4|0.8|1|1|5|0|468; (*GB) gi|225451915|ref|XP_002282805.1| (e_value=1e-124) PREDICTED: uncharacterized protein LOC100244706 [Vitis vinifera]

Query	Observed	Mr(expt)	Mr(calc)	ppm	Miss	Score	Expect	Rank	Unique	Peptide
13170	568.7886	1135.5627	1135.5621	0.50	0	63	4.2e-005	1	U	K.GSALAAYEAQR.G
13733	607.3492	1212.6838	1212.6826	0.97	0	48	0.0007	1	U	K.VQLLTQDLQR.A

---

28. NbS00010743g0014.1 **Mass:** 32611 **Score:** 79 **Matches:** 4(4) **Sequences:** 4(4) **emPAI:** 0.47
NbS00010743g0014.1 protein AED:0.24 eAED:0.24 QI:0|1|1|1|1|1|8|290|288; (*GB) gi|77416949|gb|ABA81870.1| (e_value=2e-155) unknown [Solanum tuberosum];; (*SWP) sp|P43333|RU2A_ARATH (e_value=2e-117)

Query	Observed	Mr(expt)	Mr(calc)	ppm	Miss	Score	Expect	Rank	Unique	Peptide
13400	583.3046	1164.5947	1164.5927	1.69	0	34	0.035	1		K.LENFPYLNR.L
13937	621.3486	1240.6827	1240.6815	0.94	0	31	0.047	1	U	R.INPNIGEFLPK.L
14221	639.3589	1276.7032	1276.7027	0.42	0	50	0.00058	1		K.FLSLLDNNITK.R
18677	930.9714	1859.9282	1859.9265	0.92	0	54	0.00023	1		R.SGQLPADLNIGDYDIAAK.K

---

29. NbS00043180g0003.1 **Mass:** 52871 **Score:** 77 **Matches:** 2(2) **Sequences:** 2(2) **emPAI:** 0.13
NbS00043180g0003.1 protein AED:0.10 eAED:0.10 QI:0|1|0.5|1|1|1|4|944|486; (*GB) gi|113461|sp|P27081.1|ADT2_SOLTU (e_value=0.0) RecName: Full=ADP,ATP carrier protein, mitochondrial; AltName: Full=AI

Query	Observed	Mr(expt)	Mr(calc)	ppm	Miss	Score	Expect	Rank	Unique	Peptide
13585	596.3228	1190.6310	1190.6295	1.20	0	57	0.00017	1		R.AVAGAGVLAGYDK.L
13903	618.8329	1235.6513	1235.6510	0.29	0	44	0.003	1	U	K.TLASDGIAGLYR.G

Proteins matching the same set of peptides:
NbS00043181g0003.1 **Mass:** 52686 **Score:** 77 **Matches:** 2(2) **Sequences:** 2(2)
NbS00043181g0003.1 protein AED:0.20 eAED:0.20 QI:0|1|0.5|1|1|1|4|682|483; (*GB) gi|113461|sp|P27081.1|ADT2_SOLTU (e_value=0.0) RecName: Full=ADP,ATP carrier protein, mitochondrial; AltName: Full=AI

---

30. NbS00014429g0006.1 **Mass:** 33726 **Score:** 77 **Matches:** 3(3) **Sequences:** 3(3) **emPAI:** 0.32
NbS00014429g0006.1 protein AED:0.21 eAED:0.23 QI:0|0|0|1|1|1|2|0|322; (*GB) gi|255578055|ref|XP_002529898.1| (e_value=4e-54) small nuclear ribonucleoprotein-associated protein, putative [Ricinus co

Query	Observed	Mr(expt)	Mr(calc)	ppm	Miss	Score	Expect	Rank	Unique	Peptide
11319	442.8002	883.5859	883.5855	0.49	0	42	0.00091	1	U	R.TLGLVLLR.G
12908	550.7819	1099.5492	1099.5484	0.68	0	39	0.0088	1	U	K.MLQYINYR.M
19808	1013.4763	2024.9380	2024.9361	0.93	0	43	0.0024	1	U	R.GEEVISMTVEGPPPPDESR.V

---

31. NbS00045545g0004.1 **Mass:** 64751 **Score:** 75 **Matches:** 3(3) **Sequences:** 3(3) **emPAI:** 0.16

NbS00045545g0004.1 protein AED:0.13 eAED:0.13 QI:0|0.78|0.73|0.86|0.92|0.86|15|481|616; (*SWP) sp|Q7XTT4|NUCL2_ORYSJ (e_value=6e-73) Nucleolin 2 OS=Oryza sativa subsp. japonica GN=Os04g0620700 PE=2

Query	Observed	Mr(expt)	Mr(calc)	ppm	Miss	Score	Expect	Rank	Unique	Peptide
12220	505.2691	1008.5237	1008.5240	-0.36	0	51	0.0006	1	U	R.SEGTTIFVR.G
14439	649.8576	1297.7007	1297.6990	1.29	0	38	0.0096	1	U	K.ALELNGQDLLGR.A
18611	923.4244	1844.8343	1844.8250	5.03	0	33	0.019	1	U	K.GMAYIEFANADSDALNK.A

32. NbS00008911g0002.1 **Mass:** 47595 **Score:** 75 **Matches:** 3(3) **Sequences:** 3(3) **emPAI:** 0.22

NbS00008911g0002.1 protein AED:0.35 eAED:0.35 QI:460|0.77|0.7|1|1|1|10|229|443; (*GB) gi|327198779|emb|CBL43264.1| (e_value=0.0) glyceraldehyde-3-phosphate dehydrogenase [Solanum tuberosum];; (*SWI

Query	Observed	Mr(expt)	Mr(calc)	ppm	Miss	Score	Expect	Rank	Unique	Peptide
14375	645.3337	1288.6529	1288.6511	1.45	0	42	0.0053	1	U	K.IVDNETISVDGK.H
15115	692.8940	1383.7734	1383.7722	0.91	0	37	0.0082	1		R.AAALNIVPTSTGAAK.A
18252	886.9048	1771.7950	1771.7954	-0.23	0	39	0.0045	1	U	K.VVAWYDNEWGYSQR.V

Proteins matching the same set of peptides:

NbS00033331g0008.1 **Mass:** 45260 **Score:** 75 **Matches:** 3(3) **Sequences:** 3(3)

NbS00033331g0008.1 protein AED:0.30 eAED:0.30 QI:475|0.77|0.8|1|0.77|0.7|10|324|419; (*GB) gi|327198779|emb|CBL43264.1| (e_value=0.0) glyceraldehyde-3-phosphate dehydrogenase [Solanum tuberosum];;

33. NbS00003763g0016.1 **Mass:** 99171 **Score:** 73 **Matches:** 3(3) **Sequences:** 3(3) **emPAI:** 0.10

NbS00003763g0016.1 protein AED:0.10 eAED:0.12 QI:0|0.92|0.8|0.93|1|1|15|0|884; (*GB) gi|225441896|ref|XP_002284404.1| (e_value=0.0) PREDICTED: uncharacterized protein At2g41620 [Vitis vinifera];;

Query	Observed	Mr(expt)	Mr(calc)	ppm	Miss	Score	Expect	Rank	Unique	Peptide
12716	536.8226	1071.6307	1071.6288	1.80	0	40	0.0053	1	U	K.QLLTELLSR.D
12909	550.7885	1099.5625	1099.5621	0.30	0	38	0.01	1	U	R.EGINAEQLAR.D
13090	563.3178	1124.6211	1124.6190	1.86	0	54	0.00018	1	U	R.DGGIDILLGPR.G

Proteins matching the same set of peptides:

NbS00021908g0010.1 **Mass:** 102988 **Score:** 73 **Matches:** 3(3) **Sequences:** 3(3)

NbS00021908g0010.1 protein AED:0.11 eAED:0.11 QI:0|0.71|0.73|1|0.85|0.8|15|307|915; (*SWP) sp|O22224|Y2162_ARATH (e_value=0.0) Uncharacterized protein At2g41620 OS=Arabidopsis thaliana GN=At2g4162(

34. NbS00027742g0013.1 **Mass:** 39244 **Score:** 73 **Matches:** 3(2) **Sequences:** 2(1) **emPAI:** 0.08

NbS00027742g0013.1 protein AED:0.00 eAED:0.01 QI:0|1|1|1|1|1|7|309|363; (*GB) gi|77416977|gb|ABA81884.1| (e_value=4e-80) nuclear RNA binding protein-like [Solanum tuberosum];; (*TAIR) AT5G47210.1

Query	Observed	Mr(expt)	Mr(calc)	ppm	Miss	Score	Expect	Rank	Unique	Peptide
13061	561.2960	1120.5775	1120.5764	0.96	1	30	0.073	1	U	K.KNEDDIFIK.L
14954	680.8611	1359.7077	1359.7034	3.17	0	54	0.00026	1	U	K.IEDVGQFPSLGAK.- 14952

35. NbS00002894g0003.1 **Mass:** 42068 **Score:** 68 **Matches:** 2(2) **Sequences:** 2(2) **emPAI:** 0.16

NbS00002894g0003.1 protein AED:0.02 eAED:0.02 QI:0|1|0.5|1|1|1|2|0|386; (*GB) gi|78191448|gb|ABB29945.1| (e_value=0.0) ADP/ATP translocator-like [Solanum tuberosum];; (*SWP) sp|P25083|ADT1_SOLTU (e

Query	Observed	Mr(expt)	Mr(calc)	ppm	Miss	Score	Expect	Rank	Unique	Peptide
13585	596.3228	1190.6310	1190.6295	1.20	0	57	0.00017	1		R.AVAGAGVLAGYDK.L
13721	605.8200	1209.6254	1209.6142	9.28	0	38	0.011	1	U	R.QFNGLVDVYR.K

Proteins matching the same set of peptides:

NbS00020014g0003.1 **Mass:** 44842 **Score:** 68 **Matches:** 2(2) **Sequences:** 2(2)

NbS00020014g0003.1 protein AED:0.26 eAED:0.29 QI:0|0.66|0.75|1|1|1|4|459|410; (*GB) gi|78191448|gb|ABB29945.1| (e_value=0.0) ADP/ATP translocator-like [Solanum tuberosum];; (*SWP) sp|P25083|ADT1_SC

NbS00017949g0009.1 **Mass:** 42022 **Score:** 68 **Matches:** 2(2) **Sequences:** 2(2)

NbS00017949g0009.1 protein AED:0.29 eAED:0.29 QI:81|1|1|1|0.66|0.75|4|419|386; (*GB) gi|78191448|gb|ABB29945.1| (e_value=0.0) ADP/ATP translocator-like [Solanum tuberosum];; (*SWP) sp|P25083|ADT1_S

36. NbS00002044g0005.1 **Mass:** 29321 **Score:** 67 **Matches:** 2(2) **Sequences:** 2(2) **emPAI:** 0.24

NbS00002044g0005.1 protein AED:0.28 eAED:0.28 QI:0|1|0.66|1|1|1|3|0|273; (*GB) gi|226872|prf||1609235A (e_value=3e-155) chlorophyll a/b binding protein;; (*SWP) sp|P27522|CB13_SOLLC (e_value=4e-15(

Query	Observed	Mr(expt)	Mr(calc)	ppm	Miss	Score	Expect	Rank	Unique	Peptide
14387	646.3432	1290.6718	1290.6720	-0.15	0	40	0.0082	1	U	K.WLAYGEIINGR.F
17063	815.4561	1628.8976	1628.8960	0.98	0	55	0.00012	1	U	R.FAMLGAAGAIAPEILGK.A

Proteins matching the same set of peptides:

NbS00006820g0015.1 **Mass:** 29146 **Score:** 67 **Matches:** 2(2) **Sequences:** 2(2)

NbS00006820g0015.1 protein AED:0.07 eAED:0.08 QI:0|1|0.66|1|1|1|3|0|273; (*GB) gi|226872|prf||1609235A (e_value=1e-149) chlorophyll a/b binding protein;; (*SWP) sp|P27522|CB13_SOLLC (e_value=3e-15(
NbS00010402g0001.1 **Mass:** 22688 **Score:** 67 **Matches:** 2(2) **Sequences:** 2(2)
NbS00010402g0001.1 protein AED:0.03 eAED:0.03 QI:315|0.5|0.66|1|0|0.66|3|354|209; (*GB) gi|226872|prf||1609235A (e_value=3e-129) chlorophyll a/b binding protein;; (*SWP) sp|P27522|CB13_SOLLC (e_va]
NbS00021892g0003.1 **Mass:** 26702 **Score:** 67 **Matches:** 2(2) **Sequences:** 2(2)
NbS00021892g0003.1 protein AED:0.04 eAED:0.04 QI:191|1|1|1|0.5|0.4|5|817|252; (*GB) gi|226872|prf||1609235A (e_value=2e-124) chlorophyll a/b binding protein;; (*SWP) sp|P27522|CB13_SOLLC (e_value=

---

37. NbS00027807g0002.1 **Mass:** 45317 **Score:** 66 **Matches:** 3(3) **Sequences:** 3(3) **emPAI:** 0.24
NbS00027807g0002.1 protein AED:0.05 eAED:0.05 QI:0|1|0.5|1|0|0|2|953|413; (*GB) gi|68566313|sp|Q40450.2|EFTUA_NICSY (e_value=0.0) RecName: Full=Elongation factor TuA, chloroplastic; Short=EF-TuA; I

Query	Observed	Mr(expt)	Mr(calc)	ppm	Miss	Score	Expect	Rank	Unique	Peptide
14524	654.2927	1306.5709	1306.5677	2.46	0	39	0.0051	1	U	K.YDEIDAAPEER.A
14719	664.3329	1326.6512	1326.6490	1.69	0	43	0.0028	1	U	R.STTVTGVEMFQK.I
17914	858.4550	1714.8955	1714.8923	1.83	0	33	0.029	1	U	K.ILDEAMAGDNVGLLLR.G

---

38. NbS00003163g0002.1 **Mass:** 22262 **Score:** 63 **Matches:** 2(2) **Sequences:** 1(1) **emPAI:** 0.15
NbS00003163g0002.1 protein ; (*GB) gi|146188483|emb|CAK12837.1| (e_value=1e-52) ribulose 1,5 biphosphate carboxylase/oxygenase [Liparia genistoides];; (*SWP) sp|P48709|RBL_NICDE (e_value=2e-52) Rib

Query	Observed	Mr(expt)	Mr(calc)	ppm	Miss	Score	Expect	Rank	Unique	Peptide
7527	445.2400	888.4654	888.4739	-9.49	0	51	0.0015	1	U	R.VALEACVK.A 11345

---

39. NbS00013071g0004.1 **Mass:** 11839 **Score:** 63 **Matches:** 1(1) **Sequences:** 1(1) **emPAI:** 0.29
NbS00013071g0004.1 protein AED:0.33 eAED:0.33 QI:0|-1|0|1|-1|1|1|0|106; (*SWP) sp|P27524|CB4A_SOLLC (e_value=3e-69) Chlorophyll a-b binding protein CP24 10A, chloroplastic OS=Solanum lycopersicum (

Query	Observed	Mr(expt)	Mr(calc)	ppm	Miss	Score	Expect	Rank	Unique	Peptide
18807	944.4308	1886.8470	1886.8435	1.86	0	63	1.9e-005	1	U	K.TAENFANFTGEQGYPGGK.F

Proteins matching the same set of peptides:
NbS00042109g0017.1 **Mass:** 29420 **Score:** 63 **Matches:** 1(1) **Sequences:** 1(1)
NbS00042109g0017.1 protein AED:0.10 eAED:0.10 QI:0|1|0.5|1|1|1|2|0|274; (*SWP) sp|P27524|CB4A_SOLLC (e_value=2e-160) Chlorophyll a-b binding protein CP24 10A, chloroplastic OS=Solanum lycopersicum
NbS00042109g0018.1 **Mass:** 27332 **Score:** 63 **Matches:** 1(1) **Sequences:** 1(1)
NbS00042109g0018.1 protein AED:0.00 eAED:0.00 QI:0|1|0.5|1|1|1|2|0|256; (*SWP) sp|P27524|CB4A_SOLLC (e_value=3e-163) Chlorophyll a-b binding protein CP24 10A, chloroplastic OS=Solanum lycopersicum

---

40. NbS00036430g0009.1 **Mass:** 48651 **Score:** 63 **Matches:** 2(2) **Sequences:** 2(2) **emPAI:** 0.14
NbS00036430g0009.1 protein AED:0.35 eAED:0.35 QI:0|0|0|1|0|0|4|0|448; (*GB) gi|113170490|ref|YP_717281.1| (e_value=2e-170) Atp1 [Ostreococcus tauri];; (*SWP) sp|B3PQ70|ATPA_RHIE6 (e_value=2e-158) A

Query	Observed	Mr(expt)	Mr(calc)	ppm	Miss	Score	Expect	Rank	Unique	Peptide
9503	595.3300	1188.6454	1188.6350	8.81	0	57	0.00029	1	U	R.AAELTSLLESR.I
14334	642.3551	1282.6957	1282.6921	2.76	0	34	0.023	1		K.QPQYAPLPIEK.Q

---

41. sp|TRYP_PIG| **Mass:** 25078 **Score:** 62 **Matches:** 3(3) **Sequences:** 1(1) **emPAI:** 0.13
sp|TRYP_PIG|

Query	Observed	Mr(expt)	Mr(calc)	ppm	Miss	Score	Expect	Rank	Unique	Peptide
11056	421.7581	841.5016	841.5022	-0.63	0	55	0.00014	1	U	R.VATVSLPR.S 11060 11062

Proteins matching the same set of peptides:
gi|3318722|pdb|1AN1|E **Mass:** 24142 **Score:** 62 **Matches:** 3(3) **Sequences:** 1(1)
gi|3318722|pdb|1AN1|E Chain E, Leech-Derived Tryptase InhibitorTRYPSIN COMPLEX

---

42. NbS00000634g0101.1 **Mass:** 15316 **Score:** 62 **Matches:** 5(5) **Sequences:** 2(2) **emPAI:** 0.49
NbS00000634g0101.1 protein AED:0.21 eAED:0.21 QI:0|-1|0|1|-1|1|1|0|136

Query	Observed	Mr(expt)	Mr(calc)	ppm	Miss	Score	Expect	Rank	Unique	Peptide
10994	416.2498	830.4851	830.4861	-1.21	0	54	0.00029	1	U	K.STELLIR.K 10997 10998
12279	339.5380	1015.5922	1015.5927	-0.53	0	30	0.051	1	U	R.FRPGTVALR.E 12285

Proteins matching the same set of peptides:
NbS00002567g0001.1 **Mass:** 15316 **Score:** 62 **Matches:** 5(5) **Sequences:** 2(2)
NbS00002567g0001.1 protein AED:0.20 eAED:0.20 QI:0|-1|0|1|-1|1|1|0|136; (*GB) gi|300681467|emb|CBH32561.1| (e_value=8e-79) histone H3, expressed [Triticum aestivum];; (*SWP) sp|P68428|H32_WHEAT (e_
NbS00006262g0002.1 **Mass:** 8846 **Score:** 62 **Matches:** 5(5) **Sequences:** 2(2)

NbS00006262g0002.1 protein AED:0.31 eAED:0.39 QI:0|0|0|1|1|1|2|0|79; (*GB) gi|222631727|gb|EEE63859.1| (e_value=1e-42) hypothetical protein OsJ_18683 [Oryza sativa Japonica Group];; (*SWP) sp|Q402I
NbS00007322g0001.1 **Mass:** 15316 **Score:** 62 **Matches:** 5(5) **Sequences:** 2(2)
NbS00007322g0001.1 protein AED:0.09 eAED:0.09 QI:0|1|0.5|1|0|0|2|203|136; (*GB) gi|300681467|emb|CBH32561.1| (e_value=8e-79) histone H3, expressed [Triticum aestivum];; (*SWP) sp|P68428|H32_WHEAT
NbS00009503g0001.1 **Mass:** 15316 **Score:** 62 **Matches:** 5(5) **Sequences:** 2(2)
NbS00009503g0001.1 protein AED:0.19 eAED:0.19 QI:0|-1|0|1|-1|1|1|0|136; (*GB) gi|300681467|emb|CBH32561.1| (e_value=8e-79) histone H3, expressed [Triticum aestivum];; (*SWP) sp|P68428|H32_WHEAT (e_
NbS00010093g0001.1 **Mass:** 15316 **Score:** 62 **Matches:** 5(5) **Sequences:** 2(2)
NbS00010093g0001.1 protein AED:0.19 eAED:0.19 QI:0|-1|0|1|-1|1|1|0|136; (*SWP) sp|P68428|H32_WHEAT (e_value=3e-80) Histone H3.2 OS=Triticum aestivum PE=1 SV=2;; (*TAIR) AT5G65360.1 (e_value=2e-81)
NbS00014992g0013.1 **Mass:** 56465 **Score:** 62 **Matches:** 5(5) **Sequences:** 2(2)
NbS00014992g0013.1 protein AED:0.12 eAED:0.14 QI:0|0|0|0.66|1|1|3|0|505; (*GB) gi|351722020|ref|NP_001238509.1| (e_value=2e-72) uncharacterized protein LOC100306416 [Glycine max];; (*SWP) sp|P6842
NbS00019233g0001.1 **Mass:** 62065 **Score:** 62 **Matches:** 5(5) **Sequences:** 2(2)
NbS00019233g0001.1 protein AED:0.09 eAED:0.09 QI:0|0|0|1|1|1|2|0|553; (*GB) gi|222631727|gb|EEE63859.1| (e_value=2e-40) hypothetical protein OsJ_18683 [Oryza sativa Japonica Group];; (*SWP) sp|Q76N
NbS00020349g0004.1 **Mass:** 14009 **Score:** 62 **Matches:** 5(5) **Sequences:** 2(2)
NbS00020349g0004.1 protein AED:0.03 eAED:0.03 QI:0|-1|0|1|-1|1|1|0|125; (*SWP) sp|P68428|H32_WHEAT (e_value=1e-72) Histone H3.2 OS=Triticum aestivum PE=1 SV=2;; (*TAIR) AT5G65360.1 (e_value=1e-73)
NbS00020781g0004.1 **Mass:** 8022 **Score:** 62 **Matches:** 5(5) **Sequences:** 2(2)
NbS00020781g0004.1 protein AED:0.32 eAED:0.34 QI:0|0|0|1|1|1|2|0|72; (*GB) gi|308081499|ref|NP_001183546.1| (e_value=2e-42) uncharacterized protein LOC100502113 [Zea mays];; (*SWP) sp|Q402E1|H32_L
NbS00026286g0015.1 **Mass:** 20246 **Score:** 62 **Matches:** 5(5) **Sequences:** 2(2)
NbS00026286g0015.1 protein AED:0.06 eAED:0.06 QI:0|0|0|1|0|0|2|0|178; (*GB) gi|156350379|ref|XP_001622258.1| (e_value=8e-77) predicted protein [Nematostella vectensis];; (*SWP) sp|P68428|H32_WHEAT

---

43. NbS00042812g0008.1 **Mass:** 20462 **Score:** 61 **Matches:** 2(1) **Sequences:** 2(1) **emPAI:** 0.36
NbS00042812g0008.1 protein AED:0.19 eAED:0.19 QI:0|1|1|1|1|1|4|386|180; (*GB) gi|132118|sp|P26573.1|RBS8_NICPL (e_value=3e-119) RecName: Full=Ribulose bisphosphate carboxylase small chain 8B, chlo

Query	Observed	Mr(expt)	Mr(calc)	ppm	Miss	Score	Expect	Rank	Unique	Peptide
11649	467.2611	932.5077	932.5080	-0.32	0	60	9.3e-005	1		R.IIGFDNVR.Q
12514	**524.7640**	**1047.5134**	**1047.5138**	**-0.34**	**0**	**30**	**0.081**	**1**	**U**	**K.EYPQAWVR.I**

Proteins matching the same set of peptides:
NbC25292626g0004.1 **Mass:** 18446 **Score:** 61 **Matches:** 2(1) **Sequences:** 2(1)
NbC25292626g0004.1 protein AED:0.08 eAED:0.08 QI:0|1|0.5|1|1|1|4|0|159; (*GB) gi|132118|sp|P26573.1|RBS8_NICPL (e_value=2e-112) RecName: Full=Ribulose bisphosphate carboxylase small chain 8B, chlo

---

44. NbS00018705g0023.1 **Mass:** 7957 **Score:** 58 **Matches:** 2(2) **Sequences:** 2(2) **emPAI:** 1.08
NbS00018705g0023.1 protein AED:0.30 eAED:0.31 QI:0|0.66|0.75|1|0.33|0.75|4|388|71; (*GB) gi|242050850|ref|XP_002463169.1| (e_value=7e-39) hypothetical protein SORBIDRAFT_02g039000 [Sorghum bicolor]

Query	Observed	Mr(expt)	Mr(calc)	ppm	Miss	Score	Expect	Rank	Unique	Peptide
10524	**379.2514**	**756.4883**	**756.4858**	**3.27**	**0**	**39**	**0.0054**	**1**	**U**	**R.LVVGTLR.G**
16428	**777.9285**	**1553.8424**	**1553.8413**	**0.69**	**0**	**48**	**0.00066**	**1**	**U**	**R.GNSVVTIEALEPVAR.A**

Proteins matching the same set of peptides:
NbS00024371g0006.1 **Mass:** 8908 **Score:** 58 **Matches:** 2(2) **Sequences:** 2(2)
NbS00024371g0006.1 protein AED:0.00 eAED:0.00 QI:102|1|1|1|1|1|4|366|80; (*GB) gi|242050850|ref|XP_002463169.1| (e_value=4e-48) hypothetical protein SORBIDRAFT_02g039000 [Sorghum bicolor];; (*SWP)

---

45. NbS00019305g0027.1 **Mass:** 59772 **Score:** 57 **Matches:** 2(2) **Sequences:** 1(1) **emPAI:** 0.06
NbS00019305g0027.1 protein AED:0.37 eAED:0.37 QI:0|1|0.88|1|1|1|9|0|556; (*GB) gi|3676296|gb|AAD03392.1| (e_value=0.0) mitochondrial ATPase beta subunit [Nicotiana sylvestris];; (*SWP) sp|P17614|A

Query	Observed	Mr(expt)	Mr(calc)	ppm	Miss	Score	Expect	Rank	Unique	Peptide
11969	**488.2846**	**974.5546**	**974.5549**	**-0.34**	**0**	**54**	**0.00027**	**1**	**U**	**K.IGLFGGAGVGK.T** 11970

Proteins matching the same set of peptides:
gi|297692195|ref|XP_002823453.1| **Mass:** 62271 **Score:** 57 **Matches:** 2(2) **Sequences:** 1(1)
gi|297692195|ref|XP_002823453.1| PREDICTED: LOW QUALITY PROTEIN: ATP synthase subunit beta, mitochondrial-like [Pongo abelii]
NbS00020973g0010.1 **Mass:** 58453 **Score:** 57 **Matches:** 2(2) **Sequences:** 1(1)
NbS00020973g0010.1 protein AED:0.23 eAED:0.23 QI:0|0.87|0.77|1|0.87|1|9|0|545; (*GB) gi|3676296|gb|AAD03392.1| (e_value=0.0) mitochondrial ATPase beta subunit [Nicotiana sylvestris];; (*SWP) sp|P1

---

46. NbS00003075g0011.1 **Mass:** 15245 **Score:** 57 **Matches:** 2(2) **Sequences:** 2(2) **emPAI:** 0.50
NbS00003075g0011.1 protein AED:0.28 eAED:0.28 QI:0|1|0.66|1|1|1|3|0|143; (*GB) gi|2499967|sp|Q41229.1|PSAEB_NICSY (e_value=1e-59) RecName: Full=Photosystem I reaction center subunit IV B, chloropl

Query	Observed	Mr(expt)	Mr(calc)	ppm	Miss	Score	Expect	Rank	Unique	Peptide
15981	**757.3847**	**1512.7549**	**1512.7533**	**1.05**	**0**	**50**	**0.00066**	**1**	**U**	**K.GVGSVVAVDQDPNTR.Y**
20216	**743.0668**	**2226.1785**	**2226.1757**	**1.24**	**1**	**35**	**0.014**	**1**	**U**	**K.GVGSVVAVDQDPNTRYPVVVR.F**

Proteins matching the same set of peptides:
NbS00019085g0006.1 **Mass:** 15263 **Score:** 57 **Matches:** 2(2) **Sequences:** 2(2)
NbS00019085g0006.1 protein AED:0.21 eAED:0.21 QI:205|1|1|1|1|1|3|512|143; (*GB) gi|2499967|sp|Q41229.1|PSAEB_NICSY (e_value=3e-57) RecName: Full=Photosystem I reaction center subunit IV B, chlorop

---

47. NbS00008430g0013.1 **Mass:** 124678 **Score:** 56 **Matches:** 4(2) **Sequences:** 4(2) **emPAI:** 0.08
NbS00008430g0013.1 protein AED:0.20 eAED:0.20 QI:6|0.90|0.86|0.95|0.90|0.95|22|289|1122; (*GB) gi|84688908|gb|ABC61503.1| (e_value=0.0) AGO1-2, partial [Nicotiana benthamiana];; (*SWP) sp|Q7XSA2|AG

Query	Observed	Mr(expt)	Mr(calc)	ppm	Miss	Score	Expect	Rank	Unique	Peptide
8674	517.2900	1032.5654	1032.5564	8.80	0	31	0.12	2		R.ISGLTSQATR.E
12819	544.3096	1086.6047	1086.6033	1.28	0	30	0.071	1	U	R.NTVLVDAISR.R
15372	710.8967	1419.7788	1419.7762	1.82	0	47	0.0011	1		K.SLYTAGPLPFVQK.D
15420	**714.8525**	**1427.6904**	**1427.6892**	**0.82**	**0**	**32**	**0.041**	**1**	**U**	**K.ITLIDDEDGPGGAR.R**

Proteins matching the same set of peptides:
NbS00014977g0008.1 **Mass:** 125165 **Score:** 56 **Matches:** 4(2) **Sequences:** 4(2)
NbS00014977g0008.1 protein AED:0.16 eAED:0.16 QI:16|0.95|0.90|1|0.95|0.90|22|432|1125; (*GB) gi|84688908|gb|ABC61503.1| (e_value=0.0) AGO1-2, partial [Nicotiana benthamiana];; (*SWP) sp|Q7XSA2|AGO

---

48. NbS00001559g0021.1 **Mass:** 90961 **Score:** 56 **Matches:** 2(2) **Sequences:** 2(2) **emPAI:** 0.07
NbS00001559g0021.1 protein AED:0.25 eAED:0.31 QI:138|0.5|0.53|1|0.85|0.93|15|300|837; (*GB) gi|356513635|ref|XP_003525517.1| (e_value=0.0) PREDICTED: DEAD-box ATP-dependent RNA helicase 30-like [G

Query	Observed	Mr(expt)	Mr(calc)	ppm	Miss	Score	Expect	Rank	Unique	Peptide
13957	**622.8369**	**1243.6592**	**1243.6594**	**-0.23**	**0**	**35**	**0.028**	**1**	**U**	**R.SPIMIATDVAAR.G**
15491	**719.8532**	**1437.6919**	**1437.6922**	**-0.23**	**0**	**44**	**0.0027**	**1**	**U**	**R.ANISSTCIYGGAPK.G**

---

49. NbS00011860g0002.1 **Mass:** 87858 **Score:** 56 **Matches:** 2(2) **Sequences:** 2(2) **emPAI:** 0.08
NbS00011860g0002.1 protein AED:0.14 eAED:0.14 QI:0|0.83|0.76|0.84|1|1|13|0|788; (*GB) gi|255561268|ref|XP_002521645.1| (e_value=0.0) arsenite-resistance protein, putative [Ricinus communis];; (*SW

Query	Observed	Mr(expt)	Mr(calc)	ppm	Miss	Score	Expect	Rank	Unique	Peptide
15178	**697.8053**	**1393.5960**	**1393.5939**	**1.56**	**0**	**33**	**0.014**	**1**	**U**	**R.FGYDYQPGYER.E**
15195	**698.8989**	**1395.7832**	**1395.7834**	**-0.17**	**0**	**44**	**0.0015**	**1**	**U**	**R.VQIDVGQAQALVR.K**

---

50. NbS00044448g0012.1 **Mass:** 82518 **Score:** 56 **Matches:** 2(2) **Sequences:** 2(2) **emPAI:** 0.08
NbS00044448g0012.1 protein AED:0.24 eAED:0.25 QI:232|0.85|0.87|1|1|1|8|0|762; (*GB) gi|194396261|gb|ACF60500.1| (e_value=0.0) plastid transketolase [Nicotiana tabacum];; (*SWP) sp|Q43848|TKTC_SOLT

Query	Observed	Mr(expt)	Mr(calc)	ppm	Miss	Score	Expect	Rank	Unique	Peptide
14577	**657.3630**	**1312.7115**	**1312.7099**	**1.26**	**0**	**45**	**0.0019**	**1**	**U**	**R.NLSQQNLNALAK.V**
15122	**693.8679**	**1385.7212**	**1385.7191**	**1.51**	**0**	**33**	**0.031**	**1**	**U**	**K.SIVTGELPAGWEK.A**

---

51. NbS00020748g0007.1 **Mass:** 61062 **Score:** 55 **Matches:** 1(1) **Sequences:** 1(1) **emPAI:** 0.05
NbS00020748g0007.1 protein AED:0.36 eAED:0.37 QI:0|0.64|0.66|0.86|0.85|0.8|15|0|555; (*GB) gi|587562|emb|CAA56520.1| (e_value=0.0) mitochondrial processing peptidase [Solanum tuberosum];; (*SWP) sp

Query	Observed	Mr(expt)	Mr(calc)	ppm	Miss	Score	Expect	Rank	Unique	Peptide
13152	**567.3027**	**1132.5909**	**1132.5910**	**-0.08**	**0**	**55**	**0.0003**	**1**	**U**	**K.SAILMNLESR.M**

Proteins matching the same set of peptides:
NbS00020749g0011.1 **Mass:** 56674 **Score:** 55 **Matches:** 1(1) **Sequences:** 1(1)
NbS00020749g0011.1 protein AED:0.27 eAED:0.29 QI:0|0.85|0.73|0.93|0.92|0.8|15|608|521; (*GB) gi|587562|emb|CAA56520.1| (e_value=0.0) mitochondrial processing peptidase [Solanum tuberosum];; (*SWP)
NbS00027821g0012.1 **Mass:** 54872 **Score:** 55 **Matches:** 1(1) **Sequences:** 1(1)
NbS00027821g0012.1 protein AED:0.20 eAED:0.20 QI:139|0.91|0.92|1|0.75|0.76|13|354|504; (*GB) gi|266567|sp|P29677.1|MPPA_SOLTU (e_value=0.0) RecName: Full=Mitochondrial-processing peptidase subunit
NbS00033159g0010.1 **Mass:** 47264 **Score:** 55 **Matches:** 1(1) **Sequences:** 1(1)
NbS00033159g0010.1 protein AED:0.39 eAED:0.39 QI:136|0.81|0.91|1|0.81|0.83|12|0|433; (*GB) gi|266567|sp|P29677.1|MPPA_SOLTU (e_value=0.0) RecName: Full=Mitochondrial-processing peptidase subunit a

---

52. NbS00019623g0001.1 **Mass:** 49575 **Score:** 55 **Matches:** 1(1) **Sequences:** 1(1) **emPAI:** 0.07
NbS00019623g0001.1 protein AED:0.21 eAED:0.21 QI:0|1|0.5|1|1|1|2|0|447; (*GB) gi|3869088|dbj|BAA34348.1| (e_value=0.0) elongation factor-1 alpha [Nicotiana paniculata];; (*SWP) sp|P17786|EF1A_SOLC

Query	Observed	Mr(expt)	Mr(calc)	ppm	Miss	Score	Expect	Rank	Unique	Peptide
12353	**513.3084**	**1024.6023**	**1024.6030**	**-0.66**	**0**	**55**	**0.00011**	**1**	**U**	**K.IGGIGTVPVGR.V**

Proteins matching the same set of peptides:
NbS00023178g0001.1 **Mass:** 45458 **Score:** 55 **Matches:** 1(1) **Sequences:** 1(1)

NbS00023178g0001.1 protein AED:0.03 eAED:0.03 QI:0|0|0|1|1|1|3|0|413; (*SWP) sp|Q40034|EF1A2_HORVU (e_value=0.0) Elongation factor 1-alpha OS=Hordeum vulgare GN=BLT63 PE=1 SV=1;; (*TAIR) AT5G60390.
NbS00024768g0013.1 **Mass:** 49347 **Score:** 55 **Matches:** 1(1) **Sequences:** 1(1)
NbS00024768g0013.1 protein AED:0.17 eAED:0.19 QI:3|0.33|0|1|0.66|0.75|4|0|443; (*GB) gi|3869088|dbj|BAA34348.1| (e_value=0.0) elongation factor-1 alpha [Nicotiana paniculata];; (*SWP) sp|P17786|EF
NbS00026055g0002.1 **Mass:** 40879 **Score:** 55 **Matches:** 1(1) **Sequences:** 1(1)
NbS00026055g0002.1 protein AED:0.04 eAED:0.06 QI:0|-1|0|1|-1|1|1|0|373; (*GB) gi|1864017|dbj|BAA09709.1| (e_value=0.0) elongation factor-1 alpha [Nicotiana tabacum];; (*SWP) sp|P43643|EF1A_TOBAC (e
NbS00037026g0004.1 **Mass:** 49587 **Score:** 55 **Matches:** 1(1) **Sequences:** 1(1)
NbS00037026g0004.1 protein AED:0.17 eAED:0.17 QI:0|1|0.5|1|1|1|2|0|447; (*GB) gi|3869088|dbj|BAA34348.1| (e_value=0.0) elongation factor-1 alpha [Nicotiana paniculata];; (*SWP) sp|P43643|EF1A_TOBAC
NbS00060738g0002.1 **Mass:** 50846 **Score:** 55 **Matches:** 1(1) **Sequences:** 1(1)
NbS00060738g0002.1 protein AED:0.07 eAED:0.09 QI:0|0|0.33|0.25|1|1|1|4|0|458; (*SWP) sp|P43643|EF1A_TOBAC (e_value=0.0) Elongation factor 1-alpha OS=Nicotiana tabacum PE=2 SV=1;; (*TAIR) AT5G60390.3
gi|311263698|ref|XP_003129826.1| **Mass:** 50519 **Score:** 55 **Matches:** 1(1) **Sequences:** 1(1)
gi|311263698|ref|XP_003129826.1| PREDICTED: elongation factor 1-alpha, oocyte form-like [Sus scrofa]
NbS00007372g0013.1 **Mass:** 54761 **Score:** 55 **Matches:** 1(1) **Sequences:** 1(1)
NbS00007372g0013.1 protein AED:0.21 eAED:0.21 QI:0|0|0|1|0.5|0.66|3|0|494; (*GB) gi|1864017|dbj|BAA09709.1| (e_value=0.0) elongation factor-1 alpha [Nicotiana tabacum];; (*SWP) sp|P43643|EF1A_TOBAC
NbS00024768g0017.1 **Mass:** 101076 **Score:** 55 **Matches:** 1(1) **Sequences:** 1(1)
NbS00024768g0017.1 protein AED:0.14 eAED:0.15 QI:0|0|0.33|1|0|0|6|0|911; (*GB) gi|297849086|ref|XP_002892424.1| (e_value=0.0) T6D22.2 [Arabidopsis lyrata subsp. lyrata];; (*SWP) sp|P17786|EF1A_SOLI

---

53. NbS00001988g0005.1 **Mass:** 66344 **Score:** 54 **Matches:** 2(2) **Sequences:** 2(2) **emPAI:** 0.10
NbS00001988g0005.1 protein AED:0.30 eAED:0.30 QI:0|1|0.83|1|1|1|6|0|610; (*GB) gi|225441549|ref|XP_002281113.1| (e_value=0.0) PREDICTED: DEAD-box ATP-dependent RNA helicase 37-like [Vitis vinifera]

Query	Observed	Mr(expt)	Mr(calc)	ppm	Miss	Score	Expect	Rank	Unique	Peptide
16571	792.9464	1583.8783	1583.8784	-0.06	0	44	0.0016	1	U	R.VVVAYGGAPINQQLR.E
20051	716.0070	2144.9990	2144.9909	3.79	0	33	0.02	1	U	R.NKPMGAEPPAPSPGGPPSGNDR.S

---

54. NbS00000681g0006.1 **Mass:** 33521 **Score:** 54 **Matches:** 2(2) **Sequences:** 2(2) **emPAI:** 0.21
NbS00000681g0006.1 protein AED:0.29 eAED:0.30 QI:89|0.75|0.88|1|0.75|0.77|9|282|301; (*GB) gi|358248282|ref|NP_001239854.1| (e_value=7e-62) uncharacterized protein LOC100818986 [Glycine max];; (*T

Query	Observed	Mr(expt)	Mr(calc)	ppm	Miss	Score	Expect	Rank	Unique	Peptide
14044	628.8276	1255.6407	1255.6408	-0.07	0	44	0.0029	1	U	R.VETPIDANEIR.I
17229	827.4342	1652.8539	1652.8522	1.00	0	38	0.01	1	U	K.NNIGYQPPIPADQVK.V

Proteins matching the same set of peptides:
NbS00014238g0013.1 **Mass:** 27940 **Score:** 54 **Matches:** 2(2) **Sequences:** 2(2)
NbS00014238g0013.1 protein AED:0.37 eAED:0.37 QI:89|1|1|1|0.87|0.88|9|404|254; (*GB) gi|358248282|ref|NP_001239854.1| (e_value=1e-64) uncharacterized protein LOC100818986 [Glycine max];; (*SWP) sp

---

55. NbS00035152g0024.1 **Mass:** 73867 **Score:** 53 **Matches:** 2(2) **Sequences:** 2(2) **emPAI:** 0.09
NbS00035152g0024.1 protein AED:0.26 eAED:0.26 QI:0|1|0.92|1|0.69|0.78|14|405|631; (*GB) gi|359494535|ref|XP_002266085.2| (e_value=0.0) PREDICTED: peptidyl-prolyl cis-trans isomerase-like 4-like [V

Query	Observed	Mr(expt)	Mr(calc)	ppm	Miss	Score	Expect	Rank	Unique	Peptide
12758	540.7633	1079.5121	1079.5135	-1.31	0	36	0.017	1	U	R.INEAYVDEK.G
13602	597.3306	1192.6466	1192.6452	1.17	0	44	0.0028	1	U	R.FGTVTSAEIIR.D

---

56. NbS00027428g0011.1 **Mass:** 84144 **Score:** 53 **Matches:** 1(1) **Sequences:** 1(1) **emPAI:** 0.04
NbS00027428g0011.1 protein AED:0.20 eAED:0.20 QI:0|0|0|1|1|1|2|0|723; (*GB) gi|359475106|ref|XP_003631587.1| (e_value=0.0) PREDICTED: DEAD-box ATP-dependent RNA helicase 21-like [Vitis vinifera];;

Query	Observed	Mr(expt)	Mr(calc)	ppm	Miss	Score	Expect	Rank	Unique	Peptide
13297	578.3405	1154.6664	1154.6659	0.36	0	53	0.00019	1	U	R.NPVVVTIGTAGK.T

Proteins matching the same set of peptides:
NbS00035002g0004.1 **Mass:** 83277 **Score:** 53 **Matches:** 1(1) **Sequences:** 1(1)
NbS00035002g0004.1 protein AED:0.32 eAED:0.32 QI:0|-1|0|1|-1|1|1|0|715; (*GB) gi|359475106|ref|XP_003631587.1| (e_value=0.0) PREDICTED: DEAD-box ATP-dependent RNA helicase 21-like [Vitis vinifera];

---

57. NbS00015934g0007.1 **Mass:** 67076 **Score:** 53 **Matches:** 1(1) **Sequences:** 1(1) **emPAI:** 0.05
NbS00015934g0007.1 protein AED:0.16 eAED:0.16 QI:0|0.66|0.28|1|0.5|0.57|7|0|612; (*GB) gi|255576310|ref|XP_002529048.1| (e_value=0.0) WRKY transcription factor, putative [Ricinus communis];; (*SWP)

Query	Observed	Mr(expt)	Mr(calc)	ppm	Miss	Score	Expect	Rank	Unique	Peptide
13817	613.8585	1225.7025	1225.7216	-15.62	1	53	0.00018	1	U	K.LMTPAKLPISR.S

Proteins matching the same set of peptides:
NbS00026917g0001.1 **Mass:** 65836 **Score:** 53 **Matches:** 1(1) **Sequences:** 1(1)

NbS00026917g0001.1 protein AED:0.26 eAED:0.26 QI:0|0.66|0.42|1|1|1|7|0|602; (*GB) gi|224056431|ref|XP_002298853.1| (e_value=0.0) predicted protein [Populus trichocarpa];; (*SWP) sp|Q93WV0|WRK20_AR

---

58. NbS00004739g0001.1 **Mass:** 27728 **Score:** 53 **Matches:** 1(1) **Sequences:** 1(1) **emPAI:** 0.12
NbS00004739g0001.1 protein AED:0.17 eAED:0.17 QI:0|1|0.66|1|1|1|3|0|251; (*GB) gi|100801744|emb|CAK24966.1| (e_value=2e-175) chlorophyll a/b binding protein [Solanum tuberosum];; (*SWP) sp|Q9SQL2|C

Query	Observed	Mr(expt)	Mr(calc)	ppm	Miss	Score	Expect	Rank	Unique	Peptide
14593	658.3359	1314.6572	1314.6568	0.29	0	53	0.00038	1	U	K.NPGSVNQDPIFK.N

Proteins matching the same set of peptides:
NbS00030208g0012.1 **Mass:** 25902 **Score:** 53 **Matches:** 1(1) **Sequences:** 1(1)
NbS00030208g0012.1 protein AED:0.05 eAED:0.05 QI:0|0.5|0|1|1|1|3|0|233; (*GB) gi|100801744|emb|CAK24966.1| (e_value=1e-158) chlorophyll a/b binding protein [Solanum tuberosum];; (*SWP) sp|Q9SQL2|CH
NbS00043913g0001.1 **Mass:** 27579 **Score:** 53 **Matches:** 1(1) **Sequences:** 1(1)
NbS00043913g0001.1 protein AED:0.36 eAED:0.36 QI:170|1|0.66|1|1|1|3|0|250; (*GB) gi|100801744|emb|CAK24966.1| (e_value=7e-166) chlorophyll a/b binding protein [Solanum tuberosum];; (*SWP) sp|P2752
NbS00052312g0004.1 **Mass:** 27591 **Score:** 53 **Matches:** 1(1) **Sequences:** 1(1)
NbS00052312g0004.1 protein AED:0.28 eAED:0.28 QI:166|1|0.66|1|0.5|0.66|3|0|250; (*SWP) sp|P27521|CA4_ARATH (e_value=1e-154) Chlorophyll a-b binding protein 4, chloroplastic OS=Arabidopsis thaliana

---

59. NbS00011643g0015.1 **Mass:** 10334 **Score:** 52 **Matches:** 1(1) **Sequences:** 1(1) **emPAI:** 0.34
NbS00011643g0015.1 protein AED:0.07 eAED:0.07 QI:151|1|1|1|0.8|0.83|6|432|88; (*GB) gi|356508935|ref|XP_003523208.1| (e_value=2e-56) PREDICTED: small nuclear ribonucleoprotein E-like [Glycine max];

Query	Observed	Mr(expt)	Mr(calc)	ppm	Miss	Score	Expect	Rank	Unique	Peptide
14825	673.3870	1344.7595	1344.7588	0.55	0	52	0.00027	1	U	R.IMTQPINLIFR.F

Proteins matching the same set of peptides:
NbS00034990g0011.1 **Mass:** 14072 **Score:** 52 **Matches:** 1(1) **Sequences:** 1(1)
NbS00034990g0011.1 protein AED:0.13 eAED:0.13 QI:153|0.8|0.83|1|0.8|0.66|6|389|118; (*GB) gi|356562483|ref|XP_003549500.1| (e_value=1e-53) PREDICTED: small nuclear ribonucleoprotein E [Glycine max]
NbS00056919g0001.1 **Mass:** 9341 **Score:** 52 **Matches:** 1(1) **Sequences:** 1(1)
NbS00056919g0001.1 protein AED:0.17 eAED:0.17 QI:148|0.8|0.83|1|0.8|0.83|6|459|79; (*SWP) sp|A1XQR9|RUXE_PIG (e_value=1e-33) Small nuclear ribonucleoprotein E OS=Sus scrofa GN=SNRPE PE=3 SV=1;; (*

---

60. NbS00030061g0007.1 **Mass:** 40220 **Score:** 52 **Matches:** 1(1) **Sequences:** 1(1) **emPAI:** 0.08
NbS00030061g0007.1 protein AED:0.13 eAED:0.13 QI:243|1|1|1|0.85|0.75|8|1441|373; (*GB) gi|77416977|gb|ABA81884.1| (e_value=7e-108) nuclear RNA binding protein-like [Solanum tuberosum];; (*TAIR) AT

Query	Observed	Mr(expt)	Mr(calc)	ppm	Miss	Score	Expect	Rank	Unique	Peptide
19724	997.4975	1992.9804	1992.9792	0.60	0	52	0.00034	1	U	K.NYSAPSIEDAGQFPSLVAK.-

---

61. NbS00023006g0207.1 **Mass:** 9109 **Score:** 52 **Matches:** 2(2) **Sequences:** 1(1) **emPAI:** 0.38
NbS00023006g0207.1 protein

Query	Observed	Mr(expt)	Mr(calc)	ppm	Miss	Score	Expect	Rank	Unique	Peptide
12501	523.3063	1044.5981	1044.5968	1.29	0	41	0.0049	1	U	K.VVDLLAPYR.R 12500

---

62. NbS00001594g0015.1 **Mass:** 62334 **Score:** 52 **Matches:** 2(2) **Sequences:** 2(2) **emPAI:** 0.11
NbS00001594g0015.1 protein AED:0.04 eAED:0.04 QI:64|0.5|0.6|1|0.75|0.6|5|0|553; (*GB) gi|268619136|gb|ACZ13344.1| (e_value=0.0) tubulin alpha chain [Bursaphelenchus xylophilus];; (*SWP) sp|Q9ZRR5|

Query	Observed	Mr(expt)	Mr(calc)	ppm	Miss	Score	Expect	Rank	Unique	Peptide
12171	501.2855	1000.5564	1000.5553	1.13	0	42	0.0045	1	U	K.DVNAAVATIK.T
17818	851.4575	1700.9004	1700.8985	1.09	0	32	0.038	1	U	R.AVFVDLEPTVIDEVR.T

Proteins matching the same set of peptides:
NbS00003471g0210.1 **Mass:** 46317 **Score:** 52 **Matches:** 2(2) **Sequences:** 2(2)
NbS00003471g0210.1 protein AED:0.09 eAED:0.09 QI:260|1|0.75|1|0.66|0.5|4|0|414
NbS00006458g0003.1 **Mass:** 55859 **Score:** 52 **Matches:** 2(2) **Sequences:** 2(2)
NbS00006458g0003.1 protein AED:0.07 eAED:0.07 QI:12|0.33|0.25|1|0.66|0.5|4|0|500; (*GB) gi|17402471|emb|CAD13178.1| (e_value=0.0) alpha-tubulin [Nicotiana tabacum];; (*SWP) sp|P33629|TBA_PRUDU (e_
NbS00031544g0010.1 **Mass:** 50360 **Score:** 52 **Matches:** 2(2) **Sequences:** 2(2)
NbS00031544g0010.1 protein AED:0.10 eAED:0.10 QI:4|1|1|1|1|1|4|217|450; (*GB) gi|386870485|gb|AFJ42573.1| (e_value=0.0) alpha-tubulin [Sesamum indicum];; (*SWP) sp|P33629|TBA_PRUDU (e_value=0.0) Tu
NbS00038051g0004.1 **Mass:** 55743 **Score:** 52 **Matches:** 2(2) **Sequences:** 2(2)
NbS00038051g0004.1 protein AED:0.04 eAED:0.04 QI:0|0|0|1|1|1|3|0|493; (*GB) gi|17402469|emb|CAD13177.1| (e_value=0.0) alpha-tubulin [Nicotiana tabacum];; (*SWP) sp|P33629|TBA_PRUDU (e_value=0.0) Tu
NbS00058547g0002.1 **Mass:** 58434 **Score:** 52 **Matches:** 2(2) **Sequences:** 2(2)
NbS00058547g0002.1 protein AED:0.08 eAED:0.08 QI:4|0.75|0.4|1|1|1|5|0|526; (*GB) gi|348515729|ref|XP_003445392.1| (e_value=0.0) PREDICTED: tubulin alpha chain-like [Oreochromis niloticus];; (*SWP)

---

63. NbS00001525g0121.1 **Mass:** 62315 **Score:** 51 **Matches:** 1(1) **Sequences:** 1(1) **emPAI:** 0.05
NbS00001525g0121.1 protein AED:0.18 eAED:0.18 QI:3|0.62|0.22|1|0.75|0.77|9|0|561

Query	Observed	Mr(expt)	Mr(calc)	ppm	Miss	Score	Expect	Rank	Unique	Peptide
17072	816.3558	1630.6970	1630.6933	2.26	0	51	0.00021	1	U	R.CYESYSEDPNIVR.A

Proteins matching the same set of peptides:
NbS00017675g0018.1 **Mass:** 79118 **Score:** 51 **Matches:** 1(1) **Sequences:** 1(1)
NbS00017675g0018.1 protein AED:0.22 eAED:0.23 QI:3|0.44|0.2|1|0.77|0.8|10|0|714; (*GB) gi|356565758|ref|XP_003551104.1| (e_value=0.0) PREDICTED: lysosomal beta glucosidase-like [Glycine max];; (*SW

64. >gi|74181742|dbj|BAE32582.1| **Mass:** 55528 **Score:** 51 **Matches:** 3(2) **Sequences:** 3(2) **emPAI:** 0.19
>gi|74181742|dbj|BAE32582.1| unnamed protein product [Mus musculus]

Query	Observed	Mr(expt)	Mr(calc)	ppm	Miss	Score	Expect	Rank	Unique	Peptide
12480	522.2899	1042.5652	1042.5659	-0.70	0	32	0.052	1	U	R.TGSIVDVPAGK.A
14334	642.3551	1282.6957	1282.6921	2.76	0	34	0.023	1		K.QPQYAPLPIEK.Q
14461	650.8796	1299.7446	1299.7398	3.70	0	37	0.0091	1	U	K.TAIAIDTILNQK.Q

65. NbS00009714g0011.1 **Mass:** 45483 **Score:** 50 **Matches:** 2(2) **Sequences:** 2(2) **emPAI:** 0.15
NbS00009714g0011.1 protein AED:0.20 eAED:0.20 QI:131|0.83|0.71|1|0.83|0.57|7|463|410; (*SWP) sp|Q40565|RCA2_TOBAC (e_value=0.0) Ribulose bisphosphate carboxylase/oxygenase activase 2, chloroplastic

Query	Observed	Mr(expt)	Mr(calc)	ppm	Miss	Score	Expect	Rank	Unique	Peptide
9906	666.8400	1331.6654	1331.6721	-5.02	0	38	0.012	1	U	K.WVSGTGIEAIGDK.L
18044	868.9095	1735.8044	1735.8013	1.78	0	40	0.0051	1	U	K.GLVQDFSDDQQDITR.G

Proteins matching the same set of peptides:
NbS00047700g0013.1 **Mass:** 81330 **Score:** 50 **Matches:** 2(2) **Sequences:** 2(2)
NbS00047700g0013.1 protein AED:0.19 eAED:0.19 QI:131|0.6|0.63|1|0.8|0.54|11|307|726; (*GB) gi|12643758|sp|Q40565.1|RCA2_TOBAC (e_value=0.0) RecName: Full=Ribulose bisphosphate carboxylase/oxygenase

66. NbS00010545g0303.1 **Mass:** 17050 **Score:** 48 **Matches:** 2(2) **Sequences:** 2(2) **emPAI:** 0.43
NbS00010545g0303.1 protein AED:0.34 eAED:0.34 QI:0|1|0.5|1|1|1|2|0|169

Query	Observed	Mr(expt)	Mr(calc)	ppm	Miss	Score	Expect	Rank	Unique	Peptide
13664	602.2883	1202.5620	1202.5608	0.97	0	37	0.012	1	U	R.GFGFVTFGDEK.S
18170	879.4352	1756.8559	1756.8519	2.28	0	32	0.04	1	U	R.TLGEAFSQYGEVLESK.I

Proteins matching the same set of peptides:
NbS00027114g0020.1 **Mass:** 16680 **Score:** 48 **Matches:** 2(2) **Sequences:** 2(2)
NbS00027114g0020.1 protein AED:0.25 eAED:0.25 QI:0|0.5|0.33|0.66|0|0.33|3|0|161; (*GB) gi|469072|dbj|BAA03743.1| (e_value=9e-56) RNA-binding gricine-rich protein-1c [Nicotiana sylvestris];; (*SWP)

67. NbS00020769g0006.1 **Mass:** 26377 **Score:** 48 **Matches:** 2(2) **Sequences:** 2(2) **emPAI:** 0.27
NbS00020769g0006.1 protein AED:0.25 eAED:0.25 QI:307|0.66|1|1|0.66|0.75|4|448|250; (*GB) gi|84620804|gb|ABC59516.1| (e_value=5e-102) chloroplast photosystem II 22 kDa component [Nicotiana benthamia

Query	Observed	Mr(expt)	Mr(calc)	ppm	Miss	Score	Expect	Rank	Unique	Peptide
15334	708.3570	1414.6994	1414.6980	1.00	0	42	0.0043	1	U	K.FIDDPTPPTGLDK.A
16565	792.8965	1583.7784	1583.7832	-3.00	0	32	0.038	1		K.VEDGIFGTSGGIGFTK.Q

68. NbS00004213g0104.1 **Mass:** 14742 **Score:** 47 **Matches:** 2(2) **Sequences:** 2(2) **emPAI:** 0.51
NbS00004213g0104.1 protein AED:0.32 eAED:0.32 QI:0|-1|0|1|-1|1|1|0|132

Query	Observed	Mr(expt)	Mr(calc)	ppm	Miss	Score	Expect	Rank	Unique	Peptide
10353	368.6924	735.3703	735.3704	-0.18	0	43	0.0041	1		R.FWDLR.A
12707	536.2648	1070.5151	1070.5145	0.58	0	35	0.017	1	U	K.DIQPWQER.C

69. NbS00005183g0002.1 **Mass:** 64758 **Score:** 46 **Matches:** 2(2) **Sequences:** 2(2) **emPAI:** 0.10
NbS00005183g0002.1 protein AED:0.18 eAED:0.18 QI:0|0.69|0.57|0.92|1|1|14|0|607; (*GB) gi|1762130|gb|AAB39827.1| (e_value=0.0) chaperonin-60 beta subunit [Solanum tuberosum];; (*SWP) sp|P08927|RUBB_

Query	Observed	Mr(expt)	Mr(calc)	ppm	Miss	Score	Expect	Rank	Unique	Peptide
14246	640.8888	1279.7630	1279.7612	1.42	0	34	0.0078	1	U	K.VVAAGANPVLITR.G
16134	771.3909	1540.7672	1540.7620	3.34	0	31	0.047	1	U	R.EVELEDPVENIGAK.L

Proteins matching the same set of peptides:
NbS00034791g0001.1 **Mass:** 64590 **Score:** 46 **Matches:** 2(2) **Sequences:** 2(2)
NbS00034791g0001.1 protein AED:0.18 eAED:0.18 QI:0|0.73|0.68|1|1|1|16|447|608; (*GB) gi|1762130|gb|AAB39827.1| (e_value=0.0) chaperonin-60 beta subunit [Solanum tuberosum];; (*SWP) sp|Q9LJE4|CPNB2_

---

70. NbS00001859g0006.1 **Mass:** 58802 **Score:** 46 **Matches:** 2(1) **Sequences:** 2(1) **emPAI:** 0.11
NbS00001859g0006.1 protein AED:0.10 eAED:0.10 QI:0|0.42|0.5|1|0.85|0.75|8|0|558; (*GB) gi|313585890|gb|ADR71054.1| (e_value=0.0) phosphoglycerate kinase [Nicotiana benthamiana];; (*SWP) sp|Q42961|I

Query	Observed	Mr(expt)	Mr(calc)	ppm	Miss	Score	Expect	Rank	Unique	Peptide
12933	**552.3015**	**1102.5884**	**1102.5870**	**1.24**	**0**	**31**	**0.071**	**1**	**U**	**K.SVGDLTAAELK.G**
19816	**1014.9794**	**2027.9443**	**2027.9396**	**2.33**	**0**	**38**	**0.0071**	**1**	**U**	**R.ADLNVPLDDNQNITDDTR.I**

Proteins matching the same set of peptides:
NbS00006821g0002.1 **Mass:** 53479 **Score:** 46 **Matches:** 2(1) **Sequences:** 2(1)
NbS00006821g0002.1 protein AED:0.20 eAED:0.20 QI:0|0.5|0.57|1|1|1|7|278|508; (*SWP) sp|Q42961|PGKH_TOBAC (e_value=0.0) Phosphoglycerate kinase, chloroplastic OS=Nicotiana tabacum PE=2 SV=1;; (*TAII

---

71. NbS00010454g0009.1 **Mass:** 35657 **Score:** 45 **Matches:** 2(2) **Sequences:** 2(2) **emPAI:** 0.19
NbS00010454g0009.1 protein AED:0.20 eAED:0.20 QI:0|1|0.5|1|1|1|2|0|333; (*SWP) sp|Q40459|PSBO_TOBAC (e_value=0.0) Oxygen-evolving enhancer protein 1, chloroplastic OS=Nicotiana tabacum GN=PSBO PE=2

Query	Observed	Mr(expt)	Mr(calc)	ppm	Miss	Score	Expect	Rank	Unique	Peptide
5578	**425.7200**	**849.4254**	**849.4232**	**2.61**	**0**	**32**	**0.061**	**1**	**U**	**R.GSSFLDPK.G**
18184	**880.9442**	**1759.8738**	**1759.8741**	**-0.19**	**0**	**39**	**0.0071**	**1**	**U**	**K.DGIDYAAVTVQLPGGER.V**

Proteins matching the same set of peptides:
NbS00019818g0001.1 **Mass:** 35404 **Score:** 45 **Matches:** 2(2) **Sequences:** 2(2)
NbS00019818g0001.1 protein AED:0.28 eAED:0.28 QI:0|1|0.5|1|1|1|2|0|332; (*GB) gi|384038815|gb|AFH57998.1| (e_value=0.0) chloroplast PsbO2 precursor [Nicotiana benthamiana];; (*SWP) sp|Q40459|PSBO_T
NbS00027134g0004.1 **Mass:** 39762 **Score:** 45 **Matches:** 2(2) **Sequences:** 2(2)
NbS00027134g0004.1 protein AED:0.02 eAED:0.02 QI:0|1|0.5|1|1|1|2|0|374; (*GB) gi|384038819|gb|AFH58000.1| (e_value=0.0) chloroplast PsbO4 precursor [Nicotiana benthamiana];; (*SWP) sp|Q40459|PSBO_T
NbS00032545g0003.1 **Mass:** 35462 **Score:** 45 **Matches:** 2(2) **Sequences:** 2(2)
NbS00032545g0003.1 protein AED:0.18 eAED:0.18 QI:0|1|0.5|1|1|1|2|0|332; (*GB) gi|384038813|gb|AFH57997.1| (e_value=0.0) chloroplast PsbO1 precursor [Nicotiana benthamiana];; (*SWP) sp|Q40459|PSBO_T
NbC23666742g0001.1 **Mass:** 9076 **Score:** 45 **Matches:** 2(2) **Sequences:** 2(2)
NbC23666742g0001.1 protein ; (*GB) gi|61697115|gb|AAX53163.1| (e_value=8e-52) chloroplast photosynthetic oxygen-evolving protein 33 kDa subunit [Nicotiana benthamiana];; (*SWP) sp|Q40459|PSBO_TOBAC

---

72. NbS00000529g0002.1 **Mass:** 12164 **Score:** 45 **Matches:** 1(1) **Sequences:** 1(1) **emPAI:** 0.28
NbS00000529g0002.1 protein AED:0.44 eAED:0.48 QI:0|0.66|0.5|1|1|1|4|0|104; (*GB) gi|224068340|ref|XP_002302713.1| (e_value=7e-69) predicted protein [Populus trichocarpa];; (*SWP) sp|Q9VI10|SMD2_DRO

Query	Observed	Mr(expt)	Mr(calc)	ppm	Miss	Score	Expect	Rank	Unique	Peptide
13855	**616.3147**	**1230.6148**	**1230.6139**	**0.77**	**0**	**45**	**0.0025**	**1**	**U**	**K.NNTQVLINCR.N**

Proteins matching the same set of peptides:
NbS00008935g0003.1 **Mass:** 12678 **Score:** 45 **Matches:** 1(1) **Sequences:** 1(1)
NbS00008935g0003.1 protein AED:0.00 eAED:0.00 QI:0|-1|0|1|-1|1|1|0|108; (*GB) gi|224068340|ref|XP_002302713.1| (e_value=2e-71) predicted protein [Populus trichocarpa];; (*SWP) sp|Q9VI10|SMD2_DROME
NbS00010757g0002.1 **Mass:** 12636 **Score:** 45 **Matches:** 1(1) **Sequences:** 1(1)
NbS00010757g0002.1 protein AED:0.41 eAED:0.42 QI:185|1|0.6|1|1|1|5|0|108; (*GB) gi|224068340|ref|XP_002302713.1| (e_value=5e-72) predicted protein [Populus trichocarpa];; (*SWP) sp|Q9VI10|SMD2_DRO

---

73. NbS00059497g0003.1 **Mass:** 77510 **Score:** 44 **Matches:** 3(3) **Sequences:** 1(1) **emPAI:** 0.04
NbS00059497g0003.1 protein AED:0.17 eAED:0.17 QI:0|1|1|1|1|1|3|550|697; (*SWP) sp|Q653H7|ARFR_ORYSJ (e_value=0.0) Auxin response factor 18 OS=Oryza sativa subsp. japonica GN=ARF18 PE=2 SV=1;; (*TA

Query	Observed	Mr(expt)	Mr(calc)	ppm	Miss	Score	Expect	Rank	Unique	Peptide
11056	421.7581	841.5016	841.5022	-0.63	0	38	0.009	2	U	R.GLTVSLPR.D 11060 11062

---

74. NbS00003380g0113.1 **Mass:** 45551 **Score:** 44 **Matches:** 1(1) **Sequences:** 1(1) **emPAI:** 0.07
NbS00003380g0113.1 protein AED:0.20 eAED:0.20 QI:170|1|1|1|1|1|8|290|428

Query	Observed	Mr(expt)	Mr(calc)	ppm	Miss	Score	Expect	Rank	Unique	Peptide
14683	**663.3370**	**1324.6595**	**1324.6511**	**6.39**	**0**	**44**	**0.003**	**1**	**U**	**R.VTLADQTTYDAK.V**

---

75. NbS00001538g0002.1 **Mass:** 88204 **Score:** 43 **Matches:** 1(1) **Sequences:** 1(1) **emPAI:** 0.04
NbS00001538g0002.1 protein AED:0.19 eAED:0.19 QI:248|1|1|1|1|1|11|261|788; (*SWP) sp|Q7TP47|HNRPQ_RAT (e_value=2e-31) Heterogeneous nuclear ribonucleoprotein Q OS=Rattus norvegicus GN=Syncrip PE=2

Query	Observed	Mr(expt)	Mr(calc)	ppm	Miss	Score	Expect	Rank	Unique	Peptide

14866	675.3573	1348.7000	1348.6987	1.00	0	43	0.0038	1	U	K.VFSQVGEVTEVR.L

Proteins matching the same set of peptides:
NbS00010360g0002.1 **Mass:** 58440 **Score:** 43 **Matches:** 1(1) **Sequences:** 1(1)
NbS00010360g0002.1 protein AED:0.17 eAED:0.17 QI:711|0.91|0.92|1|0.66|0.53|13|531|510; (*GB) gi|255571057|ref|XP_002526479.1| (e_value=0.0) RNA binding protein, putative [Ricinus communis];; (*SWP)

76. NbS00007843g0002.1 **Mass:** 90945 **Score:** 43 **Matches:** 1(1) **Sequences:** 1(1) **emPAI:** 0.04
NbS00007843g0002.1 protein AED:0.14 eAED:0.14 QI:0|0.76|0.78|1|0.84|0.85|14|2042|813; (*GB) gi|359489218|ref|XP_002270340.2| (e_value=0.0) PREDICTED: uncharacterized protein LOC100232913 [Vitis vi

Query	Observed	Mr(expt)	Mr(calc)	ppm	Miss	Score	Expect	Rank	Unique	Peptide
14866	675.3573	1348.7000	1348.7351	-25.97	1	43	0.0038	1	U	K.VFSKVGEVTEVR.L

77. NbS00061216g0001.1 **Mass:** 20473 **Score:** 43 **Matches:** 1(1) **Sequences:** 1(1) **emPAI:** 0.16
NbS00061216g0001.1 protein AED:0.17 eAED:0.17 QI:0|-1|0|1|-1|1|1|0|179; (*SWP) sp|P11670|PRB1_TOBAC (e_value=3e-109) Basic form of pathogenesis-related protein 1 OS=Nicotiana tabacum PE=3 SV=1;; (

Query	Observed	Mr(expt)	Mr(calc)	ppm	Miss	Score	Expect	Rank	Unique	Peptide
14887	676.8419	1351.6693	1351.6633	4.45	0	43	0.0038	1	U	R.VAAFAQNYANQR.A

78. NbS00008941g0022.1 **Score:** 43 **Matches:** 1(1) **Sequences:** 1(1) **emPAI:** 0.09
NbS00008941g0022.1 protein AED:0.25 eAED:0.25 QI:0|0.5|0.42|1|0.5|0.71|7|0|326; (*GB) gi|350535032|ref|NP_001234428.1| (e_value=0.0) signal recognition particle 54 kDa protein 2 [Solanum lycopersic

Query	Observed	Mr(expt)	Mr(calc)	ppm	Miss	Score	Expect	Rank	Unique	Peptide
12921	551.3300	1100.6454	1100.6190	24.1	0	43	0.0026	2	U	M.VLAELGGSISR.A

Proteins matching the same set of peptides:
NbC25397795g0001.1 **Score:** 43 **Matches:** 1(1) **Sequences:** 1(1)

79. NbS00010323g0010.1 **Mass:** 22088 **Score:** 43 **Matches:** 1(1) **Sequences:** 1(1) **emPAI:** 0.15
NbS00010323g0010.1 protein AED:0.16 eAED:0.16 QI:709|0.5|0.2|1|0.75|0.6|5|0|200; (*GB) gi|298205243|emb|CBI17302.3| (e_value=2e-105) unnamed protein product [Vitis vinifera];; (*SWP) sp|Q54EW1|GLY

Query	Observed	Mr(expt)	Mr(calc)	ppm	Miss	Score	Expect	Rank	Unique	Peptide
16005	759.3896	1516.7646	1516.7634	0.80	0	43	0.0038	1	U	K.NAVFGDSSALAPGGVR.I

Proteins matching the same set of peptides:
NbS00010831g0013.1 **Mass:** 50225 **Score:** 43 **Matches:** 1(1) **Sequences:** 1(1)
NbS00010831g0013.1 protein AED:0.18 eAED:0.18 QI:90|0.75|0.8|1|0.75|0.6|5|271|453; (*GB) gi|211906466|gb|ACJ11726.1| (e_value=0.0) serine hydroxymethyltransferase [Gossypium hirsutum];; (*SWP) sp|
NbS00011304g0014.1 **Mass:** 50225 **Score:** 43 **Matches:** 1(1) **Sequences:** 1(1)
NbS00011304g0014.1 protein AED:0.14 eAED:0.14 QI:87|0.66|0.75|1|1|1|4|310|453; (*GB) gi|211906466|gb|ACJ11726.1| (e_value=0.0) serine hydroxymethyltransferase [Gossypium hirsutum];; (*SWP) sp|Q54Z
NbS00012020g0003.1 **Mass:** 56331 **Score:** 43 **Matches:** 1(1) **Sequences:** 1(1)
NbS00012020g0003.1 protein AED:0.04 eAED:0.04 QI:0|1|0.75|1|0.66|0.5|4|130|507; (*GB) gi|118484713|gb|ABK94226.1| (e_value=0.0) unknown [Populus trichocarpa];; (*SWP) sp|Q54Z26|GLYC1_DICDI (e_value

80. NbS00014997g0006.1 **Mass:** 21403 **Score:** 43 **Matches:** 1(1) **Sequences:** 1(1) **emPAI:** 0.16
NbS00014997g0006.1 protein AED:0.37 eAED:0.37 QI:179|0.8|1|1|0.6|0.66|6|185|195; (*GB) gi|4774163|dbj|BAA77508.1| (e_value=2e-95) F1-ATP synthase delta subunit [Ipomoea batatas];; (*SWP) sp|P22778

Query	Observed	Mr(expt)	Mr(calc)	ppm	Miss	Score	Expect	Rank	Unique	Peptide
13860	616.3445	1230.6744	1230.6721	1.91	0	43	0.0037	1	U	R.NFLVVLAENGR.L

Proteins matching the same set of peptides:
NbS00020265g0006.1 **Mass:** 24140 **Score:** 43 **Matches:** 1(1) **Sequences:** 1(1)
NbS00020265g0006.1 protein AED:0.32 eAED:0.32 QI:176|1|1|1|0.83|0.71|7|243|219; (*GB) gi|4774163|dbj|BAA77508.1| (e_value=4e-104) F1-ATP synthase delta subunit [Ipomoea batatas];; (*SWP) sp|P22778

81. NbS00022525g0113.1 **Mass:** 35306 **Score:** 42 **Matches:** 1(1) **Sequences:** 1(1) **emPAI:** 0.09
NbS00022525g0113.1 protein AED:0.18 eAED:0.18 QI:189|0.87|0.77|1|0.75|0.66|9|554|335

Query	Observed	Mr(expt)	Mr(calc)	ppm	Miss	Score	Expect	Rank	Unique	Peptide
16617	796.9552	1591.8958	1591.8934	1.57	0	42	0.0019	1	U	K.ALEGSDVVIIPAGVPR.K

82. NbS00000088g0014.1 **Mass:** 68182 **Score:** 42 **Matches:** 1(1) **Sequences:** 1(1) **emPAI:** 0.05
NbS00000088g0014.1 protein AED:0.33 eAED:0.33 QI:288|0.94|0.9|1|1|1|20|0|617; (*GB) gi|350537129|ref|NP_001234281.1| (e_value=0.0) vacuolar H+-ATPase A1 subunit isoform [Solanum lycopersicum];; (*S

Query	Observed	Mr(expt)	Mr(calc)	ppm	Miss	Score	Expect	Rank	Unique	Peptide
18366	898.4392	1794.8639	1794.8610	1.58	0	42	0.0034	1	U	R.LAEMPADSGYPAYLAAR.L

Proteins matching the same set of peptides:
NbS00033958g0004.1 **Mass:** 67803 **Score:** 42 **Matches:** 1(1) **Sequences:** 1(1)
NbS00033958g0004.1 protein AED:0.28 eAED:0.28 QI:407|0.89|0.9|1|0.94|1|20|408|613; (*GB) gi|350537129|ref|NP_001234281.1| (e_value=0.0) vacuolar H+-ATPase A1 subunit isoform [Solanum lycopersicum];
NbS00051768g0005.1 **Mass:** 67844 **Score:** 42 **Matches:** 1(1) **Sequences:** 1(1)
NbS00051768g0005.1 protein AED:0.24 eAED:0.24 QI:410|0.89|0.9|1|0.94|0.95|20|403|613; (*GB) gi|350537129|ref|NP_001234281.1| (e_value=0.0) vacuolar H+-ATPase A1 subunit isoform [Solanum lycopersicu

---

83. NbS00002621g0212.1 **Mass:** 26223 **Score:** 42 **Matches:** 2(2) **Sequences:** 2(2) **emPAI:** 0.27
NbS00002621g0212.1 protein AED:0.19 eAED:0.19 QI:122|0.8|1|1|1|1|6|306|255

Query	Observed	Mr(expt)	Mr(calc)	ppm	Miss	Score	Expect	Rank	Unique	Peptide
18854	632.0311	1893.0714	1893.0724	-0.53	0	35	0.0078	1	U	K.LAAAVLGGVDDIWIKPGAK.V
20397	774.0625	2319.1657	2319.1569	3.79	0	31	0.044	1	U	K.LQAEQFKPMEQVTLEPFER.D

Proteins matching the same set of peptides:
NbS00030218g0006.1 **Mass:** 26271 **Score:** 42 **Matches:** 2(2) **Sequences:** 2(2)
NbS00030218g0006.1 protein AED:0.28 eAED:0.28 QI:126|0.83|1|1|0.83|0.71|7|182|255; (*SWP) sp|Q22053|FBRL_CAEEL (e_value=1e-70) rRNA 2'-O-methyltransferase fibrillarin OS=Caenorhabditis elegans GN=

---

84. NbS00013798g0018.1 **Mass:** 7199 **Score:** 42 **Matches:** 1(1) **Sequences:** 1(1) **emPAI:** 0.49
NbS00013798g0018.1 protein AED:0.47 eAED:0.47 QI:0|0|0|1|0|0.5|2|0|68; (*GB) gi|55977763|sp|P00823.2|ATPA_TOBAC (e_value=7e-32) RecName: Full=ATP synthase subunit alpha, chloroplastic; AltName: Fu

Query	Observed	Mr(expt)	Mr(calc)	ppm	Miss	Score	Expect	Rank	Unique	Peptide
15341	708.8962	1415.7778	1415.7772	0.40	0	42	0.0034	1	U	R.IAQIPVSEAYLGR.V

Proteins matching the same set of peptides:
NbS00014241g0002.1 **Mass:** 21890 **Score:** 42 **Matches:** 1(1) **Sequences:** 1(1)
NbS00014241g0002.1 protein AED:0.09 eAED:0.09 QI:0|0|0|1|0|0|2|0|201; (*SWP) sp|P00823|ATPA_TOBAC (e_value=5e-67) ATP synthase subunit alpha, chloroplastic OS=Nicotiana tabacum GN=atpA PE=2 SV=2;;

---

85. NbS00003380g0115.1 **Mass:** 26058 **Score:** 42 **Matches:** 1(1) **Sequences:** 1(1) **emPAI:** 0.13
NbS00003380g0115.1 protein AED:0.12 eAED:0.12 QI:355|0.66|0.85|1|0.33|0.42|7|134|238

Query	Observed	Mr(expt)	Mr(calc)	ppm	Miss	Score	Expect	Rank	Unique	Peptide
12853	546.2900	1090.5654	1090.5658	-0.36	0	42	0.0063	1	U	K.NPALYGELSK.G

Proteins matching the same set of peptides:
NbS00016159g0006.1 **Mass:** 32702 **Score:** 42 **Matches:** 1(1) **Sequences:** 1(1)
NbS00016159g0006.1 protein AED:0.34 eAED:0.36 QI:356|0.57|0.5|1|0.71|0.62|8|0|302; (*GB) gi|115473|sp|P27141.1|CAHC_TOBAC (e_value=3e-179) RecName: Full=Carbonic anhydrase, chloroplastic; AltName:

---

86. NbS00001259g0009.1 **Mass:** 35402 **Score:** 41 **Matches:** 2(2) **Sequences:** 2(2) **emPAI:** 0.20
NbS00001259g0009.1 protein AED:0.04 eAED:0.04 QI:128|0.75|0.77|1|0.75|0.88|9|0|318; (*GB) gi|357505379|ref|XP_003622978.1| (e_value=2e-55) Ribonuclease P protein subunit p25 [Medicago truncatula];;

Query	Observed	Mr(expt)	Mr(calc)	ppm	Miss	Score	Expect	Rank	Unique	Peptide
16061	765.3975	1528.7805	1528.7773	2.10	0	37	0.012	1	U	R.NYISYATSLLQEK.R
17162	822.4011	1642.7877	1642.7852	1.53	0	34	0.024	1	U	R.YNYQPQQQPPPR.H

Proteins matching the same set of peptides:
NbS00012174g0007.1 **Mass:** 27249 **Score:** 41 **Matches:** 2(2) **Sequences:** 2(2)
NbS00012174g0007.1 protein AED:0.23 eAED:0.26 QI:0|0.85|0.75|1|0.71|0.87|8|0|245; (*GB) gi|357505379|ref|XP_003622978.1| (e_value=8e-62) Ribonuclease P protein subunit p25 [Medicago truncatula];;

---

87. NbS00043723g0002.1 **Mass:** 37018 **Score:** 40 **Matches:** 1(1) **Sequences:** 1(1) **emPAI:** 0.09
NbS00043723g0002.1 protein AED:0.01 eAED:0.01 QI:646|0.5|0.66|1|0.5|0.66|3|282|323; (*GB) gi|85068594|gb|ABC69377.1| (e_value=0.0) CYP81B2v2 [Nicotiana tabacum];; (*SWP) sp|O65790|C81F1_ARATH (e_va

Query	Observed	Mr(expt)	Mr(calc)	ppm	Miss	Score	Expect	Rank	Unique	Peptide
17740	844.3767	1686.7389	1686.7373	0.94	0	40	0.0029	1	U	K.YYGENVEDIEEATR.F

---

88. NbS00025246g0002.1 **Score:** 40 **Matches:** 2(2) **Sequences:** 1(1) **emPAI:** 0.07
NbS00025246g0002.1 protein AED:0.04 eAED:0.05 QI:170|1|0.5|1|0|0.5|2|0|447; (*GB) gi|147767800|emb|CAN60219.1| (e_value=1e-95) hypothetical protein VITISV_011848 [Vitis vinifera];; (*TAIR) AT5G578

Query	Observed	Mr(expt)	Mr(calc)	ppm	Miss	Score	Expect	Rank	Unique	Peptide
11506	457.7092	913.4039	913.3851	20.6	0	36	0.0081	2	U	K.MEAEQYK.R 11505

89. NbS00028092g0004.1 **Mass:** 47442 **Score:** 40 **Matches:** 1(1) **Sequences:** 1(1) **emPAI:** 0.07
NbS00028092g0004.1 protein AED:0.36 eAED:0.36 QI:0|0.6|0.33|1|1|1|6|0|450; (*GB) gi|555655|gb|AAA50196.1| (e_value=6e-121) DNA-binding protein [Nicotiana tabacum];; (*TAIR) AT3G18035.1 (e_value=5e-

Query	Observed	Mr(expt)	Mr(calc)	ppm	Miss	Score	Expect	Rank	Unique	Peptide
13408	583.8071	1165.5997	1165.5979	1.56	0	40	0.0068	1	U	K.SYSATAAAPTVK.R

90. NbS00011451g0001.1 **Mass:** 103836 **Score:** 40 **Matches:** 1(1) **Sequences:** 1(1) **emPAI:** 0.03
NbS00011451g0001.1 protein AED:0.10 eAED:0.10 QI:0|0.6|0.33|1|1|1|6|0|925; (*GB) gi|357933581|dbj|BAL15057.1| (e_value=0.0) glutamate receptor 3.4 [Solanum lycopersicum];; (*SWP) sp|Q8GXJ4|GLR34_AR

Query	Observed	Mr(expt)	Mr(calc)	ppm	Miss	Score	Expect	Rank	Unique	Peptide
12085	495.7822	989.5499	989.5142	36.1	0	40	0.0078	1	U	R.ENTLSTLGR.C

91. NbS00000420g0008.1 **Mass:** 45666 **Score:** 40 **Matches:** 1(1) **Sequences:** 1(1) **emPAI:** 0.07
NbS00000420g0008.1 protein AED:0.16 eAED:0.16 QI:0|0.66|0.75|1|1|1|4|509|413; (*GB) gi|15223426|ref|NP_174029.1| (e_value=3e-82) uncharacterized glycine-rich protein [Arabidopsis thaliana];; (*TAIR

Query	Observed	Mr(expt)	Mr(calc)	ppm	Miss	Score	Expect	Rank	Unique	Peptide
16465	782.9055	1563.7964	1563.7926	2.37	0	40	0.0074	1	U	K.SLQQSDLTATMLTR.T

92. NbS00002298g0008.1 **Mass:** 35243 **Score:** 40 **Matches:** 1(1) **Sequences:** 1(1) **emPAI:** 0.09
NbS00002298g0008.1 protein AED:0.21 eAED:0.21 QI:0|0.87|0.55|1|1|1|9|0|332; (*SWP) sp|P19446|MDHG_CITLA (e_value=0.0) Malate dehydrogenase, glyoxysomal OS=Citrullus lanatus PE=1 SV=1;; (*TAIR) AT2

Query	Observed	Mr(expt)	Mr(calc)	ppm	Miss	Score	Expect	Rank	Unique	Peptide
19379	984.4688	1966.9231	1966.9272	-2.10	0	40	0.0053	1	U	R.TGAEEVYQLGPLNEYER.I

Proteins matching the same set of peptides:
NbS00032475g0008.1 **Mass:** 38834 **Score:** 40 **Matches:** 1(1) **Sequences:** 1(1)
NbS00032475g0008.1 protein AED:0.09 eAED:0.10 QI:0|1|0.62|1|1|1|8|0|366; (*GB) gi|350536711|ref|NP_001234005.1| (e_value=0.0) glyoxisomal malate dehydrogenase [Solanum lycopersicum];; (*SWP) sp|P1

93. NbS00007897g0006.1 **Mass:** 26422 **Score:** 39 **Matches:** 2(1) **Sequences:** 2(1) **emPAI:** 0.13
NbS00007897g0006.1 protein AED:0.13 eAED:0.13 QI:259|1|1|1|1|1|5|1352|252; (*GB) gi|51490665|emb|CAG26903.1| (e_value=4e-104) ALY protein [Nicotiana benthamiana];; (*SWP) sp|B5FXN8|THOC4_TAEGU (e_v

Query	Observed	Mr(expt)	Mr(calc)	ppm	Miss	Score	Expect	Rank	Unique	Peptide
11897	483.2563	964.4980	964.4978	0.16	0	39	0.011	1	U	K.GTAEVVFSR.R
15259	469.5747	1405.7024	1405.7024	0.02	0	30	0.064	1		R.YNNVQLDGKPMK.I

Proteins matching the same set of peptides:
NbS00056669g0006.1 **Mass:** 26768 **Score:** 39 **Matches:** 2(1) **Sequences:** 2(1)
NbS00056669g0006.1 protein AED:0.08 eAED:0.08 QI:167|1|1|1|1|1|5|1743|252; (*SWP) sp|B5FXN8|THOC4_TAEGU (e_value=4e-36) THO complex subunit 4 OS=Taeniopygia guttata GN=ALYREF PE=2 SV=1;; (*TAIR) A

94. NbS00025302g0002.1 **Mass:** 12534 **Score:** 39 **Matches:** 1(1) **Sequences:** 1(1) **emPAI:** 0.27
NbS00025302g0002.1 protein

Query	Observed	Mr(expt)	Mr(calc)	ppm	Miss	Score	Expect	Rank	Unique	Peptide
12893	366.5523	1096.6352	1096.6240	10.2	1	39	0.0042	1	U	K.LLPENIKDR.I

95. NbS00005980g0002.1 **Mass:** 62502 **Score:** 39 **Matches:** 1(1) **Sequences:** 1(1) **emPAI:** 0.05
NbS00005980g0002.1 protein AED:0.10 eAED:0.10 QI:0|0.4|0.33|1|1|1|6|0|548; (*GB) gi|350537917|ref|NP_001234829.1| (e_value=0.0) ascorbate oxidase precursor [Solanum lycopersicum];; (*SWP) sp|P2479

Query	Observed	Mr(expt)	Mr(calc)	ppm	Miss	Score	Expect	Rank	Unique	Peptide
13682	603.3222	1204.6299	1204.6274	2.04	0	39	0.011	1	U	K.NYNLVNPIMK.N

Proteins matching the same set of peptides:
NbS00052235g0008.1 **Mass:** 61657 **Score:** 39 **Matches:** 1(1) **Sequences:** 1(1)
NbS00052235g0008.1 protein AED:0.09 eAED:0.09 QI:76|0.66|0.5|1|1|1|4|0|542; (*GB) gi|350537917|ref|NP_001234829.1| (e_value=0.0) ascorbate oxidase precursor [Solanum lycopersicum];; (*SWP) sp|P247

96. NbS00033391g0006.1 **Mass:** 59777 **Score:** 39 **Matches:** 1(1) **Sequences:** 1(1) **emPAI:** 0.05

NbS00033391g0006.1 protein AED:0.14 eAED:0.14 QI:195|0.85|0.87|1|0.85|1|8|383|567; (*GB) gi|359479362|ref|XP_002277357.2| (e_value=0.0) PREDICTED: ruBisCO large subunit-binding protein subunit alpl

Query	Observed	Mr(expt)	Mr(calc)	ppm	Miss	Score	Expect	Rank	Unique	Peptide
17113	818.8929	1635.7712	1635.7700	0.73	0	39	0.0081	1	U	K.TNDSAGDGTTTASVLAR.E

Proteins matching the same set of peptides:

NbS00040945g0003.1 **Mass:** 62276 **Score:** 39 **Matches:** 1(1) **Sequences:** 1(1)
NbS00040945g0003.1 protein AED:0.10 eAED:0.10 QI:277|1|0.87|1|1|1|8|307|589; (*GB) gi|359479362|ref|XP_002277357.2| (e_value=0.0) PREDICTED: ruBisCO large subunit-binding protein subunit alpha, chl

---

97. NbS00021832g0023.1 **Mass:** 9182 **Score:** 39 **Matches:** 1(1) **Sequences:** 1(1) **emPAI:** 0.38
NbS00021832g0023.1 protein AED:0.35 eAED:0.35 QI:0|0|0|1|0|0|2|0|80; (*GB) gi|305671961|gb|ADM63350.1| (e_value=2e-44) photosystem II cp47 protein [Aralidium pinnatifidum];; (*SWP) sp|A4QLM0|PSBB_I

Query	Observed	Mr(expt)	Mr(calc)	ppm	Miss	Score	Expect	Rank	Unique	Peptide
14382	645.8386	1289.6627	1289.6615	0.89	0	39	0.011	1	U	R.AQLGEIFELDR.A

---

98. NbS00033277g0006.1 **Score:** 38 **Matches:** 1(1) **Sequences:** 1(1) **emPAI:** 0.26
NbS00033277g0006.1 protein AED:0.56 eAED:0.56 QI:0|0|0|0.16|1|1|6|0|118

Query	Observed	Mr(expt)	Mr(calc)	ppm	Miss	Score	Expect	Rank	Unique	Peptide
10994	416.2498	830.4851	830.4861	-1.21	0	38	0.011	2	U	K.SETLILR.Q

---

99. NbS00025171g0003.1 **Mass:** 24079 **Score:** 38 **Matches:** 1(1) **Sequences:** 1(1) **emPAI:** 0.14
NbS00025171g0003.1 protein AED:0.00 eAED:0.00 QI:54|1|1|1|1|1|3|6|221; (*GB) gi|3893824|gb|AAD03394.1| (e_value=4e-114) ATPase beta subunit [Nicotiana sylvestris];; (*SWP) sp|P17614|ATPBM_NICPL (e_

Query	Observed	Mr(expt)	Mr(calc)	ppm	Miss	Score	Expect	Rank	Unique	Peptide
14783	671.3719	1340.7293	1340.7299	-0.45	0	38	0.008	1	U	R.IINVIGEAIDER.G

---

100. NbS00005969g0002.1 **Mass:** 63583 **Score:** 38 **Matches:** 2(1) **Sequences:** 2(1) **emPAI:** 0.05
NbS00005969g0002.1 protein AED:0.07 eAED:0.07 QI:0|0.5|0.55|0.88|1|1|9|177|561; (*GB) gi|225456270|ref|XP_002283518.1| (e_value=0.0) PREDICTED: nucleolar protein 56-like [Vitis vinifera];; (*SWP) s

Query	Observed	Mr(expt)	Mr(calc)	ppm	Miss	Score	Expect	Rank	Unique	Peptide
11865	481.2715	960.5284	960.5280	0.42	0	38	0.015	1	U	K.FSLGLAEPK.L
15233	702.3642	1402.7138	1402.7126	0.88	0	30	0.071	1	U	K.CPSSTLQILGAEK.A

Proteins matching the same set of peptides:

NbS00023900g0001.1 **Mass:** 64240 **Score:** 38 **Matches:** 2(1) **Sequences:** 2(1)
NbS00023900g0001.1 protein AED:0.11 eAED:0.11 QI:0|0.85|0.75|1|1|1|8|178|567; (*SWP) sp|O94514|NOP56_SCHPO (e_value=0.0) Nucleolar protein 56 OS=Schizosaccharomyces pombe (strain 972 / ATCC 24843)

---

101. NbS00000247g0008.1 **Mass:** 74604 **Score:** 38 **Matches:** 1(1) **Sequences:** 1(1) **emPAI:** 0.04
NbS00000247g0008.1 protein AED:0.26 eAED:0.27 QI:16|0.85|0.73|1|1|1|15|0|662; (*GB) gi|416664|sp|Q03194.1|PMA4_NICPL (e_value=0.0) RecName: Full=Plasma membrane ATPase 4; AltName: Full=Proton pump

Query	Observed	Mr(expt)	Mr(calc)	ppm	Miss	Score	Expect	Rank	Unique	Peptide
12714	536.7809	1071.5473	1071.5461	1.14	0	38	0.013	1	U	R.EAQWALAQR.T

Proteins matching the same set of peptides:

NbS00001908g0012.1 **Mass:** 73584 **Score:** 38 **Matches:** 1(1) **Sequences:** 1(1)
NbS00001908g0012.1 protein AED:0.22 eAED:0.22 QI:329|0.91|0.92|1|1|1|13|0|657; (*GB) gi|416664|sp|Q03194.1|PMA4_NICPL (e_value=0.0) RecName: Full=Plasma membrane ATPase 4; AltName: Full=Proton pump
NbS00028315g0030.1 **Mass:** 60565 **Score:** 38 **Matches:** 1(1) **Sequences:** 1(1)
NbS00028315g0030.1 protein AED:0.21 eAED:0.23 QI:30|0.55|0.5|1|0.66|0.7|10|104|545; (*GB) gi|64460298|gb|AAR32129.2| (e_value=0.0) proton P-ATPase [Nicotiana tabacum];; (*SWP) sp|Q03194|PMA4_NICPL
NbS00028637g0005.1 **Mass:** 77087 **Score:** 38 **Matches:** 1(1) **Sequences:** 1(1)
NbS00028637g0005.1 protein AED:0.23 eAED:0.23 QI:0|0.72|0.58|1|0.81|0.66|12|0|693; (*GB) gi|5669151|gb|AAD46186.1| (e_value=0.0) plasma membrane proton ATPase [Nicotiana plumbaginifolia];; (*SWP) s
NbS00047432g0001.1 **Mass:** 66424 **Score:** 38 **Matches:** 1(1) **Sequences:** 1(1)
NbS00047432g0001.1 protein AED:0.20 eAED:0.20 QI:0|0.55|0.7|1|1|1|10|104|600; (*GB) gi|255572779|ref|XP_002527322.1| (e_value=0.0) H(\+)-transporting atpase plant/fungi plasma membrane type, putat:

---

102. NbS00005125g0015.1 **Mass:** 38864 **Score:** 37 **Matches:** 1(1) **Sequences:** 1(1) **emPAI:** 0.09
NbS00005125g0015.1 protein AED:0.27 eAED:0.27 QI:600|0.91|0.84|1|0.83|0.69|13|286|356; (*GB) gi|304368145|gb|ADM26718.1| (e_value=0.0) glycolate oxidase [Nicotiana benthamiana];; (*SWP) sp|P05414|(

Query	Observed	Mr(expt)	Mr(calc)	ppm	Miss	Score	Expect	Rank	Unique	Peptide
12715	536.8111	1071.6076	1071.6077	-0.05	0	37	0.013	1	U	R.IPVFLDGGVR.R

Proteins matching the same set of peptides:
NbS00025736g0004.1 **Mass:** 38849 **Score:** 37 **Matches:** 1(1) **Sequences:** 1(1)
NbS00025736g0004.1 protein AED:0.28 eAED:0.28 QI:605|0.91|0.84|1|0.83|0.76|13|359|356; (*GB) gi|304368145|gb|ADM26718.1| (e_value=0.0) glycolate oxidase [Nicotiana benthamiana];; (*SWP) sp|P05414|(

---

103. NbS00042363g0004.1 **Mass:** 39921 **Score:** 37 **Matches:** 1(1) **Sequences:** 1(1) **emPAI:** 0.08
NbS00042363g0004.1 protein AED:0.29 eAED:0.29 QI:95|1|1|1|0.25|0.2|5|264|361; (*SWP) sp|Q8LB81|GDL79_ARATH (e_value=0.0) GDSL esterase/lipase At5g33370 OS=Arabidopsis thaliana GN=At5g33370 PE=2 SV=

Query	Observed	Mr(expt)	Mr(calc)	ppm	Miss	Score	Expect	Rank	Unique	Peptide
18146	876.9401	1751.8657	1751.8591	3.78	0	37	0.012	1	U	R.ISQQLQNFEQYQAR.V

---

104. NbS00003662g0021.1 **Mass:** 29426 **Score:** 37 **Matches:** 1(1) **Sequences:** 1(1) **emPAI:** 0.11
NbS00003662g0021.1 protein AED:0.24 eAED:0.34 QI:0|0|0|1|0|0|3|0|257; (*GB) gi|78102516|ref|YP_358657.1| (e_value=6e-112) ATP synthase CF1 alpha subunit [Nicotiana sylvestris];; (*SWP) sp|Q3C1H4|A[

Query	Observed	Mr(expt)	Mr(calc)	ppm	Miss	Score	Expect	Rank	Unique	Peptide
15842	744.9052	1487.7959	1487.7944	1.01	0	37	0.012	1	U	K.ASSVAQVVTTLQER.G

---

105. NbS00006781g0007.1 **Mass:** 25029 **Score:** 37 **Matches:** 1(1) **Sequences:** 1(1) **emPAI:** 0.13
NbS00006781g0007.1 protein AED:0.08 eAED:0.08 QI:0|1|0.33|1|1|1|3|0|248; (*GB) gi|292653543|gb|ADE34289.1| (e_value=3e-125) aquaporin TIP2;3 [Gossypium hirsutum];; (*SWP) sp|Q41951|TIP21_ARATH (e_

Query	Observed	Mr(expt)	Mr(calc)	ppm	Miss	Score	Expect	Rank	Unique	Peptide
10484	374.2112	746.4079	746.4075	0.56	0	37	0.025	1	U	M.PSIAFGR.F

---

106. NbS00004222g0004.1 **Mass:** 16815 **Score:** 37 **Matches:** 1(1) **Sequences:** 1(1) **emPAI:** 0.20
NbS00004222g0004.1 protein AED:0.16 eAED:0.16 QI:44|1|1|1|1|1|2|322|146; (*GB) gi|311893225|dbj|BAJ25784.1| (e_value=2e-97) putative PR-10 type pathogenesis-related protein [Nicotiana tabacum];; (

Query	Observed	Mr(expt)	Mr(calc)	ppm	Miss	Score	Expect	Rank	Unique	Peptide
7471	444.7500	887.4854	887.4865	-1.20	0	37	0.02	1	U	K.VGSVVNWK.Y

Proteins matching the same set of peptides:
NbS00013017g0003.1 **Mass:** 16718 **Score:** 37 **Matches:** 1(1) **Sequences:** 1(1)
NbS00013017g0003.1 protein AED:0.23 eAED:0.23 QI:44|1|1|1|1|1|2|330|146; (*GB) gi|311893225|dbj|BAJ25784.1| (e_value=7e-95) putative PR-10 type pathogenesis-related protein [Nicotiana tabacum];; (

---

107. NbS00000058g0018.1 **Mass:** 24519 **Score:** 37 **Matches:** 1(1) **Sequences:** 1(1) **emPAI:** 0.14
NbS00000058g0018.1 protein AED:0.02 eAED:0.02 QI:0|1|0|1|1|1|2|0|221; (*GB) gi|30013659|gb|AAP03872.1| (e_value=2e-130) putative photosystem I subunit III precursor [Nicotiana tabacum];; (*SWP) sp

Query	Observed	Mr(expt)	Mr(calc)	ppm	Miss	Score	Expect	Rank	Unique	Peptide
13807	612.8645	1223.7145	1223.7125	1.59	0	37	0.0052	1	U	K.EIIIDVPLANK.L

Proteins matching the same set of peptides:
NbS00009051g0007.1 **Mass:** 27636 **Score:** 37 **Matches:** 1(1) **Sequences:** 1(1)
NbS00009051g0007.1 protein AED:0.31 eAED:0.31 QI:0|0.5|0|1|1|1|3|0|249; (*GB) gi|30013659|gb|AAP03872.1| (e_value=1e-120) putative photosystem I subunit III precursor [Nicotiana tabacum];; (*SWP) s
NbS00035792g0016.1 **Mass:** 24757 **Score:** 37 **Matches:** 1(1) **Sequences:** 1(1)
NbS00035792g0016.1 protein AED:0.06 eAED:0.06 QI:0|0|0|1|1|1|2|0|224; (*GB) gi|30013659|gb|AAP03872.1| (e_value=3e-103) putative photosystem I subunit III precursor [Nicotiana tabacum];; (*SWP) sp
NbS00061093g0007.1 **Mass:** 22605 **Score:** 37 **Matches:** 1(1) **Sequences:** 1(1)
NbS00061093g0007.1 protein AED:0.06 eAED:0.06 QI:0|0|0|1|0|0.5|2|0|206; (*GB) gi|30013659|gb|AAP03872.1| (e_value=3e-90) putative photosystem I subunit III precursor [Nicotiana tabacum];; (*SWP) sp

---

108. NbS00009732g0022.1 **Mass:** 44722 **Score:** 37 **Matches:** 1(1) **Sequences:** 1(1) **emPAI:** 0.07
NbS00009732g0022.1 protein AED:0.27 eAED:0.28 QI:90|0.9|0.90|1|0.9|0.81|11|248|411; (*GB) gi|50400860|sp|Q43497.1|MDAR_SOLLC (e_value=0.0) RecName: Full=Monodehydroascorbate reductase; Short=MDAR;

Query	Observed	Mr(expt)	Mr(calc)	ppm	Miss	Score	Expect	Rank	Unique	Peptide
18762	939.9977	1877.9808	1877.9775	1.76	0	37	0.011	1	U	K.TSVPDVYAVGDVATFPLK.M

Proteins matching the same set of peptides:
NbS00032372g0009.1 **Mass:** 49364 **Score:** 37 **Matches:** 1(1) **Sequences:** 1(1)
NbS00032372g0009.1 protein AED:0.22 eAED:0.22 QI:92|0.9|0.72|1|0.8|0.81|11|329|452; (*GB) gi|50400860|sp|Q43497.1|MDAR_SOLLC (e_value=0.0) RecName: Full=Monodehydroascorbate reductase; Short=MDAR;

---

109. NbS00028158g0015.1 **Mass:** 18033 **Score:** 37 **Matches:** 1(1) **Sequences:** 1(1) **emPAI:** 0.19
NbS00028158g0015.1 protein AED:0.44 eAED:0.54 QI:0|0.75|0.4|1|1|1|5|0|169; (*GB) gi|388500696|gb|AFK38414.1| (e_value=1e-63) unknown [Lotus japonicus];; (*SWP) sp|O04616|Y4115_ARATH (e_value=4e-59)

Query	Observed	Mr(expt)	Mr(calc)	ppm	Miss	Score	Expect	Rank	Unique	Peptide
13554	594.3116	1186.6087	1186.6081	0.55	0	37	0.018	1	U	K.ELAEDIEQLK.K

110. NbS00013071g0001.1 **Mass:** 39307 **Score:** 37 **Matches:** 2(1) **Sequences:** 2(1) **emPAI:** 0.08
NbS00013071g0001.1 protein AED:0.14 eAED:0.14 QI:0|0.66|0.25|1|1|1|4|0|360; (*SWP) sp|Q9XIV8|PERN1_TOBAC (e_value=0.0) Peroxidase N1 OS=Nicotiana tabacum GN=poxN1 PE=1 SV=1;; (*TAIR) AT5G64120.1 (e

Query	Observed	Mr(expt)	Mr(calc)	ppm	Miss	Score	Expect	Rank	Unique	Peptide
12096	497.2476	992.4807	992.4815	-0.79	0	32	0.061	1	U	K.GFDVIEDAK.T
13452	587.2715	1172.5285	1172.5285	0.07	0	37	0.0093	1	U	R.VGFYSSTCPR.A

Proteins matching the same set of peptides:
NbS00037769g0004.1 **Mass:** 36161 **Score:** 37 **Matches:** 2(1) **Sequences:** 2(1)
NbS00037769g0004.1 protein AED:0.16 eAED:0.16 QI:0|1|0.33|1|1|1|3|0|330; (*GB) gi|75315324|sp|Q9XIV8.1|PERN1_TOBAC (e_value=0.0) RecName: Full=Peroxidase N1; AltName: Full=Peroxidase B2; AltName: I

111. NbS00000172g0008.1 **Mass:** 28107 **Score:** 37 **Matches:** 1(1) **Sequences:** 1(1) **emPAI:** 0.12
NbS00000172g0008.1 protein AED:0.09 eAED:0.09 QI:94|0.66|0.75|1|0.33|0|4|260|260; (*SWP) sp|P0C582|M2OM_NEUCR (e_value=7e-53) Putative mitochondrial 2-oxoglutarate/malate carrier protein OS=Neurosp

Query	Observed	Mr(expt)	Mr(calc)	ppm	Miss	Score	Expect	Rank	Unique	Peptide
17655	557.6428	1669.9065	1669.9039	1.55	0	37	0.01	1	U	K.AIEANEGKPLPLYQK.A

Proteins matching the same set of peptides:
NbS00003287g0001.1 **Mass:** 29238 **Score:** 37 **Matches:** 1(1) **Sequences:** 1(1)
NbS00003287g0001.1 protein AED:0.38 eAED:0.38 QI:0|0.75|0.4|1|1|1|5|0|271; (*GB) gi|19913105|emb|CAC84545.1| (e_value=0.0) dicarboxylate/tricarboxylate carrier [Nicotiana tabacum];; (*SWP) sp|P0C58
NbS00007342g0007.1 **Mass:** 36558 **Score:** 37 **Matches:** 1(1) **Sequences:** 1(1)
NbS00007342g0007.1 protein AED:0.17 eAED:0.24 QI:0|0|0|0.75|0.33|0.25|4|0|329; (*GB) gi|19913105|emb|CAC84545.1| (e_value=3e-112) dicarboxylate/tricarboxylate carrier [Nicotiana tabacum];; (*SWP) s
NbS00014260g0003.1 **Mass:** 16528 **Score:** 37 **Matches:** 1(1) **Sequences:** 1(1)
NbS00014260g0003.1 protein AED:0.32 eAED:0.33 QI:0|-1|0|1|-1|1|1|0|152; (*GB) gi|19913107|emb|CAC84546.1| (e_value=7e-82) dicarboxylate/tricarboxylate carrier [Nicotiana tabacum];; (*SWP) sp|Q54PY
NbS00056566g0003.1 **Mass:** 32051 **Score:** 37 **Matches:** 1(1) **Sequences:** 1(1)
NbS00056566g0003.1 protein AED:0.31 eAED:0.31 QI:246|1|1|1|1|1|6|596|297; (*GB) gi|19913105|emb|CAC84545.1| (e_value=0.0) dicarboxylate/tricarboxylate carrier [Nicotiana tabacum];; (*SWP) sp|P0C582

112. NbS00005930g0008.1 **Mass:** 32501 **Score:** 36 **Matches:** 1(1) **Sequences:** 1(1) **emPAI:** 0.10
NbS00005930g0008.1 protein AED:0.34 eAED:0.35 QI:0|0.5|0|1|0.5|0.66|3|0|299; (*GB) gi|359495241|ref|XP_003634942.1| (e_value=7e-112) PREDICTED: uncharacterized protein LOC100248314 isoform 2 [Vitis

Query	Observed	Mr(expt)	Mr(calc)	ppm	Miss	Score	Expect	Rank	Unique	Peptide
17271	828.8874	1655.7602	1655.7614	-0.69	0	36	0.011	1	U	R.MVIDEGQEAFGFTGR.N

Proteins matching the same set of peptides:
NbS00007597g0013.1 **Mass:** 31610 **Score:** 36 **Matches:** 1(1) **Sequences:** 1(1)
NbS00007597g0013.1 protein AED:0.37 eAED:0.37 QI:0|1|0.33|1|1|1|3|0|291; (*GB) gi|359495241|ref|XP_003634942.1| (e_value=2e-111) PREDICTED: uncharacterized protein LOC100248314 isoform 2 [Vitis vi

113. NbS00006448g0011.1 **Mass:** 20920 **Score:** 36 **Matches:** 1(1) **Sequences:** 1(1) **emPAI:** 0.16
NbS00006448g0011.1 protein AED:0.25 eAED:0.25 QI:194|1|1|1|1|1|5|350|181; (*GB) gi|10799832|emb|CAC12883.1| (e_value=3e-116) ribosomal protein L11-like [Nicotiana tabacum];; (*SWP) sp|P42794|RL112_

Query	Observed	Mr(expt)	Mr(calc)	ppm	Miss	Score	Expect	Rank	Unique	Peptide
16011	759.9144	1517.8142	1517.8090	3.44	0	36	0.014	1	U	K.VLEQLSGQSPVFSK.A

Proteins matching the same set of peptides:
NbS00007322g0010.1 **Mass:** 20946 **Score:** 36 **Matches:** 1(1) **Sequences:** 1(1)
NbS00007322g0010.1 protein AED:0.24 eAED:0.24 QI:159|1|1|1|0.25|0.4|5|341|181; (*GB) gi|10799832|emb|CAC12883.1| (e_value=8e-121) ribosomal protein L11-like [Nicotiana tabacum];; (*SWP) sp|P42795|I
NbS00009164g0009.1 **Mass:** 18458 **Score:** 36 **Matches:** 1(1) **Sequences:** 1(1)
NbS00009164g0009.1 protein AED:0.14 eAED:0.14 QI:134|0.8|0.83|1|0.6|0.5|6|370|159; (*GB) gi|10799832|emb|CAC12883.1| (e_value=7e-100) ribosomal protein L11-like [Nicotiana tabacum];; (*SWP) sp|P42
NbS00009449g0002.1 **Mass:** 20916 **Score:** 36 **Matches:** 1(1) **Sequences:** 1(1)
NbS00009449g0002.1 protein AED:0.34 eAED:0.34 QI:103|1|0.8|1|1|1|5|0|181; (*GB) gi|10799832|emb|CAC12883.1| (e_value=7e-120) ribosomal protein L11-like [Nicotiana tabacum];; (*SWP) sp|P42795|RL111_
NbS00018998g0022.1 **Mass:** 23955 **Score:** 36 **Matches:** 1(1) **Sequences:** 1(1)
NbS00018998g0022.1 protein AED:0.40 eAED:0.40 QI:0|0.75|0.4|0.8|0.5|0.6|5|0|209; (*GB) gi|223944105|gb|ACN26136.1| (e_value=5e-74) unknown [Zea mays];; (*SWP) sp|Q0DK10|RL11_ORYSJ (e_value=2e-74) (
NbS00024632g0002.1 **Mass:** 19816 **Score:** 36 **Matches:** 1(1) **Sequences:** 1(1)
NbS00024632g0002.1 protein AED:0.07 eAED:0.07 QI:0|1|0.5|1|1|1|4|0|171; (*SWP) sp|P42795|RL111_ARATH (e_value=5e-110) 60S ribosomal protein L11-1 OS=Arabidopsis thaliana GN=RPL11A PE=1 SV=2;; (*TA
NbS00028977g0006.1 **Mass:** 20946 **Score:** 36 **Matches:** 1(1) **Sequences:** 1(1)
NbS00028977g0006.1 protein AED:0.25 eAED:0.25 QI:134|1|1|1|1|1|5|428|181; (*GB) gi|10799832|emb|CAC12883.1| (e_value=8e-121) ribosomal protein L11-like [Nicotiana tabacum];; (*SWP) sp|P42795|RL111_
NbS00040668g0004.1 **Mass:** 19692 **Score:** 36 **Matches:** 1(1) **Sequences:** 1(1)

NbS00040668g0004.1 protein AED:0.33 eAED:0.33 QI:28|1|0.75|1|1|1|4|0|170; (*GB) gi|10799832|emb|CAC12883.1| (e_value=7e-110) ribosomal protein L11-like [Nicotiana tabacum];; (*SWP) sp|P42794|RL112_

---

114. NbS00015227g0002.1 **Mass:** 18882 **Score:** 36 **Matches:** 1(1) **Sequences:** 1(1) **emPAI:** 0.18
NbS00015227g0002.1 protein AED:0.14 eAED:0.16 QI:0|-1|0|1|-1|1|1|0|171; (*GB) gi|237783971|gb|ACR19782.1| (e_value=5e-91) ATP synthase CF1 beta subunit [Nolana galapagensis];; (*SWP) sp|P26530|ATPI

Query	Observed	Mr(expt)	Mr(calc)	ppm	Miss	Score	Expect	Rank	Unique	Peptide
19885	**1031.0223**	**2060.0301**	**2060.0248**	**2.57**	**0**	**36**	**0.012**	**1**	**U**	**K.GIYPAVDPLDSTSTMLQPR.I**

---

115. NbS00009537g0010.1 **Mass:** 79898 **Score:** 36 **Matches:** 1(1) **Sequences:** 1(1) **emPAI:** 0.04
NbS00009537g0010.1 protein AED:0.14 eAED:0.14 QI:0|0.6|0.33|1|0.6|0.5|6|257|695; (*GB) gi|297738216|emb|CBI27417.3| (e_value=4e-139) unnamed protein product [Vitis vinifera];; (*SWP) sp|A8MQR0|WIT2

Query	Observed	Mr(expt)	Mr(calc)	ppm	Miss	Score	Expect	Rank	Unique	Peptide
14371	**645.3016**	**1288.5887**	**1288.5895**	**-0.59**	**0**	**36**	**0.012**	**1**	**U**	**K.ANELNEVSEER.L**

Proteins matching the same set of peptides:
NbS00012103g0005.1 **Mass:** 80705 **Score:** 36 **Matches:** 1(1) **Sequences:** 1(1)
NbS00012103g0005.1 protein AED:0.10 eAED:0.10 QI:0|1|1|1|1|0.75|4|287|699; (*GB) gi|297738216|emb|CBI27417.3| (e_value=5e-175) unnamed protein product [Vitis vinifera];; (*SWP) sp|A8MQR0|WIT2_ARATH

---

116. NbS00012621g0112.1 **Mass:** 34710 **Score:** 36 **Matches:** 1(1) **Sequences:** 1(1) **emPAI:** 0.10
NbS00012621g0112.1 protein AED:0.39 eAED:0.39 QI:0|0.54|0.58|0.91|0.54|0.66|12|726|314

Query	Observed	Mr(expt)	Mr(calc)	ppm	Miss	Score	Expect	Rank	Unique	Peptide
14483	651.8616	1301.7087	1301.6616	36.2	0	36	0.017	2	U	R.ADDGSLIQGLWK.V

---

117. NbS00004792g0016.1 **Mass:** 104270 **Score:** 36 **Matches:** 2(1) **Sequences:** 2(1) **emPAI:** 0.06
NbS00004792g0016.1 protein AED:0.26 eAED:0.27 QI:7|0.55|0.4|1|0.77|0.7|10|0|938; (*GB) gi|399213|sp|P31542.1|CLPAB_SOLLC (e_value=0.0) RecName: Full=ATP-dependent Clp protease ATP-binding subunit c

Query	Observed	Mr(expt)	Mr(calc)	ppm	Miss	Score	Expect	Rank	Unique	Peptide
12377	**515.2892**	**1028.5639**	**1028.5614**	**2.44**	**0**	**32**	**0.047**	**1**	**U**	**K.TAIAEGLAQR.I**
15761	**738.8992**	**1475.7839**	**1475.7773**	**4.49**	**0**	**31**	**0.051**	**1**	**U**	**R.GSGFVAVEIPFTPR.A**

Proteins matching the same set of peptides:
NbS00017400g0004.1 **Mass:** 102872 **Score:** 36 **Matches:** 2(1) **Sequences:** 2(1)
NbS00017400g0004.1 protein AED:0.33 eAED:0.33 QI:0|0.7|0.54|0.90|1|1|11|0|928; (*SWP) sp|P31542|CLPAB_SOLLC (e_value=0.0) ATP-dependent Clp protease ATP-binding subunit clpA homolog CD4B, chloropla
NbS00020863g0003.1 **Mass:** 59028 **Score:** 36 **Matches:** 2(1) **Sequences:** 2(1)
NbS00020863g0003.1 protein AED:0.25 eAED:0.25 QI:0|0.62|0.33|1|1|1|9|0|535; (*GB) gi|399213|sp|P31542.1|CLPAB_SOLLC (e_value=0.0) RecName: Full=ATP-dependent Clp protease ATP-binding subunit clpA h
NbS00024706g0002.1 **Mass:** 100869 **Score:** 36 **Matches:** 2(1) **Sequences:** 2(1)
NbS00024706g0002.1 protein AED:0.27 eAED:0.27 QI:0|0.87|0.66|1|1|1|9|0|908; (*GB) gi|399213|sp|P31542.1|CLPAB_SOLLC (e_value=0.0) RecName: Full=ATP-dependent Clp protease ATP-binding subunit clpA h

---

118. NbS00007742g0006.1 **Mass:** 33872 **Score:** 35 **Matches:** 2(1) **Sequences:** 2(1) **emPAI:** 0.10
NbS00007742g0006.1 protein AED:0.40 eAED:0.42 QI:0|0.5|0.42|1|1|1|7|0|319; (*GB) gi|94466659|emb|CAJ44458.1| (e_value=2e-110) ALY protein [Nicotiana benthamiana];; (*SWP) sp|Q9JJW6|REFP2_MOUSE (e_v

Query	Observed	Mr(expt)	Mr(calc)	ppm	Miss	Score	Expect	Rank	Unique	Peptide
13495	**589.3066**	**1176.5986**	**1176.5986**	**-0.01**	**0**	**35**	**0.026**	**1**	**U**	**R.AAGISSGLESGTK.V**
15259	469.5747	1405.7024	1405.7024	0.02	0	30	0.064	1		R.YNNVQLDGKPMK.I

---

119. NbS00007661g0019.1 **Mass:** 105444 **Score:** 35 **Matches:** 2(1) **Sequences:** 2(1) **emPAI:** 0.03
NbS00007661g0019.1 protein AED:0.18 eAED:0.18 QI:34|0.90|0.91|1|1|0.91|12|402|1023; (*GB) gi|22329468|ref|NP_172510.2| (e_value=0.0) Nucleoporin autopeptidase [Arabidopsis thaliana];; (*SWP) sp|P52

Query	Observed	Mr(expt)	Mr(calc)	ppm	Miss	Score	Expect	Rank	Unique	Peptide
11259	**438.7608**	**875.5071**	**875.5076**	**-0.57**	**0**	**30**	**0.07**	**1**	**U**	**R.ISSLLTSR.H**
17774	**848.3917**	**1694.7688**	**1694.7676**	**0.71**	**0**	**35**	**0.014**	**1**	**U**	**K.VPFFSDDEETPSTPK.A**

---

120. NbS00017034g0014.1 **Mass:** 18705 **Score:** 35 **Matches:** 1(1) **Sequences:** 1(1) **emPAI:** 0.18
NbS00017034g0014.1 protein AED:0.20 eAED:0.20 QI:0|0.8|0.83|0.8|0.83|6|26|160; (*GB) gi|225454123|ref|XP_002269748.1| (e_value=9e-100) PREDICTED: cleavage and polyadenylation specificity facto

Query	Observed	Mr(expt)	Mr(calc)	ppm	Miss	Score	Expect	Rank	Unique	Peptide
16444	**779.9334**	**1557.8523**	**1557.8515**	**0.50**	**0**	**35**	**0.015**	**1**	**U**	**R.YGPVISTIPQQLSR.F**

Proteins matching the same set of peptides:

NbS00018945g0009.1 **Mass:** 16278 **Score:** 35 **Matches:** 1(1) **Sequences:** 1(1)
NbS00018945g0009.1 protein AED:0.30 eAED:0.30 QI:161|0.5|0.8|1|0.5|0.4|5|27|139; (*GB) gi|315259982|gb|ADT92189.1| (e_value=7e-72) cleavage and polyadenylation specificity factor 5 [Zea mays];; (*S

---

121. NbS00003269g0011.1 **Mass:** 67651 **Score:** 35 **Matches:** 1(1) **Sequences:** 1(1) **emPAI:** 0.05
NbS00003269g0011.1 protein AED:0.29 eAED:0.29 QI:0|0.86|0.83|1|0.91|0.87|24|420|599; (*SWP) sp|Q42656|AGAL_COFAR (e_value=9e-149) Alpha-galactosidase OS=Coffea arabica PE=1 SV=1;; (*TAIR) AT5G0838(

Query	Observed	Mr(expt)	Mr(calc)	ppm	Miss	Score	Expect	Rank	Unique	Peptide
16694	**799.9225**	**1597.8305**	**1597.8311**	**-0.38**	**0**	**35**	**0.018**	**1**	**U**	**K.EVIAVNQDEIGVQGK.K**

Proteins matching the same set of peptides:
NbS00006909g0017.1 **Mass:** 46304 **Score:** 35 **Matches:** 1(1) **Sequences:** 1(1)
NbS00006909g0017.1 protein AED:0.20 eAED:0.20 QI:179|0.92|0.86|1|0.85|0.86|15|403|409; (*GB) gi|255544191|ref|XP_002513158.1| (e_value=0.0) alpha-galactosidase/alpha-n-acetylgalactosaminidase, puta

---

122. NbS00001849g0017.1 **Mass:** 8189 **Score:** 35 **Matches:** 2(1) **Sequences:** 2(1) **emPAI:** 0.43
NbS00001849g0017.1 protein AED:0.76 eAED:1.00 QI:0|0|0|0.66|0.5|0.66|3|0|72; (*GB) gi|94466657|emb|CAJ44457.1| (e_value=3e-26) ALY protein [Nicotiana benthamiana];; (*ITAG) Solyc10g086400.1.1 (e_va

Query	Observed	Mr(expt)	Mr(calc)	ppm	Miss	Score	Expect	Rank	Unique	Peptide
15259	469.5747	1405.7024	1405.7024	0.02	0	30	0.064	1		K.YNNVQLDGKPMK.I
18020	**866.9941**	**1731.9736**	**1731.9672**	**3.72**	**0**	**35**	**0.0096**	**1**	**U**	**K.IEFAGPNIGAPALPPIR.N**

Proteins matching the same set of peptides:
NbS00004226g0001.1 **Mass:** 16820 **Score:** 35 **Matches:** 2(1) **Sequences:** 2(1)
NbS00004226g0001.1 protein ; (*GB) gi|94466657|emb|CAJ44457.1| (e_value=2e-33) ALY protein [Nicotiana benthamiana];; (*TAIR) AT5G02530.2 (e_value=1e-14) | Symbols: | RNA-binding (RRM/RBD/RNP motifs

---

123. NbS00003461g0001.1 **Mass:** 14908 **Score:** 35 **Matches:** 2(1) **Sequences:** 2(1) **emPAI:** 0.23
NbS00003461g0001.1 protein AED:0.23 eAED:0.30 QI:0|-1|0|1|-1|1|1|0|132; (*GB) gi|94466657|emb|CAJ44457.1| (e_value=9e-45) ALY protein [Nicotiana benthamiana];; (*TAIR) AT5G59950.2 (e_value=7e-20)

Query	Observed	Mr(expt)	Mr(calc)	ppm	Miss	Score	Expect	Rank	Unique	Peptide
15259	469.5747	1405.7024	1405.7024	0.02	0	30	0.064	1		K.YNNVQLDGKPMK.I
18020	866.9941	1731.9736	1731.9672	3.72	0	35	0.0096	1	U	K.IEFAGPNIGAPALPPLR.N

Proteins matching the same set of peptides:
NbS00025223g0014.1 **Mass:** 15188 **Score:** 35 **Matches:** 2(1) **Sequences:** 2(1)
NbS00025223g0014.1 protein AED:0.37 eAED:0.37 QI:0|1|0.33|1|1|0.66|3|0|136; (*GB) gi|51490663|emb|CAG26902.1| (e_value=3e-63) ALY protein [Nicotiana benthamiana];; (*SWP) sp|Q86V81|THOC4_HUMAN (e_v

---

124. NbS00010099g0014.1 **Score:** 35 **Matches:** 1(1) **Sequences:** 1(1) **emPAI:** 0.57
NbS00010099g0014.1 protein AED:0.43 eAED:0.65 QI:0|0.5|0.33|1|0|0|3|29|57

Query	Observed	Mr(expt)	Mr(calc)	ppm	Miss	Score	Expect	Rank	Unique	Peptide
11602	464.2904	926.5662	926.5661	0.09	1	35	0.0093	1	U	R.RIEIQIR.K

---

125. NbS00009739g0005.1 **Mass:** 54154 **Score:** 35 **Matches:** 1(1) **Sequences:** 1(1) **emPAI:** 0.06
NbS00009739g0005.1 protein AED:0.12 eAED:0.15 QI:183|0.78|0.93|1|0.85|0.8|15|362|489; (*GB) gi|359495798|ref|XP_002262872.2| (e_value=0.0) PREDICTED: serine hydroxymethyltransferase, mitochondrial-

Query	Observed	Mr(expt)	Mr(calc)	ppm	Miss	Score	Expect	Rank	Unique	Peptide
12855	**546.3100**	**1090.6054**	**1090.6135**	**-7.35**	**0**	**35**	**0.022**	**1**	**U**	**K.LIVAGASAYAR.L**

Proteins matching the same set of peptides:
NbS00015224g0103.1 **Mass:** 67993 **Score:** 35 **Matches:** 1(1) **Sequences:** 1(1)
NbS00015224g0103.1 protein AED:0.15 eAED:0.16 QI:0|0.87|0.88|1|0.81|0.82|17|389|610
NbS00027670g0006.1 **Mass:** 59238 **Score:** 35 **Matches:** 1(1) **Sequences:** 1(1)
NbS00027670g0006.1 protein AED:0.24 eAED:0.24 QI:614|0.93|0.93|1|1|1|16|163|536; (*SWP) sp|P50433|GLYM_SOLTU (e_value=0.0) Serine hydroxymethyltransferase, mitochondrial OS=Solanum tuberosum PE=2 S
NbS00042478g0005.1 **Mass:** 57495 **Score:** 35 **Matches:** 1(1) **Sequences:** 1(1)
NbS00042478g0005.1 protein AED:0.25 eAED:0.25 QI:312|1|1|1|0.93|0.93|16|436|518; (*GB) gi|1707998|sp|P50433.1|GLYM_SOLTU (e_value=0.0) RecName: Full=Serine hydroxymethyltransferase, mitochondrial;
NbS00046993g0004.1 **Mass:** 67370 **Score:** 35 **Matches:** 1(1) **Sequences:** 1(1)
NbS00046993g0004.1 protein AED:0.27 eAED:0.28 QI:266|0.73|0.68|1|0.86|0.81|16|137|602; (*SWP) sp|P50433|GLYM_SOLTU (e_value=0.0) Serine hydroxymethyltransferase, mitochondrial OS=Solanum tuberosum

---

126. NbS00060216g0002.1 **Mass:** 52806 **Score:** 35 **Matches:** 1(1) **Sequences:** 1(1) **emPAI:** 0.06
NbS00060216g0002.1 protein AED:0.22 eAED:0.42 QI:0|0.33|0.25|1|1|1|4|324|479; (*GB) gi|356556739|ref|XP_003546680.1| (e_value=9e-113) PREDICTED: SUN domain-containing protein 1-like [Glycine max];;

Query	Observed	Mr(expt)	Mr(calc)	ppm	Miss	Score	Expect	Rank	Unique	Peptide

14083 630.8201 1259.6257 1259.6259 -0.12 0 35 0.025 1 U K.SGGGDVWSLLNR.N

127. NbS00005099g0011.1 **Mass:** 29831 **Score:** 35 **Matches:** 1(1) **Sequences:** 1(1) **emPAI:** 0.11
NbS00005099g0011.1 protein AED:0.26 eAED:0.26 QI:0|0.66|0.85|1|1|1|7|0|274; (*GB) gi|255552828|ref|XP_002517457.1| (e_value=2e-141) H(\+)-transporting atpase plant/fungi plasma membrane type, putat

Query	Observed	Mr(expt)	Mr(calc)	ppm	Miss	Score	Expect	Rank	Unique	Peptide
15239	702.8580	1403.7015	1403.7045	-2.12	0	35	0.026	1	U	R.SLGVAYQEVPEGR.K

Proteins matching the same set of peptides:
NbS00006964g0008.1 **Mass:** 79801 **Score:** 35 **Matches:** 1(1) **Sequences:** 1(1)
NbS00006964g0008.1 protein AED:0.23 eAED:0.23 QI:394|0.87|1|1|0.93|0.88|17|339|721; (*GB) gi|255552828|ref|XP_002517457.1| (e_value=0.0) H(\+)-transporting atpase plant/fungi plasma membrane type,
NbS00022217g0004.1 **Mass:** 78168 **Score:** 35 **Matches:** 1(1) **Sequences:** 1(1)
NbS00022217g0004.1 protein AED:0.21 eAED:0.21 QI:283|0.93|1|1|0.93|0.88|17|503|702; (*GB) gi|350535937|ref|NP_001234477.1| (e_value=0.0) plasma membrane H+-ATPase [Solanum lycopersicum];; (*SWP) sp

128. NbS00004507g0104.1 **Mass:** 22489 **Score:** 35 **Matches:** 1(1) **Sequences:** 1(1) **emPAI:** 0.15
NbS00004507g0104.1 protein

Query	Observed	Mr(expt)	Mr(calc)	ppm	Miss	Score	Expect	Rank	Unique	Peptide
12101	497.2717	992.5289	992.5035	25.6	0	35	0.03	1	U	-.MDLQMIVK.H

129. NbS00000485g0008.1 **Mass:** 26716 **Score:** 34 **Matches:** 1(1) **Sequences:** 1(1) **emPAI:** 0.12
NbS00000485g0008.1 protein AED:0.27 eAED:0.27 QI:81|1|1|1|0.8|0.83|6|329|238; (*GB) gi|225442156|ref|XP_002275541.1| (e_value=6e-155) PREDICTED: 40S ribosomal protein S3-3 [Vitis vinifera];; (*SWP)

Query	Observed	Mr(expt)	Mr(calc)	ppm	Miss	Score	Expect	Rank	Unique	Peptide
10262	712.3400	1422.6654	1422.6627	1.95	0	34	0.055	1	U	R.ELAEDGYSGVEVR.V

Proteins matching the same set of peptides:
NbS00003420g0016.1 **Mass:** 26587 **Score:** 34 **Matches:** 1(1) **Sequences:** 1(1)
NbS00003420g0016.1 protein AED:0.15 eAED:0.15 QI:81|1|1|1|0.8|1|6|333|237; (*GB) gi|358248410|ref|NP_001239621.1| (e_value=5e-156) uncharacterized protein LOC100808705 [Glycine max];; (*SWP) sp|Q9
NbS00035854g0012.1 **Mass:** 29045 **Score:** 34 **Matches:** 1(1) **Sequences:** 1(1)
NbS00035854g0012.1 protein AED:0.09 eAED:0.09 QI:143|1|0.85|1|0.83|0.71|7|0|260; (*GB) gi|225444782|ref|XP_002279950.1| (e_value=5e-151) PREDICTED: 40S ribosomal protein S3-3 [Vitis vinifera];; (*S

130. NbS00009983g0008.1 **Mass:** 42145 **Score:** 34 **Matches:** 2(1) **Sequences:** 2(1) **emPAI:** 0.08
NbS00009983g0008.1 protein AED:0.11 eAED:0.11 QI:0|0|0|1|0|0.5|2|0|384; (*GB) gi|108864705|gb|ABG22608.1| (e_value=0.0) Heat shock cognate 70 kDa protein, putative, expressed [Oryza sativa Japonica

Query	Observed	Mr(expt)	Mr(calc)	ppm	Miss	Score	Expect	Rank	Unique	Peptide
9658	616.3300	1230.6454	1230.6391	5.20	0	31	0.17	1	U	K.DAGVISGLNVMR.I
15483	718.8775	1435.7405	1435.7460	-3.84	0	34	0.027	1	U	K.VQQLLQDFFNGK.E

Proteins matching the same set of peptides:
NbS00022307g0013.1 **Mass:** 58674 **Score:** 34 **Matches:** 2(1) **Sequences:** 2(1)
NbS00022307g0013.1 protein AED:0.22 eAED:0.22 QI:0|0|0|1|0.66|0.5|4|0|536; (*GB) gi|108864705|gb|ABG22608.1| (e_value=0.0) Heat shock cognate 70 kDa protein, putative, expressed [Oryza sativa Japon
NbS00025223g0018.1 **Mass:** 65424 **Score:** 34 **Matches:** 2(1) **Sequences:** 2(1)
NbS00025223g0018.1 protein AED:0.09 eAED:0.10 QI:0|0|0|0.6|0.75|0.6|5|0|591; (*GB) gi|108864706|gb|ABG22609.1| (e_value=0.0) Heat shock cognate 70 kDa protein, putative, expressed [Oryza sativa Jap
NbS00027735g0001.1 **Mass:** 70349 **Score:** 34 **Matches:** 2(1) **Sequences:** 2(1)
NbS00027735g0001.1 protein AED:0.18 eAED:0.18 QI:0|0|0|0.5|1|1|2|0|633; (*GB) gi|123650|sp|P09189.1|HSP7C_PETHY (e_value=0.0) RecName: Full=Heat shock cognate 70 kDa protein;; (*SWP) sp|P09189|HSP
NbS00037714g0003.1 **Mass:** 53453 **Score:** 34 **Matches:** 2(1) **Sequences:** 2(1)
NbS00037714g0003.1 protein AED:0.12 eAED:0.12 QI:0|0|0|0.75|1|1|4|0|487; (*GB) gi|38325815|gb|AAR17080.1| (e_value=0.0) heat shock protein 70-3 [Nicotiana tabacum];; (*SWP) sp|P27322|HSP72_SOLLC (e
NbS00039744g0003.1 **Mass:** 67714 **Score:** 34 **Matches:** 2(1) **Sequences:** 2(1)
NbS00039744g0003.1 protein AED:0.25 eAED:0.32 QI:0|0|0|0.75|1|1|4|0|618; (*GB) gi|123620|sp|P27322.1|HSP72_SOLLC (e_value=0.0) RecName: Full=Heat shock cognate 70 kDa protein 2;; (*SWP) sp|P27322|H

131. NbS00006644g0116.1 **Mass:** 121848 **Score:** 34 **Matches:** 1(1) **Sequences:** 1(1) **emPAI:** 0.03
NbS00006644g0116.1 protein AED:0.00 eAED:0.00 QI:364|1|1|1|1|1|3|541|1093

Query	Observed	Mr(expt)	Mr(calc)	ppm	Miss	Score	Expect	Rank	Unique	Peptide
14878	676.3367	1350.6588	1350.6568	1.47	0	34	0.03	1	U	R.WALIDFSSQER.N

132. NbS00011208g0006.1 **Mass:** 53003 **Score:** 34 **Matches:** 2(2) **Sequences:** 2(2) **emPAI:** 0.13

NbS00011208g0006.1 protein AED:0.18 eAED:0.20 QI:0|0.81|0.83|1|1|1|12|47|443; (*GB) gi|225430786|ref|XP_002270129.1| (e_value=1e-97) PREDICTED: U1 small nuclear ribonucleoprotein 70 kDa-like [Vitis

Query	Observed	Mr(expt)	Mr(calc)	ppm	Miss	Score	Expect	Rank	Unique	Peptide
10978	415.2425	828.4704	828.4705	-0.10	0	32	0.048	1	U	R.VLVDVER.G
13003	556.7664	1111.5183	1111.5145	3.37	0	31	0.046	1	U	R.LNYETTESR.V

Proteins matching the same set of peptides:
NbS00015731g0004.1 **Mass:** 56280 **Score:** 34 **Matches:** 2(2) **Sequences:** 2(2)
NbS00015731g0004.1 protein AED:0.34 eAED:0.35 QI:0|0.35|0.46|0.8|0.92|1|15|216|479; (*GB) gi|255561192|ref|XP_002521608.1| (e_value=8e-112) U1 small nuclear ribonucleoprotein 70 kDa, putative [Ric

133. NbS00019490g0007.1 **Mass:** 28653 **Score:** 33 **Matches:** 1(1) **Sequences:** 1(1) **emPAI:** 0.12
NbS00019490g0007.1 protein AED:0.32 eAED:0.34 QI:300|0.66|0.42|0.85|0.5|0.71|7|0|253; (*SWP) sp|O14327|PAB2_SCHPO (e_value=2e-38) Polyadenylate-binding protein 2 OS=Schizosaccharomyces pombe (stra

Query	Observed	Mr(expt)	Mr(calc)	ppm	Miss	Score	Expect	Rank	Unique	Peptide
14802	672.3640	1342.7135	1342.7092	3.19	1	33	0.031	1	U	R.LKEIEDEAGALR.E

Proteins matching the same set of peptides:
NbS00019985g0011.1 **Mass:** 10377 **Score:** 33 **Matches:** 1(1) **Sequences:** 1(1)
NbS00019985g0011.1 protein AED:0.02 eAED:0.02 QI:0|1|0.5|1|1|1|2|0|91; (*GB) gi|224134274|ref|XP_002321779.1| (e_value=1e-22) predicted protein [Populus trichocarpa];; (*TAIR) AT5G65260.1 (e_value=
NbS00034222g0006.1 **Mass:** 9254 **Score:** 33 **Matches:** 1(1) **Sequences:** 1(1)
NbS00034222g0006.1 protein AED:0.32 eAED:0.32 QI:0|1|0.5|1|1|0.5|2|0|81; (*GB) gi|224114678|ref|XP_002339510.1| (e_value=2e-21) predicted protein [Populus trichocarpa];; (*TAIR) AT5G65260.1 (e_valu
NbC24974876g0004.1 **Mass:** 7280 **Score:** 33 **Matches:** 1(1) **Sequences:** 1(1)
NbC24974876g0004.1 protein AED:0.13 eAED:0.15 QI:0|1|0|1|1|1|2|0|65; (*GB) gi|224134274|ref|XP_002321779.1| (e_value=1e-16) predicted protein [Populus trichocarpa];; (*TAIR) AT5G51120.2 (e_value=3e

134. NbS00003570g0008.1 **Mass:** 26757 **Score:** 33 **Matches:** 1(1) **Sequences:** 1(1) **emPAI:** 0.12
NbS00003570g0008.1 protein AED:0.13 eAED:0.14 QI:0|0|0|0.6|0.5|0.6|5|0|237; (*GB) gi|40748265|gb|AAR89617.1| (e_value=1e-130) 40S ribosomal protein S5 [Capsicum annuum];; (*SWP) sp|P51427|RS52_ARA

Query	Observed	Mr(expt)	Mr(calc)	ppm	Miss	Score	Expect	Rank	Unique	Peptide
15364	710.4019	1418.7893	1418.7881	0.80	0	33	0.024	1	U	R.VNQAIYLLTTGAR.E

Proteins matching the same set of peptides:
NbS00011165g0113.1 **Mass:** 33956 **Score:** 33 **Matches:** 1(1) **Sequences:** 1(1)
NbS00011165g0113.1 protein AED:0.03 eAED:0.03 QI:141|0.66|0.75|1|0.66|0.5|4|369|294
NbS00024629g0018.1 **Mass:** 23054 **Score:** 33 **Matches:** 1(1) **Sequences:** 1(1)
NbS00024629g0018.1 protein AED:0.24 eAED:0.24 QI:96|0.66|0.5|1|0.33|0.25|4|0|206; (*GB) gi|40748265|gb|AAR89617.1| (e_value=1e-138) 40S ribosomal protein S5 [Capsicum annuum];; (*SWP) sp|P51427|RS
NbS00035760g0016.1 **Mass:** 23224 **Score:** 33 **Matches:** 1(1) **Sequences:** 1(1)
NbS00035760g0016.1 protein AED:0.11 eAED:0.11 QI:93|0.75|0.6|1|0.75|0.8|5|403|207; (*GB) gi|40748265|gb|AAR89617.1| (e_value=4e-140) 40S ribosomal protein S5 [Capsicum annuum];; (*SWP) sp|P51427|R
NbS00054406g0002.1 **Mass:** 15684 **Score:** 33 **Matches:** 1(1) **Sequences:** 1(1)
NbS00054406g0002.1 protein AED:0.02 eAED:0.02 QI:0|-1|0|1|-1|1|1|0|139; (*GB) gi|40748265|gb|AAR89617.1| (e_value=2e-71) 40S ribosomal protein S5 [Capsicum annuum];; (*SWP) sp|Q9ZUT9|RS51_ARATH (e_
NbS00055607g0001.1 **Mass:** 24871 **Score:** 33 **Matches:** 1(1) **Sequences:** 1(1)
NbS00055607g0001.1 protein AED:0.24 eAED:0.24 QI:133|0.66|0.5|1|1|1|4|361|222; (*GB) gi|40748265|gb|AAR89617.1| (e_value=5e-117) 40S ribosomal protein S5 [Capsicum annuum];; (*SWP) sp|P51427|RS52_

135. NbS00004226g0004.1 **Mass:** 14244 **Score:** 33 **Matches:** 1(1) **Sequences:** 1(1) **emPAI:** 0.24
NbS00004226g0004.1 protein ; (*GB) gi|94466657|emb|CAJ44457.1| (e_value=4e-18) ALY protein [Nicotiana benthamiana];; (*ITAG) Solyc10g086400.1.1 (e_value=3e-16) evidence_code:10F1H1E1IEG genomic_re

Query	Observed	Mr(expt)	Mr(calc)	ppm	Miss	Score	Expect	Rank	Unique	Peptide
13343	580.3195	1158.6245	1158.6245	0.03	0	33	0.041	1	U	R.GAGGISGIETGIK.L

Proteins matching the same set of peptides:
NbS00025223g0004.1 **Mass:** 16135 **Score:** 33 **Matches:** 1(1) **Sequences:** 1(1)
NbS00025223g0004.1 protein ; (*GB) gi|51490663|emb|CAG26902.1| (e_value=4e-28) ALY protein [Nicotiana benthamiana];; (*ITAG) Solyc10g086400.1.1 (e_value=5e-19) evidence_code:10F1H1E1IEG genomic_re

136. NbS00021398g0012.1 **Mass:** 92487 **Score:** 33 **Matches:** 1(1) **Sequences:** 1(1) **emPAI:** 0.04
NbS00021398g0012.1 protein AED:0.33 eAED:0.35 QI:0|0.81|0.75|1|0.81|0.75|12|0|838; (*GB) gi|225450401|ref|XP_002278318.1| (e_value=0.0) PREDICTED: DEAD-box ATP-dependent RNA helicase 3, chloroplast

Query	Observed	Mr(expt)	Mr(calc)	ppm	Miss	Score	Expect	Rank	Unique	Peptide
9737	635.8700	1269.7254	1269.7292	-2.97	0	33	0.024	1	U	R.LIDLINNNTLK.L

Proteins matching the same set of peptides:
NbS00058562g0005.1 **Mass:** 80323 **Score:** 33 **Matches:** 1(1) **Sequences:** 1(1)

NbS00058562g0005.1 protein AED:0.30 eAED:0.32 QI:0|0.77|0.8|1|0.88|0.7|10|0|734; (*SWP) sp|Q8L7S8|RH3_ARATH (e_value=0.0) DEAD-box ATP-dependent RNA helicase 3, chloroplastic OS=Arabidopsis thaliar

---

137. NbS00002113g0001.1 **Mass:** 43922 **Score:** 33 **Matches:** 1(1) **Sequences:** 1(1) **emPAI:** 0.08

NbS00002113g0001.1 protein AED:0.00 eAED:0.05 QI:0|-1|0|1|-1|1|1|0|394; (*SWP) sp|Q00874|DR100_ARATH (e_value=1e-127) DNA-damage-repair/toleration protein DRT100 OS=Arabidopsis thaliana GN=DRT100 I

Query	Observed	Mr(expt)	Mr(calc)	ppm	Miss	Score	Expect	Rank	Unique	Peptide
13036	**559.3091**	**1116.6036**	**1116.6026**	**0.88**	**0**	**33**	**0.048**	**1**	**U**	**R.LSDLDLSLNK.L**

---

138. NbS00005637g0007.1 **Mass:** 102029 **Score:** 33 **Matches:** 1(1) **Sequences:** 1(1) **emPAI:** 0.03

NbS00005637g0007.1 protein AED:0.01 eAED:0.01 QI:0|1|0.5|1|1|1|2|0|892; (*GB) gi|15144509|gb|AAK84476.1| (e_value=0.0) unknown [Solanum lycopersicum];; (*TAIR) AT1G58210.1 (e_value=0.0) | Symbols:

Query	Observed	Mr(expt)	Mr(calc)	ppm	Miss	Score	Expect	Rank	Unique	Peptide
1454	**323.1500**	**966.4282**	**966.4658**	**-38.92**	**1**	**33**	**0.031**	**1**	**U**	**R.EASKEFEK.I**

---

139. NbS00006911g0001.1 **Mass:** 47718 **Score:** 33 **Matches:** 1(1) **Sequences:** 1(1) **emPAI:** 0.07

NbS00006911g0001.1 protein AED:0.02 eAED:0.02 QI:286|0|0.5|1|0|0.5|2|0|422; (*GB) gi|347953950|gb|AEP33595.1| (e_value=0.0) CBL-interacting protein kinase 6 [Solanum lycopersicum];; (*SWP) sp|O655!

Query	Observed	Mr(expt)	Mr(calc)	ppm	Miss	Score	Expect	Rank	Unique	Peptide
1454	323.1500	966.4282	966.4514	-24.07	0	33	0.031	1	U	K.VGMMEQIK.R

Proteins matching the same set of peptides:

NbS00044379g0001.1 **Mass:** 47801 **Score:** 33 **Matches:** 1(1) **Sequences:** 1(1)

NbS00044379g0001.1 protein AED:0.25 eAED:0.25 QI:0|-1|0|1|-1|1|1|0|425; (*GB) gi|347953950|gb|AEP33595.1| (e_value=0.0) CBL-interacting protein kinase 6 [Solanum lycopersicum];; (*SWP) sp|O65554|C]

---

140. NbS00018511g0004.1 **Mass:** 39588 **Score:** 33 **Matches:** 1(1) **Sequences:** 1(1) **emPAI:** 0.08

NbS00018511g0004.1 protein AED:0.03 eAED:0.03 QI:152|1|1|1|0.25|0.2|5|139|338; (*GB) gi|29825611|gb|AAO92303.1| (e_value=0.0) gibberellin 2-oxidase 1 [Nicotiana sylvestris];; (*SWP) sp|O49561|G2OX8

Query	Observed	Mr(expt)	Mr(calc)	ppm	Miss	Score	Expect	Rank	Unique	Peptide
1454	323.1500	966.4282	966.4626	-35.67	1	33	0.031	1	U	K.MRMEQIK.L

---

141. NbS00001711g0013.1 **Mass:** 36166 **Score:** 33 **Matches:** 1(1) **Sequences:** 1(1) **emPAI:** 0.09

NbS00001711g0013.1 protein AED:0.26 eAED:0.26 QI:422|0.85|0.87|1|0.85|0.75|8|401|334; (*TAIR) AT4G17520.1 (e_value=2e-31) | Symbols: | Hyaluronan / mRNA binding family | chr4:9771496-9773313 FORWAI

Query	Observed	Mr(expt)	Mr(calc)	ppm	Miss	Score	Expect	Rank	Unique	Peptide
12927	**551.7994**	**1101.5843**	**1101.5852**	**-0.79**	**0**	**33**	**0.048**	**1**	**U**	**K.GLLALMAEER.K**

---

142. NbS00010073g0013.1 **Score:** 33 **Matches:** 1(1) **Sequences:** 1(1) **emPAI:** 0.14

NbS00010073g0013.1 protein AED:0.19 eAED:0.22 QI:0|0.66|0.75|1|0.33|0.25|4|0|220; (*SWP) sp|Q9LX82|MYB48_ARATH (e_value=5e-48) Transcription factor MYB48 OS=Arabidopsis thaliana GN=MYB48 PE=2 SV=1;

Query	Observed	Mr(expt)	Mr(calc)	ppm	Miss	Score	Expect	Rank	Unique	Peptide
11050	**421.2789**	**840.5432**	**840.5433**	**-0.03**	**0**	**33**	**0.015**	**1**	**U**	**R.LIVQLQK.Q**

---

143. NbS00013320g0008.1 **Score:** 33 **Matches:** 1(1) **Sequences:** 1(1) **emPAI:** 0.05

NbS00013320g0008.1 protein AED:0.19 eAED:0.25 QI:82|0.66|0.69|1|0.75|0.76|13|0|616; (*GB) gi|297734868|emb|CBI17102.3| (e_value=5e-160) unnamed protein product [Vitis vinifera];; (*TAIR) AT5G22820.

Query	Observed	Mr(expt)	Mr(calc)	ppm	Miss	Score	Expect	Rank	Unique	Peptide
11050	421.2789	840.5432	840.5433	-0.03	0	33	0.015	1	U	R.LLVQIQK.T

---

144. NbS00038102g0001.1 **Score:** 33 **Matches:** 1(1) **Sequences:** 1(1) **emPAI:** 0.28

NbS00038102g0001.1 protein

Query	Observed	Mr(expt)	Mr(calc)	ppm	Miss	Score	Expect	Rank	Unique	Peptide
11050	421.2789	840.5432	840.5797	-43.31	1	33	0.015	1	U	K.LLVKLQK.R

---

145. NbS00018023g0003.1 **Mass:** 48157 **Score:** 33 **Matches:** 1(1) **Sequences:** 1(1) **emPAI:** 0.07

NbS00018023g0003.1 protein AED:0.25 eAED:0.25 QI:0|1|0.75|1|1|1|4|510|435; (*GB) gi|297845476|ref|XP_002890619.1| (e_value=2e-95) hypothetical protein ARALYDRAFT_472693 [Arabidopsis lyrata subsp. I

Query	Observed	Mr(expt)	Mr(calc)	ppm	Miss	Score	Expect	Rank	Unique	Peptide
14071	**629.8491**	**1257.6837**	**1257.6816**	**1.67**	**0**	**33**	**0.038**	**1**	**U**	**K.SAVLAGIEELEK.L**

---

146. NbS00001635g0020.1 **Mass:** 40084 **Score:** 33 **Matches:** 1(1) **Sequences:** 1(1) **emPAI:** 0.08
NbS00001635g0020.1 protein AED:0.07 eAED:0.07 QI:0|0.4|0.33|1|0.8|0.5|6|0|371; (*SWP) sp|Q944G9|ALFC2_ARATH (e_value=0.0) Probable fructose-bisphosphate aldolase 2, chloroplastic OS=Arabidopsis tha

Query	Observed	Mr(expt)	Mr(calc)	ppm	Miss	Score	Expect	Rank	Unique	Peptide
15130	694.3631	1386.7116	1386.7103	0.99	0	33	0.041	1	U	R.LASIGLENTEANR.Q

Proteins matching the same set of peptides:
NbS00004219g0021.1 **Mass:** 50083 **Score:** 33 **Matches:** 1(1) **Sequences:** 1(1)
NbS00004219g0021.1 protein AED:0.09 eAED:0.09 QI:0|0.66|0.57|1|0.83|0.85|7|0|463; (*GB) gi|354464671|gb|AER26531.1| (e_value=0.0) fructose 1,6 bisphosphate aldolase class 1 [Carica papaya];; (*SWP)
NbS00007016g0020.1 **Mass:** 42498 **Score:** 33 **Matches:** 1(1) **Sequences:** 1(1)
NbS00007016g0020.1 protein AED:0.12 eAED:0.12 QI:0|0.8|0.66|1|0.8|0.83|6|0|391; (*GB) gi|4827251|dbj|BAA77604.1| (e_value=0.0) plastidic aldolase NPALDP1 [Nicotiana paniculata];; (*SWP) sp|Q944G9|
NbS00008558g0004.1 **Mass:** 43072 **Score:** 33 **Matches:** 1(1) **Sequences:** 1(1)
NbS00008558g0004.1 protein AED:0.13 eAED:0.13 QI:0|1|0.8|1|1|1|5|0|398; (*GB) gi|4827253|dbj|BAA77603.1| (e_value=0.0) plastidic aldolase [Nicotiana paniculata];; (*SWP) sp|Q944G9|ALFC2_ARATH (e_va
NbS00014581g0001.1 **Mass:** 42279 **Score:** 33 **Matches:** 1(1) **Sequences:** 1(1)
NbS00014581g0001.1 protein AED:0.17 eAED:0.17 QI:214|0.83|0.71|1|0.83|0.71|7|0|390; (*GB) gi|4827251|dbj|BAA77604.1| (e_value=0.0) plastidic aldolase NPALDP1 [Nicotiana paniculata];; (*SWP) sp|Q94
NbS00028064g0012.1 **Mass:** 43060 **Score:** 33 **Matches:** 1(1) **Sequences:** 1(1)
NbS00028064g0012.1 protein AED:0.27 eAED:0.27 QI:0|1|0.8|1|1|1|5|0|398; (*GB) gi|4827253|dbj|BAA77603.1| (e_value=0.0) plastidic aldolase [Nicotiana paniculata];; (*SWP) sp|Q944G9|ALFC2_ARATH (e_va
NbS00042047g0015.1 **Mass:** 42812 **Score:** 33 **Matches:** 1(1) **Sequences:** 1(1)
NbS00042047g0015.1 protein AED:0.19 eAED:0.19 QI:212|1|0.83|1|1|1|6|0|395; (*GB) gi|4827251|dbj|BAA77604.1| (e_value=0.0) plastidic aldolase NPALDP1 [Nicotiana paniculata];; (*SWP) sp|Q944G9|ALFC2_
NbS00045171g0004.1 **Mass:** 41190 **Score:** 33 **Matches:** 1(1) **Sequences:** 1(1)
NbS00045171g0004.1 protein AED:0.08 eAED:0.08 QI:0|0.5|0.6|1|0.75|0.8|5|310|377; (*GB) gi|4827253|dbj|BAA77603.1| (e_value=0.0) plastidic aldolase [Nicotiana paniculata];; (*SWP) sp|Q944G9|ALFC2_AR

147. NbS00013764g0007.1 **Mass:** 53134 **Score:** 33 **Matches:** 1(1) **Sequences:** 1(1) **emPAI:** 0.06
NbS00013764g0007.1 protein AED:0.11 eAED:0.11 QI:116|0.85|0.87|1|0.85|0.75|8|606|455; (*GB) gi|1705613|sp|P49319.2|CATA1_TOBAC (e_value=0.0) RecName: Full=Catalase isozyme 1; AltName: Full=Salicyl

Query	Observed	Mr(expt)	Mr(calc)	ppm	Miss	Score	Expect	Rank	Unique	Peptide
12792	542.7758	1083.5371	1083.5349	2.02	0	33	0.034	1	U	R.IFAYADTQR.H

148. NbS00025410g0006.1 **Mass:** 115303 **Score:** 32 **Matches:** 1(1) **Sequences:** 1(1) **emPAI:** 0.03
NbS00025410g0006.1 protein AED:0.12 eAED:0.12 QI:214|0.95|0.86|1|1|1|22|0|1037; (*GB) gi|255570428|ref|XP_002526173.1| (e_value=0.0) suppressor of ty, putative [Ricinus communis];; (*SWP) sp|Q9STN

Query	Observed	Mr(expt)	Mr(calc)	ppm	Miss	Score	Expect	Rank	Unique	Peptide
15126	463.2257	1386.6553	1386.6528	1.81	0	32	0.032	1	U	R.YGLGSETPSHPSR.T

149. NbS00049664g0004.1 **Mass:** 25462 **Score:** 32 **Matches:** 1(1) **Sequences:** 1(1) **emPAI:** 0.13
NbS00049664g0004.1 protein AED:0.29 eAED:0.29 QI:0|0.6|0.33|0.66|1|0.83|6|0|236; (*GB) gi|584795|sp|Q08436.1|PMA3_NICPL (e_value=1e-107) RecName: Full=Plasma membrane ATPase 3; AltName: Full=Proto

Query	Observed	Mr(expt)	Mr(calc)	ppm	Miss	Score	Expect	Rank	Unique	Peptide
16442	779.9203	1557.8261	1557.8250	0.74	0	32	0.032	1	U	K.IDQSALTGESLPVTK.G

150. NbS00002520g0008.1 **Mass:** 37714 **Score:** 32 **Matches:** 1(1) **Sequences:** 1(1) **emPAI:** 0.09
NbS00002520g0008.1 protein AED:0.16 eAED:0.17 QI:128|1|0.75|1|1|1|4|0|340; (*GB) gi|384038823|gb|AFH58002.1| (e_value=0.0) chloroplast PsbP2 precursor [Nicotiana benthamiana];; (*SWP) sp|P18212|PSB

Query	Observed	Mr(expt)	Mr(calc)	ppm	Miss	Score	Expect	Rank	Unique	Peptide
16789	804.8438	1607.6731	1607.6740	-0.59	0	32	0.012	1	U	K.TDTDFQTYNGDGFK.L

Proteins matching the same set of peptides:
NbS00028820g0007.1 **Mass:** 27567 **Score:** 32 **Matches:** 1(1) **Sequences:** 1(1)
NbS00028820g0007.1 protein AED:0.28 eAED:0.28 QI:0|0.66|0.5|1|1|1|4|0|251; (*GB) gi|384038821|gb|AFH58001.1| (e_value=4e-173) chloroplast PsbP1 precursor [Nicotiana benthamiana];; (*SWP) sp|P18212

151. NbS00002523g0003.1 **Mass:** 78674 **Score:** 32 **Matches:** 1(1) **Sequences:** 1(1) **emPAI:** 0.04
NbS00002523g0003.1 protein AED:0.18 eAED:0.18 QI:0|-1|0|1|-1|1|1|0|704; (*GB) gi|75249421|sp|Q93YF5.1|SUVH1_TOBAC (e_value=0.0) RecName: Full=Histone-lysine N-methyltransferase, H3 lysine-9 specif

Query	Observed	Mr(expt)	Mr(calc)	ppm	Miss	Score	Expect	Rank	Unique	Peptide
11942	486.7621	971.5096	971.5076	2.05	0	32	0.045	1	U	R.VPGQPEAFK.V

Proteins matching the same set of peptides:
NbS00004793g0007.1 **Mass:** 83339 **Score:** 32 **Matches:** 1(1) **Sequences:** 1(1)
NbS00004793g0007.1 protein AED:0.17 eAED:0.17 QI:0|-1|0|1|-1|1|1|0|749; (*SWP) sp|Q93YF5|SUVH1_TOBAC (e_value=0.0) Histone-lysine N-methyltransferase, H3 lysine-9 specific SUVH1 OS=Nicotiana tabacu

152. NbS00006116g0019.1 **Mass:** 55149 **Score:** 32 **Matches:** 1(1) **Sequences:** 1(1) **emPAI:** 0.06
NbS00006116g0019.1 protein AED:0.27 eAED:0.27 QI:568|1|1|1|1|1|7|0|473; (*GB) gi|219560127|gb|ACL27272.1| (e_value=0.0) catalase [Nicotiana benthamiana];; (*SWP) sp|P49315|CATA1_NICPL (e_value=0.0)

Query	Observed	Mr(expt)	Mr(calc)	ppm	Miss	Score	Expect	Rank	Unique	Peptide
12713	536.7745	1071.5344	1071.5349	-0.47	0	32	0.04	1	U	R.WVEALSDPR.I

Proteins matching the same set of peptides:
NbS00012784g0015.1 **Mass:** 51219 **Score:** 32 **Matches:** 1(1) **Sequences:** 1(1)
NbS00012784g0015.1 protein AED:0.24 eAED:0.24 QI:310|0.71|0.75|0.87|0.85|0.87|8|0|437; (*GB) gi|2459684|gb|AAB71764.1| (e_value=0.0) catalase 1 [Nicotiana tabacum];; (*SWP) sp|P49315|CATA1_NICPL (e

---

153. NbS00008675g0003.1 **Mass:** 54451 **Score:** 32 **Matches:** 1(1) **Sequences:** 1(1) **emPAI:** 0.06
NbS00008675g0003.1 protein AED:0.03 eAED:0.03 QI:0|0|0|1|1|1|2|0|492; (*GB) gi|75216452|sp|Q9ZS34.1|CHLP_TOBAC (e_value=0.0) RecName: Full=Geranylgeranyl diphosphate reductase, chloroplastic; AltNa

Query	Observed	Mr(expt)	Mr(calc)	ppm	Miss	Score	Expect	Rank	Unique	Peptide
16631	798.4413	1594.8680	1594.8679	0.09	0	32	0.027	1	U	R.VAVVGGGPAGGAAAETLAK.G

Proteins matching the same set of peptides:
NbS00056940g0001.1 **Mass:** 51663 **Score:** 32 **Matches:** 1(1) **Sequences:** 1(1)
NbS00056940g0001.1 protein AED:0.00 eAED:0.00 QI:0|-1|0|1|-1|1|1|0|464; (*GB) gi|75216452|sp|Q9ZS34.1|CHLP_TOBAC (e_value=0.0) RecName: Full=Geranylgeranyl diphosphate reductase, chloroplastic; Alt

---

154. NbS00007190g0001.1 **Mass:** 30678 **Score:** 32 **Matches:** 1(1) **Sequences:** 1(1) **emPAI:** 0.11
NbS00007190g0001.1 protein AED:0.08 eAED:0.08 QI:179|1|1|1|0.5|0.33|3|443|275; (*GB) gi|225448932|ref|XP_002267178.1| (e_value=1e-93) PREDICTED: heterogeneous nuclear ribonucleoprotein G [Vitis vir

Query	Observed	Mr(expt)	Mr(calc)	ppm	Miss	Score	Expect	Rank	Unique	Peptide
15168	697.3688	1392.7231	1392.7249	-1.28	0	32	0.039	1	U	R.IFVGGLSSDITER.Q

Proteins matching the same set of peptides:
NbS00029940g0009.1 **Mass:** 30714 **Score:** 32 **Matches:** 1(1) **Sequences:** 1(1)
NbS00029940g0009.1 protein AED:0.36 eAED:0.36 QI:205|1|1|1|0.5|0.66|3|523|275; (*GB) gi|225448932|ref|XP_002267178.1| (e_value=6e-80) PREDICTED: heterogeneous nuclear ribonucleoprotein G [Vitis vir

---

155. NbS00000548g0008.1 **Mass:** 40225 **Score:** 32 **Matches:** 1(1) **Sequences:** 1(1) **emPAI:** 0.08
NbS00000548g0008.1 protein AED:0.29 eAED:0.30 QI:0|0.8|0.5|1|1|0.66|6|0|363; (*GB) gi|255573386|ref|XP_002527619.1| (e_value=2e-130) Ras-GTPase-activating protein-binding protein, putative [Ricinu:

Query	Observed	Mr(expt)	Mr(calc)	ppm	Miss	Score	Expect	Rank	Unique	Peptide
20710	884.7487	2651.2243	2651.2207	1.35	0	32	0.023	1	U	R.FYQDSSVLSRPDSNGTMTSVTTMK.N

---

156. NbS00010498g0007.1 **Mass:** 24174 **Score:** 32 **Matches:** 1(0) **Sequences:** 1(0) **emPAI:** 0.14
NbS00010498g0007.1 protein AED:0.12 eAED:0.12 QI:0|1|1|1|0.5|1|3|646|228; (*GB) gi|384038831|gb|AFH58006.1| (e_value=5e-128) chloroplast PsbQ2 precursor [Nicotiana benthamiana];; (*SWP) sp|P12301|I

Query	Observed	Mr(expt)	Mr(calc)	ppm	Miss	Score	Expect	Rank	Unique	Peptide
14091	631.3179	1260.6212	1260.6251	-3.11	0	32	0.055	1	U	K.AWPYVQNDLR.L

---

157. NbS00011355g0109.1 **Mass:** 19836 **Score:** 31 **Matches:** 1(0) **Sequences:** 1(0) **emPAI:** 0.17
NbS00011355g0109.1 protein AED:0.07 eAED:0.07 QI:222|1|1|1|1|1|5|560|168

Query	Observed	Mr(expt)	Mr(calc)	ppm	Miss	Score	Expect	Rank	Unique	Peptide
10090	683.3400	1364.6654	1364.6572	6.04	0	31	0.12	1	U	R.AFDDVNSQLQTK.F

Proteins matching the same set of peptides:
NbS00016020g0006.1 **Mass:** 19781 **Score:** 31 **Matches:** 1(0) **Sequences:** 1(0)
NbS00016020g0006.1 protein AED:0.04 eAED:0.04 QI:156|1|1|1|1|1|5|441|168; (*SWP) sp|Q9FT52|ATP5H_ARATH (e_value=1e-93) ATP synthase subunit d, mitochondrial OS=Arabidopsis thaliana GN=At3g52300 PE=
NbS00033972g0005.1 **Mass:** 19751 **Score:** 31 **Matches:** 1(0) **Sequences:** 1(0)
NbS00033972g0005.1 protein AED:0.11 eAED:0.11 QI:170|1|1|1|1|1|5|383|168; (*GB) gi|48209968|gb|AAT40531.1| (e_value=1e-103) ATP synthase D chain, mitochondrial, putative [Solanum demissum];; (*SWP)

---

158. NbS00028210g0125.1 **Score:** 31 **Matches:** 1(1) **Sequences:** 1(1) **emPAI:** 0.04
NbS00028210g0125.1 protein AED:0.13 eAED:0.14 QI:0|0|0|0.94|0.93|0.94|17|0|680

Query	Observed	Mr(expt)	Mr(calc)	ppm	Miss	Score	Expect	Rank	Unique	Peptide
15148	695.8451	1389.6756	1389.7326	-40.98	1	31	0.055	2	U	R.STAEMWPSLIKK.A

---

159. NbS00005651g0008.1 **Mass:** 53710 **Score:** 31 **Matches:** 1(1) **Sequences:** 1(1) **emPAI:** 0.06
NbS00005651g0008.1 protein AED:0.24 eAED:0.24 QI:27|0.62|0.33|1|0.5|0.44|9|0|470; (*GB) gi|175363751|gb|ACB72462.1| (e_value=0.0) elongation factor 1 gamma-like protein [Nicotiana tabacum];; (*SWP)

Query	Observed	Mr(expt)	Mr(calc)	ppm	Miss	Score	Expect	Rank	Unique	Peptide
13699	604.3261	1206.6375	1206.6318	4.75	0	31	0.054	1	U	K.MLVIGSEAPYK.V

Proteins matching the same set of peptides:
NbS00006811g0211.1 **Mass:** 90233 **Score:** 31 **Matches:** 1(1) **Sequences:** 1(1)
NbS00006811g0211.1 protein AED:0.22 eAED:0.23 QI:0|0.6|0.36|1|0.7|0.54|11|0|778
NbS00015969g0002.1 **Mass:** 50891 **Score:** 31 **Matches:** 1(1) **Sequences:** 1(1)
NbS00015969g0002.1 protein AED:0.18 eAED:0.18 QI:201|0.71|0.75|1|1|1|8|0|442; (*GB) gi|175363751|gb|ACB72462.1| (e_value=0.0) elongation factor 1 gamma-like protein [Nicotiana tabacum];; (*SWP) sp

---

160. NbS00005673g0011.1 **Mass:** 80468 **Score:** 31 **Matches:** 1(1) **Sequences:** 1(1) **emPAI:** 0.04
NbS00005673g0011.1 protein AED:0.19 eAED:0.19 QI:0|0.8|0.63|1|0.8|0.81|11|273|722; (*GB) gi|356547867|ref|XP_003542326.1| (e_value=0.0) PREDICTED: 5-methyltetrahydropteroyltriglutamate--homocystein

Query	Observed	Mr(expt)	Mr(calc)	ppm	Miss	Score	Expect	Rank	Unique	Peptide
12888	548.7925	1095.5704	1095.5713	-0.82	0	31	0.052	1	U	K.YLFAGVVDGR.N

Proteins matching the same set of peptides:
NbS00007447g0013.1 **Mass:** 67417 **Score:** 31 **Matches:** 1(1) **Sequences:** 1(1)
NbS00007447g0013.1 protein AED:0.23 eAED:0.34 QI:9|0.22|0|0.9|0.44|0.3|10|0|607; (*SWP) sp|Q42699|METE_CATRO (e_value=2e-176) 5-methyltetrahydropteroyltriglutamate--homocysteine methyltransferase (
NbS00010381g0002.1 **Mass:** 17184 **Score:** 31 **Matches:** 1(1) **Sequences:** 1(1)
NbS00010381g0002.1 protein ; (*GB) gi|356547867|ref|XP_003542326.1| (e_value=1e-72) PREDICTED: 5-methyltetrahydropteroyltriglutamate--homocysteine methyltransferase-like [Glycine max];; (*SWP) sp|(
NbS00011007g0119.1 **Mass:** 84742 **Score:** 31 **Matches:** 1(1) **Sequences:** 1(1)
NbS00011007g0119.1 protein AED:0.10 eAED:0.10 QI:52|1|1|1|1|1|11|282|765
NbS00012577g0009.1 **Mass:** 84751 **Score:** 31 **Matches:** 1(1) **Sequences:** 1(1)
NbS00012577g0009.1 protein AED:0.13 eAED:0.13 QI:321|1|1|1|0.9|0.81|11|196|765; (*SWP) sp|Q42699|METE_CATRO (e_value=0.0) 5-methyltetrahydropteroyltriglutamate--homocysteine methyltransferase OS=Ca
NbS00017361g0009.1 **Mass:** 84814 **Score:** 31 **Matches:** 1(1) **Sequences:** 1(1)
NbS00017361g0009.1 protein AED:0.18 eAED:0.18 QI:280|1|1|1|0.90|0.83|12|349|765; (*GB) gi|8439545|gb|AAF74983.1|AF082893_1 (e_value=0.0) methionine synthase [Solanum tuberosum];; (*SWP) sp|Q42699|M
NbS00022063g0009.1 **Mass:** 86878 **Score:** 31 **Matches:** 1(1) **Sequences:** 1(1)
NbS00022063g0009.1 protein AED:0.10 eAED:0.10 QI:183|1|0.90|1|1|1|11|285|785; (*GB) gi|115361539|gb|ABI95860.1| (e_value=0.0) methionine synthase [Nicotiana suaveolens];; (*SWP) sp|Q42699|METE_CATF
NbS00044592g0017.1 **Mass:** 88066 **Score:** 31 **Matches:** 1(1) **Sequences:** 1(1)
NbS00044592g0017.1 protein AED:0.22 eAED:0.22 QI:367|0.90|0.83|1|0.81|0.83|12|0|795; (*GB) gi|8439545|gb|AAF74983.1|AF082893_1 (e_value=0.0) methionine synthase [Solanum tuberosum];; (*SWP) sp|Q42

---

161. NbS00003717g0028.1 **Mass:** 27691 **Score:** 31 **Matches:** 1(0) **Sequences:** 1(0) **emPAI:** 0.12
NbS00003717g0028.1 protein AED:0.30 eAED:0.30 QI:203|0.71|0.5|1|0.71|0.87|8|0|244; (*GB) gi|225438529|ref|XP_002279389.1| (e_value=6e-119) PREDICTED: probable ATP synthase 24 kDa subunit, mitochond

Query	Observed	Mr(expt)	Mr(calc)	ppm	Miss	Score	Expect	Rank	Unique	Peptide
17939	859.9278	1717.8410	1717.8410	0.01	0	31	0.054	1	U	K.ITIDPEDPAAVSEYAK.V

Proteins matching the same set of peptides:
NbS00036261g0008.1 **Mass:** 9199 **Score:** 31 **Matches:** 1(0) **Sequences:** 1(0)
NbS00036261g0008.1 protein ; (*GB) gi|255637866|gb|ACU19252.1| (e_value=3e-16) unknown [Glycine max];; (*TAIR) AT2G21870.2 (e_value=2e-14) | Symbols: MGP1 | copper ion binding;cobalt ion binding;z:

---

162. NbS00010860g0014.1 **Mass:** 9991 **Score:** 31 **Matches:** 1(0) **Sequences:** 1(0) **emPAI:** 0.35
NbS00010860g0014.1 protein AED:0.05 eAED:0.05 QI:88|1|1|1|1|1|5|242|88; (*GB) gi|225468340|ref|XP_002272246.1| (e_value=5e-54) PREDICTED: probable small nuclear ribonucleoprotein F [Vitis vinifera]

Query	Observed	Mr(expt)	Mr(calc)	ppm	Miss	Score	Expect	Rank	Unique	Peptide
12544	526.2709	1050.5272	1050.5280	-0.79	0	31	0.062	1	U	R.CNNVLYLR.G

Proteins matching the same set of peptides:
NbS00029443g0003.1 **Mass:** 9991 **Score:** 31 **Matches:** 1(0) **Sequences:** 1(0)
NbS00029443g0003.1 protein AED:0.25 eAED:0.25 QI:64|1|1|1|1|1|5|228|88; (*GB) gi|225468340|ref|XP_002272246.1| (e_value=5e-54) PREDICTED: probable small nuclear ribonucleoprotein F [Vitis vinifera]

---

163. NbS00022787g0008.1 **Mass:** 30166 **Score:** 31 **Matches:** 1(1) **Sequences:** 1(1) **emPAI:** 0.11
NbS00022787g0008.1 protein AED:0.33 eAED:0.33 QI:0|1|0.85|1|1|1|7|0|270; (*GB) gi|21912927|emb|CAC84143.2| (e_value=0.0) thioredoxin peroxidase [Nicotiana tabacum];; (*SWP) sp|Q9C5R8|BAS1B_ARATH (e

Query	Observed	Mr(expt)	Mr(calc)	ppm	Miss	Score	Expect	Rank	Unique	Peptide
16443	779.9329	1557.8513	1557.8515	-0.11	0	31	0.043	1	U	K.SYNVLIPDQGIALR.G

Proteins matching the same set of peptides:
NbS00026425g0011.1 **Mass:** 29723 **Score:** 31 **Matches:** 1(1) **Sequences:** 1(1)
NbS00026425g0011.1 protein AED:0.06 eAED:0.06 QI:0|1|0.85|1|1|1|7|0|268; (*GB) gi|21912927|emb|CAC84143.2| (e_value=5e-147) thioredoxin peroxidase [Nicotiana tabacum];; (*SWP) sp|Q9C5R8|BAS1B_ARATH
NbS00044024g0004.1 **Mass:** 38370 **Score:** 31 **Matches:** 1(1) **Sequences:** 1(1)
NbS00044024g0004.1 protein AED:0.01 eAED:0.01 QI:0|1|1|1|1|1|7|357|341; (*GB) gi|21912927|emb|CAC84143.2| (e_value=1e-144) thioredoxin peroxidase [Nicotiana tabacum];; (*SWP) sp|Q6ER94|BAS1_ORYSJ
NbS00050305g0001.1 **Mass:** 19523 **Score:** 31 **Matches:** 1(1) **Sequences:** 1(1)
NbS00050305g0001.1 protein AED:0.46 eAED:0.46 QI:72|0.8|0.5|0.83|1|1|6|0|173; (*GB) gi|21912927|emb|CAC84143.2| (e_value=4e-123) thioredoxin peroxidase [Nicotiana tabacum];; (*SWP) sp|Q6ER94|BAS1_(

---

164. NbS00013023g0001.1 **Mass:** 44550 **Score:** 31 **Matches:** 1(0) **Sequences:** 1(0) **emPAI:** 0.07
NbS00013023g0001.1 protein AED:0.21 eAED:0.21 QI:0|1|0.66|1|1|1|3|0|406; (*GB) gi|1707878|sp|P54260.1|GCST_SOLTU (e_value=0.0) RecName: Full=Aminomethyltransferase, mitochondrial; AltName: Full=Gly

Query	Observed	Mr(expt)	Mr(calc)	ppm	Miss	Score	Expect	Rank	Unique	Peptide
15669	733.3777	1464.7409	1464.7395	0.98	0	31	0.059	1	U	R.VLDINGASCFLTR.T

Proteins matching the same set of peptides:
NbS00039057g0010.1 **Mass:** 44608 **Score:** 31 **Matches:** 1(0) **Sequences:** 1(0)
NbS00039057g0010.1 protein AED:0.31 eAED:0.31 QI:0|1|0.66|1|1|1|3|0|406; (*GB) gi|1707878|sp|P54260.1|GCST_SOLTU (e_value=0.0) RecName: Full=Aminomethyltransferase, mitochondrial; AltName: Full=Gly

---

165. NbS00007101g0012.1 **Mass:** 111562 **Score:** 30 **Matches:** 1(0) **Sequences:** 1(0) **emPAI:** 0.03
NbS00007101g0012.1 protein AED:0.11 eAED:0.11 QI:0|0.85|0.8|1|1|1|15|492|1014; (*GB) gi|3334200|sp|O49954.1|GCSP_SOLTU (e_value=0.0) RecName: Full=Glycine dehydrogenase [decarboxylating], mitochonc

Query	Observed	Mr(expt)	Mr(calc)	ppm	Miss	Score	Expect	Rank	Unique	Peptide
9631	611.3400	1220.6654	1220.6587	5.55	0	30	0.12	1	U	K.IAILNANYMAK.R

Proteins matching the same set of peptides:
NbS00022684g0008.1 **Mass:** 129101 **Score:** 30 **Matches:** 1(0) **Sequences:** 1(0)
NbS00022684g0008.1 protein AED:0.15 eAED:0.15 QI:0|0.77|0.78|1|0.94|0.89|19|372|1168; (*GB) gi|3334200|sp|O49954.1|GCSP_SOLTU (e_value=0.0) RecName: Full=Glycine dehydrogenase [decarboxylating], m:

---

166. NbS00000710g0004.1 **Mass:** 54003 **Score:** 30 **Matches:** 1(0) **Sequences:** 1(0) **emPAI:** 0.06
NbS00000710g0004.1 protein AED:0.10 eAED:0.12 QI:195|0.77|0.7|1|0.77|0.5|10|0|501; (*SWP) sp|O94260|G3BP_SCHPO (e_value=3e-21) Putative G3BP-like protein OS=Schizosaccharomyces pombe (strain 972 /

Query	Observed	Mr(expt)	Mr(calc)	ppm	Miss	Score	Expect	Rank	Unique	Peptide
14090	631.3070	1260.5995	1260.5961	2.64	0	30	0.063	1	U	R.GYFVLNDMFR.Y

Proteins matching the same set of peptides:
NbS00002060g0018.1 **Mass:** 51753 **Score:** 30 **Matches:** 1(0) **Sequences:** 1(0)
NbS00002060g0018.1 protein AED:0.26 eAED:0.28 QI:356|0.63|0.41|1|0.81|0.75|12|0|480; (*GB) gi|359495838|ref|XP_002273770.2| (e_value=8e-98) PREDICTED: uncharacterized protein LOC100264206 [Vitis v:
NbS00012886g0004.1 **Mass:** 43582 **Score:** 30 **Matches:** 1(0) **Sequences:** 1(0)
NbS00012886g0004.1 protein AED:0.26 eAED:0.28 QI:196|0.66|0.8|1|0.44|0.4|10|0|401; (*GB) gi|359495838|ref|XP_002273770.2| (e_value=2e-74) PREDICTED: uncharacterized protein LOC100264206 [Vitis vin:
NbS00014019g0029.1 **Mass:** 48469 **Score:** 30 **Matches:** 1(0) **Sequences:** 1(0)
NbS00014019g0029.1 protein AED:0.29 eAED:0.29 QI:172|0.66|0.6|0.9|0.55|0.4|10|0|445; (*GB) gi|147842983|emb|CAN80553.1| (e_value=4e-88) hypothetical protein VITISV_024360 [Vitis vinifera];; (*SWP)

---

167. NbS00004213g0009.1 **Mass:** 32294 **Score:** 30 **Matches:** 1(1) **Sequences:** 1(1) **emPAI:** 0.10
NbS00004213g0009.1 protein AED:0.11 eAED:0.11 QI:0|1|0.83|1|1|0.83|6|0|298; (*GB) gi|19913109|emb|CAC84547.1| (e_value=0.0) dicarboxylate/tricarboxylate carrier [Nicotiana tabacum];; (*SWP) sp|Q54I

Query	Observed	Mr(expt)	Mr(calc)	ppm	Miss	Score	Expect	Rank	Unique	Peptide
14927	679.3784	1356.7422	1356.7361	4.45	0	30	0.054	1	U	R.IQLGQGSAVEVTR.T

---

168. NbS00028915g0014.1 **Mass:** 19116 **Score:** 30 **Matches:** 1(0) **Sequences:** 1(0) **emPAI:** 0.18
NbS00028915g0014.1 protein AED:0.24 eAED:0.24 QI:142|0|0.33|1|0|0.33|3|0|178; (*SWP) sp|Q9SUI4|PSAL_ARATH (e_value=7e-75) Photosystem I reaction center subunit XI, chloroplastic OS=Arabidopsis thal

Query	Observed	Mr(expt)	Mr(calc)	ppm	Miss	Score	Expect	Rank	Unique	Peptide
15637	730.3737	1458.7329	1458.7314	1.00	0	30	0.068	1	U	K.EGDASTAPALTLTGR.K

Mascot: http://www.matrixscience.com/
