# Supplementary material for: Alternative splicing regulation in plants by SP7-like effectors from symbiotic arbuscular mycorrhizal fungi
Source: Nat Commun. 2024 Aug 19;15:7107. doi: 10.1038/s41467-024-51512-5 (PMC11333574; doi:10.1038/s41467-024-51512-5)
Supplement: Supplementary file 10 — Source Data [file 41467_2024_51512_MOESM10_ESM.zip › Requena_8071-4_validation (eGFP).pdf]

Mascot Search Results

```
User            :
Email           :
Search title    : 8071-4
MS data file    : \\Server3\user\Kunden_Projekte\Requena_8071\8071\8071_4.mgf
Database        : Jessi 1 (4 sequences; 1794 residues)
Timestamp       : 1 Dec 2016 at 15:04:46 GMT
Significant hits: eGFP
```

Probability Based Mowse Score

Ions score is -10*Log(P), where P is the probability that the observed match is a random event.
Individual ions scores > 5 indicate identity or extensive homology (p<0.05).
Protein scores are derived from ions scores as a non-probabilistic basis for ranking protein hits.

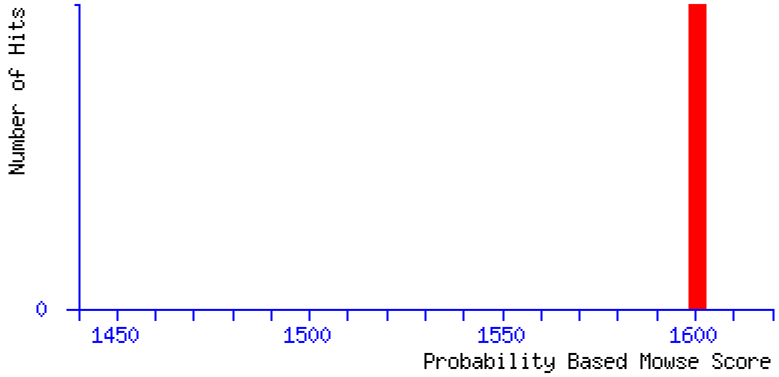

Peptide Summary Report

Format As | Peptide Summary | Help

Significance threshold p< 0.05 Max. number of hits AUTO

Standard scoring ○ MudPIT scoring ◉ Ions score cut-off 20 Show sub-sets ☐

Show pop-ups ◉ Suppress pop-ups ○ Sort unassigned Decreasing Score Require bold red ☐

Select All | Select None | Search Selected | ☐ **Error tolerant** | Archive Report

1. eGFP **Mass:** 27039 **Score:** 1600 **Queries matched:** 38

☐ Check to include this hit in error tolerant search or archive report

Query	Observed	Mr(expt)	Mr(calc)	Delta	Miss	Score	Expect	Rank	Peptide
☑ 12530	525.7641	1049.5136	1049.5141	-0.0005	0	62	2.9e-008	1	K.FEGDTLVNR.I
☑ 12531	525.7649	1049.5152	1049.5141	0.0011	0	(30)	5e-005	1	K.FEGDTLVNR.I
☑ 14132	633.7918	1265.5691	1265.5710	-0.0019	0	80	5.1e-010	1	K.SAMPEGYVQER.T
☑ 14133	633.7922	1265.5698	1265.5710	-0.0012	0	(35)	1.6e-005	1	K.SAMPEGYVQER.T
☑ 14134	633.7928	1265.5710	1265.5710	0.0000	0	(66)	1.3e-008	1	K.SAMPEGYVQER.T
☑ 14309	641.7906	1281.5666	1281.5659	0.0007	0	(44)	2.1e-006	1	K.SAMPEGYVQER.T + Oxidation (M)
☑ 14310	641.7906	1281.5666	1281.5659	0.0007	0	(30)	5.5e-005	1	K.SAMPEGYVQER.T + Oxidation (M)
☑ 14312	641.7906	1281.5667	1281.5659	0.0008	0	(46)	1.2e-006	1	K.SAMPEGYVQER.T + Oxidation (M)
☑ 14316	641.7908	1281.5670	1281.5659	0.0011	0	(36)	1.2e-005	1	K.SAMPEGYVQER.T + Oxidation (M)
☑ 14838	674.3324	1346.6502	1346.6506	-0.0004	1	(32)	3.4e-005	1	R.TIFFKDDGNYK.T
☑ 14845	674.3329	1346.6513	1346.6506	0.0007	1	(36)	1.2e-005	1	R.TIFFKDDGNYK.T
☑ 14847	674.3331	1346.6517	1346.6506	0.0011	1	43	2.4e-006	1	R.TIFFKDDGNYK.T
☑ 14848	674.3332	1346.6518	1346.6506	0.0012	1	(27)	0.00011	1	R.TIFFKDDGNYK.T
☑ 14851	674.3333	1346.6520	1346.6506	0.0013	1	(39)	6.1e-006	1	R.TIFFKDDGNYK.T
☑ 14853	674.3333	1346.6521	1346.6506	0.0015	1	(38)	7.4e-006	1	R.TIFFKDDGNYK.T
☑ 14855	674.3339	1346.6532	1346.6506	0.0026	1	(24)	0.00018	1	R.TIFFKDDGNYK.T
☑ 15764	739.3851	1476.7556	1476.7572	-0.0016	1	(28)	8.6e-005	1	R.AEVKFEGDTLVNR.I
☑ 15766	493.2596	1476.7569	1476.7572	-0.0003	1	(35)	1.5e-005	1	R.AEVKFEGDTLVNR.I
☑ 15768	739.3857	1476.7569	1476.7572	-0.0003	1	(26)	0.00011	1	R.AEVKFEGDTLVNR.I
☑ 15769	493.2596	1476.7571	1476.7572	-0.0001	1	(38)	8.9e-006	1	R.AEVKFEGDTLVNR.I
☑ 15773	739.3868	1476.7590	1476.7572	0.0018	1	74	2e-009	1	R.AEVKFEGDTLVNR.I
☑ 15934	752.3325	1502.6505	1502.6524	-0.0019	0	(29)	5.8e-005	1	K.FSVSGEGEGDATYGK.L
☑ 15935	752.3330	1502.6515	1502.6524	-0.0010	0	(50)	5.5e-007	1	K.FSVSGEGEGDATYGK.L
☑ 15936	752.3333	1502.6520	1502.6524	-0.0005	0	(23)	0.00027	1	K.FSVSGEGEGDATYGK.L

☑ 15937	752.3334	1502.6522	1502.6524	-0.0002	0	109	5.8e-013	1	K.FSVSGEGEGDATYGK.L
☑ 15939	752.3335	1502.6524	1502.6524	0.0000	0	(68)	8.9e-009	1	K.FSVSGEGEGDATYGK.L
☑ 15941	752.3338	1502.6530	1502.6524	0.0006	0	(59)	5.9e-008	1	K.FSVSGEGEGDATYGK.L
☑ 15942	752.3338	1502.6530	1502.6524	0.0006	0	(34)	2e-005	1	K.FSVSGEGEGDATYGK.L
☑ 15947	752.3343	1502.6540	1502.6524	0.0016	0	(42)	3.1e-006	1	K.FSVSGEGEGDATYGK.L
☑ 15948	752.3345	1502.6544	1502.6524	0.0020	0	(69)	5.6e-009	1	K.FSVSGEGEGDATYGK.L
☑ 15950	752.3346	1502.6546	1502.6524	0.0022	0	(21)	0.00036	1	K.FSVSGEGEGDATYGK.L
☑ 15951	752.3350	1502.6554	1502.6524	0.0029	0	(38)	7.4e-006	1	K.FSVSGEGEGDATYGK.L
☑ 16140	514.9353	1541.7841	1541.7837	0.0003	1	(34)	2.1e-005	1	K.GIDFKEDGNILGHK.L
☑ 16141	514.9353	1541.7841	1541.7837	0.0003	1	(23)	0.00027	1	K.GIDFKEDGNILGHK.L
☑ 16142	514.9355	1541.7846	1541.7837	0.0009	1	41	4.5e-006	1	K.GIDFKEDGNILGHK.L
☑ 16144	514.9357	1541.7852	1541.7837	0.0014	1	(36)	1.2e-005	1	K.GIDFKEDGNILGHK.L
☑ 19322	653.6622	1957.9647	1957.9632	0.0015	1	20	0.00049	1	K.FSVSGEGEGDATYGKLTLK.F
☑ 19553	658.6411	1972.9013	1972.8988	0.0025	0	35	1.7e-005	1	K.LEYNYNSHNVYIMADK.Q

Peptide matches not assigned to protein hits: (no details means no match)

Query	Observed	Mr(expt)	Mr(calc)	Delta	Miss	Score	Expect	Rank	Peptide
☑ 15949	752.3345	1502.6544	1502.6524	0.0020	0	20	0.00055	1	FSVSGEGEGDATYGK
☑ 14139	633.7949	1265.5752	1265.5710	0.0042	0	19	0.00066	1	SAMPEGYVQER
☑ 14850	674.3333	1346.6520	1346.6506	0.0013	1	17	0.00096	1	TIFFKDDGNYK
☑ 12017	491.7508	981.4870	981.4879	-0.0009	0	17	0.0011	1	EDGNILGHK
☑ 14314	641.7907	1281.5669	1281.5659	0.0010	0	16	0.0012	1	SAMPEGYVQER + Oxidation (M)
☑ 14315	641.7908	1281.5670	1281.5659	0.0011	0	16	0.0012	1	SAMPEGYVQER + Oxidation (M)
☑ 20220	744.0256	2229.0549	2229.0524	0.0025	1	16	0.0012	1	LEYNYNSHNVYIMADKQK
☑ 14306	641.7904	1281.5663	1281.5659	0.0004	0	16	0.0013	1	SAMPEGYVQER + Oxidation (M)
☑ 16084	511.9471	1532.8193	1532.8198	-0.0005	1	16	0.0013	1	FEGDTLVNRIELK
☑ 14135	633.7929	1265.5713	1265.5710	0.0003	0	16	0.0014	1	SAMPEGYVQER
☑ 14837	449.8907	1346.6502	1346.6506	-0.0004	1	16	0.0014	1	TIFFKDDGNYK
☑ 15938	752.3334	1502.6523	1502.6524	-0.0001	0	16	0.0014	1	FSVSGEGEGDATYGK
☑ 14305	641.7903	1281.5661	1281.5659	0.0002	0	15	0.0015	1	SAMPEGYVQER + Oxidation (M)
☑ 16083	511.9469	1532.8188	1532.8198	-0.0010	1	14	0.0019	1	FEGDTLVNRIELK

☑ 14839	449.8908	1346.6506	1346.6506	-0.0000	1	14	0.002	1	TIFFKDDGNYK
☑ 14302	641.7897	1281.5649	1281.5659	-0.0010	0	14	0.002	1	SAMPEGYVQER + Oxidation (M)
☑ 15943	752.3339	1502.6533	1502.6524	0.0009	0	14	0.002	1	FSVSGEGEGDATYGK
☑ 5010	407.2700	812.5254	812.4180	0.1074	1	14	0.002	1	NYGKGFK
☑ 36	301.1400	900.3982	900.4665	-0.0683	1	14	0.0021	1	RLSPDEGK
☑ 14137	633.7932	1265.5719	1265.5710	0.0009	0	14	0.0022	1	SAMPEGYVQER
☑ 15767	493.2596	1476.7569	1476.7572	-0.0003	1	13	0.0023	1	AEVKFEGDTLVNR
☑ 19325	653.6624	1957.9652	1957.9632	0.0020	1	13	0.0024	1	FSVSGEGEGDATYGKLTLK
☑ 15944	752.3340	1502.6535	1502.6524	0.0011	0	13	0.0024	1	FSVSGEGEGDATYGK
☑ 19549	658.6359	1972.8858	1972.8988	-0.0131	0	13	0.0026	1	LEYNYNSHNVYIMADK
☑ 9850	328.1940	654.3735	654.3741	-0.0006	0	13	0.0026	1	TIFFK
☑ 14304	641.7902	1281.5659	1281.5659	-0.0000	0	13	0.0027	1	SAMPEGYVQER + Oxidation (M)
☑ 16085	511.9475	1532.8208	1532.8198	0.0010	1	13	0.0028	1	FEGDTLVNRIELK
☑ 14311	641.7906	1281.5666	1281.5659	0.0007	0	13	0.0028	1	SAMPEGYVQER + Oxidation (M)
☑ 14849	674.3333	1346.6520	1346.6506	0.0013	1	12	0.0029	1	TIFFKDDGNYK
☑ 19326	653.6625	1957.9658	1957.9632	0.0026	1	12	0.0029	1	FSVSGEGEGDATYGKLTLK
☑ 15765	739.3855	1476.7564	1476.7572	-0.0007	1	12	0.003	1	AEVKFEGDTLVNR
☑ 16086	767.4202	1532.8258	1532.8198	0.0060	1	12	0.003	1	FEGDTLVNRIELK
☑ 14317	641.7908	1281.5670	1281.5659	0.0011	0	12	0.0031	1	SAMPEGYVQER + Oxidation (M)
☑ 14318	641.7908	1281.5670	1281.5659	0.0011	0	11	0.0038	1	SAMPEGYVQER + Oxidation (M)
☑ 16143	514.9356	1541.7850	1541.7837	0.0013	1	11	0.0042	1	GIDFKEDGNILGHK
☑ 19700	663.9726	1988.8960	1988.8938	0.0022	0	11	0.0044	1	LEYNYNSHNVYIMADK + Oxidation (M)
☑ 10956	413.7100	825.4054	825.4055	-0.0001	0	10	0.005	1	FICTTGK
☑ 14298	641.7878	1281.5610	1281.5659	-0.0049	0	10	0.005	1	SAMPEGYVQER + Oxidation (M)
☑ 14835	674.3323	1346.6500	1346.6506	-0.0006	1	9	0.006	1	TIFFKDDGNYK
☑ 20219	744.0247	2229.0521	2229.0524	-0.0002	1	9	0.0066	1	LEYNYNSHNVYIMADKQK
☑ 14854	674.3334	1346.6522	1346.6506	0.0016	1	9	0.007	1	TIFFKDDGNYK
☑ 19323	653.6622	1957.9649	1957.9632	0.0017	1	8	0.0076	1	FSVSGEGEGDATYGKLTLK
☑ 15771	493.2601	1476.7584	1476.7572	0.0012	1	8	0.0079	1	AEVKFEGDTLVNR
☑ 14308	641.7904	1281.5663	1281.5659	0.0004	0	6	0.012	1	SAMPEGYVQER + Oxidation (M)
☑ 19701	663.9736	1988.8991	1988.8938	0.0053	0	6	0.012	1	LEYNYNSHNVYIMADK + Oxidation (M)
☑ 10966	414.2180	826.4214	826.3895	0.0320	0	6	0.012	1	FGMVETK + Oxidation (M)

☑ 14320	641.7910	1281.5674	1281.5659	0.0015	0	6	0.012	1	SAMPEGYVQER + Oxidation (M)
☑ 19327	653.6627	1957.9661	1957.9632	0.0030	1	6	0.012	1	FSVSGEGEGDATYGKLTLK
☑ 15770	493.2598	1476.7575	1476.7572	0.0003	1	6	0.012	1	AEVKFEGDTLVNR
☑ 7918	449.8900	1346.6482	1346.6506	-0.0024	1	6	0.013	1	TIFFKDDGNYK
☑ 14136	633.7932	1265.5719	1265.5710	0.0009	0	6	0.013	1	SAMPEGYVQER
☑ 15940	752.3338	1502.6530	1502.6524	0.0006	0	6	0.013	1	FSVSGEGEGDATYGK
☑ 14852	449.8913	1346.6520	1346.6506	0.0014	1	5	0.015	1	TIFFKDDGNYK
☑ 19324	653.6623	1957.9650	1957.9632	0.0019	1	5	0.016	1	FSVSGEGEGDATYGKLTLK
☑ 20544	813.0895	2436.2468	2436.2535	-0.0068	0	5	0.033	1	GEELFTGVVPILVELDGDVNGHK
☑ 14836	449.8906	1346.6500	1346.6506	-0.0006	1	5	0.017	1	TIFFKDDGNYK
☑ 16087	767.4203	1532.8261	1532.8198	0.0064	1	5	0.017	1	FEGDTLVNRIELK
☑ 3327	359.1700	1074.4882	1074.4506	0.0376	0	4	0.018	1	DSTPSYDYK
☑ 14323	641.7922	1281.5699	1281.5659	0.0040	0	4	0.019	1	SAMPEGYVQER + Oxidation (M)
☑ 10500	752.3400	1502.6654	1502.6524	0.0130	0	4	0.019	1	FSVSGEGEGDATYGK
☑ 14301	641.7897	1281.5649	1281.5659	-0.0010	0	4	0.02	1	SAMPEGYVQER + Oxidation (M)
☑ 4400	376.8500	1127.5282	1127.6298	-0.1016	1	4	0.021	1	NILAEGVERK
☑ 15952	752.3350	1502.6554	1502.6524	0.0029	0	4	0.021	1	FSVSGEGEGDATYGK
☑ 14295	641.7868	1281.5591	1281.5659	-0.0068	0	3	0.023	1	SAMPEGYVQER + Oxidation (M)
☑ 9889	663.9700	1988.8882	1988.8938	-0.0056	0	3	0.026	1	LEYNYNSHNVYIMADK + Oxidation (M)
☑ 14842	449.8910	1346.6511	1346.6506	0.0005	1	3	0.026	1	TIFFKDDGNYK
☑ 15774	493.2603	1476.7590	1476.7572	0.0018	1	3	0.027	1	AEVKFEGDTLVNR
☑ 15264	469.5814	1405.7224	1405.6976	0.0248	0	3	0.028	1	IETLFDPEEVSK
☑ 15945	752.3342	1502.6538	1502.6524	0.0013	0	3	0.028	1	FSVSGEGEGDATYGK
☑ 12023	328.1995	981.5767	981.4879	0.0888	0	3	0.028	1	EDGNILGHK
☑ 14303	641.7900	1281.5654	1281.5659	-0.0005	0	2	0.028	1	SAMPEGYVQER + Oxidation (M)
☑ 20221	744.0264	2229.0575	2229.0524	0.0051	1	2	0.029	1	LEYNYNSHNVYIMADKQK
☑ 16139	514.9350	1541.7832	1541.7837	-0.0006	1	2	0.03	1	GIDFKEDGNILGHK
☑ 14840	449.8909	1346.6508	1346.6506	0.0002	1	2	0.032	1	TIFFKDDGNYK
☑ 19552	658.6407	1972.9002	1972.8988	0.0014	0	2	0.032	1	LEYNYNSHNVYIMADK
☑ 14355	643.8265	1285.6385	1285.6410	-0.0025	1	2	0.032	1	FGMVETKLSMK + Oxidation (M)
☑ 148	301.1400	900.3982	900.4665	-0.0683	1	2	0.032	1	RLSPDEGK
☑ 14623	659.8558	1317.6970	1317.5725	0.1245	1	2	0.033	1	DSTPSYDYKDK

☑	14319	641.7908	1281.5671	1281.5659	0.0012	0	2	0.034	1	SAMPEGYVQER + Oxidation (M)
☑	19554	987.4600	1972.9054	1972.8988	0.0065	0	2	0.034	1	LEYNYNSHNVYIMADK
☑	14300	641.7891	1281.5637	1281.5659	-0.0022	0	1	0.036	1	SAMPEGYVQER + Oxidation (M)
☑	11917	323.5279	967.5618	967.4359	0.1259	1	1	0.036	1	DDGNYKTR
☑	7265	434.8800	1301.6182	1301.6359	-0.0177	1	1	0.037	1	FGMVETKLSMK + 2 Oxidation (M)
☑	4404	376.9000	1127.6782	1127.6298	0.0484	1	1	0.042	1	NILAEGVERK
☑	14297	641.7876	1281.5606	1281.5659	-0.0053	0	1	0.044	1	SAMPEGYVQER + Oxidation (M)
☑	14157	635.8303	1269.6460	1269.6461	-0.0001	1	0	0.045	1	FGMVETKLSMK
☑	14138	633.7933	1265.5720	1265.5710	0.0010	0	0	0.046	1	SAMPEGYVQER
☑	14299	641.7885	1281.5624	1281.5659	-0.0036	0	0	0.047	1	SAMPEGYVQER + Oxidation (M)
☑	15954	752.3356	1502.6567	1502.6524	0.0043	0	0	0.048	1	FSVSGEGEGDATYGK
☑	20	301.1400	900.3982	900.4665	-0.0683	1	0	0.049	1	RLSPDEGK
☑	1	300.0100	299.0027							
☑	2	300.0100	299.0027							
☑	3	300.0200	299.0127							
☑	4	300.1800	299.1727							
☑	5	300.2000	299.1927							
☑	6	300.2000	299.1927							
☑	7	300.2000	299.1927							
☑	8	300.2000	299.1927							
☑	9	300.2000	299.1927							
☑	10	300.2100	299.2027							
☑	11	300.2900	299.2827							
☑	12	301.0300	300.0227							
☑	13	301.0300	300.0227							
☑	14	301.0300	300.0227							
☑	15	301.0300	300.0227							
☑	16	301.0300	300.0227							
☑	17	301.1400	300.1327							
☑	18	301.1400	300.1327							
☑	19	301.1400	300.1327							
☑	21	301.1400	300.1327							

☑	22	301.1400	300.1327
☑	23	301.1400	300.1327
☑	24	301.1400	300.1327
☑	25	301.1400	300.1327
☑	26	301.1400	300.1327
☑	27	301.1400	300.1327
☑	28	301.1400	300.1327
☑	29	301.1400	300.1327
☑	30	301.1400	300.1327
☑	31	301.1400	300.1327
☑	32	301.1400	300.1327
☑	33	301.1400	300.1327
☑	34	301.1400	300.1327
☑	35	301.1400	300.1327
☑	37	301.1400	300.1327
☑	38	301.1400	300.1327
☑	39	301.1400	300.1327
☑	40	301.1400	300.1327
☑	41	301.1400	300.1327
☑	42	301.1400	300.1327
☑	43	301.1400	300.1327
☑	44	301.1400	300.1327
☑	45	301.1400	300.1327
☑	46	301.1400	300.1327
☑	47	301.1400	300.1327
☑	48	301.1400	300.1327
☑	49	301.1400	300.1327
☑	50	301.1400	300.1327
☑	51	301.1400	300.1327
☑	52	301.1400	300.1327
☑	53	301.1400	300.1327
☑	54	301.1400	300.1327

☑	55	*301.1400*	*300.1327*
☑	56	*301.1400*	*300.1327*
☑	57	*301.1400*	*300.1327*
☑	58	*301.1400*	*300.1327*
☑	59	*301.1400*	*300.1327*
☑	60	*301.1400*	*300.1327*
☑	61	*301.1400*	*300.1327*
☑	62	*301.1400*	*300.1327*
☑	63	*301.1400*	*300.1327*
☑	64	*301.1400*	*300.1327*
☑	65	*301.1400*	*300.1327*
☑	66	*301.1400*	*300.1327*
☑	67	*301.1400*	*300.1327*
☑	68	*301.1400*	*300.1327*
☑	69	*301.1400*	*300.1327*
☑	70	*301.1400*	*300.1327*
☑	71	*301.1400*	*300.1327*
☑	72	*301.1400*	*300.1327*
☑	73	*301.1400*	*300.1327*
☑	74	*301.1400*	*300.1327*
☑	75	*301.1400*	*300.1327*
☑	76	*301.1400*	*300.1327*
☑	77	*301.1400*	*300.1327*
☑	78	*301.1400*	*300.1327*
☑	79	*301.1400*	*300.1327*
☑	80	*301.1400*	*300.1327*
☑	81	*301.1400*	*300.1327*
☑	82	*301.1400*	*300.1327*
☑	83	*301.1400*	*300.1327*
☑	84	*301.1400*	*300.1327*
☑	85	*301.1400*	*300.1327*
☑	86	*301.1400*	*300.1327*

☑	87	301.1400	300.1327
☑	88	301.1400	300.1327
☑	89	301.1400	300.1327
☑	90	301.1400	300.1327
☑	91	301.1400	300.1327
☑	92	301.1400	300.1327
☑	93	301.1400	300.1327
☑	94	301.1400	300.1327
☑	95	301.1400	300.1327
☑	96	301.1400	300.1327
☑	97	301.1400	300.1327
☑	98	301.1400	300.1327
☑	99	301.1400	300.1327
☑	100	301.1400	300.1327
☑	101	301.1400	300.1327
☑	102	301.1400	300.1327
☑	103	301.1400	300.1327
☑	104	301.1400	300.1327
☑	105	301.1400	300.1327
☑	106	301.1400	300.1327
☑	107	301.1400	300.1327
☑	108	301.1400	300.1327
☑	109	301.1400	300.1327
☑	110	301.1400	300.1327
☑	111	301.1400	300.1327
☑	112	301.1400	300.1327
☑	113	301.1400	300.1327
☑	114	301.1400	300.1327
☑	115	301.1400	300.1327
☑	116	301.1400	300.1327
☑	117	301.1400	300.1327
☑	118	301.1400	300.1327
