# Supplementary material for: Alternative splicing regulation in plants by SP7-like effectors from symbiotic arbuscular mycorrhizal fungi
Source: Nat Commun. 2024 Aug 19;15:7107. doi: 10.1038/s41467-024-51512-5 (PMC11333574; doi:10.1038/s41467-024-51512-5)

Source Data file.

Overview of nuclear status, project IDs and references of the analyzed *R. irregularis* genomes.

	Nuclear status	Assembly	Level	Chrs	BioSample	strain	Taxonomy	Reference
	HOMOKARYON	GCA_020716765.1	Chromosome	33	SAMN20669906	**A1**	Rhizophagus irregularis	Yildirir et al., 2021
	HOMOKARYON	GCA_020716745.1	Chromosome	33	SAMN20669907	**C2**	Rhizophagus irregularis	Yildirir et al., 2021
	HOMOKARYON	GCA_020716725.1	Chromosome	33	SAMN20669910	**DAOM-197198**	Rhizophagus irregularis	Yildirir et al., 2021
	HOMOKARYON	GCA_020716705.1	Chromosome	33	SAMN20669909	**4401**	Rhizophagus irregularis	Yildirir et al., 2021
	HOMOKARYON	GCA_020716685.1	Chromosome	33	SAMN20669908	**B3**	Rhizophagus irregularis	Yildirir et al., 2021
**REFERENCE strain**	HOMOKARYON	GCA_026210795.1	Chromosome	32	SAMN31081226	**DAOM-197198**	Rhizophagus irregularis	Manley et al., 2023
	HETEROKARYON	/	Chromosome	33	/	**A4**	Rhizophagus irregularis	Sperschneider et al., 2023
	HETEROKARYON	/	Chromosome	33	/	**A5**	Rhizophagus irregularis	Sperschneider et al., 2023
	HETEROKARYON	/	Chromosome	33	/	**G1**	Rhizophagus irregularis	Sperschneider et al., 2023
	HETEROKARYON	/	Chromosome	33	/	**SL1**	Rhizophagus irregularis	Sperschneider et al., 2023

Figure legend for Karyoplots:

Genomic location of SP-like genes in homo- and heterokaryotic strains of R. irregularis. The karyotype and location of SP-like effector genes of each assembly was plotted using the R package KaryoploteR. Effector coordinates were extracted from BLASTN-based sequence homology searches. Arrows indicate the location of effector genes. Red color in assemblies of heterokaryotic strains indicates variation between the two haplotypes.

Figure legend for Dotplots:

SP-like effectors show extensive gene loss in heterokaryotic strains. Whole genome alignments comparing nine publicly available genomes to the reference strain DAOM-197198 (Manley et al., 2023). Alignments were generated using nucmer and viualized using mummerplot and gnuplot. Arrows indicate the coordinates of SP7-like effector genes. Blue color indicates effector genes in the reference assembly, black indicates effectors in query assemblies. Red color indicates presence/absence variation between the reference and query assemblies.

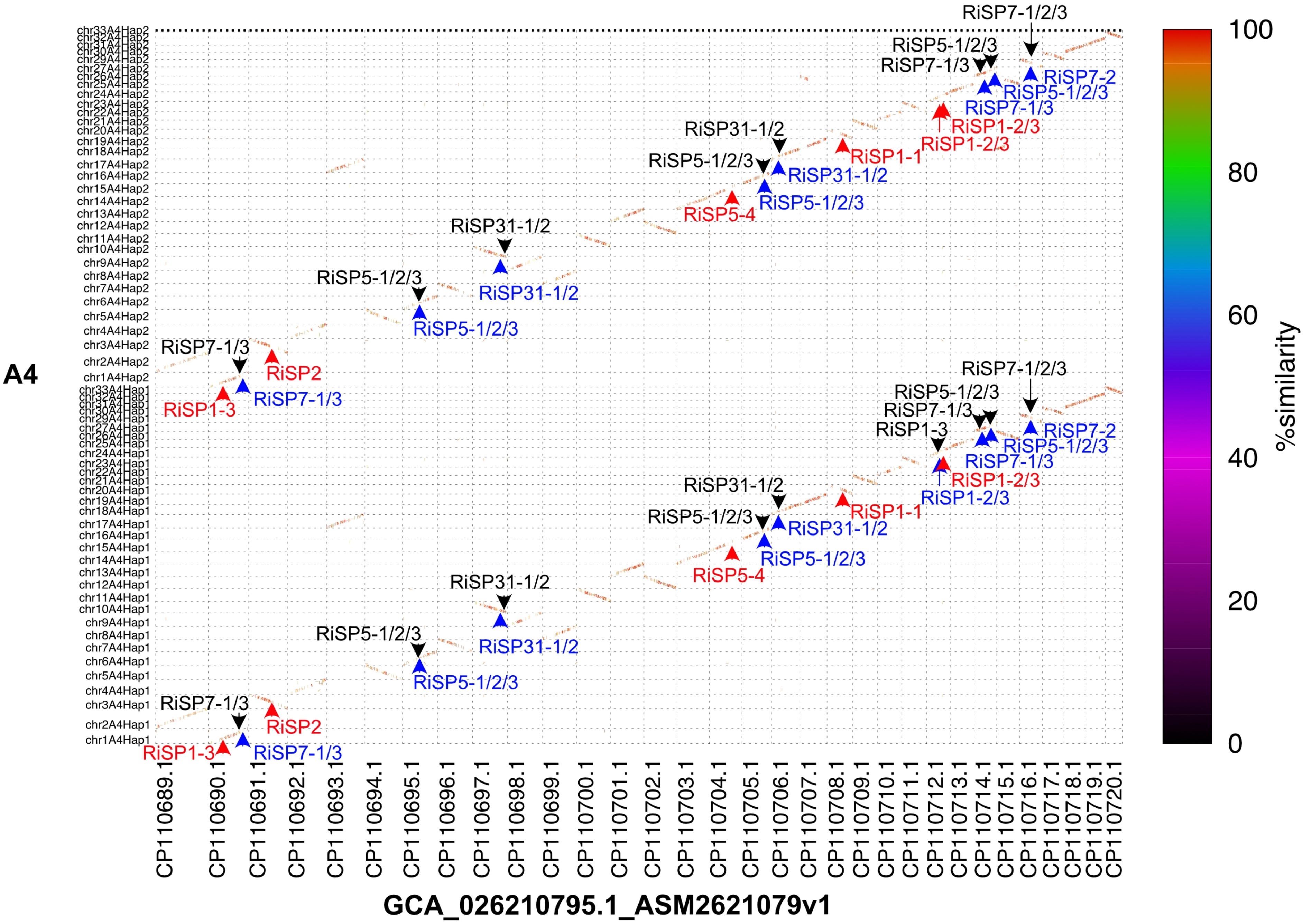
A4
chr33A4Hap2
chr32A4Hap2
chr31A4Hap2
chr30A4Hap2
chr29A4Hap2
chr27A4Hap2
chr26A4Hap2
chr25A4Hap2
chr24A4Hap2
chr23A4Hap2
chr22A4Hap2
chr21A4Hap2
chr20A4Hap2
chr19A4Hap2
chr18A4Hap2
chr17A4Hap2
chr16A4Hap2
chr15A4Hap2
chr14A4Hap2
chr13A4Hap2
chr12A4Hap2
chr11A4Hap2
chr10A4Hap2
chr9A4Hap2
chr8A4Hap2
chr7A4Hap2
chr6A4Hap2
chr5A4Hap2
chr4A4Hap2
chr3A4Hap2
chr2A4Hap2
chr1A4Hap2
chr33A4Hap1
chr32A4Hap1
chr31A4Hap1
chr30A4Hap1
chr29A4Hap1
chr27A4Hap1
chr26A4Hap1
chr25A4Hap1
chr24A4Hap1
chr23A4Hap1
chr22A4Hap1
chr21A4Hap1
chr20A4Hap1
chr19A4Hap1
chr18A4Hap1
chr17A4Hap1
chr16A4Hap1
chr15A4Hap1
chr14A4Hap1
chr13A4Hap1
chr12A4Hap1
chr11A4Hap1
chr10A4Hap1
chr9A4Hap1
chr8A4Hap1
chr7A4Hap1
chr6A4Hap1
chr5A4Hap1
chr4A4Hap1
chr3A4Hap1
chr2A4Hap1
chr1A4Hap1
RiSP7-1/2/3
RiSP5-1/2/3
RiSP7-1/3
RiSP7-2
RiSP5-1/2/3
RiSP7-1/3
RiSP1-2/3
RiSP1-2/3
RiSP31-1/2
RiSP1-1
RiSP5-1/2/3
RiSP31-1/2
RiSP5-1/2/3
RiSP5-4
RiSP31-1/2
RiSP31-1/2
RiSP5-1/2/3
RiSP5-1/2/3
RiSP7-1/3
RiSP2
RiSP7-1/3
RiSP1-3
RiSP7-1/2/3
RiSP5-1/2/3
RiSP7-1/3
RiSP1-3
RiSP7-2
RiSP5-1/2/3
RiSP7-1/3
RiSP1-2/3
RiSP1-2/3
RiSP31-1/2
RiSP1-1
RiSP5-1/2/3
RiSP31-1/2
RiSP5-1/2/3
RiSP5-4
RiSP31-1/2
RiSP31-1/2
RiSP5-1/2/3
RiSP5-1/2/3
RiSP7-1/3
RiSP2
RiSP1-3
RiSP7-1/3
CP110689.1
CP110690.1
CP110691.1
CP110692.1
CP110693.1
CP110694.1
CP110695.1
CP110696.1
CP110697.1
CP110698.1
CP110699.1
CP110700.1
CP110701.1
CP110702.1
CP110703.1
CP110704.1
CP110705.1
CP110706.1
CP110707.1
CP110708.1
CP110709.1
CP110710.1
CP110711.1
CP110712.1
CP110713.1
CP110714.1
CP110715.1
CP110716.1
CP110717.1
CP110718.1
CP110719.1
CP110720.1
GCA_026210795.1_ASM2621079v1
100
80
60
40
20
0
%similarity

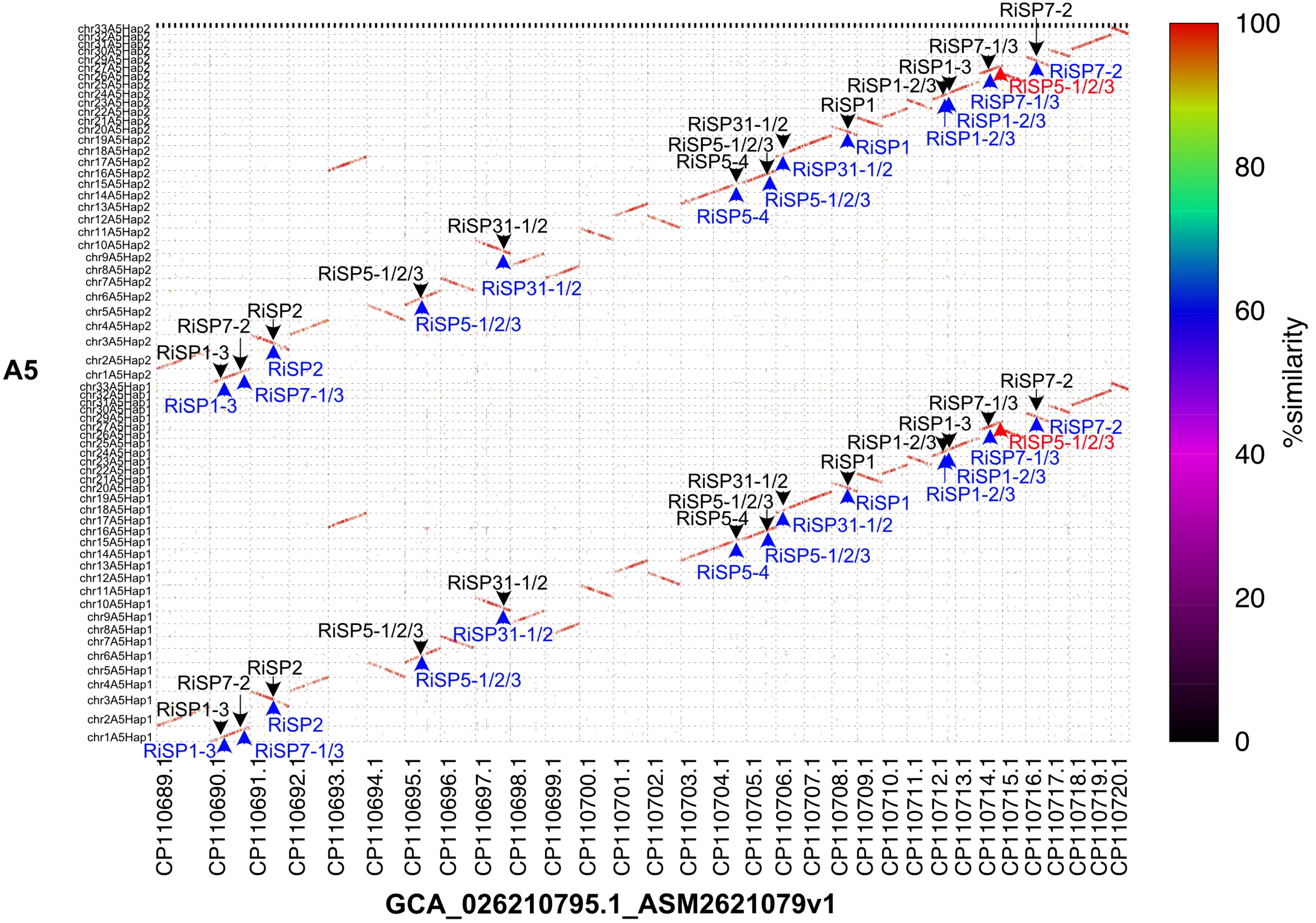
A5
GCA_026210795.1_ASM2621079v1
%similarity
100
80
60
40
20
0
RiSP7-2
RiSP7-1/3
RiSP1-3
RiSP1-2/3
RiSP1
RiSP31-1/2
RiSP5-1/2/3
RiSP5-4
RiSP2
RiSP5-1/2/3
RiSP1-2/3
chr1A5Hap1
chr33A5Hap2
CP110689.1
CP110720.1

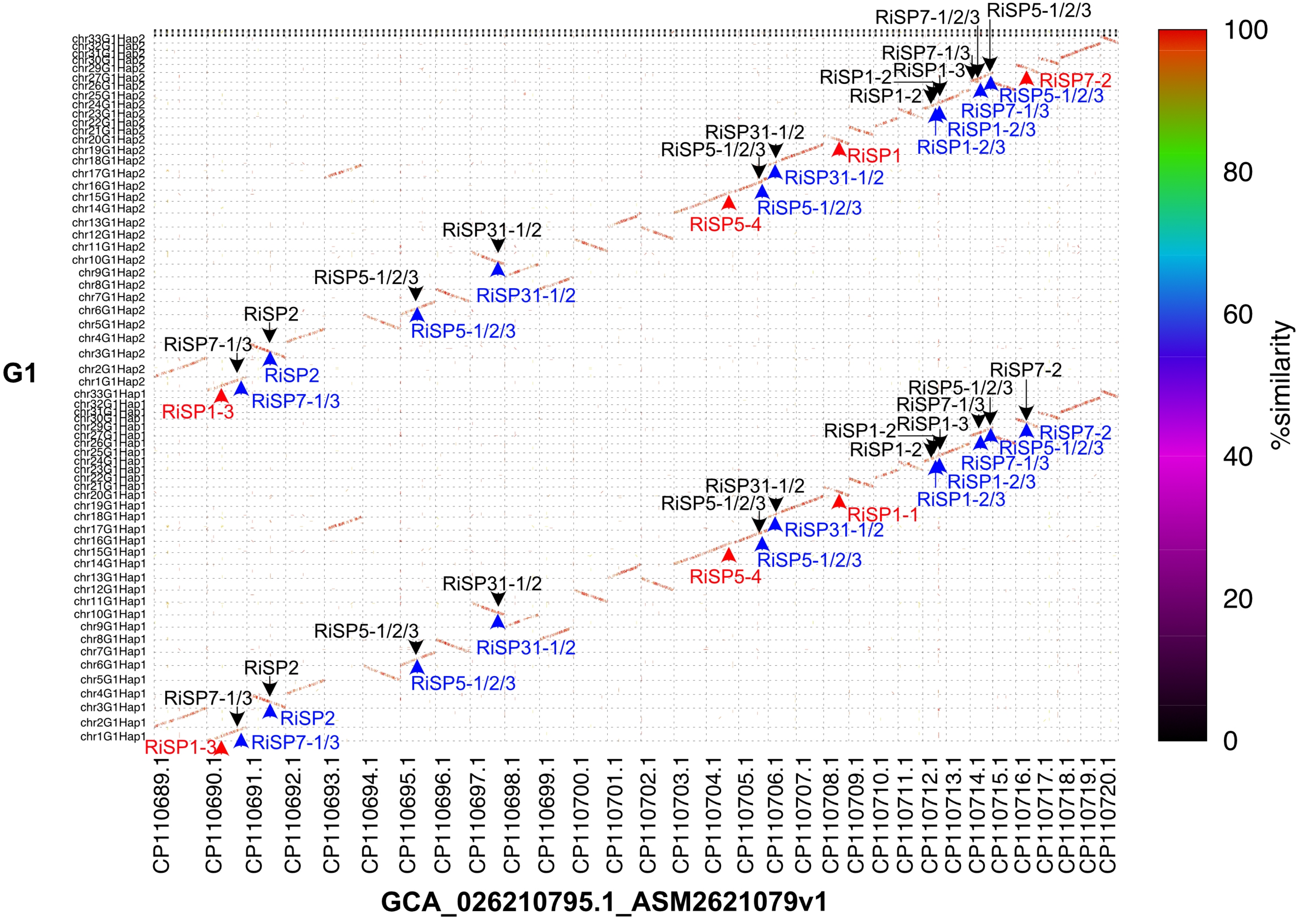

G1
GCA_026210795.1_ASM2621079v1
%similarity
100
80
60
40
20
0
chr33G1Hap2
chr18G1Hap2
chr17G1Hap2
chr16G1Hap2
chr15G1Hap2
chr14G1Hap2
chr13G1Hap2
chr12G1Hap2
chr11G1Hap2
chr10G1Hap2
chr9G1Hap2
chr8G1Hap2
chr7G1Hap2
chr6G1Hap2
chr5G1Hap2
chr4G1Hap2
chr3G1Hap2
chr2G1Hap2
chr1G1Hap2
chr33G1Hap1
chr18G1Hap1
chr17G1Hap1
chr16G1Hap1
chr15G1Hap1
chr14G1Hap1
chr13G1Hap1
chr12G1Hap1
chr11G1Hap1
chr10G1Hap1
chr9G1Hap1
chr8G1Hap1
chr7G1Hap1
chr6G1Hap1
chr5G1Hap1
chr4G1Hap1
chr3G1Hap1
chr2G1Hap1
chr1G1Hap1
CP110689.1
CP110690.1
CP110691.1
CP110692.1
CP110693.1
CP110694.1
CP110695.1
CP110696.1
CP110697.1
CP110698.1
CP110699.1
CP110700.1
CP110701.1
CP110702.1
CP110703.1
CP110704.1
CP110705.1
CP110706.1
CP110707.1
CP110708.1
CP110709.1
CP110710.1
CP110711.1
CP110712.1
CP110713.1
CP110714.1
CP110715.1
CP110716.1
CP110717.1
CP110718.1
CP110719.1
CP110720.1
RiSP7-1/2/3
RiSP5-1/2/3
RiSP7-1/3
RiSP1-3
RiSP1-2
RiSP1-2
RiSP7-2
RiSP5-1/2/3
RiSP7-1/3
RiSP1-2/3
RiSP1-2/3
RiSP31-1/2
RiSP5-1/2/3
RiSP1
RiSP31-1/2
RiSP5-1/2/3
RiSP5-4
RiSP31-1/2
RiSP31-1/2
RiSP5-1/2/3
RiSP5-1/2/3
RiSP2
RiSP7-1/3
RiSP2
RiSP7-1/3
RiSP1-3
RiSP7-2
RiSP5-1/2/3
RiSP7-1/3
RiSP1-2
RiSP1-3
RiSP1-2
RiSP7-2
RiSP5-1/2/3
RiSP7-1/3
RiSP1-2/3
RiSP1-2/3
RiSP31-1/2
RiSP5-1/2/3
RiSP1-1
RiSP31-1/2
RiSP5-1/2/3
RiSP5-4
RiSP31-1/2
RiSP31-1/2
RiSP5-1/2/3
RiSP5-1/2/3
RiSP2
RiSP7-1/3
RiSP2
RiSP1-3
RiSP7-1/3

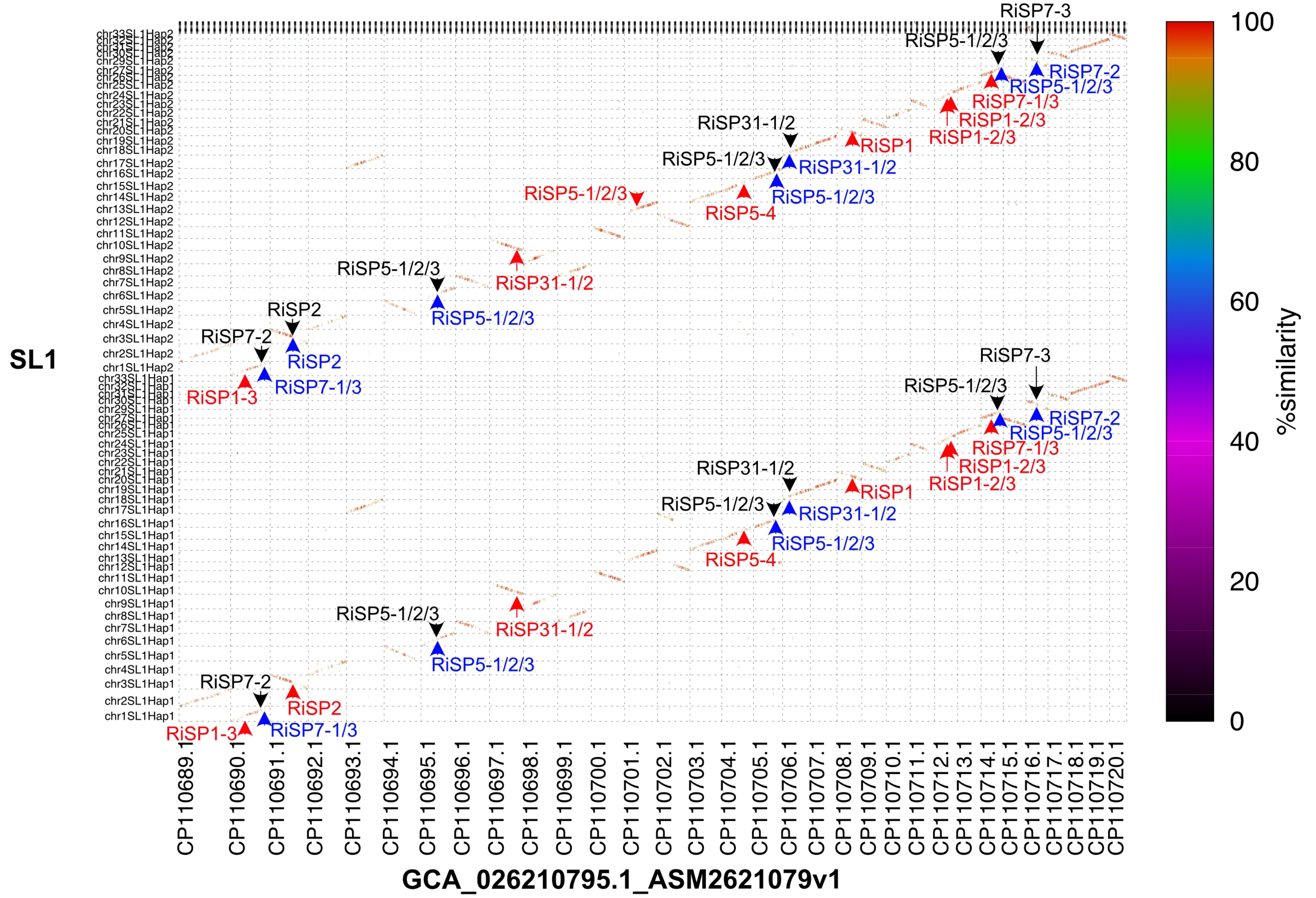
SL1
GCA_026210795.1_ASM2621079v1
%similarity
100
80
60
40
20
0
chr1SL1Hap2
chr2SL1Hap2
chr3SL1Hap2
chr4SL1Hap2
chr5SL1Hap2
chr6SL1Hap2
chr7SL1Hap2
chr8SL1Hap2
chr9SL1Hap2
chr10SL1Hap2
chr11SL1Hap2
chr12SL1Hap2
chr13SL1Hap2
chr14SL1Hap2
chr15SL1Hap2
chr16SL1Hap2
chr17SL1Hap2
chr18SL1Hap2
chr19SL1Hap2
chr1SL1Hap1
chr2SL1Hap1
chr3SL1Hap1
chr4SL1Hap1
chr5SL1Hap1
chr6SL1Hap1
chr7SL1Hap1
chr8SL1Hap1
chr9SL1Hap1
chr10SL1Hap1
chr11SL1Hap1
chr14SL1Hap1
chr15SL1Hap1
chr16SL1Hap1
chr17SL1Hap1
chr18SL1Hap1
chr19SL1Hap1
RiSP7-3
RiSP5-1/2/3
RiSP7-2
RiSP7-1/3
RiSP1-2/3
RiSP31-1/2
RiSP1
RiSP5-4
RiSP2
RiSP1-3
CP110689.1
CP110690.1
CP110691.1
CP110692.1
CP110693.1
CP110694.1
CP110695.1
CP110696.1
CP110697.1
CP110698.1
CP110699.1
CP110700.1
CP110701.1
CP110702.1
CP110703.1
CP110704.1
CP110705.1
CP110706.1
CP110707.1
CP110708.1
CP110709.1
CP110710.1
CP110711.1
CP110712.1
CP110713.1
CP110714.1
CP110715.1
CP110716.1
CP110717.1
CP110718.1
CP110719.1
CP110720.1

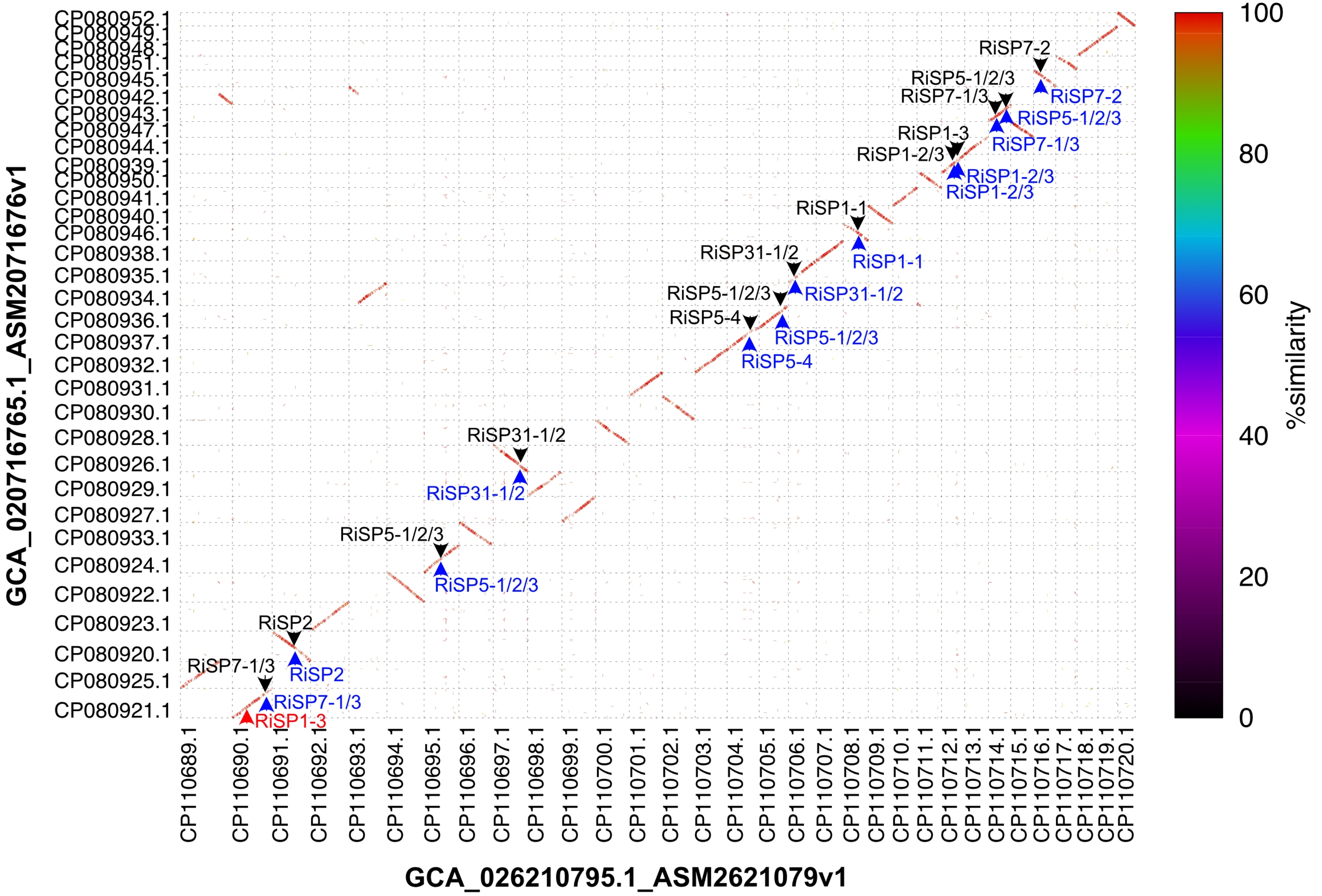
GCA_020716765.1_ASM2071676v1
CP080952.1
CP080949.1
CP080948.1
CP080951.1
CP080945.1
CP080942.1
CP080943.1
CP080947.1
CP080944.1
CP080939.1
CP080950.1
CP080941.1
CP080940.1
CP080946.1
CP080938.1
CP080935.1
CP080934.1
CP080936.1
CP080937.1
CP080932.1
CP080931.1
CP080930.1
CP080928.1
CP080926.1
CP080929.1
CP080927.1
CP080933.1
CP080924.1
CP080922.1
CP080923.1
CP080920.1
CP080925.1
CP080921.1
RiSP7-2
RiSP7-2
RiSP5-1/2/3
RiSP7-1/3
RiSP5-1/2/3
RiSP7-1/3
RiSP1-3
RiSP1-2/3
RiSP1-2/3
RiSP1-2/3
RiSP1-1
RiSP1-1
RiSP31-1/2
RiSP31-1/2
RiSP5-1/2/3
RiSP5-4
RiSP5-1/2/3
RiSP5-4
RiSP31-1/2
RiSP31-1/2
RiSP5-1/2/3
RiSP5-1/2/3
RiSP2
RiSP2
RiSP7-1/3
RiSP7-1/3
RiSP1-3
CP110689.1
CP110690.1
CP110691.1
CP110692.1
CP110693.1
CP110694.1
CP110695.1
CP110696.1
CP110697.1
CP110698.1
CP110699.1
CP110700.1
CP110701.1
CP110702.1
CP110703.1
CP110704.1
CP110705.1
CP110706.1
CP110707.1
CP110708.1
CP110709.1
CP110710.1
CP110711.1
CP110712.1
CP110713.1
CP110714.1
CP110715.1
CP110716.1
CP110717.1
CP110718.1
CP110719.1
CP110720.1
GCA_026210795.1_ASM2621079v1
100
80
60
40
20
0
%similarity

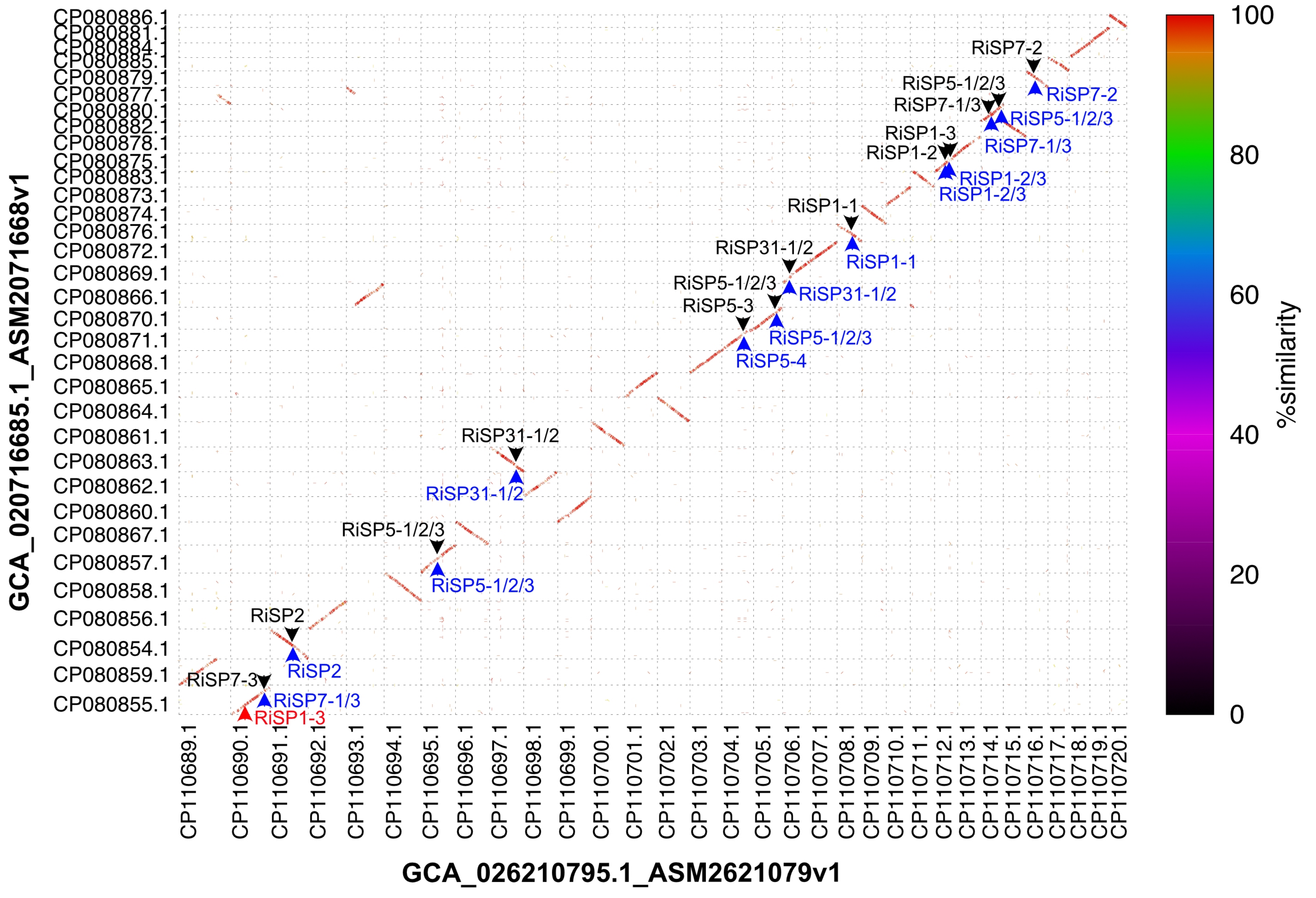

GCA_020716685.1_ASM2071668v1
GCA_026210795.1_ASM2621079v1
%similarity
100
80
60
40
20
0
CP080886.1
CP080881.1
CP080884.1
CP080885.1
CP080879.1
CP080877.1
CP080880.1
CP080882.1
CP080878.1
CP080875.1
CP080883.1
CP080873.1
CP080874.1
CP080876.1
CP080872.1
CP080869.1
CP080866.1
CP080870.1
CP080871.1
CP080868.1
CP080865.1
CP080864.1
CP080861.1
CP080863.1
CP080862.1
CP080860.1
CP080867.1
CP080857.1
CP080858.1
CP080856.1
CP080854.1
CP080859.1
CP080855.1
CP110689.1
CP110690.1
CP110691.1
CP110692.1
CP110693.1
CP110694.1
CP110695.1
CP110696.1
CP110697.1
CP110698.1
CP110699.1
CP110700.1
CP110701.1
CP110702.1
CP110703.1
CP110704.1
CP110705.1
CP110706.1
CP110707.1
CP110708.1
CP110709.1
CP110710.1
CP110711.1
CP110712.1
CP110713.1
CP110714.1
CP110715.1
CP110716.1
CP110717.1
CP110718.1
CP110719.1
CP110720.1
RiSP7-2
RiSP7-2
RiSP5-1/2/3
RiSP7-1/3
RiSP5-1/2/3
RiSP7-1/3
RiSP1-3
RiSP1-2
RiSP1-2/3
RiSP1-2/3
RiSP1-1
RiSP1-1
RiSP31-1/2
RiSP31-1/2
RiSP5-1/2/3
RiSP5-3
RiSP5-1/2/3
RiSP5-4
RiSP31-1/2
RiSP31-1/2
RiSP5-1/2/3
RiSP5-1/2/3
RiSP2
RiSP2
RiSP7-3
RiSP7-1/3
RiSP1-3

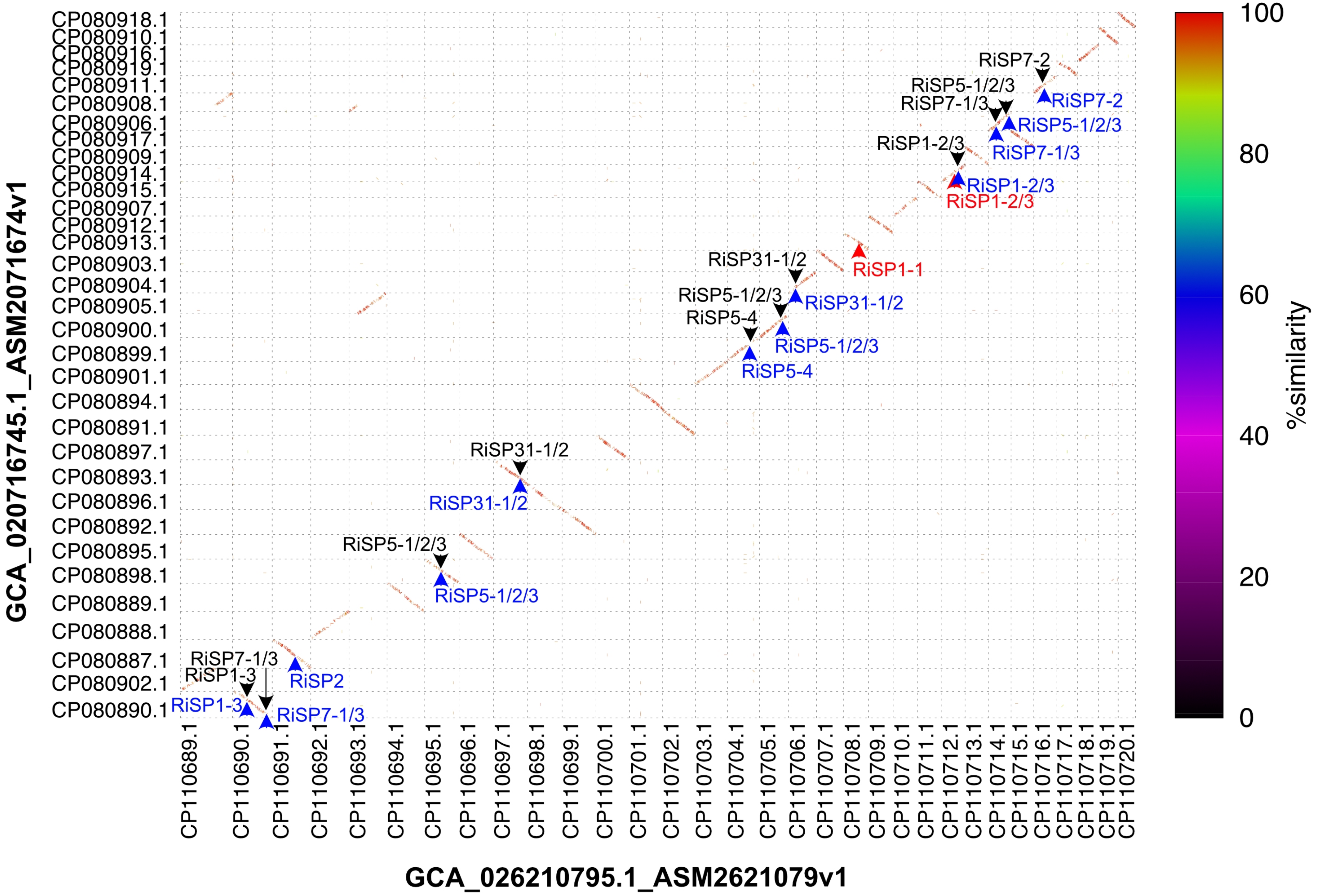
GCA_020716745.1_ASM2071674v1
GCA_026210795.1_ASM2621079v1
%similarity
100
80
60
40
20
0
CP080918.1
CP080910.1
CP080916.1
CP080919.1
CP080911.1
CP080908.1
CP080906.1
CP080917.1
CP080909.1
CP080914.1
CP080915.1
CP080907.1
CP080912.1
CP080913.1
CP080903.1
CP080904.1
CP080905.1
CP080900.1
CP080899.1
CP080901.1
CP080894.1
CP080891.1
CP080897.1
CP080893.1
CP080896.1
CP080892.1
CP080895.1
CP080898.1
CP080889.1
CP080888.1
CP080887.1
CP080902.1
CP080890.1
CP110689.1
CP110690.1
CP110691.1
CP110692.1
CP110693.1
CP110694.1
CP110695.1
CP110696.1
CP110697.1
CP110698.1
CP110699.1
CP110700.1
CP110701.1
CP110702.1
CP110703.1
CP110704.1
CP110705.1
CP110706.1
CP110707.1
CP110708.1
CP110709.1
CP110710.1
CP110711.1
CP110712.1
CP110713.1
CP110714.1
CP110715.1
CP110716.1
CP110717.1
CP110718.1
CP110719.1
CP110720.1
RiSP7-2
RiSP7-2
RiSP5-1/2/3
RiSP7-1/3
RiSP5-1/2/3
RiSP7-1/3
RiSP1-2/3
RiSP1-2/3
RiSP1-2/3
RiSP1-1
RiSP31-1/2
RiSP31-1/2
RiSP5-1/2/3
RiSP5-4
RiSP5-1/2/3
RiSP5-4
RiSP31-1/2
RiSP31-1/2
RiSP5-1/2/3
RiSP5-1/2/3
RiSP7-1/3
RiSP1-3
RiSP2
RiSP1-3
RiSP7-1/3

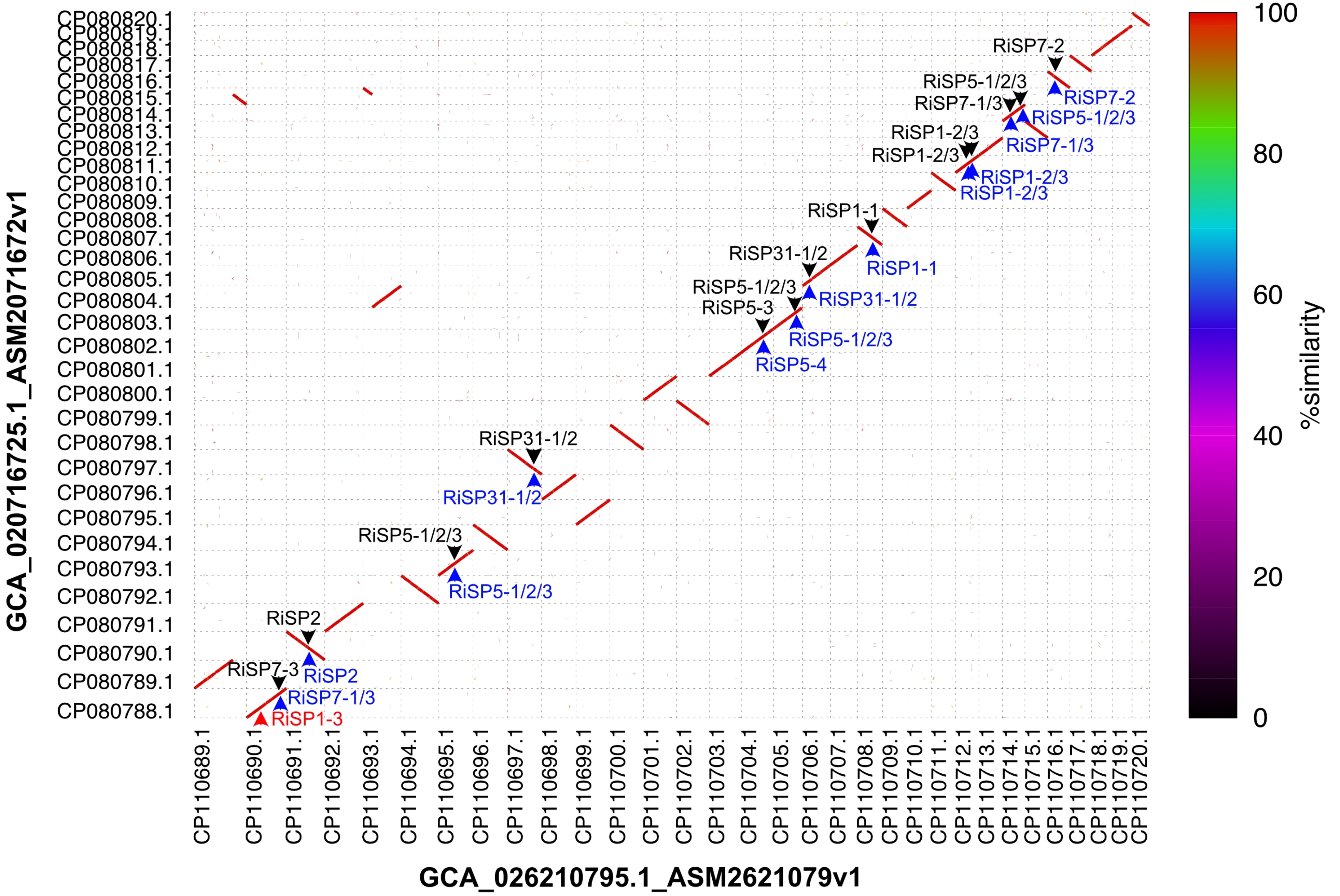

GCA_020716725.1_ASM2071672v1
GCA_026210795.1_ASM2621079v1
%similarity
100
80
60
40
20
0
RiSP7-2
RiSP5-1/2/3
RiSP7-1/3
RiSP1-2/3
RiSP1-1
RiSP31-1/2
RiSP5-3
RiSP5-4
RiSP2
RiSP7-3
RiSP1-3

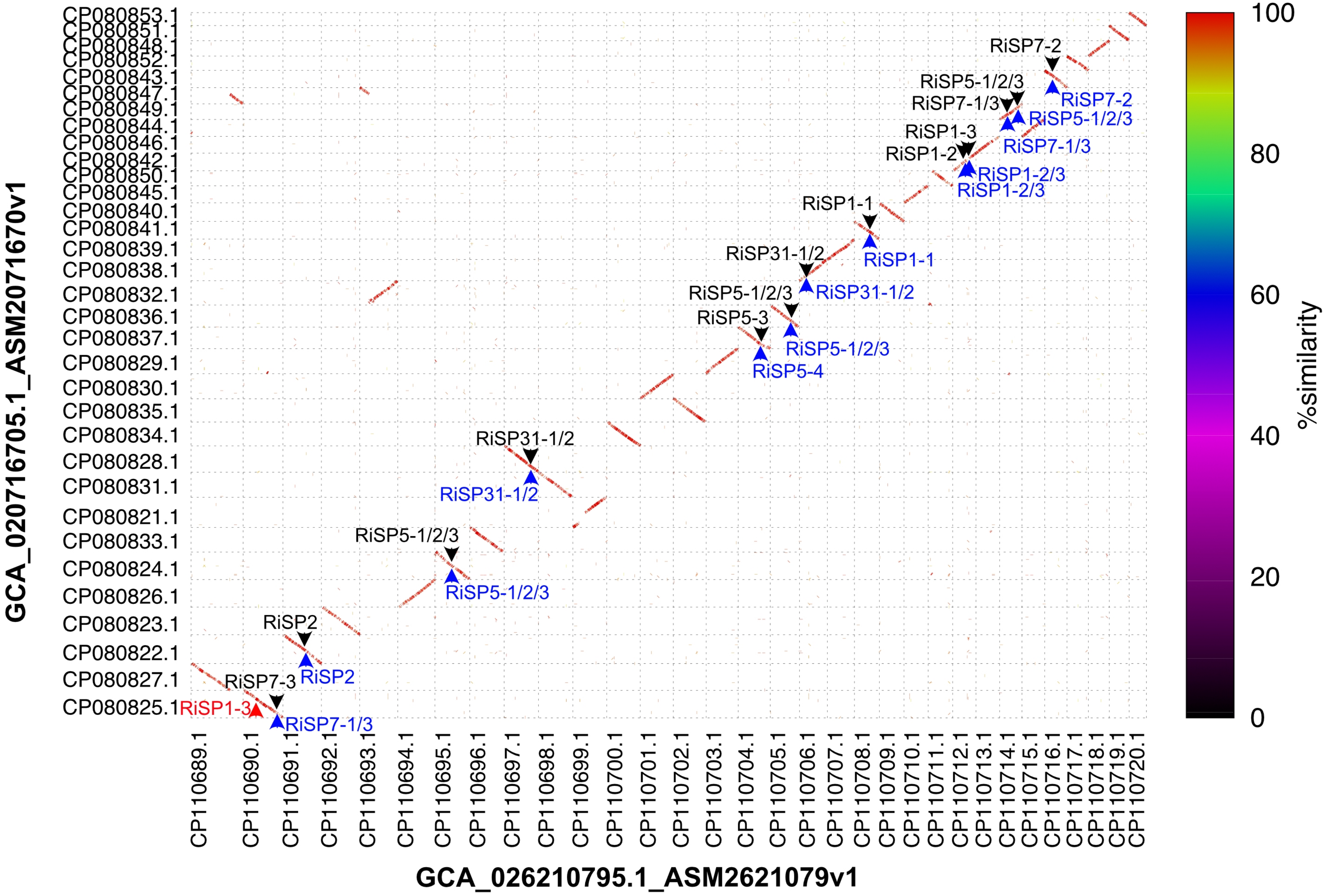
GCA_020716705.1_ASM2071670v1
GCA_026210795.1_ASM2621079v1
%similarity
100
80
60
40
20
0
CP080853.1
CP080851.1
CP080848.1
CP080852.1
CP080843.1
CP080847.1
CP080849.1
CP080844.1
CP080846.1
CP080842.1
CP080850.1
CP080845.1
CP080840.1
CP080841.1
CP080839.1
CP080838.1
CP080832.1
CP080836.1
CP080837.1
CP080829.1
CP080830.1
CP080835.1
CP080834.1
CP080828.1
CP080831.1
CP080821.1
CP080833.1
CP080824.1
CP080826.1
CP080823.1
CP080822.1
CP080827.1
CP080825.1
CP110689.1
CP110690.1
CP110691.1
CP110692.1
CP110693.1
CP110694.1
CP110695.1
CP110696.1
CP110697.1
CP110698.1
CP110699.1
CP110700.1
CP110701.1
CP110702.1
CP110703.1
CP110704.1
CP110705.1
CP110706.1
CP110707.1
CP110708.1
CP110709.1
CP110710.1
CP110711.1
CP110712.1
CP110713.1
CP110714.1
CP110715.1
CP110716.1
CP110717.1
CP110718.1
CP110719.1
CP110720.1
RiSP7-2
RiSP7-2
RiSP5-1/2/3
RiSP7-1/3
RiSP5-1/2/3
RiSP7-1/3
RiSP1-3
RiSP1-2
RiSP1-2/3
RiSP1-2/3
RiSP1-1
RiSP1-1
RiSP31-1/2
RiSP31-1/2
RiSP5-1/2/3
RiSP5-3
RiSP5-1/2/3
RiSP5-4
RiSP31-1/2
RiSP31-1/2
RiSP5-1/2/3
RiSP5-1/2/3
RiSP2
RiSP2
RiSP7-3
RiSP1-3
RiSP7-1/3

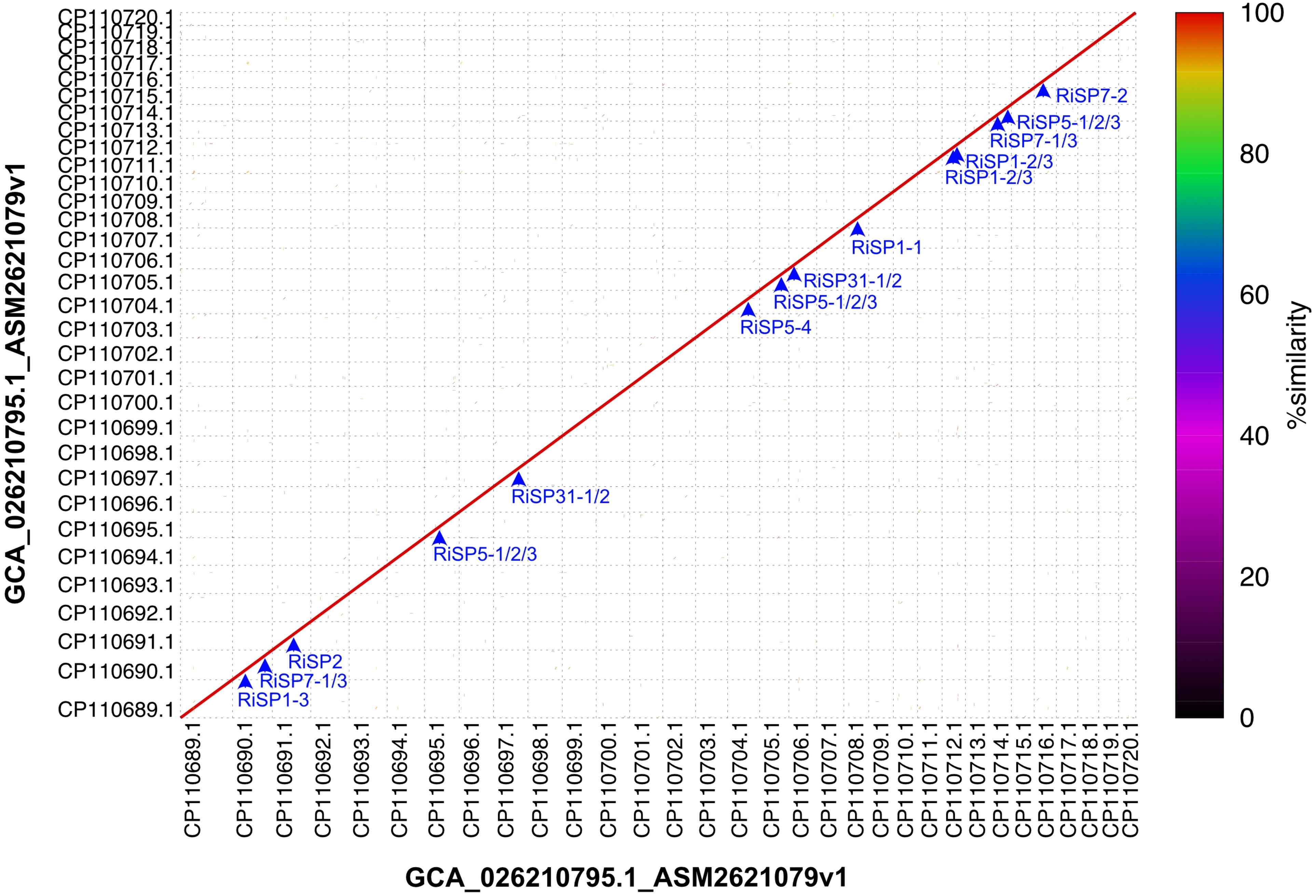
GCA_026210795.1_ASM2621079v1
GCA_026210795.1_ASM2621079v1
%similarity
100
80
60
40
20
0
CP110689.1
CP110690.1
CP110691.1
CP110692.1
CP110693.1
CP110694.1
CP110695.1
CP110696.1
CP110697.1
CP110698.1
CP110699.1
CP110700.1
CP110701.1
CP110702.1
CP110703.1
CP110704.1
CP110705.1
CP110706.1
CP110707.1
CP110708.1
CP110709.1
CP110710.1
CP110711.1
CP110712.1
CP110713.1
CP110714.1
CP110715.1
CP110716.1
CP110717.1
CP110718.1
CP110719.1
CP110720.1
RiSP1-3
RiSP7-1/3
RiSP2
RiSP5-1/2/3
RiSP31-1/2
RiSP5-4
RiSP5-1/2/3
RiSP31-1/2
RiSP1-1
RiSP1-2/3
RiSP1-2/3
RiSP7-1/3
RiSP5-1/2/3
RiSP7-2

A4

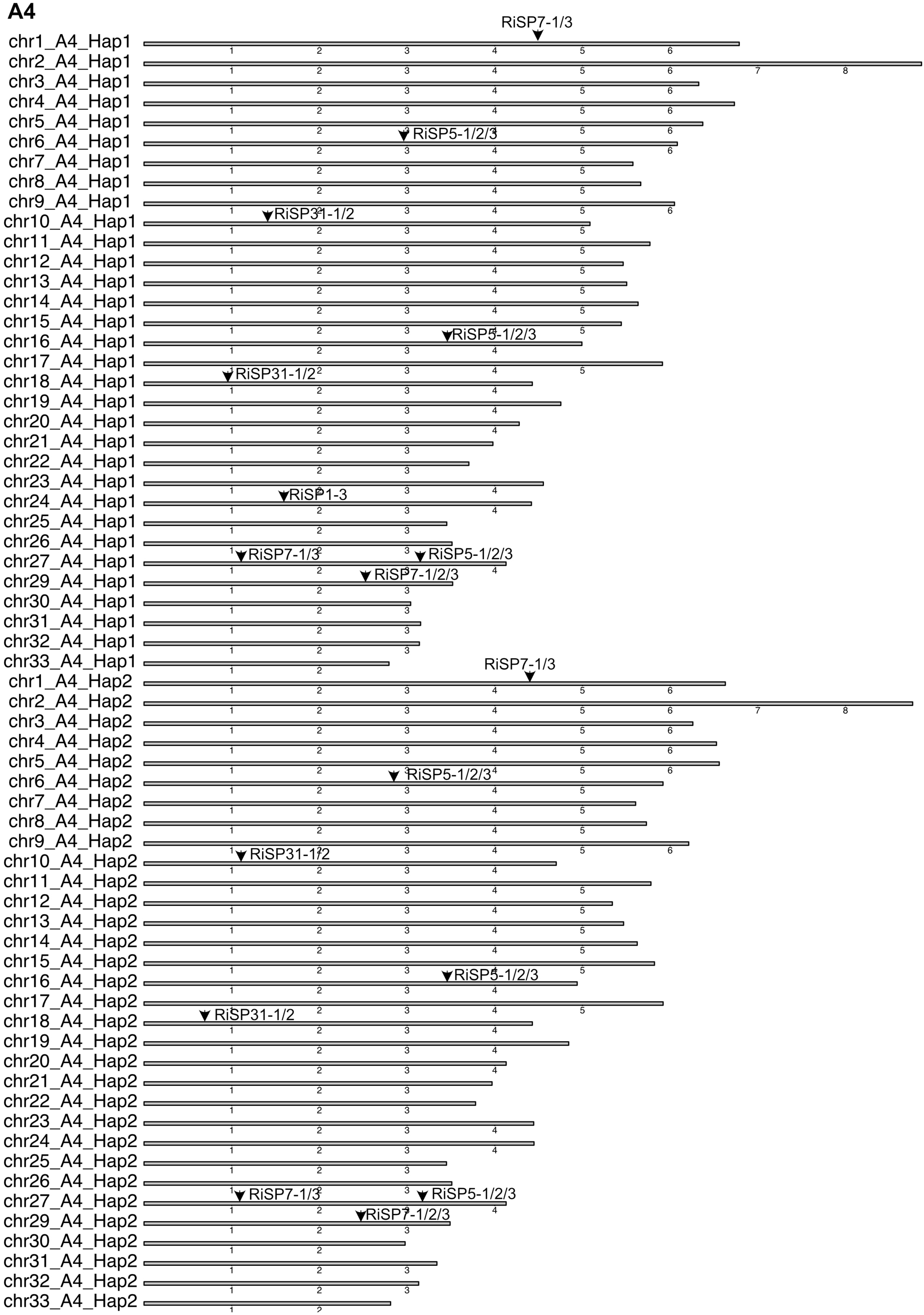
RiSP7-1/3
chr1_A4_Hap1
chr2_A4_Hap1
chr3_A4_Hap1
chr4_A4_Hap1
chr5_A4_Hap1
RiSP5-1/2/3
chr6_A4_Hap1
chr7_A4_Hap1
chr8_A4_Hap1
chr9_A4_Hap1
RiSP31-1/2
chr10_A4_Hap1
chr11_A4_Hap1
chr12_A4_Hap1
chr13_A4_Hap1
chr14_A4_Hap1
chr15_A4_Hap1
RiSP5-1/2/3
chr16_A4_Hap1
chr17_A4_Hap1
RiSP31-1/2
chr18_A4_Hap1
chr19_A4_Hap1
chr20_A4_Hap1
chr21_A4_Hap1
chr22_A4_Hap1
chr23_A4_Hap1
RiSP1-3
chr24_A4_Hap1
chr25_A4_Hap1
chr26_A4_Hap1
RiSP7-1/3
RiSP5-1/2/3
chr27_A4_Hap1
RiSP7-1/2/3
chr29_A4_Hap1
chr30_A4_Hap1
chr31_A4_Hap1
chr32_A4_Hap1
chr33_A4_Hap1
RiSP7-1/3
chr1_A4_Hap2
chr2_A4_Hap2
chr3_A4_Hap2
chr4_A4_Hap2
chr5_A4_Hap2
RiSP5-1/2/3
chr6_A4_Hap2
chr7_A4_Hap2
chr8_A4_Hap2
chr9_A4_Hap2
RiSP31-1/2
chr10_A4_Hap2
chr11_A4_Hap2
chr12_A4_Hap2
chr13_A4_Hap2
chr14_A4_Hap2
chr15_A4_Hap2
RiSP5-1/2/3
chr16_A4_Hap2
chr17_A4_Hap2
RiSP31-1/2
chr18_A4_Hap2
chr19_A4_Hap2
chr20_A4_Hap2
chr21_A4_Hap2
chr22_A4_Hap2
chr23_A4_Hap2
chr24_A4_Hap2
chr25_A4_Hap2
chr26_A4_Hap2
RiSP7-1/3
RiSP5-1/2/3
chr27_A4_Hap2
RiSP7-1/2/3
chr29_A4_Hap2
chr30_A4_Hap2
chr31_A4_Hap2
chr32_A4_Hap2
chr33_A4_Hap2

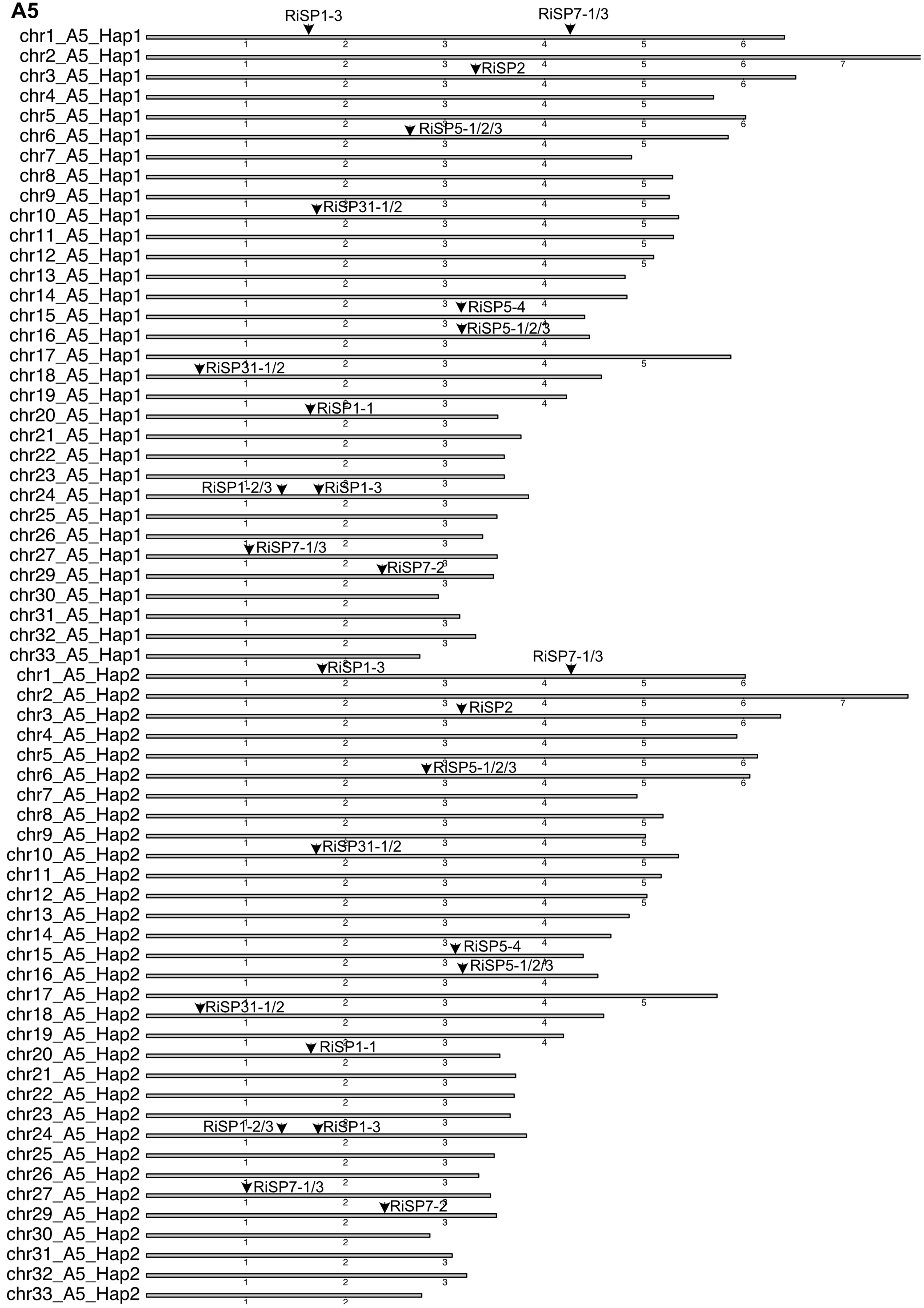
A5
RiSP1-3
RiSP7-1/3
chr1_A5_Hap1
chr2_A5_Hap1
RiSP2
chr3_A5_Hap1
chr4_A5_Hap1
chr5_A5_Hap1
RiSP5-1/2/3
chr6_A5_Hap1
chr7_A5_Hap1
chr8_A5_Hap1
chr9_A5_Hap1
RiSP31-1/2
chr10_A5_Hap1
chr11_A5_Hap1
chr12_A5_Hap1
chr13_A5_Hap1
chr14_A5_Hap1
RiSP5-4
chr15_A5_Hap1
RiSP5-1/2/3
chr16_A5_Hap1
chr17_A5_Hap1
RiSP31-1/2
chr18_A5_Hap1
chr19_A5_Hap1
RiSP1-1
chr20_A5_Hap1
chr21_A5_Hap1
chr22_A5_Hap1
chr23_A5_Hap1
RiSP1-2/3
RiSP1-3
chr24_A5_Hap1
chr25_A5_Hap1
chr26_A5_Hap1
RiSP7-1/3
chr27_A5_Hap1
RiSP7-2
chr29_A5_Hap1
chr30_A5_Hap1
chr31_A5_Hap1
chr32_A5_Hap1
chr33_A5_Hap1
RiSP1-3
RiSP7-1/3
chr1_A5_Hap2
chr2_A5_Hap2
RiSP2
chr3_A5_Hap2
chr4_A5_Hap2
chr5_A5_Hap2
RiSP5-1/2/3
chr6_A5_Hap2
chr7_A5_Hap2
chr8_A5_Hap2
chr9_A5_Hap2
RiSP31-1/2
chr10_A5_Hap2
chr11_A5_Hap2
chr12_A5_Hap2
chr13_A5_Hap2
chr14_A5_Hap2
RiSP5-4
chr15_A5_Hap2
RiSP5-1/2/3
chr16_A5_Hap2
chr17_A5_Hap2
RiSP31-1/2
chr18_A5_Hap2
chr19_A5_Hap2
RiSP1-1
chr20_A5_Hap2
chr21_A5_Hap2
chr22_A5_Hap2
chr23_A5_Hap2
RiSP1-2/3
RiSP1-3
chr24_A5_Hap2
chr25_A5_Hap2
chr26_A5_Hap2
RiSP7-1/3
chr27_A5_Hap2
RiSP7-2
chr29_A5_Hap2
chr30_A5_Hap2
chr31_A5_Hap2
chr32_A5_Hap2
chr33_A5_Hap2

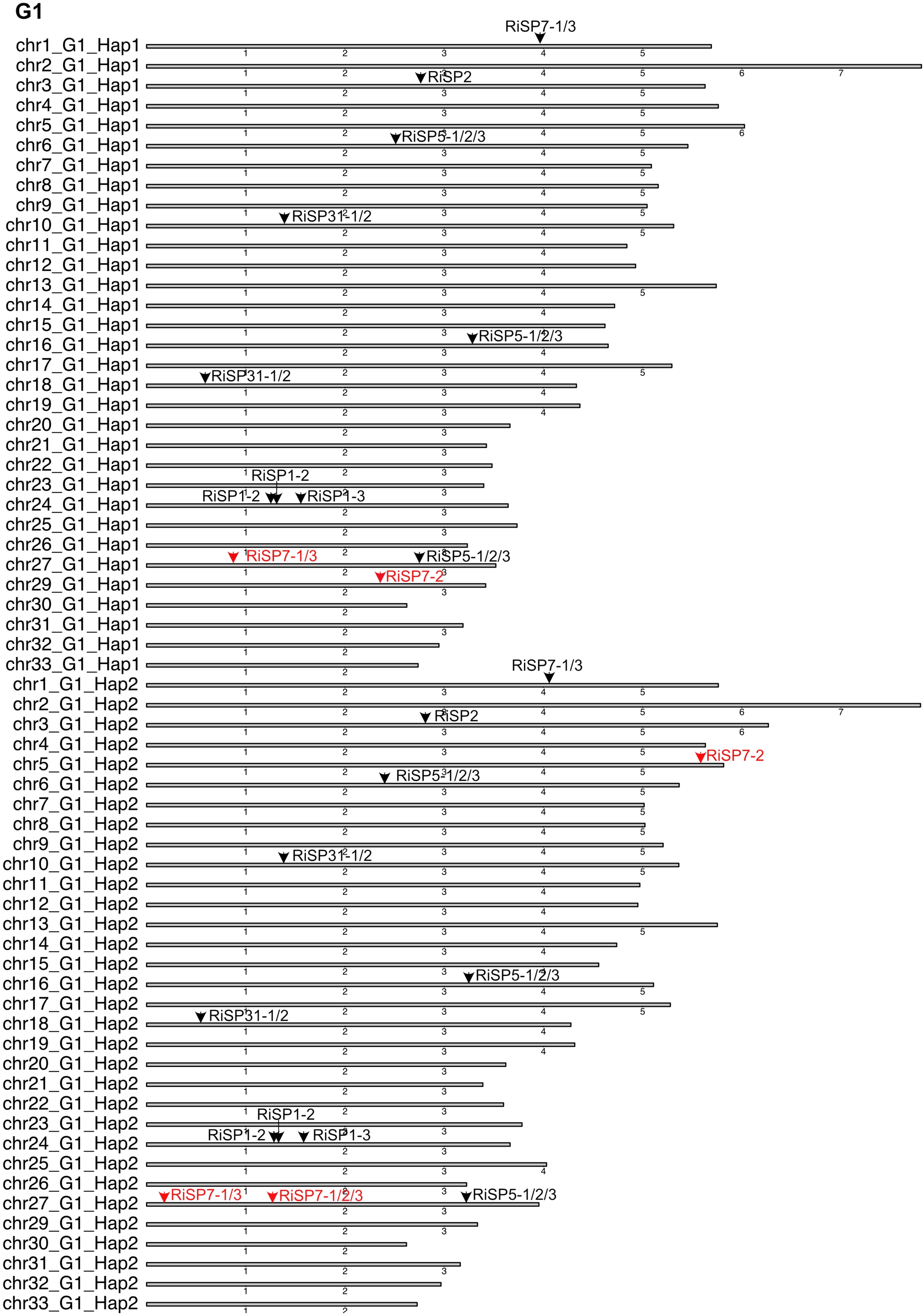

G1
chr1_G1_Hap1
RiSP7-1/3
chr2_G1_Hap1
chr3_G1_Hap1
RiSP2
chr4_G1_Hap1
chr5_G1_Hap1
chr6_G1_Hap1
RiSP5-1/2/3
chr7_G1_Hap1
chr8_G1_Hap1
chr9_G1_Hap1
chr10_G1_Hap1
RiSP31-1/2
chr11_G1_Hap1
chr12_G1_Hap1
chr13_G1_Hap1
chr14_G1_Hap1
chr15_G1_Hap1
chr16_G1_Hap1
RiSP5-1/2/3
chr17_G1_Hap1
chr18_G1_Hap1
RiSP31-1/2
chr19_G1_Hap1
chr20_G1_Hap1
chr21_G1_Hap1
chr22_G1_Hap1
chr23_G1_Hap1
RiSP1-2
chr24_G1_Hap1
RiSP1-2
RiSP1-3
chr25_G1_Hap1
chr26_G1_Hap1
chr27_G1_Hap1
RiSP7-1/3
RiSP5-1/2/3
chr29_G1_Hap1
RiSP7-2
chr30_G1_Hap1
chr31_G1_Hap1
chr32_G1_Hap1
chr33_G1_Hap1
chr1_G1_Hap2
RiSP7-1/3
chr2_G1_Hap2
chr3_G1_Hap2
RiSP2
chr4_G1_Hap2
chr5_G1_Hap2
RiSP7-2
chr6_G1_Hap2
RiSP5-1/2/3
chr7_G1_Hap2
chr8_G1_Hap2
chr9_G1_Hap2
chr10_G1_Hap2
RiSP31-1/2
chr11_G1_Hap2
chr12_G1_Hap2
chr13_G1_Hap2
chr14_G1_Hap2
chr15_G1_Hap2
chr16_G1_Hap2
RiSP5-1/2/3
chr17_G1_Hap2
chr18_G1_Hap2
RiSP31-1/2
chr19_G1_Hap2
chr20_G1_Hap2
chr21_G1_Hap2
chr22_G1_Hap2
chr23_G1_Hap2
RiSP1-2
chr24_G1_Hap2
RiSP1-2
RiSP1-3
chr25_G1_Hap2
chr26_G1_Hap2
chr27_G1_Hap2
RiSP7-1/3
RiSP7-1/2/3
RiSP5-1/2/3
chr29_G1_Hap2
chr30_G1_Hap2
chr31_G1_Hap2
chr32_G1_Hap2
chr33_G1_Hap2

SL1

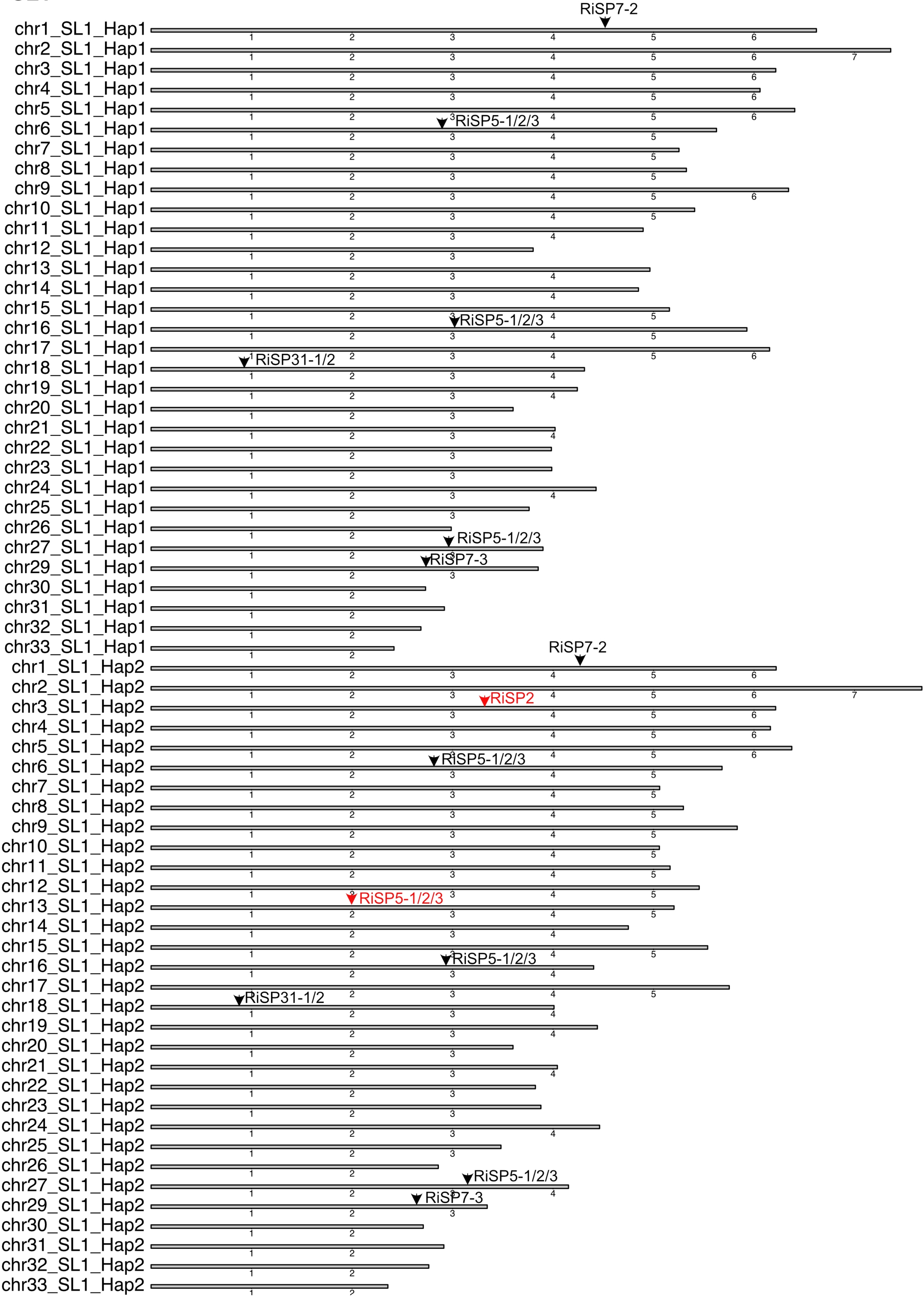
RiSP7-2
chr1_SL1_Hap1
chr2_SL1_Hap1
chr3_SL1_Hap1
chr4_SL1_Hap1
chr5_SL1_Hap1
RiSP5-1/2/3
chr6_SL1_Hap1
chr7_SL1_Hap1
chr8_SL1_Hap1
chr9_SL1_Hap1
chr10_SL1_Hap1
chr11_SL1_Hap1
chr12_SL1_Hap1
chr13_SL1_Hap1
chr14_SL1_Hap1
chr15_SL1_Hap1
RiSP5-1/2/3
chr16_SL1_Hap1
chr17_SL1_Hap1
RiSP31-1/2
chr18_SL1_Hap1
chr19_SL1_Hap1
chr20_SL1_Hap1
chr21_SL1_Hap1
chr22_SL1_Hap1
chr23_SL1_Hap1
chr24_SL1_Hap1
chr25_SL1_Hap1
chr26_SL1_Hap1
RiSP5-1/2/3
chr27_SL1_Hap1
RiSP7-3
chr29_SL1_Hap1
chr30_SL1_Hap1
chr31_SL1_Hap1
chr32_SL1_Hap1
chr33_SL1_Hap1
RiSP7-2
chr1_SL1_Hap2
chr2_SL1_Hap2
RiSP2
chr3_SL1_Hap2
chr4_SL1_Hap2
chr5_SL1_Hap2
RiSP5-1/2/3
chr6_SL1_Hap2
chr7_SL1_Hap2
chr8_SL1_Hap2
chr9_SL1_Hap2
chr10_SL1_Hap2
chr11_SL1_Hap2
chr12_SL1_Hap2
RiSP5-1/2/3
chr13_SL1_Hap2
chr14_SL1_Hap2
chr15_SL1_Hap2
RiSP5-1/2/3
chr16_SL1_Hap2
chr17_SL1_Hap2
RiSP31-1/2
chr18_SL1_Hap2
chr19_SL1_Hap2
chr20_SL1_Hap2
chr21_SL1_Hap2
chr22_SL1_Hap2
chr23_SL1_Hap2
chr24_SL1_Hap2
chr25_SL1_Hap2
chr26_SL1_Hap2
RiSP5-1/2/3
chr27_SL1_Hap2
RiSP7-3
chr29_SL1_Hap2
chr30_SL1_Hap2
chr31_SL1_Hap2
chr32_SL1_Hap2
chr33_SL1_Hap2

GCA_020716765.1_ASM2071676v1

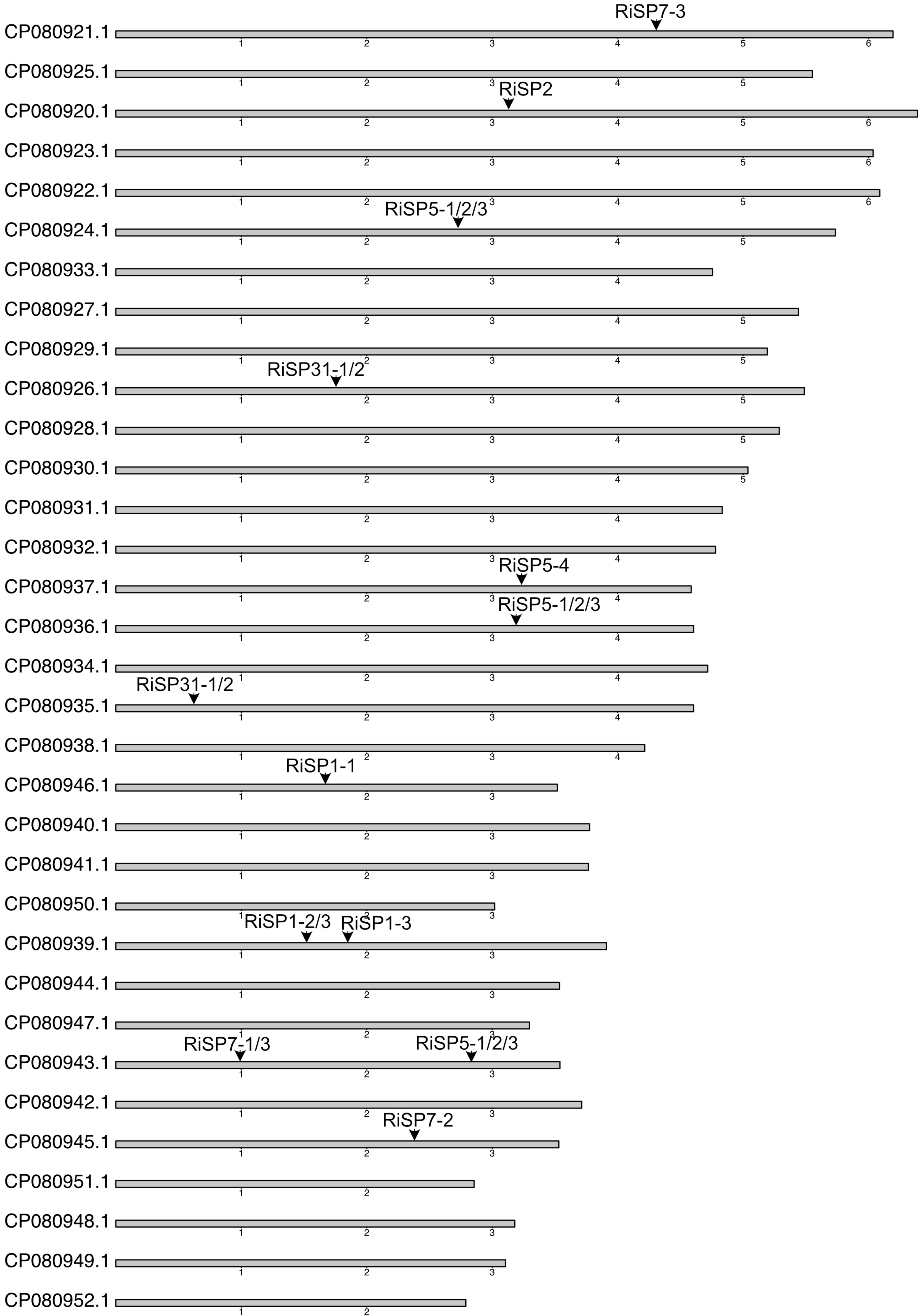

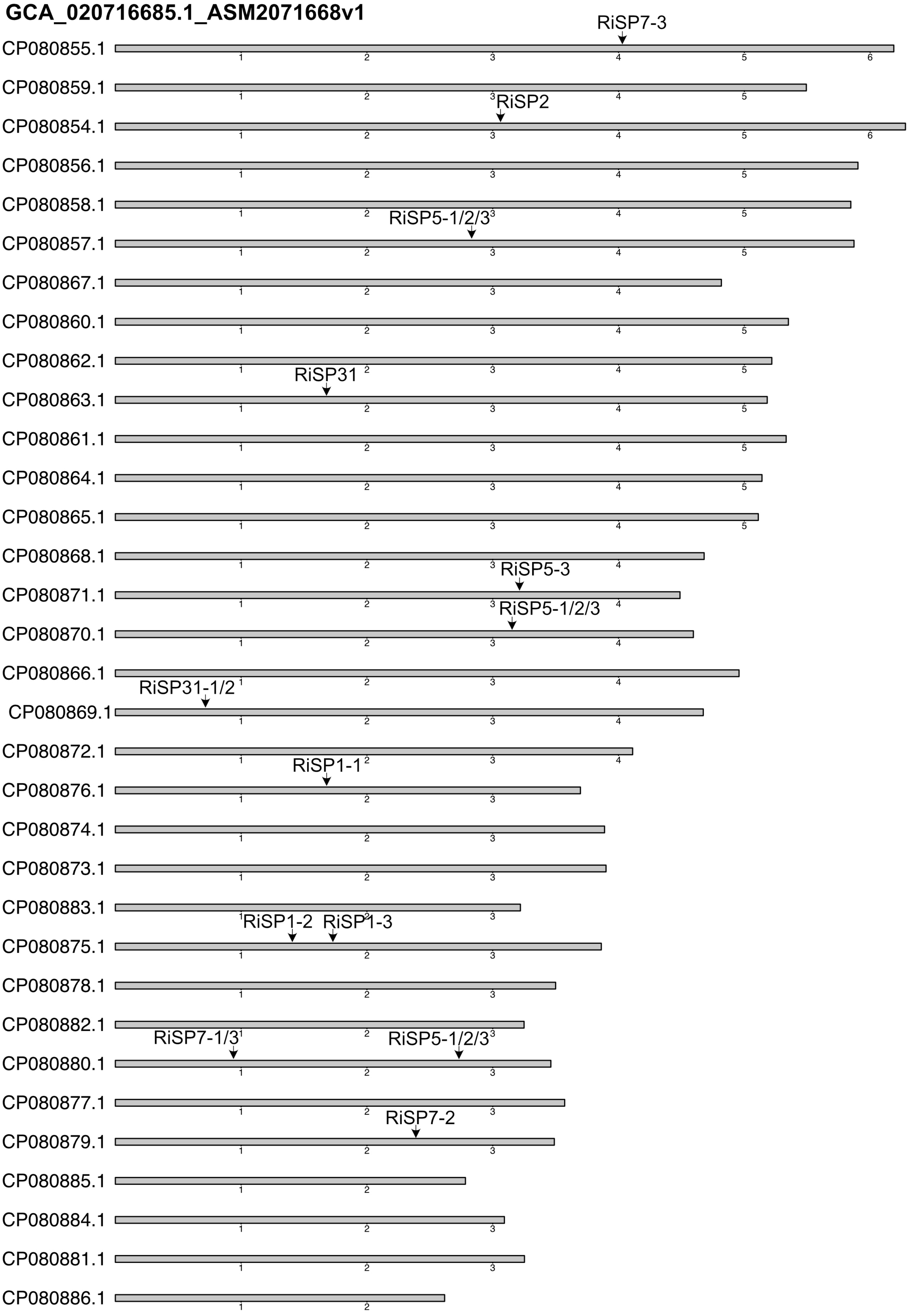
GCA_020716685.1_ASM2071668v1
CP080855.1
RiSP7-3
CP080859.1
CP080854.1
RiSP2
CP080856.1
CP080858.1
CP080857.1
RiSP5-1/2/3
CP080867.1
CP080860.1
CP080862.1
CP080863.1
RiSP31
CP080861.1
CP080864.1
CP080865.1
CP080868.1
CP080871.1
RiSP5-3
CP080870.1
RiSP5-1/2/3
CP080866.1
CP080869.1
RiSP31-1/2
CP080872.1
CP080876.1
RiSP1-1
CP080874.1
CP080873.1
CP080883.1
CP080875.1
RiSP1-2
RiSP1-3
CP080878.1
CP080882.1
CP080880.1
RiSP7-1/3
RiSP5-1/2/3
CP080877.1
CP080879.1
RiSP7-2
CP080885.1
CP080884.1
CP080881.1
CP080886.1

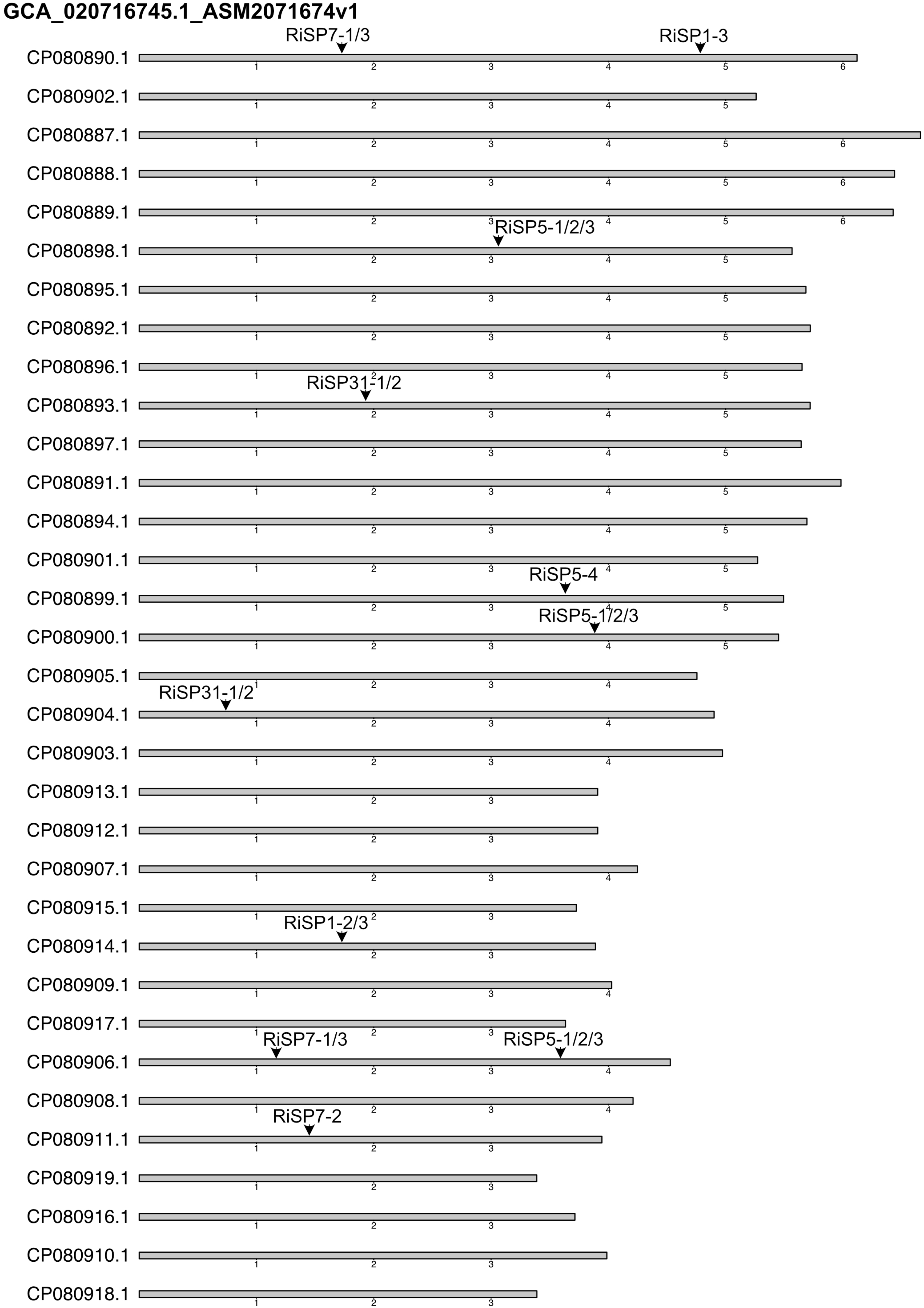

GCA_020716745.1_ASM2071674v1
CP080890.1
RiSP7-1/3
RiSP1-3
CP080902.1
CP080887.1
CP080888.1
CP080889.1
CP080898.1
RiSP5-1/2/3
CP080895.1
CP080892.1
CP080896.1
CP080893.1
RiSP31-1/2
CP080897.1
CP080891.1
CP080894.1
CP080901.1
CP080899.1
RiSP5-4
CP080900.1
RiSP5-1/2/3
CP080905.1
CP080904.1
RiSP31-1/2
CP080903.1
CP080913.1
CP080912.1
CP080907.1
CP080915.1
CP080914.1
RiSP1-2/3
CP080909.1
CP080917.1
CP080906.1
RiSP7-1/3
RiSP5-1/2/3
CP080908.1
CP080911.1
RiSP7-2
CP080919.1
CP080916.1
CP080910.1
CP080918.1

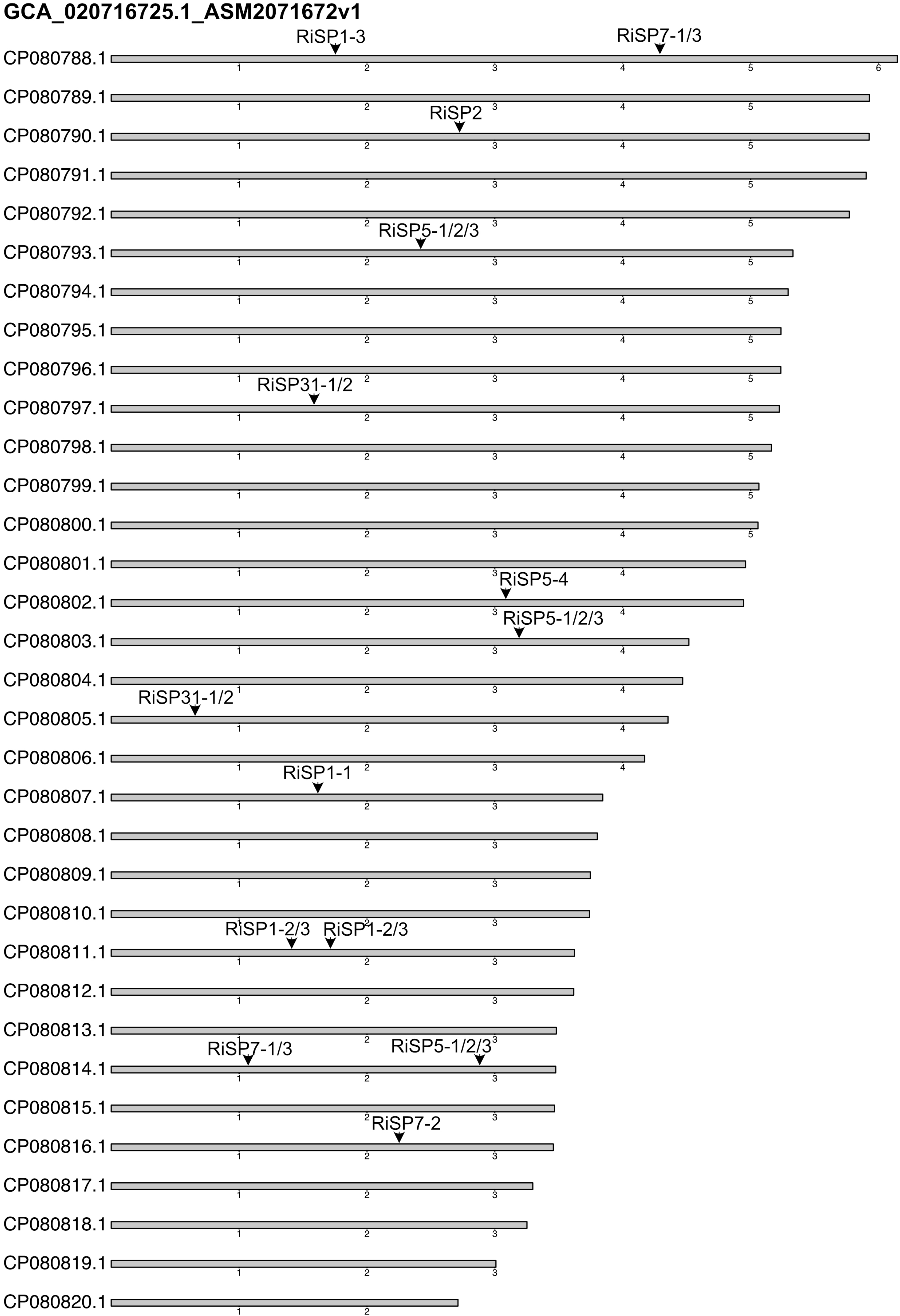
GCA_020716725.1_ASM2071672v1
CP080788.1
RiSP1-3
RiSP7-1/3
CP080789.1
CP080790.1
RiSP2
CP080791.1
CP080792.1
CP080793.1
RiSP5-1/2/3
CP080794.1
CP080795.1
CP080796.1
CP080797.1
RiSP31-1/2
CP080798.1
CP080799.1
CP080800.1
CP080801.1
CP080802.1
RiSP5-4
CP080803.1
RiSP5-1/2/3
CP080804.1
CP080805.1
RiSP31-1/2
CP080806.1
CP080807.1
RiSP1-1
CP080808.1
CP080809.1
CP080810.1
CP080811.1
RiSP1-2/3
RiSP1-2/3
CP080812.1
CP080813.1
CP080814.1
RiSP7-1/3
RiSP5-1/2/3
CP080815.1
CP080816.1
RiSP7-2
CP080817.1
CP080818.1
CP080819.1
CP080820.1

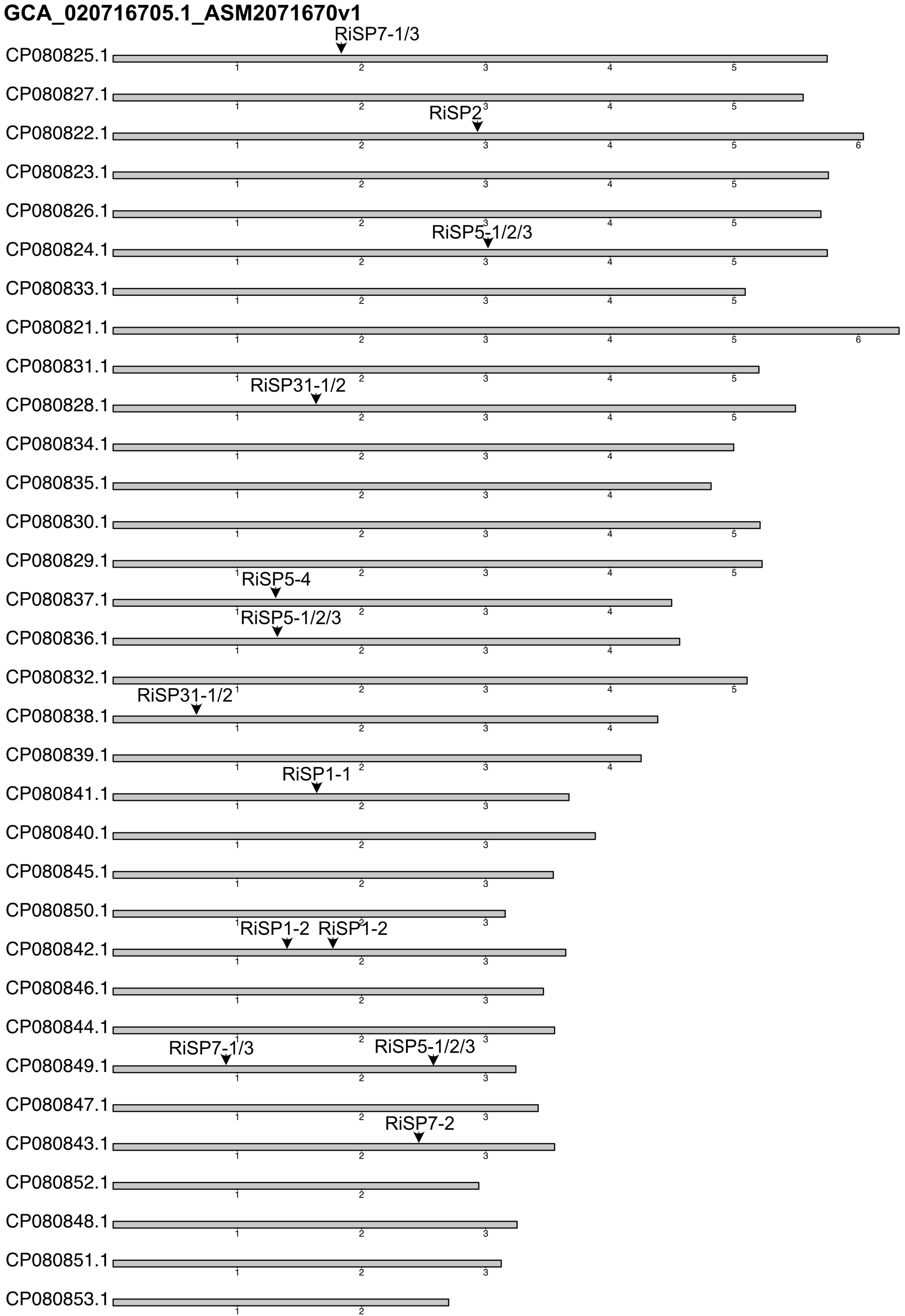
GCA_020716705.1_ASM2071670v1
CP080825.1
RiSP7-1/3
CP080827.1
CP080822.1
RiSP2
CP080823.1
CP080826.1
CP080824.1
RiSP5-1/2/3
CP080833.1
CP080821.1
CP080831.1
CP080828.1
RiSP31-1/2
CP080834.1
CP080835.1
CP080830.1
CP080829.1
CP080837.1
RiSP5-4
CP080836.1
RiSP5-1/2/3
CP080832.1
CP080838.1
RiSP31-1/2
CP080839.1
CP080841.1
RiSP1-1
CP080840.1
CP080845.1
CP080850.1
CP080842.1
RiSP1-2
RiSP1-2
CP080846.1
CP080844.1
CP080849.1
RiSP7-1/3
RiSP5-1/2/3
CP080847.1
CP080843.1
RiSP7-2
CP080852.1
CP080848.1
CP080851.1
CP080853.1

GCA_026210795.1_ASM2621079v1

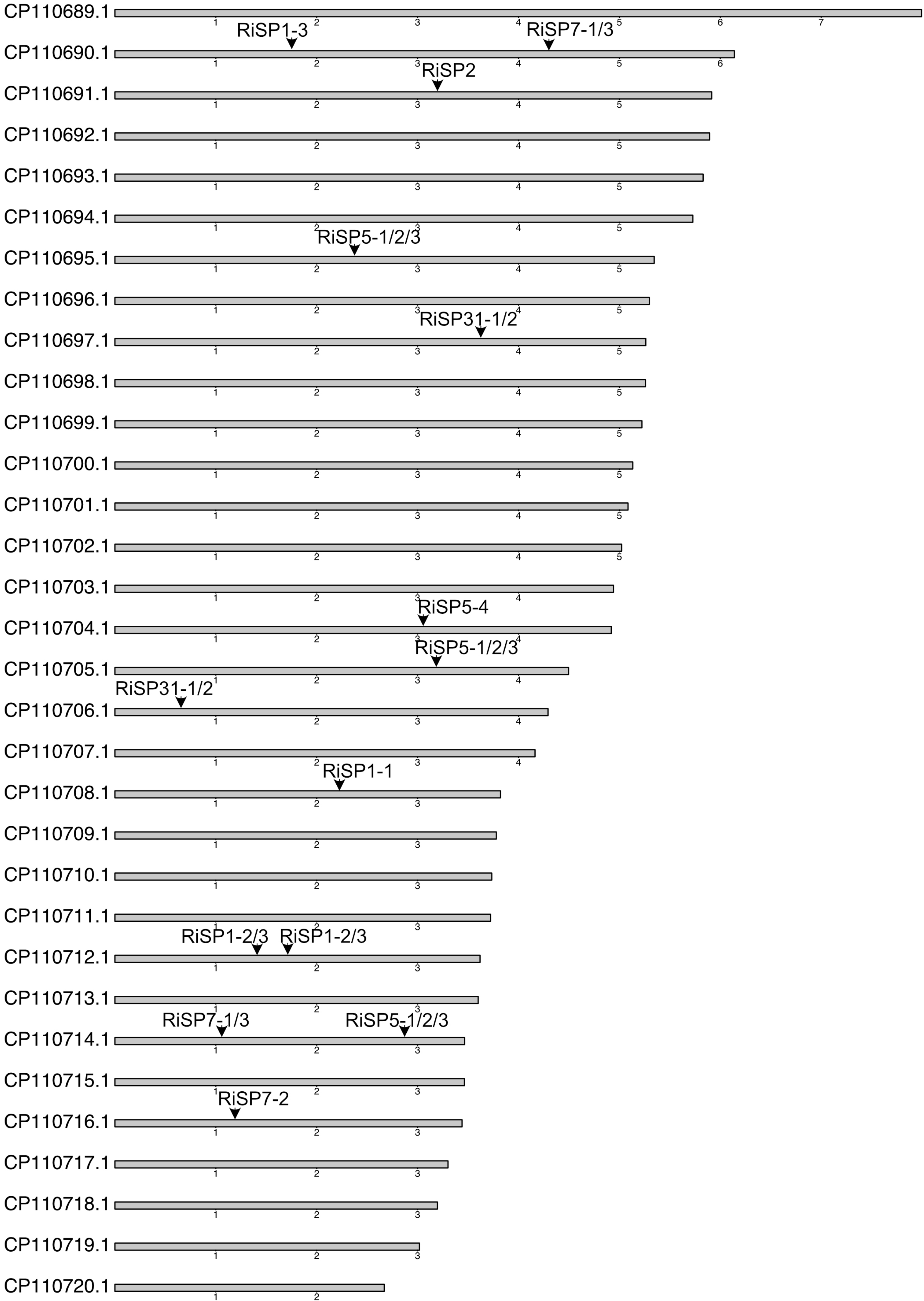

Supplement: Supplementary file 10 — Source Data [file 41467_2024_51512_MOESM10_ESM.zip › Source data R. irregularis strains all genomes.pdf]
